# Parkinson’s disease-associated *PINK1* loss disrupts ensheathing glia and causes dopaminergic neuron synapse loss

**DOI:** 10.7554/eLife.105386

**Published:** 2026-08-13

**Authors:** Lorenzo Ghezzi, Sabine Kuenen, Ulrike Pech, Nils Schoovaerts, Ayse Kilic, Suresh Poovathingal, Kristofer Davie, Jochen Lamote, Roman Praschberger, Patrik Verstreken

**Affiliations:** 1 VIB-KU Leuven Center for Neuroscience Leuven Belgium; 2 KU Leuven, Department of Neurosciences, Leuven Brain Institute Leuven Belgium; 3 VIB-KU Leuven Center for Neuroscience, Single Cell and Microfluidics Expertise Unit Leuven Belgium; 4 VIB-KU Leuven Center for Neuroscience, Single Cell Bioinformatics Unit Leuven Belgium; 5 VIB Flow Core Leuven, VIB Technologies Leuven Belgium; 6 Medical University of Innsbruck, Institute of Human Genetics Innsbruck Austria; Baylor College of Medicine United States; Tata Institute for Genetics and Society India

**Keywords:** Parkinson's disease, glial cell, neuron-glia interactions, Pink1, *D. melanogaster*

## Abstract

Parkinson’s disease (PD) is commonly associated with the loss of dopaminergic neurons in the *substantia nigra*, but many other cell types are affected even before neuron loss occurs. Recent studies have linked oligodendrocytes to early stages of PD, though their precise role is still unclear. *PINK1* is mutated in familial PD, and through unbiased single-cell sequencing of the entire brain of *Drosophila Pink1* models, we observed significant gene deregulation in ensheathing glia (EG), cells that share functional similarities with oligodendrocytes. We found that the loss of *Pink1* leads to abnormalities in EG, similar to the reactive response of EG seen upon nerve injury. Using cell-type-specific transcriptomics, we identified deregulated genes in EG as potential functional modifiers. Specifically downregulating two trafficking factors in EG, Vps35 and Vps13, also mutated in PD, was sufficient to rescue neuronal function and protect against dopaminergic synapse loss. Our findings demonstrate that *Pink1* loss in neurons triggers an injury-like response in EG, and that *Pink1* loss in EG, in turn, disrupts neuronal function. Vesicle trafficking components, which may regulate membrane interactions between organelles in EG, seem to play a role in maintaining neuronal health and ultimately preventing dopaminergic synapse loss. Our work highlights the essential role of glial support cells in the pathogenesis of PD and identifies vesicle trafficking within these cells in disease progression.

## Introduction

Parkinson’s disease (PD) is characterized by motor symptoms such as bradykinesia, rigidity, and tremor that are caused by the progressive loss of dopaminergic neurons in the substantia nigra (Lang and Lozano, 1998). However, most of the patients report non-motor symptoms, such as constipation, hyposmia, and sleep defects, even before the onset of motor symptoms (Munhoz et al., 2015). This suggests that PD is a progressive neurodegenerative disease that initiates many years before the diagnosis and involves different neuronal systems, multiple anatomical areas, and different cell types. While therapies symptomatically improve motor functioning temporarily by restoring dopaminergic tone, they do not effectively prevent neurodegeneration and disease progression. This highlights the necessity to identify cell-types and biological mechanisms that act at the earliest stages of disease.

With the advent of single-cell sequencing applied to PD and control brains in combination with evidence from genome-wide association studies (GWAS), it is now possible to identify which cells and mechanisms are at play in PD models that recapitulate early stages of PD (Kaempf et al., 2026; Pech et al., 2025). Among these, one study reported that genes related to GWAS loci are enriched for oligodendrocyte-specific gene expression (Bryois et al., 2020). Interestingly, differential gene expression (DGE) between post-mortem brains with Braak scores of 1–2, 3–4, and 5–6 and controls suggested a role for oligodendrocytes at an early stage of PD prior to the overt loss of dopaminergic neurons (Bryois et al., 2020). Additionally, two independent single-cell sequencing studies, one of the human substantia nigra and one of the midbrain, both associated PD genetic risk with oligodendrocyte-specific expression patterns (Agarwal et al., 2020; Smajić et al., 2022). While these studies started to link dysfunction of oligodendrocytes to the early stages of PD, they did not address their role in the pathophysiology of the disease.

Here, we describe a *Drosophila* model to start assessing how neuron-glia cross-talk contributes to PD. *Drosophila* has been successfully used to investigate cellular and molecular dysfunction preceding the onset of age-related symptoms, including dopaminergic neuron-dependent motor symptoms (Kaempf et al., 2026; Pech et al., 2025; Valadas et al., 2018). Furthermore, *Drosophila* glial cells display several anatomical and functional features that show remarkable similarity to their mammalian counterparts (Freeman and Doherty, 2006; Kremer et al., 2017). While the small size of the *Drosophila* brain does not necessitate elaborate myelination, ensheathing glia (EG) in this species share not only anatomical features with oligodendrocytes, such as the ability to wrap around nerve tracts in the central nervous system (Kremer et al., 2017; Yildirim et al., 2019), but also important molecular and functional features, such as providing metabolic support and regulating neuronal activity (Delgado et al., 2018; Otto et al., 2018).

In a brain-wide single-cell sequencing experiment, we identified EG as the most deregulated cell type in a young (pre-motor) *Pink1* loss-of-function *Drosophila* model. Using cell-specific labeling, immunohistochemistry, and electrophysiological recordings, we correlated the transcriptional deregulation in EG with changes that appear similar to those seen upon nerve injury. Additionally, we find that healthy EG are fundamental to support dopaminergic synapse integrity in *Pink1* mutants. Finally, using cell-type-specific transcriptomic, high-throughput screening, and immunohistochemistry, we identified vesicle trafficking as a genetic modifier and showed that *Vps13* and *Vps35* are EG-expressed regulators that maintain dopaminergic synapse integrity. Our study shows that neuron-EG cross-talk is already disrupted at a young age by the loss of *Pink1* in neurons, but also in EG, and that EG are fundamental to support dopaminergic synapse integrity, suggesting a role for oligodendrocytes in the progression of PD.

## Results

### EG in Pink1 loss-of-function flies show cell non-autonomous response

**Figure 1.**
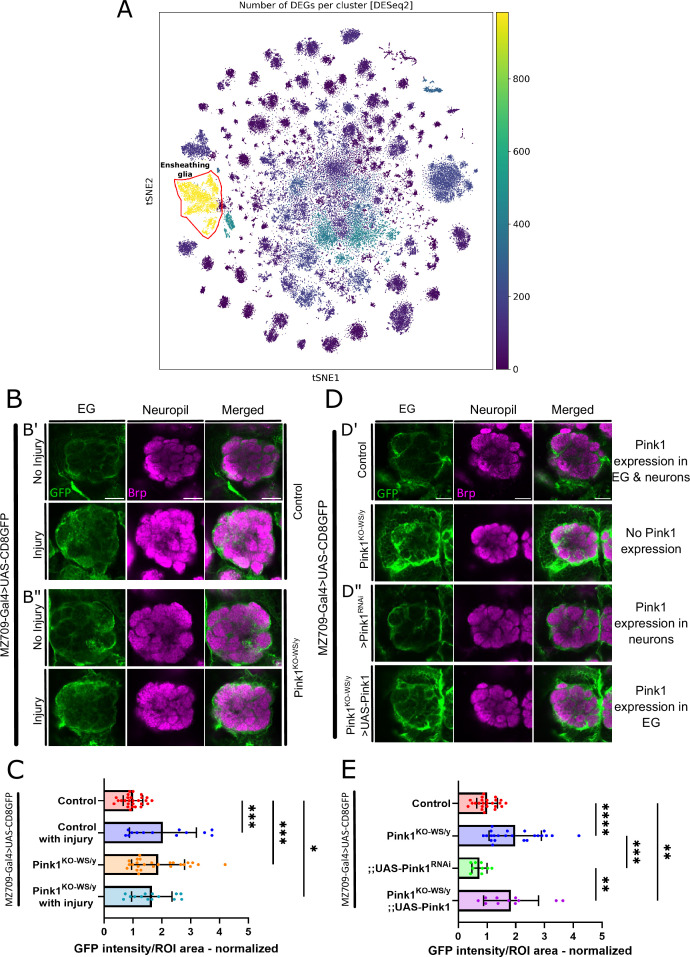
Ensheathing glia (EG) are affected non-cell autonomously by *Pink1* loss-of-function (with Figure 1—figure supplement 1). (**A**) tSNE of the cells of *Pink1^P399L^* knock-in mutants (5-day-old). Cell types are labeled with colors, indicating the number of deregulated genes compared to control. EG are encircled and labeled. (**B–B”**) Maximum intensity projections of confocal images of fly brains (5±1-day-old) stained with anti-GFP (green) and anti-Brp (magenta), where anti-GFP marks EG and anti-Brp marks presynaptic sites of the antennal lobes in flies where CD8GFP is expressed via the EG driver MZ709-Gal4. Scale bar: 20 µm. (**B’**) Maximum intensity projection of confocal images of controls vs. controls 24 hr after olfactory receptor neuron (ORN)-severing (injury). (**B”**) Maximum intensity projection of confocal images of *Pink1^KO-WS/y^* vs. *Pink1^KO-WS/y^* 24 hr after ORNs severing (injury). (**C**) Quantification of GFP intensity within the glomeruli of the antennal lobe area, region of interest (ROI), in 5±1-day-old flies (as in B) relative to controls. ANOVA with Dunnett’s multiple comparison test, * is p<0.05, *** is p<0.001. Effect size: η^2^=0.23. Bars: mean ± SD; points are individual animals, N≥13 per genotype, 4 replicates. (**D–D”**) Maximum intensity projection of confocal images of fly brains (5±1-day-old) stained with anti-GFP (green) and anti-Brp (magenta), where anti-GFP marks EG and anti-Brp marks presynaptic sites of the antennal lobes in flies where CD8GFP is expressed via the EG driver MZ709-Gal4. Scale bar: 20 µm. (**D’**) Maximum intensity projection of confocal images of control (*w^1118^*) and *Pink1^KO-WS/y^* animals. (**D”**) Maximum intensity projection of confocal images of animals with *Pink1* downregulation in EG and *Pink1^KO-WS/y^* with *Pink1* rescued in EG. (**E**) Quantification of GFP intensity within the glomeruli of the antennal lobe area in 5-day-old flies (as in D) relative to controls. ANOVA with Tukey’s multiple comparison test, ** is p<0.01, *** is p<0.001. **** is p<0.0001. Effect size: η^2^=0.36. Bars: mean ± SD; points are individual animals, N≥10 per genotype, 4 replicates.

We previously created a whole-brain single-cell RNAseq dataset from different *Drosophila* PD knock-in models (Pech et al., 2025). This dataset was generated from young 5-day-old flies to capture ‘early’ changes related to PD pathophysiology. We re-analyzed the data from *Pink1^P399L^* knock-in loss-of-function mutant flies and isogenic controls and performed differentially expressed gene (DEG) analysis for each cell type using DESeq2 (Figure 1A). Glial cell subtypes show the highest degree of transcriptional deregulation; particularly, EG are strongly affected (Figure 1A). While flies do not myelinate neurons, EG serve similar supporting functions as oligodendrocytes (Otto et al., 2018; Yildirim et al., 2019). These results suggest that EG are deregulated in young *Pink1^P399L^* mutants (and also *Pink1^KO-WS/y^*, see below) at an age prior to dopaminergic neuron-dependent motor defects (Kaempf et al., 2026; Pech et al., 2025).

**Figure 1—figure supplement 1.**
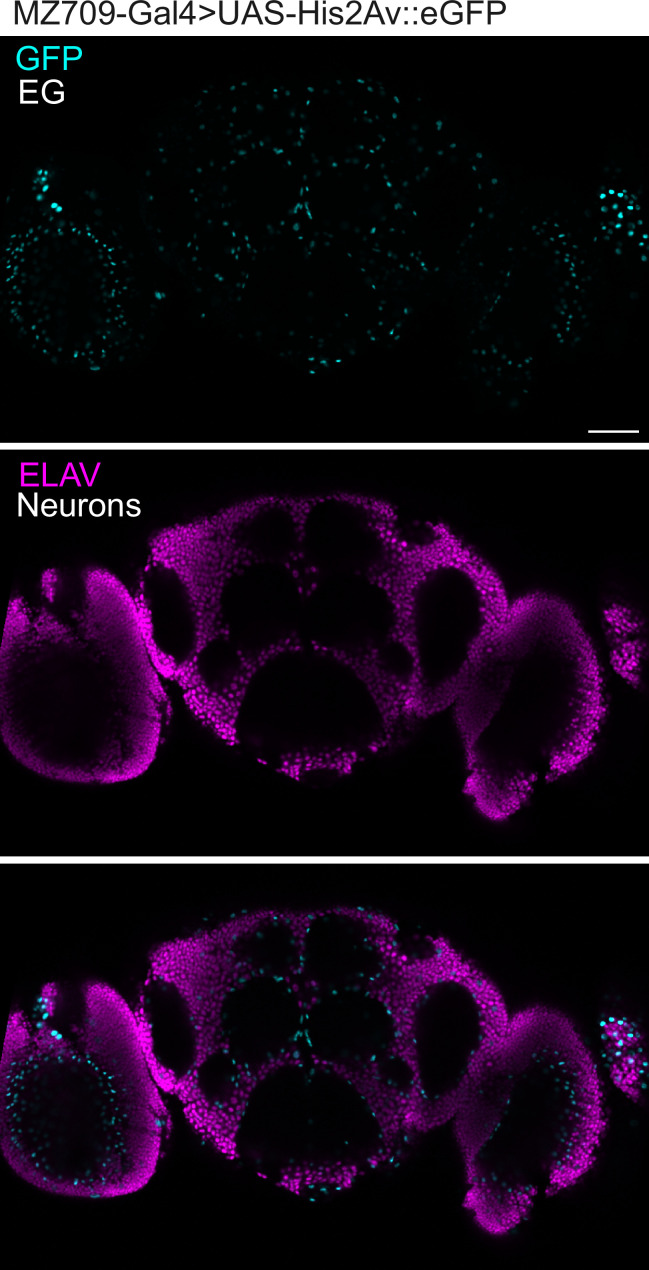
Representative confocal image of a 5±1-day-old MZ709-Gal4>UAS-His2Av::eGFP brain stained with anti-GFP (cyan) and anti-ELAV (magenta). Scale bar: 50 µm; N=3, 1 replicate.

When neurons are damaged, activated EG invade the neuropil (Doherty et al., 2009; MacDonald et al., 2006). For example, when fly antennal lobes are removed, severing the olfactory receptor neurons (ORNs), EG invade the antennal lobe neuropil (MacDonald et al., 2006), potentially as a protective response (Figure 1B’–C). To test if EG invaded the neuropil in *Pink1* mutant flies, we expressed a transmembrane fluorescent protein (UAS-CD8GFP) under the control of a promoter specific to EG (*MZ709-Gal4*) (Doherty et al., 2009, Figure 1—figure supplement 1) in control and *Pink1^KO-WS/y^* flies. Samples were labeled with anti-GFP and the pre-synaptic protein Bruchpilot (NC82). Similar to ORN-severed controls, GFP signal, lining the membrane of EG, is visible inside the antennal neuropil in *Pink1^KO-WS/y^*, and severing the ORN in *Pink1^KO-WS/y^* does not further exacerbate this phenotype (Figure 1B”–C). Even though we cannot exclude that in *Pink1* mutants the GFP signal is merely upregulated, *Pink1* loss and neuron-severing that triggers EG activation appear to display a similar neuropil-invasion phenotype.

In order to determine whether this phenotype is cell-autonomous, we generated cell-type-specific *Pink1* perturbations. We either downregulated *Pink1* using a previously well-validated RNAi line specifically in EG (EG-specific *Pink1* loss-of-function – Figure 1D’’), or we re-expressed wild-type *Pink1* specifically in EG in *Pink1^KO-WS/y^* flies (all cells, but the EG, loss-of-function – Figure 1D”). When *Pink1* is knocked down in EG (but present in other cells, including neurons), no additional GFP signal is detected in the neuropil (Figure 1D’’–E). However, when *Pink1* is not present in neurons and expressed in EG, the GFP is present inside the antennal neuropil (Figure 1D”–E). This suggests that *Pink1*-defective neurons activate the EG in a cell non-autonomous fashion to invade, and potentially protect, the neuropil.

### EG function supports synaptic integrity

Our finding that *Pink1^KO-WS/y^* in neurons elicits a cell non-autonomous response in EG suggested that EG might, in turn, also functionally modulate neuronal integrity in mutant flies. To test this, we made use of a readily accessible model circuit in the fly visual system with an easy electrophysiological readout, electroretinograms (ERGs). Notably, whereas our functional readout here interrogates neuronal activity in the visual system, our analysis of EG morphology was performed in the antennal lobe, a distinct brain region. We therefore make the explicit assumption that EG perform comparable functions across fly brain regions. This assumption is supported by our single-cell sequencing dataset, in which we did not detect region-specific segregation of EG into distinct clusters that could be attributed to different brain areas. The ERG waveforms are susceptible to changes in intracellular signaling, neuronal function, and synaptic transmission, and have been well characterized and amply used to assess neuronal and cellular function (Hardie and Raghu, 2001; Praschberger et al., 2023; Soukup et al., 2016; Wu and Wong, 1977). We exploited this method to understand the role of *Pink1* in EG on neuronal function and synaptic transmission in young flies.

**Figure 2.**
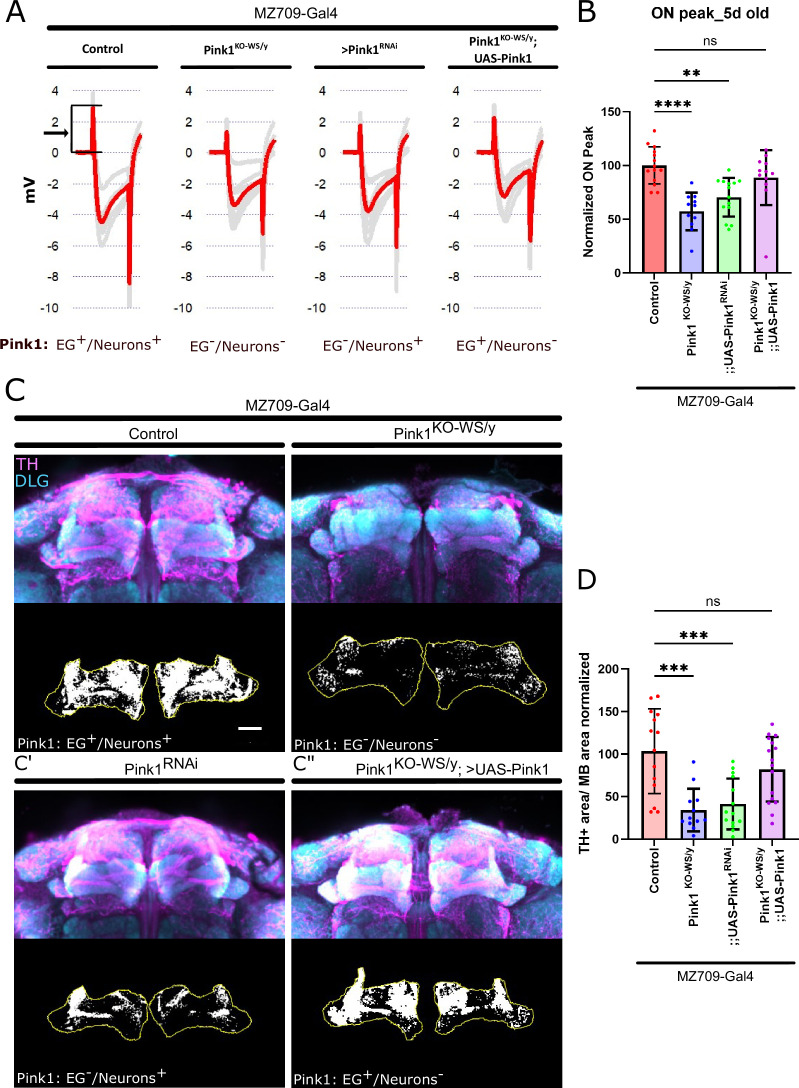
Pink1 in ensheathing glia (EG) is necessary to support synaptic integrity (with Figure 2—figure supplement 1). (**A**) Representative electroretinogram (ERG) traces of indicated genotypes: ON peak is highlighted by the arrow. (**B**) Normalized ON peak amplitude of flies (5±1-day-old). ANOVA with Tukey’s multiple comparison test, ns is p>0.05, ** is p<0.01, **** is p<0.0001. Effect size: η^2^=0.55. Bars: mean ± SD; points are individual animals, N≥11 per genotype, 3 replicates. (**C–C”**) (**C**) Maximum intensity projection of confocal images of control and *Pink1^KO-WS/y^* in mushroom bodies (MBs) of aged flies (22±2-day-old), stained with anti-TH (cyan) and anti-DLG (magenta) antibodies – DLG is used to mark post-synaptic sites of MBs. The black-and-white image is the middle Z-plane within the region of interest of the MB (region of interest [ROI], yellow), which is used to represent the thresholded TH area (white). Scale bar: 20 µm. (**C’**) Maximum intensity projection of confocal images of *w^1118^* with *Pink1* downregulation in EG. (**C”**) Maximum intensity projection of confocal images of *Pink1^KO-WS/y^* with *Pink1* rescued in EG. (**D**) Quantification of the dopaminergic synaptic area at MB neuropil in aged flies (22±2-day-old). ANOVA with Tukey’s multiple comparison test, ns is p>0.05, *** is p<0.001. Effect size: η^2^=0.38. Bars: mean ± SD; points are individual animals, N≥12 per genotype, 3 replicates.

Comparing control with young *Pink1^KO-WS/y^* flies does not show a difference in the ‘depolarization response’, indicating that – as expected – there is no neurodegeneration occurring in the photoreceptors at this stage (Figure 2A). However, when comparing the ON peak – which represents synaptic transmission from the photoreceptors and within the underlying neuronal circuit, and is a more robust readout of this than the OFF peak (Vilinsky and Johnson, 2012) – we detect a significant reduction in mutant compared to control animals, suggesting that in *Pink1^KO-WS/y^* flies, synaptic communication in this circuit is partially impaired, already in 5-day-old animals (Figure 2A and B).

**Figure 2—figure supplement 1.**
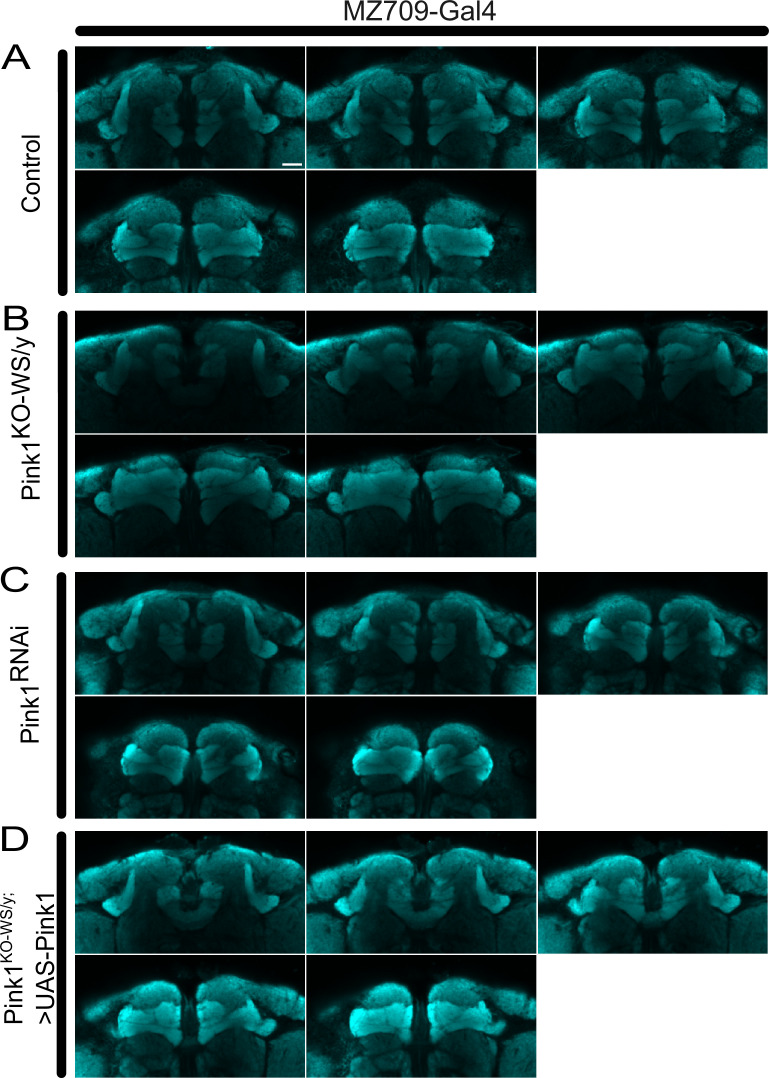
Examples of confocal Z-stacks of anti-DLG-labeled fly brains (22±2-day-old) of the indicated genotypes. Such images were used to delineate regions of interest (ROIs) to quantify DAN innervation onto the mushroom bodies (MBs). (**A**) Control (w^1118/y^;; MZ709-Gal4/+); (**B**) *Pink1^KO-WS/y^*;; MZ709-Gal4/+; (**C**) flies where Pink1 is downregulated in EG (w^1118/y^;; MZ709-Gal4/*Pink1*RNAi); (**D**) *Pink1^KO-WS/y^* flies with expression of wild-type Pink1 in EG (*Pink1^KO-WS/y^*;; MZ709-Gal4/UAS-*Pink1*). Scale bar: 20 µm.

In order to discern the role of EG in this defective ERG response, we (1) downregulated *Pink1* specifically in EG and (2) we expressed *Pink1* specifically in the EG of *Pink1^KO-WS/y^*. When *Pink1* is knocked down specifically in EG, we observe the same reduction in ON peak response as in the *Pink1^KO-WS/y^* flies. This indicates that EG integrity is necessary for efficient neurotransmission in this circuit. Conversely, the ON peak defect of *Pink1^KO-WS/y^* mutants is rescued when *Pink1* is reintroduced in EG only. This suggests that activated EG are able to, at least partially, buffer the detrimental effects of *Pink1* deficiency in neurons (Figure 2A and B).

To also test EG function in relation to PD-relevant dopaminergic (DA) synapses, we evaluated the loss of dopaminergic neuron afferents. In a previous paper from our laboratory, we have shown that the loss of *Pink1* function causes a progressive loss of protocerebral anterior medial dopaminergic neuron (PAM DAN) afferents in the mushroom body (MB) neuropil in 25-day-old animals, but not when they are 5-day-old (Kaempf et al., 2026). Indeed, we confirm that 25-day-old animals show a decrease in the dopaminergic synaptic area in MB lobes (Figure 2C and D). Hence, while EG are already activated at a young age in *Pink1^KO-WS/y^*, DA synapses are structurally still intact (at 5 days of age) and only deteriorate in older flies. We then knocked down *Pink1* specifically in EG and found a similar strong decrease in DA synaptic area as compared to global *Pink*1*^KO^* (Figure 2C’–D). Conversely, when *Pink1* is reintroduced specifically in EG of *Pink1^KO-WS/y^* animals, DA synapse loss is significantly rescued (Figure 2C”–D, Figure 2—figure supplement 1). Together, these results indicate that *Pink1* in EG is necessary to protect dopaminergic neurons from synapse loss.

### EG cell-type-specific transcriptomics reveals modifiers of neuronal dysfunction

**Figure 3.**
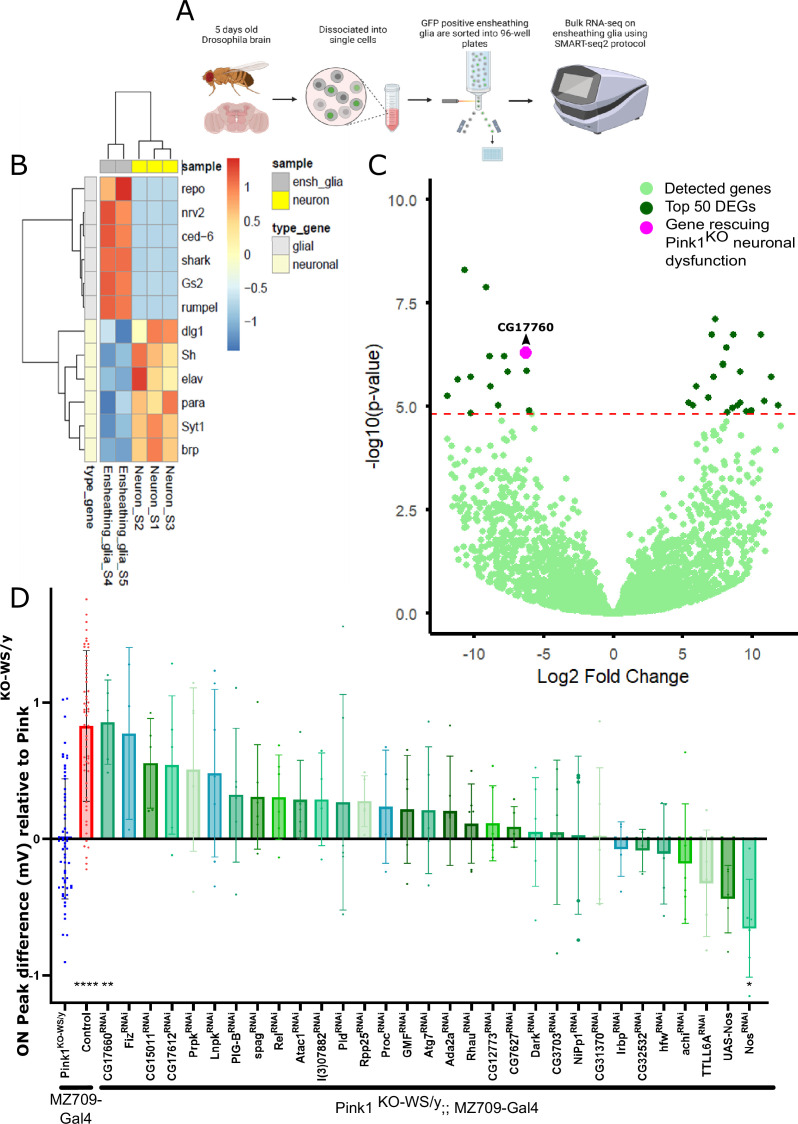
Cell-type-specific transcriptomics reveals modifiers of neuronal dysfunction. (**A**) Scheme of cell-type-specific transcriptomics (Created in BioRender. Verstreken, P. (2025) https://biorender.com/p56u250). (**B**) Scaled gene expression of representative genes for ensheathing glia (EG) and neurons after sorting EG or neurons using the protocol described in (**A**). N=2, 2 replicates. (**C**) Differentially expressed genes (DEGs) in EG in *Pink1^KO-WS/y^* compared to control flies, plotted according to their log_2_foldchange and the –log_10_ of the adjusted p-value. Intercept in red (–log_10_ adjusted p-value = 4.31); light green dots are all detected genes, dark green are the 50 most deregulated genes, and the pink dot is from a gene positive in the genetic screen in (**D**). *Two data points are outside the boundaries of the plot. To determine the transcriptomic profile of each genotype, N=3 independent repeat experiments were used, with 3 replicates each time. (**D**) Electroretinogram (ERG) ON peak value differences of control (red) and of *Pink1^KO-WS/y^* flies (5±1-day-old) with DEGs downregulated or upregulated, specifically in EG relative to *Pink1^KO-WS/y^*. ANOVA with Dunnett’s test, * is p<0.05, ** is p<0.01, **** is p<0.0001. Effect size: η^2^=0.44. Bars: mean ± SD; points are individual animals, N≥3 per genotype. *One data point is outside the boundaries of the plot.

To identify pathways in EG that are deregulated by *Pink1* loss-of-function, we optimized a method to isolate EGs from the rest of the brain, enabling us to perform much higher-sensitivity transcriptomics than what we achieved with our 10× droplet-based single-cell sequencing (Pech et al., 2025). We fluorescently labeled EG nuclei (*GMR-56-Gal4*) with histone-fused GFP (*UAS-His2Av::eGFP*) and used fluorescence-activated cell sorting (FACS) to isolate EG. We sorted 300 cells per technical replicate and performed bulk RNAseq using the highly sensitive SMART-seq2 protocol to robustly generate RNAseq libraries from this low number of input material (Figure 3A). The sequencing results of EG-sorted cells vs. neuron-sorted cells (*nSyb-Gal4* instead of *GMR-56-Gal4*) show strong enrichment of EG cell markers in the former and strong enrichment of neuronal markers in the latter (Figure 3B), thus indicating that we can reliably obtain brain-cell-type-specific transcriptomes with this approach. We then compared the transcriptomic profile of EG in *Pink1^KO-WS/y^* to that of control using differential expression analysis and found 617 deregulated genes in EG (Supplementary file 1, padj<0.05) (Figure 3C). To understand whether the deregulated genes were enriched for a specific pathway or associated with a cellular component compared to non-significantly deregulated genes, we performed Gene Ontology analysis of the deregulated genes in *Pink1^KO-WS/y^* flies. Our analysis resulted in generic terms and very diverse pathways that did not allow us to draw further conclusions.

To test if the genes deregulated in EG are modifiers of the neuronal *Pink1^KO-WS/y^* phenotypes, we downregulated and upregulated (when possible) the 50 most deregulated genes (Figure 3C), for which RNAi lines were available, specifically in the EG of *Pink1^KO-WS/y^* flies and recorded ERGs in 5-day-old animals. Knockdown of one of these genes (*CG17660*) resulted in a statistically significant rescue of the ON transient defects in *Pink1^KO-WS/y^*, and a few exacerbated the defect (Figure 3D). Interestingly, the human orthologs of *CG17660, TMEM87A/B*, have an established role in endosomal sorting (Gaudet et al., 2011; Hirata et al., 2015). Hence, our genetic screening suggests that *Pink1^KO-WS/y^* phenotypes might be suppressed by downregulating genes involved in vesicle trafficking in EG.

### PD causative vesicle trafficking gene downregulation in EG rescues synaptic dopaminergic neurons afferent loss

**Figure 4.**
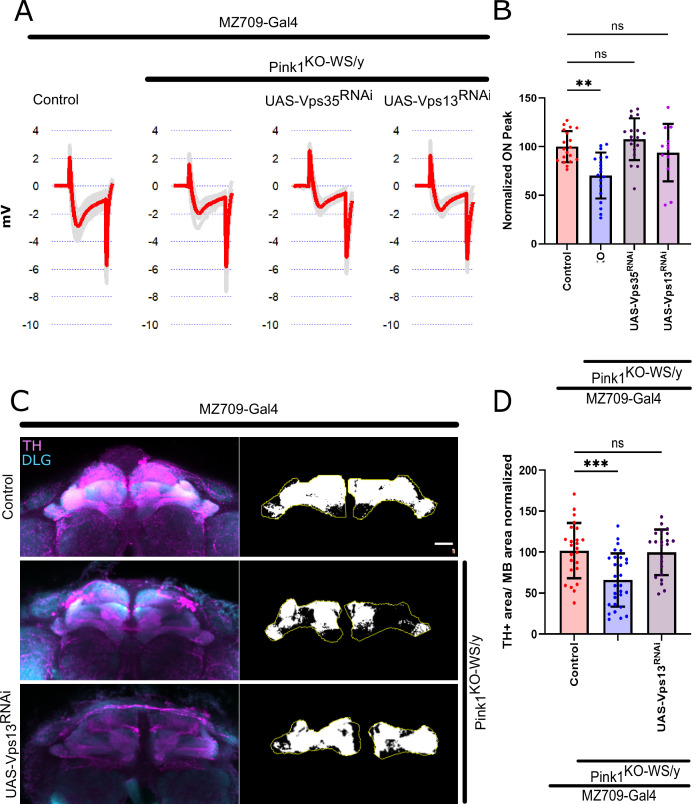
Vps35 and Vps13 downregulation in ensheathing glia (EG) rescues synaptic deficits in *Pink1^KO-WS-y^* flies (with Figure 4—figure supplement 1). (**A**) Representative electroretinogram (ERG) traces of control, *Pink1^KO-WS/y^* flies, and *Pink1^KO-WS/y^* flies with *Vps35* or *Vps13* downregulated in EG. (**B**) Quantification of the normalized ON peak response of flies with the genotypes in (**A**) (5±1-day-old). ANOVA with Dunnett’s multiple comparison test, ns is p>0.05, ** is p<0.01. Effect size: η^2^=0.48. Bars: mean ± SD; points are individual animals, N≥10 per genotype, 3 replicates. (**C**) Maximum intensity projection of confocal images of mushroom bodies (MBs) of aged flies (22±2-day-old) of control, *Pink1^KO-WS/y^,* and *Pink1^KO-WS/y^* with *Vps13* downregulated in EG , labeled with anti-TH (cyan) and anti-DLG (magenta); DLG is used to mark the MB neuropil. The black-and-white image is the middle Z-plane within the region of interest of the MB (regions of interest [ROI], yellow), which is used to represent the thresholded TH area (white). Scale bar: 20 µm. (**D**) Quantification of the dopaminergic synaptic area within MB of aged flies (22±2-day-old). ANOVA with Dunnett’s multiple comparison test, ns is p>0.05, *** is p<0.001. Effect size: η^2^=0.23. Bars: mean ± SD; points are individual animals, n≥22 per genotype, 5 replicates.

Because our ERG-based modifier screen for the *Pink1^KO-WS/y^* phenotype identified a gene with a known role in vesicle trafficking, we next asked whether *Pink1* in EG also genetically interacts with other PD-causative genes that function in vesicle trafficking, specifically vesicle trafficking components affecting membrane interactions between mitochondria and the endoplasmic reticulum (ER), like *Vps35* and *Vps13* (Brickner and Fuller, 1997; Lesage et al., 2016; Vilariño-Güell et al., 2011; Zimprich et al., 2011). We find that the knockdown of each of these genes in EG also rescues the ON transient defect of *Pink1^KO-WS/y^* mutants (Figure 4A and B).

**Figure 4—figure supplement 1.**
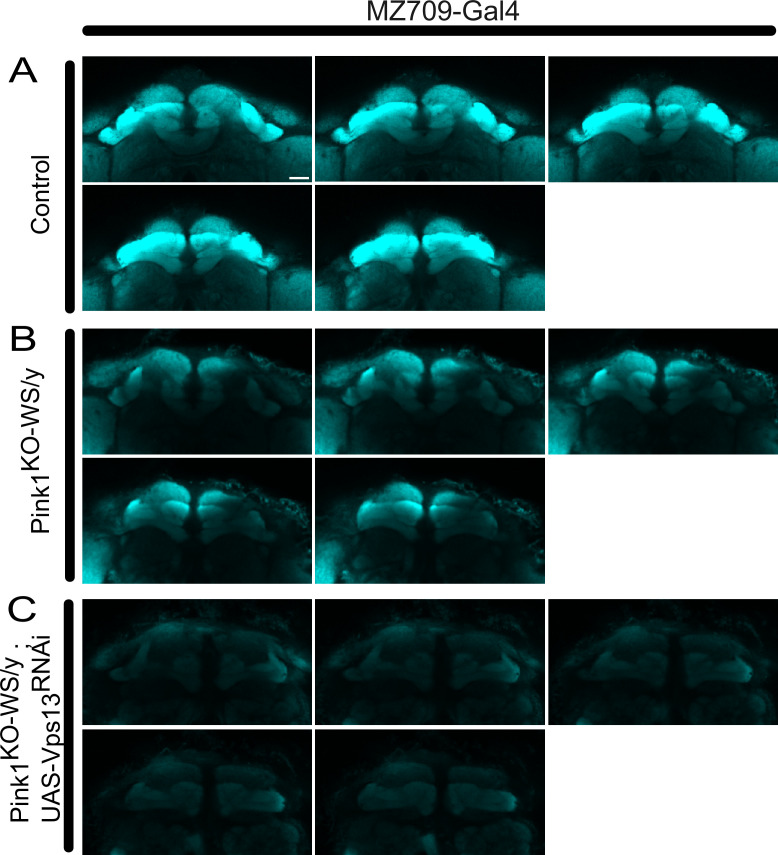
Examples of confocal Z-stacks of anti-DLG labeled fly brains (22±2-day-old) of the indicated genotypes. Such images were used to delineate regions of interest (ROIs) to quantify dopaminergic neuron (DAN) innervation onto the mushroom bodies (MBs). (**A**) Control (w^1118/y^;; MZ709-Gal4/+); (**B**) *Pink1^KO-WS/y^*;; MZ709-Gal4/+; (**C**) *Pink1^KO-WS/y^* flies with *Vps13* downregulation in ensheathing glia (EG) (*Pink1^KO-WS/y^;; Vps13*RNAi/MZ709-Gal4). Scale bar: 20 µm.

To determine if manipulation of this pathway in EG can also rescue the synaptic innervation defect of PAM DAN onto the MBs in old (20- to 25-day-old) *Pink1^KO-WS/y^* mutants, we expressed *Vps13* RNAi in EG using *MZ709-Gal4*. Anti-TH labeling (marking DAN synapses) in the MB area (marked by anti-DLG) is significantly reduced in aged *Pink1^KO-WS/y^*, but is rescued to levels comparable to that observed in controls when *Vps13* is knocked down in EG (Figure 4C, D, Figure 4—figure supplement 1). These results suggest that manipulation of vesicle trafficking components affecting membrane interactions between mitochondria and the ER in EG regulates glia-neuron cross-talk to maintain synaptic integrity of dopaminergic neuron synapses in the fly brain of *Pink1* mutants. Note that the DA synaptic rescue was tested for Vps13 but not for Vps35, and whether EG-specific Vps35 knockdown similarly protects dopaminergic synapses remains to be determined.

## Discussion

In this work, we provide evidence for early, non-cell-autonomous activation of EG in a PD-relevant *Drosophila Pink1* model. This occurs already in young *Pink1*-mutant flies when neuronal defects in dopaminergic neurons are not yet measurable. The EG activation and invasion phenotype elicited by *Pink1*-deficient neurons appears as a protective response as it seems to mimic the response to nerve injury (Doherty et al., 2009; MacDonald et al., 2006). We show that there is a non-autonomous role of EG to support *Pink1*-deficient neurons. When we manipulate the expression of membrane-lipid trafficking genes, the homologs of the established PD genes VPS13C and VPS35 (Lesage et al., 2016; Vilariño-Güell et al., 2011; Zimprich et al., 2011), specifically in EG, we rescue the dysfunction of *Pink1* mutant neuron defects as measured by ERGs and age-dependent PAM DAN synapse loss. Our work shows the importance of neuron-glia cross-talk in the context of *Pink1-*deficiency. We suggest (1) there is early EG activation secondary to *Pink1*-loss-induced neuronal impairments; (2) there are specific lipid- and membrane trafficking problems caused by *Pink1* loss in EG that center on mitochondria/ER contacts; and (3) there is a convergence of Parkinson-relevant genetic factors that act in EG to maintain neuronal function. *CG17660*, the single ERG screen hit that initially pointed toward vesicle trafficking as a relevant pathway, has not been further validated in this study; full characterization and testing of DA synaptic rescue in Pink1 mutants remain an important direction for future work.

The loss of Pink1 function in the context of PD has been amply linked to the regulation of mitochondrial health. Pink1 maintains the integrity of the electron transport chain by phosphorylating NDUFA10 (Morais et al., 2009; Morais et al., 2014; Pogson et al., 2014), and when mitochondria are damaged, it phosphorylates Parkin and ubiquitin to facilitate mitophagy (Kane et al., 2014; Narendra et al., 2010; Narendra and Youle, 2024). The regulation of mitophagy is complex and requires mitochondrial rearrangements controlled by mitochondria-organelle contacts (ER and lysosomes) (Wong et al., 2018; Wong et al., 2019; Yamano et al., 2018). Such contacts mediate inter-organellar lipid exchange, as well as facilitate organellar fusion (Kumar et al., 2018; Valadas et al., 2018). In *Pink1* mutants, these contacts appear to be increased in number, and this causes cellular defects (Grossmann et al., 2023; Valadas et al., 2018).

**Figure 5.**
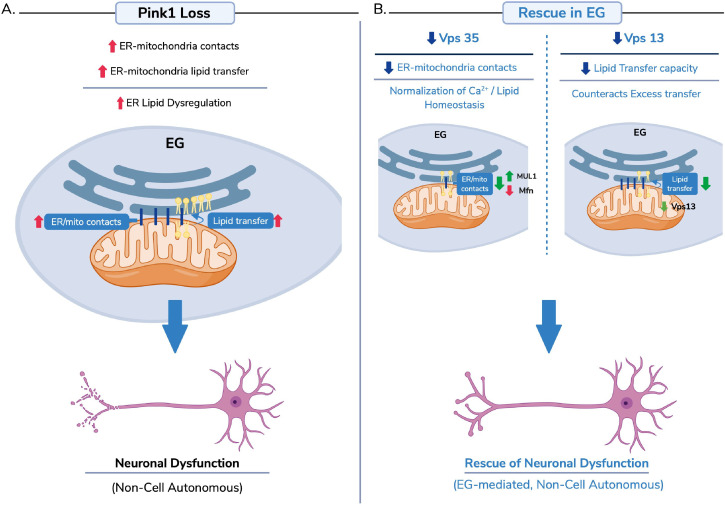
Modulation of endoplasmic reticulum (ER)-mitochondria contact sites and lipid transfer in ensheathing glia (EG) rescues Pink1-dependent neuronal dysfunction. Schematic representation of the suggested model (Created with BioRender.com). (**A**) Loss of *Pink1* leads to an abnormal increase in endoplasmic reticulum (ER)-mitochondria contact sites (represented by blue thick lines), resulting in enhanced ER-to-mitochondria lipid transfer and dysregulation of ER lipid composition (represented by yellow lipids). Increased organelle membrane contacts and lipid flux in EG contribute to neuronal dysfunction in a non-cell-autonomous manner. (**B**) Genetic downregulation of ER-mitochondria contact and lipid transfer regulators in EG rescues *Pink1*-induced neuronal phenotypes through two convergent mechanisms. Reduction of *Vps35* may decrease the number of ER-mitochondria contact sites, possibly via MUL1-mediated Mitofusin (Mfn) turnover, potentially normalizing calcium and lipid homeostasis. In parallel, downregulation of *Vps13*, a lipid transfer facilitator at organelle contact sites, limits ER-to-mitochondria lipid transfer capacity, counteracting the excessive lipid flux induced by *Pink1* loss. Both interventions restore organelle homeostasis in EG and result in rescue of neuronal dysfunction through a non-cell-autonomous mechanism. Rescue of neuronal dysfunction by EG-specific *Vps35^RNAi^* was demonstrated in the visual system (electroretinogram [ERG] on-transient; Figure 4A and B). Whether this extends to dopaminergic synaptic loss, as demonstrated for *Vps13* (Figure 4C and D), has not yet been examined and may be cell-type- and/or phenotype-specific.

We found that in EG, *Vps35* functionally interacts with Pink1-induced neuronal dysfunction: its genetic manipulation in EG rescues cell non-autonomous neuronal dysfunction. Loss of *Vps35* itself only in EG is sufficient to rescue neuronal *Pink1* phenotypes. *Pink1* loss-of-function models show increased numbers of mitochondria-ER contact sites (Valadas et al., 2018), affecting mitochondrial calcium levels (Barazzuol et al., 2020; de Brito and Scorrano, 2008; Ham et al., 2023) and dysregulating lipidic ER composition (Valadas et al., 2018; Figure 5A). Indeed, Pink1 is necessary for ubiquitination and degradation of Mitofusin (Poole et al., 2010), which is fundamental for the mitochondria-ER tethering (de Brito and Scorrano, 2008). Interestingly, *Vps35* deficiency promotes untethering of mitochondria-ER contact sites by increasing the mitochondrial levels of MUL1, which is necessary for the ubiquitination and degradation of Mitofusin (Puri et al., 2019; Tang et al., 2015; Yun et al., 2014). Hence, conditions that affect mitochondria-ER contact site regulation in EG, by modifying an endosomal trafficking regulator such as *Vps35,* are expected to rescue Pink1 neuronal dysfunction, possibly by restoring mitochondrial calcium levels and the lipid composition of the ER (Figure 5B), but further work is needed to sort these mechanistic elements in a cell-type-specific manner. We note that while EG-specific *Vps35* downregulation rescues the ERG on-transient defect in the visual system, whether it similarly protects dopaminergic synapses, as shown for *Vps13* (Figure 4C and D), has not been tested, and this rescue may be cell-type- or phenotype-specific.

Our observation that downregulation of *Vps13* in EG also rescues *Pink1* is in further support of a role for ER-mitochondria contact site regulation. *Vps13* has two mammalian homologs, VPS13A and VPS13C (Velayos-Baeza et al., 2004; Vonk et al., 2017; Vrijsen et al., 2022), which are also mutated in familial Parkinsonism/neurodegenerative disease (Lesage et al., 2016). VPS13A tethers ER to mitochondria and lipid droplets, and VPS13C tethers ER to late endosomes, lysosomes, and lipid droplets (Kumar et al., 2018; Muñoz-Braceras et al., 2019; Vrijsen et al., 2022). The function of these proteins is to facilitate lipid transfer between organelles, a function we previously showed to be increased in *Pink1* mutants (Valadas et al., 2018). Hence, the downregulation of *Vps13* would counteract the effects of excessive organelle membrane contact formation and dysregulated lipid homeostasis in *Pink1* mutants (Figure 5B). We note, however, that while we propose modulation of ER-mitochondria contacts as the mechanism underlying both the *Vps13* and *Vps35* rescue, direct molecular evidence for this in EG is currently lacking and represents an important question for future investigation.

Lipid homeostasis in glial cells is crucial for supporting neuronal function by providing energy, protecting against oxidative stress, and regulating synaptic health. In oligodendrocytes, maintaining myelin sheaths is critical (Ettle et al., 2016; Lappe-Siefke et al., 2003). While fly EG do not generate such structures, they do wrap processes around neuropil regions, also requiring lipid membrane production (Kremer et al., 2017; Pogodalla et al., 2021). Through lipid metabolism, glia also supply neurons with essential metabolites, cholesterol, and signaling molecules that aid in membrane integrity, synaptic remodeling, and neuroprotection (Delgado et al., 2018; Otto et al., 2018; Saab et al., 2016; Suárez-Pozos et al., 2020). Further work is now required to define which functions, supported by organelle contact sites in glial cells, drive cell-non-autonomous protective mechanisms in neurons. Our work in flies indicates these glial changes precede dopaminergic synapse loss, and, importantly, that rescuing glial defects helps to prevent dopaminergic problems later in life.

## Methods

### Resource availability

#### Lead contact

Further information and requests for resources and reagents should be directed to the lead contact, Patrik Verstreken (patrik.verstreken@kuleuven.be).

#### Materials availability

Data, code, *Drosophila* models, and reagents are available upon request.

### Fly stocks

The fruit flies were maintained in an incubator at 25°C under a 12 hr:12 hr light-dark cycle and provided with a standard diet consisting of corn meal and molasses. For experiments, only male flies were used. Flies were raised in parallel on the same batch of food, which was exchanged every 3–4 days and kept in only male populations of similar density. Flies were aged to 5±1-day-old and 22±2-day-old, as indicated for immunohistochemistry and ERGs, respectively.

To create a set of *Drosophila* models for PD, CRISPR/Cas9-based gene editing was utilized, as outlined in Kaempf et al., 2026; Pech et al., 2025. In brief, each knockout line contained an attP-flanked w+ reporter cassette that replaced the first shared exon across different isoforms of the targeted gene. For this study, we used knockout flies for *Pink1* from this collection (Kaempf et al., 2026). The ‘control’ strain denotes a semi-isogenized *w^1118^* strain that underwent backcrossing to Canton-S for 10 successive generations, resulting in a strain termed Canton-S-w^1118^. To generate the fly line *yw; UAS-His2Av::eGFP.VK27/TM3Sb*, we created the pUASTattB_His2Av::eGFP plasmid, linearizing the pUASTattB (Bischof et al., 2007) with EcoRI-BamHI. A gBlock containing the His2Av, a GS linker, and eGFP sequence was cloned into this linearized plasmid with Gibson Assembly. The plasmid was inserted into the VK27 landing site by BestGene. The sequence His2Av::GS::eGFP gBlock is:

TGAATAGGGAATTGGGcAAacATGGCTGGCGGTAAAGCAGGCAAGGATTCGGGCAAGGCCAAGGCGAAGGCGGTATCGCGTTCCGCGCGCGCGGGTCTTCAGTTCCCCGTGGGTCGCATCCATCGTCATCTCAAGAGCCGCACTACGTCACATGGACGCGTCGGAGCCACTGCAGCCGTGTACTCCGCTGCCATATTGGAATACCTGACCGCCGAGGTCCTGGAGTTGGCAGGCAACGCATCGAAGGACTTGAAAGTGAAACGTATCACTCCTCGCCACTTACAGCTCGCCATTCGCGGAGACGAGGAGCTGGACAGCCTGATCAAGGCAACCATCGCTGGTGGCGGTGTCATTCCGCACATACACAAGTCGCTGATCGGCAAAAAGGAGGAAACGGTGCAGGAcCCGCAGCGGAAGGGCAACGTCATTCTGTCGCAGGCCTACGGTTCAGGCGGAGGTGGCAGCGGCGGTGGCGGATCCATGGTGAGCAAGGGCGAGGAGCTGTTCACCGGGGTGGTGCCCATCCTGGTCGAGCTGGACGGCGACGTAAACGGCCACAAGTTCAGCGTGTCCGGCGAGGGCGAGGGCGATGCCACCTACGGCAAGCTGACCCTGAAGTTCATCTGCACCACCGGCAAGCTGCCCGTGCCCTGGCCCACCCTCGTGACCACCCTGACCTACGGCGTGCAGTGCTTCAGCCGCTACCCCGACCACATGAAGCAGCACGACTTCTTCAAGTCCGCCATGCCCGAAGGCTACGTCCAGGAGCGCACCATCTTCTTCAAGGACGACGGCAACTACAAGACCCGCGCCGAGGTGAAGTTCGAGGGCGACACCCTGGTGAACCGCATCGAGCTGAAGGGCATCGACTTCAAGGAGGACGGCAACATCCTGGGGCACAAGCTGGAGTACAACTACAACAGCCACAACGTCTATATCATGGCCGACAAGCAGAAGAACGGCATCAAGGTGAACTTCAAGATCCGCCACAACATCGAGGACGGCAGCGTGCAGCTCGCCGACCACTACCAGCAGAACACCCCCATCGGCGACGGCCCCGTGCTGCTGCCCGACAACCACTACCTGAGCACCCAGTCCGCCCTGAGCAAAGACCCCAACGAGAAGCGCGATCACATGGTCCTGCTGGAGTTCGTGACCGCCGCCGGGATCACTCTCGGCATGGACGAGCTGTACAAATAAGGGTACCTCTAGAGGATCTT

Flies were backcrossed for five generations into our Canton-S-w^1118^ reference background. The genotypes used in this study are listed in the Key resources table and Supplementary file 2.

### ORN axotomy

ORN bilateral axotomy was performed on 4±1-day-old flies by surgical ablation of the third antennal segment. Briefly, flies were anesthetized with CO_2_, and then both antennae were removed with Dumont #5 forceps and returned to the vial (Purice, 2020; Purice et al., 2017). Flies were allowed to recover for 24 hr on food at 25°C before dissection to perform imaging.

### Immunohistochemistry and confocal imaging – invasion phenotype

Immunohistochemistry was performed on adult fly brains of 5±1-day-old, with at least two independent experiments. The brains were dissected in ice-cold PBS and fixed for 20 min in freshly prepared 3.7% paraformaldehyde (in 1× PBS, 0.3% Triton X-100 [PBX] [Sigma]) at room temperature (RT), followed by three 15 min washes in PBX at RT on a shaker. Then, the brains were incubated for 1 hr in blocking solution (PBX, 10% normal goat serum [NGS]) at RT. Following blocking, the brains were incubated with primary antibodies (rabbit anti-GFP [Thermo Fisher Scientific], 1:1000, mouse anti-Brp [DSHB, nc82], 1:100) in blocking solution at 4°C overnight, followed by three 15 min washes in PBX at RT. Secondary antibodies (goat anti-rabbit Alexa488, goat anti-mouse Alexa555, both at 1:1000 [Thermo Fisher Scientific]) in PBX with 10% NGS were subsequently applied overnight at 4°C. Afterward, the brains were washed three times for 15 min in PBT at RT on a shaker and mounted with the anterior facing up in RapiClear 1.47 (SUNJin Lab). Imaging was performed using a Nikon A1R confocal microscope with a 40× (NA 1.15) water immersion lens; Z-stacks (with 0,5 μm step intervals) of the entire antennal lobes were acquired, and the same imaging settings were applied across all genotypes and sessions. The acquisition was carried out using a Galvano scanner, with a zoom factor of 1, scan speed of 0.5, and line averaging set to 2. All images were captured with a pinhole of 0.9 Airy units and a resolution of 1024×1024 pixels. Image analysis was performed using Fiji (Schindelin et al., 2012). Each antennal lobe is composed of multiple glomeruli, and EG separate them and invade them when activated (Doherty et al., 2009; Pogodalla et al., 2021). To quantify EG invasion into the antennal lobe, we calculated the invasion in every single glomerulus. To do so and be consistent with the section of the antennal lobe analyzed, anti-Brp was used to identify the three Z-planes, where DM6 and DM1 glomeruli were present (Wu et al., 2017a). Then, to automatically detect and segment each glomerulus of the antennal lobe section, regions of interest (ROIs) were defined in the SUM projection of the three Z-planes by using the Fiji plugin Stardist (Weigert et al., 2019). The GFP intensity was detected in each ROI, and it was normalized to the area of each ROI (GFP intensity/glomeruli area) to calculate the EG invasion in each glomerulus. Then, the normalized GFP intensity of each glomerulus of an antennal lobe was summed and normalized for the number of glomeruli detected in the antennal lobe. For each experiment, the EG antennal lobe invasion of each fly was normalized to the mean of the control. Representative images show the maximum intensity projections of the three Z-planes.

### Immunohistochemistry and confocal imaging – TH staining

Immunohistochemistry was performed on adult fly brains of 22±2-day-old, with at least two independent experiments. The brains were dissected in ice-cold PBS and fixed for 20 min in freshly prepared 3.7% paraformaldehyde (in 1× PBS, 0.2% Triton X-100 [PBX]) at RT, followed by three 15 min washes in PBX at RT on a shaker. Then, the brains were incubated for 1 hr in blocking solution (PBX, 10% NGS) at RT. Following blocking, the brains were incubated with primary antibodies (rabbit anti-TH [Sigma], 1:200, mouse anti-DLG [DSHB], 1:100) in blocking solution at 4°C for 1.5–2 days, followed by three 15 min washes in PBX at RT. Secondary antibodies (goat anti-rabbit Alexa488, goat anti-mouse Alexa555, both at 1:500 [Thermo Fisher Scientific]) in PBX with 10% NGS were applied overnight at 4°C. Afterward, the brains were washed three times for 15 min in PBT at RT on a shaker and mounted with the anterior facing up in RapiClear 1.47 (SUNJin Lab). Imaging was performed using a Nikon A1R confocal microscope with a 20× (NA 0.95) water immersion lens; Z-stacks (with 3 μm step intervals) of the entire brain were acquired, and the same imaging settings were applied across all genotypes and sessions. The acquisition was carried out using a Galvano scanner, with a zoom factor of 1, scan speed of 0.5, and line averaging set to 2. All images were captured with a pinhole of 2.3 Airy units and a resolution of 1024×1024 pixels. Image analysis was performed using Fiji (Schindelin et al., 2012). To quantify dopaminergic neuron innervation of the MB, anti-DLG was used to identify the five Z-planes containing the synaptic region of the MB lobes. The ROI for the MB was defined in the sum projection of the five Z-planes. To exclude background signal comparable to control, the area of anti-TH fluorescence was thresholded (using the default threshold for all Z-planes) within the selected Z-stacks. Quantification of the thresholded area within the ROI was performed in each Z-plane, then summed and normalized to the MB ROI area for each brain individually (TH+area/MB area). For each experiment, the individual TH+area/MB area values were normalized to the mean of the control. Representative images show the maximum projection of five Z-planes and the thresholded middle Z-plane.

### Electroretinograms

ERGs were recorded from flies 5±1-day-old as previously described (Heisenberg, 1971; Slabbaert et al., 2016). Flies were immobilized on glass microscope slides using double-sided tape. For recordings, glass electrodes (borosilicate, 1.5 mm outer diameter) filled with 3 M NaCl were placed in the thorax as a reference and on the fly eye for recordings. Each fly was exposed to 5 cycles of 3 s of darkness, followed by 1 s of light stimuli with LED illumination. Response to the stimuli was recorded using Axoscope 10.7 and analyzed using Clampfit 10.7 software (Molecular Devices). ERG traces were analyzed with Igor Pro 6.37 (Wave Metrics) using a custom-made macro.

### Single-cell dissociation for cell-type-specific transcriptomics

To test that EG could be isolated from the rest of the brain, we labeled EG and neurons separately in two cohorts of flies with *GMR-56-Gal4>UAS-His2Av::eGFP* and *nSyb-Gal4>UAS-His2Av::eGFP*. To identify DEGs in EG, two cohorts were used: the control *w^1118^* expressing *GMR-56-Gal4>UAS-His2Av::eGFP* and *Pink1^KO-WS/y^* expressing *GMR-56-Gal4>UAS-His2Av::eGFP*. For each genotype, 10 brains of flies 5±1-day-old were collected, with most of the laminae removed, from each experimental repeat. The dissections were performed in ice-cold PBS containing 5 μM Actinomycin D (Sigma) to inhibit changes in gene expression during tissue processing (Wu et al., 2017b). To prevent any bias caused by different experimental batches, all the genotypes were processed in parallel. To minimize variability across batches, the same reagents and procedures were used throughout the experiment. Dissections were completed within 1 hr. The dissection order and the assignment of genotypes to dissectors were alternated to reduce any experimenter-related variation.

For the dissociation of brain tissue, a mix of Dispase I (0.6 mg/ml) (Sigma), Collagenase I (30 mg/ml) (Thermo Fisher), Trypsin (0.5×) (Thermo Fisher), and 5 μM Actinomycin D was used. The brains were incubated for 20 min at 25°C with 1000 rpm shaking, performing four triturations at 5 min intervals. After dissociation, the cell suspensions were washed with PBS containing 5 μM Actinomycin D and then filtered through a 10 μm strainer (Pluriselect), using 300 μl of PBS EDTA (Sigma) 1 μM. DAPI (Sigma) (1:300,000) was added after filtration.

### FACS for cell-type-specific transcriptomics

Immediately after the dissociation, cells were sorted using a FACSAria Fusion (BD Biosciences) flow cytometer equipped with four lasers (405 nm, 488 nm, 561 nm, and 640 nm) and a 100 µm nozzle at 20 psi. Live (DAPI-negative), GFP-positive cells were sorted into 96-well PCR plates at 300 cells per well (three wells per genotype) using a four-way purity mask with the FACSDiva software v9.0.1 (BD Biosciences).

### Bulk transcriptomics of EG cells

Library preparations and sequencing for the bulk RNAseq of the EG cells were performed using a modified Smart-seq2 protocol (Picelli et al., 2013). Briefly, 3 μl of cell lysis buffer (0.1% Triton X-100, Sigma-Aldrich; 1 U/µl of RNase Inhibitor, RNase OUT [Thermo Fisher]; 2.5 μM Oligo dT[25], (IDT); and 2.5 mM dNTP [Promega]) was aliquoted in triplicate to 96-well plates (4titude, Cat. No. 4TI-0960/C). Roughly 300 cells were sorted into the wells with lysis buffer and the plate was spun down at 2000×*g* for 1 min prior to storage at –80°C. For the first-strand synthesis, the Smart-Seq2 plates were thawed to RT for a minute and subsequently spun down at 2000×*g* for a minute. The RNA denaturation was carried out at 72°C for 10 min and then flash-cooled on ice for 5 min. First-strand synthesis mix (1× SuperScript II first strand synthesis buffer [Thermo Fisher]; 5 mM DTT [Thermo Fisher]; 100 U SuperScript II reverse transcriptase enzyme [Thermo Fisher]; 10 U RNAse OUT [Thermo Fisher]; 1 M Betaine [Sigma-Aldrich]; 6 mM MgCl_2_ [Thermo Fisher]; and 1 µM TSO [IDT Technologies]) was added to the cell lysis mix to a total volume of 10 μl. The first-strand synthesis reaction was carried out with the following program: 42°C for 90 min; 10 cycles of {50°C for 2 min; 42°C for 2 min}; 72°C for 15 min; 4°C indefinite hold. PCR amplification of the first-strand product was performed by adding 15 μl of the PCR mix to the first-strand product (1× KAPA HiFi HotStart Ready Mix [Roche]; and 0.1 μM of IS PCR primer [IDT Technologies]). 22 cycles of the following PCR program were used to amplify the first-strand product: 98°C for 3 min; 22 cycles of {98°C for 20 s; 67°C for 15 s; 72°C for 6 min}; 72°C for 5 min; 4°C indefinite hold. 20 μl of Ampure XP was added to each well and mixed. Standard Ampure XP purification was carried out as per the manufacturer’s recommendation, and the cDNA library was eluted in 17.5 μl of Elution buffer. 17 μl of the eluted library was transferred to a fresh 96-well plate (4titude, Cat. No. 4TI-0960/C). 5 μl of Tn5 tagmentation mix (1× Tagment DNA buffer, Illumina; ATM mix [Illumina], and cDNA library 1 ng) was prepared for cDNA fragmentation. Tn5 tagmentation was performed with the following program: 55°C for 10 min; 4°C indefinite hold. Tagmentation reaction was stopped by quenching the reaction with 1.25 μl of NT buffer for 5 min (Illumina). 1.25 μl of the i5 and i7 Illumina indexes were added to the plates. Finally, 3.75 μl of NPM master mix was added to the plate and mixed well and put for the index PCR amplification: 72°C for 3 min; 95°C for 30 s; 12 cycles of {95°C for 10 s; 55°C for 30 s; 72°C for 30 s}; 72°C for 5 min, and 4°C indefinite hold.

The indexed PCR products were pooled in a single tube, and 0.8× Ampure XP purification (Beckman Coulter) was carried out as per the manufacturer’s recommendation, and finally, the sequencing library was eluted in 35.5 μl of Elution buffer (QIAGEN).

Smart-seq2 libraries were sequenced on the NextSeq 500 (Illumina) sequencing platform. Sequencing was done as per the protocol recommendations: paired-end read of 76 bps (read 1), 76 bps (read 2), 8 bps (index 1), and 8 bps (index 2), targeting a sequencing depth of ~30 million reads per sample.

### Analysis of RNAseq data – neuron vs. EG

Raw reads from neuron- vs. glia-specific FACS-sorting experiments were processed using the nf-core/rnaseq pipeline (Patel et al., 2020) with default parameters and the BDGP6 reference genome. The STAR-aligned and salmon-quantified expression matrix was then subset to genes known to be enriched in neurons or glia and displayed as a per-gene scaled heatmap.

### Analysis of RNAseq data – EG-specific DEGs

After FACS sorting of *Pink^KO-WS^* vs. control EG, FASTQ files resulting from library preparation and sequencing were cleaned with fastp (Chen et al., 2018) with default settings. Aligning and counting were carried out with STAR (Dobin et al., 2013) and the 4th 2020 FlyBase *Drosophila melanogaster* release (r6.35) with the –quantMode flag set as GeneCounts. For DGE testing, we used DESeq2 (Love et al., 2014), where we fit a negative binomial model and carried out the Wald test. We removed the experimental batch as a covariate according to the design formula~date+genotype. All genes below a Benjamini-Hochberg-corrected p-value of 0.05 were considered deregulated.

### scRNAseq data loading, filtering, clustering, and differential expression analysis

Raw count data from Pech et al., 2025, alongside cell annotations provided by the authors, was loaded into Scanpy (v1.9.1) (Wolf et al., 2018) and subset to only include cells in the young control (≤6-day-old) or *Pink1^P399L^* samples. After subsetting the data, filtering was performed to remove any cell with less than 250 genes expressed or a percentage of counts coming from mitochondrial genes greater than 15%, the data was then normalized to a total of 10,000 counts and log-transformed. Highly variable genes were detected, total counts and percent of mitochondrial reads were regressed out, and finally the data was scaled to unit variance with a zero mean, clipped to a max of 10.

After data pre-processing, a PCA was performed, Harmony (Korsunsky et al., 2019) was used to correct batches using ‘sample_id’ as a batch key, and 122 components were used for dimensionality reduction and clustering analyses. Leiden clustering was performed with a resolution of 8.0 and using the annotations from Pech et al., 2025, all cells within each cluster were labeled by taking the most common, original annotation per cluster.

Per cluster, a DGE analysis was performed. First, pseudobulk counts were generated by summing the counts per cell type from all cells per sample. These counts were then used in DESeq2 (v1.44.0) (Love et al., 2014) comparing control vs. *Pink1^P399L^* samples. The total number of genes significantly up- or downregulated per cluster (padj≤0.05 and an abs (log_2_foldchange) ≥ 1.5) was counted and plotted.

### Statistical analysis

GraphPad Prism was used for visualization and to determine statistical significance. Datasets were tested for normal distribution using the D’Agostino-Pearson Omnibus and the Shapiro-Wilk normality test. For a normally distributed dataset, ordinary one-way ANOVA was used, followed to correct for multiple comparisons by a post hoc Tukey’s test when comparing all the datasets with each other or Dunnett’s test when comparing all the datasets to a general control. For non-normally distributed datasets, the Kruskal-Wallis test is used, followed by a post hoc Dunn’s test to correct for multiple comparisons. Significance levels are defined as * is p<0.05, ** is p<0.01, *** is p<0.001, **** is p<0.0001, and ns, not significant, p>0.05. Effect size is calculated with eta-squared when one-way ANOVA is used, indicated in the figure legend as η^2^. ‘N’ in the legends is used to indicate how many animals were analyzed. Data are plotted as mean ± SD. Specifics on the statistical test used for each analysis are reported in the figure legends.

### Inclusion and diversity

We support inclusive, diverse, and equitable conduct of research.

## Data Availability

RNA sequence data were deposited in GEO (accession number GSE322929). All data generated or analyzed during this study are included in the manuscript and supporting files; source data file has been provided for all figures in Source data 1. The following dataset was generated: GhezziL KuenenS PechU SchoovaertsN KilicA PoovathingalS DavieK LamoteJ PraschbergerR VerstrekenP 2026Parkinson's Disease-Associated Pink1 Loss Disrupts Ensheathing Glia And Causes Dopaminergic Neuron Synapse LossNCBI Gene Expression OmnibusGSE32292910.7554/eLife.105386PMC1347262942592915 The following previously published dataset was used: JanssensJ PechU AertsS VerstrekenP 2025Rescuing early Parkinson-induced hyposmia prevents dopaminergic system failure [fruit fly]NCBI Gene Expression OmnibusGSE228843

## Additional files

Supplementary file 1. Differentially expressed genes (DEGs) in ensheathing glia (EG) identified by cell-type-specific transcriptomics.

Supplementary file 2. Complete list of *Drosophila melanogaster* genotypes used in this study.

MDAR checklist

Source data 1. Numerical source data for Figures 1—4.

## Appendix 1

**Appendix 1—key resources table:**

Reagent type (species) or resource	Designation	Source or reference	Identifiers	Additional information
Antibody	Rabbit polyclonal anti-GFP	Thermo Fisher Scientific	Cat#A-11122; RRID:AB_221569	(1:1000)
Antibody	Mouse monoclonal anti-Brp	DSHB	Cat#nc82; RRID:AB_2314866	(1:100)
Antibody	Mouse monoclonal anti-DLG	DSHB	Cat#4F3; RRID:AB_528203	(1:100)
Antibody	Rabbit polyclonal anti-TH	Sigma-Aldrich	Cat#AB 152	(1:200)
Antibody	Alexa Fluor 488 goat anti-rabbit	Invitrogen	Cat#A11034	(1:1000) in invasion phenotype, (1:500) in TH staining
Antibody	Alexa Fluor 555 goat anti-mouse IgG2a	Invitrogen	Cat#A21137	(1:1000) in invasion phenotype, (1:500) in TH staining
Commercial assay, kit	Agencourt AMPure XP	Beckman Coulter	Cat#A63880	
Commercial assay, kit	KAPA HiFi HotStart ReadyMix	Roche	Cat#07958927001	
Commercial assay, kit	Nextera XT DNA Library Preparation Kit	Illumina	Cat#FC-131-1096	
Peptide, recombinant protein	Dispase I	Sigma-Aldrich	Cat#D4818	
Peptide, recombinant protein	Collagenase I	Thermo Fisher Scientific	Cat#17100017	
Peptide, recombinant protein	SuperScript II Reverse Transcriptase	Thermo Fisher Scientific	Cat#18064022	
Chemical compound, drug	Trypsin-EDTA (0.5%)	Thermo Fisher Scientific	Cat#15400054	
Chemical compound, drug	Triton X-100 Solution	Sigma-Aldrich	Cat#93443-100Ml	
Chemical compound, drug	Paraformaldehyde	Sigma-Aldrich	Cat#252549	
Chemical compound, drug	Actinomycin D	Sigma-Aldrich	Cat#A1410	
Chemical compound, drug	EDTA	Sigma-Aldrich	Cat#E6511	
Chemical compound, drug	DAPI	Sigma-Aldrich	Cat#D9542	
Chemical compound, drug	MgCl_2_ (1 M)	Thermo Fisher Scientific	Cat#AM9530G	
Chemical compound, drug	RNaseOUT	Thermo Fisher Scientific	Cat#10777019	
Chemical compound, drug	Betaine	Sigma-Aldrich	Cat#B0300	
Chemical compound, drug	DTT (100 mM Solution)	Thermo Fisher Scientific	Cat#707265ML	
Chemical compound, drug	Buffer EB	QIAGEN	Cat#19086	
Chemical compound, drug	RapiClear 1.47	Sunjin Lab	Cat#RC147001	
Sequence-based reagent	dNTP Mix	Promega	Cat#U1511	
Sequence-based reagent	Primers, gRNAs, oligos, gBlocks	Integrated DNA Technologies (IDT)		
Strain background (*D. melanogaster*)	*w[1118] (w^1118^*)	Kaempf et al., 2026	NA	
Strain background (*D. melanogaster*)	*w[1118] M{w+} (w^1118^ w+*)	Kaempf et al., 2026	NA	
Genetic reagent (*D. melanogaster*)	*w[1118] TI{w[+]=white-STAR}Pink1[KO-WS]/FM7a (Pink1^KO-WS/y^*)	Kaempf et al., 2026	NA	
Genetic reagent (*D. melanogaster*)	*Yw;; UAS-His2Av::eGFP.VK27/TM3Sb (UAS- His2Av::eGFP*)	This study	NA	
Genetic reagent (*D. melanogaster*)	*;;MZ0709-Gal4*	Ito et al., 1995	NA	
Genetic reagent (*D. melanogaster*)	*y[1] w[*]; P{w[+mC]=UAS-mCD8::GFP.L}LL5, P{UAS-mCD8::GFP.L}2 (UAS-mCD8-GFP*)	Lee and Luo, 1999	BDSC_5137	
Genetic reagent (*D. melanogaster*)	*w[*]; P{y[+t7.7] w[+mC]=GMR-56-GAL4}attP24/CyO (GMR-56-Gal4*)	Jenett et al., 2012	BDSC_77469	
Genetic reagent (*D. melanogaster*)	*y[1] v[1]; P{y[+t7.7] v[+t1.8]=TRiP.JF01672}attP2 (Pink1^RNAi^*)	Perkins et al., 2015	BDSC_31170	
Genetic reagent (*D. melanogaster*)	*w[*]; P{w[+mC]=UAS-Pink1.C}A (UAS-Pink1*)	Bloomington Drosophila Stock Center	BDSC_51648	
Genetic reagent (*D. melanogaster*)	*w[1118]; P{y[+t7.7] w[+mC]=GMR57C10-GAL4}attP2 (nSyb-Gal4*)	Jenett et al., 2012	BDSC_39171	
Genetic reagent (*D. melanogaster*)	*y[1] sc[*] v[1] sev[21]; P{y[+t7.7] v[+t1.8]=TRiP.HMS01715}attP40 (Vps13^RNAi^*)	Perkins et al., 2015	BDSC_38270	
Genetic reagent (*D. melanogaster*)	*y[1] sc[*] v[1] sev[21]; P{y[+t7.7] v[+t1.8]=TRiP.HMS01858}attP40 (Vps35^RNAi^*)	Perkins et al., 2015	BDSC 38944	
Genetic reagent (*D. melanogaster*)	*y[1] sc[*] v[1] sev[21]; P{y[+t7.7] v[+t1.8]=TRiP.HMS05488}attP40 (CG17660^RNAi^*)	Perkins et al., 2015	BDSC_67022	
Genetic reagent (*D. melanogaster*)	*y[1] v[1]; P{y[+t7.7] v[+t1.8]=TRiP.JF03249}attP2 (Proc^RNAi^*)	Perkins et al., 2015	BDSC_29570	
Genetic reagent (*D. melanogaster*)	*y[1] sc[*] v[1] sev[21]; P{y[+t7.7] v[+t1.8]=TRiP.HMC05229}attP40 (fiz^RNAi^*)	Perkins et al., 2015	BDSC_62222	
Genetic reagent (*D. melanogaster*)	*y[1] v[1]; P{y[+t7.7] v[+t1.8]=TRiP.JF01165}attP2 (CG15011^RNAi^*)	Perkins et al., 2015	BDSC_31589	
Genetic reagent (*D. melanogaster*)	*y[1] sc[*] v[1] sev[21]; P{y[+t7.7] v[+t1.8]=TRiP.HMC04857}attP40 (CG17612^RNAi^*)	Perkins et al., 2015	BDSC_57540	
Genetic reagent (*D. melanogaster*)	*y[1] sc[*] v[1] sev[21]; P{y[+t7.7] v[+t1.8]=TRiP.HMC05717}attP40 (Prpk^RNAi^*)	Perkins et al., 2015	BDSC_64844	
Genetic reagent (*D. melanogaster*)	*y[1] v[1]; P{y[+t7.7] v[+t1.8]=TRiP.HMJ22434}attP40 (Lnpk^RNAi^*)	Perkins et al., 2015	BDSC_64036	
Genetic reagent (*D. melanogaster*)	*y[1] sc[*] v[1] sev[21]; P{y[+t7.7] v[+t1.8]=TRiP.HMS01827}attP2 (PIG-B^RNAi^*)	Perkins et al., 2015	BDSC_38359	
Genetic reagent (*D. melanogaster*)	*y[1] sc[*] v[1] sev[21]; P{y[+t7.7] v[+t1.8]=TRiP.HMS01840}attP2/TM3, Sb[1] (spag^RNAi^*)	Perkins et al., 2015	BDSC_38371	
Genetic reagent (*D. melanogaster*)	*y[1] sc[*] v[1] sev[21]; P{y[+t7.7] v[+t1.8]=TRiP.HMS00070}attP2 (Rel^RNAi^*)	Perkins et al., 2015	BDSC_33661	
Genetic reagent (*D. melanogaster*)	*y[1] sc[*] v[1] sev[21]; P{y[+t7.7] v[+t1.8]=TRiP.HMC04640}attP40 (Atac1^RNAi^*)	Perkins et al., 2015	BDSC_57250	
Genetic reagent (*D. melanogaster*)	*y[1] sc[*] v[1] sev[21]; P{y[+t7.7] v[+t1.8]=TRiP.GLV21056}attP2 (l(3)07882^RNAi^*)	Perkins et al., 2015	BDSC_35691	
Genetic reagent (*D. melanogaster*)	*y[1] sc[*] v[1] sev[21]; P{y[+t7.7] v[+t1.8]=TRiP.HMS00529}attP2 (Pld^RNAi^*)	Perkins et al., 2015	BDSC_32839	
Genetic reagent (*D. melanogaster*)	*y[1] sc[*] v[1] sev[21]; P{y[+t7.7] v[+t1.8]=TRiP.HMS02243}attP2 (Rpp25^RNAi^*)	Perkins et al., 2015	BDSC_41679	
Genetic reagent (*D. melanogaster*)	*y[1] v[1]; P{y[+t7.7] v[+t1.8]=TRiP.HMC03184}attP40 (GMF^RNAi^*)	Perkins et al., 2015	BDSC_51452	
Genetic reagent (*D. melanogaster*)	*y[1] sc[*] v[1] sev[21]; P{y[+t7.7] v[+t1.8]=TRiP.HMS01358}attP2/TM3, Sb[1] (Atg7^RNAi^*)	Perkins et al., 2015	BDSC_34369	
Genetic reagent (*D. melanogaster*)	*y[1] v[1]; P{y[+t7.7] v[+t1.8]=TRiP.HMJ03126}attP40 (Ada2a^RNAi^*)	Perkins et al., 2015	BDSC_50905	
Genetic reagent (*D. melanogaster*)	*y[1] sc[*] v[1] sev[21]; P{y[+t7.7] v[+t1.8]=TRiP.HMC06224}attP2 (CG12773^RNAi^*)	Perkins et al., 2015	BDSC_65949	
Genetic reagent (*D. melanogaster*)	*y[1] sc[*] v[1] sev[21]; P{y[+t7.7] v[+t1.8]=TRiP.HMS00328}attP2 (CG7627^RNAi^*)	Perkins et al., 2015	BDSC_32337	
Genetic reagent (*D. melanogaster*)	*y[1] v[1]; P{y[+t7.7] v[+t1.8]=TRiP.HMS00870}attP2 (Dark^RNAi^*)	Perkins et al., 2015	BDSC_33924	
Genetic reagent (*D. melanogaster*)	*y[1] v[1]; P{y[+t7.7] v[+t1.8]=TRiP.JF03275}attP2 (CG3703^RNAi^*)	Perkins et al., 2015	BDSC_29596	
Genetic reagent (*D. melanogaster*)	*y[1] sc[*] v[1] sev[21]; P{y[+t7.7] v[+t1.8]=TRiP.HMS01175}attP2/TM3, Sb[1] (NiPp1^RNAi^*)	Perkins et al., 2015	BDSC_34696	
Genetic reagent (*D. melanogaster*)	*y[1] v[1]; P{y[+t7.7] v[+t1.8]=TRiP.HMJ22813}attP40 (CG31370^RNAi^*)	Perkins et al., 2015	BDSC_60456	
Genetic reagent (*D. melanogaster*)	*y[1] v[1]; P{y[+t7.7] v[+t1.8]=TRiP.HMJ23526}attP40 (Irbp^RNAi^*)	Perkins et al., 2015	BDSC_61942	
Genetic reagent (*D. melanogaster*)	*y[1] v[1]; P{y[+t7.7] v[+t1.8]=TRiP.JF02313}attP2 (CG32532^RNAi^*)	Perkins et al., 2015	BDSC_26750	
Genetic reagent (*D. melanogaster*)	*y[1] v[1]; P{y[+t7.7] v[+t1.8]=TRiP.HMJ30013}attP40/CyO (Rhau^RNAi^*)	Perkins et al., 2015	BDSC 62936	
Genetic reagent (*D. melanogaster*)	*y[1] v[1]; P{y[+t7.7] v[+t1.8]=TRiP.HM05047}attP2 (hfw^RNAi^*)	Perkins et al., 2015	BDSC_28561	
Genetic reagent (*D. melanogaster*)	*y[1] v[1]; P{y[+t7.7] v[+t1.8]=TRiP.JF02192}attP2 (achi^RNAi^*)	Perkins et al., 2015	BDSC_31903	
Genetic reagent (*D. melanogaster*)	*y[1] v[1]; P{y[+t7.7] v[+t1.8]=TRiP.HMJ23972}attP40/CyO (TTLL6A^RNAi^*)	Perkins et al., 2015	BDSC_62488	
Genetic reagent (*D. melanogaster*)	*y[1] sc[*] v[1] sev[21]; P{y[+t7.7] v[+t1.8]=TRiP.HMC03076}attP2 (Nos^RNAi^*)	Perkins et al., 2015	BDSC_50675	
Genetic reagent (*D. melanogaster*)	*w[1118]; P{w[+mC]=UAS-Nos.L}2 (UAS-Nos*)	Bloomington Drosophila Stock Center	BDSC_56823	
Recombinant DNA reagent	pUASTattB	Bischof et al., 2007	https://www.flyc31.org	
Software, algorithm	Fiji	Schindelin et al., 2012	RRID:SCR_002285	https://imagej.net/Fiji
Software, algorithm	GraphPad Prism	GraphPad Software	RRID:SCR_002798	https://www.graphpad.com/scientific-software/prism/
Software, algorithm	Python (v3.7.3)	Python	RRID:SCR_008394	http://www.python.org/
Software, algorithm	scanpy	Wolf et al., 2018	RRID:SCR_018139	https://github.com/theislab/scanpy
Software, algorithm	DESeq2	Love et al., 2014	RRID:SCR_015687	https://bioconductor.org/packages/release/bioc/html/DESeq2.html
Software, algorithm	Clampfit	Molecular Devices		https://www.moleculardevices.com
Software, algorithm	Axoscope	Molecular Devices		https://www.moleculardevices.com
Software, algorithm	Igor Pro	WaveMetrics	RRID:CR_000325	https://www.wavemetrics.com/products/ igorpro/igorpro.htm
Software, algorithm	FACSDiva software v9.0.1	BD Biosciences	RRID:SCR_001456	https://www.bdbiosciences.com/en-be/products/software/instrument-software/bd-facsdiva-software
Software, algorithm	nf-core/rnaseq	Patel et al., 2020		https://nf-co.re/rnaseq/3.14.0/
Software, algorithm	fastp	Chen et al., 2018	RRID:SCR_016962	https://github.com/OpenGene/fastp
Software, algorithm	RStudio	RStudio, PBC/Posit		
Software, algorithm	BioRender	BioRender		https://BioRender.com
Software, algorithm	STAR	Dobin et al., 2013	RRID:SCR_004463	https://github.com/alexdobin/STAR
Software, algorithm	Harmony	Korsunsky et al., 2019	RRID:SCR_022206	https://github.com/immunogenomics/harmony
