# Supplementary material for: Parkinson’s disease-associated PINK1 loss disrupts ensheathing glia and causes dopaminergic neuron synapse loss
Source: eLife. 2026 Aug 13;14:RP105386. doi: 10.7554/eLife.105386 (PMC13472629; doi:10.7554/eLife.105386)
Supplement: Supplementary file 1 [file elife-105386-supp1.pdf]

	baseMean	log2FoldCl	lfcSE	stat	pvalue	padj
TTLL6A	4750.559	-11.3181	1.051882	-10.7598	5.33E-27	4.28E-23
l(3)07882	1189.898	-10.1921	1.303389	-7.81967	5.30E-15	2.13E-11
CG10936	791.4433	-10.6891	1.517311	-7.04474	1.86E-12	4.97E-09
CG15011	937.1086	-9.15508	1.333765	-6.86408	6.69E-12	1.34E-08
CG7627	1298.357	7.298828	1.109944	6.575853	4.84E-11	7.77E-08
CG10799	2865.974	8.638146	1.35321	6.383447	1.73E-10	1.86E-07
Nos	945.041	7.084464	1.111621	6.373091	1.85E-10	1.86E-07
CG10646	546.699	10.6305	1.665295	6.383556	1.73E-10	1.86E-07
Rhau	911.6146	8.130053	1.30148	6.246776	4.19E-10	3.74E-07
CG17660	2133.143	-6.28132	1.015593	-6.18488	6.21E-10	4.99E-07
Proc	2353.574	-7.82446	1.278466	-6.12019	9.35E-10	6.25E-07
FASN2	738.2926	-8.90389	1.451616	-6.13377	8.58E-10	6.25E-07
fiz	953.792	7.856676	1.299757	6.044726	1.50E-09	9.24E-07
Rpp25	822.1356	7.853897	1.304709	6.019656	1.75E-09	1.00E-06
PIG-B	2082.336	-6.23226	1.047009	-5.95244	2.64E-09	1.41E-06
Lnpk	1258.999	9.121406	1.536087	5.93808	2.88E-09	1.45E-06
hfw	2664.466	-7.57176	1.278148	-5.92401	3.14E-09	1.48E-06
CG2126	1440.227	7.189026	1.224281	5.87204	4.30E-09	1.92E-06
lncRNA:CR	451.2195	11.34112	1.934365	5.862969	4.55E-09	1.92E-06
spag	1089.173	-10.2702	1.755652	-5.8498	4.92E-09	1.98E-06
Prpk	976.9641	-11.1885	1.922361	-5.82017	5.88E-09	2.25E-06
CG31370	978.5736	-8.8501	1.539494	-5.74871	8.99E-09	3.28E-06
CG17712	2483.84	5.98876	1.043279	5.740324	9.45E-09	3.30E-06
achi	416.894	-11.9462	2.117389	-5.64196	1.68E-08	5.62E-06
Slc25A46a	1861.854	6.855485	1.219843	5.619975	1.91E-08	6.13E-06
CG34232	848.2188	10.83669	1.94161	5.581289	2.39E-08	7.37E-06
NiPp1	656.2872	9.111064	1.640156	5.554997	2.78E-08	8.11E-06
DUBAI	1722.491	5.431373	0.978346	5.551585	2.83E-08	8.11E-06
CG12773	1275.05	8.915078	1.614981	5.520236	3.39E-08	9.35E-06
tadr	1560.365	5.683654	1.032008	5.507375	3.64E-08	9.35E-06
CG30357	397.499	11.84152	2.151088	5.504899	3.69E-08	9.35E-06
Fic	1217.805	-8.27567	1.503747	-5.50336	3.73E-08	9.35E-06
Ccz1	475.0219	8.548615	1.564171	5.46527	4.62E-08	1.12E-05
Atac1	426.3187	9.930944	1.827597	5.43388	5.51E-08	1.29E-05
CG30424	1121.918	-6.02376	1.109398	-5.42975	5.64E-08	1.29E-05
CG9004	617.8992	9.572376	1.766005	5.420355	5.95E-08	1.33E-05
Rel	935.8779	8.204279	1.516904	5.408568	6.35E-08	1.38E-05
Ada2a	2019.076	-10.2619	1.901264	-5.39742	6.76E-08	1.43E-05
CG32532	1547.466	-5.87735	1.091874	-5.38281	7.33E-08	1.51E-05
firl	1009.197	-8.02402	1.512882	-5.3038	1.13E-07	2.28E-05
Dark	573.9756	8.110682	1.533639	5.288523	1.23E-07	2.41E-05
CG17612	1020.991	12.03429	2.295015	5.243666	1.57E-07	3.01E-05
pwn	1323.361	7.869095	1.503215	5.234844	1.65E-07	3.08E-05
Atg7	836.3293	7.916463	1.517504	5.216767	1.82E-07	3.32E-05
Pld	1231.724	-5.67404	1.089622	-5.20735	1.92E-07	3.42E-05
CG6867	1366.312	7.862875	1.513154	5.196348	2.03E-07	3.55E-05
Wdr33	1005.735	7.185195	1.390908	5.165829	2.39E-07	4.09E-05
M6	951.0725	-7.28518	1.413304	-5.15471	2.54E-07	4.25E-05
GMF	879.7349	7.398752	1.445361	5.118966	3.07E-07	4.93E-05

Camp	1876.748	6.519438	1.272894	5.121747	3.03E-07	4.93E-05
Cyfip	1000.608	7.178368	1.406078	5.105241	3.30E-07	5.20E-05
Tpc1	883.0577	9.192462	1.802175	5.100761	3.38E-07	5.22E-05
mira	1037.83	-7.15616	1.407081	-5.08582	3.66E-07	5.54E-05
Cyp6w1	1435.289	6.583727	1.297643	5.073606	3.90E-07	5.80E-05
CG34347	1347.73	-7.85718	1.552718	-5.06028	4.19E-07	6.11E-05
Rh4	300.8536	-11.9419	2.363857	-5.05188	4.37E-07	6.27E-05
dnt	855.0223	7.602648	1.512799	5.02555	5.02E-07	6.95E-05
Usp20-33	1164.981	-7.6412	1.520467	-5.02556	5.02E-07	6.95E-05
CG6428	761.7536	6.031842	1.208578	4.990858	6.01E-07	8.18E-05
GstD2	639.3842	-11.612	2.338095	-4.96643	6.82E-07	8.99E-05
lncRNA:CR	759.4647	-8.82499	1.777071	-4.96603	6.83E-07	8.99E-05
CG9547	548.1134	-10.467	2.125966	-4.92339	8.51E-07	0.00011
pad	763.5107	10.42587	2.119736	4.918474	8.72E-07	0.000111
CG4603	753.7642	6.627124	1.348244	4.915374	8.86E-07	0.000111
CG6638	473.3985	11.66592	2.378263	4.905225	9.33E-07	0.000115
CG7601	498.7434	-9.68964	1.976787	-4.90171	9.50E-07	0.000116
asRNA:CR4	363.8035	10.10342	2.072028	4.8761	1.08E-06	0.00013
CG4676	1262.173	8.907078	1.840739	4.838859	1.31E-06	0.000154
dnk	1080.378	-7.87587	1.628822	-4.83531	1.33E-06	0.000155
mre11	532.8426	-8.54291	1.771117	-4.82346	1.41E-06	0.000162
ppk12	588.873	-11.6578	2.426559	-4.80426	1.55E-06	0.000176
alpha-Est8	1375.689	-6.02904	1.262605	-4.77508	1.80E-06	0.0002
Csl4	715.1897	7.912328	1.661571	4.761956	1.92E-06	0.000211
Mau2	534.9165	8.877389	1.870235	4.746671	2.07E-06	0.000218
Dhap-at	926.5075	6.023835	1.26839	4.7492	2.04E-06	0.000218
CG4594	305.5028	-10.6636	2.245392	-4.74908	2.04E-06	0.000218
CG5704	317.6176	9.988691	2.112231	4.728976	2.26E-06	0.000235
nst	484.3521	-11.1984	2.374566	-4.71599	2.41E-06	0.000248
CG10171	748.0433	8.011489	1.702111	4.706797	2.52E-06	0.000256
CG15436	515.9871	-11.3495	2.4133	-4.70289	2.57E-06	0.000257
CG4627	1152.258	7.021276	1.505195	4.664695	3.09E-06	0.000306
CG30491	1428.071	6.5103	1.399045	4.653388	3.27E-06	0.00032
adp	334.5566	9.035789	1.946097	4.643032	3.43E-06	0.000332
CG13609	260.4131	-9.63685	2.082569	-4.62739	3.70E-06	0.000342
asRNA:CR4	631.3889	-9.24778	1.998025	-4.62846	3.68E-06	0.000342
CG6793	430.3684	10.53007	2.272193	4.634319	3.58E-06	0.000342
Blos2	438.3986	7.294316	1.57518	4.630783	3.64E-06	0.000342
Pyroxd1	1170.982	-6.8667	1.486109	-4.62059	3.83E-06	0.000345
Pdxk	1438.566	-6.33072	1.369757	-4.62178	3.80E-06	0.000345
CG16789	607.5918	7.164701	1.552375	4.615317	3.92E-06	0.00035
CG6830	539.0547	-10.0033	2.17103	-4.60763	4.07E-06	0.000359
CG9246	369.4685	-8.24787	1.792973	-4.60011	4.22E-06	0.000368
Est-Q	469.7577	-7.83016	1.707343	-4.58617	4.51E-06	0.00039
CG12448	399.8078	-9.48382	2.070497	-4.58046	4.64E-06	0.000396
Syx18	616.1109	-8.35214	1.827677	-4.56981	4.88E-06	0.000412
CG13151	432.9034	-11.6207	2.550673	-4.55594	5.22E-06	0.000436
Ror	1022.192	6.577376	1.444827	4.552361	5.30E-06	0.000439
CG6550	585.0607	-7.65072	1.684011	-4.54315	5.54E-06	0.000454
pelo	931.3385	-6.94618	1.531768	-4.53475	5.77E-06	0.000468

PIG-A	493.3573	9.592711	2.116743	4.531826	5.85E-06	0.000469
alpha-PheI	775.3718	-6.84436	1.513692	-4.52163	6.14E-06	0.000488
coil	366.9633	-10.1972	2.256625	-4.5188	6.22E-06	0.000489
Cyp28d2	695.7862	9.641996	2.14127	4.502934	6.70E-06	0.000522
phr	430.8601	-7.81265	1.737268	-4.49709	6.89E-06	0.000532
HisCl1	1214.398	-6.87144	1.530979	-4.48826	7.18E-06	0.000544
CG33978	333.9723	-11.3906	2.537149	-4.48954	7.14E-06	0.000544
CG8379	706.0137	8.454017	1.88676	4.480706	7.44E-06	0.000554
Dip-B	354.8804	10.39864	2.323962	4.474529	7.66E-06	0.000554
CG10321	639.2847	-8.6795	1.940645	-4.47248	7.73E-06	0.000554
CG7206	2811.474	-3.3786	0.754762	-4.47638	7.59E-06	0.000554
Rpc53	790.2336	7.802903	1.744342	4.473264	7.70E-06	0.000554
MTF-1	687.6926	-7.04686	1.572933	-4.48008	7.46E-06	0.000554
CG34357	1161.319	4.89049	1.094028	4.470168	7.82E-06	0.000555
CG15107	745.3173	-6.63378	1.485867	-4.46458	8.02E-06	0.000565
CG2614	641.2481	-7.44849	1.670142	-4.45979	8.20E-06	0.000569
CG9008	645.4038	-8.10254	1.816992	-4.45931	8.22E-06	0.000569
CG6144	1510.144	-5.55041	1.246265	-4.45363	8.44E-06	0.000579
CG10623	751.1332	6.476712	1.456945	4.445406	8.77E-06	0.000597
CG8298	1073.967	-6.42129	1.447333	-4.43664	9.14E-06	0.000616
obst-A	518.7521	9.031956	2.042299	4.422446	9.76E-06	0.000653
CG14722	586.0011	-7.42685	1.681661	-4.41638	1.00E-05	0.000666
CG1602	604.9766	10.05862	2.285526	4.401008	1.08E-05	0.000709
Rcd2	1008.269	6.907473	1.576127	4.382561	1.17E-05	0.000765
betaTub6C	2092.102	-5.43934	1.242521	-4.37767	1.20E-05	0.000776
ru	641.2174	-9.49179	2.1719	-4.37027	1.24E-05	0.000797
CG5292	580.8667	-6.52555	1.497765	-4.35686	1.32E-05	0.00084
dx	661.8949	-9.69874	2.231913	-4.34548	1.39E-05	0.000875
TfIIEalpha	1301.796	6.650406	1.530728	4.344604	1.40E-05	0.000875
CG5641	2018.42	-5.22316	1.203806	-4.33887	1.43E-05	0.000891
RluA-2	622.5085	-7.71479	1.780042	-4.33405	1.46E-05	0.000904
CG12219	674.1425	-8.00116	1.848699	-4.32799	1.50E-05	0.000915
cos	1072.298	-6.63968	1.533869	-4.32872	1.50E-05	0.000915
fu	499.4366	7.900491	1.829059	4.31943	1.56E-05	0.000944
ymp	335.7695	-10.8397	2.51436	-4.3111	1.62E-05	0.000973
CG5555	1051.403	5.941167	1.381806	4.299565	1.71E-05	0.001017
CG4951	606.0654	-7.95497	1.857217	-4.28328	1.84E-05	0.001079
rod	491.4583	-9.14753	2.135462	-4.28363	1.84E-05	0.001079
otp	723.9208	6.966963	1.629643	4.275148	1.91E-05	0.001111
CG3552	357.4748	7.997576	1.871768	4.272738	1.93E-05	0.001115
CG3407	574.2291	6.368619	1.49309	4.265396	2.00E-05	0.001144
CG13097	754.3208	-7.46558	1.757137	-4.24872	2.15E-05	0.001224
rig	369.5297	8.56305	2.018031	4.243271	2.20E-05	0.001245
CG17802	735.7165	7.532725	1.777363	4.238146	2.25E-05	0.001256
CG5880	637.0677	-7.02653	1.657498	-4.23924	2.24E-05	0.001256
Camta	1862.904	3.638238	0.862456	4.218462	2.46E-05	0.001362
CCHa2	523.4574	-8.118	1.925747	-4.21551	2.49E-05	0.00137
asRNA:CR4	243.6834	-10.7339	2.54745	-4.21357	2.51E-05	0.001372
CG3703	1143.821	6.486977	1.540411	4.211198	2.54E-05	0.001378
Smyd4-4	1077.187	5.559314	1.32504	4.195581	2.72E-05	0.001466

Alg10	907.2355	-7.90167	1.888532	-4.18403	2.86E-05	0.001532
Alg3	343.8811	-7.80808	1.86933	-4.17694	2.95E-05	0.00157
Adgf-C	1431.711	-6.2167	1.48995	-4.17242	3.01E-05	0.001571
CG11779	520.3206	7.336643	1.757379	4.174764	2.98E-05	0.001571
CG7208	553.4062	8.881809	2.128733	4.172346	3.01E-05	0.001571
IntS1	536.0844	-7.13695	1.711473	-4.17006	3.05E-05	0.001577
Rpp30	434.4003	-11.3755	2.731802	-4.16409	3.13E-05	0.001604
sl	313.5023	-7.13075	1.713265	-4.16208	3.15E-05	0.001604
ssp6	1191.295	-5.51122	1.324253	-4.16176	3.16E-05	0.001604
CG17026	1582.285	-5.55945	1.336467	-4.15981	3.19E-05	0.001608
PSR	450.0398	-7.96811	1.918847	-4.15255	3.29E-05	0.001649
Sfmbt	889.3905	6.560389	1.582526	4.145517	3.39E-05	0.00168
CG1941	358.1842	10.41666	2.512683	4.145632	3.39E-05	0.00168
Mesh1	201.0673	-11.3017	2.729385	-4.14075	3.46E-05	0.001696
CG42327	2142.227	-3.69198	0.891673	-4.14051	3.47E-05	0.001696
Dpit47	382.5126	-10.001	2.41638	-4.13885	3.49E-05	0.001698
lncRNA:CR	230.0664	9.197144	2.228907	4.126302	3.69E-05	0.001782
trbl	220.8146	-10.992	2.665278	-4.12415	3.72E-05	0.001788
Dbp21E2	470.2418	9.106004	2.212632	4.115462	3.86E-05	0.001846
CG32344	479.2301	8.142051	1.984282	4.103274	4.07E-05	0.001935
Cyp12b2	3795.63	-2.76883	0.675607	-4.09829	4.16E-05	0.001965
CG17726	572.5821	6.852312	1.674336	4.092554	4.27E-05	0.002003
CG4935	885.0364	7.371235	1.808744	4.075334	4.59E-05	0.002132
CG8152	438.2612	-9.28722	2.278704	-4.07566	4.59E-05	0.002132
CG2794	311.3938	-9.83769	2.417395	-4.06954	4.71E-05	0.002148
crok	342.0966	-9.64099	2.368509	-4.07049	4.69E-05	0.002148
tex	325.5767	8.753308	2.150543	4.070278	4.70E-05	0.002148
CG31457	490.7809	8.547122	2.102339	4.065529	4.79E-05	0.002173
CysRS-m	346.5391	7.146401	1.761913	4.056047	4.99E-05	0.00225
CG12288	503.5113	-7.00612	1.728305	-4.05375	5.04E-05	0.00226
spirit	234.8125	9.373786	2.313414	4.051928	5.08E-05	0.002265
TM9SF3	2296.193	4.931159	1.217635	4.049784	5.13E-05	0.002273
ssp	370.5257	-8.4051	2.07813	-4.04455	5.24E-05	0.002312
CG14589	231.2725	-10.685	2.650055	-4.03198	5.53E-05	0.002412
CG30291	928.9416	-7.99955	1.984611	-4.03079	5.56E-05	0.002412
CstF64	621.4064	-8.5998	2.132895	-4.03199	5.53E-05	0.002412
CG10949	447.0743	-6.89439	1.711005	-4.02944	5.59E-05	0.002413
Rae1	1056.038	-5.60417	1.391323	-4.02794	5.63E-05	0.002415
jnj	362.518	8.214528	2.040043	4.026644	5.66E-05	0.002415
l(2)10685	493.5839	5.847429	1.452879	4.024719	5.70E-05	0.002422
CG13773	324.8891	-7.90106	1.968229	-4.0143	5.96E-05	0.002505
CG30466	227.1361	7.940211	1.977519	4.01524	5.94E-05	0.002505
lncRNA:CR	272.0478	8.888315	2.216865	4.009407	6.09E-05	0.002505
RAF2	641.6294	-8.58163	2.139269	-4.01148	6.03E-05	0.002505
CG7011	190.8441	-11.2011	2.792507	-4.01113	6.04E-05	0.002505
CG17593	2006.614	-4.19264	1.045596	-4.00981	6.08E-05	0.002505
cv-d	979.4725	5.91847	1.476721	4.007846	6.13E-05	0.002509
Aos1	787.3889	6.320943	1.578301	4.004903	6.20E-05	0.002528
CG17359	281.0305	11.20301	2.799852	4.001285	6.30E-05	0.002554
CG11334	390.1485	-11.5608	2.897887	-3.98938	6.62E-05	0.002672

lncRNA:CR	207.9927	10.75406	2.697993	3.985948	6.72E-05	0.002697
Idgf1	606.467	-7.60776	1.909333	-3.98451	6.76E-05	0.0027
CSN7	388.4597	6.301425	1.582063	3.983042	6.80E-05	0.002703
CG11436	942.9732	7.477989	1.879286	3.979166	6.92E-05	0.002732
Cyp28c1	1835.342	-4.4683	1.123189	-3.97822	6.94E-05	0.002732
CG12267	339.2301	-8.31067	2.092897	-3.97089	7.16E-05	0.002776
ProRS-m	237.4179	-10.3161	2.597517	-3.97153	7.14E-05	0.002776
CG9609	1001.69	6.020454	1.515515	3.972547	7.11E-05	0.002776
Rich	499.5593	-8.00467	2.01887	-3.96493	7.34E-05	0.002819
Upf2	335.5828	-8.97987	2.264212	-3.966	7.31E-05	0.002819
CG6347	310.4918	-8.9772	2.268101	-3.95802	7.56E-05	0.002888
CG15118	1714.726	3.908311	0.98828	3.954661	7.66E-05	0.002915
Plzf	625.5818	7.397519	1.872025	3.951613	7.76E-05	0.002939
Ugt302C1	218.0225	-9.5825	2.426706	-3.94877	7.86E-05	0.002947
Irbp	202.0334	-10.8426	2.745879	-3.94867	7.86E-05	0.002947
Drep3	555.8074	7.857601	1.991845	3.944886	7.98E-05	0.00298
thoc5	208.8412	-8.97273	2.275251	-3.94362	8.03E-05	0.002982
CG12814	381.5944	-7.88869	2.003464	-3.93752	8.23E-05	0.003045
Fam92	536.6875	-5.95175	1.513715	-3.93188	8.43E-05	0.003073
CG8531	1277.986	-6.6103	1.68119	-3.93192	8.43E-05	0.003073
U2A	738.656	-6.83666	1.739676	-3.92985	8.50E-05	0.003073
cana	296.5751	-7.14343	1.816001	-3.93361	8.37E-05	0.003073
l(2)37Cg	473.8694	6.68439	1.70059	3.930629	8.47E-05	0.003073
Cerk	2206.886	-2.90162	0.738891	-3.92699	8.60E-05	0.003096
TBC1d7	303.1996	-7.81456	1.991507	-3.92394	8.71E-05	0.003121
CG12224	490.9956	-7.04642	1.797329	-3.9205	8.84E-05	0.003152
CG18094	1190.452	5.379339	1.372823	3.91845	8.91E-05	0.003165
CG18622	353.4835	-8.32758	2.129344	-3.91087	9.20E-05	0.003252
Mics1	390.3873	9.343207	2.391251	3.907246	9.34E-05	0.003286
CG15743	613.4656	-7.39825	1.895894	-3.90225	9.53E-05	0.00334
Hira	381.2118	-8.18672	2.100676	-3.89718	9.73E-05	0.003396
Sin	385.0068	-7.8396	2.014992	-3.89064	1.00E-04	0.003455
DIP-zeta	437.2753	-11.0154	2.83224	-3.8893	0.000101	0.003455
CG10602	802.7276	-6.13685	1.578073	-3.88883	0.000101	0.003455
CG17027	682.8516	6.153834	1.581975	3.88997	0.0001	0.003455
MED8	503.0481	-7.6591	1.973148	-3.88167	0.000104	0.003528
LeuRS	854.3417	-6.01486	1.549283	-3.88235	0.000103	0.003528
CG2972	342.6367	8.260128	2.131228	3.875759	0.000106	0.0036
Cyp6a17	4346.845	-2.49194	0.644138	-3.86864	0.000109	0.003675
CG42393	266.6757	-8.92492	2.306571	-3.86934	0.000109	0.003675
CG7130	403.2081	7.993299	2.066729	3.867608	0.00011	0.003676
TTLL15	596.5043	-8.45342	2.188397	-3.86284	0.000112	0.003732
CG15084	396.396	-9.11404	2.359995	-3.86189	0.000113	0.003732
Mtr4	413.491	6.240751	1.617775	3.857615	0.000114	0.003769
CG8128	320.5576	-8.61998	2.234621	-3.85747	0.000115	0.003769
SiaT	450.8944	8.684454	2.252927	3.854742	0.000116	0.003795
WDR79	782.8013	7.567826	1.964517	3.852257	0.000117	0.003818
Uba4	532.9401	-7.37356	1.916329	-3.84775	0.000119	0.003873
lncRNA:CR	243.5297	-10.4376	2.714122	-3.84567	0.00012	0.003875
Ing3	284.602	-7.62149	1.981818	-3.8457	0.00012	0.003875

Dhod	707.3627	-5.69567	1.484058	-3.8379	0.000124	0.003953
Rpt5	1092.509	-5.91004	1.539922	-3.83788	0.000124	0.003953
Hyccin	696.6034	6.94532	1.809002	3.83931	0.000123	0.003953
NANS	1057.412	-4.33274	1.129265	-3.83678	0.000125	0.003955
chm	789.445	-5.46329	1.425017	-3.83384	0.000126	0.003986
Art3	372.1751	11.12566	2.903438	3.831891	0.000127	0.004002
Vps33B	479.694	-9.79747	2.562587	-3.82327	0.000132	0.004129
CCHa1	1732.036	6.221105	1.627647	3.822148	0.000132	0.004132
CG30010	543.8857	6.767821	1.773099	3.816945	0.000135	0.004203
CG5498	948.1067	5.525568	1.448505	3.814671	0.000136	0.004226
Nrd1	483.5141	-5.34265	1.404843	-3.80302	0.000143	0.004413
KaiR1D	1733.27	4.229191	1.112863	3.800281	0.000145	0.004445
CG13016	355.2934	7.85528	2.06783	3.798805	0.000145	0.004454
Uros1	372.3157	-7.31633	1.92866	-3.79348	0.000149	0.004533
jumu	529.7174	-6.69663	1.765959	-3.79207	0.000149	0.004542
CG17141	352.7418	8.885027	2.345619	3.787924	0.000152	0.004587
CG7191	1381.679	-5.47969	1.446691	-3.78774	0.000152	0.004587
sip1	666.6298	7.233956	1.911704	3.784036	0.000154	0.004638
CG32191	464.2185	-5.8942	1.558824	-3.78118	0.000156	0.004674
Taf7	396.902	7.411811	1.962032	3.77762	0.000158	0.004691
trh	292.0806	-8.87308	2.348903	-3.77754	0.000158	0.004691
tut	1189.383	4.376568	1.158074	3.779179	0.000157	0.004691
NC2beta	740.7553	5.594179	1.481722	3.775458	0.00016	0.004713
Keap1	541.9236	7.560549	2.005221	3.770431	0.000163	0.004791
CG13408	605.9705	8.310606	2.206864	3.765799	0.000166	0.004863
CG15387	230.1346	-10.7056	2.848067	-3.75892	0.000171	0.004981
CG9706	1015.245	-4.98589	1.328348	-3.75345	0.000174	0.005072
CG11168	360.3669	-9.8921	2.636829	-3.75151	0.000176	0.005079
CG16896	399.7606	-6.29843	1.679009	-3.75128	0.000176	0.005079
CG42553	283.3301	-9.15774	2.4429	-3.74872	0.000178	0.005113
UbcE2M	1278.138	-3.28822	0.877515	-3.74719	0.000179	0.005126
CG2681	297.3664	-10.35	2.767218	-3.74023	0.000184	0.005251
Rev1	1074.932	7.303795	1.953312	3.739185	0.000185	0.005254
CG12129	431.8526	-8.03781	2.150822	-3.73709	0.000186	0.00528
CG33051	382.5251	-7.11048	1.906592	-3.72942	0.000192	0.005424
CG8067	500.172	-8.79919	2.364193	-3.72186	0.000198	0.005561
IleRS-m	562.8845	-8.31867	2.235385	-3.72136	0.000198	0.005561
CG9626	173.9439	-10.7308	2.891439	-3.71123	0.000206	0.005754
Gcn5	1529.812	3.272344	0.8818	3.710982	0.000206	0.005754
CG18449	214.4182	-9.78152	2.637669	-3.7084	0.000209	0.005793
ntc	294.2206	10.75939	2.908566	3.699209	0.000216	0.005965
Dph1	1316.282	5.742012	1.551933	3.69991	0.000216	0.005965
CG9302	1246.95	-5.41874	1.465815	-3.69674	0.000218	0.006003
CG6345	226.0583	-10.6879	2.896854	-3.68948	0.000225	0.006155
CG13876	259.6387	-8.47047	2.297426	-3.68694	0.000227	0.006196
CG14906	271.7683	7.37445	2.00181	3.683891	0.00023	0.006249
CG43091	299.2089	-8.55033	2.324508	-3.67834	0.000235	0.006365
CG34183	357.9084	-7.34043	1.998076	-3.67375	0.000239	0.006459
Psf2	226.8873	10.37649	2.829876	3.666765	0.000246	0.006616
CG31633	535.2604	7.131974	1.946534	3.663935	0.000248	0.006667

CG6910	507.0548	8.230874	2.248401	3.660767	0.000251	0.006727
CG2875	862.5305	5.048583	1.38635	3.641636	0.000271	0.007224
senju	390.4874	-8.65438	2.379083	-3.63769	0.000275	0.007263
DIP-eta	372.7961	6.311828	1.734796	3.638369	0.000274	0.007263
CG8728	1945.347	-3.65005	1.003173	-3.6385	0.000274	0.007263
ko	419.3295	6.308475	1.743579	3.618119	0.000297	0.007777
CG14535	1233.562	3.410078	0.943103	3.615805	0.000299	0.007777
Reg-5	383.1895	-7.10121	1.963721	-3.6162	0.000299	0.007777
Usp39	436.3149	-8.60387	2.378556	-3.61726	0.000298	0.007777
skl	314.2048	9.030236	2.495126	3.61915	0.000296	0.007777
Strn-Mlck	763.9584	-4.93127	1.369471	-3.60086	0.000317	0.008211
Nup54	315.1	10.09361	2.807216	3.595595	0.000324	0.008352
MFS16	359.6643	9.821248	2.734476	3.591638	0.000329	0.008453
mRRF1	264.677	-10.549	2.937842	-3.59074	0.00033	0.008455
Akt1	947.1016	-5.10935	1.425798	-3.5835	0.000339	0.008666
LKRSDH	192.7737	-10.6853	2.984686	-3.58003	0.000344	0.008753
Taf5	833.4791	-6.07965	1.700923	-3.57432	0.000351	0.008918
CG5114	1311.668	-5.0184	1.40511	-3.57154	0.000355	0.008985
aph-1	623.8694	6.354992	1.780629	3.568958	0.000358	0.009046
Kr-h2	4153.233	2.842019	0.798998	3.556978	0.000375	0.009439
Nct	822.04	5.820405	1.637209	3.555077	0.000378	0.009477
Loxl1	205.3145	10.15359	2.859638	3.550656	0.000384	0.009608
Cp110	253.5559	10.38475	2.928775	3.545767	0.000391	0.009758
r2d2	626.9538	-4.19037	1.182723	-3.54298	0.000396	0.009831
lncRNA:CR	244.8413	-8.59186	2.427854	-3.53887	0.000402	0.009954
Uhg8	257.8805	-8.85452	2.504133	-3.53596	0.000406	0.010034
CG31251	299.1629	-8.71856	2.467166	-3.53384	0.00041	0.010053
CG4882	1413.123	-3.98982	1.128984	-3.53399	0.000409	0.010053
spn-A	1079.667	3.7897	1.072856	3.532348	0.000412	0.010079
CG7131	477.8142	8.588082	2.433788	3.528689	0.000418	0.010152
CG7747	282.8831	8.147512	2.309032	3.52854	0.000418	0.010152
por	366.7378	-7.72816	2.190506	-3.52803	0.000419	0.010152
Pez	499.477	-5.4802	1.554163	-3.52615	0.000422	0.010193
Caf1-55	1054.57	-4.84443	1.376359	-3.51974	0.000432	0.010411
Ercc1	180.7639	-9.37286	2.670403	-3.5099	0.000448	0.010772
lncRNA:CR	385.6089	7.219855	2.057561	3.508939	0.00045	0.010779
Asciz	332.4081	-7.03236	2.005208	-3.50705	0.000453	0.010823
CG14131	300.2395	8.52298	2.438512	3.495156	0.000474	0.011284
Act79B	600.4079	-6.70352	1.919909	-3.49158	0.00048	0.011308
CG9395	2305.633	2.634085	0.754436	3.491464	0.00048	0.011308
CG8740	319.0128	-8.82065	2.52568	-3.49239	0.000479	0.011308
CG17030	306.4606	9.777462	2.800415	3.491434	0.00048	0.011308
ste24b	344.2349	9.395568	2.692235	3.489877	0.000483	0.011341
esc	310.8567	-8.0532	2.309069	-3.48764	0.000487	0.011403
CG11170	243.071	8.134664	2.333967	3.485339	0.000492	0.011461
hzg	6959.567	-2.09789	0.60203	-3.48469	0.000493	0.011461
Cpr67B	275.1188	8.973047	2.575538	3.48395	0.000494	0.011461
lncRNA:CR	253.743	7.890796	2.265579	3.482905	0.000496	0.011472
CG14102	186.1773	10.79347	3.100895	3.480758	0.0005	0.011532
CG8326	204.4709	-8.63954	2.483313	-3.47904	0.000503	0.011573

slif	249.2391	10.77482	3.099956	3.475799	0.000509	0.01168
l(2)k01209	896.2943	5.946463	1.711879	3.473647	0.000513	0.011707
CG14764	1841.654	3.488649	1.004107	3.474378	0.000512	0.011707
ECSIT	843.2693	-3.7059	1.068319	-3.46891	0.000523	0.011721
Zip88E	168.2501	9.323875	2.68737	3.469517	0.000521	0.011721
Eip55E	1125.887	-4.97588	1.434065	-3.46978	0.000521	0.011721
CG8314	368.3937	-6.75039	1.944553	-3.47143	0.000518	0.011721
CR18166	1351.337	-4.74364	1.366116	-3.47235	0.000516	0.011721
Vps8	274.5246	-6.27679	1.809502	-3.46879	0.000523	0.011721
CG6752	592.638	-6.3682	1.840316	-3.46038	0.000539	0.012059
CG4407	495.2125	5.638813	1.630502	3.458329	0.000544	0.012118
Hs3st-B	672.8181	5.845631	1.692962	3.452901	0.000555	0.01233
CG14785	173.0263	10.12	2.940355	3.441763	0.000578	0.012811
htl	2461.282	-2.60372	0.756661	-3.44106	0.000579	0.012811
CG15547	268.5494	-7.51151	2.18386	-3.43955	0.000583	0.012848
CG8478	551.4226	6.817863	1.982794	3.438513	0.000585	0.012862
mt:lrRNA	406634.3	1.225081	0.356418	3.437201	0.000588	0.012889
DIP-epsilon	456.2795	5.700916	1.662387	3.429356	0.000605	0.013231
Zip48C	497.4721	6.833859	1.994191	3.426883	0.000611	0.013315
CG7194	323.9607	-7.26066	2.119176	-3.42617	0.000612	0.013315
scramb2	1511.706	-4.16989	1.217534	-3.42486	0.000615	0.013343
RagC-D	2362.087	-2.55488	0.746699	-3.42157	0.000623	0.013469
CG32590	541.4213	6.765557	1.977874	3.42062	0.000625	0.01348
Cdk2	904.3587	5.045419	1.476293	3.417627	0.000632	0.013556
Fdx1	402.4264	8.166966	2.389159	3.418344	0.00063	0.013556
CG3603	251.8577	-8.80068	2.57862	-3.41294	0.000643	0.013727
CG15784	632.6849	-7.81268	2.289246	-3.41277	0.000643	0.013727
PolD3	625.6669	5.490858	1.610047	3.410371	0.000649	0.013811
Cyp6d5	266.4088	-8.94168	2.63292	-3.39611	0.000684	0.014513
Ns4	942.7734	5.954801	1.75442	3.394171	0.000688	0.014577
Uch	2546.599	3.324232	0.981297	3.387589	0.000705	0.014892
CG11563	331.1363	-5.28506	1.56246	-3.38253	0.000718	0.01513
CG14483	254.102	-7.89056	2.336186	-3.37754	0.000731	0.015367
AhcyL2	306.8452	9.416037	2.794524	3.36946	0.000753	0.015759
Ids	1309.249	-3.84726	1.141905	-3.36916	0.000754	0.015759
PIG-F	948.425	-4.30601	1.278446	-3.36816	0.000757	0.015775
CG18343	1006.049	-3.59776	1.068869	-3.36595	0.000763	0.015861
eIF2D	226.6383	6.658608	1.980654	3.361823	0.000774	0.016058
Mettl14	347.0842	-8.00217	2.384406	-3.35604	0.000791	0.016355
CG12547	4535.787	-2.17667	0.648977	-3.354	0.000797	0.016434
CG1231	387.4295	-6.30563	1.882746	-3.34917	0.000811	0.016681
l(2)03659	191.0695	9.918963	2.964133	3.346328	0.000819	0.016766
Rnmt	250.6406	7.773669	2.322942	3.346476	0.000818	0.016766
SecS	2515.165	3.178664	0.95113	3.341986	0.000832	0.016952
mute	152.2992	-9.53876	2.854328	-3.34186	0.000832	0.016952
CG6833	246.5426	-8.46486	2.533566	-3.34108	0.000835	0.016957
CG6227	710.4709	4.686905	1.403516	3.339402	0.00084	0.017017
CG10550	1895.179	4.201064	1.259703	3.334964	0.000853	0.017204
Lst8	333.7743	-7.31565	2.193236	-3.33555	0.000851	0.017204
CG15525	183.9904	8.011043	2.404609	3.331537	0.000864	0.017304

CG7857	220.6829	8.751744	2.627155	3.331263	0.000865	0.017304
CG6024	4638.892	2.072432	0.622119	3.331248	0.000865	0.017304
asRNA:CR4	189.6959	9.893587	2.971658	3.329315	0.000871	0.017382
Exo70	388.5384	-6.59708	1.982731	-3.32727	0.000877	0.017466
pps	1011.266	4.407321	1.325195	3.325791	0.000882	0.017516
CG18011	371.5926	-8.05208	2.422241	-3.32423	0.000887	0.017571
polybromo	424.3416	-6.7659	2.035927	-3.32325	0.00089	0.017589
btz	914.6689	-4.55535	1.371975	-3.32029	0.000899	0.017733
CG10616	1051.758	-3.83995	1.156762	-3.31956	0.000902	0.017736
CG7497	420.8888	-6.99422	2.107758	-3.31832	0.000906	0.017771
mTTF	362.3099	7.504594	2.262328	3.317201	0.000909	0.017799
CG1129	532.725	-5.66945	1.709793	-3.31587	0.000914	0.017806
CG10467	647.4067	6.810735	2.054071	3.315726	0.000914	0.017806
twit	272.4656	7.90224	2.385011	3.313293	0.000922	0.017875
Cortactin	1798.371	-3.11755	0.940814	-3.31367	0.000921	0.017875
pdm2	1182.603	4.790153	1.447344	3.309616	0.000934	0.017981
Sirt6	357.8013	-6.47529	1.956366	-3.30986	0.000933	0.017981
Pif1A	296.7722	-8.26454	2.49707	-3.30969	0.000934	0.017981
CG8003	362.1163	-7.14342	2.159766	-3.3075	0.000941	0.018075
CG13096	344.2302	6.120375	1.85267	3.303543	0.000955	0.018288
BCL7-like	241.198	-8.27431	2.505907	-3.30192	0.00096	0.01835
Naxd	378.7256	7.110286	2.155085	3.299307	0.000969	0.018478
mab-21	484.5247	5.621723	1.704856	3.297476	0.000976	0.018555
Psc	440.7514	-6.98255	2.118231	-3.29641	0.000979	0.018581
CG13604	1563.386	4.385104	1.331175	3.29416	0.000987	0.018686
Dlip1	219.1201	9.404478	2.857174	3.291532	0.000996	0.018817
Dph2	485.8629	-4.98967	1.517247	-3.28863	0.001007	0.018968
Hakai	259.1976	-7.70939	2.3455	-3.28689	0.001013	0.019041
Tip60	973.9042	3.890049	1.184822	3.283235	0.001026	0.019174
Atg14	1109.619	-4.58213	1.395266	-3.28406	0.001023	0.019174
CG12717	1887.21	4.238788	1.291153	3.282949	0.001027	0.019174
CG8908	281.9925	8.981193	2.742536	3.274776	0.001057	0.019692
CG7218	931.4355	5.282666	1.614116	3.272793	0.001065	0.019785
Ir68a	1107.767	-5.08113	1.553442	-3.27089	0.001072	0.019872
CG12883	569.3011	-7.16612	2.193845	-3.26647	0.001089	0.020105
ida	320.7105	7.995967	2.448026	3.266292	0.00109	0.020105
DIP-lambd	406.5989	-6.57254	2.012738	-3.26547	0.001093	0.020117
Gas41	536.9897	5.122634	1.569148	3.264595	0.001096	0.020133
Pym	541.4174	-5.58514	1.714182	-3.2582	0.001121	0.020545
mRRF2	498.7612	7.302163	2.242386	3.256425	0.001128	0.02058
CG1792	1010.659	4.07513	1.251318	3.256671	0.001127	0.02058
TyrRS	1220.483	3.43126	1.054094	3.255175	0.001133	0.020624
CG32944	250.7137	8.042536	2.471367	3.254287	0.001137	0.020642
CG5380	159.8673	-8.22409	2.531853	-3.24825	0.001161	0.021006
Mrtf	3643.333	-2.82294	0.869121	-3.24804	0.001162	0.021006
Plekhm1	201.5792	7.661095	2.359767	3.246547	0.001168	0.021021
CG33642	259.8787	8.190736	2.522661	3.246864	0.001167	0.021021
CG15365	193.9673	-7.23634	2.230426	-3.24438	0.001177	0.021135
lncRNA:CR	503.624	6.915868	2.133447	3.241641	0.001188	0.021244
nmdyn-D6	723.3789	6.292849	1.940895	3.242241	0.001186	0.021244

Dgp-1	773.7901	-4.17655	1.290005	-3.23762	0.001205	0.02148
CR14798	218.4587	10.29215	3.179942	3.236586	0.00121	0.02148
CG5897	254.6749	-8.4828	2.620751	-3.23678	0.001209	0.02148
CG7453	362.3817	6.167975	1.909654	3.229892	0.001238	0.021941
CG14881	428.6932	6.218247	1.926322	3.228041	0.001246	0.021946
CG1750	240.6317	-8.85493	2.742773	-3.22846	0.001245	0.021946
SIFa	8481.706	3.696151	1.145493	3.22669	0.001252	0.021946
E(var)3-9	272.7733	-8.97347	2.780712	-3.22704	0.001251	0.021946
Hph	1983.311	3.370618	1.044337	3.227518	0.001249	0.021946
brun	947.2972	4.710196	1.462455	3.220746	0.001279	0.022357
Ent3	1261.506	4.156484	1.291158	3.219191	0.001286	0.02243
FER	1894.721	-3.36506	1.046042	-3.21694	0.001296	0.022512
trus	1739.267	2.977742	0.925658	3.216891	0.001296	0.022512
CG32075	454.5363	-4.91209	1.527417	-3.21595	0.0013	0.022538
Spn77Ba	1127.538	-3.62359	1.126974	-3.21533	0.001303	0.022538
CG13558	944.2302	-6.68242	2.078928	-3.21436	0.001307	0.022565
CG10195	313.0799	-6.53526	2.035345	-3.21089	0.001323	0.022763
Cad96Ca	205.45	6.82565	2.125959	3.210621	0.001324	0.022763
Cbp80	956.9911	-4.27861	1.332951	-3.20988	0.001328	0.022773
CG18467	1991.158	-2.63627	0.821798	-3.20793	0.001337	0.022879
tho2	2408.985	-2.19941	0.687213	-3.20048	0.001372	0.023429
CG5180	1157.47	-3.30293	1.033279	-3.19655	0.001391	0.0237
Rrp6	197.3971	9.208751	2.881931	3.195341	0.001397	0.023749
CG5023	249.0937	8.14376	2.551651	3.191565	0.001415	0.024011
CG10516	403.4278	-6.96909	2.191828	-3.17958	0.001475	0.024974
Uxt	185.8906	-10.7286	3.378247	-3.17578	0.001494	0.02525
sdk	1247.649	-3.63583	1.145516	-3.17396	0.001504	0.0253
Ent2	658.1535	4.553409	1.434879	3.173376	0.001507	0.0253
CG9143	508.7361	-4.85535	1.529518	-3.17443	0.001501	0.0253
lncRNA:CR	521.8903	7.691456	2.424282	3.172674	0.00151	0.025308
aux	1179.221	-4.03266	1.271841	-3.17073	0.001521	0.025335
CG4572	968.3636	5.249194	1.655081	3.171563	0.001516	0.025335
lncRNA:CR	181.3571	6.531592	2.06008	3.170552	0.001521	0.025335
CG7840	180.3894	-9.17453	2.8962	-3.16778	0.001536	0.025367
lectin-46Cl	271.0089	-7.98731	2.521147	-3.16813	0.001534	0.025367
CG33941	826.786	-4.23065	1.335269	-3.16839	0.001533	0.025367
Zfrp8	144.3175	-10.1382	3.200066	-3.16812	0.001534	0.025367
Nf-YC	575.0382	-4.22732	1.335117	-3.16625	0.001544	0.025448
aru	229.0191	-6.02552	1.903432	-3.16561	0.001548	0.025452
asRNA:CR4	129.3359	-9.43195	2.980061	-3.16502	0.001551	0.025452
SA	1061.037	-4.06664	1.286691	-3.16055	0.001575	0.025794
Dgkepsilor	1797.896	-2.59178	0.820449	-3.15898	0.001583	0.025827
CG32579	529.0231	7.268353	2.300806	3.159047	0.001583	0.025827
CG33958	1281.555	3.963367	1.254939	3.158215	0.001587	0.025842
CG32572	256.8676	-7.56015	2.394455	-3.15736	0.001592	0.025866
tgo	1306.939	4.154633	1.31642	3.156009	0.001599	0.025934
p38b	610.1778	5.326797	1.688235	3.155247	0.001604	0.025949
CCT6	1177.688	3.434527	1.089203	3.153246	0.001615	0.026075
Cdc45	710.5731	5.226303	1.660695	3.147058	0.001649	0.026549
CG5087	565.8672	-5.57265	1.770888	-3.14681	0.001651	0.026549

CG10361	2046.026	3.095165	0.983797	3.146143	0.001654	0.026556
Pgm1	3474.609	-2.40413	0.764433	-3.14498	0.001661	0.026608
CG6686	2117.859	-2.7711	0.881454	-3.14379	0.001668	0.026664
CG11404	234.2933	7.589843	2.418168	3.138675	0.001697	0.02708
Bsg25D	1912.231	2.870322	0.917723	3.127655	0.001762	0.02806
E(spl)m3-H	481.2278	5.394114	1.725276	3.126522	0.001769	0.028113
CG15725	420.5907	-5.72047	1.83056	-3.12498	0.001778	0.028204
Npl4	858.0111	-4.78774	1.532866	-3.12339	0.001788	0.028301
Adck5	145.3715	-11.2562	3.60687	-3.12077	0.001804	0.028499
gny	507.4095	5.019873	1.609154	3.119574	0.001811	0.028558
CG12204	326.7437	-8.37963	2.686849	-3.11876	0.001816	0.028572
CG4069	146.9021	-10.2128	3.275154	-3.11828	0.001819	0.028572
CG34220	345.882	-7.14562	2.292055	-3.11756	0.001824	0.028586
Hmr	143.8704	-7.86686	2.528288	-3.11154	0.001861	0.029092
CG14274	2330.026	2.308738	0.742204	3.110651	0.001867	0.029092
CG34180	166.6966	9.963093	3.202834	3.110712	0.001866	0.029092
eIF2Bdelta	460.6257	-5.69431	1.831414	-3.10924	0.001876	0.029175
Suv3	843.8438	5.348948	1.721428	3.107273	0.001888	0.029313
Vps13B	475.9299	-4.4053	1.418133	-3.10641	0.001894	0.029342
CG1103	335.3466	7.097025	2.286439	3.103965	0.001909	0.029529
CG43340	366.5888	-5.98607	1.928897	-3.10336	0.001913	0.029532
CG8768	1270.57	3.796387	1.223844	3.102019	0.001922	0.029609
Ntmt	320.8124	-7.29746	2.353693	-3.10043	0.001932	0.029712
Ns2	632.969	4.901191	1.583213	3.095724	0.001963	0.030072
CG17224	997.0162	-4.69924	1.517938	-3.0958	0.001963	0.030072
CG5828	311.7113	7.647859	2.473747	3.091609	0.001991	0.030434
Nprl2	264.0648	-8.42347	2.725847	-3.09022	0.002	0.030518
Pex2	497.2781	6.485832	2.106328	3.079212	0.002075	0.031609
lncRNA:CR	143.9626	-9.54791	3.103335	-3.07666	0.002093	0.03182
Hf	2394.058	-3.52923	1.148151	-3.07383	0.002113	0.032063
sxe2	748.8704	-6.00621	1.955373	-3.07165	0.002129	0.032182
CG14907	234.0529	-8.28743	2.698089	-3.07159	0.002129	0.032182
Mst87F	193.7006	-9.97514	3.24947	-3.06977	0.002142	0.032289
CG42339	1418.206	-4.39576	1.432086	-3.06948	0.002144	0.032289
lncRNA:CR	151.5731	10.88626	3.549792	3.066731	0.002164	0.032466
cpb	848.8881	-4.30298	1.403078	-3.06682	0.002164	0.032466
Pex3	2389.306	-2.21023	0.721061	-3.06525	0.002175	0.032567
CG4679	1045.302	3.632887	1.18708	3.060355	0.002211	0.033027
CG9512	732.9641	3.358927	1.097885	3.059451	0.002217	0.033027
smid	630.3253	-4.95243	1.618771	-3.05938	0.002218	0.033027
CG12512	843.8631	-4.96064	1.622511	-3.05739	0.002233	0.033175
CG14402	500.9265	6.672982	2.183077	3.056686	0.002238	0.033175
CG6040	4198.909	1.850666	0.605725	3.05529	0.002248	0.033175
boly	163.5486	-8.40763	2.751201	-3.05599	0.002243	0.033175
asRNA:CR4	187.7005	9.518524	3.115443	3.055271	0.002249	0.033175
AlkB	203.6788	-7.0195	2.302971	-3.04802	0.002304	0.033924
Exd2	291.5768	9.393876	3.082718	3.04727	0.002309	0.033946
Dph4	360.6354	-5.43862	1.788316	-3.0412	0.002356	0.034575
CG12698	213.9935	-7.82471	2.5755	-3.03813	0.00238	0.034865
unc-45	1380.353	4.545576	1.498299	3.033825	0.002415	0.035188

Gp150	740.1867	-4.61235	1.519949	-3.03454	0.002409	0.035188
Uba3	200.3697	7.652632	2.52254	3.033701	0.002416	0.035188
CG8461	192.838	8.20623	2.705873	3.032748	0.002423	0.035236
CG13982	818.9768	-4.07116	1.344046	-3.02904	0.002453	0.035607
noi	394.9077	-7.80853	2.578809	-3.02796	0.002462	0.035669
Cht11	550.4085	5.511126	1.822069	3.024653	0.002489	0.035997
daw	828.0157	-4.52335	1.496468	-3.02268	0.002505	0.036167
VhaSFD	9605.574	-1.86681	0.618606	-3.01778	0.002546	0.036691
CG17279	1719.317	-4.57191	1.516746	-3.01429	0.002576	0.037049
Task7	3641.67	3.02253	1.003922	3.010721	0.002606	0.037343
Hs6st	628.9733	-5.09381	1.692147	-3.01027	0.00261	0.037343
Arfrp1	951.6222	4.200139	1.395246	3.010321	0.00261	0.037343
Spps	732.8364	-4.72143	1.572805	-3.00191	0.002683	0.038315
Iru	1738.358	3.342589	1.113977	3.000591	0.002695	0.038413
CG7381	870.7647	4.509094	1.503387	2.999291	0.002706	0.03848
mahj	170.3569	6.194743	2.065615	2.998983	0.002709	0.03848
lncRNA:CR	823.3874	3.348702	1.119144	2.992199	0.00277	0.039276
caup	165.5269	-7.21652	2.413546	-2.99001	0.00279	0.039489
Alg13	191.867	-7.4715	2.500997	-2.98741	0.002814	0.039756
CG31957	306.1941	-5.2922	1.772332	-2.98601	0.002826	0.039868
CG15296	445.8205	6.697415	2.245151	2.983058	0.002854	0.040184
CG10324	151.4422	-8.43134	2.828328	-2.98103	0.002873	0.040309
mrt	2082.552	2.211863	0.741966	2.981086	0.002872	0.040309
lncRNA:CR	273.6858	-5.60465	1.881505	-2.97881	0.002894	0.040531
CG2006	195.9753	-8.63279	2.899004	-2.97785	0.002903	0.040589
Smu1	1367.777	-3.75653	1.26376	-2.9725	0.002954	0.041087
CG43707	5219.841	1.841053	0.619261	2.972984	0.002949	0.041087
Sec3	386.4934	-7.3563	2.474207	-2.97319	0.002947	0.041087
Rcp	161.5665	-10.5392	3.546384	-2.97182	0.00296	0.041107
CG12003	148.1954	7.659175	2.578375	2.970543	0.002973	0.041208
mRpL51	2197.612	3.08941	1.041075	2.967521	0.003002	0.041543
CG11619	576.4201	4.562028	1.537619	2.966944	0.003008	0.04155
l(2)41Ab	1348.805	-2.6731	0.901751	-2.96434	0.003033	0.04183
CG8301	2203.975	2.203496	0.743542	2.963512	0.003042	0.041872
GluProRS	853.7945	-4.41813	1.49376	-2.95772	0.003099	0.04252
CG15443	578.6738	4.958264	1.676274	2.957907	0.003097	0.04252
kri	2220.823	-2.70816	0.917408	-2.95197	0.003158	0.043247
asRNA:CR4	179.5114	-8.32857	2.823215	-2.95003	0.003177	0.043445
a6	431.2616	-4.51821	1.532178	-2.94888	0.003189	0.043532
Slbp	134.2679	9.373119	3.179813	2.947695	0.003202	0.043552
CG15747	790.2822	3.889128	1.319245	2.947996	0.003198	0.043552
CG17333	162.4258	-9.51362	3.230877	-2.94459	0.003234	0.043916
puf	1049.184	3.592071	1.221167	2.941505	0.003266	0.044275
mthl10	2817.928	-1.98226	0.674121	-2.94051	0.003277	0.044275
CG7326	529.0427	5.029001	1.710034	2.940878	0.003273	0.044275
CG8134	664.4708	4.085597	1.390551	2.938113	0.003302	0.044543
Dhfr	333.323	-7.17612	2.444015	-2.9362	0.003323	0.044743
Hen1	297.4203	-5.57907	1.902049	-2.93319	0.003355	0.045104
CG32095	192.163	-7.26085	2.477407	-2.93083	0.003381	0.045373
Atg4a	409.0695	6.250511	2.133252	2.930039	0.003389	0.045412

nvy	209.3376	6.883474	2.351248	2.927583	0.003416	0.045696
CG6136	334.6365	-6.10499	2.086103	-2.9265	0.003428	0.045778
cnk	1040.83	-3.27176	1.119801	-2.92173	0.003481	0.046255
CG7650	133.6169	-9.67938	3.312632	-2.92196	0.003478	0.046255
dock	2303.497	2.464331	0.84323	2.92249	0.003472	0.046255
Tango6	306.702	6.050854	2.07325	2.918536	0.003517	0.046602
Mfap1	405.6509	6.793838	2.327958	2.918368	0.003519	0.046602
GluRIID	166.8356	-8.9029	3.056623	-2.91266	0.003584	0.047306
Fancd2	556.5936	-4.96731	1.705189	-2.91306	0.003579	0.047306
Mocs1	319.6345	-5.25331	1.808676	-2.9045	0.003678	0.048477
CCDC53	566.1721	-4.16044	1.432932	-2.90345	0.003691	0.048482
Rrp4	189.4188	7.628534	2.627101	2.903784	0.003687	0.048482
CG6685	254.8936	-6.18806	2.133027	-2.90107	0.003719	0.048771
Fer3	171.4533	-9.89462	3.413201	-2.89893	0.003744	0.049012
asRNA:CR4	128.511	-9.31718	3.214482	-2.8985	0.00375	0.049012
Sf3b3	304.9988	-6.46898	2.232349	-2.89784	0.003757	0.049037
CG3919	222.711	-7.85099	2.711525	-2.89541	0.003787	0.049336
CG42806	242.4264	-5.98533	2.071086	-2.88995	0.003853	0.050121
sr	1440.362	2.463844	0.85323	2.887668	0.003881	0.050404
CG12817	1005.012	-3.55558	1.23224	-2.88546	0.003908	0.050455
Spc25	224.3514	6.816733	2.361626	2.886458	0.003896	0.050455
Ir76a	760.3641	4.564817	1.581575	2.886247	0.003899	0.050455
thoc6	107.2361	-11.0941	3.845009	-2.88532	0.00391	0.050455
CG30060	237.3832	8.409392	2.91634	2.883543	0.003932	0.050659
CG5867	1053.993	5.170509	1.794635	2.881092	0.003963	0.050891
Miga	1019.141	4.04607	1.404204	2.881397	0.003959	0.050891
Arc42	1029.605	-3.14927	1.09382	-2.87915	0.003988	0.051124
BCAS2	333.4019	4.984891	1.732216	2.877754	0.004005	0.051269
CR43086	298.2857	7.04338	2.455724	2.868148	0.004129	0.052767
CG11899	341.2765	5.986335	2.088384	2.866491	0.00415	0.05296
CG13409	1237.993	-3.94998	1.378822	-2.86475	0.004173	0.053167
Lhr	143.1335	9.774337	3.41443	2.862656	0.004201	0.053435
ZIPIC	315.3225	6.781727	2.371559	2.859608	0.004242	0.053781
Arpc2	1197.437	-3.94163	1.378244	-2.85989	0.004238	0.053781
Ak3	1070.858	3.209396	1.123406	2.856845	0.004279	0.053996
CG4282	208.6549	7.572222	2.650075	2.857361	0.004272	0.053996
bonsai	548.5875	-4.56433	1.597625	-2.85695	0.004277	0.053996
CG3386	1038.778	-3.38383	1.185445	-2.85448	0.004311	0.054314
CG7461	755.8778	-4.5306	1.588035	-2.85296	0.004331	0.054406
Pkcdelta	602.1091	-4.36181	1.528879	-2.85295	0.004332	0.054406
mt:ND4	65940.37	1.11424	0.390657	2.852219	0.004342	0.054445
Archease	826.5847	4.391931	1.540142	2.85164	0.004349	0.05446
CG17652	310.1083	6.200059	2.174917	2.85071	0.004362	0.054534
Gorab	296.4488	-5.23129	1.836437	-2.84861	0.004391	0.05481
CG31921	168.7302	-8.41463	2.95582	-2.8468	0.004416	0.055037
CG5188	145.2386	-7.87452	2.76916	-2.84365	0.00446	0.055498
CG43231	153.7393	-7.27298	2.561543	-2.8393	0.004521	0.056
asRNA:CR4	158.9915	7.714989	2.716436	2.840115	0.00451	0.056
teq	14286.24	-1.50949	0.531581	-2.83962	0.004517	0.056
CG14182	1284.321	3.559793	1.254381	2.837888	0.004541	0.056161

Nepl16	6791.656	1.941232	0.684467	2.836121	0.004567	0.056386
CG8027	359.0962	7.438512	2.624346	2.834424	0.004591	0.056599
CG3223	487.3376	4.073228	1.438034	2.832497	0.004619	0.056626
geminin	878.2448	-2.90306	1.024977	-2.83232	0.004621	0.056626
CG11710	659.1755	4.589984	1.620019	2.833291	0.004607	0.056626
lncRNA:CR	173.1715	8.423506	2.973612	2.832752	0.004615	0.056626
onecut	10125.62	1.31003	0.462876	2.830198	0.004652	0.056915
CG3740	384.2468	-5.10767	1.805701	-2.82864	0.004675	0.057023
MFS9	2329.926	3.00032	1.060914	2.828051	0.004683	0.057023
CG30403	381.9495	6.12507	2.166138	2.827645	0.004689	0.057023
Cdk8	294.5117	-6.53333	2.31022	-2.82801	0.004684	0.057023
ArfGAP3	1767.776	2.196935	0.777331	2.826253	0.00471	0.057099
lncRNA:CR	214.6175	6.569622	2.324426	2.826341	0.004708	0.057099
sls	563.3432	4.179674	1.479821	2.824447	0.004736	0.057335
CCKLR-17D	609.199	4.931289	1.746316	2.823823	0.004745	0.05736
nerfin-2	139.9929	-9.68463	3.431805	-2.82202	0.004772	0.057596
Vps13	1807.039	-2.17431	0.770622	-2.82151	0.00478	0.057603
MED28	716.8749	3.602928	1.277747	2.819751	0.004806	0.057689
Chrac-14	344.6763	6.823345	2.419422	2.820237	0.004799	0.057689
Plod	1114.121	-3.72004	1.319357	-2.81958	0.004809	0.057689
Uck	3900.445	1.795396	0.636928	2.818836	0.00482	0.057737
CG43861	384.0517	5.018219	1.781076	2.817521	0.00484	0.057888
HtrA2	1058.058	3.936277	1.397914	2.815822	0.004865	0.058108
lncRNA:CR	186.2343	7.504041	2.665606	2.815135	0.004876	0.058146
janA	305.4322	-6.43843	2.28959	-2.81204	0.004923	0.058166
CG8841	733.288	-4.53161	1.610552	-2.8137	0.004897	0.058166
RYBP	1370.764	-4.43925	1.578579	-2.81218	0.004921	0.058166
Taldo	2164.103	-2.45664	0.873722	-2.8117	0.004928	0.058166
naz	296.9732	-5.51785	1.961636	-2.81288	0.00491	0.058166
CG12716	365.2292	6.183745	2.198519	2.812686	0.004913	0.058166
Naa20A	1238.03	-4.05773	1.441844	-2.81427	0.004889	0.058166
CG14434	321.2856	5.194838	1.849822	2.808291	0.004981	0.058612
lncRNA:CR	123.3862	9.261278	3.297781	2.808336	0.00498	0.058612
Fbw5	318.6209	6.915958	2.470034	2.799945	0.005111	0.059972
Kyat	1521.112	3.481576	1.24358	2.79964	0.005116	0.059972
CG32687	207.9352	-8.41883	3.00777	-2.79903	0.005126	0.059972
mri	2549.194	-3.16378	1.130321	-2.79901	0.005126	0.059972
Tace	4143.692	3.025633	1.08174	2.797005	0.005158	0.060258
Plap	421.2174	5.296261	1.894566	2.795501	0.005182	0.060451
Nhe2	4864.39	2.090221	0.748031	2.794299	0.005201	0.060588
lncRNA:CR	195.5539	7.653577	2.743315	2.7899	0.005272	0.061328
Ssu72	836.1773	5.737415	2.057208	2.788932	0.005288	0.061423
Odj	449.5065	-4.01	1.438726	-2.78719	0.005317	0.061574
Regnase-1	248.0933	8.313861	2.983003	2.787078	0.005319	0.061574
lncRNA:CR	203.786	7.750659	2.78127	2.786734	0.005324	0.061574
CG33293	152.602	-7.59272	2.725868	-2.78543	0.005346	0.061699
MICAL-like	1848.413	2.858362	1.026461	2.784678	0.005358	0.061699
CG7600	745.1858	-4.35515	1.563947	-2.78472	0.005357	0.061699
CG4294	3745.538	-1.76303	0.633353	-2.78364	0.005375	0.061808
wol	1413.544	3.367744	1.211283	2.780311	0.005431	0.062356

CG5022	1295.321	-3.59291	1.294346	-2.77585	0.005506	0.062948
CG4622	567.7456	-4.15606	1.497092	-2.77609	0.005502	0.062948
kmr	12552.25	-1.3208	0.475732	-2.77635	0.005497	0.062948
disp	1190.192	3.59178	1.295201	2.773144	0.005552	0.063258
CG11836	121.2299	-8.38893	3.025871	-2.7724	0.005564	0.063258
Marf1	1411.1	2.717357	0.979931	2.773007	0.005554	0.063258
Mip	3659.284	3.150347	1.1362	2.772705	0.005559	0.063258
CG4896	2435.26	-1.95025	0.703778	-2.77112	0.005586	0.063417
nompC	228.9207	6.469343	2.335799	2.769648	0.005612	0.063615
ZnT33D	238.0963	7.766818	2.805349	2.768575	0.00563	0.063735
Cdk5alpha	7896.256	2.059186	0.744014	2.767671	0.005646	0.063763
CCT1	566.8566	-3.7565	1.357353	-2.76752	0.005649	0.063763
CG11898	182.1878	6.131679	2.216614	2.766236	0.005671	0.063903
flr	669.0114	4.706198	1.701518	2.765882	0.005677	0.063903
hb	137.6619	8.401518	3.041004	2.762745	0.005732	0.06443
ush	248.3504	6.330136	2.294997	2.758233	0.005811	0.065235
FoxP	9681.467	1.464058	0.531554	2.754297	0.005882	0.065932
Dcr-2	837.441	-3.75589	1.364583	-2.75241	0.005916	0.066221
cerv	377.3708	-5.74989	2.090497	-2.75049	0.005951	0.066518
Alr	369.2748	-5.81996	2.117119	-2.749	0.005978	0.066728
SrpRbeta	5297.872	-2.05935	0.749545	-2.74746	0.006006	0.066948
Rnp4F	504.7707	-4.69297	1.71035	-2.74386	0.006072	0.067499
CG7322	1264.359	-2.47462	0.901739	-2.74428	0.006064	0.067499
CG15317	266.545	-7.7003	2.807659	-2.7426	0.006095	0.067572
Elp3	285.6166	-8.29952	3.025891	-2.74283	0.006091	0.067572
CG7728	91.67118	-10.54	3.844551	-2.74155	0.006115	0.067695
CG18547	120.723	9.521299	3.475221	2.739768	0.006148	0.06797
CG2698	4019.654	2.183482	0.797354	2.738409	0.006174	0.068157
asRNA:CR4	190.0945	6.047265	2.208844	2.737751	0.006186	0.0682
TyrR	373.1447	-5.43559	1.988818	-2.73307	0.006275	0.069081
dnd	194.9955	6.880055	2.520515	2.729622	0.006341	0.069522
ClC-b	1839.227	2.561073	0.938246	2.729639	0.00634	0.069522
Gasz	1452.514	2.318114	0.849186	2.729809	0.006337	0.069522
Ate1	1229.926	-3.09136	1.133004	-2.72846	0.006363	0.069577
CG41520	143.581	-9.14795	3.352404	-2.72877	0.006357	0.069577
Oct-TyrR	886.2351	3.085967	1.131698	2.726847	0.006394	0.069784
Rep	1132.465	-3.17344	1.163886	-2.72659	0.006399	0.069784
Sas10	389.7616	5.952047	2.183823	2.725518	0.00642	0.069915
CG11686	855.127	-4.58003	1.680905	-2.72474	0.006435	0.069985
Mfe2	846.1458	3.431208	1.259774	2.72367	0.006456	0.070117
Naa60	3148.017	1.717837	0.630976	2.722506	0.006479	0.07027
CG3842	406.8108	-5.56164	2.043258	-2.72195	0.00649	0.070294
Fer1	913.3424	-3.69044	1.358822	-2.71591	0.006609	0.071492
CG12868	1099.437	-3.11565	1.147897	-2.71422	0.006643	0.07176
CG9485	92.76707	-8.16752	3.01052	-2.71299	0.006668	0.071833
lawc	5093.352	1.512278	0.557404	2.713074	0.006666	0.071833
Eip63F-1	2859.852	2.441385	0.900463	2.711254	0.006703	0.072115
Hmt-1	2174.013	-2.99827	1.107654	-2.70686	0.006792	0.07277
CG17841	8056.388	-1.67895	0.620343	-2.70648	0.0068	0.07277
Rhp	950.2928	3.373105	1.245815	2.70755	0.006778	0.07277

Use1	120.482	9.481931	3.502764	2.706986	0.00679	0.07277
CG6903	2210.825	-2.1168	0.783523	-2.70164	0.0069	0.07358
ATP8A	5869.129	1.451224	0.537197	2.701476	0.006903	0.07358
CG5535	655.9094	-4.39312	1.626082	-2.70166	0.006899	0.07358
ebd2	609.2361	-4.81654	1.783324	-2.70088	0.006916	0.073614
CG33096	169.3463	-6.99857	2.593165	-2.69885	0.006958	0.073857
koi	2400.069	2.46713	0.914004	2.699254	0.00695	0.073857
CG9951	472.1606	5.499629	2.038062	2.698461	0.006966	0.073857
CG7110	208.0684	6.511041	2.414959	2.696129	0.007015	0.074278
Caf1-105	638.3333	5.1296	1.903025	2.695498	0.007028	0.074321
Lrch	1916.72	2.884784	1.070426	2.694986	0.007039	0.074337
nopo	144.7412	-9.82702	3.647312	-2.69432	0.007053	0.074388
Gfat2	931.2668	-3.41709	1.268725	-2.69333	0.007074	0.074511
CG3645	700.2804	-3.74205	1.390523	-2.69111	0.007121	0.074714
CG5335	542.0544	-5.87906	2.184265	-2.69155	0.007112	0.074714
CG2889	1588.458	2.73283	1.015482	2.691165	0.00712	0.074714
lncRNA:CR	169.9043	7.290064	2.709598	2.69046	0.007135	0.074763
pkaap	1399.246	2.308616	0.860364	2.683301	0.00729	0.076058
htt	1357.774	-3.40694	1.269489	-2.68371	0.007281	0.076058
CG8389	1152.404	-4.74436	1.768318	-2.68298	0.007297	0.076058
CG15412	361.8056	6.589387	2.455323	2.683715	0.007281	0.076058
NC2alpha	266.8113	-6.61556	2.467345	-2.68125	0.007335	0.076256
lbk	1977.756	-2.03935	0.760498	-2.68161	0.007327	0.076256
CG10431	179.9195	-8.02439	2.993999	-2.68016	0.007359	0.076405
Nep4	5067.539	-1.57998	0.590395	-2.67615	0.007447	0.077226
CG15343	156.2043	-8.37741	3.131826	-2.67493	0.007474	0.077407
dgt2	144.4108	9.743285	3.64486	2.673158	0.007514	0.077617
Nph	752.0837	-3.45232	1.291419	-2.67327	0.007511	0.077617
CG3505	229.7952	7.117419	2.666137	2.669563	0.007595	0.078352
pk	171.8179	-7.90012	2.962394	-2.6668	0.007658	0.078896
SCCRO	1799.13	-2.2745	0.853371	-2.66532	0.007692	0.079144
lncRNA:CR	300.9669	-6.14813	2.309583	-2.66201	0.007768	0.079824
kat80	366.7111	4.549341	1.709405	2.661359	0.007783	0.079876
Atac2	356.4966	6.934933	2.606774	2.660351	0.007806	0.080013
CG9314	92.43829	-9.67628	3.639069	-2.659	0.007837	0.080233
CG31875	457.3288	6.529753	2.460202	2.654153	0.007951	0.080981
Nse4	563.2484	-4.54551	1.711879	-2.65527	0.007924	0.080981
CG11103	1910.278	2.402606	0.90508	2.654578	0.007941	0.080981
CG7692	589.2173	4.364879	1.644124	2.654836	0.007935	0.080981
alphaCOP	1616.733	-2.26247	0.853205	-2.65173	0.008008	0.081462
CG11656	108.32	-9.34513	3.525179	-2.65097	0.008026	0.081542
snoRNA:M	213.5648	-7.19975	2.71925	-2.6477	0.008104	0.08223
CG9286	439.9763	-5.20594	1.968417	-2.64473	0.008176	0.082849
Mst33A	127.2006	7.724825	2.922508	2.643217	0.008212	0.083116
MED27	731.8493	4.004002	1.51511	2.642714	0.008224	0.083135
Klp64D	2210.577	2.037925	0.77132	2.642126	0.008239	0.083175
atk	274.0617	-7.00388	2.652252	-2.64073	0.008273	0.083414
CG4813	124.4774	9.614789	3.646828	2.63648	0.008377	0.08436
CG18528	187.208	7.723485	2.930563	2.635495	0.008401	0.084393
CG32335	137.9682	-9.78053	3.710522	-2.63589	0.008392	0.084393

asRNA:CR4	191.9417	-7.46051	2.831252	-2.63506	0.008412	0.084396
CG13126	189.8393	-5.80331	2.205042	-2.63184	0.008492	0.085094
Wdr24	155.6991	-7.23691	2.753955	-2.62782	0.008593	0.085997
Dr	298.6096	-6.56969	2.501428	-2.62638	0.00863	0.086257
Kul	3876.102	1.692052	0.644665	2.624698	0.008673	0.086575
CG4957	942.7696	3.934087	1.500838	2.621261	0.008761	0.087329
lncRNA:CR	141446.4	1.987161	0.7582	2.620895	0.00877	0.087329
CG30467	370.5213	-4.68726	1.789729	-2.61898	0.008819	0.087604
calypso	499.9495	4.475093	1.708643	2.619091	0.008816	0.087604
Prx6005	660.7555	-4.18639	1.600616	-2.61549	0.00891	0.088396
lncRNA:CR	114.3273	-8.04679	3.079368	-2.61313	0.008972	0.088788
CG43783	904.7741	3.243639	1.241117	2.613483	0.008962	0.088788
CG2861	158.8498	-8.08567	3.095161	-2.61236	0.008992	0.088879
CG42354	154.0031	-6.42675	2.462083	-2.61029	0.009047	0.089309
rump	1125.541	2.393972	0.918229	2.607162	0.00913	0.090018
CG12877	693.336	3.912282	1.504661	2.600108	0.009319	0.091776
CG4278	302.7341	4.652477	1.790498	2.598427	0.009365	0.092114
didum	2053.645	2.584544	0.995793	2.595464	0.009446	0.092798
acj6	316.4632	-4.79472	1.848082	-2.59443	0.009475	0.092965
CG30148	133.7965	-9.67521	3.732203	-2.59236	0.009532	0.093152
CG8490	391.8206	-4.34442	1.675578	-2.59279	0.00952	0.093152
Hip1	571.4306	-4.75902	1.835176	-2.59322	0.009508	0.093152
CG8549	826.0761	-3.74326	1.444128	-2.59206	0.00954	0.093152
CG8485	1304.715	-2.72333	1.051412	-2.59017	0.009593	0.093551
CG11617	170.3903	-6.57674	2.543886	-2.58531	0.009729	0.094764
CG15211	818.8386	-3.13077	1.212832	-2.58137	0.009841	0.095736
CG16978	768.3719	4.942168	1.916159	2.579205	0.009903	0.096106
lncRNA:CR	335.2464	6.305685	2.444594	2.57944	0.009896	0.096106
CG15522	284.9283	5.299251	2.057541	2.575526	0.010009	0.097017
CG9154	488.8327	-5.61008	2.181106	-2.57212	0.010108	0.09774
lncRNA:CR	216.8461	7.120063	2.768075	2.572207	0.010105	0.09774
sll	558.3221	3.910259	1.520714	2.571332	0.010131	0.097846
CG34126	2524.589	-1.84235	0.717289	-2.56848	0.010214	0.098535
Art7	244.0741	-6.78229	2.646764	-2.56248	0.010393	0.100134
CG15651	1503.816	-3.40602	1.330986	-2.55902	0.010497	0.101016
CG32473	3123.402	-1.80668	0.706577	-2.55694	0.01056	0.101256
CG13605	626.9189	4.171893	1.631359	2.557311	0.010548	0.101256
GXIVsPLA2	1344.602	3.443096	1.346332	2.557391	0.010546	0.101256
CG4936	343.1679	-5.00305	1.960115	-2.55242	0.010698	0.102457
CR43087	192.5759	5.829759	2.287582	2.548437	0.010821	0.103512
CG5348	602.3145	4.484237	1.761944	2.545051	0.010926	0.104149
Vmat	46892.74	-1.12892	0.443507	-2.54544	0.010914	0.104149
Rint1	916.7615	4.017065	1.578233	2.545293	0.010919	0.104149
Glos	262.7868	6.307239	2.480916	2.542303	0.011012	0.104847
CG3149	1115.484	-3.94652	1.553025	-2.54118	0.011048	0.105059
lace	2171.316	2.022117	0.796426	2.538988	0.011117	0.105531
CG5721	303.1778	-5.58584	2.200367	-2.5386	0.01113	0.105531
lncRNA:CR	144.2179	8.155998	3.213081	2.538373	0.011137	0.105531
Obp44a	102158.4	-1.1526	0.454347	-2.53683	0.011186	0.105873
CG12375	1815.905	-3.4134	1.3458	-2.53633	0.011202	0.105897

GstE8	403.9929	-6.41212	2.530209	-2.53423	0.01127	0.10624
Nup98-96	323.0065	4.55253	1.796018	2.534791	0.011251	0.10624
CG42346	3096.181	2.124174	0.838279	2.533969	0.011278	0.10624
Pex13	1152.871	-2.45349	0.968647	-2.53291	0.011312	0.106437
Cyp6t1	1097.167	3.50813	1.385567	2.531909	0.011344	0.106616
CG10731	359.0777	5.43658	2.147625	2.531438	0.01136	0.106634
Spg7	1079.773	3.39865	1.344133	2.528508	0.011455	0.107403
lncRNA:CR	153.6518	-8.29152	3.284419	-2.5245	0.011586	0.108508
deltaCOP	1435.02	2.263154	0.896981	2.52308	0.011633	0.108821
Tak1	816.6313	-3.72735	1.477887	-2.52208	0.011666	0.108876
Vps29	1377.821	2.410049	0.955557	2.52214	0.011664	0.108876
SrpRalpha	858.2759	-3.44519	1.366735	-2.52075	0.011711	0.109163
CG34354	2680.216	-1.80744	0.717144	-2.52032	0.011725	0.109167
CG11906	775.0646	3.378429	1.341336	2.518704	0.011779	0.109505
CheB74a	159.1881	-7.26945	2.886513	-2.51842	0.011788	0.109505
CG6834	1866.975	-2.37717	0.94432	-2.51734	0.011825	0.109716
dgt6	441.5481	6.967658	2.76878	2.516508	0.011852	0.109847
sgll	2009.447	2.523735	1.004049	2.513557	0.011952	0.110643
CG2846	793.938	-3.8732	1.541716	-2.51227	0.011996	0.110874
Vps36	450.5631	-5.56995	2.217383	-2.51195	0.012007	0.110874
Sec8	497.6353	-3.89612	1.551248	-2.5116	0.012018	0.110874
ZnT77C	423.4169	-4.39462	1.750662	-2.51026	0.012064	0.111167
CG42797	1152.239	3.106843	1.238169	2.509224	0.0121	0.111367
Ccdc85	148.1112	8.866447	3.539812	2.504779	0.012253	0.112647
CG14712	1257.243	-3.50089	1.400067	-2.50052	0.012401	0.113363
Nepl17	758.5318	4.592304	1.836455	2.500634	0.012397	0.113363
lncRNA:CR	75.99087	-9.6159	3.844241	-2.50138	0.012371	0.113363
Lkr	903.8137	4.15584	1.661877	2.50069	0.012395	0.113363
insv	83.21657	9.614721	3.844211	2.501091	0.012381	0.113363
Pa1	1738.868	3.050449	1.22108	2.498157	0.012484	0.11399
CG7168	118.135	-8.79899	3.522877	-2.49767	0.012501	0.114016
pyr	123.0127	-8.60232	3.448279	-2.49467	0.012607	0.114855
CG4230	7765.958	2.019711	0.810514	2.491889	0.012707	0.115627
MESR4	4174.953	-1.62831	0.653571	-2.49141	0.012724	0.115641
CG34396	146.3505	6.395174	2.567269	2.491042	0.012737	0.115641
wek	336.3106	-3.96231	1.593915	-2.4859	0.012922	0.11706
CG31323	779.6546	-3.07103	1.235182	-2.4863	0.012908	0.11706
CG3356	1262.201	2.747659	1.106576	2.483027	0.013027	0.117876
CG31612	91.03427	9.43331	3.800644	2.48203	0.013064	0.118073
CG2662	375.5819	4.887669	1.971976	2.478564	0.013191	0.119092
CG5973	677.0084	-3.65686	1.477222	-2.4755	0.013305	0.119984
Lectin-gal(	102.629	-9.65529	3.905256	-2.47238	0.013422	0.120899
CG9839	875.7462	2.520811	1.019809	2.471846	0.013442	0.120946
CG17068	222.0788	-5.96187	2.413679	-2.47004	0.01351	0.121423
Vps25	1856.523	1.888768	0.764805	2.469608	0.013526	0.121432
Atet	3467.676	1.79726	0.72799	2.468797	0.013557	0.121572
CG8132	152.1612	7.778855	3.152425	2.467578	0.013603	0.121851
Teh4	1293.55	3.781414	1.533492	2.465884	0.013668	0.122292
hpo	621.9195	3.611933	1.465391	2.464825	0.013708	0.122409
CG3831	471.7593	5.047277	2.04779	2.464743	0.013711	0.122409

CG9664	1307.466	-3.22268	1.307988	-2.46385	0.013745	0.122579
CG13284	236.0507	-7.92849	3.220403	-2.46196	0.013818	0.12309
lectin-37D.	94.2488	-9.61637	3.907623	-2.46093	0.013858	0.123308
GABA-B-R:	5039.632	1.329835	0.540497	2.460396	0.013878	0.123353
CG2199	459.4961	4.119637	1.675919	2.458136	0.013966	0.123995
Ptr	2863.427	-1.62143	0.66013	-2.45623	0.01404	0.124518
CG4133	406.4583	3.880542	1.581874	2.453129	0.014162	0.125457
cry	1694.471	-2.91271	1.188264	-2.45123	0.014237	0.125842
msd5	604.1853	4.554141	1.857853	2.451292	0.014234	0.125842
CG13641	145.8122	-8.1817	3.343345	-2.44716	0.014399	0.127133
RhoGAP15	341.7353	-4.48878	1.835045	-2.44614	0.01444	0.127213
rept	180.1645	6.481443	2.649486	2.446302	0.014433	0.127213
Nep2	874.457	3.549801	1.451942	2.444865	0.014491	0.127479
mld	2778.85	-1.62989	0.666732	-2.4446	0.014501	0.127479
CG1575	271.1246	-6.29201	2.575707	-2.44283	0.014573	0.127965
Dcr-1	157.1839	5.916817	2.425001	2.439923	0.01469	0.12864
oys	3934.841	-1.77069	0.725624	-2.44023	0.014678	0.12864
Alh	2939.405	-1.74438	0.7151	-2.43935	0.014714	0.12864
CG8209	461.013	-4.17634	1.71188	-2.43962	0.014703	0.12864
CG32147	618.4242	-3.92536	1.609859	-2.43833	0.014755	0.128865
Uba1	2787.384	-2.3031	0.945605	-2.43558	0.014868	0.129707
Pbp95	341.825	6.611232	2.715837	2.434326	0.01492	0.130016
Patsas	899.3449	-2.6101	1.072657	-2.4333	0.014962	0.130242
CG5390	584.6631	-4.20635	1.729537	-2.43207	0.015013	0.130545
egr	1191.767	-2.48694	1.025141	-2.42595	0.015269	0.132565
Fitm	383.3514	-4.41051	1.818228	-2.42572	0.015278	0.132565
lncRNA:CR	81.43331	-9.46333	3.907749	-2.42168	0.015449	0.133901
TwdlG	1325.817	-2.81545	1.164374	-2.418	0.015606	0.135119
Stam	699.4173	2.802851	1.159838	2.416589	0.015667	0.135497
trbd	1463.228	-3.30138	1.367707	-2.4138	0.015787	0.13639
mt:ND4L	2113.996	1.771671	0.734235	2.412948	0.015824	0.136513
CG32591	68.50143	-9.42801	3.907673	-2.41269	0.015835	0.136513
CG12379	92.22378	-8.83953	3.664989	-2.41188	0.01587	0.136668
lncRNA:CR	841.2904	3.546801	1.470985	2.411174	0.015901	0.136788
L2HGDH	1038.289	2.733135	1.133954	2.410269	0.015941	0.136981
Fkbp59	2166.675	-2.85251	1.183809	-2.40961	0.01597	0.137083
CG43347	4578.766	1.417856	0.588641	2.408693	0.01601	0.13728
Es2	241.6902	-5.37239	2.234152	-2.40467	0.016187	0.138505
Pop2	4302.467	1.504379	0.625558	2.404859	0.016179	0.138505
CG13663	1276.561	-3.45557	1.438546	-2.40213	0.0163	0.139175
eas	2981.266	-1.73189	0.720899	-2.40241	0.016287	0.139175
mRF1	422.1329	5.279972	2.198602	2.401513	0.016327	0.139221
BORCS6	378.519	4.74101	1.97441	2.401228	0.01634	0.139221
lncRNA:CR	105.5176	-7.6655	3.194117	-2.39988	0.0164	0.139586
Tsp26A	1808.21	2.650644	1.105088	2.398581	0.016459	0.139638
ea	706.8779	-3.94402	1.643803	-2.39933	0.016425	0.139638
CG7546	1732.132	-1.89438	0.789743	-2.39873	0.016452	0.139638
elg1	75.70688	8.704242	3.631228	2.397052	0.016528	0.140074
lncRNA:CR	309.9374	6.503714	2.71468	2.395757	0.016586	0.140422
CG5611	174.0861	-6.44903	2.695543	-2.39248	0.016735	0.141384

lncRNA:CR	129.9206	8.29753	3.46807	2.39255	0.016732	0.141384
Bap55	1169.551	-2.18943	0.915421	-2.39172	0.01677	0.141528
CG9109	176.1035	6.686191	2.79649	2.390922	0.016806	0.141687
Rpl12	173.6397	5.993842	2.510299	2.387701	0.016954	0.142785
Sik2	1541.669	-3.1336	1.314035	-2.38472	0.017092	0.143798
Cyp6a22	641.0827	-3.35958	1.409554	-2.38344	0.017152	0.144148
CG11407	12694.09	-1.31438	0.553191	-2.376	0.017502	0.146521
CG6145	1026.99	2.275314	0.957674	2.375876	0.017507	0.146521
lncRNA:CR	164.7585	-8.22063	3.459887	-2.37598	0.017502	0.146521
CG12992	219.2428	5.684737	2.392524	2.376042	0.017499	0.146521
smo	117.5145	8.081426	3.408184	2.371182	0.017731	0.147933
nwk	7243.603	1.212543	0.511338	2.371312	0.017725	0.147933
Syx16	416.22	-3.94091	1.661689	-2.37163	0.01771	0.147933
CG14450	986.6928	-2.95383	1.246145	-2.37037	0.01777	0.148021
Sgsh	1638.439	-3.15921	1.332892	-2.37019	0.017779	0.148021
CG14561	177.7163	7.982514	3.368934	2.369448	0.017815	0.148166
lncRNA:CR	95.35342	9.250206	3.908098	2.366933	0.017936	0.149022
AdSL	323.6056	-5.47646	2.315089	-2.36555	0.018003	0.149426
Sf3a1	203.1351	4.39143	1.856853	2.364985	0.018031	0.149499
CG11755	104.0804	-7.55492	3.195825	-2.364	0.018079	0.149741
Spn55B	116.9008	-8.17721	3.459609	-2.36362	0.018097	0.149741
CG2921	1543.01	-2.18045	0.922737	-2.36303	0.018126	0.149828
Pmm2	500.6458	-3.05144	1.291547	-2.36262	0.018146	0.149836
Cyp6a13	3515.743	-1.50121	0.635563	-2.36201	0.018176	0.149929
TfIIB	2254.315	2.109657	0.893555	2.360969	0.018227	0.150197
Rpb11	671.4262	-3.22433	1.366842	-2.35897	0.018326	0.150855
CG5916	1104.716	3.329097	1.411734	2.358161	0.018366	0.151028
CG8230	3013.541	2.274999	0.96497	2.357586	0.018394	0.151107
Rgk3	2192.08	-1.8605	0.78958	-2.35632	0.018457	0.151467
CG1492	321.3623	5.884543	2.498096	2.355611	0.018492	0.151603
CG44085	93.3906	7.232464	3.071906	2.354389	0.018553	0.151947
CG8569	610.7515	4.306007	1.831242	2.351414	0.018702	0.153011
vtd	7050.009	1.238424	0.527564	2.347438	0.018903	0.154497
yata	1402.589	-2.16019	0.920526	-2.34669	0.018941	0.154648
Vps37B	663.3752	3.977523	1.695936	2.345326	0.01901	0.155059
Scp1	242.6894	-5.90425	2.518371	-2.34447	0.019054	0.155105
bou	570.626	3.580563	1.527246	2.344457	0.019055	0.155105
Ugt35A1	789.5732	-3.68262	1.571696	-2.34308	0.019125	0.155362
lncRNA:CR	77.25779	9.156098	3.907561	2.343174	0.01912	0.155362
Ugt35C1	3445.945	1.596313	0.681514	2.342306	0.019165	0.155529
asRNA:CR4	1262.29	-3.49394	1.492283	-2.34134	0.019215	0.155774
CG3368	963.4852	-3.40534	1.454992	-2.34046	0.01926	0.155986
CG1236	412.7891	-4.98633	2.132062	-2.33874	0.019349	0.156548
HemK1	3053.93	-1.47726	0.632041	-2.33729	0.019424	0.156996
Adgf-D	3259.049	-1.60497	0.686966	-2.33631	0.019475	0.157249
lncRNA:CR	251.4618	-6.08774	2.60752	-2.33469	0.01956	0.157776
CG31125	500.4991	3.876234	1.661947	2.332344	0.019683	0.158607
Nup44A	474.221	-3.99704	1.715451	-2.33002	0.019805	0.159273
Gbs-70E	192.4575	-5.09633	2.186924	-2.33037	0.019787	0.159273
Hat1	830.825	-3.34964	1.439078	-2.32763	0.019932	0.159694

CG3430	1082.061	-3.10055	1.331527	-2.32857	0.019882	0.159694
ppan	151.2999	-6.73352	2.892984	-2.32753	0.019937	0.159694
CG8237	521.6492	4.131274	1.774671	2.32791	0.019917	0.159694
OdsH	96.58521	9.083209	3.903309	2.327053	0.019962	0.159739
DnaJ-H	4848.167	1.735996	0.746356	2.325964	0.02002	0.159885
Wnt4	614.4947	3.872985	1.665001	2.326116	0.020012	0.159885
Orc4	313.029	-6.09476	2.628698	-2.31855	0.02042	0.16291
Task6	2805.442	1.615557	0.698216	2.313834	0.020677	0.164553
U4-U6-60k	136.2326	-7.81623	3.377703	-2.31407	0.020664	0.164553
Agpat3	4132.993	2.114219	0.913803	2.313649	0.020687	0.164553
CG31648	1524.647	2.948734	1.276266	2.310438	0.020864	0.165796
CG13796	244.7284	6.122127	2.650365	2.309918	0.020893	0.16586
Fatp1	3355.519	1.50382	0.651448	2.308427	0.020975	0.166352
Cyp4s3	297.2046	-4.81472	2.088024	-2.30588	0.021118	0.167314
mei-W68	98.58035	-8.96446	3.889801	-2.30461	0.021189	0.167712
Atg18b	2694.032	-1.57755	0.685081	-2.30273	0.021294	0.168382
Ctl1	1937.828	2.540598	1.103842	2.301597	0.021358	0.168719
CG7745	997.6966	2.92938	1.273331	2.300564	0.021416	0.169014
Obp57b	161.8818	8.158455	3.546959	2.300127	0.021441	0.169043
Sep-01	1611.9	-3.43784	1.49542	-2.29892	0.02151	0.169418
Gnpnat	448.705	4.052035	1.765021	2.295743	0.021691	0.170675
NELF-B	669.892	-3.13863	1.367593	-2.295	0.021733	0.170843
CG15233	262.5973	7.104894	3.09654	2.294462	0.021764	0.170918
Usp30	2846.133	-1.75035	0.763463	-2.29264	0.021869	0.171404
Prip	623.0634	5.55882	2.424518	2.292753	0.021862	0.171404
CG9684	191.3764	-5.42386	2.368799	-2.28971	0.022038	0.172564
CG11777	172.4942	-7.18477	3.138517	-2.28922	0.022066	0.172616
Krn	2152.794	-3.42936	1.498338	-2.28877	0.022092	0.172652
bocks	839.427	-3.03823	1.328785	-2.28647	0.022227	0.173532
Tim17b1	364.8492	4.403774	1.927407	2.284817	0.022324	0.173843
ouib	301.6094	5.616679	2.458212	2.284864	0.022321	0.173843
dunk	90.44686	8.92958	3.908454	2.284684	0.022331	0.173843
CG17597	186.4513	-6.79771	2.976413	-2.28386	0.02238	0.174051
SCCRO4	1886.412	-1.96453	0.860638	-2.28264	0.022451	0.174438
CG3756	407.1122	-3.70843	1.625547	-2.28134	0.022528	0.174697
Jarid2	4113.363	-1.43522	0.629044	-2.28158	0.022514	0.174697
dpr2	1574.85	2.186625	0.959132	2.279797	0.02262	0.175237
CG30105	90.62655	-8.81139	3.866668	-2.27881	0.022679	0.175355
lncRNA:CR	181.9488	5.254548	2.30575	2.278889	0.022674	0.175355
CG10747	349.5569	4.214348	1.84968	2.27842	0.022702	0.175364
lncRNA:CR	76.06034	-8.90418	3.909009	-2.27786	0.022735	0.175452
CG3061	2387.194	-2.47207	1.087027	-2.27415	0.022957	0.176655
CG6006	3714.915	-1.68404	0.740513	-2.27416	0.022956	0.176655
cag	492.3129	-4.48653	1.972296	-2.27477	0.022919	0.176655
CG6959	1628.584	2.702251	1.188949	2.272806	0.023038	0.17677
CG6654	722.3553	3.552969	1.562892	2.273331	0.023006	0.17677
CG3045	114.6378	-6.96571	3.064436	-2.27308	0.023021	0.17677
CG3335	391.5061	-4.48033	1.972115	-2.27184	0.023096	0.177048
Arl6IP1	3206.035	-1.69926	0.748619	-2.26986	0.023216	0.17763
Xpd	289.5167	4.921898	2.168366	2.269865	0.023216	0.17763

goe	3092.98	-1.63176	0.719216	-2.26881	0.02328	0.177779
CG8108	3666.338	-1.39711	0.615785	-2.26882	0.023279	0.177779
Mys45A	169.3572	-6.51632	2.875328	-2.26629	0.023434	0.178613
CG3163	599.8545	3.592113	1.584883	2.266484	0.023422	0.178613
CG32236	125.0404	6.052232	2.671757	2.265263	0.023497	0.178752
CG6761	160.0972	-8.04656	3.551857	-2.26545	0.023485	0.178752
CG8520	172.6011	4.761085	2.104732	2.262086	0.023692	0.180069
Ddx1	72.83565	8.829581	3.908548	2.259044	0.023881	0.180988
Tsp42Ek	556.7387	3.171388	1.403473	2.259672	0.023842	0.180988
Muc96D	94.20904	8.581291	3.798395	2.259189	0.023872	0.180988
muskelin	260.6175	4.066176	1.80169	2.256867	0.024016	0.181033
dpr4	833.4687	-2.57718	1.1417	-2.25731	0.023988	0.181033
su(r)	641.6428	-2.83286	1.254907	-2.25742	0.023982	0.181033
magu	873.8997	2.602194	1.153057	2.256779	0.024022	0.181033
CG34377	717.9036	3.646164	1.614645	2.258183	0.023934	0.181033
CG32655	95.59628	-6.79193	3.009195	-2.25706	0.024004	0.181033
metl	1479.531	-2.68994	1.192196	-2.25629	0.024053	0.181094
bru1	970.5266	-2.33185	1.034273	-2.25458	0.02416	0.18173
CG4078	495.8875	-4.22007	1.872166	-2.25411	0.024189	0.181782
CG30108	72.31162	8.805623	3.908578	2.252897	0.024266	0.182185
CG13293	1125.94	2.411319	1.070607	2.252291	0.024304	0.182302
CG9360	252.9369	3.985997	1.770633	2.25117	0.024375	0.182663
mi	133.5379	-8.39701	3.730968	-2.25062	0.024409	0.182751
CycA	673.3977	-4.15369	1.846572	-2.2494	0.024487	0.183161
Not11	928.783	-3.27375	1.45639	-2.24785	0.024585	0.183727
dve	362.2043	-5.98479	2.664259	-2.24632	0.024683	0.184115
Coq5	518.1083	4.506086	2.005912	2.246402	0.024678	0.184115
IntS14	497.4355	4.24637	1.893583	2.242505	0.024929	0.185774
AnxB11	348.5143	-4.97672	2.221542	-2.24021	0.025077	0.186706
Cyp6d4	424.1692	5.88339	2.629338	2.237593	0.025248	0.187801
Irp-1A	669.1596	3.327497	1.488327	2.23573	0.025369	0.188533
CRAT	641.6437	3.030592	1.356936	2.233409	0.025522	0.189491
Ilk	1125.238	2.694077	1.206891	2.232245	0.025599	0.189885
Hr38	8674.528	1.49946	0.672027	2.231248	0.025665	0.190199
CG4858	2313.593	-3.21591	1.442394	-2.22957	0.025776	0.190673
Fmo-2	162.7355	-5.60379	2.513235	-2.22971	0.025766	0.190673
az2	557.2212	-3.8992	1.749401	-2.22888	0.025822	0.190836
Rexo5	2284.079	-1.85182	0.83127	-2.2277	0.025901	0.19124
Arp6	129.2275	6.461796	2.905756	2.223792	0.026162	0.192996
Cog1	1003.92	3.331914	1.499374	2.222203	0.02627	0.193522
pll	67.57122	8.682076	3.907289	2.222021	0.026282	0.193522
Hs3st-A	6238.857	1.20447	0.542408	2.220598	0.026378	0.194053
CG6724	639.4748	-4.644	2.094537	-2.2172	0.02661	0.195243
comt	11570.28	0.990628	0.446675	2.21778	0.02657	0.195243
comm	966.2716	-2.23977	1.010205	-2.21715	0.026613	0.195243
Nrk	438.5134	4.025555	1.816021	2.21669	0.026644	0.195294
Mettl3	305.9721	6.599848	2.978732	2.215656	0.026715	0.195456
CG13531	113.806	6.776839	3.058139	2.216001	0.026691	0.195456
CG7987	1589.511	2.159252	0.975543	2.213384	0.026871	0.196419
TTLL4A	4575.528	1.256983	0.568034	2.212867	0.026907	0.196496

ddbt	85.5661	7.519242	3.398495	2.212521	0.026931	0.196496
MFS3	177.7059	7.547372	3.412358	2.211776	0.026982	0.196693
Fs	428.3627	5.392948	2.442422	2.208032	0.027242	0.198407
hbs	134.4268	-5.4637	2.475226	-2.20736	0.027289	0.198571
beat-IIIb	3547.364	1.371853	0.621891	2.205939	0.027388	0.199111
CG15083	1516.16	-2.12085	0.961874	-2.20491	0.02746	0.199455
lncRNA:CR	62.99831	8.614641	3.908833	2.203891	0.027532	0.199794
ADPS	1074.743	3.90416	1.772104	2.203121	0.027586	0.200007
pdgy	8034.116	-1.42279	0.645933	-2.20269	0.027617	0.200047
CG14445	160.5348	-6.8332	3.107129	-2.1992	0.027864	0.201521
subdued	2763.808	2.159889	0.982483	2.198399	0.027921	0.201521
eIF3i	4522.689	-1.51623	0.689608	-2.19869	0.0279	0.201521
CG6175	377.5301	-3.6274	1.649795	-2.1987	0.027899	0.201521
CG13123	92.26905	7.471618	3.402195	2.196117	0.028084	0.202505
CG2767	189.8726	-5.49712	2.503489	-2.19578	0.028107	0.202505
Apc	3846.628	1.4109	0.64279	2.194963	0.028166	0.202746
Mkk4	4046.403	1.483089	0.676041	2.193785	0.028251	0.203173
Arp5	776.4538	-3.21186	1.465594	-2.19151	0.028415	0.203443
CG11771	789.0679	-3.78275	1.725151	-2.1927	0.028329	0.203443
gwl	363.4277	-4.66799	2.130015	-2.19153	0.028413	0.203443
CG42554	476.8771	3.532079	1.61151	2.191783	0.028395	0.203443
CG14229	2056.148	1.797802	0.820211	2.191879	0.028388	0.203443
SMSr	1285.643	-2.19867	1.003927	-2.19007	0.028519	0.204007
CG7791	2091.21	1.796623	0.820752	2.188996	0.028597	0.204382
Nach	89.08091	8.524585	3.895767	2.188166	0.028658	0.204631
ebo	722.832	-3.27896	1.499209	-2.18713	0.028733	0.204988
CG11438	281.1789	6.176511	2.82495	2.186414	0.028785	0.205179
CG31694	1531.125	2.170299	0.993347	2.184836	0.028901	0.205819
CG9784	1061.758	-2.4323	1.114607	-2.1822	0.029095	0.207015
CG31937	192.944	-5.56597	2.552066	-2.18096	0.029186	0.207482
CG5013	75.18197	-7.81035	3.583116	-2.17976	0.029275	0.207746
Rpb5	621.0788	3.40564	1.562317	2.179865	0.029267	0.207746
Naam	63.18637	8.391702	3.853135	2.177889	0.029414	0.20855
Dl	125.9047	-6.54286	3.006586	-2.17618	0.029542	0.209271
CG44008	169.0836	5.431971	2.498029	2.174503	0.029667	0.209974
CG2701	696.7191	2.870954	1.320746	2.173736	0.029725	0.210166
CadN2	1087.193	2.893043	1.331157	2.17333	0.029755	0.210166
CG9948	548.9503	3.592292	1.653075	2.173097	0.029773	0.210166
Dcp-1	253.259	-5.02236	2.31202	-2.17228	0.029834	0.210413
CG11902	531.6454	3.45632	1.591844	2.171267	0.029911	0.210419
Cndp2	1815.714	2.189322	1.008113	2.171704	0.029878	0.210419
CG32365	120.3512	-6.3783	2.937644	-2.17123	0.029914	0.210419
Zif	1276.869	-2.81289	1.296383	-2.1698	0.030022	0.210678
CG6424	701.1973	-2.43649	1.122652	-2.1703	0.029984	0.210678
CG9760	276.5509	4.734184	2.181951	2.169702	0.030029	0.210678
mRpS33	1802.99	1.775182	0.819338	2.166604	0.030265	0.212146
Lpt	102.9718	5.476798	2.529265	2.165372	0.030359	0.212621
Surf6	460.5218	-4.66816	2.157707	-2.16348	0.030504	0.213449
CG8319	555.7655	-3.70648	1.713526	-2.16307	0.030536	0.213483
Gclm	2582.211	-1.83139	0.847192	-2.16172	0.03064	0.214027

CG11454	768.7772	3.342859	1.54697	2.160908	0.030702	0.214064
barr	109.7205	5.970649	2.763847	2.160267	0.030752	0.214064
CG16908	201.1758	-6.12852	2.836316	-2.16073	0.030716	0.214064
GNBP2	120.0015	-6.59264	3.051481	-2.16047	0.030736	0.214064
PIP82	63.74152	-8.30809	3.846828	-2.15972	0.030794	0.214171
Pal1	1652.901	-2.48211	1.150006	-2.15835	0.030901	0.214728
CG18764	304.2054	4.783735	2.218164	2.15662	0.031035	0.215475
CG4716	79.04047	8.394302	3.898249	2.153352	0.031291	0.216875
Bap60	1382.455	-3.06317	1.422464	-2.15343	0.031285	0.216875
PIG-H	621.0078	-3.42469	1.590668	-2.15299	0.03132	0.216887
Mms19	640.0486	-3.15373	1.4659	-2.15139	0.031445	0.217568
CG8042	919.5113	2.915682	1.356154	2.149964	0.031558	0.218161
CG4768	2212.34	-1.59395	0.742216	-2.14756	0.031749	0.219289
hbn	251.2863	3.99483	1.860895	2.146725	0.031815	0.219372
ND-49	11393	1.399654	0.651985	2.146757	0.031813	0.219372
X11L	1344.943	2.944026	1.37168	2.146292	0.03185	0.219421
CG8435	464.5807	3.32056	1.547561	2.145673	0.031899	0.219573
CG17230	2089.011	-2.22762	1.039401	-2.14317	0.032099	0.22076
Sec20	447.2717	-4.25055	1.983924	-2.14249	0.032154	0.220947
CheB53a	83.55624	-8.16827	3.813624	-2.14187	0.032204	0.221105
Oseg5	132.0242	6.36288	2.971199	2.141519	0.032232	0.221107
kcc	17737.55	1.166676	0.545762	2.137703	0.032541	0.223034
Lap1	1563.849	1.84182	0.861868	2.137008	0.032597	0.223231
Diap1	4600.053	-1.5179	0.71047	-2.13647	0.032641	0.223337
FIG4	155.7213	5.253981	2.460516	2.135316	0.032735	0.223793
Tcs3	105.0606	6.800484	3.188357	2.132912	0.032932	0.224946
CG15237	778.5703	3.129441	1.468688	2.130774	0.033108	0.225955
Ggt-1	63.99092	-8.25588	3.879545	-2.12805	0.033333	0.227297
Rpb8	860.6255	3.129477	1.472109	2.125846	0.033516	0.228353
Urod	1414.234	-2.39557	1.127625	-2.12444	0.033634	0.228528
Gmd	201.4693	4.85936	2.288748	2.123152	0.033741	0.228528
jv	469.8229	-2.81341	1.32499	-2.12334	0.033725	0.228528
garz	1469.966	2.66489	1.254349	2.124521	0.033627	0.228528
lncRNA:CR	80.37937	-8.17469	3.849227	-2.12372	0.033693	0.228528
CngB	660.0954	4.410523	2.076806	2.123704	0.033695	0.228528
ergic53	1082.006	-2.80368	1.320497	-2.1232	0.033737	0.228528
CG12942	122.4057	-8.12737	3.829509	-2.1223	0.033813	0.228645
asRNA:CR4	194.4818	-4.03051	1.899152	-2.12227	0.033815	0.228645
CG3651	135.5276	-5.67296	2.674254	-2.12132	0.033895	0.228945
lute	868.7817	3.072641	1.448635	2.12106	0.033917	0.228945
Rpn2	2240.594	-1.80137	0.850142	-2.1189	0.034099	0.229596
mesh	306.9389	4.924656	2.323947	2.119091	0.034083	0.229596
APP-BP1	2111.811	2.201601	1.039021	2.118919	0.034097	0.229596
Fdh	720.4438	-3.18607	1.504653	-2.11748	0.034219	0.230211
CG14864	322.7109	-3.50263	1.655902	-2.11524	0.034409	0.230333
CG14502	356.2138	-3.41675	1.615086	-2.11552	0.034385	0.230333
CG14440	839.3655	2.517713	1.189671	2.11631	0.034318	0.230333
lncRNA:CR	89.61315	8.226942	3.887637	2.116181	0.034329	0.230333
CG7509	22827.27	-0.90723	0.428727	-2.1161	0.034336	0.230333
CG14352	213.4933	-5.7613	2.723327	-2.11554	0.034384	0.230333

Xpc	4124.812	-2.08365	0.985342	-2.11464	0.03446	0.230483
asRNA:CR4	525.4796	-3.39319	1.606408	-2.11228	0.034662	0.231639
CG3309	279.2528	-5.24324	2.483492	-2.11124	0.034752	0.232045
Rab39	1240.972	2.484787	1.178564	2.108318	0.035003	0.233531
CG5599	162.5673	-6.40979	3.043684	-2.10593	0.03521	0.234716
lncRNA:CR	319.4154	-3.93912	1.870968	-2.10539	0.035257	0.234832
Myo95E	688.0318	2.797884	1.329167	2.10499	0.035292	0.234871
mip130	118.6368	-6.92632	3.292182	-2.10387	0.03539	0.235326
MetRS	1074.643	-2.60502	1.238448	-2.10346	0.035426	0.235372
RanBP3	168.6788	-6.31335	3.004047	-2.10162	0.035587	0.236172
snoRNA:gc	85.6192	-8.19269	3.89867	-2.10141	0.035605	0.236172
Ttc30	182.5491	-6.20256	2.952857	-2.10053	0.035682	0.236209
Srlp	1201.76	2.367055	1.126952	2.100404	0.035693	0.236209
Nup58	100.5899	-8.07774	3.845923	-2.10034	0.035699	0.236209
CG2915	955.9955	-2.27197	1.083015	-2.09782	0.035921	0.237384
for	577.1121	-2.67783	1.276585	-2.09765	0.035936	0.237384
CG11504	845.0313	4.255597	2.029299	2.097078	0.035987	0.237524
CG33331	374.4024	3.324712	1.585882	2.096443	0.036043	0.2377
CG4752	513.9044	-3.5494	1.694393	-2.09479	0.03619	0.238471
Spn42Dd	174.12	7.216904	3.446476	2.093995	0.03626	0.238742
CG2712	194.5193	6.15279	2.941224	2.091915	0.036446	0.239389
Ppt2	815.0611	-2.80757	1.342333	-2.09156	0.036478	0.239389
Nprl3	606.1558	-3.40899	1.629787	-2.09168	0.036467	0.239389
Pus7	87.75545	-8.03684	3.841688	-2.09201	0.036438	0.239389
RfC3	420.2724	-3.53229	1.689831	-2.09032	0.036589	0.239921
PolH	591.1932	3.963434	1.897521	2.088743	0.036731	0.240067
Ugt302K1	516.7517	-3.0591	1.464148	-2.08934	0.036677	0.240067
CG10927	73.58641	8.106286	3.880648	2.0889	0.036717	0.240067
mio	121.5483	6.243691	2.989018	2.088877	0.036719	0.240067
Cad87A	1574.861	-2.09809	1.004656	-2.08837	0.036765	0.240092
CG31523	1724.964	1.756317	0.841217	2.08783	0.036813	0.240214
CG8272	187.0621	5.912158	2.833257	2.086701	0.036915	0.240684
CG34430	436.2869	4.649596	2.228562	2.086367	0.036945	0.240685
CG42326	90.35201	-8.01929	3.845584	-2.08532	0.03704	0.241105
CG34376	1062.751	2.358383	1.131695	2.083938	0.037166	0.241728
Top3beta	446.2045	-3.44177	1.652409	-2.08288	0.037262	0.241962
Pp1alpha-9	3270.06	1.484926	0.712869	2.083026	0.037249	0.241962
CG8060	166.1092	6.381068	3.064158	2.082486	0.037298	0.242
CG5554	1237.737	2.006797	0.964233	2.081236	0.037412	0.242545
CG10089	1155.306	2.680369	1.288965	2.079473	0.037574	0.243396
Ir56a	459.7524	4.042581	1.944798	2.078663	0.037648	0.243682
Cyp6a15Ps	1557.116	-1.99113	0.958524	-2.07729	0.037775	0.244305
qvr	34967.11	0.814565	0.39221	2.076857	0.037815	0.244365
pod1	2551.677	-1.84237	0.887812	-2.07518	0.03797	0.245169
CG11350	136.1122	-6.78873	3.272143	-2.0747	0.038014	0.245257
CG10589	98.01135	-7.953	3.835568	-2.07349	0.038127	0.245591
Ice2	93.01024	-6.96061	3.356878	-2.07354	0.038122	0.245591
Pex14	2982.651	1.845371	0.890986	2.071156	0.038344	0.246496
CG8086	397.2571	3.331482	1.608323	2.071402	0.038321	0.246496
CG17806	269.8614	-4.59073	2.216682	-2.07099	0.03836	0.246496

GCS2beta	739.3104	-2.93195	1.416597	-2.06971	0.038479	0.247069
Alas	2436.747	-1.43505	0.693843	-2.06826	0.038616	0.247746
Nca	20773.37	0.900338	0.435672	2.066553	0.038776	0.248379
Est-6	213.7106	6.067059	2.935528	2.066769	0.038756	0.248379
glob1	4169.051	-1.22629	0.593877	-2.06488	0.038934	0.248993
CG8111	130.7741	5.625411	2.724176	2.064995	0.038923	0.248993
TBC1D23	1348.117	2.18327	1.058586	2.06244	0.039166	0.250275
Hers	3736.648	-1.22342	0.593433	-2.06159	0.039247	0.250591
tomboy40	69.7579	6.785258	3.296241	2.058484	0.039544	0.252288
Axud1	2016.971	1.697988	0.825516	2.05688	0.039698	0.253069
CG17760	116.1842	7.125761	3.465927	2.055946	0.039788	0.253441
CG5026	2543.158	-1.72937	0.841553	-2.05498	0.039881	0.253835
lncRNA:CR	540.0747	-2.68778	1.308371	-2.0543	0.039947	0.254052
l(2)efl	80.61701	-8.00051	3.895453	-2.05381	0.039994	0.254152
H	1230.604	-2.14001	1.042203	-2.05335	0.040039	0.254233
fat-spondi	1909.006	-2.08924	1.018153	-2.05199	0.040171	0.25487
htk	886.9545	-2.19799	1.071502	-2.05132	0.040236	0.255082
mRpL11	376.5976	3.636177	1.773659	2.050098	0.040355	0.255634
CG5003	677.7734	-2.79238	1.363782	-2.04752	0.040607	0.257027
CG2200	543.8187	-3.84323	1.877705	-2.04677	0.04068	0.25729
CG32354	368.6616	-3.46132	1.691851	-2.04588	0.040768	0.257643
Rh7	355.7074	-4.37476	2.13993	-2.04435	0.040919	0.258393
mt:ATPase	216804	0.76895	0.376255	2.043691	0.040984	0.258599
nAChRalpl	18441.58	0.857021	0.419418	2.043361	0.041017	0.258602
CDase	6962.216	-1.13332	0.555208	-2.04126	0.041225	0.259104
Clbn	1129.863	-2.89929	1.420112	-2.04159	0.041192	0.259104
lncRNA:CR	207.0608	-5.29955	2.595274	-2.042	0.041152	0.259104
asRNA:CR4	257.7987	3.50775	1.718234	2.041485	0.041203	0.259104
CG1646	2775.614	1.421185	0.696519	2.040411	0.041309	0.259225
CG34310	68.31972	7.846583	3.845004	2.040722	0.041279	0.259225
eIF2Bbeta	2766.016	-1.92935	0.946255	-2.03894	0.041456	0.259945
JMJD5	584.9014	-3.66847	1.799983	-2.03806	0.041544	0.260293
CG8066	449.9857	-3.53989	1.738793	-2.03583	0.041767	0.261399
CG13458	488.7261	-3.21436	1.579038	-2.03564	0.041786	0.261399
CG6907	191.8811	-4.8328	2.37486	-2.03498	0.041853	0.261407
CG17600	636.1221	2.92935	1.4394	2.035118	0.041839	0.261407
140up	321.222	4.142577	2.037904	2.032763	0.042076	0.262601
brv3	89.14538	7.866328	3.87109	2.032071	0.042147	0.262777
beat-IIb	2151.074	1.784127	0.878085	2.031839	0.04217	0.262777
Amacr	853.4449	-2.62295	1.291728	-2.03057	0.042298	0.263167
Arv1	268.4205	3.892349	1.916828	2.03062	0.042294	0.263167
CG33116	2234.941	-1.76144	0.867733	-2.02993	0.042364	0.263371
CG4953	219.1634	-4.77653	2.353471	-2.02957	0.0424	0.263393
kar	222.2859	4.883869	2.407512	2.028596	0.042499	0.263806
SCCRO3	1537.032	1.94937	0.961461	2.027509	0.04261	0.26429
Hsc70-2	144.0694	-6.35126	3.138808	-2.02346	0.043025	0.266657
CG31249	245.8867	4.440477	2.198384	2.019883	0.043396	0.268744
CG5757	87.48157	-6.83914	3.390036	-2.01742	0.043651	0.27012
SPR	1458.983	1.988743	0.986027	2.016926	0.043703	0.270233
MTA1-like	2472.746	1.519834	0.754049	2.015564	0.043846	0.270629

CG4459	123.1108	6.351328	3.151484	2.015345	0.043868	0.270629
Pex6	547.1948	-3.18314	1.579151	-2.01573	0.043828	0.270629
Zn72D	4576.827	-1.386	0.689089	-2.01136	0.044288	0.273006
axed	589.6423	2.414363	1.201704	2.009115	0.044525	0.274257
SF1	749.1142	-2.86807	1.429411	-2.00647	0.044806	0.275355
Non1	6265.88	1.149376	0.572809	2.006561	0.044796	0.275355
mRpL22	1673.785	1.598837	0.796625	2.007014	0.044748	0.275355
mRpL40	597.7726	2.402406	1.198958	2.003745	0.045097	0.276722
fz4	117.4064	5.739317	2.863905	2.004018	0.045068	0.276722
CG11374	486.0805	3.100183	1.549502	2.00076	0.045418	0.278355
CR43170	193.4151	-4.88588	2.442177	-2.00062	0.045433	0.278355
DIP-delta	6233.02	1.055748	0.527931	1.999783	0.045524	0.278519
lncRNA:CR	133.3537	6.044161	3.022485	1.999732	0.045529	0.278519
S6kII	1388.287	-1.89408	0.947476	-1.99908	0.045599	0.278523
CG7991	871.5946	2.486709	1.243787	1.999304	0.045575	0.278523
CG7656	1285.375	-2.15362	1.077764	-1.99823	0.045691	0.278874
CG17646	1375.01	-2.56202	1.282452	-1.99775	0.045744	0.278982
CG12071	2861.088	-1.38317	0.692574	-1.99714	0.04581	0.279172
THG	179.1605	-6.13375	3.075049	-1.99468	0.046077	0.280484
mRpS2	1145.822	2.334855	1.170634	1.994523	0.046095	0.280484
Rpt6	1025.652	-2.3663	1.186734	-1.99396	0.046156	0.280642
CG4497	855.1118	3.260829	1.636114	1.993033	0.046258	0.280876
Acph-1	655.6219	2.797657	1.403761	1.992973	0.046264	0.280876
CG11807	2904.932	1.419068	0.712187	1.99255	0.046311	0.280903
Su(var)2-H	2245.964	1.704816	0.855705	1.992293	0.046339	0.280903
mthl8	2040.095	1.872571	0.940275	1.991514	0.046424	0.281209
l(3)72Dp	373.5511	4.820518	2.424547	1.988214	0.046788	0.282984
CG15202	1535.245	2.660906	1.338316	1.98825	0.046784	0.282984
CG16868	1347.036	2.951552	1.485187	1.987327	0.046886	0.283152
Snx1	2011.478	2.090875	1.052078	1.987375	0.046881	0.283152
RIOK1	299.8654	4.25182	2.140217	1.98663	0.046963	0.283405
Vps11	1406.879	2.087446	1.05184	1.984566	0.047193	0.284575
CG33310	205.0628	4.397754	2.217272	1.983407	0.047322	0.284926
CG13405	107.5731	6.239835	3.145843	1.983517	0.04731	0.284926
Rubicon	483.5554	-2.8002	1.412331	-1.98268	0.047403	0.285202
GlcAT-S	4349.922	-2.10201	1.061042	-1.98108	0.047582	0.286062
p47	809.7203	-3.24239	1.637376	-1.98024	0.047677	0.286308
CG8009	480.3909	2.740225	1.384116	1.979766	0.04773	0.286308
CG1486	1504.663	-2.51461	1.27009	-1.97987	0.047718	0.286308
Vps51	704.4347	-3.87215	1.956598	-1.97902	0.047813	0.286595
lva	1151.95	2.409867	1.218278	1.978093	0.047918	0.287009
cher	167.4667	5.631477	2.847842	1.977455	0.04799	0.287226
Cog7	1232.668	2.831298	1.432234	1.97684	0.04806	0.287427
PGRP-LA	2672.202	-2.19679	1.111666	-1.97612	0.048141	0.287698
Nxt1	751.911	-2.36726	1.198654	-1.97493	0.048276	0.288291
sNPF	1929.616	-2.62993	1.331962	-1.97447	0.048328	0.288386
Hsp68	598.0582	-2.45056	1.242363	-1.9725	0.048553	0.289512
POLDIP2	1184.634	2.73736	1.388264	1.971786	0.048634	0.289711
CG34015	1869.472	2.621155	1.329636	1.971333	0.048686	0.289711
TORIP	2034.784	1.443678	0.732364	1.971259	0.048694	0.289711

CG46397	314.9272	-3.7676	1.912762	-1.96972	0.048871	0.290545
CG33640	365.6556	-4.16507	2.116711	-1.96771	0.049102	0.291696
CG5321	1252.076	-1.91624	0.973994	-1.9674	0.049137	0.291696
CG4666	319.3761	4.081799	2.075065	1.967071	0.049175	0.291707
pn	306.3275	4.114035	2.093134	1.965491	0.049357	0.292572
CG13313	95.88356	-7.38506	3.762068	-1.96303	0.049643	0.294045
CG32176	766.5889	2.171783	1.107316	1.961303	0.049844	0.295019
CG4045	767.3254	-2.55778	1.305294	-1.95955	0.050049	0.296015
CG8837	66.90387	7.580787	3.873894	1.956891	0.05036	0.297638
msps	3941.245	1.194725	0.610758	1.956135	0.050449	0.297944
tra2	3309.114	2.815375	1.439511	1.955785	0.05049	0.297968
CAH8	82.13328	-7.53683	3.856239	-1.95445	0.050648	0.298251
Pat1	2337.581	-1.62427	0.831172	-1.9542	0.050678	0.298251
Rph	1109.855	2.647794	1.354981	1.95412	0.050687	0.298251
lncRNA:CR	177.3416	-5.00536	2.560206	-1.95506	0.050576	0.298251
Qtzl	1189.987	2.247248	1.150672	1.952988	0.050821	0.29882
Irp-1B	1079.51	-2.56837	1.31691	-1.9503	0.05114	0.299709
Manf	4386.112	1.250203	0.640827	1.950921	0.051066	0.299709
mib2	1963.713	1.589631	0.814968	1.950544	0.051111	0.299709
ben	5201.889	-1.15161	0.590196	-1.95124	0.051029	0.299709
Snx21	436.0153	3.461901	1.775202	1.950144	0.051159	0.299709
beat-Ic	4271.438	1.267064	0.650121	1.948966	0.051299	0.300069
Mtp	658.8395	-2.78131	1.426917	-1.94917	0.051275	0.300069
CG9521	74.61461	7.49598	3.846679	1.948689	0.051333	0.300069
CG5861	3206.156	1.252707	0.64301	1.948193	0.051392	0.300197
RpLP0-like	285.5221	4.646331	2.385755	1.94753	0.051471	0.300442
CG8306	68.30741	-7.5358	3.870308	-1.94708	0.051525	0.300539
CG30392	2626.604	-2.26045	1.16214	-1.94507	0.051766	0.301725
Sec31	2060.278	-1.5164	0.77997	-1.94418	0.051873	0.302131
Cap-D3	907.9253	3.165024	1.628931	1.943007	0.052015	0.302235
Nup75	271.7323	5.194947	2.672925	1.943544	0.05195	0.302235
TBCC	1276.011	-1.91918	0.987847	-1.94279	0.052042	0.302235
Cep97	696.958	3.175229	1.634361	1.942795	0.052041	0.302235
CG4287	1690.955	1.813781	0.934092	1.941758	0.052166	0.302739
CG8910	730.1187	2.25921	1.164033	1.940847	0.052277	0.302802
dpa	128.5973	6.556025	3.377789	1.940922	0.052268	0.302802
CG7394	1331.925	1.818355	0.936942	1.940734	0.052291	0.302802
Der-2	3347.543	-2.26961	1.169808	-1.94016	0.052361	0.302989
CG9646	4704.17	1.430605	0.737487	1.939838	0.052399	0.302995
Nup37	148.6359	7.017407	3.618206	1.939471	0.052444	0.303035
CG11523	830.5088	-2.86157	1.475836	-1.93895	0.052508	0.303163
Capr	917.9164	2.032056	1.048171	1.938668	0.052542	0.303163
Vps13D	1555.906	-2.29436	1.183728	-1.93825	0.052593	0.30324
CG32647	3640.303	-1.22495	0.632404	-1.93697	0.052749	0.303781
Taf8	230.8218	-4.93558	2.548239	-1.93686	0.052762	0.303781
Cyp12a4	2197.09	2.550219	1.317172	1.936132	0.052852	0.304076
JMJD7	748.7031	2.303648	1.190108	1.935663	0.052909	0.304189
Atg9	747.137	4.061287	2.09958	1.934334	0.053072	0.30469
CG44098	8094.822	-1.56751	0.810235	-1.93464	0.053035	0.30469
lost	7379.066	-1.00108	0.517679	-1.93378	0.053141	0.304865

CG12118	92.52845	-6.81133	3.523658	-1.93303	0.053233	0.304958
Mul1	506.2078	4.055104	2.097467	1.933334	0.053195	0.304958
CG7943	3197.534	1.811796	0.937801	1.931962	0.053364	0.305493
CG6230	2141.307	-2.57409	1.333526	-1.93029	0.053571	0.30646
CG31300	1315.896	2.67464	1.386781	1.928667	0.053772	0.30739
CG3847	999.425	-2.24365	1.164358	-1.92694	0.053987	0.308172
CG2641	247.8879	-4.82642	2.504937	-1.92676	0.054009	0.308172
Arpc5	622.9952	2.590756	1.3447	1.926642	0.054024	0.308172
TppII	2101.886	-2.20548	1.146541	-1.9236	0.054405	0.310124
CG2016	667.0227	-3.7661	1.958613	-1.92284	0.0545	0.310445
CG7806	1765.415	-2.46319	1.282646	-1.9204	0.054807	0.311753
Hsp27	169.5863	-6.07681	3.164017	-1.9206	0.054782	0.311753
gt	206.2008	4.921831	2.563916	1.919654	0.054902	0.311972
Ac13E	323.0404	3.3763	1.759811	1.918559	0.05504	0.311972
beat-VII	8456.244	1.135427	0.591685	1.918973	0.054988	0.311972
Pdfr	1553.094	-1.57166	0.819174	-1.91859	0.055037	0.311972
Gp210	511.7236	-3.68395	1.919765	-1.91896	0.05499	0.311972
CG31104	68.55416	7.393389	3.855714	1.917515	0.055173	0.312501
twe	346.5103	-4.85365	2.536109	-1.91382	0.055644	0.314948
Nepl3	286.2054	5.223376	2.729862	1.913421	0.055694	0.315011
lncRNA:CR	331.1606	-3.35663	1.755361	-1.91221	0.055849	0.315663
PCNA2	320.7195	4.118077	2.154854	1.91107	0.055996	0.31627
rnh1	993.8837	-2.99849	1.569585	-1.91037	0.056085	0.316555
CG15861	225.4511	4.046489	2.119382	1.909278	0.056226	0.317127
CG12105	510.8156	-2.93981	1.54142	-1.90721	0.056493	0.31841
wuho	553.6737	3.12903	1.641122	1.90664	0.056567	0.318602
Prp4k	662.7402	2.261301	1.18665	1.905617	0.0567	0.319126
Synj	7538.994	-1.22547	0.643436	-1.90457	0.056837	0.319515
CG1161	4370.649	-1.09172	0.57324	-1.90447	0.056849	0.319515
CG9896	1787.149	-2.3656	1.242661	-1.90365	0.056955	0.319889
Sema1b	3542.466	1.668117	0.876742	1.902631	0.057089	0.320416
Syx8	574.327	2.360131	1.240973	1.901839	0.057192	0.320772
IKKepsilon	745.2161	3.124227	1.644333	1.899997	0.057434	0.3219
CG16854	1851.305	-2.52619	1.329898	-1.89954	0.057494	0.322013
CG31548	791.9065	-2.03858	1.073851	-1.89838	0.057646	0.322577
ASPP	2794.274	1.371454	0.722517	1.898161	0.057675	0.322577
lncRNA:spl	379.9648	4.00596	2.111607	1.897114	0.057813	0.323124
lncRNA:CR	83.32897	7.341261	3.870976	1.896488	0.057895	0.323361
CG8516	471.8205	-3.67669	1.941006	-1.89422	0.058196	0.323913
Rbcn-3A	5919.533	1.20858	0.637913	1.894586	0.058147	0.323913
CR40354	104.8918	5.146533	2.716781	1.89435	0.058179	0.323913
CDK2AP1	353.7465	4.091234	2.159753	1.894307	0.058184	0.323913
Cpsf5	4632.794	1.066185	0.562712	1.894727	0.058129	0.323913
CG7328	447.8493	3.321555	1.754687	1.892961	0.058363	0.324616
brp	26121.68	0.878066	0.464008	1.892351	0.058444	0.324843
CG17029	3150.114	-1.47769	0.781163	-1.89165	0.058537	0.325134
mt:ND5	77522.58	0.700917	0.370762	1.890474	0.058695	0.325589
wcy	3647.274	1.177072	0.622647	1.890433	0.0587	0.325589
CG17778	3370.258	1.176813	0.622771	1.88964	0.058806	0.32565
CG11425	334.593	-3.60473	1.907298	-1.88997	0.058762	0.32565

lncRNA:CR	420.3659	3.048727	1.613562	1.88944	0.058833	0.32565
CAH3	1469.081	-1.70347	0.902104	-1.88833	0.058982	0.326083
CG9691	14519.73	-1.01501	0.537541	-1.88825	0.058992	0.326083
Apoltp	232.0738	-4.41462	2.338809	-1.88755	0.059087	0.326157
CG8958	580.0384	-3.62025	1.917965	-1.88755	0.059087	0.326157
Pvr	714.8702	-3.12876	1.657895	-1.88719	0.059135	0.326199
Snp	254.0938	-3.87364	2.053073	-1.88675	0.059194	0.326297
Cyp6a2	602.0069	2.413353	1.280268	1.885036	0.059425	0.327347
CG14894	1427.159	2.007454	1.065759	1.883591	0.05962	0.328198
CG5800	205.6131	5.397159	2.866599	1.882774	0.059731	0.328582
Rcc1	365.991	2.777751	1.476551	1.881243	0.059939	0.3295
Gpdh3	209.4834	-3.8795	2.06331	-1.88023	0.060076	0.330029
prd1	3715.879	1.176409	0.626687	1.877187	0.060492	0.331408
Pur-alpha	18041.19	0.862725	0.459523	1.877434	0.060459	0.331408
CR40190	194.8484	-4.90572	2.613008	-1.87742	0.06046	0.331408
CG30419	600.3822	2.923411	1.55707	1.877508	0.060448	0.331408
Aef1	2269.766	-1.85114	0.987086	-1.87536	0.060744	0.331541
CG14459	7253.063	1.008271	0.537559	1.875647	0.060704	0.331541
csul	751.8566	-3.21549	1.714782	-1.87516	0.060771	0.331541
CG3520	138.9014	5.823264	3.103711	1.876226	0.060624	0.331541
CG17032	106.4841	-6.16449	3.287541	-1.87511	0.060778	0.331541
Adi1	1145.647	2.779448	1.482085	1.875363	0.060743	0.331541
SerRS	642.2161	-3.12676	1.667692	-1.8749	0.060806	0.331541
Tsf2	510.7417	-3.23027	1.723284	-1.87449	0.060863	0.331628
lncRNA:CR	67.98101	7.253593	3.870766	1.873942	0.060938	0.331812
CG42521	87.67812	7.226693	3.864006	1.870259	0.061448	0.33436
Nepl21	382.0383	3.822447	2.04644	1.867852	0.061783	0.335954
lncRNA:CR	140.4323	5.269914	2.822133	1.867352	0.061852	0.336106
Gbeta5	7447.721	-0.95771	0.513031	-1.86677	0.061933	0.336318
lectin-46Ca	423.5051	-3.6777	1.970741	-1.86615	0.06202	0.336561
CG11737	1569.039	-1.8442	0.988503	-1.86565	0.06209	0.336714
CG18476	581.7805	2.418526	1.297369	1.864177	0.062297	0.337606
HUWE1	4044.718	-1.14951	0.616744	-1.86384	0.062345	0.337636
CG6013	781.5532	-1.99696	1.071715	-1.86333	0.062415	0.337671
aft	326.0835	4.041253	2.168992	1.863194	0.062435	0.337671
CG32686	798.7654	-3.26087	1.751457	-1.8618	0.062631	0.337944
lncRNA:CR	2498.15	2.291094	1.230392	1.862085	0.062591	0.337944
CG18259	975.2053	2.003349	1.075815	1.86217	0.062579	0.337944
Nufip	169.5917	-5.13404	2.757803	-1.86164	0.062654	0.337944
mRpS10	3748.8	1.287286	0.69215	1.859836	0.062909	0.33909
CG15561	134.0692	-5.38656	2.896842	-1.85946	0.062962	0.33915
CG43742	662.2992	-3.3781	1.817113	-1.85905	0.06302	0.339237
gek	2055.779	2.091799	1.125966	1.857781	0.0632	0.339976
Gart	823.3965	-2.26192	1.218199	-1.85677	0.063343	0.340175
Mer	239.5631	3.576151	1.925869	1.856903	0.063325	0.340175
Rbsn-5	810.3907	-2.19126	1.180235	-1.85663	0.063364	0.340175
CG5276	899.9185	-1.92384	1.036817	-1.85552	0.063522	0.340793
Mekk1	1904.636	1.879422	1.01318	1.854973	0.0636	0.340985
Spec2	299.9368	4.026097	2.171083	1.854419	0.063679	0.341035
Arl8	4078.682	-1.62414	0.875874	-1.85431	0.063694	0.341035

brm	1202.508	-2.48671	1.34161	-1.85353	0.063807	0.34141
E(z)	1480.355	2.273043	1.227327	1.852027	0.064022	0.342332
Shc	428.4593	-3.81277	2.063213	-1.84798	0.064606	0.345224
CG10565	1490.537	1.914595	1.037833	1.8448	0.065067	0.346904
SPoCk	5272.995	1.003393	0.543957	1.844618	0.065093	0.346904
pita	1015.874	1.837388	0.995794	1.845149	0.065016	0.346904
PDZ-GEF	3453.046	1.400629	0.758954	1.845472	0.064969	0.346904
Tailor	2671.015	1.606029	0.871196	1.843475	0.06526	0.347561
Alg9	2876.802	1.509439	0.819333	1.842279	0.065434	0.34826
Jupiter	5764.015	1.053958	0.572689	1.840368	0.065714	0.349518
Or13a	134.8253	5.128996	2.788621	1.839259	0.065877	0.350152
CG44837	3610.197	1.416407	0.770391	1.838558	0.06598	0.350468
CG43085	70.91953	-6.81738	3.709617	-1.83776	0.066098	0.350861
Parp	4433.767	1.087188	0.591745	1.837257	0.066172	0.351022
Eogt	329.2101	-4.1788	2.275422	-1.8365	0.066284	0.351386
Con	6769.621	0.992928	0.540769	1.836142	0.066337	0.351431
PAN2	1069.256	1.979654	1.079059	1.834612	0.066563	0.352398
CG2790	465.8267	-2.95234	1.60955	-1.83426	0.066615	0.35244
CG12241	252.3794	4.38595	2.392474	1.833228	0.066769	0.35266
mip40	375.5024	2.838931	1.548946	1.832815	0.06683	0.35266
ix	520.4621	-3.34404	1.823916	-1.83344	0.066737	0.35266
Sce	831.0602	-2.51342	1.371353	-1.8328	0.066832	0.35266
DNAlig3	712.2498	2.515635	1.373946	1.830956	0.067107	0.353878
mtm	1184.911	-2.63853	1.443266	-1.82816	0.067525	0.355796
fd102C	402.3852	-4.39813	2.406453	-1.82764	0.067604	0.355796
Dref	2587.334	-1.64917	0.902243	-1.82786	0.067571	0.355796
CG15643	757.5769	-2.71133	1.484474	-1.82646	0.067781	0.356496
CG14805	925.7035	-2.97378	1.628942	-1.82559	0.067912	0.356527
Nsf2	873.4614	-2.25577	1.235672	-1.82554	0.067919	0.356527
sti	146.3123	-4.90069	2.684523	-1.82553	0.06792	0.356527
ana1	812.7773	3.008623	1.648579	1.824979	0.068004	0.356703
CG10948	13343.22	0.953141	0.522349	1.824722	0.068043	0.356703
CG12007	1158.931	-2.47655	1.357783	-1.82396	0.068157	0.35707
atilla	263.6808	-5.16806	2.837674	-1.82123	0.068571	0.359005
osy	1360.2	-1.83381	1.007576	-1.82002	0.068756	0.359737
CG9596	334.7507	4.170398	2.292161	1.819418	0.068848	0.359953
Vps50	1161.415	-2.57356	1.415382	-1.81828	0.069022	0.359953
HDAC1	812.1445	-3.32251	1.827217	-1.81835	0.069011	0.359953
CG10353	8055.601	1.240184	0.681974	1.818521	0.068985	0.359953
Twdlalpha	103.4791	5.226577	2.873986	1.818581	0.068975	0.359953
RhoGEF2	1509.157	1.917975	1.05554	1.817056	0.069209	0.360693
BtbVII	3268.098	-1.16089	0.63919	-1.81619	0.069342	0.361153
Fsn	2252.16	1.328512	0.731886	1.815191	0.069495	0.361645
CG31751	8900.564	0.930365	0.512602	1.814984	0.069526	0.361645
EDTP	765.3655	-1.9839	1.093495	-1.81428	0.069635	0.361974
pasha	521.1563	-2.42964	1.34066	-1.81227	0.069944	0.363111
CG6664	1454.547	-1.69142	0.93321	-1.81247	0.069913	0.363111
Hsp67Bc	2670.989	1.78097	0.982903	1.811949	0.069994	0.363137
lncRNA:CR	77.80271	-7.00713	3.8692	-1.811	0.07014	0.363661
mRpS26	1373.438	2.321172	1.282386	1.810042	0.070289	0.364197

CG16972	530.9608	3.166308	1.752118	1.807132	0.070742	0.366305
Wdr59	1133.936	-2.33652	1.293592	-1.80623	0.070883	0.366799
pho	6194.313	0.960421	0.53187	1.805744	0.070958	0.366954
fmt	6312.051	1.019006	0.564533	1.805041	0.071068	0.367285
CG16717	1351.574	1.570997	0.870606	1.804486	0.071155	0.367497
lncRNA:CR	145.2738	5.862107	3.249441	1.804035	0.071226	0.367626
Hexo1	1277.902	-2.12108	1.176444	-1.80296	0.071395	0.368263
Wdr81	2452.952	2.226842	1.236595	1.800785	0.071737	0.369313
CG16898	118.0422	6.953107	3.860166	1.801246	0.071664	0.369313
CG15027	192.3378	4.225817	2.346595	1.80083	0.07173	0.369313
ana2	337.0325	-3.68783	2.049123	-1.79971	0.071906	0.369948
CG31785	2673.927	-1.63218	0.907063	-1.79941	0.071953	0.369954
CG11695	207.5974	-4.05012	2.251192	-1.7991	0.072003	0.36997
cold	407.4566	-3.92242	2.182027	-1.7976	0.07224	0.370818
Dhx15	1806.759	1.442428	0.802474	1.797476	0.07226	0.370818
ACXD	1390.762	-1.73602	0.966546	-1.79611	0.072478	0.371698
CG1434	67.69439	6.941592	3.866109	1.795498	0.072574	0.371955
CG9044	1235.704	1.852341	1.032513	1.794013	0.072811	0.372692
Ccm3	4317.42	-1.37123	0.764266	-1.79418	0.072785	0.372692
CG3348	191.1902	5.046392	2.814804	1.792804	0.073004	0.373443
Inos	29721.36	-0.84777	0.472985	-1.79239	0.073071	0.373545
Klp68D	2554.165	1.984026	1.107793	1.790971	0.073298	0.374468
CG7523	548.9823	-2.83082	1.580893	-1.79065	0.07335	0.374495
cyc	2430.963	1.515269	0.846637	1.789749	0.073494	0.374755
Sod1	36562.62	-0.76054	0.424906	-1.7899	0.07347	0.374755
scny	1536.971	-1.56433	0.874205	-1.78944	0.073544	0.374773
Adk2	843.2528	2.191628	1.225277	1.78868	0.073666	0.375157
elB	2832.568	1.578679	0.883425	1.786998	0.073938	0.3763
CG8289	2628.571	1.387424	0.776695	1.786317	0.074048	0.376622
CG11399	1541.306	2.73573	1.532577	1.785052	0.074253	0.37725
RNaseZ	419.721	-3.15682	1.768553	-1.78497	0.074266	0.37725
l(1)G0007	704.8622	2.544029	1.425763	1.784328	0.07437	0.377544
CG17637	123.9137	4.760741	2.668629	1.783966	0.074429	0.377604
CG7135	2721.666	2.321694	1.302302	1.782762	0.074625	0.378358
CG40498	13134.32	0.9355	0.52513	1.781463	0.074837	0.379192
Actbeta	5308.563	1.099223	0.617229	1.7809	0.074929	0.379419
eIF3a	2046.975	1.408248	0.791571	1.779055	0.075231	0.380467
AP-1mu	2839.664	-1.48037	0.832062	-1.77916	0.075213	0.380467
fws	432.958	2.553839	1.436357	1.777997	0.075404	0.380852
CG12259	281.5784	-3.45075	1.941103	-1.77772	0.075449	0.380852
nbs	673.5599	2.566121	1.44313	1.778163	0.075377	0.380852
Gmer	622.5542	-3.00498	1.690663	-1.7774	0.075503	0.380882
pds5	642.337	2.12988	1.19879	1.776691	0.075619	0.38123
DCTN3-p2⁄	1492.955	-2.44097	1.374623	-1.77574	0.075776	0.381781
psidin	673.2579	3.083382	1.73676	1.775365	0.075838	0.381794
lncRNA:CR	131.2057	-5.3433	3.010059	-1.77515	0.075874	0.381794
wfs1	679.1988	3.291455	1.856048	1.773367	0.076168	0.382114
Rab10	679.2426	-2.39702	1.351933	-1.77304	0.076223	0.382114
Haspin	4871.069	-0.98596	0.555845	-1.7738	0.076097	0.382114
Cpsf73	346.5571	3.373597	1.901914	1.77379	0.076098	0.382114

mRpS6	1234.542	1.592239	0.897921	1.773251	0.076187	0.382114
hay	493.1412	3.826495	2.156855	1.774108	0.076045	0.382114
nac	69.38527	-6.87461	3.878356	-1.77256	0.076302	0.382273
RpL40	41786.41	0.72339	0.408321	1.77162	0.076458	0.382813
Ced-12	1866.589	1.683824	0.951613	1.769443	0.07682	0.384148
CRMP	2998.954	1.303455	0.736644	1.769452	0.076818	0.384148
CG8229	1310.024	1.633932	0.924475	1.767417	0.077158	0.3856
mRpL41	1893.401	1.997144	1.13078	1.766165	0.077368	0.386344
Rai1	1686.577	-1.86	1.053256	-1.76595	0.077404	0.386344
CG11403	196.6626	5.141117	2.912226	1.765357	0.077504	0.386604
CG12341	1344.274	-2.4687	1.399137	-1.76445	0.077657	0.387125
Syx6	11566.84	0.839666	0.475971	1.764112	0.077713	0.387131
l(2)dtl	86.4685	6.43156	3.646283	1.763868	0.077754	0.387131
CG8407	109.6929	-5.57263	3.160262	-1.76334	0.077843	0.387331
ZnT86D	1549.951	-2.37837	1.349507	-1.7624	0.078002	0.387643
ValRS	759.9496	-2.67609	1.518301	-1.76256	0.077975	0.387643
Pal2	26280.26	-0.7353	0.417515	-1.76114	0.078215	0.388459
CG10904	789.5753	-2.40768	1.368373	-1.75952	0.078489	0.389581
Tsr1	462.3763	-2.41862	1.3769	-1.75657	0.078991	0.391651
CG10366	1165.204	2.519276	1.434705	1.755953	0.079096	0.391651
CG6236	2084.01	-1.27221	0.7246	-1.75574	0.079133	0.391651
MED25	125.1943	-5.94267	3.383389	-1.75643	0.079016	0.391651
Atf6	3582.035	1.139116	0.648832	1.755639	0.07915	0.391651
Past1	1347.871	-1.92377	1.096703	-1.75414	0.079407	0.392437
CycK	1663.283	2.129713	1.214018	1.754268	0.079385	0.392437
lncRNA:CR	179.0491	5.327767	3.039807	1.752666	0.079659	0.393444
gkt	793.2629	-2.50866	1.432883	-1.75078	0.079984	0.394803
Ubc7	1294.831	1.847837	1.055776	1.750217	0.080081	0.395039
Rab9	1970.582	-2.02077	1.15508	-1.74946	0.080211	0.395439
Idgf4	4136.79	-2.10797	1.205205	-1.74905	0.080282	0.395459
CG10362	1254.57	-1.80759	1.03358	-1.74887	0.080314	0.395459
GstE1	8408.579	-0.99195	0.567379	-1.74829	0.080413	0.395464
Fpps	796.9782	-2.09058	1.19559	-1.74858	0.080364	0.395464
CG45218	417.5752	-2.93086	1.678438	-1.74618	0.080779	0.397018
CG2182	1182.113	-2.09075	1.197715	-1.74561	0.080878	0.397029
CG2926	3647.431	-1.23493	0.707449	-1.7456	0.08088	0.397029
Sccpdh1	2141.879	-2.07632	1.189782	-1.74513	0.080963	0.397191
Srrm1	1105.241	1.882	1.079295	1.743731	0.081206	0.398143
CG4707	237.2776	-4.14439	2.378314	-1.74257	0.081408	0.398891
Cdc2rk	465.9214	-3.45101	1.980786	-1.74224	0.081466	0.398929
CG10492	1250.73	-2.79004	1.602503	-1.74105	0.081674	0.399706
MFS15	809.8833	-2.99296	1.719845	-1.74025	0.081815	0.400151
Nep1	617.1727	3.049997	1.753022	1.739851	0.081885	0.40025
Daxx	1485.776	1.638056	0.942069	1.738786	0.082072	0.400634
Afti	1507.054	2.301241	1.323653	1.738553	0.082113	0.400634
Mctp	1497.537	1.598664	0.919411	1.738791	0.082072	0.400634
CG12768	2203.476	-1.71448	0.986676	-1.73764	0.082275	0.401178
Mob4	3121.405	1.270918	0.732093	1.736007	0.082563	0.402336
IntS4	148.3766	-5.63321	3.246538	-1.73514	0.082715	0.402347
CG8545	183.6249	4.346481	2.504678	1.735345	0.08268	0.402347

CG9578	709.1287	-2.8851	1.662446	-1.73546	0.08266	0.402347
CG3156	389.5592	-2.97655	1.716814	-1.73376	0.08296	0.403048
CG42878	376.9589	3.719125	2.144906	1.733933	0.08293	0.403048
Ssadh	7984.198	-0.91602	0.52865	-1.73276	0.083139	0.403674
Meics	323.2703	-3.31094	1.911309	-1.73229	0.083222	0.403833
CG2993	7066.795	-0.91901	0.530708	-1.73167	0.083332	0.404123
CG14184	3487.115	1.699199	0.981566	1.731111	0.083432	0.404363
CG6201	701.1767	2.593316	1.498783	1.730282	0.08358	0.404591
CG12213	3032.344	-1.54972	0.895611	-1.73035	0.083568	0.404591
Ca-alpha1I	8914.665	0.809476	0.468116	1.729221	0.08377	0.405265
Snx3	3271.862	1.142568	0.661021	1.728489	0.083901	0.405654
CG7368	575.0951	3.488049	2.018327	1.728188	0.083955	0.405671
CG18596	780.5323	-3.44566	1.994665	-1.72744	0.084089	0.406076
CG15734	68.11227	6.666756	3.864265	1.725233	0.084486	0.407746
Cypl	547.5358	3.106633	1.801831	1.724153	0.08468	0.408439
Vps35	1417.326	1.943991	1.128037	1.72334	0.084827	0.408657
CG12605	8934.627	0.831352	0.482401	1.723361	0.084823	0.408657
Slip1	7263.355	0.896316	0.520242	1.722885	0.084909	0.408808
CG4587	5273.268	1.046921	0.608101	1.721624	0.085138	0.409661
GlnRS	1439.117	-1.7834	1.036804	-1.72009	0.085416	0.410754
RpII15	1646.992	1.54108	0.896577	1.718849	0.085642	0.41096
GstE7	1498.172	2.147755	1.249617	1.71873	0.085664	0.41096
Prp19	793.5568	-2.55942	1.488633	-1.71931	0.085559	0.41096
Cht6	432.4786	3.891876	2.264138	1.718922	0.085629	0.41096
Rab23	243.3842	-4.07839	2.374413	-1.71764	0.085862	0.411653
CG8097	138.6989	5.285984	3.078447	1.717094	0.085962	0.411653
Ak1	9931.213	0.920448	0.53598	1.717318	0.085921	0.411653
Kdm4A	205.4587	5.524857	3.219177	1.716233	0.086119	0.411726
CG15891	1853.25	1.298829	0.756819	1.716169	0.086131	0.411726
tsr	4640.557	1.063009	0.619386	1.716229	0.08612	0.411726
Prat	158.6672	6.081901	3.544767	1.715741	0.086209	0.411856
CG14647	1546.032	1.748627	1.020355	1.713745	0.086576	0.412377
CG1105	1153.689	-2.21176	1.290368	-1.71405	0.086519	0.412377
Pkc53E	7422.172	0.858633	0.50096	1.713976	0.086533	0.412377
prel	2463.407	1.557677	0.908783	1.714025	0.086524	0.412377
Ptp10D	3599.312	1.271102	0.741694	1.713781	0.086569	0.412377
CG7744	67.17347	-6.34244	3.701694	-1.71339	0.086641	0.412444
CG14186	6177.328	1.013516	0.591646	1.713045	0.086704	0.4125
Dnz1	502.0743	2.218234	1.295725	1.711964	0.086903	0.412987
CG7084	14195.49	-0.91023	0.531783	-1.71165	0.086961	0.412987
Hr39	2420.157	1.257323	0.73454	1.711715	0.086949	0.412987
CG33521	705.6907	1.936022	1.131479	1.711054	0.087071	0.413266
Sh3beta	3187.388	1.234238	0.721563	1.710506	0.087172	0.413501
sage	728.0742	3.134825	1.834005	1.709278	0.0874	0.413846
CG31223	1044.309	-2.44407	1.429682	-1.70952	0.087354	0.413846
c11.1	926.3628	-2.05636	1.202932	-1.70945	0.087367	0.413846
SelR	8293.503	-0.82008	0.479916	-1.70879	0.08749	0.414031
l(2)37Cd	367.5942	3.566531	2.087873	1.708212	0.087597	0.414292
AdoR	632.4936	-2.03437	1.193419	-1.70466	0.088258	0.41626
Gadd45	2588.565	2.347966	1.37728	1.704785	0.088235	0.41626

mim	7375.902	1.07272	0.629316	1.704582	0.088272	0.41626
CG43115	265.6082	-4.44697	2.608101	-1.70506	0.088183	0.41626
lncRNA:CR	648.5985	2.956856	1.733922	1.705299	0.088139	0.41626
smog	4963.577	1.127768	0.661726	1.704284	0.088328	0.416278
Nepl20	109.0726	5.439146	3.193466	1.703211	0.088529	0.416568
CG7246	469.5489	3.339129	1.960436	1.703259	0.08852	0.416568
Gale	665.5568	-2.62585	1.541788	-1.70312	0.088545	0.416568
Git	806.0663	2.407655	1.414313	1.70235	0.08869	0.417003
Oscillin	1833.208	-1.48586	0.872987	-1.70204	0.088748	0.41703
CG7966	768.7711	-2.22575	1.309144	-1.70016	0.089101	0.418035
WASp	1421.6	-1.51277	0.889586	-1.70053	0.089031	0.418035
uex	8204.369	0.969429	0.570228	1.700071	0.089118	0.418035
tapas	3252.103	-1.06317	0.625581	-1.6995	0.089225	0.418294
SkpA	9361.337	0.937366	0.552051	1.69797	0.089513	0.419183
Rac2	1673.499	-1.5573	0.917172	-1.69794	0.089519	0.419183
CG34242	1335.892	-2.17278	1.280805	-1.69642	0.089807	0.420041
Tl	6301.772	0.932252	0.549515	1.696498	0.089792	0.420041
sty	8115.958	-0.86096	0.50787	-1.69524	0.09003	0.42084
LManI	978.0983	-2.223	1.31204	-1.69431	0.090206	0.421173
CG6791	713.9972	2.793568	1.648561	1.694549	0.090161	0.421173
Cdk12	2363.047	1.423316	0.840333	1.693753	0.090312	0.421422
Appl	21773.53	0.764715	0.451573	1.69345	0.09037	0.421446
CG5618	1262.18	-1.49129	0.881262	-1.69222	0.090604	0.422294
Hsc70-5	3293.242	-1.33333	0.788299	-1.6914	0.090761	0.422777
CG5946	8319.817	0.857573	0.507295	1.69048	0.090936	0.423349
CG44245	1600.986	2.285786	1.352628	1.689885	0.09105	0.423633
CG31717	2297.849	1.459818	0.864576	1.688478	0.091319	0.423707
lncRNA:CR	325.2626	-3.41402	2.021766	-1.68863	0.09129	0.423707
CG2034	363.1803	-3.80177	2.250662	-1.68918	0.091185	0.423707
wgn	1212.215	-1.51678	0.898039	-1.689	0.09122	0.423707
CG7668	1112.388	1.664422	0.985945	1.688149	0.091383	0.423707
AIF	1322.918	-1.77664	1.052347	-1.68827	0.09136	0.423707
Ranbp9	143.9602	4.867092	2.885718	1.686614	0.091678	0.424511
Mpc1	2395.654	-1.96745	1.166329	-1.68688	0.091627	0.424511
lncRNA:CR	95.51997	5.825476	3.454343	1.686421	0.091715	0.424511
ph-d	704.2687	2.373731	1.408997	1.684695	0.092047	0.425805
CG10877	185.9406	-3.79272	2.251932	-1.68421	0.092141	0.425994
Acox57D-p	771.6539	3.311368	1.966819	1.683617	0.092256	0.426278
CG43778	6225.984	0.86271	0.512643	1.682868	0.092401	0.426701
Gs1	8193.521	1.027924	0.610932	1.68255	0.092462	0.42674
RYa-R	159.2199	5.054892	3.006156	1.681514	0.092663	0.427422
CG3568	361.5026	-3.34956	1.994819	-1.67913	0.093127	0.428518
CG10137	766.3777	2.472829	1.472869	1.67892	0.093168	0.428518
spas	992.9759	-2.28556	1.361067	-1.67924	0.093105	0.428518
ave	2018.581	1.679146	0.999578	1.679854	0.092986	0.428518
Kr	1122.76	-2.06216	1.228103	-1.67914	0.093124	0.428518
wake	1230.07	-1.62263	0.967204	-1.67765	0.093415	0.429408
PSMG1	857.2849	2.576944	1.537208	1.676379	0.093664	0.430183
CG4424	368.3491	-3.16477	1.888014	-1.67624	0.093691	0.430183
t-cup	290.8421	-3.46519	2.067599	-1.67595	0.093748	0.430202

CG11790	6393.447	0.879395	0.524831	1.675577	0.093821	0.43029
Yif1	1732.974	-2.0605	1.23048	-1.67455	0.094022	0.430474
CG30373	791.3475	1.979715	1.182195	1.67461	0.094011	0.430474
Urm1	532.9823	2.918433	1.742304	1.675043	0.093926	0.430474
CG15073	669.9093	-2.73597	1.634351	-1.67404	0.094122	0.430687
CG34266	136.3872	-5.39989	3.232269	-1.67062	0.094797	0.433438
pHCl-1	3429.378	1.222012	0.731549	1.670445	0.094831	0.433438
Taf1	1568.181	1.481527	0.8873	1.669702	0.094978	0.433862
Edem1	683.2102	-2.45507	1.472812	-1.66693	0.095529	0.436128
CG8916	362.7631	3.628966	2.178837	1.665552	0.095803	0.437131
RabX4	2389.966	1.184325	0.711403	1.664775	0.095958	0.437589
CG14868	2208.504	1.71724	1.03181	1.664299	0.096053	0.437774
CG6567	67.14641	6.378528	3.836572	1.662559	0.096401	0.437868
CG12662	137.0833	4.95136	2.977935	1.662683	0.096376	0.437868
Prosalpha7	1484.364	-2.03033	1.220569	-1.66343	0.096227	0.437868
CG14043	1208.511	-1.62761	0.978528	-1.66333	0.096247	0.437868
FeCH	2000.702	-2.12947	1.279897	-1.66378	0.096157	0.437868
RhoGAP68	2436.072	-1.7547	1.055365	-1.66265	0.096384	0.437868
Nup62	439.7779	3.074675	1.850677	1.661378	0.096638	0.438695
Pask	1193.962	1.916117	1.154127	1.66023	0.096868	0.439494
CG13933	1280.758	-2.07542	1.251024	-1.65898	0.09712	0.440388
Rnf146	566.9266	-2.69243	1.62385	-1.65805	0.097307	0.440986
Exn	1450.239	1.484592	0.89562	1.657613	0.097396	0.441139
nSMase	958.2528	-2.58805	1.561674	-1.65723	0.097474	0.441243
AP-1sigma	7638.456	0.986213	0.5954	1.656388	0.097643	0.441762
CG2540	700.645	2.317823	1.399594	1.656069	0.097708	0.441805
RpII18	2540.391	1.137924	0.68772	1.654632	0.097999	0.442125
CG15270	4964.685	1.21603	0.734701	1.655136	0.097897	0.442125
Mps1	65.12655	-6.29604	3.804073	-1.65508	0.097909	0.442125
lncRNA:CR	876.5326	1.912534	1.155686	1.65489	0.097947	0.442125
Jra	1270.469	1.961908	1.186391	1.653678	0.098193	0.442503
Chrac-16	172.7549	3.537574	2.138944	1.653889	0.09815	0.442503
CG4364	1688.882	-1.46115	0.883885	-1.6531	0.098311	0.442786
CG7718	845.2252	2.923294	1.771335	1.650333	0.098875	0.443505
CG4553	151.1856	4.563251	2.765194	1.650246	0.098893	0.443505
CG9686	35266.09	-0.94104	0.569938	-1.65113	0.098713	0.443505
Syn	18924.17	0.870227	0.527111	1.650937	0.098752	0.443505
GC	1190.068	-2.4248	1.469213	-1.65041	0.098859	0.443505
CG32640	139.3121	5.051592	3.059281	1.651235	0.098691	0.443505
CG1764	1764.902	-1.44805	0.877489	-1.65022	0.098897	0.443505
CG5290	116.5576	-5.62879	3.411082	-1.65015	0.098913	0.443505
Cyp4ac3	689.6547	-2.59568	1.573722	-1.64939	0.099068	0.443952
bves	3212.948	1.375033	0.833822	1.649073	0.099133	0.443994
Hip14	640.0183	-2.54893	1.546383	-1.64832	0.099288	0.44444
La	1427.991	1.614979	0.980326	1.647389	0.099478	0.445045
CenG1A	1130.282	-2.00698	1.218692	-1.64683	0.099593	0.445312
Dsp1	12166.96	0.743836	0.452023	1.645569	0.099852	0.446223
Lnk	3385.038	-1.53557	0.933708	-1.6446	0.100053	0.446536
CIA30	1406.814	2.229892	1.356035	1.64442	0.100089	0.446536
Girdin	785.5866	-1.90448	1.15789	-1.64479	0.100013	0.446536

Dis3	275.3901	-3.49664	2.126972	-1.64395	0.100186	0.446717
step	4356.92	-1.0469	0.636959	-1.6436	0.100259	0.446797
Tsen54	445.149	-3.36233	2.046092	-1.64329	0.100322	0.44683
Rab6	1701.274	1.284035	0.781991	1.642008	0.100588	0.446831
Rpn13	1502.617	-1.50966	0.919237	-1.64229	0.100529	0.446831
Mef2	4210.46	0.997374	0.607322	1.642249	0.100538	0.446831
CG42808	945.4676	-2.2057	1.343034	-1.64232	0.100523	0.446831
Ice1	226.9675	-4.36888	2.66079	-1.64195	0.100601	0.446831
GlcT	761.7291	-2.37987	1.450205	-1.64106	0.100785	0.447155
ara	224.0285	4.742649	2.889714	1.641217	0.100752	0.447155
Ravus	658.8213	1.921484	1.171227	1.640573	0.100886	0.447355
Rpt2	1338.952	1.530556	0.934617	1.63763	0.101499	0.449766
Sytbeta	1038.658	-2.1617	1.320305	-1.63727	0.101573	0.449766
CG14968	185.4094	-3.19071	1.948937	-1.63716	0.101598	0.449766
Mppe	1734.948	-2.32557	1.421229	-1.63631	0.101775	0.450299
Crag	928.5932	-1.98032	1.210731	-1.63564	0.101914	0.450459
CG16935	1371.265	-1.51387	0.925574	-1.6356	0.101923	0.450459
kek4	2643.414	1.088244	0.665507	1.635211	0.102005	0.450573
EMC8-9	2837.198	-1.28866	0.788411	-1.6345	0.102153	0.45098
sofe	285.2035	4.74687	2.905685	1.633649	0.102333	0.451523
cm	1250.54	1.721343	1.054558	1.632289	0.102619	0.452537
CG6511	92.83817	5.255734	3.221109	1.631654	0.102752	0.452878
Rab8	2371.584	-1.14051	0.700389	-1.62839	0.103442	0.455669
CG11444	1421.768	1.788686	1.098978	1.62759	0.103612	0.455945
Ptp4E	2943.217	-1.12317	0.69017	-1.62739	0.103655	0.455945
CG42489	510.1235	-2.96992	1.825071	-1.62729	0.103675	0.455945
Sap130	812.6738	-2.66398	1.638195	-1.62617	0.103914	0.456745
Srrm234	33631.08	0.693726	0.426816	1.625353	0.104087	0.457057
Meltrin	1418.118	1.653918	1.01761	1.625297	0.104099	0.457057
Vps2	1436.912	-1.9981	1.229794	-1.62475	0.104216	0.457322
MED18	560.5644	-2.50519	1.542835	-1.62375	0.104428	0.457374
CG17278	470.9252	2.811907	1.731912	1.623586	0.104464	0.457374
beat-IV	1512.693	-1.88068	1.158516	-1.62336	0.104513	0.457374
CG30183	241.8088	-4.2553	2.620136	-1.62408	0.10436	0.457374
Pdi	7599.764	-0.8473	0.521637	-1.62431	0.104309	0.457374
Eps-15	4885.641	1.099962	0.677944	1.622496	0.104697	0.457929
CG6123	1812.059	1.242816	0.766472	1.621478	0.104915	0.458618
CG14340	217.6289	4.072199	2.5118	1.621227	0.104969	0.458618
Su(fu)	485.5843	2.844322	1.756091	1.619689	0.105299	0.458811
CG31357	2062.906	-2.08908	1.289601	-1.61994	0.105245	0.458811
PIG-X	1170.741	-2.38799	1.473717	-1.62039	0.105149	0.458811
Ptp36E	1342.21	-1.44054	0.888981	-1.62044	0.105138	0.458811
lncRNA:CR	142.1894	4.684732	2.891656	1.620086	0.105214	0.458811
rasp	206.3255	-4.35765	2.691512	-1.61903	0.10544	0.459177
Su(H)	125.9258	4.243204	2.62233	1.618105	0.10564	0.459798
CG34325	311.3529	3.687943	2.279646	1.61777	0.105712	0.459862
CG5377	675.0553	3.275541	2.026742	1.616161	0.10606	0.461124
lwr	3930.502	-1.16572	0.722126	-1.61429	0.106464	0.46213
CCT2	1405.035	-1.8913	1.171566	-1.61434	0.106454	0.46213
pck	475.9226	2.618757	1.622165	1.614359	0.10645	0.46213

AstC-R2	136.0003	-4.97071	3.079752	-1.614	0.106528	0.462158
Nepl10	704.528	-3.24632	2.011912	-1.61355	0.106625	0.462329
CG43093	391.2353	2.703421	1.676183	1.612843	0.106779	0.462496
dbo	2274.525	1.559854	0.967118	1.612888	0.106769	0.462496
lncRNA:CR	449.0199	2.565934	1.591399	1.612376	0.10688	0.462686
mAcon1	13104.83	0.708821	0.439704	1.612043	0.106953	0.46275
CG8043	487.6947	-2.82819	1.757322	-1.60938	0.107534	0.463981
egg	440.4261	2.833191	1.760059	1.609713	0.10746	0.463981
Nop60B	606.0103	2.562009	1.592153	1.609148	0.107584	0.463981
norpA	16915.57	0.713823	0.44356	1.609305	0.10755	0.463981
cN-IIIB	1256.446	-1.86408	1.157955	-1.6098	0.107441	0.463981
Eno	30156.41	-0.66826	0.415093	-1.60991	0.107417	0.463981
MrgBP	984.4724	-2.46993	1.53532	-1.60874	0.107674	0.464119
Ndfip	2648.77	1.112845	0.692781	1.606344	0.108198	0.46613
Cap-H2	471.2333	-2.13506	1.329618	-1.60577	0.108325	0.466355
l(2)SH083	2826.431	-1.05799	0.658944	-1.60558	0.108367	0.466355
SMC3	989.4937	1.567441	0.977131	1.604125	0.108686	0.46748
Nnp-1	461.5936	2.918855	1.819934	1.603825	0.108753	0.467514
CG9518	78.7764	-6.19933	3.868604	-1.60247	0.109051	0.468548
CG3955	3615.025	-0.97183	0.60683	-1.6015	0.109267	0.469224
kon	370.4755	-2.7414	1.712473	-1.60084	0.109412	0.469594
Art1	1265.735	2.207107	1.379026	1.600483	0.109492	0.469684
KrT95D	7957.892	0.800639	0.501024	1.598005	0.110042	0.471212
CG12075	95.93914	4.673222	2.923632	1.59843	0.109947	0.471212
ste24a	2033.735	-1.82711	1.143294	-1.59811	0.110019	0.471212
Cals	39568.34	0.64173	0.401628	1.597822	0.110083	0.471212
Pomp	3232.537	1.723893	1.07923	1.597336	0.110191	0.471221
Cow	13741.83	0.712183	0.44587	1.597286	0.110202	0.471221
CG11317	5210.328	0.951654	0.596851	1.594458	0.110834	0.473204
CG9410	1465.753	1.754337	1.1003	1.594417	0.110843	0.473204
svr	15276.47	0.864467	0.542012	1.594922	0.11073	0.473204
CG1307	2535.828	-1.23161	0.773199	-1.59287	0.111189	0.47443
Syx4	2692.018	1.2124	0.761884	1.591318	0.111538	0.475063
Fas3	1065.148	1.631031	1.024929	1.59136	0.111529	0.475063
tsu	1095.134	1.951777	1.226324	1.591567	0.111482	0.475063
CG5877	540.0106	2.241357	1.408829	1.590936	0.111624	0.475063
CG14074	358.4236	3.123703	1.963488	1.590895	0.111633	0.475063
CG31436	738.1626	-2.54347	1.59931	-1.59035	0.111755	0.475079
mRpL46	1391.321	-1.42649	0.896847	-1.59057	0.111707	0.475079
mtTFB2	484.6938	3.594355	2.2614	1.589438	0.111961	0.475451
Prosalpha	1310.066	1.712333	1.077196	1.58962	0.11192	0.475451
Srp68	997.0144	-2.17627	1.369505	-1.58909	0.11204	0.475532
Ir51a	159.4092	4.80178	3.022694	1.588576	0.112156	0.475774
CG2201	1964.049	-1.19333	0.751361	-1.58822	0.112237	0.475864
nAChRbet	4314.761	-1.24284	0.782845	-1.58759	0.112378	0.475962
Sil1	859.2222	-2.06902	1.303142	-1.58772	0.112351	0.475962
LTV1	4836.309	0.90402	0.5696	1.587114	0.112487	0.476171
CG7414	3441.374	1.00012	0.630505	1.586222	0.112689	0.476775
wry	10105.83	-0.73938	0.466531	-1.58485	0.113001	0.477591
CG43056	273.6259	3.361332	2.120585	1.585097	0.112944	0.477591

Tsp96F	2990.014	-1.35491	0.855907	-1.58301	0.11342	0.478317
elav	9052.451	0.787604	0.497607	1.582785	0.113471	0.478317
PIG-S	290.5485	-3.47015	2.192354	-1.58284	0.113457	0.478317
PHGPx	5849.002	-0.82176	0.518966	-1.58346	0.113316	0.478317
fry	2405.447	-1.24027	0.783165	-1.58366	0.113271	0.478317
Lsm11	265.0145	-3.02734	1.913184	-1.58236	0.113568	0.478478
CG5969	1028.368	1.948031	1.231472	1.581871	0.113679	0.478692
CG17378	1980.419	1.265575	0.800589	1.580805	0.113923	0.478752
lgs	5240.13	0.947898	0.599645	1.580765	0.113932	0.478752
stops	371.4909	-2.73781	1.731594	-1.58109	0.113857	0.478752
MKP-4	3089.066	1.91535	1.211464	1.581021	0.113873	0.478752
Tnks	1122.783	2.432126	1.539379	1.57994	0.114121	0.479251
Aldh7A1	103.1596	-4.97194	3.147864	-1.57946	0.11423	0.479251
CG5144	180.7383	-4.55753	2.885249	-1.5796	0.114199	0.479251
Dop1R1	19414.91	-0.66057	0.418449	-1.57861	0.114426	0.479572
Tmem63	1683.705	2.051502	1.299512	1.57867	0.114412	0.479572
Cyp49a1	699.2814	2.298754	1.456606	1.578157	0.114529	0.479757
CG7133	606.4555	-2.97343	1.884884	-1.57751	0.114678	0.480127
CG2906	642.9965	3.372478	2.138287	1.577187	0.114753	0.48019
CG9215	320.2362	-3.59108	2.277589	-1.5767	0.114864	0.480406
CG32369	2282.115	1.11731	0.709033	1.575822	0.115067	0.481003
viaf	2158.001	1.259268	0.799298	1.575467	0.115149	0.481095
GluRIIA	162.2427	4.659235	2.959276	1.574451	0.115383	0.481425
a	2518.998	1.068329	0.678524	1.57449	0.115374	0.481425
CG13698	1063.716	-2.38349	1.513958	-1.57435	0.115408	0.481425
scat	450.4966	2.696071	1.713363	1.573555	0.11559	0.481937
Ncoa6	2649.567	1.090133	0.693343	1.572285	0.115884	0.482392
agt	802.4482	-2.61316	1.662268	-1.57205	0.11594	0.482392
RagA-B	1420.986	-2.15298	1.369532	-1.57206	0.115937	0.482392
Alg2	636.7587	2.666717	1.695658	1.572674	0.115794	0.482392
Tpr2	4849.265	-1.03771	0.660345	-1.57147	0.116074	0.482412
CG4293	302.1314	-3.06374	1.949581	-1.57149	0.116069	0.482412
Ugt317A1	385.3954	-3.55761	2.26464	-1.57094	0.116197	0.482412
Sac1	2116.663	-1.15272	0.733644	-1.57122	0.116131	0.482412
eIF5B	1486.285	1.349367	0.85907	1.570731	0.116245	0.482412
CG1824	956.4565	-1.98446	1.264116	-1.56984	0.116452	0.48302
CdGAPr	2214.09	1.220948	0.778559	1.568214	0.116831	0.483843
car	280.3999	2.861307	1.824528	1.568245	0.116824	0.483843
CG4022	470.1736	-2.47326	1.577056	-1.56828	0.116817	0.483843
CG16734	275.2178	-3.91211	2.49578	-1.56749	0.117001	0.484295
S1P	1039.83	2.435525	1.554341	1.566918	0.117134	0.484475
ash1	1265.69	-1.39909	0.892972	-1.56678	0.117165	0.484475
CG11417	477.2987	-2.50164	1.597236	-1.56623	0.117294	0.484511
GatB	365.5693	3.439107	2.195572	1.566383	0.117259	0.484511
EMC3	2448.06	-1.09181	0.69725	-1.56587	0.117378	0.484607
CG16779	8499.432	0.768282	0.490751	1.565522	0.11746	0.484698
slp2	108.7838	-5.45234	3.483751	-1.56508	0.117564	0.484878
dao	1920.911	-1.25343	0.801055	-1.56472	0.117648	0.484971
ohgt	2390.264	-1.71241	1.094761	-1.56419	0.117774	0.485242
Pus1	760.3401	2.426302	1.551669	1.563672	0.117895	0.485491

CG33213	100.8003	5.78296	3.699737	1.563073	0.118035	0.485821
CG11997	312.0247	-3.52563	2.259155	-1.5606	0.118619	0.485901
CG14721	954.4637	2.121769	1.359069	1.561193	0.118478	0.485901
spn-B	67.23171	6.027094	3.862749	1.560312	0.118686	0.485901
CG3631	518.8128	-2.91483	1.868586	-1.55991	0.118781	0.485901
CG5412	355.957	2.294481	1.469625	1.56127	0.11846	0.485901
PTPMT1	322.4637	-3.19153	2.042561	-1.56251	0.118167	0.485901
RNaseP:RN	479.2452	-2.08043	1.332729	-1.56103	0.118517	0.485901
Cyp6a20	1902.673	-1.30597	0.836392	-1.56144	0.11842	0.485901
Prp8	3053.256	1.486386	0.952733	1.560129	0.11873	0.485901
CG14969	1418.647	1.525325	0.977077	1.56111	0.118498	0.485901
Drak	1964.296	1.158061	0.741831	1.561085	0.118504	0.485901
CG6891	203.7443	-3.88235	2.488816	-1.55992	0.118779	0.485901
CG8149	1017.116	-2.24292	1.438558	-1.55914	0.118963	0.486222
CG43394	642.84	-2.44173	1.56615	-1.55907	0.118981	0.486222
CG1620	1766.278	-2.01268	1.29165	-1.55822	0.119181	0.486791
MICU1	1376.206	-1.45934	0.937116	-1.55727	0.119406	0.486879
CG30122	1854.135	1.287448	0.82682	1.557108	0.119445	0.486879
Ptp69D	1950.117	-1.78148	1.143979	-1.55727	0.119406	0.486879
CG4887	3277.813	-1.1259	0.722795	-1.5577	0.119304	0.486879
CG32262	1970.121	1.549924	0.99698	1.554619	0.120037	0.489044
mRpL28	2107.031	-1.2132	0.780579	-1.55424	0.120128	0.489166
CG8032	1637.732	1.696512	1.092356	1.553076	0.120405	0.489893
CAHbeta	113.8404	-6.00579	3.867991	-1.55269	0.120497	0.489893
CG3040	1223.173	1.644841	1.059428	1.552575	0.120525	0.489893
CG10257	684.7073	2.676815	1.724233	1.552467	0.120551	0.489893
unk	3994.335	1.135746	0.73253	1.550442	0.121036	0.491413
CG2278	425.5499	-2.73039	1.761096	-1.55039	0.121047	0.491413
robo3	1423.796	-1.3773	0.88853	-1.55009	0.12112	0.491459
GAPsec	70.48179	5.441931	3.511618	1.549693	0.121215	0.491598
Schip1	2782.061	-1.028	0.663826	-1.54859	0.12148	0.491937
Ipp	4784.746	-1.03361	0.667881	-1.54759	0.12172	0.491937
Gyf	8309.189	-0.83713	0.540937	-1.54756	0.121728	0.491937
CG9795	1633.769	-1.61313	1.041966	-1.54816	0.121583	0.491937
CG42404	2010.858	-1.60843	1.03905	-1.54798	0.121626	0.491937
CG46280	2842.81	-1.01226	0.65398	-1.54785	0.121659	0.491937
nmo	11628.4	0.753801	0.487008	1.54782	0.121666	0.491937
CG10903	199.8217	3.700759	2.39217	1.54703	0.121856	0.492207
MAPk-Ak2	1426.195	1.391709	0.900498	1.545488	0.122228	0.493165
chif	1222.284	-1.41958	0.918466	-1.5456	0.122202	0.493165
sv	430.9798	-2.29984	1.488297	-1.54528	0.122277	0.493165
Tbc1d15-1	404.0663	-2.80472	1.816454	-1.54406	0.122574	0.493615
SLC22A	3961.961	1.308075	0.84703	1.544309	0.122514	0.493615
Ltn1	730.5543	-1.76909	1.145736	-1.54406	0.122573	0.493615
tkv	5249.007	-0.84844	0.549805	-1.54316	0.122791	0.493994
prod	1058.426	-1.65584	1.073001	-1.54318	0.122787	0.493994
CG9853	1394.728	-2.16407	1.403056	-1.5424	0.122977	0.494298
CG5027	2409.903	-1.52388	0.988026	-1.54235	0.122989	0.494298
TfIIS	622.0018	-2.04609	1.330033	-1.53837	0.123957	0.496323
CG10435	1726.114	-1.4558	0.94605	-1.53882	0.123849	0.496323

CG15080	100.1636	-5.66775	3.684182	-1.5384	0.123951	0.496323
Pabp2	769.8756	-1.93991	1.260894	-1.53852	0.123922	0.496323
CG13404	507.358	2.062604	1.340879	1.538248	0.123988	0.496323
Snap25	46482.19	0.637707	0.414466	1.538626	0.123896	0.496323
pain	1346.216	-1.67555	1.088197	-1.53975	0.123621	0.496323
CG9119	325.8469	-3.32993	2.16445	-1.53846	0.123935	0.496323
CG4025	806.7837	-2.3506	1.529634	-1.53671	0.124364	0.497582
CG16865	520.1678	2.373902	1.546699	1.534818	0.124828	0.498941
Orcokinin	188.1804	4.348365	2.833142	1.534821	0.124828	0.498941
CG44247	11072.77	0.889139	0.580009	1.532975	0.125282	0.500504
Syt4	12650.43	0.719383	0.469616	1.531855	0.125558	0.501121
c12.2	225.5692	3.491254	2.279119	1.531843	0.125561	0.501121
CG14657	1589.229	-1.2256	0.800277	-1.53146	0.125655	0.501245
Ciao1	813.981	-1.98672	1.297874	-1.53075	0.125831	0.501697
CG9663	196.7174	-3.54455	2.318025	-1.52913	0.126233	0.503053
CG1983	2675.205	1.244955	0.814455	1.528574	0.12637	0.503348
qkr58E-2	1717.475	1.638822	1.072845	1.527548	0.126625	0.503731
CG3402	2363.168	-1.49609	0.979238	-1.52781	0.12656	0.503731
Taf9	333.0177	-2.65443	1.737844	-1.52743	0.126654	0.503731
Idh3b	7045.214	0.840546	0.550741	1.526209	0.126958	0.504688
CG33298	1257.599	-1.83754	1.204449	-1.52563	0.127102	0.50501
ver	220.6708	-3.28848	2.157484	-1.52422	0.127453	0.506156
CG17270	916.5104	1.925985	1.264264	1.523404	0.127658	0.506547
CG11586	713.6747	-2.20048	1.444526	-1.52332	0.127678	0.506547
CG43103	247.662	-3.88211	2.548893	-1.52306	0.127745	0.50656
CG1662	1007.558	-1.92305	1.262862	-1.52277	0.127815	0.506591
CG33914	149.0754	4.04018	2.654214	1.522176	0.127965	0.506684
CG12177	9507.797	-0.87551	0.575126	-1.52229	0.127936	0.506684
CG3652	2663.897	1.12321	0.739526	1.518823	0.128807	0.509766
CG5037	4141.105	-1.60557	1.057319	-1.51853	0.128881	0.509806
Vta1	1551.551	-1.92475	1.268355	-1.51751	0.129137	0.51048
CG13455	198.5275	-4.06444	2.678643	-1.51735	0.129178	0.51048
Coq6	1164.421	-1.80617	1.190866	-1.51669	0.129346	0.510891
CG43149	548.3306	-2.47263	1.63065	-1.51634	0.129432	0.510981
CG42233	466.6445	2.844288	1.876336	1.515873	0.129551	0.511199
beat-Vc	2841.173	1.157291	0.764052	1.514676	0.129855	0.511892
asRNA:CR4	111.2839	-4.23559	2.796356	-1.51468	0.129853	0.511892
TrpA1	161.342	5.293748	3.495815	1.51431	0.129947	0.512006
CG7156	166.6577	3.773275	2.493453	1.513273	0.13021	0.512544
CG7834	1927.506	-1.17333	0.775358	-1.51327	0.130211	0.512544
bdl	7932.612	-0.76946	0.508909	-1.51198	0.130539	0.513582
Sgf29	1242.624	-1.42162	0.940736	-1.51118	0.130743	0.514132
CG2493	2108.074	1.16084	0.768465	1.510596	0.130891	0.514248
Naa15-16	865.0898	1.949476	1.290566	1.510559	0.130901	0.514248
CG6923	1308.458	1.939878	1.285345	1.509228	0.131241	0.514375
CG33108	590.9614	-3.19894	2.119376	-1.50938	0.131202	0.514375
Sik3	2217.481	-1.26835	0.840352	-1.5093	0.131222	0.514375
Pi3K59F	692.9534	-2.08491	1.381375	-1.5093	0.131222	0.514375
sturkopf	820.8208	1.656497	1.097777	1.508956	0.13131	0.514375
CG14613	463.4021	2.49507	1.65354	1.508926	0.131318	0.514375

Sar1	4056.974	-1.02464	0.679263	-1.50846	0.131436	0.514386
CG2277	3599.349	-1.08065	0.716417	-1.50841	0.131449	0.514386
H15	267.0048	-3.41275	2.264185	-1.50727	0.13174	0.514788
Blm	228.718	-3.4901	2.315545	-1.50725	0.131747	0.514788
Gycalpha9	9384.103	-0.74657	0.49548	-1.50676	0.131872	0.514788
RpL15	56965.82	0.612429	0.406186	1.507757	0.131617	0.514788
CG6843	541.8271	-2.13945	1.419673	-1.507	0.13181	0.514788
CG3862	443.185	3.214941	2.135395	1.505549	0.132183	0.515
Rsf1	4152.281	-0.97265	0.645982	-1.5057	0.132145	0.515
Fer2	323.1204	3.587492	2.38207	1.50604	0.132057	0.515
Usp5	357.6421	2.730318	1.812984	1.50598	0.132072	0.515
S6k	1984.071	-1.46269	0.97178	-1.50516	0.132282	0.515135
CG17493	6185.989	0.867454	0.576425	1.504887	0.132353	0.515163
Arp3	1097.833	1.71834	1.14215	1.504478	0.132458	0.515323
rdgB	10751.56	-0.73736	0.49044	-1.50347	0.132717	0.516078
PPO2	379.4702	-2.39525	1.594026	-1.50264	0.132931	0.51641
sxc	8421.767	0.725258	0.482615	1.502767	0.132899	0.51641
Myo31DF	77.39112	5.0096	3.334554	1.50233	0.133012	0.516475
Sugb	295.6368	-3.10528	2.068078	-1.50153	0.133219	0.51703
CG31759	168.6084	-4.22117	2.814344	-1.49988	0.133647	0.518187
CG41378	2584.114	1.022049	0.68133	1.500079	0.133594	0.518187
CG10638	346.6404	3.093441	2.063594	1.499055	0.133859	0.518762
Sk2	367.4236	-3.15587	2.105609	-1.49879	0.133928	0.518777
CG14562	3875.627	0.901187	0.601747	1.497617	0.134233	0.519456
CG34423	63.81692	4.548812	3.037198	1.4977	0.134211	0.519456
CG14964	92.71105	4.72995	3.162028	1.495859	0.13469	0.520975
CG12299	494.2367	2.466943	1.649917	1.495192	0.134864	0.521029
cu	1487.269	-1.7314	1.157891	-1.49531	0.134834	0.521029
CG43845	808.4635	1.674335	1.119911	1.49506	0.134899	0.521029
sprt	621.9213	1.914588	1.280909	1.49471	0.13499	0.52113
CG12948	359.0289	2.664384	1.784445	1.493116	0.135407	0.521382
Blos1	2028.545	1.392636	0.932827	1.49292	0.135458	0.521382
eIF4G2	3046.393	1.054188	0.705713	1.493792	0.13523	0.521382
Gpat4	2991.157	1.193015	0.798854	1.493409	0.13533	0.521382
DIP2	4072.507	-0.87561	0.586126	-1.4939	0.135201	0.521382
CG7556	518.1243	-2.36016	1.581108	-1.49272	0.13551	0.521382
CG11658	3394.529	-1.37085	0.917888	-1.49348	0.135311	0.521382
ics	1237.579	1.3306	0.892565	1.490761	0.136024	0.52286
CG1309	1007.303	2.074193	1.39118	1.49096	0.135972	0.52286
stj	11283.22	0.692699	0.46487	1.490091	0.1362	0.523286
Nab2	885.7175	1.721275	1.155553	1.489568	0.136338	0.523563
Nop56	1387.417	-1.98567	1.333789	-1.48875	0.136554	0.523753
Pgant9	5272.631	0.898687	0.603898	1.488142	0.136713	0.523753
Egm	1172.568	-1.80612	1.213586	-1.48825	0.136685	0.523753
CG12301	510.0574	-2.78599	1.871482	-1.48865	0.136579	0.523753
CG32099	229.3789	-4.03824	2.713406	-1.48826	0.136683	0.523753
CG10126	2401.301	1.875192	1.260604	1.487535	0.136874	0.52405
E2f1	2507.578	-1.12183	0.754619	-1.48661	0.137118	0.52405
CG15514	1267.743	-1.50493	1.011995	-1.48709	0.136991	0.52405
CG18004	778.8288	2.117208	1.424017	1.486785	0.137072	0.52405

CG34384	8904.526	0.698692	0.469924	1.486817	0.137063	0.52405
RnrL	90.46017	-5.7317	3.861331	-1.48438	0.137707	0.524306
beta4GalT	174.9152	3.885878	2.61692	1.484905	0.137569	0.524306
Rbm13	433.7091	3.390923	2.283948	1.484677	0.13763	0.524306
CG8858	693.9719	-2.1452	1.444901	-1.48467	0.137632	0.524306
CG33639	847.7238	-2.14998	1.448229	-1.48456	0.137661	0.524306
AGO3	291.7059	-2.62473	1.766997	-1.48542	0.137433	0.524306
Vlet	1240.775	2.091519	1.407759	1.485708	0.137356	0.524306
msl-3	1484.693	1.519671	1.023706	1.484479	0.137682	0.524306
CG30197	14331.58	-0.76572	0.516017	-1.4839	0.137836	0.524539
CG42671	1943.013	1.238463	0.834735	1.48366	0.137899	0.524539
CG5815	479.3659	-2.58774	1.744784	-1.48313	0.13804	0.524827
CG2617	249.3189	3.62788	2.448565	1.481635	0.138437	0.526024
CG12945	638.6133	-2.35368	1.589026	-1.48121	0.138552	0.526024
Bem46	263.3584	3.455208	2.332691	1.481211	0.13855	0.526024
MED17	411.1651	-2.4782	1.675357	-1.4792	0.139086	0.527803
lncRNA:CR	319.4573	-2.68394	1.815029	-1.47873	0.139212	0.528032
rl	8425.342	0.767698	0.519372	1.478127	0.139374	0.528396
eIF3d1	2467.067	-1.52207	1.030362	-1.47722	0.139617	0.529069
CG5262	1612.111	1.269273	0.860136	1.475665	0.140034	0.529718
CG10621	147.4287	-4.06991	2.757304	-1.47605	0.139931	0.529718
CG30089	1046.761	-2.01552	1.365905	-1.4756	0.140052	0.529718
l(1)G0004	375.6924	2.236484	1.515035	1.476192	0.139892	0.529718
CG32815	2022.526	1.857513	1.259552	1.474741	0.140282	0.529833
Corp	129.5101	3.749048	2.542589	1.4745	0.140347	0.529833
CG30082	89.63715	5.653949	3.83289	1.475114	0.140182	0.529833
CG42340	2688.921	1.088605	0.738081	1.474912	0.140236	0.529833
CG32809	9670.938	0.856475	0.581073	1.473955	0.140494	0.530138
Idgf6	264.168	3.647011	2.475235	1.4734	0.140643	0.530453
cnn	2298.075	-1.04858	0.711883	-1.47297	0.140758	0.530636
CG13366	2365.138	1.341181	0.910882	1.472399	0.140913	0.530639
eIF3g1	3724.851	1.324595	0.899716	1.472236	0.140957	0.530639
dpr11	3107.433	1.120096	0.760616	1.472617	0.140854	0.530639
CG14757	7835.487	0.753993	0.512358	1.471615	0.141125	0.531021
lid	3388.855	-1.09555	0.745564	-1.46943	0.141717	0.531754
CG11257	95.99311	5.537329	3.768123	1.469519	0.141692	0.531754
l(2)k10201	136.4441	-4.23911	2.884029	-1.46986	0.141601	0.531754
CG9527	295.3469	-3.10241	2.110163	-1.47022	0.141502	0.531754
DIP-beta	997.0238	1.966425	1.337773	1.469924	0.141582	0.531754
mus302	680.6277	3.202903	2.178081	1.470516	0.141422	0.531754
Tsp3A	457.5477	-2.61718	1.781857	-1.46879	0.141889	0.531809
eco	313.4622	3.58151	2.439339	1.46823	0.142042	0.531809
TfAP-2	739.5227	-2.61114	1.778357	-1.46829	0.142027	0.531809
dind	602.2724	2.419175	1.647083	1.468763	0.141897	0.531809
CG7739	3948.728	-0.88267	0.601215	-1.46815	0.142063	0.531809
CG11414	488.5286	-1.99979	1.362475	-1.46776	0.14217	0.53196
gukh	282.6897	2.771521	1.889414	1.466868	0.142412	0.53237
Secp43	1895.907	-1.977	1.347743	-1.4669	0.142403	0.53237
CG3726	1540.287	1.268114	0.865776	1.464713	0.142999	0.532437
Wdr82	955.8013	-2.10251	1.434909	-1.46526	0.142851	0.532437

CG12811	487.9474	3.259112	2.224278	1.465245	0.142854	0.532437
Sdc	13131.01	-0.66106	0.450876	-1.46617	0.142602	0.532437
Gpo1	3291.558	1.074898	0.733281	1.465875	0.142682	0.532437
del	622.6705	2.337066	1.594555	1.465653	0.142743	0.532437
CG43110	849.228	-2.16275	1.475019	-1.46625	0.142579	0.532437
Vps60	2588.738	1.088374	0.743029	1.464779	0.142981	0.532437
SH3PX1	1189.57	-2.24203	1.530798	-1.46461	0.143027	0.532437
CG8260	176.6705	4.368517	2.983827	1.464065	0.143176	0.532746
CG5846	418.7754	-3.15695	2.159803	-1.46169	0.143827	0.534077
CG9098	2649.879	1.821242	1.245406	1.462368	0.14364	0.534077
CG9917	6728.721	-0.83446	0.570785	-1.46195	0.143756	0.534077
CG32100	85.13246	5.562411	3.80585	1.461543	0.143867	0.534077
Uev1A	5614.809	0.917996	0.627908	1.461991	0.143744	0.534077
CG9689	16514.27	-0.7429	0.50843	-1.46116	0.143971	0.534217
CG13994	985.8466	2.102227	1.439369	1.46052	0.144147	0.534623
ndl	333.4508	3.910843	2.678615	1.460024	0.144283	0.534882
Grip91	386.4367	-2.43048	1.665483	-1.45932	0.144476	0.535349
AsnRS-m	851.0826	-2.5743	1.765184	-1.45838	0.144737	0.536068
Cul1	2065.364	-1.40423	0.963092	-1.45804	0.144829	0.536161
SLC5A11	402.0603	-2.55712	1.754596	-1.45739	0.14501	0.536337
crb	1167.354	2.32683	1.596366	1.45758	0.144956	0.536337
CG8366	469.3196	2.399277	1.647831	1.456021	0.145387	0.537482
Cyp4p2	172.724	-2.94534	2.023283	-1.45572	0.14547	0.537542
CG5726	516.3498	2.857866	1.964595	1.454684	0.145757	0.538354
CG17776	1747.101	1.144914	0.787298	1.454231	0.145882	0.538377
CG4393	1756.862	-1.20564	0.829088	-1.45418	0.145897	0.538377
Spn27A	476.9826	2.430316	1.672549	1.453061	0.146207	0.539024
CG14207	4177.18	-0.84892	0.584133	-1.4533	0.146141	0.539024
larp	5377.063	0.790734	0.544494	1.452235	0.146436	0.539622
Eip75B	19683.61	0.651031	0.448525	1.451492	0.146643	0.540136
CG2147	759.3366	-2.27565	1.568526	-1.45082	0.14683	0.540576
mRpL9	2271.332	1.148933	0.792516	1.449728	0.147134	0.540706
Fancm	632.6024	2.847778	1.964681	1.449486	0.147202	0.540706
CG33969	1401.714	1.418472	0.978466	1.44969	0.147145	0.540706
lncRNA:CR	1181.27	1.330603	0.917727	1.449891	0.147089	0.540706
Klp10A	3786.374	-1.10907	0.764651	-1.45043	0.146939	0.540706
Clamp	2046.479	1.436708	0.991718	1.448706	0.14742	0.541258
CG4119	996.4364	1.403252	0.968973	1.448185	0.147565	0.54132
CG9581	681.8518	2.656938	1.835001	1.447922	0.147639	0.54132
CG3281	626.9015	-2.35927	1.629366	-1.44797	0.147625	0.54132
CG2680	2538.968	-1.10061	0.760629	-1.44697	0.147905	0.542049
CG10681	85.30768	4.940443	3.416536	1.446039	0.148166	0.542758
CG34409	561.4841	-2.71346	1.877209	-1.44548	0.148324	0.543089
CG6928	341.1599	-3.59336	2.487063	-1.44482	0.148509	0.543516
opm	415.4123	-2.54614	1.763724	-1.44362	0.148846	0.544503
net	1216.872	-2.13825	1.482851	-1.44198	0.149307	0.54585
CG4186	350.6928	-2.55927	1.775016	-1.44183	0.14935	0.54585
abo	590.9693	2.764073	1.918943	1.440414	0.14975	0.546378
CG13601	1066.387	-1.57477	1.093502	-1.44011	0.149835	0.546378
Noa36	1222.371	2.035302	1.412958	1.440455	0.149739	0.546378

CG34303	267.6805	3.268337	2.269177	1.440318	0.149778	0.546378
Rgk2	1128.248	-1.39155	0.965669	-1.44103	0.149577	0.546378
gcl	2004.595	-1.18827	0.825722	-1.43907	0.150131	0.546748
CG7839	638.3545	-2.0667	1.436034	-1.43917	0.150102	0.546748
GABA-B-R3	2243.987	1.581493	1.098996	1.439033	0.150141	0.546748
CG42258	2990.836	-0.97541	0.677953	-1.43876	0.15022	0.546786
Dhpr	1189.968	1.596967	1.110198	1.438453	0.150306	0.546851
Pdss2	383.7481	-2.58997	1.801735	-1.43748	0.15058	0.546859
CG4839	66.4165	4.86397	3.382187	1.438114	0.150402	0.546859
CG14715	1543.92	-1.17269	0.815693	-1.43766	0.15053	0.546859
CG17508	13780.26	-0.97429	0.677599	-1.43786	0.150474	0.546859
CG4074	908.7542	-2.09687	1.45984	-1.43637	0.150897	0.547019
Ttc19	950.0355	1.981439	1.379269	1.436587	0.150835	0.547019
Ire1	755.3975	-1.63423	1.137326	-1.43691	0.150744	0.547019
Scamp	2816.174	1.230601	0.856605	1.436602	0.150831	0.547019
CG9067	720.9948	1.579846	1.100308	1.435821	0.151053	0.547338
side-VIII	10155.44	-0.66366	0.462733	-1.43421	0.151512	0.548752
CG9662	1731.9	1.264676	0.88231	1.43337	0.151752	0.549374
CG8311	699.5235	1.80666	1.260921	1.43281	0.151912	0.549706
row	1426.812	-1.29975	0.907403	-1.43239	0.152033	0.549896
stan	587.1955	-2.10143	1.4683	-1.4312	0.152374	0.550879
sced	636.6541	-2.38636	1.668035	-1.43064	0.152533	0.551095
CG43759	1121.059	-1.44772	1.01203	-1.43051	0.152571	0.551095
CG9135	939.0272	-2.15387	1.506222	-1.42998	0.152722	0.551392
PIG-G	1310.516	-1.91269	1.338446	-1.42904	0.152993	0.552125
neur	143.3288	-4.64057	3.249462	-1.4281	0.153262	0.552596
lncRNA:CR	75.72149	5.476654	3.834851	1.428127	0.153255	0.552596
CG12825	135.8672	4.753607	3.32993	1.42754	0.153424	0.55291
CG13484	295.0866	-2.78844	1.953615	-1.42732	0.153487	0.55291
emb	4030.954	0.866603	0.607376	1.426798	0.153638	0.55296
CG11858	720.8578	2.360572	1.654183	1.427032	0.153571	0.55296
Galphao	14743.61	0.691287	0.485204	1.424734	0.154234	0.554856
shams	211.9121	-3.89967	2.738411	-1.42406	0.154428	0.554914
casp	2290.684	1.265714	0.889092	1.423602	0.154562	0.554914
CG31224	5098.122	-0.88461	0.621647	-1.42301	0.154734	0.554914
chinmo	10652.93	0.670513	0.470912	1.423861	0.154487	0.554914
CG8924	1306.821	-2.01994	1.419474	-1.42302	0.15473	0.554914
mRpL2	1256.706	-1.69694	1.192172	-1.4234	0.15462	0.554914
MED24	1173.822	-2.30696	1.620456	-1.42365	0.154548	0.554914
ssp3	526.6432	-1.85002	1.300377	-1.42268	0.154829	0.555007
fl(2)d	5139.727	0.824726	0.580071	1.421768	0.155094	0.555706
PIG-U	442.2458	3.078142	2.16579	1.421256	0.155242	0.55599
Dyb	5542.19	0.807558	0.568401	1.420754	0.155388	0.556017
RabX6	349.4185	-2.51083	1.767242	-1.42076	0.155386	0.556017
poe	8682.685	-0.91595	0.645357	-1.4193	0.155813	0.556498
CG31706	20767.01	-0.64998	0.458122	-1.41879	0.15596	0.556498
Rfx	545.0984	-2.78006	1.959311	-1.4189	0.155929	0.556498
CG13840	1637.847	2.231317	1.57317	1.418357	0.156087	0.556498
Hrs	567.8364	2.476526	1.744571	1.419562	0.155735	0.556498
sau	1907.855	-1.38204	0.974538	-1.41815	0.156147	0.556498

AhcyL1	2669.543	0.956273	0.674128	1.418532	0.156035	0.556498
CG1640	9147.362	0.856542	0.60347	1.419363	0.155793	0.556498
CG15435	886.6881	2.90564	2.047908	1.418833	0.155948	0.556498
CG33090	4320.894	-0.97237	0.686446	-1.41653	0.15662	0.557097
EndoU	455.5697	3.535632	2.495636	1.416726	0.156563	0.557097
dco	2173.095	-1.06734	0.753374	-1.41675	0.156556	0.557097
CG1910	1824.538	-1.41271	0.997327	-1.4165	0.15663	0.557097
cora	7416.278	-0.89297	0.630555	-1.41617	0.156727	0.557097
CG12863	279.1235	-3.25809	2.300667	-1.41615	0.156731	0.557097
mura	11174.01	-0.77504	0.54757	-1.41542	0.156946	0.557119
HP1b	1178.021	-1.67862	1.185853	-1.41554	0.156911	0.557119
Fdx2	1702.088	-1.18309	0.835801	-1.41552	0.156916	0.557119
CG7706	230.5349	3.446454	2.435828	1.4149	0.157098	0.557132
CR42875	665.5368	-1.67793	1.185774	-1.41505	0.157054	0.557132
wupA	499.3521	2.167189	1.531926	1.414682	0.157162	0.557132
ND-PDSW	10338.96	0.699514	0.494545	1.41446	0.157227	0.557132
mRpS9	578.0113	2.266382	1.603825	1.41311	0.157623	0.558044
CG9919	3635.577	1.45088	1.026602	1.413283	0.157572	0.558044
CHOp24	3808.348	0.897097	0.63534	1.411994	0.157952	0.55896
fs(2)ltoPP4	1274.371	-1.83335	1.29906	-1.41129	0.15816	0.558974
Sp7	2108.345	-1.26463	0.895859	-1.41164	0.158056	0.558974
CG31007	192.5395	4.021485	2.849548	1.411271	0.158165	0.558974
CG1737	515.1454	-1.81392	1.286357	-1.41012	0.158504	0.559926
spin	1476.875	-1.90905	1.355072	-1.40882	0.158888	0.561036
Fmr1	1734.328	1.285964	0.914426	1.406308	0.159633	0.56126
CG6912	4773.009	0.998583	0.709117	1.408206	0.15907	0.56126
olf186-M	204.5306	-3.21435	2.283744	-1.40749	0.159281	0.56126
cyp33	368.0329	-3.03065	2.154289	-1.4068	0.159487	0.56126
Pura	122.1991	3.487473	2.478947	1.406836	0.159476	0.56126
crc	2491.622	-1.01867	0.724338	-1.40634	0.159622	0.56126
CG42402	20993.25	0.581175	0.413294	1.406201	0.159665	0.56126
Patronin	4388.306	0.948021	0.673405	1.407802	0.15919	0.56126
CG16952	3024.201	1.570241	1.11686	1.405943	0.159741	0.56126
CG32537	189.2658	3.224629	2.293842	1.405777	0.159791	0.56126
CG7990	4761.94	-0.97	0.688879	-1.40808	0.159106	0.56126
LanA	65.37706	5.003884	3.555512	1.40736	0.159321	0.56126
CG4467	10884.24	-0.6412	0.456271	-1.40531	0.159928	0.561496
CG7685	476.9374	-2.26559	1.613458	-1.40418	0.160264	0.562432
CG11975	2229.322	1.82328	1.299585	1.402971	0.160626	0.563453
Bdbt	164.9141	3.8195	2.723161	1.402598	0.160737	0.563597
Rpb3	578.2141	-2.46879	1.760861	-1.40204	0.160905	0.563739
asRNA:CR4	137.7469	4.049491	2.888384	1.401992	0.160918	0.563739
Mlp84B	629.2232	2.410036	1.720531	1.400751	0.161289	0.564299
CG3907	6207.94	0.735154	0.524816	1.400784	0.161279	0.564299
Fcp1	510.4682	2.37494	1.695061	1.401094	0.161186	0.564299
fog	3384.284	-0.95797	0.684324	-1.39988	0.161548	0.56496
Oga	254.6163	-3.6268	2.591705	-1.39939	0.161697	0.564974
CG17111	697.7263	-2.78166	1.988085	-1.39917	0.161763	0.564974
CG31098	703.8831	-2.44987	1.750782	-1.3993	0.161722	0.564974
CaBP1	2856.988	1.051452	0.751863	1.398463	0.161974	0.565465

mtsh	144.4536	-4.67689	3.345237	-1.39807	0.162091	0.565626
CG16743	20626.16	-0.7323	0.524382	-1.3965	0.162563	0.567029
Hmt4-20	2260.217	1.025061	0.734512	1.395566	0.162845	0.567676
CG33144	4042.335	0.809956	0.580538	1.395181	0.162961	0.567676
Nep6	680.8735	2.440836	1.749308	1.395315	0.162921	0.567676
CG9992	1479.961	-1.841	1.31977	-1.39494	0.163035	0.567686
CG6695	1416.349	-1.17742	0.844386	-1.39441	0.163195	0.567849
Rab19	2380.559	1.049778	0.7529	1.394313	0.163223	0.567849
ph-p	2078.952	1.453625	1.043371	1.3932	0.163559	0.568035
CG5062	225.5226	-3.35157	2.405403	-1.39335	0.163513	0.568035
MED6	492.2057	2.794352	2.005311	1.393476	0.163476	0.568035
Lsm10	93.98602	-5.20251	3.733256	-1.39356	0.163451	0.568035
Mpp6	134.5965	-3.69067	2.650413	-1.39249	0.163774	0.568196
Flo1	1776.894	-1.42958	1.026657	-1.39246	0.163784	0.568196
oxt	373.7279	2.761396	1.983272	1.392344	0.163818	0.568196
CG15236	2334.04	1.449627	1.042304	1.39079	0.164289	0.569337
CG8004	1332.548	-1.958	1.407604	-1.39102	0.16422	0.569337
CG30349	896.1089	-2.41965	1.740235	-1.39041	0.164403	0.569487
numb	1447.398	1.189196	0.856054	1.38916	0.164784	0.570337
Indy	930.7233	-1.59785	1.150245	-1.38914	0.164791	0.570337
Kat60	471.3723	2.063298	1.486599	1.387931	0.165158	0.570606
danr	385.939	-2.19373	1.580419	-1.38807	0.165117	0.570606
CG6051	1030.294	1.686923	1.21568	1.387637	0.165248	0.570606
lncRNA:CR	429.5792	2.812981	2.0262	1.388303	0.165045	0.570606
lncRNA:CR	527.9051	-1.8386	1.325356	-1.38725	0.165366	0.570606
CG32022	198.6391	3.230019	2.328263	1.387309	0.165348	0.570606
Snmp2	134.0143	-3.23287	2.328774	-1.38823	0.165067	0.570606
CG4341	1318.881	1.392904	1.004314	1.38692	0.165466	0.570705
alphaTub8	7799.983	0.758284	0.546862	1.386608	0.165561	0.570788
Cmpk	8147.516	0.693947	0.500694	1.385972	0.165756	0.571213
Dif	1714.218	-1.12648	0.813135	-1.38536	0.165943	0.571613
blp	776.214	-1.76901	1.279374	-1.38271	0.166753	0.573348
Smg6	1978.47	1.586231	1.146623	1.383393	0.166544	0.573348
wun	5166.309	0.791715	0.572649	1.382547	0.166804	0.573348
sm	40390.64	0.529766	0.383134	1.382716	0.166752	0.573348
betaggt-II	2424.212	-0.96168	0.695464	-1.38279	0.166731	0.573348
lncRNA:CR	150.9703	-4.41804	3.196772	-1.38203	0.166962	0.573645
Cdc5	532.1473	-2.79934	2.026766	-1.38118	0.167223	0.574296
Jheh3	1234.144	-1.75489	1.270898	-1.38083	0.167333	0.574427
CG4908	944.5369	-1.80033	1.305028	-1.37953	0.167731	0.57555
Nle	332.7228	3.025846	2.194152	1.37905	0.167879	0.575811
CG4291	323.7477	-2.59931	1.888236	-1.37658	0.168641	0.57608
PolQ	192.5967	-4.04653	2.938725	-1.37697	0.168522	0.57608
CG4562	1133.809	-1.59052	1.155103	-1.37695	0.168529	0.57608
nAChRalpł	6742.441	0.906668	0.657725	1.378491	0.168052	0.57608
CG31111	211.6566	3.244367	2.356604	1.376713	0.168601	0.57608
CG12822	3477.567	1.061411	0.770889	1.376867	0.168553	0.57608
HIP	139.4076	-3.10271	2.254101	-1.37647	0.168675	0.57608
Syt12	2781.171	1.794938	1.302852	1.377699	0.168296	0.57608
RabX1	1178.277	1.406497	1.021807	1.37648	0.168673	0.57608

Syx13	1290.677	-1.67776	1.217292	-1.37827	0.168119	0.57608
Drep4	1416.477	1.772632	1.289259	1.374923	0.169155	0.577268
CG2258	4010.645	0.827188	0.601743	1.374654	0.169239	0.577268
CG42399	626.6748	-1.96634	1.430243	-1.37483	0.169183	0.577268
tey	4916.963	0.821306	0.597582	1.374383	0.169323	0.577309
CG9727	3608.218	-0.86064	0.626854	-1.37295	0.169767	0.578088
trsn	1055.753	1.349373	0.982499	1.373409	0.169625	0.578088
Fbxl4	1077.08	-2.13466	1.554735	-1.37301	0.16975	0.578088
Vps26	538.6747	-2.27747	1.662151	-1.3702	0.170626	0.580765
Trh	347.3193	-3.06634	2.238285	-1.36995	0.170702	0.580777
cac	22035.36	0.620511	0.453051	1.369628	0.170803	0.580875
CG1814	2844.125	0.984259	0.718783	1.36934	0.170893	0.580935
Nxf3	813.3003	1.496197	1.093052	1.368825	0.171054	0.581236
CG8414	79.93224	-4.78623	3.497368	-1.36852	0.171148	0.581309
CG17734	8854.647	0.667658	0.488037	1.368048	0.171297	0.58157
CG14210	188.1256	3.205454	2.345152	1.366843	0.171674	0.582604
CG1847	1477.831	1.472543	1.07754	1.366579	0.171757	0.582639
Mst36Fa	163.1601	3.750496	2.745945	1.365831	0.171992	0.582697
Graf	4790.035	0.782808	0.572967	1.366237	0.171865	0.582697
CDC50	6696.341	0.812796	0.595056	1.365915	0.171966	0.582697
Aduk	936.2169	1.754612	1.285759	1.364651	0.172363	0.582723
GlyT	1336.543	1.855734	1.359699	1.364813	0.172312	0.582723
Neto	331.2866	-2.28864	1.676926	-1.36478	0.172321	0.582723
TSG101	3934.603	-0.97221	0.7124	-1.3647	0.172347	0.582723
CG7504	476.1169	-2.59219	1.89908	-1.36497	0.172262	0.582723
Mulk	1018.904	1.469204	1.077608	1.363394	0.172758	0.583814
CG7985	1154.554	1.497845	1.099636	1.362127	0.173158	0.584562
CG30109	680.9177	1.88698	1.385003	1.362437	0.17306	0.584562
Mvb12	905.4134	1.717507	1.261019	1.361999	0.173198	0.584562
CG5910	410.4616	-3.40019	2.497574	-1.3614	0.173388	0.584709
E(spl)mbet	624.3668	2.038304	1.497165	1.361442	0.173374	0.584709
dtn	5382.229	0.76527	0.562428	1.360654	0.173623	0.585258
Tor	159.3771	3.486909	2.563978	1.359961	0.173842	0.585751
Arp2	2545.703	-1.32186	0.972354	-1.35944	0.174007	0.586059
hts	8684.308	-0.6596	0.485366	-1.35898	0.174153	0.586305
MED4	808.4909	-2.17159	1.598338	-1.35865	0.174257	0.586408
CG6523	1707.101	-1.06243	0.782572	-1.35761	0.174587	0.586464
Ugt35B1	56100.52	-0.70774	0.521306	-1.35763	0.174582	0.586464
RpS15Ab	1303.374	-1.22323	0.900706	-1.35808	0.174438	0.586464
CG17765	5949.04	0.714734	0.526511	1.357491	0.174625	0.586464
Sam-S	12922.61	0.786496	0.579393	1.357449	0.174639	0.586464
CG5953	824.3307	1.662448	1.225523	1.356521	0.174933	0.587209
HINT1	5762.639	-0.86699	0.639329	-1.35609	0.175071	0.587425
ear	728.4163	2.05076	1.512523	1.355854	0.175146	0.587429
Tgi	1078.247	1.326431	0.978618	1.355412	0.175286	0.587655
Ahcy	335.3047	-3.06991	2.26569	-1.35496	0.175432	0.587897
HDAC11	703.3393	-2.14932	1.588435	-1.3531	0.176022	0.589385
CG17221	298.5644	-2.40258	1.775312	-1.35333	0.17595	0.589385
Tao	3207.998	0.887727	0.656252	1.352724	0.176144	0.589546
CG30054	195.9242	-3.8421	2.842682	-1.35158	0.176511	0.590529

dome	988.8321	1.753712	1.297864	1.351229	0.176622	0.590654
CG17271	1086.277	-1.65532	1.225967	-1.35022	0.176946	0.591243
CG7742	126.319	-3.96479	2.936004	-1.3504	0.176887	0.591243
Ssk	2686.303	-0.97905	0.725234	-1.34998	0.177023	0.591254
Ekar	396.7464	-2.68243	1.988679	-1.34885	0.177385	0.591996
PPP1R15	3825.392	-0.89588	0.664192	-1.34883	0.177392	0.591996
CG12237	3719.235	0.817661	0.606409	1.348366	0.177541	0.592245
CG10055	1407.341	-1.44494	1.072699	-1.34701	0.177977	0.592629
bai	3431.836	-0.83572	0.620496	-1.34686	0.178025	0.592629
jus	4824.041	-0.76353	0.566803	-1.34709	0.177952	0.592629
spri	4384.425	-0.87249	0.647383	-1.34772	0.17775	0.592629
Vps15	689.0818	-2.17323	1.613081	-1.34726	0.177898	0.592629
CG15803	791.8939	1.63957	1.217853	1.346278	0.178213	0.593008
Thd1	15964.79	0.647561	0.481281	1.345495	0.178465	0.593603
CG13255	824.714	-1.40358	1.0434	-1.3452	0.17856	0.59367
lncRNA:no	330092.2	0.492586	0.366544	1.343866	0.178992	0.594633
Taf4	3211.838	-1.0288	0.765564	-1.34385	0.178997	0.594633
CG12975	2659.005	1.040721	0.774785	1.343238	0.179195	0.595043
Hrd3	879.8204	-1.40405	1.045483	-1.34297	0.179283	0.595088
CG6041	225.8876	3.363433	2.505113	1.342627	0.179393	0.595207
CG5044	416.0274	-2.39159	1.783277	-1.34112	0.179881	0.595833
Miro	1612.178	1.549568	1.154937	1.341691	0.179696	0.595833
Cul5	303.9142	-3.19433	2.382237	-1.34089	0.179955	0.595833
CG17002	244.9541	3.344465	2.493805	1.341109	0.179885	0.595833
otk	185.7193	3.693324	2.754692	1.340739	0.180005	0.595833
Naa35	277.4797	-2.5511	1.903453	-1.34025	0.180164	0.595833
gbb	1525.772	1.954152	1.458088	1.340215	0.180175	0.595833
e(y)2	233.2162	-3.01689	2.250902	-1.3403	0.180148	0.595833
nAChRalpł	11719.16	0.614728	0.459341	1.338282	0.180804	0.597667
emc	11060.04	-0.74562	0.557243	-1.33805	0.180879	0.597669
mts	11609.73	-0.60258	0.450834	-1.33659	0.181355	0.598255
Ptp61F	2819.204	-0.95708	0.715933	-1.33683	0.181278	0.598255
byn	138.1799	-4.0686	3.043739	-1.33671	0.181316	0.598255
papi	816.9035	1.926013	1.440417	1.337122	0.181183	0.598255
Lgr1	184.6163	3.386089	2.53514	1.335661	0.18166	0.598923
Myo81F	2732.217	-0.89085	0.667041	-1.33552	0.181707	0.598923
DAAM	2460.085	-0.96267	0.721347	-1.33455	0.182023	0.599474
nclb	662.4333	2.511167	1.881396	1.334736	0.181963	0.599474
CG42663	201.2772	2.8932	2.168522	1.334181	0.182145	0.599628
Nrx-1	16165.16	0.676062	0.506913	1.333686	0.182307	0.599916
CycC	131.1919	-3.12235	2.343835	-1.33216	0.182809	0.601322
rempA	190.8584	3.119382	2.3421	1.331874	0.182902	0.601349
mthl9	265.7409	2.574447	1.93324	1.331675	0.182967	0.601349
esn	690.8467	-2.1384	1.606135	-1.33139	0.183059	0.601406
AMPKalph	1860.454	-1.2477	0.938759	-1.3291	0.183815	0.601414
bi	1418.794	-1.31219	0.98626	-1.33047	0.183365	0.601414
SK	8632.417	-0.80876	0.60862	-1.32884	0.183902	0.601414
CG9175	829.05	-1.92216	1.446155	-1.32916	0.183797	0.601414
CG6180	6922.196	-0.69146	0.520597	-1.3282	0.184111	0.601414
CG10947	1187.334	-1.50166	1.128167	-1.33106	0.183169	0.601414

mAChR-B	763.5099	1.442113	1.085665	1.328323	0.184072	0.601414
Osbp	669.4488	-1.52506	1.147762	-1.32873	0.183938	0.601414
eIF3k	3338.833	0.818951	0.616382	1.328642	0.183966	0.601414
MESK2	12982.12	0.627297	0.472067	1.328831	0.183904	0.601414
CG14130	898.7807	-2.17307	1.635776	-1.32847	0.184024	0.601414
CG12038	3333.044	-0.82938	0.623226	-1.33079	0.183259	0.601414
CG11811	1004.605	2.005983	1.509553	1.328859	0.183895	0.601414
Naprt	1589.88	1.515692	1.140172	1.329354	0.183731	0.601414
TBC1D16	2141.427	-1.01471	0.764307	-1.32762	0.184304	0.601798
lncRNA:let	505.3404	-1.73865	1.309952	-1.32726	0.184421	0.601938
wtrw	16038.2	-0.78659	0.592758	-1.32699	0.184512	0.601988
Tsen34	84.58349	-4.69084	3.538978	-1.32548	0.185012	0.603376
noc	1433.717	-1.19541	0.902366	-1.32475	0.185252	0.603669
betaTub85	106.4195	-4.67798	3.530802	-1.32491	0.185202	0.603669
alph	2676.577	-0.97232	0.734312	-1.32413	0.185461	0.604103
PolE4	580.2654	1.630672	1.23222	1.323361	0.185715	0.604653
CG1582	1134.817	-2.35749	1.781703	-1.32316	0.185781	0.604653
Yippee	3868.15	0.954064	0.721173	1.322933	0.185858	0.604659
CG31091	94.01192	-4.61975	3.492807	-1.32265	0.185953	0.604722
MED22	738.2565	-2.16129	1.635021	-1.32187	0.186211	0.604827
beta-PheR	334.5935	2.624545	1.985347	1.321958	0.186182	0.604827
Fkbp14	9792.822	0.619121	0.46835	1.321919	0.186195	0.604827
Topors	549.181	2.318456	1.755036	1.32103	0.186491	0.605494
CG5543	871.5534	-1.64377	1.244924	-1.32038	0.186709	0.605954
CG2918	2452.927	-1.01681	0.770878	-1.31903	0.187158	0.606329
CG18661	906.8664	1.611657	1.221591	1.31931	0.187066	0.606329
YL-1	365.2716	1.969129	1.495796	1.316443	0.188026	0.606329
CG11753	1860.177	-1.12705	0.856149	-1.31642	0.188033	0.606329
Srp72	616.6661	-1.95064	1.481666	-1.31652	0.188	0.606329
Cog6	455.9293	2.875003	2.183712	1.316567	0.187984	0.606329
blow	468.5615	-1.97658	1.500374	-1.31739	0.187707	0.606329
CG14985	575.1443	-1.91595	1.453511	-1.31815	0.187453	0.606329
Dek	3698.758	-0.81427	0.617272	-1.31915	0.187119	0.606329
mmy	2437.445	1.681209	1.274158	1.319467	0.187013	0.606329
dnr1	1025.017	-1.34297	1.01994	-1.31671	0.187935	0.606329
eIF2gamm	1146.708	-1.71424	1.30214	-1.31648	0.188012	0.606329
CTPsyn	979.9537	-1.55525	1.180691	-1.31724	0.187759	0.606329
HP5	1399.435	1.1127	0.843678	1.318867	0.187213	0.606329
CG1998	7628.41	-0.75495	0.572611	-1.31843	0.187358	0.606329
CG12316	766.8916	-2.01693	1.529626	-1.31858	0.18731	0.606329
CG12926	2079.803	0.960981	0.730795	1.31498	0.188516	0.607644
Rab21	3062.92	-1.09997	0.836723	-1.31461	0.188639	0.607682
Dll	1379.283	-1.13219	0.86131	-1.31449	0.18868	0.607682
kuk	3571.993	-1.05799	0.805163	-1.31401	0.188842	0.607686
dgt1	1022.486	1.731558	1.317551	1.314225	0.188771	0.607686
sol	933.2486	-1.75776	1.338133	-1.31359	0.188984	0.607686
PNUTS	7655.751	-0.67581	0.514419	-1.31374	0.188933	0.607686
CG34259	251.228	3.633364	2.766788	1.313207	0.189113	0.607859
abs	347.5255	2.857241	2.178624	1.311489	0.189693	0.607968
CG8036	1505.388	-1.24247	0.947169	-1.31177	0.189597	0.607968

CLS	1509.487	-1.10155	0.839557	-1.31206	0.1895	0.607968
AGBE	1702.965	-1.26879	0.967408	-1.31154	0.189676	0.607968
CG33543	4890.402	0.850691	0.648747	1.311284	0.189762	0.607968
Hsc20	989.5524	-1.40327	1.069193	-1.31246	0.189366	0.607968
lncRNA:CR	332.7482	2.159982	1.646847	1.311586	0.18966	0.607968
CG15019	1412.76	-1.17275	0.894489	-1.31109	0.189829	0.607968
U2af50	1620.861	1.888339	1.439521	1.311783	0.189593	0.607968
Lac	282.9318	3.160036	2.410825	1.310769	0.189936	0.608067
CG31678	1311.813	1.20991	0.924201	1.309143	0.190486	0.608404
CG8199	192.0142	2.969926	2.269384	1.308693	0.190639	0.608404
CG17193	240.2711	2.549199	1.94735	1.309061	0.190514	0.608404
BOD1	2278.814	-0.94751	0.723787	-1.3091	0.190499	0.608404
betaTub97	600.3488	-1.59387	1.217935	-1.30867	0.190648	0.608404
Arc1	2321.793	-0.95506	0.729074	-1.30996	0.190208	0.608404
Eip74EF	1426.445	-1.13858	0.869554	-1.30939	0.190403	0.608404
CG17249	662.994	-2.03592	1.555525	-1.30883	0.190592	0.608404
RpL5	32754.16	0.516922	0.395467	1.307118	0.191173	0.609838
CG14767	18448.42	0.541138	0.414138	1.30666	0.191328	0.610092
Prosap	10023.55	-0.69698	0.533839	-1.30559	0.191692	0.6109
CG1468	339.8211	3.243035	2.484195	1.305467	0.191734	0.6109
GalT1	5537.307	1.463063	1.122217	1.303726	0.192327	0.612546
CG6565	1672.806	-1.7527	1.345572	-1.30257	0.192723	0.613564
Arp1	2111.76	-1.45317	1.116188	-1.3019	0.19295	0.614042
CG8195	1575.274	1.241394	0.954104	1.301109	0.193221	0.614662
Dh31	5441.18	1.246053	0.957873	1.300854	0.193308	0.614696
XRCC1	162.5148	3.80251	2.924666	1.300152	0.193549	0.615217
Optix	326.9112	-2.51592	1.936059	-1.29951	0.19377	0.615676
cal1	881.4802	-1.95638	1.508067	-1.29728	0.194536	0.616889
flw	11398.34	-0.61072	0.470621	-1.29768	0.194397	0.616889
CG11275	1473.553	1.561377	1.202927	1.297982	0.194294	0.616889
Adf1	4652.053	-0.79663	0.613852	-1.29776	0.194369	0.616889
r-cup	244.4275	3.619274	2.789852	1.297299	0.194528	0.616889
Nckx30C	44193.02	0.553922	0.427335	1.296224	0.194898	0.617428
CG10543	6082.534	0.681331	0.525763	1.295889	0.195014	0.617428
CG8777	413.5862	2.728556	2.104683	1.296422	0.19483	0.617428
CG33158	1428.149	-1.32751	1.024228	-1.29611	0.194938	0.617428
CG6484	111.6383	-3.90257	3.013338	-1.2951	0.195286	0.618046
lncRNA:CR	3935.85	0.782788	0.604694	1.29452	0.195486	0.618245
ed	1583.768	-1.37007	1.058405	-1.29447	0.195503	0.618245
Irk2	12786.46	-0.61592	0.476276	-1.2932	0.195943	0.618662
xit	973.3995	1.306569	1.010267	1.293291	0.19591	0.618662
CG4617	296.4546	2.416346	1.868327	1.293321	0.1959	0.618662
PMCA	17059.06	0.590146	0.456329	1.293245	0.195926	0.618662
AP-2alpha	7072.477	-0.69958	0.541092	-1.29291	0.196044	0.618736
CG33170	946.3117	1.363983	1.055462	1.292309	0.19625	0.619145
CG5708	6260.548	0.697311	0.540158	1.290939	0.196725	0.620155
smg	2676.543	0.909361	0.704411	1.290952	0.19672	0.620155
Su(var)3-3	359.95	-2.82472	2.189274	-1.29026	0.196962	0.620657
Col4a1	4005.594	0.922145	0.715102	1.289529	0.197214	0.62071
CG30022	1631.41	-1.33352	1.034048	-1.28961	0.197186	0.62071

mtt	2030.121	-1.09304	0.847768	-1.28932	0.197288	0.62071
Nacalpha	7474.175	0.648131	0.502463	1.289908	0.197083	0.62071
CG2246	3792.122	-0.86	0.667324	-1.28872	0.197494	0.620961
CG3558	1263.761	1.485595	1.152837	1.288643	0.197522	0.620961
muc	5508.812	-0.70936	0.550869	-1.28771	0.197847	0.621738
Cat	2714.974	-1.00331	0.780018	-1.28626	0.198352	0.622949
CG4678	4836.902	0.731364	0.56874	1.285937	0.198465	0.622949
CG5989	1224.944	-1.76906	1.375587	-1.28604	0.198429	0.622949
lncRNA:CR	328.3448	2.076315	1.616173	1.284711	0.198893	0.624049
vri	959.069	-1.76611	1.375313	-1.28415	0.19909	0.624421
her	1039.766	-1.68471	1.313088	-1.28301	0.199489	0.625428
CG14435	1345.056	1.112051	0.868002	1.281162	0.200137	0.627216
CG9590	779.5023	-2.05346	1.603174	-1.28087	0.200239	0.627291
nudE	2786.142	1.246957	0.973841	1.280453	0.200386	0.627505
MFS14	608.5766	-2.02894	1.584993	-1.28009	0.200513	0.627658
Cont	1576.455	-1.46342	1.144102	-1.2791	0.200861	0.628502
CG11700	66.89872	4.43034	3.467875	1.277537	0.201413	0.62997
Tom40	3489.958	-1.02149	0.799709	-1.27733	0.201487	0.62997
CG17162	279.7211	2.946366	2.308312	1.276416	0.201809	0.63073
Orc5	130.8239	-4.4184	3.466305	-1.27467	0.202425	0.630937
CG31075	311.5856	-2.59936	2.037814	-1.27556	0.202111	0.630937
alpha-Man	2640.025	1.189513	0.932496	1.275623	0.202089	0.630937
CG10465	3644.168	0.763364	0.598625	1.275197	0.20224	0.630937
tay	5007.405	-0.73047	0.57297	-1.27489	0.202349	0.630937
Sytalpha	5055.435	0.805799	0.631984	1.275029	0.202299	0.630937
baz	267.8752	2.430517	1.905517	1.275516	0.202127	0.630937
CkIIalpha-i	2141.313	1.018606	0.799304	1.274365	0.202534	0.631032
CG6356	6201.997	-0.72883	0.572158	-1.27382	0.202727	0.631388
cno	1333.048	-1.43557	1.127213	-1.27356	0.202819	0.631431
Ubr3	7858.875	0.697344	0.548492	1.271384	0.203592	0.63359
CG7149	5795.769	-0.86912	0.683776	-1.27107	0.203705	0.633698
lncRNA:CR	167.8753	2.650836	2.085939	1.270812	0.203796	0.633733
CG17829	884.664	-1.96428	1.54957	-1.26763	0.204931	0.633825
CIAPIN1	571.4696	-1.64192	1.294772	-1.26811	0.204757	0.633825
Ndg	81.10155	-4.90502	3.867271	-1.26834	0.204676	0.633825
yellow-h	78.06376	-4.90433	3.867386	-1.26813	0.204753	0.633825
CG5789	265.2267	-3.25917	2.566222	-1.27003	0.204075	0.633825
Tbh	1845.716	-1.68928	1.330113	-1.27003	0.204075	0.633825
mbc	2899.539	-0.86272	0.679266	-1.27008	0.204057	0.633825
betaggt-I	452.5142	2.402832	1.894497	1.268322	0.204683	0.633825
nej	4562.142	-0.79767	0.628906	-1.26835	0.204672	0.633825
CG3760	1513.529	1.112311	0.877387	1.267754	0.204886	0.633825
lncRNA:CR	442.1358	2.036288	1.606062	1.267876	0.204842	0.633825
sima	7653.28	-0.65858	0.51907	-1.26876	0.204525	0.633825
MsrA	4159.617	0.823374	0.649486	1.267731	0.204894	0.633825
D19A	599.4875	2.20738	1.739139	1.269237	0.204356	0.633825
CG4462	285.7221	3.372108	2.661835	1.266836	0.205214	0.634456
lig	2628.84	-0.92514	0.730663	-1.26617	0.205452	0.634947
Hsp60C	131.2162	4.178175	3.300785	1.265813	0.20558	0.63501
Sply	3841.673	0.844224	0.667334	1.265069	0.205847	0.63501

Rpn6	1516.452	-1.63771	1.294622	-1.26501	0.205868	0.63501
lncRNA:CR	260.6201	2.692816	2.128513	1.265116	0.20583	0.63501
ttv	5047.587	-0.68669	0.54275	-1.26521	0.205797	0.63501
Culd	205.9061	-3.41968	2.704451	-1.26446	0.206063	0.635368
CG10311	1594.32	1.05607	0.835559	1.26391	0.206262	0.635738
ctp	24644.26	0.540212	0.427744	1.262934	0.206613	0.636574
CG5937	1407.759	1.919794	1.521304	1.26194	0.20697	0.636945
Sf3b1	987.8599	-1.91577	1.519188	-1.26105	0.207291	0.636945
Atx2	3341.496	0.885018	0.702183	1.260381	0.207532	0.636945
mRpL55	3172.555	0.866595	0.687462	1.260571	0.207463	0.636945
CG31126	1162.398	1.890427	1.498947	1.26117	0.207248	0.636945
Phb2	4157.325	-0.73523	0.58318	-1.26073	0.207408	0.636945
GstE3	1938.02	-1.7243	1.368194	-1.26027	0.207571	0.636945
MED16	219.843	-3.38216	2.683878	-1.26018	0.207606	0.636945
Cdk5	335.8457	-2.12875	1.687786	-1.26127	0.207211	0.636945
mxc	1168.257	1.348344	1.069128	1.261162	0.20725	0.636945
Pxn	868.6456	-2.01016	1.594099	-1.261	0.207309	0.636945
CG8635	1777.355	1.11834	0.887824	1.259641	0.207799	0.636979
Atg10	307.0521	-2.20727	1.752798	-1.25928	0.207929	0.636979
Elp2	353.5727	2.54336	2.019719	1.259264	0.207935	0.636979
tea	1044.278	1.215767	0.965015	1.259843	0.207726	0.636979
dre4	308.8461	2.520257	2.0024	1.258618	0.208168	0.637451
CG16812	243.2139	-2.65706	2.112336	-1.25788	0.208437	0.637833
Galk	1337.483	-1.35527	1.077467	-1.25783	0.208452	0.637833
baf	3490.811	0.939566	0.748146	1.25586	0.209167	0.638203
CG6583	1868.087	-1.14727	0.913383	-1.25607	0.209092	0.638203
Sirt1	2065.697	1.141073	0.908014	1.256669	0.208873	0.638203
mRpL4	1593.834	1.231777	0.980915	1.255743	0.209209	0.638203
MED7	951.4324	-1.39443	1.110165	-1.25606	0.209094	0.638203
CG11391	2970.926	1.309006	1.042136	1.25608	0.209087	0.638203
Acf	719.1344	-1.51585	1.206215	-1.2567	0.208862	0.638203
LeuRS-m	605.7535	-1.78134	1.418334	-1.25594	0.209139	0.638203
Sps1	287.4814	-2.16384	1.723749	-1.25531	0.209366	0.63844
salr	193.3387	2.939961	2.342665	1.254964	0.209492	0.63858
CG8677	7353.131	-0.89382	0.713364	-1.25296	0.21022	0.640557
PK2-R2	134.3573	3.560128	2.843261	1.252129	0.210523	0.640993
pico	3945.288	-0.81443	0.650387	-1.25223	0.210487	0.640993
Nup154	242.9026	2.8439	2.273016	1.251157	0.210877	0.641828
sno	3769.053	-0.89141	0.712813	-1.25056	0.211096	0.642251
CG4557	207.8462	3.1145	2.492227	1.249686	0.211414	0.642975
Tsp66E	11477.64	0.716969	0.573875	1.249348	0.211538	0.643107
beag	285.9391	2.787988	2.232222	1.248974	0.211675	0.643279
ctrip	3923.261	0.836345	0.670144	1.248009	0.212028	0.644108
CG4751	396.2862	1.916898	1.537876	1.246458	0.212596	0.64556
grp	1927.927	-0.96497	0.774307	-1.24624	0.212676	0.64556
lncRNA:CR	1129.801	1.284615	1.030951	1.246048	0.212747	0.64556
CG3191	2000.143	-1.17511	0.943487	-1.24549	0.212951	0.64569
Ars2	4121.408	-0.74854	0.600953	-1.24558	0.212918	0.64569
Tm2	134.5302	-3.34653	2.688702	-1.24466	0.213255	0.646126
RhoGAP18	2637.722	-0.87804	0.705432	-1.24468	0.213248	0.646126

Ibf2	79.74908	-4.00165	3.218618	-1.24328	0.213764	0.647423
CG3817	130.5576	-4.02445	3.239013	-1.24249	0.214054	0.647527
CG30020	1146.716	1.92419	1.548812	1.242365	0.214102	0.647527
Plp	2755.636	0.942799	0.758528	1.242932	0.213893	0.647527
CG7872	805.4906	-1.88807	1.519805	-1.24231	0.214121	0.647527
CG10208	1309.959	-1.27016	1.022624	-1.24206	0.214214	0.647565
LPCAT	1371.548	1.081023	0.871263	1.240754	0.214697	0.648732
CG3732	1677.549	1.143034	0.921403	1.240536	0.214777	0.648732
Cbs	567.6381	1.842847	1.485737	1.240359	0.214843	0.648732
Membrin	592.8414	1.724869	1.391126	1.239908	0.215009	0.648746
Herc4	3008.693	0.790433	0.637467	1.239959	0.214991	0.648746
phyl	2308.883	0.902306	0.728127	1.239216	0.215266	0.649275
CG7382	1520.458	-1.0584	0.855019	-1.23787	0.215765	0.649292
SCAR	1377.987	-1.57662	1.273953	-1.23758	0.215871	0.649292
GstE2	7270.429	-0.60775	0.491192	-1.2373	0.215977	0.649292
CG12056	771.9236	-1.42967	1.155244	-1.23755	0.215884	0.649292
Gga	670.7408	2.111611	1.705012	1.238473	0.215541	0.649292
Ir93a	233.084	2.558307	2.067178	1.237585	0.21587	0.649292
hoip	512.045	-2.03705	1.645501	-1.23795	0.215735	0.649292
Ncc69	6548.738	-0.9616	0.777214	-1.23724	0.215999	0.649292
ppk26	341.7271	3.192921	2.580319	1.237413	0.215934	0.649292
xmas	506.0936	2.401945	1.942636	1.236436	0.216297	0.649943
prtp	2358.825	-0.89514	0.724372	-1.23575	0.216553	0.650227
Nup205	65.88088	4.703808	3.806042	1.235879	0.216504	0.650227
x16	1311.654	1.388322	1.123792	1.235391	0.216685	0.65038
CG5439	465.2087	-2.2104	1.789869	-1.23495	0.216848	0.650382
mt:CoI	777542	0.452085	0.366028	1.235111	0.216789	0.650382
Eato	752.0366	-1.85147	1.500071	-1.23425	0.217108	0.650676
CG11560	1631.962	1.760482	1.426271	1.234325	0.217082	0.650676
GAPcenA	2116.868	-0.99643	0.80759	-1.23383	0.217267	0.650684
CG8974	1977.941	1.072374	0.869155	1.233812	0.217273	0.650684
TfIIA-L	988.1075	-1.34854	1.093828	-1.23286	0.217628	0.651503
lncRNA:CR	215.7565	2.67458	2.169795	1.232641	0.21771	0.651505
CG5028	8056.569	0.737093	0.598818	1.230914	0.218355	0.652497
CG17078	1187.418	1.735025	1.408888	1.231485	0.218142	0.652497
cass	7337.266	0.677884	0.550729	1.230885	0.218366	0.652497
RpS5a	16447.38	0.51831	0.421087	1.230886	0.218365	0.652497
Dap160	8174.955	0.637431	0.518286	1.229883	0.218741	0.653374
CG7988	495.3031	2.552677	2.076783	1.22915	0.219016	0.653951
su(sable)	1734.978	-0.9879	0.804833	-1.22745	0.219652	0.654875
CG13995	2913.147	-1.43983	1.172898	-1.22758	0.219604	0.654875
lncRNA:CR	169.4895	3.538555	2.88228	1.227693	0.219562	0.654875
mus304	116.9229	-3.69258	3.007093	-1.22796	0.219463	0.654875
ClC-a	9087.179	-0.63516	0.517793	-1.22667	0.219946	0.655435
CG17839	1063.416	1.181307	0.963136	1.226521	0.220003	0.655435
CG6983	3702.264	0.864226	0.704873	1.226073	0.220171	0.655693
CG13367	1005.499	1.97166	1.611317	1.223633	0.221091	0.656241
CG13248	2187.769	1.574498	1.286311	1.224042	0.220936	0.656241
Mes2	2411.409	0.871666	0.712182	1.223938	0.220976	0.656241
GM130	597.0389	-1.59398	1.302621	-1.22367	0.221077	0.656241

CG8388	838.5695	-2.18623	1.785422	-1.22449	0.220768	0.656241
Tret1-1	1582.384	-1.27493	1.041279	-1.22439	0.220807	0.656241
mfas	25780.32	-0.57955	0.47334	-1.22437	0.220811	0.656241
CG8173	147.2017	3.440271	2.808678	1.224872	0.220624	0.656241
Ankle2	779.7814	-1.90106	1.551526	-1.22528	0.220469	0.656241
Pdk	9913.759	-0.58582	0.478866	-1.22335	0.221197	0.656313
syd	2691.808	-0.87788	0.717727	-1.22313	0.221279	0.656315
cyst	907.8468	-1.28945	1.054532	-1.22277	0.221418	0.656482
bwa	3900.063	0.924765	0.756927	1.221737	0.221807	0.656667
Smyd5	305.8946	2.711982	2.21938	1.221955	0.221725	0.656667
RpL23A	9604.552	0.5807	0.475283	1.221799	0.221784	0.656667
Spn	4806.605	-0.68484	0.560252	-1.22237	0.221567	0.656667
Ppa	1262.554	1.14796	0.939843	1.221438	0.22192	0.656759
CG5810	175.4711	3.134348	2.569185	1.219978	0.222473	0.658154
Tsp5D	14764.38	-0.57246	0.47013	-1.21767	0.223349	0.659123
CG6712	199.1023	-2.98005	2.446657	-1.21801	0.223221	0.659123
CG6937	115.8083	3.400775	2.793015	1.2176	0.223376	0.659123
CG10513	18487.94	-0.67011	0.550203	-1.21793	0.223251	0.659123
CG1344	1911.304	-1.42437	1.169252	-1.21819	0.223151	0.659123
Prosalpha2	2273.224	0.93061	0.764168	1.217808	0.223297	0.659123
CG10672	944.2735	-1.50031	1.231238	-1.21854	0.22302	0.659123
CG3909	680.1454	-1.58361	1.301555	-1.21671	0.223716	0.659127
Cyp6a23	705.8034	1.745625	1.435147	1.216338	0.223856	0.659127
sff	5437.269	0.739954	0.608364	1.216301	0.22387	0.659127
MED14	310.5603	-2.94857	2.423194	-1.21681	0.223676	0.659127
sNPF-R	728.8763	-2.30237	1.892569	-1.21653	0.223783	0.659127
galla-2	536.0149	-1.61698	1.329236	-1.21648	0.223804	0.659127
Dmtn	2464.618	-0.93197	0.766852	-1.21532	0.224245	0.659964
CR15345	160.9399	-3.70248	3.046993	-1.21512	0.224319	0.659964
eIF2Balpha	1378.201	1.116072	0.918692	1.214849	0.224424	0.660031
pes	238.3977	2.520642	2.076077	1.214137	0.224695	0.660105
Kap3	1428.496	1.312126	1.080395	1.214487	0.224562	0.660105
cactin	144.5622	-3.00058	2.471075	-1.21428	0.22464	0.660105
CG12344	1970.836	1.421843	1.171413	1.213785	0.22483	0.660126
Amun	839.8067	-1.49218	1.229617	-1.21354	0.224925	0.660126
Rgl	2163.426	-0.96671	0.796645	-1.21347	0.224949	0.660126
ifc	3053.751	-0.83516	0.688999	-1.21214	0.225458	0.660894
Ns3	403.0116	-1.93745	1.598012	-1.21241	0.225355	0.660894
Rh50	16298.91	-0.6396	0.527646	-1.21217	0.225447	0.660894
unc79	14156.73	0.788779	0.650933	1.211768	0.225601	0.661072
p53	240.4001	3.750952	3.09728	1.211047	0.225877	0.661639
CG3823	62.73417	-4.61156	3.809225	-1.21063	0.226037	0.661756
CG17292	256.6922	-2.95497	2.441602	-1.21026	0.22618	0.661756
CG9336	605.0463	1.560332	1.289442	1.210083	0.226247	0.661756
CG33506	220.6125	2.888851	2.387288	1.210097	0.226241	0.661756
Pkc98E	11494.33	-0.58382	0.482663	-1.20958	0.226441	0.661842
CG2064	257.0568	-3.25921	2.694032	-1.20979	0.22636	0.661842
hrm	4636.118	-0.89368	0.739013	-1.20928	0.226554	0.661929
CG15812	156.6184	3.449162	2.852815	1.209038	0.226648	0.661965
CG9941	916.7044	-1.32939	1.100566	-1.20791	0.227081	0.662986

nAChRalph	15985.89	0.531325	0.440269	1.206818	0.227502	0.663976
Lim3	220.9549	2.815996	2.335512	1.20573	0.227922	0.664717
CG6574	1193.863	1.421905	1.179285	1.205735	0.22792	0.664717
chn	4632.03	-0.72434	0.60099	-1.20524	0.228111	0.665028
Mtpalpha	2406.828	-1.30071	1.081127	-1.20311	0.228934	0.666442
CG11920	265.8487	2.726858	2.265785	1.203493	0.228785	0.666442
fkh	467.756	1.89652	1.575805	1.203525	0.228773	0.666442
bab1	79.9409	-3.55004	2.951213	-1.20291	0.229011	0.666442
g	1520.703	0.996734	0.828405	1.203196	0.228901	0.666442
CR14033	1781.699	-1.18427	0.985323	-1.20191	0.229398	0.666599
CG6420	1192.745	-1.2436	1.034602	-1.202	0.229362	0.666599
chrb	15125.57	-0.54924	0.456736	-1.20253	0.229158	0.666599
Set1	7657.342	0.615532	0.512056	1.202079	0.229333	0.666599
CG30394	851.9972	1.33879	1.114333	1.201427	0.229585	0.666903
CG10265	972.724	1.709847	1.423467	1.201185	0.229679	0.666934
CG14252	286.9518	-2.55366	2.126715	-1.20075	0.229848	0.667182
AANATL2	1476.188	1.432324	1.193287	1.200318	0.230016	0.667429
CG4658	709.9847	1.442396	1.201935	1.200061	0.230116	0.667437
Tfb1	538.654	1.805284	1.50455	1.199883	0.230185	0.667437
CG32813	597.7917	1.478238	1.237982	1.19407	0.23245	0.667724
N	5014.033	-0.65928	0.553995	-1.19005	0.234028	0.667724
HIPP1	2090.968	-1.72008	1.444494	-1.19078	0.233739	0.667724
Liprin-alph	2967.62	1.08388	0.907229	1.194715	0.232198	0.667724
Rca1	1259.624	1.571829	1.320645	1.190198	0.233969	0.667724
CG1142	851.6314	-1.5781	1.325555	-1.19052	0.233843	0.667724
Elp1	476.5085	-2.27369	1.908133	-1.19158	0.233427	0.667724
PGAP3	1155.634	1.614005	1.352381	1.193454	0.232691	0.667724
CG30158	2977.399	1.391381	1.166838	1.192437	0.23309	0.667724
Inx2	15020.5	-0.61011	0.510152	-1.19594	0.231721	0.667724
CG5065	5836.926	-0.64352	0.537664	-1.19688	0.231354	0.667724
prim	2285.633	-0.94315	0.787417	-1.19778	0.231004	0.667724
Spred	5281.025	0.810477	0.677929	1.19552	0.231884	0.667724
Roe1	1799.016	-1.54049	1.287895	-1.19613	0.231645	0.667724
pnt	2119.762	-0.8635	0.723306	-1.19382	0.232549	0.667724
Start1	1352.743	-1.14869	0.965059	-1.19028	0.233937	0.667724
CG3009	3651.197	0.816236	0.685197	1.191243	0.233558	0.667724
spel1	1069.664	-1.90379	1.596807	-1.19225	0.233165	0.667724
CG14995	5389.9	0.678489	0.569177	1.192054	0.23324	0.667724
CG42232	4260.756	0.781602	0.655443	1.192478	0.233074	0.667724
Pde9	7001.444	0.701194	0.586973	1.194593	0.232246	0.667724
CG42365	789.4097	-2.07861	1.73364	-1.19899	0.230532	0.667724
wech	5726.142	-0.62946	0.52822	-1.19166	0.233396	0.667724
CG42445	249.7165	-2.91173	2.434801	-1.19588	0.231744	0.667724
lncRNA:CR	10905.45	0.593106	0.495458	1.197086	0.231273	0.667724
Chi	1229.098	-1.25774	1.055496	-1.19161	0.233414	0.667724
CG40486	280.2859	3.557676	2.980019	1.193844	0.232539	0.667724
orb2	10745.59	0.553779	0.463214	1.195516	0.231885	0.667724
CG43813	225.282	3.437662	2.882138	1.192747	0.232968	0.667724
asRNA:CR4	596.1106	1.800053	1.50199	1.198446	0.230744	0.667724
lncRNA:CR	230.3198	3.373812	2.813191	1.199283	0.230418	0.667724

lncRNA:CR	281.0032	2.387525	1.99661	1.195789	0.231779	0.667724
gry	379.8824	-1.71826	1.43556	-1.19693	0.231334	0.667724
l(3)80Fg	30907	0.472977	0.394683	1.198372	0.230772	0.667724
18w	3476.911	-0.72086	0.605373	-1.19077	0.233743	0.667724
l(3)02640	2253.677	0.838077	0.703488	1.191316	0.23353	0.667724
dos	1233.381	-1.21198	1.015494	-1.19349	0.232677	0.667724
CG1552	29751.57	-0.54062	0.45264	-1.19437	0.232332	0.667724
CG1637	7310.725	0.603353	0.505336	1.193963	0.232492	0.667724
Alg14	672.5097	-1.38259	1.155777	-1.19624	0.231603	0.667724
bnb	28811.54	-0.58856	0.493031	-1.19377	0.232569	0.667724
Prosbeta6	3039.106	-0.76334	0.640361	-1.19205	0.233243	0.667724
RhoGAP71	4999.28	-0.81447	0.683961	-1.19081	0.233727	0.667724
Liprin-beta	819.476	1.761068	1.473426	1.19522	0.232001	0.667724
Hsp26	788.2891	-1.47271	1.235543	-1.19195	0.233279	0.667724
Slh	685.909	-1.70547	1.433511	-1.18972	0.234157	0.667856
CG13506	563.3044	1.808982	1.522679	1.188026	0.234823	0.669193
Rap2l	3148.822	0.782164	0.658308	1.188144	0.234777	0.669193
Paf-AHalph	1114.137	-1.1393	0.959097	-1.18789	0.234876	0.669193
crn	81.38455	4.502695	3.791764	1.187493	0.235033	0.669257
Uba5	725.4496	2.053387	1.729298	1.187411	0.235065	0.669257
CG5021	445.2569	-1.72352	1.452332	-1.18673	0.235335	0.669788
CG3626	970.4633	-1.3901	1.173275	-1.1848	0.236096	0.670556
l(2)gl	1232.111	-1.59593	1.347317	-1.18453	0.236204	0.670556
P5CDh1	15524.76	0.605458	0.510894	1.185095	0.23598	0.670556
dpr19	9148.842	0.623424	0.526148	1.184884	0.236063	0.670556
CG9649	3874.232	0.716514	0.605412	1.183515	0.236605	0.670556
CG31469	354.1561	2.259854	1.907994	1.184413	0.23625	0.670556
Not1	9373.397	0.617469	0.52158	1.183843	0.236475	0.670556
CG42458	94.19074	4.382127	3.702273	1.183632	0.236559	0.670556
Brms1	943.8747	-1.66297	1.402919	-1.18537	0.235873	0.670556
jub	136.4862	-3.01597	2.544735	-1.18518	0.235946	0.670556
RhoGAPp1	5726.67	0.624522	0.527687	1.183508	0.236608	0.670556
lqf	6614.188	-0.62442	0.527587	-1.18354	0.236594	0.670556
Syb	3137.745	-0.86247	0.729752	-1.18186	0.23726	0.672167
Dip-C	1754.394	1.150282	0.973983	1.181008	0.237599	0.672833
Atg12	1265.998	-1.08877	0.922187	-1.18064	0.237746	0.672833
TH1	365.9924	-2.20756	1.86963	-1.18075	0.237702	0.672833
CYLD	1957.907	0.905991	0.767716	1.180113	0.237955	0.673105
CG15706	536.9696	-1.89478	1.605783	-1.17997	0.238011	0.673105
CG11089	562.9503	2.169381	1.839371	1.179415	0.238233	0.673113
ADD1	877.8322	1.603022	1.359259	1.179335	0.238265	0.673113
mRpS30	2111.203	0.943685	0.8	1.179606	0.238157	0.673113
E23	761.7385	1.293921	1.098015	1.178419	0.23863	0.67367
lncRNA:CR	308.7644	1.865729	1.583225	1.178436	0.238623	0.67367
CG6153	139.8896	3.407445	2.893841	1.177482	0.239003	0.674409
CG14694	220.1326	-3.44123	2.922887	-1.17734	0.23906	0.674409
CG6527	189.353	-2.9274	2.487561	-1.17682	0.239269	0.674761
Snx6	974.867	2.117302	1.802085	1.174918	0.240028	0.675004
BicD	3212.223	0.76141	0.64726	1.176359	0.239452	0.675004
CG10428	156.9353	-3.60782	3.068641	-1.17571	0.239712	0.675004

shg	669.3132	-1.4514	1.234936	-1.17528	0.239881	0.675004
Rme-8	1268.605	1.285134	1.093754	1.174975	0.240005	0.675004
CG11127	687.1674	1.848822	1.573267	1.175148	0.239935	0.675004
CG3735	385.4759	2.229672	1.897477	1.175072	0.239966	0.675004
Ptpa	919.9111	-1.71045	1.455496	-1.17517	0.239927	0.675004
CG8671	4316.788	-0.71374	0.607986	-1.17394	0.24042	0.675736
na	4939.006	0.92643	0.789226	1.173847	0.240456	0.675736
PpD3	1415.23	0.990401	0.844272	1.173083	0.240763	0.676092
CG12338	714.9725	-2.19262	1.868925	-1.1732	0.240716	0.676092
Rim2	1561.36	1.728588	1.473772	1.172901	0.240836	0.676092
ths	5583.116	0.681221	0.581472	1.171547	0.241379	0.677187
dpr8	3177.965	0.7527	0.642515	1.17149	0.241402	0.677187
CG7339	1041.889	-1.58688	1.354801	-1.1713	0.241479	0.677187
zetaCOP	4920.071	-0.65385	0.558578	-1.17055	0.241778	0.677553
Uch-L5	1001.046	-1.31482	1.123078	-1.17073	0.241708	0.677553
dgrn	1592.597	-0.98303	0.840126	-1.1701	0.241962	0.67781
CG8207	158.3078	-3.15639	2.698902	-1.16951	0.242199	0.67781
CG43092	197.858	2.194885	1.87713	1.169277	0.242292	0.67781
Hlc	1303.517	1.24532	1.064913	1.16941	0.242238	0.67781
CG3008	453.9166	1.706401	1.458606	1.169885	0.242047	0.67781
CG4297	3315.401	-0.74349	0.636358	-1.16836	0.242663	0.678374
Stacl	21613.71	0.517684	0.443081	1.168374	0.242656	0.678374
alrm	15593.65	-0.51941	0.445023	-1.16714	0.243153	0.679508
SmydA-7	401.5204	2.596013	2.225253	1.166615	0.243366	0.679539
Pax	1755.176	0.990246	0.848904	1.166499	0.243413	0.679539
asf1	1261.061	-1.80473	1.547433	-1.16628	0.243503	0.679539
NHP2	1894.073	-1.67013	1.431818	-1.16644	0.243436	0.679539
CG32221	446.5501	-1.95171	1.675116	-1.16512	0.24397	0.680606
eIF1A	5480.751	-0.73007	0.626881	-1.16461	0.244178	0.680948
CHKov2	2037.539	1.610477	1.384354	1.163341	0.244691	0.681578
CG1647	555.6808	1.70795	1.468303	1.163213	0.244743	0.681578
CG12112	361.2078	-2.60854	2.241761	-1.16361	0.244581	0.681578
osp	1911.165	-1.07411	0.922968	-1.16376	0.244521	0.681578
DIP-kappa	1020.112	1.869716	1.608134	1.162662	0.244967	0.681964
FRG1	230.3828	-2.3591	2.031897	-1.16104	0.245628	0.683567
mod(mdg4	4023.595	-0.74529	0.642388	-1.16019	0.245973	0.683835
Trs23	604.7027	2.023391	1.744177	1.160084	0.246015	0.683835
Ack-like	903.9007	1.205844	1.039188	1.160372	0.245898	0.683835
KdelR	3974.729	-0.68248	0.588368	-1.15996	0.246065	0.683835
Rchy1	1347.02	1.378558	1.189629	1.158813	0.246532	0.684128
Uvrag	464.3265	-2.01469	1.739983	-1.15788	0.246914	0.684128
CG10283	480.32	-1.84133	1.590111	-1.15799	0.246868	0.684128
mfrn	3146.591	0.790423	0.682682	1.157821	0.246937	0.684128
CG30389	3157.231	0.755189	0.651833	1.158562	0.246635	0.684128
pix	5569.846	0.653263	0.563812	1.158654	0.246597	0.684128
CG3548	1704.009	1.150613	0.992348	1.159485	0.246258	0.684128
mthl1	527.6822	1.630481	1.408024	1.157992	0.246867	0.684128
CG15432	418.5515	1.961217	1.692958	1.158455	0.246678	0.684128
edl	211.7934	-2.2704	1.963728	-1.15617	0.247613	0.685655
CG34367	1660.633	0.920935	0.79662	1.156053	0.247659	0.685655

RNaseMRF	198.6895	-2.34339	2.02767	-1.15571	0.247801	0.685812
CG6465	3215.222	0.77196	0.668094	1.155467	0.247899	0.685846
CG5273	18752.81	-0.49511	0.42943	-1.15295	0.248932	0.686853
tub	1199.08	1.241983	1.076183	1.154062	0.248475	0.686853
PGAP5	770.1189	-1.62906	1.416782	-1.14983	0.250215	0.686853
CG9328	13281.51	0.521009	0.451723	1.153382	0.248754	0.686853
alpha-Est9	2882.626	0.972659	0.845963	1.149765	0.250241	0.686853
CG14882	289.5724	2.936626	2.552902	1.150309	0.250017	0.686853
CG14879	1491.61	-1.94845	1.694132	-1.15012	0.250095	0.686853
Cbp20	2118.721	-1.00139	0.870299	-1.15062	0.249887	0.686853
CG6142	103.0493	-3.27093	2.844378	-1.14996	0.250159	0.686853
CG30156	75.46855	4.083458	3.54088	1.153233	0.248815	0.686853
eIF3j	1345.312	1.147193	0.997232	1.150377	0.249989	0.686853
Pka-R2	8949.556	-0.53769	0.467592	-1.14992	0.250178	0.686853
4E-T	1567.028	-1.02712	0.893479	-1.14958	0.250317	0.686853
spn-F	393.0129	-1.98455	1.720413	-1.15353	0.248693	0.686853
poly	2065.532	-1.08821	0.943568	-1.15329	0.24879	0.686853
CG42336	1578.921	-0.95671	0.830621	-1.1518	0.249402	0.686853
Nuak	3448.095	0.716608	0.622363	1.151431	0.249555	0.686853
CG3065	1741.014	-0.99886	0.867325	-1.15166	0.249461	0.686853
Vti1b	1866.032	-1.1119	0.964152	-1.15324	0.24881	0.686853
VAChT	19502.8	0.481758	0.418278	1.151764	0.249418	0.686853
Mcm3	117.7986	-3.70575	3.22285	-1.14984	0.250212	0.686853
Elys	1692.598	-1.6224	1.411227	-1.14964	0.250293	0.686853
eIF3e	2881.713	-0.83363	0.723359	-1.15245	0.249137	0.686853
CG1722	646.6927	-1.69268	1.469864	-1.15159	0.249491	0.686853
CG7785	475.1817	1.775933	1.545153	1.149357	0.250409	0.686869
spir	16709.17	0.602491	0.524853	1.147923	0.251	0.688257
Nlp	1880.364	1.070083	0.933012	1.146913	0.251418	0.68893
CG14431	866.4342	1.7052	1.486671	1.146992	0.251385	0.68893
Slu7	856.2767	-1.18532	1.033906	-1.14645	0.251608	0.689216
CtIP	141.9802	-3.05201	2.665053	-1.1452	0.252127	0.689656
CG11147	387.1078	2.781348	2.427848	1.145602	0.25196	0.689656
CG31195	239.4712	-2.70635	2.363572	-1.14503	0.252198	0.689656
Fs(2)Ket	8243.913	0.580686	0.507071	1.145176	0.252136	0.689656
asRNA:CR4	671.1373	-1.30551	1.139686	-1.1455	0.252001	0.689656
CG7148	1191.14	-1.51606	1.325121	-1.1441	0.252584	0.690146
mt:CoIII	646076.6	0.418473	0.365768	1.144093	0.252585	0.690146
Xrp1	11433.55	0.607019	0.53072	1.143765	0.252721	0.690146
Nup93-1	671.7353	-1.70975	1.494626	-1.14393	0.252652	0.690146
Tmtc3	332.6986	-2.68818	2.350767	-1.14353	0.252817	0.690173
CG31549	2772.181	0.815975	0.713794	1.143151	0.252976	0.690372
CG32486	12301.36	0.549671	0.480938	1.142916	0.253073	0.690404
puml	2783.077	0.815048	0.714296	1.14105	0.253849	0.690867
CG33523	2546.508	-0.81995	0.718269	-1.14157	0.253635	0.690867
Syt7	15630.06	0.486431	0.426376	1.140851	0.253932	0.690867
Orct2	807.2702	-1.2147	1.063674	-1.14199	0.253458	0.690867
lncRNA:CR	188.1428	-3.24954	2.847357	-1.14125	0.253767	0.690867
Sc2	4334.589	0.688566	0.60311	1.141692	0.253582	0.690867
MSBP	6399.218	-0.64924	0.568691	-1.14164	0.253603	0.690867

arx	302.4229	-3.30354	2.894264	-1.14141	0.253699	0.690867
MtnA	39144.81	-0.52259	0.458747	-1.13917	0.254633	0.691483
imd	64.6253	4.245331	3.726343	1.139275	0.254588	0.691483
CG30325	193.8318	-3.80441	3.337244	-1.13998	0.254293	0.691483
CG6984	157.8137	3.061553	2.687773	1.139067	0.254675	0.691483
lncRNA:CR	307.6499	1.814916	1.59278	1.139464	0.25451	0.691483
CG9368	4564.955	-0.78552	0.689479	-1.1393	0.254578	0.691483
lncRNA:CR	430.2245	2.524186	2.216725	1.1387	0.254828	0.691664
CG16989	789.0155	1.867367	1.642302	1.137043	0.25552	0.69284
Sec24CD	2060.242	-0.84842	0.745928	-1.1374	0.25537	0.69284
Flacc	521.3711	2.039837	1.793931	1.137077	0.255506	0.69284
mora	96.80636	3.803905	3.347276	1.136418	0.255782	0.693314
Sdr	181.5831	2.960967	2.607242	1.13567	0.256095	0.69346
Trc8	990.1174	-1.68238	1.481248	-1.13579	0.256045	0.69346
Vti1a	638.267	-1.84703	1.626196	-1.1358	0.25604	0.69346
sav	159.4807	-3.56085	3.139077	-1.13436	0.256643	0.694009
SelG	1120.471	1.289125	1.136406	1.134388	0.256632	0.694009
Prosbeta2	1193.36	-1.50574	1.327376	-1.13438	0.256637	0.694009
Mpcp2	51994.89	0.4945	0.435782	1.13474	0.256484	0.694009
Irk3	4942.495	-0.76227	0.672599	-1.13332	0.257079	0.694954
Ak6	163.7627	3.613072	3.190215	1.132548	0.257404	0.695598
CG32756	793.2285	1.429161	1.262543	1.13197	0.257647	0.696019
CG13896	83.43297	-4.38305	3.875512	-1.13096	0.258071	0.696932
cDIP	75.37623	-4.38142	3.875742	-1.13047	0.258278	0.697019
CG5323	760.2928	1.54765	1.368798	1.130664	0.258197	0.697019
CG32192	218.8956	-2.80574	2.483123	-1.12992	0.258509	0.697409
CG5445	2067.468	-0.95692	0.847049	-1.12971	0.2586	0.697421
CG43740	970.1144	1.128616	0.999361	1.129337	0.258756	0.697606
CG3815	294.2454	2.985739	2.645961	1.128414	0.259145	0.698355
Agpat2	1182.727	-1.28378	1.137904	-1.1282	0.259237	0.698355
ATP8B	5468.191	-0.62983	0.558626	-1.12745	0.25955	0.698355
Set	1467.85	-1.33417	1.182907	-1.12787	0.259374	0.698355
EcR	7325.367	-0.58804	0.521529	-1.12754	0.259514	0.698355
lncRNA:CR	1329.523	0.977835	0.867345	1.127388	0.259578	0.698355
CG13001	155.8517	3.527006	3.128897	1.127236	0.259643	0.698355
CG17036	104.3377	-3.86581	3.438054	-1.12442	0.260836	0.699801
Hs2st	1326.767	0.995149	0.885275	1.124113	0.260965	0.699801
Drice	1124.27	2.091549	1.860586	1.124134	0.260956	0.699801
sip3	4161.995	0.725698	0.645121	1.124903	0.26063	0.699801
CG4853	1085.307	-1.54922	1.377827	-1.12439	0.260848	0.699801
CG4593	197.7391	3.090226	2.748175	1.124465	0.260816	0.699801
CG6758	513.6772	-1.97054	1.750872	-1.12546	0.260394	0.699801
GstE13	1454.741	-1.31851	1.17203	-1.12498	0.260596	0.699801
mRpL49	1633.552	0.964927	0.857634	1.125104	0.260545	0.699801
CG3803	274.5546	1.83748	1.635864	1.123248	0.261332	0.700552
RpL22	5021.203	-0.60882	0.542451	-1.12235	0.261712	0.70127
f-cup	1445.689	-1.36471	1.216095	-1.12221	0.261775	0.70127
CG8963	1248.168	1.058737	0.943785	1.121799	0.261948	0.701465
Slob	7681.319	0.613369	0.546858	1.121624	0.262022	0.701465
Scgbeta	3930.308	0.729068	0.650567	1.120665	0.262431	0.702195

beat-VI	1480.323	-1.50573	1.344208	-1.12016	0.262645	0.702195
CstF50	462.2191	-1.9585	1.748221	-1.12028	0.262595	0.702195
CG4598	710.8043	1.286741	1.148495	1.120371	0.262556	0.702195
Cyp4p1	281.0208	2.648303	2.365478	1.119564	0.2629	0.70248
GluRIB	3448.221	0.725745	0.648276	1.119501	0.262927	0.70248
hyx	1171.649	1.669129	1.492229	1.118548	0.263333	0.703332
PR-Set7	198.6371	2.433533	2.176406	1.118143	0.263506	0.703559
CG31368	586.2447	1.886614	1.688599	1.117266	0.263881	0.703871
Wdr37	1194.745	-1.17169	1.048421	-1.11758	0.263747	0.703871
mGluR	2344.979	-0.81057	0.7255	-1.11725	0.263886	0.703871
Eb1	6655.501	-0.6165	0.552165	-1.11651	0.264204	0.704484
msl-2	967.5545	1.623915	1.454826	1.116226	0.264325	0.704575
D12	188.4813	-3.6731	3.298392	-1.1136	0.26545	0.704701
ClpP	2268.826	-0.88827	0.797023	-1.11448	0.265072	0.704701
ScpX	1078.024	-1.27756	1.146871	-1.11396	0.265298	0.704701
CG1234	396.8451	2.173032	1.950749	1.113948	0.265302	0.704701
RhoGAP93	5442.03	0.784984	0.70369	1.115526	0.264625	0.704701
Bili	1677.832	0.899775	0.806969	1.115005	0.264848	0.704701
bigmax	1198.038	1.115931	1.002226	1.113453	0.265514	0.704701
eEF1alpha	17902.9	0.556149	0.498998	1.114533	0.265051	0.704701
Grip128	80.13126	3.975933	3.564151	1.115534	0.264621	0.704701
mgr	1384.304	-0.96397	0.865294	-1.11403	0.265264	0.704701
AstA-R1	1219.926	-1.42223	1.275467	-1.11507	0.264822	0.704701
lncRNA:CR	1521.958	1.00277	0.899114	1.115287	0.264728	0.704701
CG4962	155.474	-3.6254	3.255713	-1.11355	0.265472	0.704701
CG32544	1656.96	-0.9135	0.820807	-1.11293	0.265738	0.705063
Prx3	2582.559	0.974911	0.876683	1.112045	0.266119	0.705839
CG17294	1368.364	-1.07488	0.9675	-1.11098	0.266575	0.705883
CG10444	157.28	-2.92566	2.633136	-1.11109	0.266528	0.705883
Hacl	493.9254	2.248623	2.023413	1.111302	0.266438	0.705883
NitFhit	2074.605	0.834307	0.750589	1.111535	0.266338	0.705883
NO66	260.9554	-2.50538	2.25421	-1.11142	0.266386	0.705883
mip120	386.9731	-2.36905	2.133072	-1.11063	0.266728	0.706031
udd	1072.975	1.043806	0.939989	1.110446	0.266807	0.706031
lncRNA:CR	250.1114	-2.30895	2.080439	-1.10984	0.26707	0.706494
ATP7	561.4912	-1.4973	1.349908	-1.10919	0.26735	0.707003
slo	20086.97	0.46791	0.422077	1.10859	0.267607	0.707448
CG17544	1569.455	1.041814	0.940093	1.108203	0.267774	0.707449
l(3)05822	4657.461	0.831718	0.750525	1.108181	0.267784	0.707449
CG3630	169.4214	-3.12177	2.821197	-1.10654	0.268493	0.707924
CG5886	1030.859	-1.67573	1.515341	-1.10585	0.268793	0.707924
CG15099	4837.017	0.780555	0.705559	1.106293	0.2686	0.707924
Drl-2	993.4645	1.273117	1.151047	1.106051	0.268704	0.707924
Arl4	13662.08	0.534483	0.482978	1.106642	0.268449	0.707924
PRAS40	2036.99	1.00315	0.9063	1.106863	0.268353	0.707924
Pdcd4	5113.242	0.60161	0.543935	1.106034	0.268712	0.707924
Idh3a	5376.747	-0.82774	0.747963	-1.10666	0.268441	0.707924
simj	2601.226	0.74745	0.675981	1.105726	0.268845	0.707924
Cdc27	762.2309	1.663808	1.502295	1.107511	0.268073	0.707924
CG5404	6370.192	0.754131	0.682588	1.104812	0.269241	0.708269

Or43b	234.8233	2.43051	2.199728	1.104914	0.269197	0.708269
l(3)72Ab	1327.119	-0.95604	0.865076	-1.10515	0.269096	0.708269
Sf3b2	973.5914	1.520835	1.377201	1.104294	0.269466	0.708628
tzn	966.8604	-1.11275	1.007896	-1.10403	0.269578	0.708691
GlyS	6100.48	-0.64631	0.585542	-1.10378	0.26969	0.708753
CG10628	574.2077	2.282717	2.068535	1.103542	0.269792	0.708788
fray	5846.944	0.623609	0.565538	1.102682	0.270165	0.709305
pAbp	17163.81	-0.48644	0.441092	-1.10281	0.270108	0.709305
swm	1255.28	1.226833	1.113277	1.102001	0.270461	0.70985
pins	1432.608	1.324216	1.20194	1.101732	0.270578	0.709925
Gyg	1865.013	-0.84119	0.763897	-1.10118	0.270817	0.710169
Neurl4	685.2491	-1.63395	1.483909	-1.10111	0.270848	0.710169
U2af38	976.4414	1.480858	1.346915	1.099445	0.271574	0.711013
Dh44-R2	1270.833	-1.03999	0.945626	-1.09979	0.271425	0.711013
ACOX1	918.2232	1.651811	1.502118	1.099654	0.271483	0.711013
Torsin	736.9198	-1.86683	1.69753	-1.09973	0.271449	0.711013
sli	4914.271	-0.69567	0.632802	-1.09936	0.271613	0.711013
CG4266	1290.257	-0.99843	0.909234	-1.0981	0.27216	0.712212
CG33469	91.60465	-4.21538	3.8425	-1.09704	0.272624	0.713194
CG14478	6045.978	-0.62128	0.566658	-1.09639	0.272909	0.713371
CG13325	233.0095	-2.77658	2.532741	-1.09628	0.272958	0.713371
yps	4925.613	0.624602	0.569586	1.096588	0.272822	0.713371
CG46339	3317.155	0.750969	0.685193	1.095997	0.27308	0.713457
Nhe1	1613.384	-1.60256	1.464662	-1.09415	0.273888	0.714333
CG7166	5150.843	0.591815	0.541058	1.09381	0.274038	0.714333
spz	1812.45	1.781292	1.627811	1.094287	0.273829	0.714333
Gcn2	1088.497	-1.59036	1.453261	-1.09434	0.273807	0.714333
Orai	1502.846	0.970544	0.887202	1.093938	0.273982	0.714333
lark	2719.985	-1.02765	0.939479	-1.09385	0.274022	0.714333
mRpL34	898.1431	1.185503	1.083098	1.094548	0.273715	0.714333
Usf	2391.433	0.839029	0.767353	1.093407	0.274215	0.714562
CG7789	861.8082	1.344443	1.229955	1.093083	0.274357	0.714566
pigs	4271.437	-0.71469	0.653879	-1.093	0.274395	0.714566
Chsy	1887.547	0.91983	0.84187	1.092603	0.274568	0.714785
CG12947	383.3821	-1.88953	1.73137	-1.09135	0.275118	0.715617
Cp190	2447.32	0.791586	0.725262	1.091449	0.275076	0.715617
CG13689	375.7243	1.725561	1.581528	1.091072	0.275241	0.715617
CG13539	278.179	-2.77705	2.545263	-1.09107	0.275244	0.715617
ssx	4404.549	-0.62843	0.576615	-1.08985	0.275778	0.716299
ihog	321.9294	-2.99005	2.745873	-1.08893	0.276187	0.716299
CG10694	208.7764	-3.22255	2.959594	-1.08885	0.276221	0.716299
CG10151	257.9799	-2.30785	2.117745	-1.08977	0.275815	0.716299
SIFaR	1115.648	1.143062	1.049599	1.089046	0.276133	0.716299
RpL30	6722.976	0.659166	0.604886	1.089736	0.275829	0.716299
Ca-beta	4557.56	-0.61664	0.566312	-1.08887	0.276213	0.716299
CG9018	786.0031	1.420765	1.304601	1.089042	0.276136	0.716299
Dpm1	632.098	1.318237	1.211083	1.088478	0.276384	0.716366
CG12343	1339.14	-1.56866	1.441276	-1.08839	0.276425	0.716366
CG17896	4854.118	-0.74275	0.682778	-1.08784	0.276667	0.716762
RhoGAP92	1270.613	1.600579	1.473851	1.085985	0.277486	0.717957

Golgin84	777.8005	-1.61804	1.489682	-1.08617	0.277405	0.717957
ras	3633.619	-1.32772	1.222033	-1.08648	0.277266	0.717957
tfc	163.8964	-3.15083	2.900335	-1.08637	0.277317	0.717957
Bx	9750.46	0.514408	0.473788	1.085735	0.277596	0.718011
TBCB	836.28	-1.16441	1.072922	-1.08527	0.277803	0.718313
CR12842	802.773	1.604807	1.479282	1.084856	0.277986	0.718555
CG4686	779.3398	-1.30302	1.201366	-1.08462	0.278092	0.718598
CG2162	1078.4	1.142345	1.05387	1.083953	0.278386	0.719126
RtGEF	1817.223	0.839517	0.775023	1.083215	0.278713	0.719276
Hel89B	1308.824	0.93572	0.863717	1.083365	0.278647	0.719276
l(2)k09848	632.0946	1.719376	1.587123	1.083329	0.278663	0.719276
Cbp53E	14674.93	0.523182	0.483252	1.082628	0.278974	0.719485
Rac1	3144.932	-0.73511	0.679129	-1.08243	0.279063	0.719485
Pino	10863.5	0.502745	0.464336	1.082718	0.278933	0.719485
CG9377	1901.395	-0.88911	0.821776	-1.08194	0.27928	0.719814
Smg5	2614.35	-0.7663	0.708528	-1.08154	0.279457	0.720039
CG31673	1434.034	-1.07833	0.997738	-1.08078	0.279795	0.720337
snoRNA:2F	140.0952	2.578249	2.385777	1.080675	0.279842	0.720337
fln	1448.676	-1.06082	0.981382	-1.08094	0.279722	0.720337
RpS18	9912.037	0.512745	0.474577	1.080425	0.279953	0.720372
Cyp6a14	163.9723	3.423724	3.170297	1.079938	0.28017	0.720372
rho-5	2121.433	-0.89264	0.826527	-1.07999	0.280148	0.720372
asRNA:CR4	2852.355	0.954737	0.884314	1.079636	0.280304	0.720372
Prps	4671.652	-0.60302	0.558532	-1.07966	0.280294	0.720372
PGRP-LE	623.4578	-1.25505	1.163388	-1.07879	0.280682	0.721113
CG3308	1101.102	-1.52319	1.412357	-1.07847	0.280822	0.721241
ArgRS-m	199.0061	-2.56262	2.377373	-1.07792	0.281069	0.721415
CG4080	2192.001	0.774984	0.718837	1.078109	0.280985	0.721415
GluClalpha	23228.57	0.474923	0.440749	1.077538	0.28124	0.721622
CG12608	1275.797	-1.55577	1.444941	-1.0767	0.281615	0.721962
mop	3552.191	-0.69047	0.64132	-1.07664	0.281643	0.721962
slgA	15815.02	0.52772	0.490037	1.076897	0.281526	0.721962
Atg16	605.5786	-1.93308	1.795845	-1.07642	0.281742	0.721985
scaRNA:M	189.3659	-2.10019	1.952812	-1.07547	0.282164	0.722837
CG3420	2030.224	0.846317	0.787165	1.075146	0.282309	0.722864
lncRNA:CR	650.459	-1.92875	1.794112	-1.07504	0.282355	0.722864
Act5C	51633.31	-0.42437	0.39495	-1.07449	0.282605	0.723275
Efa6	5079.927	-0.58047	0.540358	-1.07423	0.28272	0.723338
CG1815	666.4409	1.376181	1.2822	1.073296	0.283138	0.723715
shakB	16954	0.587676	0.547416	1.073545	0.283026	0.723715
CG11696	569.947	-1.7441	1.624901	-1.07335	0.283112	0.723715
CG14312	846.7276	1.176243	1.096696	1.072533	0.28348	0.72411
r	168.1575	-3.00665	2.803688	-1.07239	0.283544	0.72411
CG17598	662.8446	1.430529	1.334014	1.072349	0.283563	0.72411
Ipk2	264.1488	-2.28571	2.132366	-1.07191	0.283758	0.724148
Arf79F	10763.87	0.483467	0.450987	1.072021	0.283711	0.724148
CG1671	694.5633	-1.59261	1.486345	-1.07149	0.283947	0.724399
GstS1	5219.636	-0.64245	0.600094	-1.07059	0.284355	0.725026
enok	1961.959	-0.79285	0.740604	-1.07055	0.284373	0.725026
Esyt2	3257.601	0.740307	0.691707	1.070261	0.284502	0.725123

Nf-YB	1729.036	-0.89601	0.837904	-1.06935	0.284913	0.72571
CG9394	59192.99	-0.54896	0.513319	-1.06944	0.284871	0.72571
Rcd-1	2467.723	-0.77628	0.726072	-1.06915	0.285003	0.72571
CG7655	284.3944	2.060468	1.92798	1.068719	0.285196	0.725971
Pi3K21B	1514.511	-1.06379	0.995937	-1.06813	0.285462	0.726418
CoRest	4771.936	-0.84471	0.791428	-1.06733	0.285824	0.727106
CG16890	296.3267	-2.07148	1.941675	-1.06685	0.286039	0.727423
CG12320	160.5985	3.18552	2.988652	1.065872	0.286482	0.728088
ClC-c	2045.049	1.139768	1.069164	1.066036	0.286407	0.728088
sif	12702.29	0.467974	0.439221	1.065464	0.286666	0.728096
CG5644	1135.773	-1.39808	1.312118	-1.06551	0.286645	0.728096
RhoBTB	3871.33	0.942234	0.88464	1.065105	0.286829	0.728255
colt	707.7339	1.343711	1.26179	1.064924	0.28691	0.728255
ena	4760.36	-0.73632	0.691577	-1.0647	0.287013	0.728286
Hsepi	600.9777	-1.93244	1.816364	-1.0639	0.287372	0.728641
CycJ	692.1397	1.30947	1.230758	1.063954	0.287349	0.728641
Nopp140	659.4827	1.291357	1.21402	1.063703	0.287463	0.728641
312	776.2467	1.598682	1.503105	1.063586	0.287516	0.728641
PlexB	3773.142	0.75327	0.708665	1.062943	0.287808	0.729149
bon	1033.667	-1.09996	1.035352	-1.0624	0.288055	0.729545
CG3939	751.6175	-1.66636	1.569291	-1.06186	0.288301	0.729857
ATPsynG	25773.17	0.447657	0.421711	1.061526	0.288451	0.729857
CG1688	308.5203	-1.94853	1.835323	-1.06168	0.28838	0.729857
CG8245	11053.05	0.535781	0.505238	1.060453	0.288939	0.730649
CG1598	1913.308	-0.99975	0.942773	-1.06044	0.288946	0.730649
fs(1)K10	79.58536	3.150344	2.975348	1.058815	0.289684	0.731182
CG31908	438.1562	2.10211	1.983986	1.059539	0.289355	0.731182
CG9386	199.6294	-2.46981	2.33271	-1.05877	0.289703	0.731182
CG4049	3841.003	-0.64087	0.605285	-1.0588	0.289692	0.731182
eIF6	623.3093	1.611278	1.521313	1.059137	0.289538	0.731182
coro	990.3169	1.454481	1.373344	1.05908	0.289563	0.731182
Msr-110	48799.58	-0.44654	0.422169	-1.05773	0.290178	0.73215
CG5516	434.0268	1.80535	1.707502	1.057305	0.290373	0.73217
Rpn7	1892.644	0.794975	0.752113	1.056988	0.290517	0.73217
SMC1	698.4319	1.667807	1.578295	1.056714	0.290642	0.73217
Tdc2	1302.539	-1.27396	1.205198	-1.05706	0.290486	0.73217
Smn	592.3496	-2.27373	2.151481	-1.05682	0.290594	0.73217
CG13516	269.9987	-2.45635	2.327187	-1.0555	0.291197	0.732741
l(3)76BDm	1433.961	1.055825	0.999986	1.05584	0.291041	0.732741
lncRNA:CR	1642.253	0.871737	0.82596	1.055422	0.291232	0.732741
CG15628	5549.737	-0.56204	0.532528	-1.05542	0.291234	0.732741
CG11377	1119.694	1.735627	1.644987	1.055101	0.291379	0.732877
P32	3498.445	0.873194	0.828149	1.054392	0.291703	0.733462
Cyp313a1	136.0311	-3.5413	3.361303	-1.05355	0.292089	0.733973
CG17486	2121.32	-1.33797	1.269757	-1.05372	0.29201	0.733973
Dscam3	2582.033	0.717874	0.681633	1.053168	0.292264	0.734182
mAChR-A	7416.16	0.533891	0.507473	1.052059	0.292773	0.735229
CG9003	7000.316	0.650099	0.618611	1.050901	0.293304	0.735956
sog	201.4717	2.594135	2.467843	1.051175	0.293178	0.735956
betaCOP	1106.389	-1.41663	1.34811	-1.05083	0.293337	0.735956

CG11788	272.6797	-2.80431	2.670499	-1.05011	0.293669	0.736559
Pex11	730.0974	1.450944	1.382882	1.049217	0.294078	0.737355
CG10425	81.69785	3.139596	2.994154	1.048575	0.294374	0.737634
Tis11	937.6043	1.717918	1.638211	1.048655	0.294337	0.737634
CG31705	26501.34	-0.5234	0.499436	-1.04798	0.294646	0.737763
mats	771.1803	-1.21861	1.163544	-1.04732	0.29495	0.737763
wde	3947.246	-0.72469	0.691844	-1.04748	0.294877	0.737763
Iml1	1488.581	1.01647	0.970593	1.047267	0.294977	0.737763
CG8993	2800.015	-0.69518	0.663618	-1.04755	0.294844	0.737763
CG4829	10586.97	-0.60353	0.576117	-1.04758	0.29483	0.737763
trr	190.3551	2.812927	2.687485	1.046676	0.295249	0.737964
CG6700	6277.862	0.614922	0.587493	1.046689	0.295243	0.737964
Atg2	560.1758	-1.57224	1.502389	-1.04649	0.295333	0.737964
CG3556	11642.91	0.590554	0.564682	1.045816	0.295646	0.738517
drpr	15961.65	0.47681	0.456753	1.043911	0.296526	0.740486
Pgm2a	708.7552	-1.3843	1.327165	-1.04305	0.296926	0.741252
CG4615	2381.917	-0.79471	0.762816	-1.04181	0.297498	0.74245
bchs	2643.594	0.781157	0.750401	1.040986	0.297882	0.742839
CG42239	698.9471	1.641722	1.577246	1.040879	0.297932	0.742839
lncRNA:CR	179.8145	2.157371	2.072447	1.040978	0.297886	0.742839
E(Pc)	4402.451	0.645046	0.620011	1.040378	0.298164	0.743189
vig2	1928.827	-0.80044	0.770847	-1.03839	0.29909	0.744958
CG11200	225.9672	-2.59554	2.499759	-1.03832	0.299122	0.744958
zfh2	50246	-0.44839	0.43187	-1.03825	0.299153	0.744958
CG31637	3818.172	0.66876	0.644535	1.037585	0.299463	0.7455
CG3587	271.2875	-2.77154	2.672309	-1.03713	0.299674	0.745794
Cse1	541.8097	1.671657	1.612298	1.036816	0.299821	0.745801
ort	200.8197	-2.65418	2.560151	-1.03673	0.299863	0.745801
Ktl	9573.36	0.619362	0.597951	1.035808	0.300292	0.746387
Aladin	307.7817	-2.14757	2.073639	-1.03565	0.300365	0.746387
CG11073	215.184	2.405067	2.322337	1.035624	0.300378	0.746387
CG31638	2259.446	-1.03435	0.999827	-1.03453	0.300891	0.747431
CG31910	95.25486	4.002026	3.875159	1.032739	0.301726	0.747834
tun	2618.248	0.735607	0.712531	1.032386	0.301891	0.747834
CG42394	2642.43	0.742851	0.71886	1.033374	0.301429	0.747834
Cap-G	503.8116	-2.01067	1.945098	-1.03371	0.301272	0.747834
cdm	1581.552	1.035663	1.003144	1.032417	0.301877	0.747834
CG43788	409.7333	-1.9854	1.921866	-1.03306	0.301575	0.747834
CG1572	748.7853	-2.16843	2.099372	-1.0329	0.301652	0.747834
GstT4	770.8282	1.172374	1.134982	1.032945	0.301629	0.747834
CG7889	215.6947	-2.77988	2.689855	-1.03347	0.301385	0.747834
lncRNA:CR	4413.864	-0.5875	0.569459	-1.03168	0.302223	0.748423
Rabex-5	203.3906	2.40469	2.33683	1.029039	0.303461	0.751258
CG30285	4897.681	0.652701	0.635496	1.027073	0.304386	0.753316
Lrr47	819.5136	-1.36682	1.332663	-1.02563	0.305065	0.754762
Efr	2986.325	-0.92883	0.90603	-1.02516	0.305287	0.754865
CG7222	2388.282	0.723258	0.705517	1.025146	0.305294	0.754865
jtb	300.4307	-2.63185	2.568261	-1.02476	0.305476	0.755083
Bre1	1383.993	-1.06728	1.041959	-1.0243	0.305693	0.755154
CG15201	8879.216	0.601343	0.58702	1.024399	0.305647	0.755154

Gp93	2426.25	0.751716	0.734045	1.024074	0.3058	0.755186
lncRNA:CR	664.1754	1.568512	1.534148	1.0224	0.306592	0.756907
kek6	1388.883	1.168485	1.143997	1.021405	0.307063	0.757603
CG2116	269.9773	2.689626	2.632949	1.021526	0.307005	0.757603
CG7702	177.9236	-3.30506	3.240079	-1.02005	0.307703	0.758555
rho	8973.304	-0.55802	0.547076	-1.02001	0.307726	0.758555
CG7536	1302.475	-1.09514	1.073669	-1.01999	0.307732	0.758555
CG43867	5977.408	-0.55348	0.543559	-1.01825	0.308559	0.760126
lncRNA:CR	1527.315	-0.86257	0.846953	-1.01844	0.308467	0.760126
CG17528	9104.152	0.552521	0.542768	1.01797	0.308692	0.760222
TTLL5	963.5054	1.292797	1.270452	1.017588	0.308874	0.760436
CG13192	562.1521	1.873688	1.841961	1.017225	0.309047	0.760628
B4	4280.315	0.673598	0.662385	1.016929	0.309187	0.760741
CG10019	222.2548	-1.97638	1.94476	-1.01626	0.309507	0.761293
Ufl1	3739.857	-1.13471	1.117046	-1.01582	0.309717	0.761579
CycD	632.001	-2.01469	1.985764	-1.01457	0.310311	0.762805
CG2685	487.5156	1.958461	1.931269	1.01408	0.310545	0.763145
mRpL43	1120.938	0.932709	0.920224	1.013567	0.310789	0.763512
CG6154	795.7913	-1.33012	1.313646	-1.01254	0.311279	0.764073
CG1523	337.0942	1.586737	1.567161	1.012492	0.311303	0.764073
CG8323	1379.602	-1.46182	1.4435	-1.01269	0.311207	0.764073
CG31388	1814.003	-0.9285	0.917527	-1.01195	0.31156	0.764469
Nepl4	132.7151	-3.03359	3.002371	-1.0104	0.312305	0.765668
RyR	2173.731	0.856239	0.84727	1.010586	0.312215	0.765668
SmB	1248.628	-0.88981	0.880709	-1.01034	0.312335	0.765668
dbr	1021.124	1.144736	1.133267	1.01012	0.312438	0.765687
CG8668	1418.402	-1.09251	1.081855	-1.00985	0.312567	0.76577
CG3436	1144.518	1.060832	1.054212	1.00628	0.314281	0.766131
CG11601	196.4442	2.536583	2.518831	1.007048	0.313912	0.766131
CG13117	383.9642	-2.69357	2.672736	-1.0078	0.313552	0.766131
CG6443	1050.021	-1.05298	1.046264	-1.00642	0.314212	0.766131
CG5270	899.1843	1.101201	1.093627	1.006926	0.31397	0.766131
CG14883	1055.96	1.319954	1.311892	1.006145	0.314346	0.766131
mod	1890.84	0.800251	0.79487	1.00677	0.314045	0.766131
Pkn	4866.679	-0.63729	0.632045	-1.00831	0.313308	0.766131
CG14664	547.1656	-2.70368	2.68474	-1.00706	0.313908	0.766131
Nost	880.7021	1.037735	1.028709	1.008774	0.313083	0.766131
CG42700	198.1141	2.957735	2.936167	1.007346	0.313769	0.766131
fwd	8026.003	-0.6413	0.636745	-1.00715	0.313864	0.766131
CR43670	720.4428	1.394704	1.38281	1.008601	0.313166	0.766131
EloC	5192.527	-0.569	0.564346	-1.00824	0.313339	0.766131
und	1397.378	1.318375	1.310558	1.005965	0.314432	0.766131
bol	19976.67	0.438734	0.435944	1.006401	0.314223	0.766131
CG9953	4279.662	-0.79572	0.790769	-1.00626	0.314292	0.766131
lilli	9136.666	0.488293	0.485194	1.006387	0.314229	0.766131
CG15631	235.7641	2.39362	2.380608	1.005466	0.314672	0.766483
Nrg	4883.509	0.661767	0.658357	1.005179	0.31481	0.766586
CG12121	1365.814	-0.99846	0.994683	-1.0038	0.315474	0.76797
mib1	967.507	-1.11005	1.106277	-1.00341	0.315665	0.768201
Alg1	504.9376	-2.26636	2.260839	-1.00244	0.316131	0.768791

CG31997	1333.734	-1.1656	1.163021	-1.00221	0.31624	0.768791
Rab32	297.86	-2.34538	2.340446	-1.00211	0.316291	0.768791
CG15601	417.7284	2.048268	2.043474	1.002346	0.316176	0.768791
Sh	59029.1	0.394309	0.394073	1.000599	0.317021	0.770131
ArgRS	444.8042	2.051703	2.050529	1.000572	0.317034	0.770131
CG2841	774.9344	1.120787	1.120464	1.000288	0.317171	0.770231
CG4660	883.5352	1.044745	1.044674	1.000067	0.317278	0.770258
Jafrac1	9950.707	-0.45825	0.458514	-0.99943	0.317585	0.770303
Nup35	273.4787	-2.36104	2.362169	-0.99952	0.317541	0.770303
CG11279	1154.59	0.98853	0.988865	0.999661	0.317474	0.770303
axo	9942.72	-0.48714	0.487621	-0.999	0.317793	0.770553
CG13585	4525.553	0.557164	0.55782	0.998825	0.31788	0.770553
Tctp	16491.52	0.483332	0.484035	0.998547	0.318014	0.770646
CG31715	1929.817	0.75275	0.754098	0.998211	0.318177	0.770808
CG6765	1403.608	1.016528	1.018717	0.997851	0.318352	0.770999
CG14695	114.124	3.325855	3.335313	0.997164	0.318685	0.771151
CG30496	553.3259	1.546773	1.551703	0.996823	0.318851	0.771151
jar	4080.747	-0.64124	0.643321	-0.99677	0.318876	0.771151
RpS24	16551.02	0.482059	0.48364	0.996731	0.318895	0.771151
NetB	3239.737	-0.65378	0.655824	-0.99688	0.318823	0.771151
lncRNA:CR	461.6047	2.2927	2.300727	0.996511	0.319002	0.771178
Ten-m	21062.83	0.425777	0.427377	0.996255	0.319126	0.771246
cmpy	3339.862	0.785183	0.78889	0.995301	0.31959	0.77156
CG6770	13660.55	-0.42937	0.431524	-0.995	0.319737	0.77156
Patj	1073.839	-1.19193	1.197263	-0.99554	0.319471	0.77156
CG7275	569.252	-1.45429	1.461369	-0.99516	0.319659	0.77156
CG32085	4017.657	-0.75728	0.76096	-0.99517	0.319654	0.77156
CadN	11878.78	0.442112	0.444824	0.993903	0.32027	0.772614
bbx	126.4531	2.499608	2.519625	0.992055	0.321171	0.774175
Maf1	9214.972	0.522287	0.526614	0.991784	0.321303	0.774175
CG8814	1829.838	1.233518	1.243132	0.992266	0.321068	0.774175
CG15356	2224.198	-0.74205	0.748181	-0.9918	0.321293	0.774175
CG42337	2619.748	0.755072	0.761592	0.991438	0.321472	0.774349
HERC2	1862.131	-1.00661	1.015513	-0.99124	0.321571	0.774356
CG11398	420.5757	1.991638	2.010456	0.99064	0.321861	0.774731
CG7115	1030.699	1.277678	1.289905	0.990521	0.32192	0.774731
CG13384	4309.683	-0.60044	0.607349	-0.98862	0.322851	0.774784
Acer	307.0826	-1.96952	1.992918	-0.98826	0.323027	0.774784
CG4972	3438.071	-0.658	0.666335	-0.98749	0.323404	0.774784
Coq3	104.519	-3.10153	3.136369	-0.98889	0.322717	0.774784
sle	537.5075	1.411299	1.427229	0.988839	0.322742	0.774784
spab	9430.801	-0.52226	0.528614	-0.98798	0.323161	0.774784
Gat	30049.02	-0.49097	0.497474	-0.98693	0.323679	0.774784
Etl1	830.2061	-1.51699	1.532412	-0.98994	0.322205	0.774784
CG40191	8118.725	-0.73742	0.747	-0.98717	0.32356	0.774784
snoRNA:Ps	89.91511	3.79743	3.845043	0.987617	0.32334	0.774784
east	3667.103	-0.66903	0.676377	-0.98913	0.322597	0.774784
twr	1121.147	-0.99623	1.008437	-0.9879	0.323202	0.774784
Snap29	866.81	-1.55587	1.576481	-0.98693	0.323679	0.774784
Alp2	277.8794	2.991595	3.028312	0.987876	0.323214	0.774784

hpRNA:CR:	132.7505	-2.39816	2.422188	-0.99008	0.322135	0.774784
Tango1	677.6138	1.495145	1.513957	0.987574	0.323361	0.774784
CG32650	140.9033	-3.68974	3.738253	-0.98702	0.323632	0.774784
CG12355	8809.196	0.502938	0.508635	0.988798	0.322762	0.774784
l(2)01289	128.4773	-2.44299	2.477181	-0.9862	0.324036	0.774943
sns	719.2938	-1.49641	1.517117	-0.98635	0.323961	0.774943
CG32264	14822.7	0.451902	0.458082	0.98651	0.323883	0.774943
Sirt7	447.6771	1.978627	2.007124	0.985802	0.32423	0.775178
mago	923.6279	1.379238	1.399877	0.985257	0.324498	0.775587
CG11970	594.1757	-1.51167	1.536748	-0.98368	0.325272	0.776543
aPKC	5814.562	-0.56483	0.573939	-0.98413	0.32505	0.776543
RASSF8	2294.576	0.937549	0.953163	0.983618	0.325303	0.776543
Su(P)	1462.073	0.864892	0.878929	0.984029	0.325101	0.776543
Prp40	438.121	-1.5318	1.557564	-0.98346	0.325382	0.776543
lncRNA:CR	50381.63	0.389583	0.396295	0.983063	0.325577	0.776777
barc	1306.891	0.913471	0.929734	0.982508	0.32585	0.776812
CG8064	213.1209	2.56656	2.611879	0.982649	0.32578	0.776812
CG8188	3924.605	0.830794	0.846002	0.982024	0.326088	0.776812
AIMP3	1405.553	0.875547	0.891608	0.981986	0.326107	0.776812
Pep	5845.161	0.521669	0.53131	0.981854	0.326172	0.776812
AIMP2	1528.752	-1.19286	1.214272	-0.98237	0.325919	0.776812
Pfas	144.0031	-2.83581	2.896648	-0.979	0.327581	0.778718
lp259	8044.782	-0.5481	0.559945	-0.97885	0.327655	0.778718
RpL9	11757.99	0.464393	0.474589	0.978517	0.327819	0.778718
Teh1	2927.355	-0.67985	0.694274	-0.97922	0.327471	0.778718
Cyp9f2	6784.522	-0.62595	0.639284	-0.97914	0.327512	0.778718
CG3107	6396.059	0.556193	0.568435	0.978463	0.327846	0.778718
sPLA2	1427.616	0.936365	0.955778	0.979688	0.32724	0.778718
PsGEF	4857.382	0.589826	0.602439	0.979064	0.327548	0.778718
CG44153	1321.616	-0.92565	0.945948	-0.97854	0.327807	0.778718
Rnb	313.0079	-1.66568	1.703797	-0.97763	0.328257	0.779444
Fem-1	3358.855	-0.63142	0.645986	-0.97745	0.328346	0.779444
per	285.824	2.094103	2.157117	0.970788	0.331654	0.779521
brn	682.9514	-1.70837	1.758883	-0.97128	0.331407	0.779521
Tbc1d8-9	2387.487	0.682799	0.70285	0.971472	0.331313	0.779521
Srpk79D	2955.467	-0.68507	0.705725	-0.97074	0.33168	0.779521
CG12170	1020.915	-0.96795	0.992433	-0.97533	0.329399	0.779521
CG8159	278.8926	-1.87848	1.930481	-0.97306	0.330522	0.779521
alpha-Man	2052.427	1.219574	1.255788	0.971163	0.331467	0.779521
CG8031	1145.305	-1.25786	1.29569	-0.9708	0.331647	0.779521
Usp8	1223.225	-0.9634	0.991071	-0.97208	0.33101	0.779521
sba	1767.138	0.861447	0.885768	0.972543	0.33078	0.779521
twin	1755.708	0.943708	0.965972	0.976952	0.328593	0.779521
CG13436	76.3605	-3.62774	3.73614	-0.97099	0.331555	0.779521
Lrt	466.5154	1.345004	1.382163	0.973116	0.330496	0.779521
Pcl	2171.092	1.089045	1.117436	0.974593	0.329762	0.779521
Bap111	483.439	1.779212	1.831456	0.971474	0.331312	0.779521
Sema2a	2733.451	-0.73054	0.74849	-0.97602	0.329053	0.779521
bdg	485.1129	1.741628	1.785461	0.97545	0.329337	0.779521
CG8547	1994.898	-1.48378	1.52783	-0.97117	0.331466	0.779521

rho-7	471.2395	2.044224	2.104164	0.971513	0.331293	0.779521
VGAT	1884.953	-0.98109	1.009345	-0.972	0.331049	0.779521
CG33643	130.1063	2.691455	2.758003	0.975871	0.329129	0.779521
Mob3	2699.574	-1.20512	1.239103	-0.97258	0.330764	0.779521
CG30172	8278.321	0.508866	0.523834	0.971425	0.331336	0.779521
Mtch	3915.145	0.738163	0.758381	0.973341	0.330384	0.779521
fok	16275.49	-0.42723	0.438421	-0.97448	0.329816	0.779521
Snap24	774.2673	1.148601	1.183053	0.970878	0.331609	0.779521
Gk2	316.041	-2.44307	2.510278	-0.97323	0.330441	0.779521
ecd	1090.773	1.389808	1.423814	0.976116	0.329007	0.779521
CG9911	2043.264	0.955557	0.978092	0.97696	0.328589	0.779521
CG6878	2613.079	0.662734	0.680696	0.973613	0.330249	0.779521
defl	355.9789	2.333383	2.401376	0.971686	0.331207	0.779521
Pop4	552.7143	-1.60532	1.652748	-0.9713	0.331398	0.779521
Mis12	673.997	-1.70365	1.747957	-0.97465	0.329732	0.779521
CG12576	4292.543	0.574188	0.59091	0.971701	0.331199	0.779521
cact	2351.531	0.67174	0.692201	0.970441	0.331827	0.779637
CG33947	396.5404	2.13115	2.197097	0.969985	0.332054	0.779715
Rpn12	1524.612	-0.86898	0.895734	-0.97013	0.33198	0.779715
chico	1288.451	0.901051	0.929705	0.969179	0.332456	0.780199
CG6204	249.2087	2.815727	2.907016	0.968597	0.332746	0.780199
CG5504	868.4026	1.046122	1.079757	0.968849	0.332621	0.780199
CG34457	193.3199	-3.29586	3.402046	-0.96879	0.332651	0.780199
Gfrl	8525.677	-0.5324	0.549477	-0.96892	0.332587	0.780199
Dredd	670.8331	-1.55046	1.602612	-0.96746	0.333316	0.781128
Pi4KIIIalph	2177.119	-0.90288	0.933296	-0.96741	0.333337	0.781128
Nmdar2	3675.405	0.60761	0.628333	0.967019	0.333534	0.781362
CG30069	5374.633	-0.66554	0.688514	-0.96663	0.333727	0.781586
Uhg1	2473.373	0.715965	0.741226	0.965921	0.334084	0.781965
Invadolysi	13578.23	-0.47094	0.487555	-0.96592	0.334084	0.781965
rux	437.9626	-1.72079	1.782866	-0.96518	0.334454	0.782375
l(2)09851	617.2142	-1.17628	1.218524	-0.96533	0.334379	0.782375
CG14231	77.65593	-3.21534	3.333794	-0.96447	0.334812	0.782983
TER94	2683.054	0.638256	0.661922	0.964247	0.334922	0.783014
Rab18	1600.17	-0.78393	0.814582	-0.96238	0.335861	0.783295
CG11247	3923.313	-0.67849	0.704787	-0.96269	0.335704	0.783295
Xe7	7186.024	-0.48453	0.504808	-0.95983	0.337141	0.783295
Cka	3496.842	-0.70343	0.729921	-0.96371	0.335192	0.783295
CG5676	8824.829	0.47732	0.49676	0.960866	0.336619	0.783295
CG13625	152.121	2.137926	2.223751	0.961406	0.336348	0.783295
CG14543	347.6376	-1.6987	1.765405	-0.96221	0.335942	0.783295
bys	648.469	-1.43807	1.495048	-0.96189	0.336106	0.783295
c12.1	1720.189	0.874416	0.907657	0.963377	0.335358	0.783295
CG11808	194.7298	2.524436	2.624834	0.961751	0.336175	0.783295
dmpd	641.866	-1.17081	1.219465	-0.9601	0.337005	0.783295
CG1371	1939.154	-0.89591	0.932897	-0.96035	0.336879	0.783295
LRP1	4106.536	0.617264	0.642875	0.960162	0.336974	0.783295
CG10973	1038.759	1.270647	1.323361	0.960167	0.336971	0.783295
dpr13	5405.654	0.554464	0.576173	0.962322	0.335888	0.783295
sra	2599.563	0.868595	0.901455	0.963548	0.335272	0.783295

CG3663	227.7863	2.198539	2.288417	0.960725	0.336691	0.783295
Nurf-38	4470.685	0.605314	0.629819	0.961091	0.336507	0.783295
Scsalpha1	10530.74	0.471963	0.491765	0.959733	0.33719	0.783295
CG7630	28065.69	0.399592	0.415041	0.962778	0.335659	0.783295
pbl	1623.153	1.186005	1.234211	0.960941	0.336582	0.783295
morgue	921.2938	-0.97696	1.017027	-0.9606	0.336753	0.783295
CG8270	748.6242	-1.51481	1.580375	-0.95851	0.337804	0.784495
Jwa	2588.54	0.712462	0.743819	0.957843	0.338142	0.784826
Pvf3	7191.96	0.576125	0.601403	0.957968	0.338079	0.784826
Marc	1201.302	0.868849	0.907682	0.957218	0.338457	0.785281
lncRNA:CR	2859.39	-0.67998	0.710486	-0.95707	0.338534	0.785281
CG13954	1674.842	0.906658	0.94774	0.956653	0.338742	0.785538
moody	9014.071	0.531349	0.555853	0.955916	0.339115	0.786145
CG16974	16866.44	-0.41001	0.429083	-0.95555	0.339298	0.786145
CG31473	126.5455	2.560164	2.679151	0.955588	0.33928	0.786145
mof	111.7639	-3.15263	3.304727	-0.95398	0.340095	0.787765
pasi2	1461.806	-1.56408	1.641063	-0.95309	0.340546	0.78858
pum	35339.09	-0.37825	0.397551	-0.95145	0.341377	0.789163
CG10898	447.0038	2.023388	2.124578	0.952371	0.340909	0.789163
omd	205.79	-2.17333	2.28399	-0.95155	0.341325	0.789163
CG7956	2213.184	0.90621	0.952269	0.951633	0.341283	0.789163
sinu	743.2687	1.256775	1.320935	0.951428	0.341387	0.789163
CG13921	1688.57	-0.94782	0.995921	-0.9517	0.341249	0.789163
CG16721	1105.238	-1.22753	1.292103	-0.95003	0.342098	0.789198
ZnT41F	704.7742	-1.73418	1.825545	-0.94995	0.342137	0.789198
CG32568	371.8308	2.250694	2.369569	0.949833	0.342197	0.789198
Proc-R	6965.35	-0.49534	0.521201	-0.95039	0.341913	0.789198
inv	65.53148	3.672518	3.865588	0.950054	0.342085	0.789198
CG31221	27894.87	0.425216	0.447516	0.95017	0.342026	0.789198
CG34136	296.1518	-2.01781	2.123376	-0.95028	0.341969	0.789198
Su(Tpl)	5896.179	-0.49502	0.520467	-0.95111	0.341548	0.789198
Srp54k	2570.91	0.915073	0.963585	0.949655	0.342287	0.789198
Cyp9h1	337.8307	-2.63063	2.772622	-0.94879	0.342729	0.789389
wash	197.9598	2.233203	2.353815	0.948759	0.342743	0.789389
Sox14	367.9939	-1.94292	2.047942	-0.94872	0.342764	0.789389
yki	2188.027	-1.04446	1.100605	-0.94899	0.342626	0.789389
Src64B	2413.692	-0.71874	0.757899	-0.94833	0.342963	0.789395
CG14414	1177.395	0.935253	0.986051	0.948484	0.342883	0.789395
Ada2b	173.6092	-2.56942	2.71041	-0.94798	0.343138	0.789572
ppk	161.4217	-3.06218	3.232133	-0.94742	0.343426	0.789742
CG6325	962.5465	-1.13338	1.196681	-0.9471	0.343587	0.789742
mbo	263.4695	-2.38211	2.514618	-0.94731	0.343483	0.789742
CG17684	6017.777	0.531651	0.561367	0.947065	0.343606	0.789742
Nplp1	3233.979	-0.84326	0.890903	-0.94652	0.343884	0.789929
CG10984	1828.592	0.797869	0.842885	0.946593	0.343846	0.789929
Nfs1	2929.359	0.761987	0.805671	0.94578	0.344261	0.790262
CG2608	802.7585	-1.40411	1.484796	-0.94566	0.344324	0.790262
Inx3	6084.472	-0.51892	0.548522	-0.94604	0.34413	0.790262
Top3alpha	191.9047	-2.46362	2.605942	-0.94539	0.344462	0.790353
CG9471	2340.681	-0.9456	1.001043	-0.94461	0.344858	0.790808

side-VI	4285.273	0.680478	0.720343	0.944658	0.344833	0.790808
sstn	569.1393	1.790459	1.895978	0.944346	0.344993	0.790892
Cog2	719.9508	1.367396	1.448393	0.944078	0.34513	0.79098
2mit	2503.635	0.73572	0.779608	0.943705	0.34532	0.791191
CG10376	1450.677	-0.89844	0.952466	-0.94328	0.34554	0.791468
RhoGAP1A	2046.275	-1.0059	1.066959	-0.94277	0.345797	0.791605
ALiX	2080.48	-0.96582	1.02429	-0.94292	0.345724	0.791605
bic	8940.618	0.558705	0.592975	0.942208	0.346086	0.791621
Drgx	1534.343	0.949953	1.008249	0.942181	0.3461	0.791621
CHMP2B	438.2548	-1.35289	1.43567	-0.94234	0.34602	0.791621
RpL18A	22538.68	0.418381	0.44429	0.941684	0.346354	0.791752
CG6707	3369.884	-0.63927	0.678732	-0.94186	0.346263	0.791752
26-29-p	4978.001	0.658146	0.699352	0.94108	0.346664	0.791783
CG12084	3991.605	-0.63519	0.674896	-0.94117	0.346616	0.791783
sd	4141.405	0.540568	0.574385	0.941126	0.34664	0.791783
Khc-73	4839.424	-0.53457	0.568389	-0.94049	0.346964	0.791792
lncRNA:CR	108.0571	2.723258	2.895002	0.940676	0.346871	0.791792
iPLA2-VIA	1532.416	0.868402	0.923193	0.940651	0.346884	0.791792
Ldsdh1	2547.727	-0.67269	0.715629	-0.94	0.347215	0.792141
Dpck	1756.01	0.779651	0.829955	0.939389	0.347531	0.792418
Art2	122.0676	-2.83029	3.012928	-0.93938	0.347534	0.792418
CG1139	792.7481	1.839664	1.958791	0.939183	0.347637	0.792426
CG33143	1952.745	0.815665	0.868734	0.938913	0.347776	0.792518
retm	5557.421	-0.51156	0.545343	-0.93805	0.348219	0.793303
mRpS16	1332.72	0.884325	0.943469	0.937312	0.348598	0.793941
CG12746	1103.998	-1.01074	1.079822	-0.93603	0.349259	0.794462
Sps2	626.296	-1.5735	1.681526	-0.93576	0.349397	0.794462
CG5787	1857.49	1.220095	1.303562	0.93597	0.349289	0.794462
CG5285	371.6998	1.501171	1.603003	0.936474	0.349029	0.794462
asrij	624.3389	1.540914	1.64673	0.935741	0.349406	0.794462
hiw	3005.133	0.768892	0.821718	0.935713	0.349421	0.794462
CG4036	391.0741	1.456335	1.55837	0.934524	0.350033	0.795618
bsk	12411.32	-0.43099	0.461343	-0.93421	0.350198	0.795618
CG5214	6675.002	-0.6662	0.71316	-0.93415	0.350226	0.795618
CG13392	469.3546	1.852757	1.9861	0.932862	0.350891	0.795675
CG6184	1368.178	1.095939	1.17428	0.933286	0.350672	0.795675
Or65b	116.7637	-3.64976	3.910797	-0.93325	0.35069	0.795675
CG42523	115.6105	-2.62688	2.813069	-0.93381	0.3504	0.795675
CG43255	110.5052	-3.64897	3.910929	-0.93302	0.35081	0.795675
CG45071	764.3712	1.215213	1.302819	0.932756	0.350946	0.795675
CG2772	177.4731	-3.26777	3.50153	-0.93324	0.350696	0.795675
CG32638	830.592	1.210453	1.298227	0.932389	0.351136	0.795881
Spn88Ea	1822.515	0.830752	0.891565	0.931791	0.351445	0.796357
CG9331	2900.637	0.705318	0.757735	0.930824	0.351945	0.796769
Gr93a	240.9474	2.945261	3.164594	0.930691	0.352013	0.796769
Drep2	4726.728	0.593423	0.637629	0.930671	0.352024	0.796769
ND-SGDH	9580.064	0.46969	0.504446	0.9311	0.351802	0.796769
CG8891	1481.387	-0.90386	0.971586	-0.93029	0.352218	0.796985
Phf5a	964.3823	-1.01666	1.094206	-0.92913	0.35282	0.797359
nop5	284.5571	1.888537	2.032913	0.928981	0.352899	0.797359

Alg7	929.1504	1.095338	1.179516	0.928633	0.353079	0.797359
nolo	14314.29	-0.54593	0.587517	-0.92922	0.352773	0.797359
HPS1	1727.751	0.936656	1.008444	0.928813	0.352986	0.797359
Shawl	915.7199	-0.97345	1.047418	-0.92938	0.352693	0.797359
Bet1	812.514	-0.97084	1.044154	-0.92978	0.352484	0.797359
Atxn7	725.8517	-1.51331	1.632146	-0.92719	0.353829	0.798827
CG5104	185.8969	2.585644	2.791332	0.926312	0.354284	0.798973
lic	1002.604	1.052785	1.135959	0.926781	0.35404	0.798973
Ran	3083.083	-0.62686	0.676506	-0.92661	0.354128	0.798973
Tollo	490.592	-1.47667	1.594164	-0.9263	0.354292	0.798973
CG7781	31595.28	0.367269	0.396743	0.925709	0.354597	0.799437
sosie	230.7544	2.649551	2.86814	0.923787	0.355597	0.800012
CG5447	1711.709	-0.7996	0.865053	-0.92433	0.355312	0.800012
IntS12	314.3313	-1.94099	2.099304	-0.92459	0.355182	0.800012
Ero1L	593.244	1.526319	1.651027	0.924466	0.355244	0.800012
fwe	11123.14	0.429077	0.464219	0.9243	0.35533	0.800012
asRNA:CR4	131.5491	2.120164	2.295231	0.923726	0.355629	0.800012
rut	3778.987	0.658519	0.712903	0.923716	0.355634	0.800012
PVRAP	1379.392	-0.783	0.847694	-0.92369	0.35565	0.800012
Tsc1	1122.108	0.872812	0.945625	0.923	0.356007	0.800593
RhoGAP5A	91.8586	-3.05563	3.316958	-0.92122	0.356938	0.80084
Spx	324.7356	-1.6731	1.817115	-0.92075	0.357182	0.80084
CG7910	100.9259	-3.48229	3.783857	-0.9203	0.357415	0.80084
hng2	198.142	2.820941	3.062897	0.921004	0.357048	0.80084
JHDM2	480.5076	-2.04507	2.222104	-0.92033	0.357399	0.80084
Fkbp39	658.0338	-1.28553	1.394925	-0.92157	0.356751	0.80084
PIG-T	2016.179	0.806418	0.875849	0.920728	0.357193	0.80084
Ehbp1	5087.117	-0.61427	0.667205	-0.92065	0.357231	0.80084
CG12128	793.0859	1.484475	1.610181	0.921931	0.356565	0.80084
Muc14A	766.3437	-1.08538	1.178652	-0.92086	0.357123	0.80084
Spf45	1722.791	-0.86456	0.939309	-0.92042	0.357354	0.80084
UQCR-11	14083.7	0.436089	0.473785	0.920437	0.357344	0.80084
CG43219	293.0469	1.590424	1.725513	0.921711	0.356679	0.80084
CG32444	134.3177	-3.25011	3.532364	-0.92009	0.357523	0.80086
CG8641	980.1586	1.011314	1.099545	0.919757	0.3577	0.801032
CG5862	605.9803	-1.40407	1.529305	-0.91811	0.358561	0.802683
hep	1738.067	-0.8295	0.903635	-0.91796	0.358637	0.802683
SREBP	4938.479	0.573511	0.625283	0.917202	0.359037	0.80313
lncRNA:CR	70.94135	3.562236	3.883429	0.917291	0.35899	0.80313
sni	1447.652	-1.09323	1.192485	-0.91677	0.359265	0.803193
ND-B12	12510.54	0.441278	0.481334	0.91678	0.359258	0.803193
pie	378.5146	1.83972	2.008254	0.916079	0.359625	0.803657
PhKgamma	6641.777	0.55121	0.601765	0.915988	0.359673	0.803657
lncRNA:CR	243.9826	-1.79177	1.956854	-0.91564	0.359857	0.803843
CG12253	1489.559	-0.99831	1.090788	-0.91522	0.360075	0.804101
dod	621.4571	-1.20428	1.316371	-0.91485	0.360272	0.804101
NLaz	1781.577	-1.12814	1.232933	-0.915	0.36019	0.804101
ash2	1802.595	-0.76396	0.835353	-0.91454	0.360433	0.804235
AANAT1	8855.868	-0.52192	0.571122	-0.91385	0.360795	0.804817
CG7289	1335.63	-1.08144	1.183629	-0.91366	0.360894	0.804817

Nipsnap	6317.706	-0.51319	0.562263	-0.91272	0.361388	0.805023
l(2)k09913	12691.94	-0.45464	0.497882	-0.91315	0.361164	0.805023
pcm	692.109	1.574428	1.724976	0.912725	0.361387	0.805023
Sras	965.6881	-1.34637	1.47505	-0.91276	0.361368	0.805023
HP1c	496.0432	1.425551	1.56546	0.910627	0.362492	0.805917
MED26	10945.6	0.423569	0.465026	0.910849	0.362375	0.805917
Pgant2	1970.013	0.896665	0.983705	0.911519	0.362022	0.805917
mol	582.1971	-1.25769	1.38057	-0.91099	0.362299	0.805917
MYPT-75D	3729.366	-0.59811	0.656789	-0.91065	0.362479	0.805917
CG18265	1035.932	-1.23904	1.359879	-0.91114	0.362221	0.805917
Papst2	579.759	1.752359	1.923084	0.911224	0.362178	0.805917
CG14411	1968.767	-0.67369	0.740325	-0.90999	0.362829	0.806393
CG6512	2551.34	0.897889	0.986866	0.90984	0.362907	0.806393
jef	11124.53	0.431952	0.474897	0.90957	0.363049	0.806423
lncRNA:CR	144.0951	-2.39846	2.637317	-0.90943	0.363121	0.806423
CG2611	216.9678	-2.10001	2.311406	-0.90854	0.363593	0.807159
mTerf5	591.1345	-1.72189	1.895706	-0.90831	0.363715	0.807159
CG15890	1950.788	1.101347	1.212624	0.908234	0.363755	0.807159
CG9147	584.8346	-1.35222	1.489381	-0.90791	0.363925	0.807313
Ranbp16	4720.72	0.524715	0.578057	0.907722	0.364025	0.807313
CG8080	544.4963	-1.71028	1.885893	-0.90688	0.364469	0.808074
CG15771	1573.699	0.816151	0.900588	0.906243	0.364807	0.808274
DNaseII	2794.499	0.965209	1.065186	0.906141	0.364861	0.808274
CG15450	130.0104	-3.53017	3.895143	-0.9063	0.364777	0.808274
Hsc70-3	9062.966	-0.48126	0.531376	-0.90569	0.365102	0.808361
Galphai	2221.447	0.67455	0.744692	0.905811	0.365036	0.808361
CG31373	308.0624	-2.11949	2.340985	-0.90538	0.365262	0.808492
CR18275	80.74544	-3.52639	3.898595	-0.90453	0.365715	0.808604
TM9SF2	5331.776	0.623482	0.689087	0.904795	0.365574	0.808604
snu	82.06622	-3.52653	3.898572	-0.90457	0.365694	0.808604
igl	8192.356	-0.53092	0.5869	-0.90462	0.365666	0.808604
side-VII	3089.082	-0.63126	0.698067	-0.9043	0.365835	0.808645
stwl	133.9397	-2.6829	2.967482	-0.9041	0.365942	0.808659
CG42240	1118.946	-1.04193	1.153018	-0.90366	0.366178	0.808958
CG32452	291.0874	-2.31278	2.561449	-0.90292	0.366569	0.809473
PEK	1573.597	0.829743	0.919701	0.902188	0.366957	0.809473
rau	205.3676	-2.59458	2.879022	-0.9012	0.367481	0.809473
LManII	4104.455	0.619732	0.687644	0.90124	0.367461	0.809473
CG5890	6734.473	0.691417	0.767603	0.900748	0.367722	0.809473
Men-b	14949.31	-0.43694	0.484497	-0.90184	0.367141	0.809473
CG8360	1740.369	1.205887	1.338272	0.901077	0.367547	0.809473
Adar	10378.13	-0.41971	0.46533	-0.90196	0.367076	0.809473
Karybeta3	5528.101	0.572922	0.635313	0.901795	0.367166	0.809473
Opa1	4694.819	0.569285	0.631463	0.901533	0.367305	0.809473
Wnt5	876.7172	1.077461	1.195524	0.901246	0.367458	0.809473
Golgin104	940.0645	-1.1258	1.249717	-0.90084	0.367672	0.809473
Cbl	1478.097	0.762272	0.846193	0.900825	0.367681	0.809473
CG32104	66.25976	3.281465	3.647219	0.899717	0.368271	0.810459
DCP2	6279.164	-0.48571	0.540028	-0.89942	0.368427	0.810581
CG9743	212.2241	2.646176	2.944639	0.898642	0.368843	0.811051

Sec6	462.2585	1.618546	1.800859	0.898763	0.368779	0.811051
raw	1656.245	-1.22395	1.362415	-0.89837	0.368988	0.811147
Ctr9	1367.366	1.026062	1.142553	0.898044	0.369162	0.811307
Rga	839.8854	0.955179	1.064358	0.897423	0.369493	0.811664
Surf4	3054.056	-0.87277	0.973049	-0.89695	0.369747	0.811664
CG6231	190.1417	2.687941	2.999168	0.896229	0.370131	0.811664
Hcf	7812.39	0.442362	0.49353	0.896322	0.370081	0.811664
AcCoAS	22526.77	0.374225	0.417146	0.897109	0.369661	0.811664
CG17807	91.98826	-3.27355	3.650533	-0.89673	0.369862	0.811664
lncRNA:CR	192.8984	-2.11142	2.354596	-0.89672	0.369866	0.811664
CG3764	1513.679	1.057086	1.17949	0.896223	0.370134	0.811664
E2f2	1474.757	0.868137	0.969425	0.895518	0.37051	0.811955
grsm	508.771	-1.58746	1.773996	-0.89485	0.370868	0.811955
rdgBbeta	1815.995	0.697622	0.779606	0.894839	0.370873	0.811955
snz	1416.385	-1.15657	1.292221	-0.89502	0.370775	0.811955
CG4332	402.9756	2.11223	2.359381	0.895247	0.370655	0.811955
Chmp1	4388.251	-0.57016	0.636572	-0.89567	0.37043	0.811955
Rrp46	307.1054	2.311834	2.584193	0.894606	0.370998	0.812006
CG31869	340.8528	-1.36108	1.52412	-0.89303	0.371842	0.812303
Ugt49C1	137.5463	-2.82637	3.162386	-0.89375	0.371458	0.812303
Su(var)2-1(	1404.282	0.826599	0.92498	0.89364	0.371515	0.812303
CG5382	1210.343	0.884047	0.989583	0.893353	0.371668	0.812303
CG17691	5597.972	-0.52749	0.590506	-0.89328	0.371707	0.812303
VepD	584.5169	1.324025	1.481801	0.893524	0.371576	0.812303
CG10418	597.3618	-1.07923	1.20833	-0.89316	0.371772	0.812303
PGRP-LC	1540.554	-0.83546	0.936405	-0.8922	0.372284	0.812882
Su(z)12	678.2487	1.233604	1.382722	0.892156	0.372309	0.812882
Mkrn1	1640.732	-1.09781	1.231304	-0.89158	0.372615	0.813329
Usp16-45	582.1418	-1.49855	1.682108	-0.89087	0.372997	0.813555
wdb	13902.34	0.384237	0.431327	0.890825	0.373023	0.813555
Toll-7	1936.594	-0.88455	0.992744	-0.89101	0.372923	0.813555
CG1545	14109.92	-0.49639	0.557348	-0.89063	0.373128	0.813564
MBD-like	1165.685	0.836596	0.93979	0.890194	0.373362	0.813851
CG14591	4282.075	0.556733	0.625832	0.889589	0.373687	0.813891
sqd	16720.69	-0.38829	0.43657	-0.88941	0.373785	0.813891
rdgC	1480.483	-1.05653	1.187492	-0.88972	0.373619	0.813891
Sms	4317.796	0.646678	0.726935	0.889596	0.373683	0.813891
CG3226	1715.508	-1.133	1.274611	-0.8889	0.374057	0.81426
Mtap	948.3667	-1.0041	1.130558	-0.88815	0.374461	0.81492
CG10631	3034.963	-0.56746	0.639931	-0.88676	0.375209	0.815475
orb	717.8065	1.101728	1.242731	0.886538	0.375328	0.815475
CG17883	9983.485	-0.45855	0.517343	-0.88635	0.375427	0.815475
Mcr	2400.068	-1.03351	1.165881	-0.88647	0.375366	0.815475
CG7970	747.2818	1.043084	1.176036	0.886949	0.375106	0.815475
akirin	4341.502	-0.63709	0.718237	-0.88702	0.375066	0.815475
CG3609	2048.48	-0.7789	0.877889	-0.88724	0.374947	0.815475
CG17202	1534.02	-0.77727	0.877819	-0.88546	0.375909	0.815857
Tpi	15192.49	-0.40371	0.456033	-0.88527	0.376013	0.815857
Sbp2	236.5719	-2.08304	2.352294	-0.88554	0.375868	0.815857
enc	7431.313	-0.50237	0.567598	-0.88508	0.376112	0.815857

Gem2	1389.189	-0.80906	0.913673	-0.8855	0.375885	0.815857
EMC10	5558.589	0.511954	0.580839	0.881403	0.3781	0.817862
CG8673	93.76508	-3.1587	3.583941	-0.88135	0.378129	0.817862
obe	576.9523	-1.21512	1.378012	-0.88179	0.37789	0.817862
Sep-02	2209.551	-0.65555	0.743252	-0.882	0.377777	0.817862
X11Lbeta	6347.322	-0.59374	0.672541	-0.88283	0.377329	0.817862
mt:Cyt-b	489384.4	0.329395	0.373807	0.881189	0.378215	0.817862
CG34205	435.8655	-1.66767	1.890967	-0.88191	0.377825	0.817862
Trf2	4677.061	-0.64458	0.730068	-0.8829	0.377289	0.817862
Vps4	1958.027	0.756365	0.85727	0.882295	0.377617	0.817862
CG16758	993.4772	1.487581	1.688304	0.88111	0.378258	0.817862
CG17233	1521.2	0.788323	0.893628	0.88216	0.37769	0.817862
Gs1l	3601.983	0.544821	0.618243	0.881241	0.378188	0.817862
CG11723	1911.464	-0.75605	0.858714	-0.88044	0.378618	0.818419
Mur2B	1856.643	0.691572	0.7858	0.880086	0.378813	0.818619
scf	4254.336	-0.52692	0.599105	-0.87952	0.379122	0.819066
CG12935	481.4707	1.718179	1.954707	0.878996	0.379404	0.819234
Trs33	393.5066	1.249854	1.421706	0.879123	0.379335	0.819234
TMEM216	1233.529	-0.93621	1.065413	-0.87873	0.379549	0.819327
Cyp4d1	112.221	-2.5161	2.871154	-0.87634	0.380847	0.819908
CG2017	1428.289	0.883693	1.009324	0.87553	0.381286	0.819908
dpr16	188.5898	2.047909	2.340358	0.875041	0.381552	0.819908
IPIP	705.8317	1.033074	1.1811	0.874671	0.381753	0.819908
gro	2214.829	-0.63119	0.719696	-0.87702	0.380476	0.819908
CG31030	22035.73	0.351769	0.401972	0.875108	0.381515	0.819908
CG6805	3084.894	-0.58872	0.672083	-0.87597	0.381048	0.819908
CG30096	268.1036	2.093636	2.393741	0.874629	0.381776	0.819908
lt	4692.402	0.528862	0.602742	0.877426	0.380255	0.819908
trio	8024.333	0.454938	0.519595	0.875562	0.381268	0.819908
Nna1	1487.264	-0.88123	1.00629	-0.87572	0.38118	0.819908
CG12024	3084.338	-0.5952	0.67831	-0.87747	0.38023	0.819908
CG13003	66.87175	3.418398	3.906768	0.874994	0.381577	0.819908
gogo	1132.991	-1.11836	1.277233	-0.87561	0.381243	0.819908
Mbs	5841.539	-0.45718	0.522437	-0.87509	0.381524	0.819908
CG7945	2635.81	0.7987	0.909679	0.878002	0.379942	0.819908
CG5645	201.322	-2.31898	2.647968	-0.87576	0.381161	0.819908
rt	269.8823	-1.96687	2.249208	-0.87447	0.381861	0.819908
Rbfox1	24830.01	-0.36151	0.412691	-0.87598	0.381042	0.819908
VGlut	4118.042	-1.1249	1.281829	-0.87758	0.380173	0.819908
Sec61alpha	7604.989	-0.43892	0.502314	-0.87379	0.382231	0.820483
zf30C	1308.034	0.834224	0.955034	0.873502	0.38239	0.820604
FucT6	2614.846	0.583073	0.667673	0.873291	0.382505	0.820631
CG14762	15801.23	-0.38211	0.43792	-0.87255	0.382908	0.821277
FBXO11	2716.611	-0.62222	0.715278	-0.8699	0.384356	0.822189
CG1951	763.0615	-0.95755	1.099075	-0.87123	0.383626	0.822189
zda	3662.031	0.976216	1.121831	0.870199	0.384192	0.822189
Sptr	640.1761	-1.35193	1.552955	-0.87055	0.384	0.822189
CG12325	142.9098	-3.06272	3.520794	-0.8699	0.384357	0.822189
Pka-R1	8001.332	-0.49434	0.567766	-0.87067	0.383933	0.822189
CG12025	6932.583	0.434444	0.499254	0.870188	0.384198	0.822189

HBS1	517.5435	1.175183	1.350493	0.870188	0.384198	0.822189
CG6479	174.42	2.646501	3.036429	0.871583	0.383436	0.822189
CG16979	998.6005	-1.26378	1.451449	-0.8707	0.383916	0.822189
CG11378	886.7817	-1.38459	1.592085	-0.86967	0.38448	0.822232
CG10147	543.9991	1.69928	1.954603	0.869374	0.384643	0.822361
opa	1594.742	-0.69744	0.802763	-0.86879	0.38496	0.822819
fabp	1459.567	-0.73845	0.850534	-0.86822	0.385273	0.823109
CG42268	10894.61	0.414412	0.477339	0.868172	0.3853	0.823109
mt:ND1	28179.95	-0.43765	0.504582	-0.86735	0.38575	0.82333
koko	280.0743	-2.54503	2.933596	-0.86755	0.385643	0.82333
CG11263	268.5058	-2.53121	2.918723	-0.86723	0.385814	0.82333
Blimp-1	6833.007	-0.47244	0.54475	-0.86727	0.385795	0.82333
CG5783	153.5847	3.147245	3.635442	0.865712	0.386648	0.823731
Syx1A	21475.32	0.378579	0.437193	0.865931	0.386528	0.823731
CG5098	3035.935	-0.80895	0.933534	-0.86655	0.386191	0.823731
Vhl	1324.118	0.774195	0.893741	0.866241	0.386358	0.823731
Prosbeta5	5560.535	-0.49483	0.571138	-0.86639	0.386274	0.823731
nSyb	33665.47	0.35484	0.409945	0.86558	0.38672	0.823731
Clic	1543.121	0.854854	0.987599	0.865588	0.386716	0.823731
px	2607.364	-0.58789	0.679353	-0.86537	0.386833	0.823753
trpl	191.7333	2.31233	2.675572	0.864237	0.387457	0.824715
Tim8	3333.856	0.539604	0.624414	0.864177	0.387491	0.824715
CG4793	90.83306	-3.33984	3.87256	-0.86244	0.388447	0.826176
CG3328	2155.19	0.661949	0.767595	0.862366	0.388486	0.826176
Sxl	9946.819	0.413408	0.479335	0.862463	0.388433	0.826176
CG11030	2049.369	0.680241	0.78902	0.862134	0.388614	0.826229
CG3704	733.6707	-1.4601	1.696825	-0.86049	0.389521	0.826459
GlcAT-I	432.989	-1.42581	1.656516	-0.86073	0.389386	0.826459
Kdm2	1665.926	-0.70977	0.824041	-0.86132	0.389059	0.826459
cdi	4551.955	0.539838	0.627344	0.860513	0.389506	0.826459
CG10254	987.1084	0.863356	1.003388	0.860441	0.389546	0.826459
DMAP1	1147.569	0.790333	0.917871	0.86105	0.38921	0.826459
CG7137	256.792	-2.04134	2.369395	-0.86154	0.388939	0.826459
Dhc64C	4947.257	-0.59351	0.689763	-0.86045	0.38954	0.826459
ND-13A	7334.433	-0.44475	0.518198	-0.85825	0.390753	0.826498
Bub1	1677.8	0.82163	0.956532	0.858967	0.390358	0.826498
Su(var)205	2469.993	0.613179	0.714184	0.858574	0.390576	0.826498
Akap200	3615.878	0.620692	0.723243	0.858207	0.390778	0.826498
CG31676	6195.722	-0.60663	0.706746	-0.85834	0.390702	0.826498
CG31248	6372.192	-0.58729	0.685417	-0.85684	0.391534	0.826498
Alg12	834.0444	1.312939	1.534433	0.855651	0.392191	0.826498
CG11447	337.3668	-1.3971	1.632405	-0.85585	0.392078	0.826498
His2Av	4418.701	-0.50826	0.594462	-0.85499	0.392556	0.826498
CAH9	135.1734	3.26516	3.817247	0.85537	0.392346	0.826498
superdeatl	784.1769	1.251527	1.465036	0.854264	0.392959	0.826498
CG1607	5796.429	-0.76875	0.899295	-0.85483	0.392643	0.826498
CG15909	298.5976	-1.74081	2.03869	-0.85389	0.393169	0.826498
icln	269.6315	-2.14124	2.491954	-0.85926	0.390195	0.826498
Pms2	65.44615	2.849504	3.334259	0.854614	0.392765	0.826498
mbt	3306.17	0.635115	0.741472	0.856561	0.391688	0.826498

CG34348	788.7679	-0.91164	1.067438	-0.85404	0.393082	0.826498
meigo	1647.159	-1.14354	1.337895	-0.85473	0.392702	0.826498
CG42766	68.81759	3.036111	3.540279	0.857591	0.391118	0.826498
Unr	11723.46	0.421817	0.492241	0.856931	0.391483	0.826498
mth	128.9737	-2.82665	3.309618	-0.85407	0.393066	0.826498
CG44385	66.95445	-3.34908	3.90352	-0.85796	0.390913	0.826498
CG32267	1751.521	0.692944	0.810026	0.85546	0.392297	0.826498
Rab40	2787.557	-0.68099	0.794294	-0.85735	0.391253	0.826498
CG8915	405.7039	-1.91853	2.233723	-0.85889	0.390401	0.826498
CG6762	109.3859	2.049118	2.395353	0.855455	0.392299	0.826498
CG14082	280.7312	1.869273	2.177166	0.85858	0.390572	0.826498
Nc73EF	17865.43	-0.41724	0.486861	-0.85701	0.39144	0.826498
SsRbeta	3505.193	0.55919	0.654561	0.854297	0.392941	0.826498
D	731.4068	-1.20207	1.400641	-0.85823	0.390766	0.826498
vers	795.7527	-1.05853	1.235691	-0.85663	0.391651	0.826498
I-2	1523.8	-1.24308	1.454238	-0.8548	0.392661	0.826498
loj	1560.187	-0.69995	0.814565	-0.85929	0.390178	0.826498
tank	932.8746	-1.3072	1.519696	-0.86017	0.389695	0.826498
Rrp1	686.3456	1.393314	1.625426	0.857199	0.391335	0.826498
CG5902	1388.065	-1.02026	1.195952	-0.85309	0.393609	0.826774
Svil	5702.215	-0.45566	0.534101	-0.85313	0.393589	0.826774
Ccn	4340.777	0.51314	0.601448	0.853174	0.393563	0.826774
Gpdh1	11393.33	0.403292	0.473159	0.852338	0.394026	0.827218
CG14937	125.4011	-2.82668	3.315987	-0.85244	0.39397	0.827218
GstE11	386.834	-1.5194	1.783713	-0.85182	0.394313	0.827604
mEFTu1	2039.549	-0.6264	0.735991	-0.8511	0.394716	0.828234
GluRIIE	69.72957	2.888601	3.395189	0.850792	0.394885	0.828355
asRNA:CR4	511.8596	-1.19832	1.40876	-0.85062	0.39498	0.828355
Nmdmc	1238.491	-0.92749	1.091132	-0.85003	0.39531	0.828365
gb	310.5329	1.404479	1.653988	0.849147	0.395799	0.828365
Mccc2	151.0609	-2.75584	3.241666	-0.85013	0.395252	0.828365
RpS2	21044.33	0.35358	0.416264	0.849414	0.395651	0.828365
LSm1	434.8304	1.317961	1.552138	0.849126	0.395811	0.828365
HSPC300	1084.912	-0.81502	0.959805	-0.84915	0.395799	0.828365
Tat	2759.5	0.564487	0.663968	0.850172	0.39523	0.828365
CG13305	133.0809	3.261636	3.838855	0.849638	0.395526	0.828365
CG17928	1157.336	-1.18745	1.398749	-0.84893	0.395918	0.828372
CG3655	568.8761	-1.50614	1.776914	-0.84762	0.396652	0.829043
CG1890	1242.348	-0.89841	1.059708	-0.84779	0.396556	0.829043
CG3358	282.4558	2.029416	2.394024	0.847701	0.396605	0.829043
Asator	15461.91	0.369981	0.436313	0.847973	0.396453	0.829043
Syn1	1753.906	-0.82045	0.969553	-0.84621	0.397434	0.829543
TyrRII	87.32376	2.927472	3.463854	0.845149	0.398028	0.829543
tmod	14166.7	-0.37131	0.438833	-0.84614	0.397477	0.829543
pen-2	1237.931	-0.76482	0.904835	-0.84526	0.397966	0.829543
CG42750	5380.548	0.45007	0.532223	0.845642	0.397752	0.829543
hppy	4430.695	0.562401	0.665266	0.845377	0.3979	0.829543
CG4554	249.1467	-1.95841	2.312674	-0.84682	0.397097	0.829543
CG13579	2811.589	0.65813	0.778509	0.845373	0.397903	0.829543
tipE	1420.113	-0.72468	0.856979	-0.84562	0.397763	0.829543

CG7341	381.8788	1.663134	1.965742	0.846059	0.39752	0.829543
Ldh	113.3713	2.44466	2.888472	0.846351	0.397357	0.829543
bib	950.676	1.532457	1.814261	0.844673	0.398294	0.829666
CG5727	735.6762	1.214598	1.437755	0.844788	0.398229	0.829666
wb	110.2687	-3.22883	3.827959	-0.84349	0.398956	0.830831
sfl	1792.123	0.754654	0.894901	0.843282	0.399071	0.830853
CG13766	675.0993	1.302434	1.54768	0.84154	0.400046	0.831769
Gr32a	141.3699	-3.18427	3.78154	-0.84206	0.399756	0.831769
CG6689	794.4127	1.197844	1.424205	0.841061	0.400314	0.831769
eIF3c	2036.511	-0.64635	0.767643	-0.84199	0.399793	0.831769
Rcd1	3876.827	0.602241	0.715545	0.841654	0.399982	0.831769
Atpalpha	525640.9	-0.32186	0.382378	-0.84174	0.399936	0.831769
Ubi-p63E	1603.035	0.674527	0.801759	0.84131	0.400175	0.831769
rad50	734.6652	1.37192	1.631289	0.841003	0.400346	0.831769
Ae2	13970.86	0.436245	0.518827	0.84083	0.400443	0.831769
GAA1	946.7898	-1.33811	1.592336	-0.84034	0.400716	0.832121
bsf	1877.443	0.672571	0.800826	0.839847	0.400994	0.832483
Surf1	187.4742	-2.50865	2.990539	-0.83886	0.401546	0.833197
D19B	152.7091	1.939138	2.311442	0.83893	0.401509	0.833197
M1BP	1098.152	-0.85491	1.019615	-0.83846	0.401772	0.833451
FMRFa	226.5702	1.934468	2.309109	0.837755	0.402168	0.833534
Gs2	72529.54	-0.35482	0.423587	-0.83765	0.402227	0.833534
CG6769	338.5005	1.624415	1.938663	0.837905	0.402084	0.833534
CG32039	1292.358	0.7693	0.917861	0.838144	0.40195	0.833534
Mal-A5	5598.572	-0.55197	0.659311	-0.83719	0.402485	0.833637
lncRNA:CR	1733.519	-0.85921	1.02622	-0.83726	0.402448	0.833637
Fur2	3894.729	0.588063	0.702674	0.836892	0.402653	0.833771
CG13287	364.0618	-2.52693	3.020232	-0.83667	0.402779	0.833816
mRpS34	540.309	1.570827	1.878144	0.836372	0.402946	0.833946
CG10804	5313.895	0.58157	0.695952	0.835646	0.403354	0.834576
CG32091	251.0335	1.416866	1.695947	0.835443	0.403468	0.834597
CG12171	1400.911	-1.19309	1.42909	-0.83486	0.403795	0.835024
CG17904	1671.759	-0.70345	0.84313	-0.83434	0.404091	0.835024
Psa	3163.113	-0.52771	0.632328	-0.83455	0.403969	0.835024
VhaAC39-1	14372.76	0.364415	0.436764	0.834352	0.404083	0.835024
D1	2568.051	-0.56176	0.674142	-0.8333	0.404677	0.836021
CG10194	870.8975	1.532249	1.839327	0.833049	0.404817	0.836094
fng	2682.573	0.581344	0.698304	0.832508	0.405122	0.836482
CG43373	2429.906	0.715016	0.859037	0.832347	0.405213	0.836482
CG17048	64.10158	-3.24684	3.905531	-0.83134	0.405779	0.836804
Pdrg1	146.1606	2.150673	2.585832	0.831714	0.40557	0.836804
lncRNA:CR	3165.193	0.774728	0.931719	0.831504	0.405689	0.836804
CG6674	618.7654	-1.41292	1.699582	-0.83133	0.405786	0.836804
CG13843	79.30458	3.248621	3.909106	0.831039	0.405951	0.836812
CG32512	2348.45	-0.57699	0.69437	-0.83096	0.405999	0.836812
CG12499	287.6983	-1.82194	2.194098	-0.83038	0.406322	0.836835
Cfp1	813.3487	-1.23246	1.483825	-0.8306	0.406202	0.836835
lncRNA:CR	754.1037	1.190994	1.43423	0.830406	0.406309	0.836835
CG32333	1210.624	0.955451	1.15208	0.829326	0.40692	0.837205
Lztr1	1021.382	0.976597	1.177514	0.829372	0.406894	0.837205

unc-104	59338.19	0.367902	0.443431	0.829672	0.406724	0.837205
Tnpo	4307.22	-0.51406	0.619648	-0.8296	0.406764	0.837205
Dyrk3	12934.05	-0.40065	0.483299	-0.82898	0.407116	0.837395
CG10778	354.1329	-1.80716	2.183337	-0.8277	0.407838	0.837846
ND-75	4734.258	-0.54126	0.653538	-0.8282	0.407555	0.837846
ND-MWFE	915.7186	0.852612	1.03013	0.827674	0.407855	0.837846
Pss	258.0524	1.699074	2.052608	0.827763	0.407804	0.837846
Pi3K68D	1830.327	-0.62967	0.760776	-0.82767	0.407858	0.837846
CG14853	7140.381	0.689877	0.83405	0.827142	0.408157	0.838246
CkIalpha	9054.514	-0.4035	0.487991	-0.82687	0.408311	0.838349
Cks85A	554.2912	-1.36108	1.64737	-0.82622	0.408682	0.838466
Nup358	1538.342	0.773198	0.935648	0.826378	0.40859	0.838466
Sgs1	162.0292	-2.98944	3.61712	-0.82647	0.408538	0.838466
eRF1	4991.415	-0.53353	0.646248	-0.82558	0.40904	0.838791
beat-IIa	6378.725	0.441641	0.535724	0.824382	0.409722	0.838791
Klp98A	2855.447	0.72019	0.873582	0.82441	0.409707	0.838791
mys	3883.083	0.576596	0.698737	0.825196	0.40926	0.838791
CG3792	1706.445	-0.71212	0.8637	-0.8245	0.409657	0.838791
Ptpmeg2	7848.49	0.504764	0.612447	0.824177	0.409839	0.838791
CG11000	11833.26	-0.42057	0.509629	-0.82524	0.409235	0.838791
CG43736	2897.51	0.552752	0.670738	0.824096	0.409885	0.838791
CG11679	227.6528	2.123927	2.576246	0.824427	0.409697	0.838791
mRpL12	2147.013	-0.62892	0.762004	-0.82535	0.40917	0.838791
CG5245	100.8497	3.21624	3.905071	0.823606	0.410163	0.838933
sigmar	1817.409	0.68369	0.830087	0.823636	0.410146	0.838933
Ostgamma	2153.234	-0.75033	0.912263	-0.82249	0.410799	0.839402
CG4901	557.0526	1.417833	1.72378	0.822514	0.410784	0.839402
Tom70	1099.94	-0.76642	0.931932	-0.8224	0.410847	0.839402
Rlb1	67.76455	3.211683	3.905811	0.822283	0.410916	0.839402
FipoQ	196.7412	-1.99624	2.42708	-0.82249	0.4108	0.839402
Swip-1	1423.625	0.726846	0.884583	0.821681	0.411258	0.839888
Vps39	1082.539	1.499262	1.827164	0.820541	0.411908	0.840003
tnc	346.3908	-1.7962	2.189337	-0.82043	0.41197	0.840003
CG34229	168.6121	2.043929	2.488599	0.821317	0.411466	0.840003
shps	427.8704	2.10232	2.562879	0.820296	0.412047	0.840003
asRNA:CR4	198.0807	1.539201	1.875695	0.820603	0.411872	0.840003
ago	2153.042	-0.62113	0.757156	-0.82035	0.412017	0.840003
UBL3	3278.867	-0.53305	0.649592	-0.8206	0.411877	0.840003
CG30008	219.5224	2.292363	2.797159	0.819533	0.412483	0.840677
vig	5086.148	-0.55821	0.681915	-0.81859	0.41302	0.841346
Cdc42	1171.62	-0.93142	1.137604	-0.81875	0.412928	0.841346
Ugt49B1	695.6854	-1.30565	1.595561	-0.8183	0.413185	0.841468
CG42613	78696.12	-0.37105	0.453588	-0.81803	0.413342	0.841513
Fife	11430.25	0.37145	0.454154	0.817895	0.413417	0.841513
bab2	741.57	0.937419	1.1464	0.817707	0.413525	0.841518
tok	3763.006	0.58054	0.710871	0.816659	0.414123	0.842253
CG5742	513.947	1.307374	1.601144	0.816524	0.4142	0.842253
CG2818	794.132	-1.18924	1.456377	-0.81657	0.414172	0.842253
PGAP2	706.1011	1.29524	1.588417	0.815428	0.414827	0.842267
VhaM9.7-b	4891.771	0.487989	0.599126	0.814502	0.415357	0.842267

CCT4	1324.896	-0.78792	0.965839	-0.81579	0.414619	0.842267
Vha100-2	1056.691	0.94209	1.15639	0.814682	0.415254	0.842267
Idi	698.4507	-1.45709	1.787144	-0.81532	0.414889	0.842267
HmgZ	9492.146	-0.38217	0.469392	-0.81419	0.415538	0.842267
Pgant1	650.8556	1.08936	1.336879	0.814853	0.415156	0.842267
Iswi	1461.177	-0.68035	0.833903	-0.81586	0.414581	0.842267
CG8712	299.3107	2.086631	2.562944	0.814154	0.415557	0.842267
Pde1c	9760.026	0.3841	0.470612	0.816172	0.414402	0.842267
ksh	1396.9	-0.84402	1.035917	-0.81475	0.415214	0.842267
CG32137	147.2568	-2.22883	2.737689	-0.81413	0.415571	0.842267
CG15439	652.543	0.950117	1.166971	0.814173	0.415546	0.842267
eIF2alpha	1423.381	-0.7998	0.982785	-0.81381	0.415751	0.842418
Pde8	4121.999	-0.53424	0.657162	-0.81296	0.416244	0.843203
CG10874	355.5127	1.231821	1.515728	0.812692	0.416394	0.843296
epsilonCOI	2096.143	0.699945	0.862679	0.811362	0.417158	0.843564
Ask1	995.5747	-0.94715	1.167904	-0.81099	0.417374	0.843564
Cchl	3162.699	-0.55453	0.684067	-0.81064	0.417573	0.843564
CG42322	11156.42	0.481942	0.594486	0.810687	0.417546	0.843564
CG42827	153.9038	-2.11072	2.599548	-0.81195	0.416818	0.843564
St3	4414.085	0.532597	0.656573	0.811177	0.417264	0.843564
Doa	7513.797	0.571525	0.704582	0.811155	0.417277	0.843564
PMP34	1611.538	-1.12543	1.386069	-0.81196	0.416817	0.843564
CG4661	189.3311	2.317704	2.859136	0.810631	0.417578	0.843564
CG3408	309.6275	1.673956	2.06391	0.811061	0.417331	0.843564
CG12541	410.1531	-1.20517	1.487331	-0.81029	0.417773	0.843588
angel	463.7944	1.645905	2.03137	0.810244	0.4178	0.843588
CG18273	389.3723	-2.07081	2.56865	-0.80619	0.420135	0.843938
Hem	1281.177	0.807871	1.001225	0.806883	0.419734	0.843938
PrBP	3485.294	-0.48978	0.606851	-0.80708	0.419619	0.843938
Samuel	365.5859	1.461647	1.805538	0.809536	0.418207	0.843938
sky	4141.765	-0.48363	0.599848	-0.80625	0.4201	0.843938
MAGE	90.91618	-3.16127	3.916117	-0.80725	0.419524	0.843938
CG5500	1232.796	0.738113	0.913112	0.808348	0.41889	0.843938
RpL32	6937.9	0.472763	0.586081	0.806651	0.419868	0.843938
CalpA	2362.154	0.567731	0.703578	0.80692	0.419713	0.843938
CG11368	160.9572	2.258567	2.794397	0.808248	0.418948	0.843938
CG2129	229.1107	2.11789	2.616735	0.809363	0.418306	0.843938
LBR	506.0488	-1.68539	2.08556	-0.80812	0.41902	0.843938
CG30495	563.3092	-1.51104	1.87355	-0.80651	0.419948	0.843938
mtd	39722.6	0.379144	0.469032	0.808356	0.418886	0.843938
p	958.5107	-1.39753	1.733292	-0.80628	0.420079	0.843938
inaE	3742.104	0.52002	0.643738	0.807813	0.419199	0.843938
CG9861	77.54968	3.142926	3.898573	0.806173	0.420143	0.843938
cpo	12192.17	0.363885	0.449802	0.808988	0.418522	0.843938
tn	75.56184	3.142696	3.89861	0.806107	0.420181	0.843938
PyK	22643.69	-0.33348	0.411875	-0.80965	0.418139	0.843938
Pcyt1	4056.443	0.540724	0.669267	0.807934	0.419129	0.843938
CG5664	647.53	1.041635	1.293096	0.805536	0.420511	0.844177
CG5522	2199.229	0.630051	0.782109	0.80558	0.420485	0.844177
bru3	14445.3	-0.42058	0.522248	-0.80533	0.42063	0.844206

CG7766	6101.105	-0.42228	0.52479	-0.80467	0.421009	0.844542
CG31687	63.05612	-2.83256	3.519758	-0.80476	0.420958	0.844542
CG30157	130.7416	3.098849	3.853303	0.804206	0.421278	0.844872
RpLP2	20554.14	0.373166	0.464287	0.80374	0.421547	0.8452
tsh	235.9984	1.857374	2.313128	0.802971	0.421992	0.845881
Mob2	7699.863	0.453128	0.564639	0.80251	0.422258	0.846204
csw	9660.655	0.38987	0.488401	0.798258	0.424721	0.847107
CG32797	70.00773	-3.08072	3.86789	-0.79649	0.425749	0.847107
CG11307	853.3517	-1.0027	1.257988	-0.79707	0.425413	0.847107
Chro	1428.404	0.763891	0.956739	0.798431	0.42462	0.847107
corto	12419.11	-0.3884	0.48758	-0.79658	0.425697	0.847107
snRNP-U1-	1115.636	0.810782	1.016314	0.797767	0.425006	0.847107
CG5149	2408.502	1.113234	1.397559	0.796556	0.425709	0.847107
Yip1d1	1342.426	0.762643	0.958203	0.795909	0.426085	0.847107
p23	3500.221	-0.49916	0.627205	-0.79584	0.426124	0.847107
Pnn	1446.091	-0.77118	0.967492	-0.79709	0.425398	0.847107
MRG15	626.1295	1.147573	1.43978	0.797048	0.425423	0.847107
EloB	1973.273	0.89132	1.114163	0.799991	0.423716	0.847107
CG13833	323.9317	-2.24911	2.825913	-0.79589	0.426098	0.847107
CG1571	150.1423	-2.09013	2.622705	-0.79694	0.425487	0.847107
grau	998.4872	-1.01636	1.277409	-0.79564	0.426241	0.847107
Alk	12517.09	0.36278	0.455554	0.796349	0.425829	0.847107
ari-1	4378.009	-0.50322	0.632092	-0.79612	0.425961	0.847107
OstDelta	638.194	0.988634	1.239703	0.797477	0.425174	0.847107
CG17065	3572.98	0.739353	0.923729	0.8004	0.423479	0.847107
Med	619.2923	-1.12714	1.407217	-0.80097	0.423147	0.847107
unc-13	50041.25	0.369497	0.461229	0.801112	0.423067	0.847107
CG33267	669.8511	0.999314	1.255787	0.795767	0.426167	0.847107
sotv	3618.508	0.603631	0.753504	0.801099	0.423075	0.847107
CG17568	1010.181	0.768893	0.963792	0.797779	0.424999	0.847107
Frq1	11791.2	0.37128	0.463574	0.800908	0.423185	0.847107
Dbx	177.0676	-2.08665	2.620109	-0.7964	0.4258	0.847107
RpII140	1566.42	-0.8265	1.037929	-0.7963	0.425859	0.847107
l(3)72Dn	396.7408	-1.46711	1.844223	-0.79552	0.426313	0.847107
asRNA:CR4	386.715	-1.27506	1.593098	-0.80036	0.4235	0.847107
wg	84.60345	2.921474	3.669116	0.796234	0.425896	0.847107
lncRNA:CR	606.7608	1.294944	1.626666	0.796073	0.42599	0.847107
CG6179	599.7101	1.510428	1.886194	0.800781	0.423259	0.847107
Mo25	5117.076	0.561761	0.70619	0.795482	0.426333	0.847107
peng	973.1433	-0.9622	1.205824	-0.79796	0.424894	0.847107
CG11927	423.3693	1.618831	2.035341	0.795361	0.426403	0.847107
Rilpl	3788.693	-0.47334	0.595388	-0.79502	0.426604	0.847295
snRNA:7SK	5509.63	-0.49865	0.627647	-0.79448	0.426916	0.847497
Rab5	5198.056	0.445922	0.561175	0.794623	0.426833	0.847497
Kr-h1	6940.633	0.50268	0.63293	0.794212	0.427072	0.847597
lncRNA:CR	88.16136	2.456208	3.093823	0.793907	0.42725	0.847739
Prosbeta4	1700.985	0.648192	0.817128	0.793257	0.427628	0.848281
mus201	752.5815	-1.0517	1.327084	-0.79249	0.428076	0.84854
CG34401	6475.433	-0.40744	0.513993	-0.79269	0.427958	0.84854
lncRNA:ac	120.4714	2.712687	3.422832	0.792527	0.428054	0.84854

CG43795	232.662	1.417579	1.790202	0.791854	0.428446	0.848854
tth	417.2546	1.150418	1.452624	0.791958	0.428385	0.848854
CG7582	9765.512	-0.40996	0.518137	-0.79121	0.428819	0.849383
mthl5	119.6342	2.454427	3.103968	0.790738	0.429097	0.849724
SoYb	829.8415	-1.31476	1.66364	-0.79029	0.429359	0.850034
CG8679	130.0054	2.131587	2.697974	0.790069	0.429487	0.850078
RpL11	8093.472	0.447129	0.566172	0.789742	0.429679	0.850247
TBCD	200.449	-1.73687	2.200785	-0.7892	0.429994	0.85066
Paics	110.7493	-2.3502	2.97916	-0.78888	0.430182	0.850823
asRNA:CR4	485.3721	-1.02205	1.295918	-0.78867	0.430304	0.850856
AstC	62.03944	-3.09369	3.923819	-0.78844	0.43044	0.850915
CG12728	153.2474	1.689592	2.145486	0.78751	0.430983	0.851149
eIF4EHP	7955.232	0.436675	0.554492	0.787522	0.430976	0.851149
dysc	14272.51	0.37963	0.481972	0.787659	0.430896	0.851149
Fum1	3912.761	-0.5231	0.664073	-0.78771	0.430864	0.851149
tyf	4821.624	0.431669	0.549954	0.784917	0.432502	0.851561
Hpr1	557.6595	-1.04505	1.332732	-0.78414	0.432957	0.851561
Fgop2	2035.653	0.648104	0.826548	0.78411	0.432976	0.851561
CG33296	9680.704	-0.41188	0.524374	-0.78547	0.43218	0.851561
CG14401	326.5875	1.806145	2.304062	0.783896	0.433101	0.851561
CG1227	183.722	-2.06656	2.633369	-0.78476	0.432596	0.851561
dpr15	387.417	1.279686	1.63232	0.783968	0.433059	0.851561
Not10	682.911	-1.03372	1.316686	-0.78509	0.432401	0.851561
HEATR2	488.2222	1.131945	1.443551	0.78414	0.432958	0.851561
XNP	2384.784	-0.68029	0.865052	-0.78641	0.431627	0.851561
faf	4366.521	0.455024	0.579007	0.785869	0.431944	0.851561
AnxB9	7788.089	-0.40322	0.513174	-0.78573	0.432025	0.851561
NAT1	6456.725	-0.48297	0.615207	-0.78505	0.432424	0.851561
Arpc4	1743.744	0.606928	0.774148	0.783995	0.433043	0.851561
CG7974	508.5267	1.188538	1.514231	0.784912	0.432505	0.851561
CG32052	1943.039	0.69776	0.88918	0.784724	0.432616	0.851561
CG8005	125.8116	2.33112	2.97137	0.784527	0.432731	0.851561
CG15382	247.2584	-2.18546	2.77981	-0.78619	0.431757	0.851561
CG7632	1836.505	-0.99313	1.268183	-0.78311	0.433562	0.852049
smash	3748.644	0.525066	0.670485	0.783114	0.43356	0.852049
klar	2649.794	0.534745	0.683326	0.782563	0.433884	0.852473
asRNA:CR4	97.06374	-2.09221	2.675881	-0.78188	0.434288	0.853057
EMC5	847.9146	0.847375	1.0844	0.781423	0.434554	0.853371
Debcl	962.6124	-1.06999	1.370143	-0.78094	0.43484	0.853725
CG14778	172.7892	-2.38544	3.055679	-0.78066	0.435003	0.853805
RpL35A	11763.55	0.404354	0.51931	0.778637	0.436194	0.853805
Vha55	19833.55	0.382127	0.48969	0.780345	0.435188	0.853805
CG31345	6255.816	0.414285	0.531187	0.779923	0.435436	0.853805
CG14321	1618.195	-0.67399	0.865599	-0.77865	0.436189	0.853805
Rift	362.0897	1.303369	1.673629	0.778768	0.436116	0.853805
CG6701	1831.218	-0.71179	0.914148	-0.77864	0.436191	0.853805
sax	4589.502	-0.50587	0.648485	-0.78008	0.435342	0.853805
CG9902	1224.269	1.143697	1.467272	0.779472	0.435702	0.853805
dac	574.8334	-1.28614	1.650695	-0.77915	0.435889	0.853805
CG34408	1607.875	0.967543	1.242596	0.778646	0.436188	0.853805

SerT	252.1073	-1.97632	2.533673	-0.78002	0.435378	0.853805
Sec15	441.5195	-1.29519	1.66366	-0.77852	0.436264	0.853805
CG32795	1435.509	-0.82089	1.056445	-0.77703	0.437142	0.854243
CG1218	123.7079	-2.21403	2.84979	-0.77691	0.437213	0.854243
CG9766	734.5307	0.911088	1.172625	0.776964	0.43718	0.854243
CG14505	164.3581	2.151002	2.765237	0.777873	0.436644	0.854243
lncRNA:CR	158.2017	1.83318	2.357798	0.777497	0.436866	0.854243
ap	3494.692	0.499922	0.642939	0.777557	0.43683	0.854243
CG31690	2136.514	-0.5919	0.7619	-0.77687	0.437233	0.854243
CG42741	332.0562	1.486126	1.913446	0.776675	0.437351	0.854265
CG17698	16127.74	0.33071	0.425987	0.776338	0.437549	0.854445
CG17272	2413.599	-0.57664	0.74299	-0.77611	0.437687	0.854506
lncRNA:CR	80.40356	2.92491	3.770395	0.775757	0.437893	0.854564
CG13707	2475.443	-0.60306	0.777441	-0.77569	0.43793	0.854564
hng1	378.1758	-1.39389	1.798808	-0.7749	0.4384	0.855273
CG11319	3892.33	-0.45787	0.591148	-0.77454	0.438613	0.855273
PolD1	392.5349	-1.66878	2.154259	-0.77464	0.438551	0.855273
RpS27A	16809.3	0.344759	0.446425	0.772267	0.439957	0.856022
CG10383	956.7718	0.96116	1.244151	0.772543	0.439793	0.856022
Aasdh	890.6096	-1.38765	1.796193	-0.77255	0.439789	0.856022
Epg5	387.3322	1.653631	2.139243	0.772998	0.439523	0.856022
CycE	149.8854	1.74239	2.253679	0.773132	0.439445	0.856022
CycT	3782.41	0.46627	0.603394	0.772745	0.439673	0.856022
RpS13	15575.58	-0.33692	0.435496	-0.77366	0.439134	0.856022
CG34150	660.2999	-0.94744	1.226415	-0.77253	0.439802	0.856022
l(2)gd1	353.5257	-1.39226	1.802578	-0.77237	0.439894	0.856022
Irc	1697.069	0.740651	0.960322	0.771254	0.440557	0.856152
CG13917	8957.45	-0.38983	0.505219	-0.77161	0.440346	0.856152
CG7879	1642.233	0.644026	0.834919	0.771363	0.440492	0.856152
CG1265	4979.58	0.477792	0.619031	0.771838	0.440211	0.856152
CG32641	228.399	1.783222	2.310496	0.771792	0.440238	0.856152
CG2924	1441.688	-0.73918	0.965838	-0.76533	0.444075	0.856326
IntS6	2103.769	0.755103	0.986523	0.765419	0.444022	0.856326
DIP-theta	22119.29	0.319922	0.41701	0.767181	0.442974	0.856326
CG14005	778.4722	1.349979	1.76456	0.765051	0.444241	0.856326
ND-B22	14563.44	-0.33145	0.431053	-0.76893	0.441936	0.856326
CG10165	851.7026	-1.23498	1.606306	-0.76883	0.441992	0.856326
CD98hc	12783.84	-0.50429	0.657912	-0.7665	0.44338	0.856326
CG11986	435.8687	1.78705	2.325882	0.768332	0.44229	0.856326
pont	145.5152	-2.23338	2.910801	-0.76727	0.44292	0.856326
BRWD3	724.0637	-1.20064	1.557834	-0.77071	0.440876	0.856326
Mccc1	284.0727	-1.40294	1.824257	-0.76905	0.441865	0.856326
DCTN2-p5(	1404.762	-0.72225	0.939571	-0.7687	0.442073	0.856326
Pgant3	226.4621	-2.00637	2.611704	-0.76822	0.442355	0.856326
CG11360	12789.73	0.380783	0.496575	0.766817	0.44319	0.856326
Sox102F	5030.869	0.433602	0.565194	0.767174	0.442978	0.856326
AnxB10	4726.788	-0.44471	0.579148	-0.76787	0.442564	0.856326
CG2082	10699.26	0.383687	0.498724	0.769338	0.441693	0.856326
shep	46925.77	-0.30457	0.397318	-0.76656	0.443346	0.856326
mt:srRNA	7568.816	-0.39346	0.511228	-0.76964	0.441512	0.856326

snoRNA:M	109.72	-2.77077	3.615794	-0.7663	0.4435	0.856326
kra	13895.17	0.3303	0.431767	0.764997	0.444274	0.856326
Bet3	108.9106	1.930166	2.509131	0.769257	0.441741	0.856326
tinc	1966.717	0.583824	0.762829	0.765342	0.444068	0.856326
CG42788	3086.033	-0.50003	0.651886	-0.76705	0.44305	0.856326
Myc	2396.032	-0.70946	0.923075	-0.76858	0.442144	0.856326
lncRNA:CR	86.12334	2.694643	3.518375	0.765877	0.443749	0.856326
Glo1	375.1299	-1.10228	1.436852	-0.76715	0.442991	0.856326
mge	7362.374	-0.42609	0.554158	-0.7689	0.441955	0.856326
CG9372	73.14625	3.003626	3.911952	0.767808	0.442602	0.856326
not	745.4318	-1.08283	1.413047	-0.76631	0.443494	0.856326
tant	1313.444	0.948507	1.239595	0.765174	0.444168	0.856326
l(3)psg2	1387.817	0.70423	0.919559	0.765835	0.443775	0.856326
Mgstl	328.6454	1.217464	1.582276	0.769439	0.441633	0.856326
S	11480.36	-0.39179	0.509325	-0.76923	0.441757	0.856326
IntS3	2389.703	0.567322	0.741912	0.764676	0.444465	0.856488
List	33205.6	-0.42266	0.553188	-0.76404	0.444846	0.856811
CG14100	277.1925	1.930111	2.526159	0.76405	0.444838	0.856811
Wwox	1058.06	0.804654	1.055369	0.762438	0.445798	0.856819
GstD9	13412.42	0.418291	0.547616	0.763839	0.444963	0.856819
CG18549	2528.518	-1.04503	1.37049	-0.76252	0.445749	0.856819
Ctl2	3383.558	-0.47275	0.620206	-0.76224	0.445917	0.856819
CG1774	202.9842	-1.68415	2.207889	-0.76279	0.445589	0.856819
ttk	1935.689	-0.67934	0.891182	-0.76229	0.445887	0.856819
CG30344	1792.498	-0.67173	0.880699	-0.76273	0.445626	0.856819
CG11892	1014.426	-1.28389	1.68142	-0.76358	0.44512	0.856819
CG11555	311.8862	1.703038	2.233513	0.762493	0.445766	0.856819
Acbp2	6262.026	-0.43386	0.56838	-0.76333	0.445265	0.856819
His3.3B	5039.948	0.459981	0.604016	0.761538	0.446336	0.857021
Utx	3277.769	-0.56425	0.741084	-0.76139	0.446424	0.857021
CG44088	321.9156	1.608226	2.111732	0.761567	0.446318	0.857021
nudC	198.2298	-1.83762	2.413647	-0.76135	0.44645	0.857021
CG32812	66.17028	2.977038	3.918647	0.759711	0.447428	0.857066
nAChRalph	14940.72	0.342667	0.450596	0.760475	0.446971	0.857066
CG5674	2215.731	0.601302	0.791812	0.7594	0.447613	0.857066
euc	5615.372	0.404113	0.532177	0.759358	0.447638	0.857066
FLASH	72.55697	2.97771	3.918498	0.759911	0.447308	0.857066
CG13344	1279.868	-0.80706	1.060966	-0.76069	0.446843	0.857066
lncRNA:CR	65.54208	2.976964	3.918663	0.759689	0.447441	0.857066
CG14971	1753.355	-0.61188	0.805797	-0.75934	0.447648	0.857066
VhaM9.7-c	847.5327	-1.28616	1.691139	-0.76053	0.446937	0.857066
CG2202	602.7867	-1.03323	1.358351	-0.76065	0.446867	0.857066
Stt3A	2900.641	-0.6529	0.858925	-0.76013	0.447174	0.857066
Got2	6655.511	-0.38053	0.501802	-0.75834	0.448249	0.857722
Hsp60A	8621.746	-0.3894	0.513559	-0.75823	0.448311	0.857722
CG15916	2481.612	0.604742	0.797205	0.758579	0.448105	0.857722
SLIRP2	184.4511	1.807131	2.384424	0.75789	0.448517	0.857796
CG9813	7422.328	0.515453	0.680185	0.757812	0.448563	0.857796
Mnn1	7329.654	0.400904	0.529405	0.757273	0.448886	0.858209
NUCB1	6686.304	-0.43784	0.578363	-0.75703	0.44903	0.858279

Wnt6	283.9176	1.235077	1.633238	0.756213	0.449521	0.858646
Sgt	2185.705	0.583534	0.772118	0.755758	0.449794	0.858646
wge	4675.344	-0.48915	0.64707	-0.75594	0.449683	0.858646
CG11155	21241.59	0.360421	0.476973	0.755642	0.449864	0.858646
PIG-Q	1475.541	1.077593	1.425913	0.755722	0.449816	0.858646
CG9231	2005.933	-0.5636	0.745028	-0.75648	0.449361	0.858646
ApepP	1383.067	-0.94122	1.24619	-0.75528	0.450081	0.858856
RpL24	13830.37	0.344043	0.456104	0.754308	0.450665	0.859595
lili	1748.725	0.658499	0.873189	0.754131	0.450771	0.859595
CG10660	550.7946	-1.59767	2.118643	-0.7541	0.45079	0.859595
CG17724	1597.734	0.68481	0.90846	0.753814	0.450961	0.859688
CG1463	693.0889	0.85841	1.138985	0.753662	0.451052	0.859688
MCTS1	2062.717	-0.63313	0.840449	-0.75332	0.451257	0.859874
Gel	5409.385	-0.53871	0.71537	-0.75305	0.451418	0.859932
CG8204	285.2391	1.458799	1.937996	0.752736	0.451609	0.859932
kug	4217.394	0.480077	0.637666	0.752867	0.45153	0.859932
kn	471.8443	1.271032	1.689842	0.752161	0.451954	0.860386
Lrpprc2	1018.37	-0.87788	1.167691	-0.75181	0.452167	0.860587
MESR6	173.7111	-1.70275	2.265451	-0.75161	0.452283	0.860603
CCT8	1211.117	0.803707	1.070266	0.750942	0.452688	0.860761
CG11655	576.7238	1.760331	2.344144	0.750948	0.452684	0.860761
aop	4777.415	-0.41557	0.553283	-0.7511	0.452592	0.860761
mt:CoII	338473.1	0.266561	0.355175	0.750507	0.452949	0.860851
lncRNA:CR	66.23663	2.575387	3.430823	0.750661	0.452856	0.860851
wrapper	2443.803	0.703319	0.937901	0.749886	0.453324	0.861358
w	3525.339	0.586216	0.785129	0.74665	0.455275	0.861636
Stip1	1629.666	-0.60236	0.806396	-0.74698	0.455074	0.861636
BNIP3	3128.922	-0.48784	0.65366	-0.74632	0.455473	0.861636
Lam	1287.634	0.863475	1.154233	0.748094	0.454403	0.861636
CG31635	6571.184	-0.44805	0.601334	-0.74509	0.456219	0.861636
CG7154	640.8903	0.905384	1.213976	0.7458	0.455788	0.861636
Arpc1	1064.809	0.899395	1.207156	0.745052	0.45624	0.861636
Sur-8	3551.972	0.489065	0.654417	0.747329	0.454865	0.861636
CG31475	1269.029	-0.85548	1.14512	-0.74706	0.455024	0.861636
CR13656	1221.684	1.115469	1.496921	0.745176	0.456165	0.861636
Picot	10432.54	-0.37033	0.495583	-0.74726	0.454909	0.861636
CG7737	853.5475	-0.92856	1.246073	-0.74519	0.45616	0.861636
CG2269	17958.95	0.312639	0.419588	0.745109	0.456206	0.861636
Tsp42Ef	1440.7	0.771456	1.030261	0.748797	0.45398	0.861636
CG32017	11486.02	0.342428	0.459188	0.745724	0.455834	0.861636
CG11594	120.9379	2.540866	3.399084	0.747515	0.454753	0.861636
CG11590	577.0832	-1.02813	1.372101	-0.74931	0.453669	0.861636
Nelf-A	2781.051	0.522072	0.697149	0.748867	0.453938	0.861636
Chpf	1359.223	-0.80584	1.076761	-0.74839	0.454226	0.861636
Ote	192.9755	-1.97619	2.65255	-0.74502	0.456261	0.861636
CG32281	1150.381	1.081512	1.448709	0.746535	0.455345	0.861636
Ulp1	2394.4	-0.58953	0.788407	-0.74775	0.45461	0.861636
dpr10	2449.703	0.540259	0.722187	0.748087	0.454408	0.861636
Rdl	40565.36	0.288038	0.385092	0.747972	0.454477	0.861636
ImpE1	1759.076	0.673971	0.902725	0.746597	0.455307	0.861636

RpL18	11633.61	-0.39083	0.521836	-0.74896	0.453884	0.861636
CG13375	2018.935	-0.61575	0.828122	-0.74355	0.457151	0.861975
Saf6	905.7584	1.415608	1.90455	0.743277	0.457314	0.861975
Ubc6	9179.939	-0.38087	0.51396	-0.74106	0.45866	0.861975
Mal-B2	428.7229	-1.24004	1.675724	-0.74	0.459297	0.861975
vls	397.9759	1.487667	2.000996	0.743463	0.457201	0.861975
CG10068	821.1857	-0.99931	1.346153	-0.74234	0.457879	0.861975
CG11722	1057.056	0.760069	1.025077	0.741475	0.458405	0.861975
Leash	860.1093	-1.43585	1.928679	-0.74447	0.456591	0.861975
PK2-R1	222.6721	1.450718	1.960065	0.740138	0.459216	0.861975
CG14322	1016.098	1.304609	1.755286	0.743246	0.457333	0.861975
CG14285	72.4917	2.559092	3.456956	0.740273	0.459134	0.861975
Synd	3226.087	-0.51714	0.697004	-0.74195	0.458118	0.861975
C15	90.10606	2.814271	3.8107	0.738518	0.4602	0.861975
wda	429.0396	-1.37843	1.864764	-0.7392	0.459788	0.861975
CG2218	1367.267	0.643519	0.866791	0.742415	0.457836	0.861975
Tif-IA	2181.841	0.568916	0.768322	0.740465	0.459018	0.861975
CG32708	568.1927	1.262779	1.698897	0.743293	0.457304	0.861975
CG13204	721.5786	1.340348	1.812729	0.739409	0.459659	0.861975
Rs1	92.31406	-2.15237	2.908839	-0.73994	0.459335	0.861975
ChAT	9468.638	0.379369	0.512706	0.739935	0.459339	0.861975
Deaf1	3346.921	-0.51122	0.691034	-0.7398	0.459423	0.861975
Octbeta2R	4968.37	0.459049	0.619988	0.740416	0.459048	0.861975
spok	289.6518	1.40344	1.890121	0.742513	0.457776	0.861975
CG42524	578.2088	0.929637	1.254409	0.741095	0.458636	0.861975
thoc7	1605.448	0.633315	0.857391	0.738653	0.460117	0.861975
how	1008.447	0.747868	1.006747	0.742856	0.457569	0.861975
GckIII	1947.838	-0.57542	0.778989	-0.73867	0.460108	0.861975
Sec10	1249.101	-0.98809	1.337279	-0.73888	0.459977	0.861975
btsz	895.4318	0.93545	1.259068	0.74297	0.4575	0.861975
lncRNA:CR	195.6963	-1.64596	2.224336	-0.73998	0.459314	0.861975
LIMK1	1661.424	-0.76183	1.026613	-0.74208	0.458038	0.861975
Mpi	984.4733	-0.72755	0.98157	-0.74121	0.458563	0.861975
CG11134	579.0939	0.928536	1.255785	0.739407	0.45966	0.861975
hid	254.5332	-1.32064	1.77482	-0.7441	0.456816	0.861975
Klc	2617.3	-0.66836	0.904069	-0.73928	0.459739	0.861975
CG9515	73.17759	2.818852	3.821699	0.737591	0.460763	0.862828
CG1172	951.4316	-1.13907	1.54474	-0.73739	0.460887	0.862859
Pzl	203.3478	1.716711	2.328731	0.737188	0.461008	0.862885
CG9667	167.4295	1.596846	2.167644	0.736674	0.461321	0.863269
CG4592	500.5087	1.419294	1.927265	0.736429	0.461469	0.863296
zyd	13107.81	-0.45057	0.611938	-0.7363	0.46155	0.863296
Cand1	3345.244	0.487261	0.663374	0.734519	0.462632	0.864201
CG3744	753.2376	0.908066	1.236277	0.734517	0.462634	0.864201
sn	930.5069	-0.73527	1.000894	-0.73461	0.462575	0.864201
ric8a	364.2918	-1.4908	2.029845	-0.73444	0.46268	0.864201
MFS12	255.8831	-1.68345	2.291354	-0.7347	0.462523	0.864201
asRNA:CR4	138.1777	-2.41974	3.293533	-0.73469	0.462526	0.864201
CG1358	26972.94	-0.31103	0.423783	-0.73393	0.462992	0.864583
Rpn5	2253.914	-0.56074	0.76454	-0.73344	0.463293	0.864943

PTP-ER	1568.428	-0.90259	1.231948	-0.73265	0.463769	0.86543
RecQ5	1096.498	-0.8065	1.100591	-0.73279	0.463688	0.86543
CG15772	735.4628	1.276034	1.752237	0.728232	0.466472	0.865818
Ptth	238.3854	-2.04866	2.806871	-0.72987	0.465467	0.865818
isoQC	535.8507	-1.05741	1.453761	-0.72736	0.467006	0.865818
CG11241	345.5081	1.501731	2.054436	0.73097	0.464798	0.865818
Rat1	156.1233	1.523829	2.095178	0.727303	0.46704	0.865818
Megf8	829.4812	-1.17163	1.602919	-0.73093	0.464819	0.865818
strat	432.1612	1.249166	1.709917	0.730542	0.465059	0.865818
AdipoR	6508.612	0.395774	0.541665	0.730662	0.464985	0.865818
CG7006	251.5007	1.666505	2.279104	0.731211	0.464651	0.865818
CG31106	333.5251	-2.0464	2.807533	-0.7289	0.466064	0.865818
Bap170	351.1162	-1.13452	1.554696	-0.72974	0.465551	0.865818
Cdc7	2269.637	0.552184	0.755051	0.73132	0.464584	0.865818
CG18262	207.657	-1.82754	2.51137	-0.72771	0.466793	0.865818
CG1440	1127.306	0.806335	1.104527	0.730027	0.465373	0.865818
CG1789	936.4295	0.7557	1.039035	0.727309	0.467036	0.865818
CG30460	405.3079	1.029863	1.413261	0.728714	0.466176	0.865818
beta3GalT	377.6289	1.653008	2.25933	0.731636	0.464391	0.865818
Taf3	15219.38	-0.41879	0.573559	-0.73016	0.465295	0.865818
arm	10203.15	-0.37932	0.521556	-0.72728	0.467054	0.865818
alpha-Cat	10542.24	0.357139	0.489571	0.729493	0.4657	0.865818
scramb1	4457.623	0.459959	0.630939	0.729008	0.465997	0.865818
mgl	19648.73	-0.34204	0.469619	-0.72833	0.466414	0.865818
Tasp1	447.7153	1.375545	1.883757	0.730214	0.46526	0.865818
CG44158	109.6404	-2.09733	2.880745	-0.72805	0.466581	0.865818
p24-1	1479.993	1.184512	1.627991	0.727591	0.466864	0.865818
NFAT	9670.345	-0.33933	0.466279	-0.72774	0.466771	0.865818
Clc	3763.796	0.45176	0.619764	0.728922	0.466049	0.865818
roq	2200.973	0.5763	0.790112	0.72939	0.465763	0.865818
Mad	1700.614	-0.69912	0.961398	-0.7272	0.467106	0.865818
cype	21816.36	0.331367	0.456138	0.726461	0.467556	0.866292
CG10527	1713.825	-0.60569	0.834048	-0.7262	0.467713	0.866292
gprs	4586.849	0.460343	0.633808	0.726313	0.467647	0.866292
CG13912	81.16521	-2.78554	3.836434	-0.72607	0.467793	0.866292
sds22	692.6786	0.826424	1.139022	0.725556	0.468111	0.86668
CG9016	198.5481	1.718839	2.372388	0.724518	0.468748	0.867059
Kank	293.1902	-1.18582	1.636466	-0.72462	0.468684	0.867059
lncRNA:CR	309.5646	1.671672	2.307197	0.724547	0.46873	0.867059
KFase	551.9931	-1.20694	1.665065	-0.72486	0.468539	0.867059
CG31140	15416.83	-0.31071	0.429274	-0.72379	0.469192	0.867681
CG13654	827.4386	1.199314	1.658326	0.723208	0.469552	0.868147
kek1	3516.082	0.506856	0.701245	0.722794	0.469807	0.868417
Ufd1-like	1250.311	-0.76733	1.062258	-0.72236	0.470075	0.868714
su(f)	2180.149	0.525993	0.728528	0.721995	0.470298	0.868925
sta	13672.84	0.310531	0.431267	0.720042	0.471499	0.869056
CG12236	2467.654	0.775894	1.076719	0.72061	0.47115	0.869056
Sfxn1-3	909.1062	-1.13673	1.579693	-0.71959	0.471777	0.869056
srl	114.1352	-2.40913	3.346028	-0.72	0.471527	0.869056
mRpL1	1287.344	0.693352	0.962694	0.72022	0.47139	0.869056

Coq2	324.0969	1.133022	1.575661	0.719077	0.472093	0.869056
CG9346	334.2554	1.251308	1.739958	0.71916	0.472042	0.869056
CG4744	301.6825	2.056866	2.855447	0.720331	0.471321	0.869056
CG33774	502.2453	0.930122	1.293518	0.719064	0.472101	0.869056
CG44002	190.847	-2.39399	3.324807	-0.72004	0.471501	0.869056
ghi	4765.186	0.523551	0.725691	0.721452	0.470632	0.869056
Nadsyn	1444.45	0.733974	1.02058	0.719174	0.472034	0.869056
TrpRS-m	2422.768	0.650042	0.903462	0.719501	0.471832	0.869056
CG32032	5507.072	-0.46568	0.647296	-0.71943	0.471876	0.869056
CG7506	1592.777	-0.63827	0.884888	-0.7213	0.470724	0.869056
MCU	5212.421	0.389647	0.540491	0.720914	0.470962	0.869056
TkR86C	332.7744	-1.61189	2.24347	-0.71848	0.472461	0.869334
CG42709	5229.074	0.392902	0.54686	0.718468	0.472469	0.869334
CG17266	196.8683	2.076025	2.890519	0.718219	0.472622	0.869339
Srp54	1482.385	-0.61734	0.859796	-0.71801	0.472751	0.869339
CG9164	3113.807	0.884972	1.232661	0.717937	0.472796	0.869339
Eaat1	17212.89	-0.34021	0.474152	-0.71751	0.473059	0.869424
JIL-1	2978.579	0.540989	0.753813	0.717669	0.472961	0.869424
olf413	4741.073	0.404275	0.564588	0.716054	0.473958	0.870115
l(3)73Ah	998.7254	-1.0645	1.486412	-0.71615	0.473896	0.870115
Snmp1	171.1881	-2.00774	2.803188	-0.71624	0.473846	0.870115
Ih	28262.11	0.280089	0.391108	0.716144	0.473902	0.870115
ZC3H3	1124.442	-0.75462	1.053901	-0.71602	0.473977	0.870115
CG16711	1532.531	-0.60294	0.842306	-0.71582	0.474101	0.870142
CG8078	456.8956	1.201679	1.679345	0.715564	0.474261	0.870237
Vinc	81.8558	2.351565	3.291413	0.714455	0.474946	0.870303
Dlg5	762.336	-1.07492	1.503155	-0.71511	0.47454	0.870303
Mondo	2102.585	0.594147	0.831863	0.714237	0.475081	0.870303
Bub3	491.2479	-1.00919	1.412761	-0.71434	0.475016	0.870303
RpL21	15608.68	-0.31698	0.443885	-0.7141	0.475164	0.870303
rdx	5867.545	-0.41109	0.575059	-0.71487	0.474689	0.870303
lncRNA:CR	337.3348	1.335376	1.868534	0.714665	0.474816	0.870303
CG10960	13425.29	-0.33669	0.470736	-0.71525	0.474455	0.870303
CG2930	667.9567	0.882595	1.243356	0.70985	0.477797	0.870445
CG9586	611.4483	1.256013	1.765431	0.711448	0.476806	0.870445
CG10561	1197.625	0.679956	0.955765	0.711426	0.47682	0.870445
TotF	62.35554	2.776096	3.916444	0.708831	0.47843	0.870445
CG1943	2618.382	0.525783	0.738704	0.711764	0.476611	0.870445
ATP6AP2	16297.86	-0.37337	0.52424	-0.71221	0.476333	0.870445
Best1	1540.894	0.710753	0.997344	0.712646	0.476065	0.870445
Ns1	1007.388	0.914158	1.288004	0.709748	0.47786	0.870445
CG14329	110.4822	2.778428	3.91592	0.709521	0.478001	0.870445
CG14309	81.92009	2.777373	3.916157	0.709209	0.478195	0.870445
CG4000	247.1478	1.978824	2.790699	0.709078	0.478276	0.870445
CG13841	363.3178	-1.13023	1.590644	-0.71055	0.477364	0.870445
CG13829	532.2764	1.757596	2.469564	0.711703	0.476649	0.870445
mRpS18C	1132.804	0.791393	1.111281	0.712145	0.476375	0.870445
CG1544	91.78695	2.423896	3.397824	0.713367	0.475619	0.870445
CG13868	12198.44	0.38803	0.544474	0.71267	0.47605	0.870445
UQCR-6.4	7725.56	0.351239	0.494482	0.710317	0.477507	0.870445

CG18870	926.1136	-1.31042	1.84441	-0.71048	0.477406	0.870445
Cyp6t3	108.1805	2.778364	3.915934	0.709502	0.478013	0.870445
kel	1580.363	-0.59806	0.841033	-0.7111	0.47702	0.870445
CG15394	69.46992	2.776642	3.916321	0.708992	0.478329	0.870445
vlc	2110.33	0.679055	0.953782	0.71196	0.476489	0.870445
snRNP-U1-	870.2884	-1.22059	1.717934	-0.7105	0.477397	0.870445
RanBPM	1544.641	-0.77318	1.09094	-0.70872	0.478496	0.870445
CG3611	95.83498	2.777965	3.916024	0.709384	0.478086	0.870445
lncRNA:CR	75.74732	2.777041	3.916231	0.709111	0.478256	0.870445
lncRNA:CR	81.71084	2.777362	3.916159	0.709206	0.478197	0.870445
hop	129.046	-2.08353	2.927827	-0.71163	0.476694	0.870445
mew	263.7246	-1.25723	1.769808	-0.71038	0.47747	0.870445
Atg18a	2698.119	-0.57963	0.815965	-0.71036	0.477479	0.870445
CG10333	606.2395	0.978401	1.381118	0.708413	0.478689	0.8706
Bka	863.5811	0.899143	1.271502	0.70715	0.479473	0.870682
CG34155	737.996	0.89036	1.258279	0.707602	0.479192	0.870682
lap	10568.12	0.320379	0.452868	0.707445	0.47929	0.870682
lqfR	2162.961	-0.60038	0.84899	-0.70717	0.47946	0.870682
CG13894	918.2414	-1.18778	1.679369	-0.70728	0.479395	0.870682
lncRNA:CR	213.0656	-2.02435	2.862156	-0.70728	0.479391	0.870682
Ork1	7506.966	-0.37146	0.525417	-0.70699	0.479574	0.870682
Mgat4a	3564.169	-0.4818	0.681526	-0.70694	0.479602	0.870682
CG12155	867.7792	0.728017	1.030976	0.706143	0.480099	0.871388
CG9123	486.3172	-1.23962	1.756416	-0.70577	0.480333	0.871615
NP15.6	7823.966	-0.33986	0.482184	-0.70484	0.480909	0.871986
krz	2035.097	0.593706	0.842025	0.705093	0.480752	0.871986
Spt5	2549.336	-0.48409	0.6869	-0.70474	0.480972	0.871986
RhoGDI	4226.278	-0.43624	0.619006	-0.70474	0.48097	0.871986
santa-mari	2022.796	0.598092	0.849148	0.704343	0.481219	0.872113
CCT7	1233.138	-0.92237	1.309991	-0.7041	0.481368	0.872113
AsnRS	918.6662	0.790754	1.12282	0.704257	0.481272	0.872113
CG17075	1300.62	0.680115	0.96699	0.703332	0.481849	0.872787
RpL38	25366.78	0.291965	0.415235	0.703132	0.481973	0.872815
yip2	2295.467	-0.58865	0.837778	-0.70263	0.482287	0.873167
jing	4196.093	0.450905	0.642043	0.702297	0.482494	0.873167
CG13667	639.8061	1.368221	1.947838	0.702431	0.482411	0.873167
ttm50	1081.345	-0.70808	1.008838	-0.70188	0.482755	0.873442
CG4281	1168.981	0.758288	1.081024	0.701453	0.48302	0.873528
eIF3h	3359.505	-0.46476	0.662423	-0.70161	0.482923	0.873528
mRpL33	912.629	0.737065	1.052829	0.70008	0.483877	0.874094
CanA1	90.63127	2.742304	3.916307	0.700227	0.483786	0.874094
Rcd5	1448.192	-0.7422	1.059537	-0.7005	0.483617	0.874094
CG9705	906.113	-0.73967	1.056192	-0.70032	0.483729	0.874094
Tfb4	252.3778	-1.77879	2.539437	-0.70047	0.483636	0.874094
CG7519	1128.192	0.661295	0.946193	0.698901	0.484614	0.87416
RpL24-like	8916.794	0.358102	0.512905	0.698184	0.485062	0.87416
mor	338.5906	1.34884	1.931677	0.698274	0.485006	0.87416
CG17786	295.0198	-1.18696	1.701348	-0.69766	0.485389	0.87416
Tsp42Ed	905.318	-1.47303	2.110282	-0.69803	0.48516	0.87416
CG3184	85.98222	-2.34897	3.362316	-0.69862	0.484792	0.87416

ReepB	3919.519	0.415002	0.594678	0.69786	0.485265	0.87416
beta4GalN	1332.037	-1.2111	1.735258	-0.69794	0.485216	0.87416
Mat1	529.9489	-0.98152	1.403744	-0.69921	0.484419	0.87416
Gdh	9776.842	0.351677	0.502536	0.699805	0.484049	0.87416
ktub	1728.018	-0.69222	0.989646	-0.69947	0.48426	0.87416
GlyP	5489.269	-0.43841	0.628468	-0.69758	0.485439	0.87416
Nulp1	1437.205	-1.01737	1.456462	-0.69852	0.484851	0.87416
CG12795	191.7762	-1.71554	2.457523	-0.69808	0.485128	0.87416
Pfrx	1569.982	0.689502	0.989366	0.696913	0.485857	0.874717
5-HT7	2697.434	0.745719	1.071155	0.696182	0.486315	0.875223
Sec63	1928.16	-0.54408	0.7816	-0.69612	0.486356	0.875223
CG16790	195.5748	-1.88808	2.71652	-0.69503	0.487033	0.876128
Galphaq	4311.102	0.493054	0.70957	0.694863	0.487141	0.876128
Imp	5400.698	0.378174	0.544299	0.694791	0.487187	0.876128
CG3594	666.4484	1.209138	1.740894	0.69455	0.487337	0.876202
Asap	3072.738	0.48361	0.696699	0.694145	0.487592	0.876464
Baldspot	6693.328	-0.36227	0.522343	-0.69354	0.487971	0.876598
lncRNA:CR	1227.98	-0.70649	1.018286	-0.6938	0.487808	0.876598
Helz	3655.987	-0.48742	0.702836	-0.6935	0.487994	0.876598
CG14803	112.8897	-2.09504	3.022301	-0.69319	0.488188	0.876626
CG5746	2260.905	0.522557	0.754098	0.692956	0.488337	0.876626
ND-24	6316.163	0.400735	0.578275	0.692983	0.48832	0.876626
snoRNA:Ps	122.838	-2.27792	3.289796	-0.69242	0.488674	0.877035
Nepl5	11707.23	-0.39734	0.574052	-0.69217	0.488829	0.877116
Slik	2823.688	-0.49784	0.719909	-0.69153	0.489232	0.877644
sgg	8859.262	0.353384	0.511641	0.690687	0.489762	0.87801
CG14671	900.2995	-1.1111	1.609705	-0.69025	0.490035	0.87801
VhaPPA1-1	11852.06	-0.36064	0.522411	-0.69033	0.489986	0.87801
PIG-O	113.9266	2.047494	2.968183	0.689814	0.490311	0.87801
Nsun5	566.2828	1.59338	2.308007	0.69037	0.489961	0.87801
Usp10	2035.399	-0.51876	0.751867	-0.68996	0.490221	0.87801
CG8745	16053.44	-0.34726	0.502553	-0.69099	0.489573	0.87801
unc-13-4A	3410.095	-0.48709	0.70578	-0.69014	0.490104	0.87801
Scox	1829.719	-0.69672	1.010568	-0.68943	0.490551	0.878242
CG33129	5024.269	0.391359	0.567988	0.689026	0.490807	0.878343
CG18659	3220.271	0.475326	0.689882	0.688996	0.490826	0.878343
Ugt35E2	298.2688	1.330328	1.934123	0.68782	0.491566	0.87899
Ctf4	136.6344	2.139298	3.108678	0.68817	0.491346	0.87899
CG42674	2977.239	0.481909	0.700504	0.687946	0.491487	0.87899
Vps16A	1212.949	-1.03854	1.510105	-0.68773	0.491625	0.87899
CG31109	2165.404	-0.69943	1.017326	-0.68752	0.491756	0.879028
dl	2486.689	-0.51277	0.746293	-0.6871	0.492023	0.879308
MTPAP	351.473	-1.24106	1.806941	-0.68683	0.492189	0.87941
CG11562	437.2516	1.219612	1.776423	0.686555	0.492363	0.879525
stas	6163.991	0.39859	0.580824	0.686249	0.492556	0.879674
pr	1613.879	-0.99343	1.448213	-0.68597	0.492733	0.879795
ab	6457.452	-0.34908	0.509099	-0.68567	0.492919	0.879852
CG1532	848.6831	0.744417	1.085836	0.68557	0.492984	0.879852
Nop17l	3338.866	-0.43344	0.632412	-0.68537	0.49311	0.87988
Cyp9f3	506.8592	-1.50663	2.200423	-0.6847	0.493534	0.880068

Coq8	279.4568	-1.51125	2.20723	-0.68468	0.493545	0.880068
CG32069	259.724	-1.18904	1.736256	-0.68483	0.493453	0.880068
D2hgdh	792.4445	-1.37627	2.01582	-0.68274	0.494774	0.880128
CG4766	174.6119	1.897577	2.782705	0.681918	0.495291	0.880128
siz	5973.516	-0.39279	0.57604	-0.68188	0.495315	0.880128
MICU3	2628.226	-0.47502	0.695089	-0.6834	0.494355	0.880128
CG5646	277.0234	-1.6314	2.388806	-0.68293	0.494648	0.880128
Tango5	1779.022	-0.53203	0.778482	-0.68342	0.494344	0.880128
Magi	533.0187	1.135385	1.665149	0.681852	0.495333	0.880128
Tom7	3861.672	0.412428	0.604214	0.682586	0.494868	0.880128
Dhit	973.9577	-0.66856	0.980751	-0.68168	0.495442	0.880128
CG33695	455.6287	1.330857	1.946543	0.683703	0.494163	0.880128
nuf	7112.881	-0.40288	0.589849	-0.68302	0.494597	0.880128
Pak	3779.02	-0.45626	0.668862	-0.68215	0.495146	0.880128
SRPK	5156.383	-0.37077	0.543431	-0.68227	0.495066	0.880128
CG1622	1618.364	0.921308	1.350858	0.682017	0.495228	0.880128
Fim	6011.146	0.380048	0.557474	0.681732	0.495408	0.880128
CG14142	217.497	-1.53371	2.242154	-0.68404	0.493953	0.880128
Fnta	429.794	-1.07213	1.568391	-0.68359	0.494236	0.880128
CG10514	4214.79	-0.51165	0.751064	-0.68124	0.495722	0.880154
snoRNA:Ps	65.76418	2.259255	3.317497	0.681012	0.495864	0.880154
Mdh1	12142.72	-0.31421	0.461141	-0.68137	0.495635	0.880154
eIF3l	5723.569	0.389908	0.572583	0.680963	0.495895	0.880154
PGAP1	558.2778	1.402079	2.062675	0.679738	0.49667	0.880163
chic	7390.511	-0.35797	0.526032	-0.68051	0.496181	0.880163
SppL	2781.71	0.763161	1.121839	0.680277	0.496329	0.880163
HLH4C	140.9175	-2.28114	3.351829	-0.68057	0.496146	0.880163
Cadps	29351.7	0.273884	0.403027	0.679568	0.496778	0.880163
mIF3	327.3981	1.639156	2.410654	0.679963	0.496528	0.880163
lncRNA:CR	115.6679	-2.40641	3.540489	-0.67968	0.496705	0.880163
FMRFaR	172.8542	-1.89801	2.791987	-0.67981	0.496627	0.880163
CG10584	797.0386	1.112457	1.640445	0.678144	0.497681	0.881419
CG31211	2521.042	-0.48789	0.72044	-0.67721	0.498276	0.881419
dpr17	3099.482	0.457324	0.676	0.676515	0.498714	0.881419
CG7518	1783.738	-0.53262	0.787874	-0.67602	0.499027	0.881419
twf	605.583	1.130573	1.672659	0.675914	0.499095	0.881419
Hydr1	4749.054	-0.37303	0.551456	-0.67645	0.498758	0.881419
Clk	813.9843	-1.00642	1.486648	-0.67697	0.498423	0.881419
spen	53433.15	-0.26181	0.387218	-0.67614	0.498951	0.881419
Gapdh1	27711.49	-0.34117	0.503858	-0.67712	0.498329	0.881419
side-II	5039.642	0.405926	0.599621	0.676972	0.498424	0.881419
Vha68-2	2230.218	-0.63003	0.929221	-0.67802	0.49776	0.881419
slmb	3599.668	-0.52695	0.779685	-0.67585	0.499134	0.881419
trol	27066.45	-0.27892	0.412201	-0.67665	0.498629	0.881419
CG32243	1142.397	0.673091	0.994525	0.676797	0.498535	0.881419
Srp19	1328.081	0.693135	1.023598	0.677156	0.498307	0.881419
5-HT1A	1245.088	-0.6278	0.929208	-0.67563	0.499277	0.881478
Rassf	854.733	-0.76619	1.135238	-0.67491	0.499731	0.881891
CG43789	678.0457	-0.86629	1.283414	-0.67499	0.499685	0.881891
RhoGAP16	398.6423	1.497334	2.220428	0.674345	0.500092	0.882335

CG14814	122.5246	2.0063	2.977131	0.673904	0.500372	0.882513
zip	2042.099	0.556142	0.825332	0.67384	0.500413	0.882513
Gasp	231.7414	-2.10952	3.132572	-0.67341	0.500684	0.882602
Mad1	233.0724	1.19502	1.774219	0.673547	0.500599	0.882602
CCHa2-R	238.1434	1.809941	2.689769	0.672898	0.501012	0.882987
CG7800	824.7562	0.837388	1.245967	0.672079	0.501533	0.883076
Dlc90F	3564.354	0.485418	0.723125	0.671278	0.502043	0.883076
CG6425	429.6763	1.402932	2.089648	0.671373	0.501983	0.883076
Mgat1	442.2526	1.186069	1.766924	0.671262	0.502054	0.883076
CG3032	339.0434	-2.11743	3.15304	-0.67155	0.501868	0.883076
Rbpn-5	90.93689	1.765634	2.628639	0.671691	0.50178	0.883076
CG8248	3271.454	0.846802	1.260653	0.671717	0.501764	0.883076
mt:ATPase	3180.874	0.438632	0.653611	0.67109	0.502163	0.883076
mbf1	4987.675	-0.39784	0.592395	-0.67157	0.501856	0.883076
CG3530	5421.837	-0.43456	0.646543	-0.67213	0.501501	0.883076
CG33217	1640.318	0.544133	0.812969	0.669317	0.503293	0.883397
Rtnl1	23718.38	0.298002	0.445075	0.669555	0.503141	0.883397
CG10600	3358.068	0.542759	0.810972	0.669269	0.503324	0.883397
CG5515	868.0496	0.743905	1.110322	0.669991	0.502864	0.883397
CG11893	243.1354	-1.63819	2.447635	-0.6693	0.503307	0.883397
Cyp6a9	467.7191	-1.43583	2.143444	-0.66987	0.502941	0.883397
RN-tre	2358.836	0.473611	0.707856	0.669079	0.503445	0.883397
Asx	3924.241	0.522813	0.781394	0.669077	0.503446	0.883397
Ssrp	664.5239	-0.81769	1.220659	-0.66988	0.502935	0.883397
vib	11220.62	0.362005	0.540105	0.670248	0.502699	0.883397
Smyd3	217.5945	1.651288	2.471404	0.668158	0.504033	0.883545
Nhe3	6856.702	0.489093	0.733111	0.667148	0.504678	0.883545
rk	114.411	-1.90514	2.853403	-0.66767	0.504343	0.883545
by	483.7079	-1.11658	1.675644	-0.66636	0.505181	0.883545
CG31278	345.3176	1.650734	2.473714	0.66731	0.504574	0.883545
CG9588	364.7077	1.19687	1.795008	0.666777	0.504915	0.883545
CSN6	1092.061	1.094836	1.638365	0.668249	0.503975	0.883545
side	3964.271	-0.39388	0.590135	-0.66744	0.504489	0.883545
CG1902	4202.253	-0.39633	0.594773	-0.66636	0.505182	0.883545
Shrm	131.7915	1.566669	2.344281	0.668294	0.503946	0.883545
pio	63.72663	2.590077	3.882535	0.66711	0.504702	0.883545
Dis3l2	1715.283	-0.70704	1.060589	-0.66665	0.504995	0.883545
Trs31	1144.001	-0.67889	1.016469	-0.66789	0.504203	0.883545
CG10483	359.9784	1.026865	1.539927	0.666827	0.504883	0.883545
CG9886	690.4774	0.934603	1.398378	0.668348	0.503911	0.883545
CG3262	6082.814	-0.38532	0.578896	-0.66562	0.505654	0.883986
CG8818	3856.291	0.447825	0.672732	0.665681	0.505615	0.883986
Or30a	273.935	-1.63471	2.459614	-0.66462	0.506293	0.884871
PIG-C	98.99526	-2.28367	3.43736	-0.66437	0.506455	0.884871
Rtc1	1331.301	1.146332	1.725594	0.664311	0.506491	0.884871
MED1	510.4732	0.856758	1.290281	0.664008	0.506685	0.885017
PKD	1449.227	0.636483	0.958818	0.663821	0.506805	0.885022
Uggt	2973.576	0.553512	0.83403	0.663659	0.506908	0.885022
alien	1510.719	-0.69478	1.051921	-0.66049	0.508942	0.885874
alphaTub8	43825.18	0.291277	0.440925	0.660604	0.508866	0.885874

tws	3680.37	-0.3995	0.604137	-0.66128	0.508434	0.885874
ChT	4152.264	0.435412	0.658791	0.660926	0.50866	0.885874
CenB1A	558.5544	-1.374	2.074456	-0.66234	0.507752	0.885874
RpS3	11486.99	0.336706	0.508379	0.662312	0.507771	0.885874
APC4	233.1734	1.545792	2.337615	0.661269	0.50844	0.885874
kermit	655.6254	-1.20883	1.828115	-0.66125	0.508454	0.885874
hpRNA:CR:	196.7599	-1.97848	2.993834	-0.66085	0.508707	0.885874
Tina-1	5675.052	-0.36873	0.556863	-0.66216	0.507867	0.885874
hang	2798.242	-0.46388	0.701734	-0.66104	0.508585	0.885874
rudhira	14132.53	-0.32276	0.488554	-0.66065	0.508835	0.885874
Lsm12a	1695.018	0.549803	0.831085	0.661548	0.508261	0.885874
CrzR	160.4071	2.113745	3.193935	0.6618	0.5081	0.885874
Wdr92	589.3077	1.34115	2.031182	0.660281	0.509074	0.885912
or	1050.469	-0.71063	1.076816	-0.65994	0.509296	0.886106
Dci	935.275	0.654841	0.992955	0.659487	0.509583	0.886414
Usp2	11145.35	-0.33722	0.511995	-0.65865	0.510123	0.887162
brat	4817.172	-0.37215	0.56535	-0.65827	0.510365	0.887389
ppk8	234.6271	-1.58804	2.415106	-0.65755	0.51083	0.887925
bif	1031.024	0.738548	1.123604	0.657302	0.510986	0.887925
Gapdh2	46520.3	-0.27162	0.413251	-0.65727	0.511005	0.887925
CG43737	8824.466	-0.37424	0.569563	-0.65707	0.511134	0.887957
PIG-Wa	216.525	-2.08538	3.176024	-0.6566	0.511438	0.888293
Dscam1	38509.39	-0.31336	0.477465	-0.65631	0.511626	0.888372
Cyp6v1	229.1599	1.535181	2.340657	0.655876	0.511904	0.888372
Mp	5208.872	0.402398	0.61356	0.655841	0.511926	0.888372
CG7083	2724.6	-0.43363	0.660948	-0.65607	0.511777	0.888372
CG6388	477.3365	-1.51131	2.305138	-0.65563	0.512065	0.888421
CG31191	1250.56	-0.92404	1.409771	-0.65545	0.512177	0.888423
CG11885	510.7569	0.864632	1.320304	0.654874	0.512549	0.888564
Naus	3495.989	-0.45797	0.699427	-0.65478	0.512609	0.888564
ATPsynF	30609.92	0.281914	0.430868	0.654293	0.512923	0.888564
CG44812	136.3844	-1.98554	3.034563	-0.65431	0.512913	0.888564
shop	462.7911	1.20735	1.844998	0.654391	0.51286	0.888564
CG17260	135.8309	-1.45986	2.229415	-0.65482	0.512586	0.888564
Ilp6	3115.925	0.435557	0.666398	0.653599	0.51337	0.888585
e(r)	2443.288	0.491586	0.75191	0.653782	0.513252	0.888585
Zpr1	203.8763	2.139034	3.275163	0.653108	0.513687	0.888585
SmF	666.5271	0.747534	1.143892	0.653501	0.513434	0.888585
eIF4H1	2293.274	0.456827	0.698414	0.654092	0.513053	0.888585
Fatp3	1499.472	0.588694	0.901224	0.653216	0.513617	0.888585
Pfdn4	1640.999	-0.56624	0.867035	-0.65307	0.51371	0.888585
shv	859.0897	-1.07019	1.640431	-0.65238	0.514154	0.8887
CG30423	4796.631	0.406037	0.62265	0.65211	0.51433	0.8887
Spt20	629.2001	1.11443	1.707225	0.652772	0.513903	0.8887
nkd	810.4714	-0.69127	1.059616	-0.65237	0.51416	0.8887
CG13685	2118.126	-0.63879	0.979321	-0.65228	0.514222	0.8887
CG12081	309.5859	1.300194	1.996072	0.651376	0.514804	0.888753
lin	261.8796	1.534477	2.355055	0.651567	0.51468	0.888753
side-V	1834.511	-0.49919	0.766046	-0.65164	0.514634	0.888753
Trpm	3347.638	0.745977	1.145156	0.65142	0.514775	0.888753

red	4932.723	0.373861	0.574176	0.651126	0.514965	0.888841
UGP	2307.477	-0.61376	0.942889	-0.65094	0.515087	0.888859
Lis-1	10161.17	0.354308	0.544781	0.650368	0.515455	0.889303
Herp	3067.867	0.444724	0.683996	0.650186	0.515572	0.889315
CG18302	111.5381	-2.41411	3.716026	-0.64965	0.515919	0.88934
Lapsyn	1935.269	0.591635	0.910671	0.649669	0.515906	0.88934
CG10737	4211.663	0.42	0.646218	0.649936	0.515734	0.88934
CG13693	211.1265	1.734953	2.679615	0.647464	0.517332	0.889552
CG2091	452.9364	-1.26008	1.944934	-0.64788	0.517063	0.889552
Aldh	4072.175	-0.5315	0.820978	-0.64739	0.517377	0.889552
ova	3090.263	0.524871	0.809419	0.648454	0.516691	0.889552
sqz	291.7765	1.391306	2.149233	0.64735	0.517406	0.889552
lncRNA:CR	90826.26	0.248756	0.383372	0.648864	0.516426	0.889552
jigr1	4342.549	-0.45755	0.706887	-0.64728	0.517452	0.889552
CG12065	1028.62	0.713082	1.102035	0.647059	0.517594	0.889552
Atg8a	40053.62	0.252427	0.389189	0.648598	0.516598	0.889552
mtDNA-he	251.2539	-1.93014	2.974197	-0.64896	0.516363	0.889552
CG33228	443.0396	1.309923	2.023622	0.647316	0.517427	0.889552
sel	754.1414	-0.86843	1.341445	-0.64738	0.517384	0.889552
Aplip1	10321.37	0.296504	0.457626	0.647918	0.517038	0.889552
IntS2	235.5926	-1.44359	2.230643	-0.64716	0.517526	0.889552
wcd	358.3138	1.52472	2.359949	0.646082	0.518226	0.890285
MsR2	167.8904	1.902454	2.944713	0.646058	0.518242	0.890285
Fcp3C	132.2965	1.887839	2.938668	0.642413	0.520605	0.89029
CG3097	767.4125	0.870599	1.353592	0.643177	0.520109	0.89029
CG4788	182.9174	-1.71273	2.665164	-0.64264	0.520461	0.89029
CG10343	474.2308	-1.28892	2.004357	-0.64306	0.520185	0.89029
CG3337	1950.513	-0.5131	0.796479	-0.6442	0.519443	0.89029
fh	387.9489	-1.08513	1.688804	-0.64254	0.520522	0.89029
RpS28b	14244.88	0.301483	0.467075	0.64547	0.518623	0.89029
Cpr51A	115.3776	1.547905	2.409983	0.642289	0.520686	0.89029
Dad	1692.974	0.64111	0.994648	0.644559	0.519213	0.89029
CG34025	387.3742	0.936321	1.456258	0.642964	0.520248	0.89029
Pde11	4707.575	0.355436	0.55174	0.644209	0.51944	0.89029
bma	2258.817	0.567071	0.880595	0.643963	0.519599	0.89029
par-1	14433.05	0.294427	0.457484	0.643579	0.519848	0.89029
RpS29	23440.04	0.274818	0.426851	0.643826	0.519688	0.89029
SmydA-3	2281.645	0.511667	0.795552	0.643159	0.520121	0.89029
milt	5844.845	0.340381	0.528946	0.643507	0.519895	0.89029
Cpes	558.8558	-1.18374	1.840452	-0.64318	0.520108	0.89029
lncRNA:CR	153.4766	1.82707	2.832805	0.644968	0.518948	0.89029
Ric	8019.51	0.35737	0.553365	0.645813	0.518401	0.89029
RpL28	16371.08	0.277441	0.430547	0.644393	0.519321	0.89029
gig	776.4122	-0.72511	1.123071	-0.64565	0.518505	0.89029
CG6321	542.1382	1.112829	1.729801	0.643327	0.520012	0.89029
Top1	2256.4	-0.50171	0.781678	-0.64184	0.520977	0.890599
CG13887	3238.517	0.500985	0.780769	0.641656	0.521097	0.890614
CG32982	5537.209	0.339508	0.53022	0.640315	0.521968	0.891154
holn1	648.7415	-0.80933	1.262643	-0.64098	0.521536	0.891154
ck	1328.715	-0.699	1.094528	-0.63863	0.523061	0.891154

Faf2	2042.127	0.710092	1.111734	0.638724	0.523002	0.891154
CG4565	399.5587	1.574563	2.472074	0.63694	0.524164	0.891154
ATPsynCF6	14138.89	0.289204	0.453074	0.638314	0.523269	0.891154
mask	13134.52	0.283256	0.44421	0.637663	0.523693	0.891154
Alp4	5333.304	-0.56127	0.880517	-0.63743	0.523843	0.891154
RpS15	17015.54	0.270848	0.423973	0.638832	0.522933	0.891154
fra	649.9628	-0.96014	1.502323	-0.6391	0.522757	0.891154
CG13185	89.49654	1.987939	3.106859	0.639855	0.522267	0.891154
CG9170	2736.51	-0.46418	0.727895	-0.6377	0.523672	0.891154
Sirup	11298.58	-0.36261	0.567793	-0.63862	0.523068	0.891154
CG17816	12645.92	0.288148	0.452074	0.63739	0.523871	0.891154
gammaTul	1152.671	0.968492	1.511431	0.640778	0.521667	0.891154
osa	5983.942	-0.35907	0.561744	-0.6392	0.522695	0.891154
CG43066	12333.65	-0.31956	0.501447	-0.63727	0.523949	0.891154
PheRS-m	1820.385	0.826284	1.293495	0.638799	0.522953	0.891154
Socs16D	1813.676	0.528469	0.826134	0.639689	0.522375	0.891154
CG7332	2485.781	-0.45238	0.709986	-0.63716	0.52402	0.891154
MED19	797.2735	1.035888	1.624643	0.63761	0.523728	0.891154
mRpS31	1235.245	0.909312	1.426322	0.637523	0.523784	0.891154
CG10077	63120.96	0.240746	0.377958	0.636964	0.524148	0.891154
Txl	981.9813	-1.02089	1.602904	-0.6369	0.524189	0.891154
CG31663	14071.79	-0.3486	0.547135	-0.63714	0.524033	0.891154
ND-B14.5A	5627.698	-0.3467	0.549746	-0.63066	0.528262	0.891536
Rala	1848.61	-0.48804	0.770129	-0.63371	0.52627	0.891536
Nepl11	219.5357	1.49785	2.375617	0.63051	0.528361	0.891536
SelT	3492.012	0.383911	0.606625	0.632863	0.526823	0.891536
Hel25E	4534.175	-0.47068	0.747367	-0.62979	0.528834	0.891536
Pex19	4833.979	-0.45257	0.71372	-0.6341	0.526014	0.891536
Droj2	12246.8	-0.28414	0.448595	-0.63339	0.526479	0.891536
CG31287	147.5744	-2.31012	3.639166	-0.63479	0.525564	0.891536
CG16953	6151.41	-0.40833	0.642397	-0.63564	0.525014	0.891536
CG10175	264.4358	1.590733	2.516047	0.632235	0.527233	0.891536
GILT3	2127.439	-0.6521	1.028309	-0.63415	0.525981	0.891536
CG4743	455.0622	0.899369	1.419738	0.633476	0.526423	0.891536
CG31028	707.0506	0.736569	1.157613	0.636283	0.524592	0.891536
CG15097	299.1076	-1.0945	1.729263	-0.63293	0.52678	0.891536
CCHa1-R	273.9262	-1.56022	2.459172	-0.63445	0.525787	0.891536
Gclc	4474.919	-0.4553	0.72149	-0.63105	0.528008	0.891536
Prx2540-1	74.94732	2.353638	3.731275	0.630786	0.52818	0.891536
Sec24AB	2471.146	0.448919	0.709693	0.632554	0.527025	0.891536
CG8243	689.0491	-0.81405	1.291538	-0.6303	0.528501	0.891536
anne	27370.16	0.249593	0.396013	0.630266	0.528521	0.891536
qtc	2659.401	0.55017	0.873357	0.629948	0.528728	0.891536
CG12179	121.4534	-1.73236	2.749308	-0.63011	0.528624	0.891536
CG3662	66493.18	0.303715	0.481903	0.630241	0.528537	0.891536
CG34188	87.23839	-2.07606	3.267716	-0.63533	0.525216	0.891536
CG43078	1731.552	-0.53763	0.850597	-0.63206	0.527345	0.891536
wmd	1206.188	0.712795	1.126467	0.632771	0.526883	0.891536
Stoml2	2061.384	-0.48779	0.771338	-0.63239	0.527132	0.891536
lncRNA:CR	79.22575	1.943647	3.081574	0.630732	0.528216	0.891536

asRNA:CR4	76.64115	1.904662	3.016281	0.63146	0.52774	0.891536
Fhos	4993.53	-0.44916	0.70991	-0.6327	0.526932	0.891536
Ttd14	2887.71	0.448081	0.708248	0.632661	0.526955	0.891536
CG8001	416.2266	0.940502	1.494082	0.629485	0.529032	0.891536
PGRP-SA	378.7193	-1.93971	3.05623	-0.63467	0.525641	0.891536
CG15760	2318.174	0.448189	0.706875	0.634042	0.526053	0.891536
sun	9613.708	0.300555	0.476001	0.631416	0.527769	0.891536
CG5613	138.0225	-1.94208	3.070054	-0.63259	0.527002	0.891536
Ranbp21	802.1846	0.81074	1.281851	0.632476	0.527076	0.891536
CG14183	140.1748	-2.04608	3.238747	-0.63175	0.52755	0.891536
CG5895	8815.409	0.336792	0.53021	0.635205	0.525295	0.891536
CG8833	79.46504	2.417038	3.816191	0.633364	0.526496	0.891536
nkt	32455.36	-0.27926	0.443682	-0.62941	0.529079	0.891536
Dim1	856.4791	-0.99685	1.58339	-0.62956	0.528979	0.891536
CG12054	3752.223	0.416573	0.662235	0.62904	0.529323	0.891573
CG14669	4583.407	0.422209	0.671044	0.629182	0.52923	0.891573
Vha14-1	13091.77	-0.32352	0.514507	-0.6288	0.52948	0.891651
Rab1	3375.449	0.393381	0.626817	0.627585	0.530276	0.892803
sei	2355.386	-0.76022	1.211727	-0.62739	0.530405	0.892834
dia	2019.645	-0.51927	0.828905	-0.62645	0.53102	0.89312
TAF1B	129.7998	2.039727	3.253702	0.626894	0.530729	0.89312
Hexim	1571.986	0.867751	1.384624	0.626705	0.530852	0.89312
scaf6	2101.305	0.556649	0.888387	0.626584	0.530932	0.89312
rst	500.7574	-0.92898	1.483619	-0.62616	0.53121	0.893251
CG6621	369.9172	0.927056	1.481021	0.625957	0.531343	0.893289
CG10132	3044.584	0.395987	0.632852	0.625718	0.5315	0.893365
pch2	755.0151	-0.71531	1.143901	-0.62533	0.531755	0.893607
CG16791	11999.18	0.372407	0.596621	0.624195	0.5325	0.894109
CG10365	2625.523	0.430601	0.689483	0.624527	0.532282	0.894109
CG34417	1355.209	-0.58433	0.935447	-0.62465	0.532199	0.894109
Ge-1	540.5368	0.80804	1.29434	0.624287	0.532439	0.894109
CG6310	720.2448	-0.89699	1.437502	-0.62399	0.532632	0.894144
Nbr	1617.577	0.938132	1.504924	0.623375	0.533038	0.894223
CG8701	170.3542	-1.95173	3.129411	-0.62367	0.532842	0.894223
lncRNA:CR	478.176	-0.81914	1.314323	-0.62324	0.533124	0.894223
Cyp305a1	377.2198	-1.10248	1.768653	-0.62335	0.533056	0.894223
A16	469.6975	1.217112	1.955008	0.622561	0.533573	0.894788
wls	1291.475	-1.0635	1.709013	-0.62229	0.533752	0.894902
Fibp	2006.948	-0.48274	0.776072	-0.62202	0.533926	0.895006
Su(var)3-7	383.24	-1.21916	1.963482	-0.62092	0.534654	0.896039
Hnf4	3696.465	0.378869	0.611431	0.619644	0.535492	0.896322
mdy	1986.605	-0.59904	0.966112	-0.62005	0.535223	0.896322
Mdr49	103.999	-2.15043	3.468729	-0.61995	0.535292	0.896322
cwo	1642.969	0.504064	0.812876	0.620099	0.535192	0.896322
Sarm	6040.64	-0.38051	0.613424	-0.6203	0.535061	0.896322
His2Av::GF	3912.36	-0.36494	0.588842	-0.61976	0.535416	0.896322
CG5056	1747.243	0.532935	0.860773	0.619136	0.535827	0.896647
CG42392	1894.714	0.496521	0.802123	0.619009	0.535911	0.896647
CG34125	2069.464	-0.46188	0.746608	-0.61864	0.536153	0.896858
lncRNA:CR	1466.087	0.622743	1.006895	0.618479	0.53626	0.896858

Oatp26F	73.58616	-1.97116	3.18968	-0.61798	0.536588	0.896972
CG31342	2151.856	-0.55587	0.900204	-0.6175	0.536908	0.896972
CycG	19294.45	0.252616	0.409639	0.61668	0.537446	0.896972
CG33156	2088.768	0.817181	1.323413	0.61748	0.536918	0.896972
GLaz	4085.414	-0.45939	0.743567	-0.61781	0.536699	0.896972
toy	10655.09	0.315275	0.510738	0.617293	0.537041	0.896972
Dyrk2	7613.795	0.309759	0.50212	0.616902	0.537299	0.896972
salm	606.1028	-0.72999	1.182984	-0.61708	0.537183	0.896972
CG15210	97.13189	-1.99997	3.240383	-0.6172	0.537102	0.896972
Ucp4A	1440.895	0.52629	0.853306	0.616766	0.537389	0.896972
GstT1	1150.841	0.61181	0.992674	0.616325	0.53768	0.897177
cav	247.3369	1.191902	1.93525	0.61589	0.537967	0.897289
CG8405	1261.796	-0.58472	0.949626	-0.61573	0.538071	0.897289
Cul3	3031.229	-0.40026	0.650079	-0.61572	0.538083	0.897289
lncRNA:CR	81.14632	-1.68957	2.74504	-0.6155	0.538226	0.897341
CG7920	11300.39	-0.32678	0.531078	-0.61531	0.538347	0.897357
CG5196	2227.147	-0.59312	0.965035	-0.61461	0.538812	0.897876
CG5590	1443.955	-0.5947	0.967774	-0.6145	0.538884	0.897876
asRNA:CR4	109.0826	-1.54698	2.518143	-0.61433	0.538994	0.897876
cg	8347.965	0.344204	0.561515	0.612991	0.539882	0.898297
CG12567	1896.2	-0.52852	0.862273	-0.61294	0.539918	0.898297
Cdk7	620.5896	-1.00826	1.642936	-0.6137	0.539416	0.898297
RPA3	368.6584	-1.19479	1.947558	-0.61348	0.539559	0.898297
CG11164	537.0873	-1.18928	1.938914	-0.61337	0.539629	0.898297
DCTN1-p15	624.3013	-1.08469	1.768876	-0.61321	0.539737	0.898297
Hsp60D	87.51466	2.400705	3.919125	0.612562	0.540166	0.898523
CG6287	5248.991	-0.3604	0.588902	-0.61199	0.540546	0.898596
ATPsynC	72204.06	0.238536	0.389692	0.612115	0.540462	0.898596
CG42676	261.4321	1.289891	2.107455	0.612061	0.540498	0.898596
mTerf3	394.2643	-1.18111	1.931553	-0.61148	0.540881	0.898781
Lerp	1742.221	0.802753	1.312526	0.611609	0.540796	0.898781
CG15445	120.0381	1.816726	2.974846	0.610696	0.541401	0.899458
Hpd	255.2598	1.535123	2.515051	0.610374	0.541614	0.899589
CG6163	361.3577	0.971995	1.59281	0.610239	0.541703	0.899589
Dok	254.9747	-1.51004	2.476654	-0.60971	0.542055	0.899801
JTBR	851.789	-0.65928	1.081278	-0.60972	0.542048	0.899801
CG1213	3297.23	-0.47816	0.784541	-0.60948	0.542206	0.899865
CG32195	2471.763	0.503176	0.825888	0.609255	0.542355	0.899926
CG12766	439.5347	-1.65368	2.717683	-0.60849	0.542864	0.900498
CG3529	1224.514	0.607658	0.998786	0.608397	0.542925	0.900498
phtf	2939.457	0.646599	1.063784	0.607829	0.543301	0.900565
lncRNA:CR	12723.08	0.305572	0.502615	0.607964	0.543211	0.900565
CG7272	1297.723	-0.52637	0.865846	-0.60792	0.54324	0.900565
Rpn9	2407.067	0.447701	0.736866	0.607575	0.54347	0.900658
Nep3	3637.494	0.379319	0.624553	0.607346	0.543622	0.900724
Usp12-46	850.1139	-0.97398	1.605169	-0.60678	0.544	0.901165
CG5850	817.5763	0.775959	1.27997	0.606232	0.544361	0.9012
Evi5	6897.066	0.318615	0.525764	0.606005	0.544512	0.9012
CG46387	82.17634	-1.68612	2.782848	-0.6059	0.544582	0.9012
REG	4312.574	-0.53825	0.887957	-0.60617	0.544403	0.9012

Mkp3	7052.386	0.308643	0.509339	0.605968	0.544536	0.9012
CG9436	3305.809	-0.37615	0.621369	-0.60536	0.544938	0.90123
miple2	704.0406	-1.15152	1.902157	-0.60538	0.544929	0.90123
betaTub56	19768.51	0.258852	0.427412	0.605625	0.544764	0.90123
CG11426	2587.409	0.446023	0.737037	0.605157	0.545075	0.901272
Rpb12	1979.112	-0.45883	0.758957	-0.60456	0.545472	0.901371
lncRNA:CR	117.4705	2.028295	3.354742	0.604605	0.545441	0.901371
CG12096	724.5857	-0.83894	1.387241	-0.60475	0.545342	0.901371
Actn	1372.946	0.540727	0.896746	0.602988	0.546517	0.901797
CG13116	131.1612	-2.05398	3.400879	-0.60396	0.545874	0.901797
Idgf3	6813.566	-0.39617	0.656986	-0.60301	0.546505	0.901797
CG18473	311.0374	1.712611	2.838797	0.603288	0.546317	0.901797
GatA	829.2179	0.834565	1.383831	0.603083	0.546453	0.901797
mRpL3	1614.254	-0.89244	1.479337	-0.60327	0.546329	0.901797
Zip71B	1977.605	0.794194	1.316561	0.603233	0.546353	0.901797
CG8312	1981.554	0.584388	0.969786	0.602595	0.546778	0.902022
Gip	128.0248	-1.57646	2.616763	-0.60245	0.546878	0.902022
CG31998	8035.213	0.290586	0.482839	0.601829	0.547288	0.902143
conu	8549.975	0.327457	0.543993	0.60195	0.547207	0.902143
Uba2	423.437	1.318232	2.189928	0.601952	0.547206	0.902143
Atg4b	980.6177	-0.99686	1.656943	-0.60163	0.547423	0.90218
woc	3361.35	0.37715	0.627244	0.601281	0.547653	0.90221
Myd88	726.6849	-0.68964	1.14731	-0.60109	0.547778	0.90221
fuss	565.2443	-0.82504	1.372431	-0.60116	0.547736	0.90221
Gr39a	98.24589	-1.9802	3.296699	-0.60066	0.548065	0.902497
tna	7872.569	-0.31688	0.527765	-0.60042	0.548229	0.902581
CaMKII	39790.82	0.229378	0.38232	0.599964	0.54853	0.902892
CG3164	1496.81	0.57357	0.956302	0.599779	0.548653	0.90291
RpS14b	2591.636	0.459767	0.767324	0.599183	0.549051	0.903163
kay	5672.882	0.399717	0.667269	0.599034	0.54915	0.903163
AP-2mu	2060.922	0.546469	0.912752	0.598705	0.54937	0.903163
lovit	165.5626	1.416533	2.363509	0.599335	0.54895	0.903163
Pits	8470.103	0.32245	0.538488	0.598806	0.549302	0.903163
Rab27	1840.523	0.465984	0.784167	0.59424	0.552352	0.903273
Rtca	939.0366	0.603054	1.019669	0.591421	0.554238	0.903273
CG14812	1777.794	0.485423	0.819773	0.592143	0.553755	0.903273
wds	717.7125	0.859864	1.440353	0.596982	0.55052	0.903273
dbe	344.1825	1.347067	2.264597	0.594837	0.551952	0.903273
Wdfy2	2504.102	-0.59269	1.002964	-0.59094	0.55456	0.903273
loh	63.91622	2.098363	3.540958	0.592598	0.55345	0.903273
CG9934	739.6701	0.717063	1.204411	0.595364	0.5516	0.903273
grn	104.7976	-2.09595	3.521843	-0.59513	0.551757	0.903273
foxo	4088.757	-0.36057	0.608532	-0.59253	0.553499	0.903273
NK7.1	5282.804	0.323024	0.542758	0.595153	0.551741	0.903273
CG32856	363.4132	1.018225	1.721983	0.591309	0.554313	0.903273
EndoA	12282.65	0.265167	0.448001	0.591889	0.553925	0.903273
Calx	10037.79	-0.33198	0.5614	-0.59135	0.554288	0.903273
Rlip	2996.512	0.410635	0.694115	0.591596	0.554121	0.903273
glec	4536.809	-0.38179	0.639345	-0.59716	0.550398	0.903273
Sld5	90.36049	-2.29897	3.890613	-0.5909	0.554586	0.903273

Fer1HCH	28902.41	-0.25083	0.421739	-0.59474	0.552016	0.903273
CG10081	227.0014	1.776973	2.999041	0.592514	0.553507	0.903273
RhoGAP54	534.5396	1.130795	1.911724	0.591505	0.554182	0.903273
CG8878	2738.595	-0.44533	0.753077	-0.59135	0.554286	0.903273
Buffy	176.8453	-2.29621	3.856485	-0.59541	0.551566	0.903273
CG1354	1166.523	0.547936	0.927036	0.591063	0.554478	0.903273
Cf2	2514.6	0.416002	0.703753	0.59112	0.55444	0.903273
CG4041	314.5195	-1.21725	2.035411	-0.59804	0.549816	0.903273
CG10581	957.2266	-0.6606	1.107542	-0.59645	0.550873	0.903273
Fili	1263.478	0.524038	0.879941	0.595537	0.551484	0.903273
glo	3623.601	0.426261	0.71815	0.593554	0.552811	0.903273
rdgA	9228.737	0.306661	0.519132	0.590719	0.554709	0.903273
mtRNApol	633.685	0.782589	1.322737	0.591644	0.554089	0.903273
Nup160	394.6581	-1.39712	2.359081	-0.59223	0.553695	0.903273
CG4612	11423.5	-0.3226	0.539167	-0.59833	0.549617	0.903273
uzip	5973.988	-0.34026	0.571846	-0.59501	0.551835	0.903273
Trs20	1246.422	-0.55562	0.939563	-0.59135	0.554283	0.903273
asRNA:CR4	116.6883	-1.80493	3.055619	-0.59069	0.554726	0.903273
lncRNA:CR	319.7124	-1.01075	1.691308	-0.59762	0.550096	0.903273
prage	1161.679	0.603529	1.01589	0.594089	0.552452	0.903273
sicily	1536.184	0.628712	1.060711	0.592727	0.553364	0.903273
AMPdeam	4028.257	0.364276	0.6116	0.595612	0.551435	0.903273
CG12672	184.8791	1.478356	2.498262	0.591754	0.554015	0.903273
Rip11	1335.854	-0.56864	0.95471	-0.59562	0.551429	0.903273
CG14220	541.303	1.141636	1.919497	0.594758	0.552005	0.903273
CG6933	217.7172	-1.87483	3.173518	-0.59077	0.554672	0.903273
nes	2842.409	0.58917	0.989063	0.595685	0.551386	0.903273
Grx1	2426.445	-0.43939	0.736282	-0.59677	0.550664	0.903273
CG9628	445.4332	-1.02322	1.720492	-0.59472	0.552029	0.903273
CG4629	372.3901	0.900569	1.511533	0.595798	0.55131	0.903273
Snx27	3246.237	0.390511	0.661524	0.590321	0.554975	0.903496
EMC4	4760.178	-0.39099	0.662684	-0.59001	0.555182	0.903649
rpr	195.8241	-1.72749	2.929368	-0.58971	0.555383	0.903793
Cog8	506.5673	1.249788	2.120878	0.589279	0.555674	0.904083
eIF3f1	8699.77	-0.29466	0.502055	-0.58691	0.557262	0.904299
CG3793	1505.659	-0.60991	1.03721	-0.58803	0.55651	0.904299
rdog	480.7129	0.951705	1.616442	0.588765	0.556019	0.904299
botv	192.3314	1.365757	2.327998	0.586666	0.557428	0.904299
CG6665	884.8015	0.648798	1.106418	0.586395	0.55761	0.904299
pcs	5014.924	-0.3483	0.593452	-0.58691	0.557264	0.904299
CG14044	102.3207	-1.9477	3.317799	-0.58705	0.557173	0.904299
cta	3190.55	-0.36726	0.624213	-0.58836	0.556288	0.904299
CG14464	2900.312	0.431923	0.736242	0.586659	0.557433	0.904299
dlt	391.5648	-1.17657	2.004398	-0.58699	0.557208	0.904299
Fie	16490.39	-0.24814	0.421795	-0.5883	0.556329	0.904299
Upf1	446.5532	-0.87709	1.495342	-0.58655	0.557507	0.904299
Rrp45	65.32329	2.30112	3.923516	0.586494	0.557543	0.904299
CG12991	2705.223	-0.38752	0.66079	-0.58644	0.557578	0.904299
CG1529	272.4934	1.451231	2.473931	0.586609	0.557466	0.904299
daed	369.461	-1.51183	2.570434	-0.58816	0.556425	0.904299

Smr	5582.707	-0.32627	0.556576	-0.58621	0.557734	0.904317
mRpS11	2677.533	0.394354	0.673863	0.585214	0.558404	0.904673
Son	707.5193	-0.90482	1.545997	-0.58527	0.558367	0.904673
CG8578	958.6806	0.646509	1.104074	0.585567	0.558167	0.904673
Serinc	14410.56	-0.27212	0.464855	-0.58539	0.558284	0.904673
Or1a	78.49859	-2.27746	3.952138	-0.57626	0.564438	0.904691
Rbf	599.9219	0.842449	1.475753	0.570861	0.568094	0.904691
RpL36	21958.39	0.262773	0.457598	0.574244	0.565803	0.904691
CG15912	191.8854	1.562968	2.677774	0.583682	0.559435	0.904691
CG1090	5545.897	-0.38845	0.677086	-0.57371	0.566163	0.904691
CG7214	78.85622	-2.27749	3.952133	-0.57627	0.564434	0.904691
Tep2	1889.337	-0.4941	0.857571	-0.57616	0.564506	0.904691
CG13390	328.7372	1.378119	2.393626	0.575745	0.564787	0.904691
Snx17	2123.171	-0.56517	0.983242	-0.5748	0.565426	0.904691
w-cup	82.07484	-2.27768	3.952085	-0.57632	0.564396	0.904691
Sec71	282.4074	1.159924	1.991415	0.582462	0.560255	0.904691
BuGZ	6648.11	0.302274	0.519041	0.58237	0.560318	0.904691
Ugt301D1	4652.88	-0.39719	0.69545	-0.57113	0.567911	0.904691
CG8500	4069.278	0.376402	0.659063	0.571116	0.567921	0.904691
RpL3	17941.36	0.23878	0.417897	0.571386	0.567738	0.904691
CG6723	7038.124	0.366246	0.630621	0.58077	0.561396	0.904691
KLHL18	2151.245	-0.43437	0.759232	-0.57212	0.567243	0.904691
ATPsynO	24783.12	0.257468	0.445349	0.578126	0.563179	0.904691
CG5191	1258.029	-0.51509	0.884985	-0.58203	0.560546	0.904691
AdSS	4141.445	-0.33437	0.579459	-0.57704	0.563911	0.904691
CG5745	330.8597	-0.99925	1.747691	-0.57175	0.567489	0.904691
Dph5	779.5189	0.816476	1.404438	0.581354	0.561002	0.904691
Elal	2786.607	0.417925	0.727835	0.574203	0.565831	0.904691
mino	1171.358	-0.70531	1.228605	-0.57407	0.565918	0.904691
Cisd2	7113.038	-0.35253	0.615628	-0.57263	0.566895	0.904691
Snx16	666.6792	-0.76812	1.337434	-0.57433	0.565748	0.904691
CBP	216.6169	-1.38673	2.411039	-0.57516	0.565184	0.904691
RpS14a	22010.03	0.245437	0.425453	0.576883	0.564018	0.904691
CG32706	312.9273	-1.25327	2.153862	-0.58187	0.560654	0.904691
cv-2	2825.276	-0.43236	0.754371	-0.57314	0.566548	0.904691
CG9304	580.9031	1.119801	1.924154	0.581971	0.560586	0.904691
CG30099	72.06135	-2.27702	3.952248	-0.57613	0.564525	0.904691
ckn	6458.757	-0.31277	0.537464	-0.58193	0.560613	0.904691
CG8089	140.1888	-2.27965	3.951598	-0.57689	0.564011	0.904691
bbc	9275.107	-0.28833	0.496207	-0.58108	0.561189	0.904691
Dp	1654.649	-0.79833	1.39711	-0.57141	0.567719	0.904691
CG17019	4590.098	-0.44663	0.769007	-0.58078	0.561387	0.904691
RpS11	22009.93	0.238392	0.416453	0.572434	0.567028	0.904691
san	1877.718	0.44622	0.773724	0.576716	0.564131	0.904691
CG12391	1226.876	-0.52896	0.914766	-0.57824	0.563099	0.904691
CG7220	5490.539	-0.31504	0.543187	-0.57999	0.561921	0.904691
babo	3711.938	-0.35817	0.619818	-0.57787	0.563354	0.904691
Corin	7504.089	0.327962	0.569208	0.576174	0.564498	0.904691
CG12107	1814.961	-0.48112	0.831146	-0.57886	0.562684	0.904691
LRR	3418.901	0.475303	0.823965	0.576849	0.564042	0.904691

Tsp42Eh	2327.234	-0.49115	0.85885	-0.57187	0.567408	0.904691
dpr7	14812.77	0.263617	0.461807	0.570839	0.568109	0.904691
Zyx	12132.91	0.270527	0.464074	0.582939	0.559934	0.904691
CG18635	101.923	-2.2786	3.951856	-0.57659	0.564216	0.904691
MRP	2671.098	0.393233	0.678065	0.579934	0.561959	0.904691
CROT	404.6744	-1.32288	2.271043	-0.5825	0.56023	0.904691
CG4619	918.4712	-0.70418	1.217165	-0.57854	0.562902	0.904691
CG5885	2160.533	0.565783	0.972934	0.581523	0.560888	0.904691
fbl	604.6008	-0.71861	1.251904	-0.57401	0.565959	0.904691
mt:ND3	3529.506	0.352898	0.610555	0.577995	0.563267	0.904691
side-III	2666.279	0.385735	0.663033	0.581773	0.56072	0.904691
Ufm1	1455.777	-0.50947	0.883631	-0.57657	0.56423	0.904691
CG42260	7438.627	-0.40326	0.702814	-0.57378	0.566117	0.904691
CG42673	2433.239	0.406247	0.703756	0.577255	0.563767	0.904691
CG42724	3686.109	-0.35397	0.618557	-0.57226	0.567148	0.904691
Dscam4	3343.253	0.374419	0.646741	0.578932	0.562635	0.904691
bel	3280.468	0.414111	0.72252	0.573148	0.566544	0.904691
CG3500	954.0756	-0.74421	1.294132	-0.57506	0.565248	0.904691
RpL39	9806.521	0.28823	0.500991	0.57532	0.565075	0.904691
Mtpbeta	1509.494	-0.57053	0.984681	-0.57941	0.562315	0.904691
rno	2467.033	0.462508	0.804885	0.574627	0.565544	0.904691
CG43689	1401.898	0.599763	1.04352	0.57475	0.56546	0.904691
CG43693	9018.248	-0.34009	0.585995	-0.58036	0.56167	0.904691
lncRNA:CR	233.8483	1.309663	2.286156	0.572867	0.566735	0.904691
lncRNA:CR	1083.048	-0.60913	1.054674	-0.57756	0.563563	0.904691
asRNA:CR4	107.4662	-2.2788	3.951807	-0.57665	0.564177	0.904691
lncRNA:CR	109.2543	-2.27886	3.951793	-0.57667	0.564166	0.904691
lncRNA:CR	165.759	-2.28052	3.948762	-0.57753	0.563583	0.904691
Fas1	140229.6	0.215213	0.371432	0.579413	0.562311	0.904691
cv-c	141.3167	1.427063	2.488564	0.573448	0.566341	0.904691
lncRNA:CR	111.5789	-2.27894	3.951774	-0.57669	0.564151	0.904691
lncRNA:CR	122.2139	1.674433	2.888883	0.579613	0.562176	0.904691
Or63a	1568.771	0.852212	1.468058	0.580503	0.561575	0.904691
CG9240	1539.023	0.635697	1.109691	0.572859	0.56674	0.904691
Pgant7	719.8031	1.071347	1.839176	0.582515	0.56022	0.904691
Pex23	815.3398	0.645437	1.128188	0.5721	0.567254	0.904691
Prp3	786.0846	0.650978	1.121211	0.580602	0.561509	0.904691
RasGAP1	943.1549	0.714809	1.229772	0.581254	0.561069	0.904691
CG32409	142.1987	-1.79819	3.110548	-0.57809	0.563201	0.904691
fipi	17747.07	0.280705	0.490952	0.571757	0.567487	0.904691
CG9967	97.12068	2.067236	3.586505	0.576393	0.56435	0.904691
CG13252	563.1964	-0.87339	1.530587	-0.57062	0.568257	0.904747
Syx5	1781.923	-0.49331	0.867017	-0.56897	0.569375	0.905138
GstD3	6923.459	-0.32994	0.579121	-0.56973	0.568863	0.905138
CG6073	335.9102	-1.35197	2.378651	-0.56838	0.56978	0.905138
NSD	409.7312	0.891925	1.570744	0.567836	0.570146	0.905138
Ir47a	108.2357	-2.244	3.951776	-0.56785	0.57014	0.905138
CG34250	1875.731	-0.45155	0.793871	-0.5688	0.569493	0.905138
RpS25	43808.01	0.227877	0.401246	0.567924	0.570087	0.905138
pea	333.9283	1.327566	2.336574	0.568167	0.569921	0.905138

p120ctn	10760.93	0.285619	0.502318	0.568602	0.569626	0.905138
mRpL37	1549.96	-0.49497	0.868541	-0.56989	0.568753	0.905138
CG3894	2174.926	-0.53188	0.936789	-0.56777	0.570194	0.905138
kst	851.5514	0.813463	1.429745	0.568957	0.569385	0.905138
COX6B	24257.53	0.256898	0.451419	0.56909	0.569295	0.905138
caps	2198.351	0.450583	0.792427	0.568612	0.56962	0.905138
path	1078.325	0.60866	1.071259	0.568172	0.569918	0.905138
CG14015	1770.605	-0.51959	0.915928	-0.56729	0.570518	0.905474
lncRNA:CR	96.25529	-2.23941	3.95188	-0.56667	0.570939	0.905962
dpr3	576.7516	-0.82618	1.458708	-0.56638	0.571137	0.906097
CG5282	3286.061	0.425657	0.754786	0.563943	0.572793	0.906105
CG17321	2546.801	-0.3843	0.680931	-0.56438	0.572495	0.906105
Arfip	1090.824	1.05984	1.878495	0.564196	0.57262	0.906105
Pfdn5	2017.615	-0.49726	0.878979	-0.56572	0.571583	0.906105
CG6330	4940.203	-0.32967	0.584345	-0.56416	0.572643	0.906105
CG31051	978.1189	-0.9727	1.722469	-0.56471	0.57227	0.906105
CG13751	1000.922	-0.57377	1.013874	-0.56592	0.57145	0.906105
nord	1546.547	-0.75334	1.331203	-0.56591	0.571456	0.906105
l(3)72Dr	118.9076	1.396243	2.47412	0.564339	0.572523	0.906105
lncRNA:CR	87.6723	-2.23538	3.951972	-0.56564	0.571641	0.906105
Oatp74D	1499.166	-0.56676	1.005113	-0.56388	0.572835	0.906105
Msh6	137.5707	-2.06882	3.662575	-0.56485	0.572174	0.906105
Pp4-19C	686.8741	-0.75003	1.328049	-0.56476	0.572236	0.906105
fred	1809.57	-0.45289	0.802209	-0.56456	0.572376	0.906105
ND-B14.5E	6284.807	0.309518	0.548736	0.564057	0.572715	0.906105
ppk3	234.0417	-2.00115	3.550188	-0.56368	0.572975	0.906148
RpL23	11132.57	-0.25842	0.459402	-0.56251	0.573765	0.907218
cin	397.4153	1.096578	1.951195	0.562003	0.574114	0.907491
dup	265.5584	1.311405	2.334623	0.56172	0.574307	0.907491
PHDP	188.4748	1.667361	2.967297	0.561912	0.574176	0.907491
asRNA:CR4	96.98808	-1.71325	3.050669	-0.5616	0.57439	0.907491
ND-MLRQ	19126.51	0.256878	0.458043	0.560816	0.574923	0.907976
PIP5K59B	11827.71	0.26408	0.470862	0.560843	0.574905	0.907976
UQCR-Q	22276.06	-0.2465	0.439673	-0.56064	0.575046	0.907991
mRpL44	1153.014	-0.53412	0.95434	-0.55967	0.575704	0.907994
Oamb	2611.857	0.487807	0.872094	0.559352	0.575922	0.907994
RpL29	10707.6	0.260826	0.467286	0.558172	0.576727	0.907994
Pgi	5703.427	-0.35245	0.631793	-0.55785	0.576947	0.907994
Nup153	1698.782	0.500002	0.89553	0.558331	0.576618	0.907994
CG8475	5263.98	-0.38549	0.689811	-0.55883	0.57628	0.907994
CG34351	732.9684	0.917965	1.644698	0.558136	0.576751	0.907994
cnir	1299.647	-0.51025	0.914738	-0.55781	0.576971	0.907994
CG5004	523.2354	-0.9324	1.670516	-0.55815	0.576741	0.907994
Syt14	853.7427	0.760943	1.358961	0.559945	0.575517	0.907994
Obp58b	108.1112	-2.21116	3.951753	-0.55954	0.575794	0.907994
CG13594	1540.011	-0.49236	0.880077	-0.55945	0.575855	0.907994
Cyt-b5	4304.404	-0.31815	0.568054	-0.56008	0.575428	0.907994
asRNA:CR4	72.19286	1.564787	2.803909	0.558073	0.576794	0.907994
AsnS	237.9015	-1.46685	2.61832	-0.56023	0.575325	0.907994
CG1317	3439.364	-0.3429	0.614131	-0.55835	0.576604	0.907994

msk	1924.372	0.6272	1.124355	0.557831	0.57696	0.907994
CG14903	1501.457	-0.48748	0.874788	-0.55725	0.577357	0.908423
mRpL18	1035.061	0.542622	0.975658	0.55616	0.578101	0.909238
tim	8717.485	-0.3482	0.625988	-0.55624	0.578045	0.909238
Mmp2	1649.804	-0.44377	0.799142	-0.55531	0.578683	0.909512
JhI-21	953.1731	-0.80837	1.456098	-0.55516	0.578783	0.909512
E(bx)	6475.824	0.285229	0.51347	0.555492	0.578558	0.909512
eIF1	10722.28	0.25013	0.450622	0.555077	0.578842	0.909512
Rok	1095.829	-0.53715	0.967694	-0.55508	0.57884	0.909512
RpL4	13085.34	-0.26282	0.473922	-0.55456	0.579194	0.909553
RpS3A	21747.09	0.229402	0.413788	0.554394	0.579309	0.909553
CG2852	14793.33	-0.25239	0.455338	-0.55429	0.57938	0.909553
tau	9056.827	0.263575	0.475587	0.55421	0.579435	0.909553
CG7101	352.1138	-1.02808	1.853286	-0.55473	0.579076	0.909553
Raf	3720.565	0.371187	0.671337	0.552908	0.580327	0.909719
Nlg2	5901.994	0.314092	0.568193	0.552791	0.580406	0.909719
cni	2584.783	0.383767	0.69679	0.550764	0.581795	0.909719
hook	3835.408	0.490784	0.893922	0.549023	0.58299	0.909719
Pld3	466.8465	0.893895	1.614864	0.553542	0.579892	0.909719
alpha-Est4	281.5022	-1.83985	3.346726	-0.54975	0.582493	0.909719
GCC88	432.9246	0.950168	1.716028	0.553702	0.579783	0.909719
Pde6	2991.975	-0.40993	0.745185	-0.55011	0.582243	0.909719
CG6028	807.5304	-0.94254	1.706985	-0.55217	0.580834	0.909719
CG6763	520.2641	1.540615	2.7874	0.552707	0.580464	0.909719
RIOK2	73.01506	-1.64373	2.981575	-0.55129	0.581432	0.909719
neo	189.1277	1.735849	3.163466	0.548717	0.583199	0.909719
CG3168	2174.649	-0.40319	0.733711	-0.54952	0.582646	0.909719
Caf1-180	268.5966	1.334009	2.425586	0.549974	0.582337	0.909719
ZAP3	1185.717	-0.733	1.327089	-0.55234	0.580718	0.909719
CG33199	1077.703	0.560722	1.021131	0.549118	0.582924	0.909719
CCAP-R	655.598	0.744669	1.357403	0.548598	0.583281	0.909719
rumi	101.7461	2.022817	3.658837	0.552858	0.580361	0.909719
Desat1	25254.21	-0.22576	0.408101	-0.5532	0.580123	0.909719
Tudor-SN	6729.913	-0.42389	0.768203	-0.55179	0.581091	0.909719
CG12502	1467.978	-0.49491	0.900917	-0.54934	0.582775	0.909719
asRNA:CR4	313.504	1.495169	2.715161	0.550674	0.581857	0.909719
Tim23	5415.179	-0.29787	0.53825	-0.55341	0.579985	0.909719
CG1136	201.8391	-1.83742	3.330524	-0.55169	0.581159	0.909719
Lsd-2	10566.13	-0.28759	0.521822	-0.55112	0.581548	0.909719
Obp18a	98.06753	2.042659	3.720557	0.54902	0.582992	0.909719
CG32225	8480.117	-0.26639	0.484126	-0.55026	0.582143	0.909719
CG12713	328.7317	1.525419	2.778807	0.548948	0.583041	0.909719
CG4328	172.5487	1.409921	2.565679	0.549531	0.582641	0.909719
RpL10Ab	15370.47	0.235119	0.4264	0.551404	0.581357	0.909719
CG4942	1215.611	0.977077	1.772264	0.551316	0.581417	0.909719
mrva	4798.098	0.334759	0.610163	0.548638	0.583254	0.909719
Su(dx)	5046.235	-0.33151	0.600864	-0.55172	0.581143	0.909719
asRNA:CR4	220.1415	-1.31778	2.403381	-0.5483	0.583485	0.90986
Spase25	986.6054	-0.65139	1.18896	-0.54786	0.583785	0.910152
RpIII128	190.2333	1.246778	2.280728	0.546658	0.584614	0.910844

CG33229	1128.666	0.646251	1.182404	0.546557	0.584683	0.910844
CG2186	3173.03	-0.42079	0.769485	-0.54684	0.584486	0.910844
Bmcp	494.142	-0.90134	1.649045	-0.54658	0.584665	0.910844
Als2	1050.736	0.738587	1.354624	0.545234	0.585593	0.910858
Mst85C	1471.162	-0.50253	0.922668	-0.54465	0.585992	0.910858
CG8176	4054.621	-0.33378	0.612478	-0.54497	0.585773	0.910858
AOX3	504.2757	-1.0929	2.002882	-0.54566	0.585297	0.910858
CG11857	2955.172	-0.37365	0.685435	-0.54512	0.585669	0.910858
CG15098	8885.937	0.265873	0.487248	0.545663	0.585297	0.910858
CG30116	1832.459	0.445349	0.817495	0.544773	0.58591	0.910858
CG3295	2765.208	0.380088	0.69797	0.544563	0.586054	0.910858
mRpL42	1429.923	0.470008	0.862521	0.544924	0.585806	0.910858
pan	39272.61	-0.20846	0.381611	-0.54626	0.584886	0.910858
Glg1	1402.096	0.681417	1.251269	0.544581	0.586042	0.910858
mv	1419.02	0.586517	1.076849	0.54466	0.585987	0.910858
DIP-alpha	245.0775	1.23545	2.270158	0.544213	0.586295	0.910879
beta-Spec	6011.778	-0.32005	0.588001	-0.5443	0.586235	0.910879
BHD	360.8405	1.136453	2.090425	0.543647	0.586684	0.911308
Nsun2	241.9519	-1.07742	1.984971	-0.54279	0.587274	0.911375
CG9391	1712.524	-0.43359	0.798697	-0.54287	0.587222	0.911375
TTLL6B	155.2959	-1.97222	3.634791	-0.54259	0.587409	0.911375
Aldh-III	6007.822	-0.37417	0.68882	-0.5432	0.586991	0.911375
CG33111	420.8793	1.361579	2.507626	0.542975	0.587147	0.911375
Hsc70-4	20559.47	-0.22616	0.416803	-0.5426	0.587406	0.911375
CG8336	557.7202	1.083342	1.997597	0.542323	0.587596	0.91149
Shmt	413.4624	1.308797	2.420789	0.540649	0.58875	0.911517
PNKP	474.9776	0.981361	1.814141	0.540951	0.588542	0.911517
CG8202	1403.347	0.946673	1.748559	0.541402	0.588231	0.911517
EMC2A	1401.548	0.483311	0.893613	0.54085	0.588611	0.911517
CG13603	7337.102	0.278764	0.514985	0.541306	0.588297	0.911517
atl	1519.318	0.469752	0.868326	0.540986	0.588518	0.911517
Cul4	1369.037	0.522989	0.965913	0.541445	0.588201	0.911517
mEFTu2	453.7255	-0.74036	1.369304	-0.54069	0.588723	0.911517
Fer3HCH	65.70623	1.904294	3.516672	0.541505	0.58816	0.911517
Sfxn2	5340.546	0.32137	0.594203	0.540842	0.588616	0.911517
kz	219.5307	-1.39611	2.58389	-0.54031	0.588982	0.911526
HDAC6	6939.817	-0.31654	0.585712	-0.54044	0.588895	0.911526
SPARC	10148.19	-0.32134	0.594999	-0.54006	0.589155	0.911543
robl	5408.141	-0.29895	0.553643	-0.53997	0.58922	0.911543
Cyp1	24445.97	-0.2346	0.434852	-0.53949	0.589547	0.911872
CG4646	1373.639	0.478431	0.887356	0.539165	0.589773	0.912046
Tsp42Eg	146.6801	1.549415	2.877363	0.538484	0.590243	0.91207
ncm	1240.341	-0.50699	0.941077	-0.53873	0.590073	0.91207
CG42238	1591.992	-0.43874	0.814091	-0.53894	0.58993	0.91207
mRpL39	1464.947	-0.75681	1.405351	-0.53852	0.59022	0.91207
CG1907	2751.198	-0.42648	0.792376	-0.53823	0.59042	0.912168
Spt3	480.0332	1.092098	2.029964	0.537989	0.590585	0.912246
G9a	2358.262	-0.37994	0.714621	-0.53167	0.594958	0.912265
Ugalt	263.6819	-0.9599	1.793892	-0.53509	0.592586	0.912265
ICA69	2173.356	-0.67673	1.273046	-0.53159	0.595012	0.912265

ND-51	11846.07	-0.25812	0.48567	-0.53148	0.595087	0.912265
cdc14	1120.015	-0.50259	0.937487	-0.5361	0.591888	0.912265
mon2	1652.183	-0.42074	0.791392	-0.53165	0.594969	0.912265
mRpS7	1296.464	-0.48766	0.919834	-0.53016	0.595998	0.912265
Mal-B1	147.74	-1.95811	3.653127	-0.53601	0.591953	0.912265
CG4631	792.6926	-0.58273	1.091205	-0.53402	0.593328	0.912265
Nedd8	2405.387	-0.36663	0.6867	-0.5339	0.593413	0.912265
TfIIFalpha	254.8155	-1.25959	2.352212	-0.53549	0.592309	0.912265
CG4511	838.585	-0.77618	1.461889	-0.53094	0.595459	0.912265
CG17187	433.8071	-0.98523	1.858476	-0.53013	0.596024	0.912265
Hsp70Bb	76.42786	-1.49978	2.794266	-0.53673	0.591452	0.912265
Ace	29394.31	0.227682	0.425706	0.534834	0.592765	0.912265
Trap1	2827.553	-0.42254	0.78987	-0.53495	0.592685	0.912265
CG9945	1656.906	-0.75948	1.425033	-0.53295	0.594065	0.912265
GstE6	5402.919	-0.37967	0.716087	-0.5302	0.595973	0.912265
CG5174	12014.42	0.256428	0.477421	0.537111	0.591191	0.912265
HPS4	1605.634	0.694669	1.305694	0.532031	0.594705	0.912265
sca	359.6923	1.074884	2.00695	0.535581	0.592248	0.912265
Roc2	1731.204	0.456844	0.854173	0.534838	0.592762	0.912265
CG13743	7029.286	0.313643	0.588407	0.533038	0.594007	0.912265
TBPH	3250.64	-0.41839	0.788479	-0.53062	0.59568	0.912265
Tm1	9670.058	-0.26468	0.498644	-0.53081	0.595552	0.912265
udt	342.3257	-1.25507	2.342284	-0.53583	0.592076	0.912265
B52	10388.76	-0.3796	0.713546	-0.53199	0.594733	0.912265
CG41099	5452.455	-0.31737	0.594558	-0.53379	0.593487	0.912265
CG34132	1116.831	-0.68261	1.287363	-0.53024	0.595948	0.912265
CG40045	8657.394	0.260761	0.490685	0.531423	0.595126	0.912265
NKAIN	14650.92	0.265992	0.498243	0.533859	0.593439	0.912265
cbs	1462.098	0.797695	1.505452	0.529871	0.596201	0.912265
CG42235	9694.976	0.318412	0.601052	0.529758	0.59628	0.912265
SdhA	17981.63	0.244339	0.455086	0.536908	0.591331	0.912265
Pdk1	6008.304	-0.31154	0.58456	-0.53295	0.594066	0.912265
CG9205	1840.298	-0.4078	0.767261	-0.5315	0.595071	0.912265
mRpL45	2216.939	0.379244	0.711832	0.532772	0.594192	0.912265
ocm	601.6342	0.79287	1.48855	0.532646	0.594279	0.912265
asRNA:CR4	680.4666	-0.62763	1.176682	-0.53339	0.593766	0.912265
CG32278	5151.602	0.292271	0.545486	0.5358	0.592097	0.912265
Fit1	2266.845	-0.44873	0.839034	-0.53482	0.592773	0.912265
Set2	1344.442	-0.46097	0.86963	-0.53007	0.596061	0.912265
ND-18	5139.417	-0.28681	0.541043	-0.5301	0.596041	0.912265
CG5589	317.8726	1.050335	1.978506	0.530873	0.595507	0.912265
sina	3161.442	-0.33687	0.633039	-0.53215	0.594621	0.912265
Nrx-IV	3510.566	0.429056	0.801368	0.535404	0.59237	0.912265
Taf2	287.7418	1.436506	2.709316	0.53021	0.595966	0.912265
RpS17	8054.606	0.295456	0.55096	0.536258	0.59178	0.912265
Cpsf6	1252.458	-0.51031	0.951455	-0.53634	0.591722	0.912265
fliI	176.35	1.330642	2.503058	0.531607	0.594999	0.912265
RpA-70	1830.914	0.433364	0.81928	0.528957	0.596836	0.912351
dpr14	3318.634	-0.35235	0.66617	-0.52892	0.596859	0.912351
CG31324	4504.729	-0.29582	0.558978	-0.52922	0.59665	0.912351

Mid1	5637.93	-0.29843	0.564283	-0.52886	0.596904	0.912351
CG3632	2417.178	0.491153	0.928009	0.529255	0.596629	0.912351
CanB2	4569.07	0.349798	0.66205	0.528356	0.597252	0.912474
lncRNA:CR	731.6116	-0.62054	1.174714	-0.52825	0.597326	0.912474
CG32204	590.513	0.667008	1.262489	0.528328	0.597272	0.912474
asRNA:CR4	2932.118	0.345643	0.654537	0.528073	0.597448	0.912487
DOR	7345.861	-0.27944	0.529653	-0.52759	0.597786	0.91283
Sym	493.6207	-0.9948	1.889666	-0.52644	0.598582	0.913761
CG9330	2300.206	0.402138	0.763968	0.526381	0.598624	0.913761
CCT5	959.7605	-0.70022	1.331836	-0.52575	0.59906	0.914253
Sema1a	17994.51	-0.21945	0.417903	-0.52513	0.599493	0.914739
nrv3	84112.69	0.210773	0.402006	0.524303	0.600068	0.915095
ci	1514.654	0.562129	1.071571	0.524584	0.599872	0.915095
CG3700	552.6148	-0.97854	1.866126	-0.52437	0.600023	0.915095
RpL27A	18432.33	0.234434	0.447368	0.524029	0.600258	0.915211
gzl	4843.716	-0.28755	0.549622	-0.52317	0.600854	0.915826
Scm	911.0006	0.845115	1.616098	0.522935	0.601019	0.915826
CG6454	2138.414	0.426704	0.815956	0.52295	0.601009	0.915826
Impbeta11	573.1419	0.905767	1.732551	0.522794	0.601118	0.915826
Eip63E	10597.98	0.262892	0.503158	0.522483	0.601334	0.915981
tRNA:Thr-/	252.8357	-1.80747	3.464942	-0.52164	0.601918	0.916201
eIF2Bgamr	482.3166	0.914808	1.754334	0.521456	0.602049	0.916201
CG17803	223.2137	1.5135	2.899021	0.522073	0.60162	0.916201
CG6044	11884.89	-0.242	0.464051	-0.5215	0.60202	0.916201
rho-4	156.7972	1.181444	2.265092	0.521588	0.601958	0.916201
CG5521	2157.247	0.400411	0.768468	0.521051	0.602331	0.916351
Mon1	455.491	1.196584	2.296764	0.520987	0.602376	0.916351
nis	92.96133	-1.99631	3.836251	-0.52038	0.602798	0.916819
beta-Man	1093.984	0.929677	1.787099	0.520216	0.602913	0.916821
Rpb10	1245.485	-0.50845	0.979117	-0.51929	0.603558	0.91728
Psi	2333.802	0.36855	0.709476	0.519468	0.603435	0.91728
fidipidine	632.622	0.669145	1.288509	0.519317	0.60354	0.91728
SoxN	515.0276	0.861925	1.660652	0.519028	0.603741	0.917385
CG14024	2106.726	-0.48735	0.940524	-0.51817	0.604342	0.918049
CG13999	2983.529	-0.41621	0.803659	-0.51789	0.604535	0.918049
Usp14	1995.183	0.403765	0.780346	0.517418	0.604865	0.918049
clt	1242.197	0.493889	0.954096	0.517651	0.604702	0.918049
CG8765	1410.146	-0.78853	1.523594	-0.51755	0.604775	0.918049
Pten	2553.55	-0.37084	0.716513	-0.51757	0.604761	0.918049
SP2353	3436.481	0.479241	0.927473	0.516717	0.605353	0.91827
Cyp6g1	75.86562	1.982047	3.834743	0.516866	0.60525	0.91827
CSN1b	2350.092	-0.45016	0.870936	-0.51687	0.605243	0.91827
Cnot4	3416.287	0.359414	0.696968	0.515682	0.606076	0.918472
Ald1	23532.39	-0.21361	0.414268	-0.51562	0.60612	0.918472
Fer2LCH	41244.83	-0.1996	0.386823	-0.51601	0.605846	0.918472
CG34293	640.0554	0.612967	1.188331	0.515821	0.605979	0.918472
Shab	15722.82	0.25892	0.501747	0.516037	0.605829	0.918472
mRpL48	1866.727	-0.42685	0.827968	-0.51554	0.606173	0.918472
Cyp4p3	222.5968	-1.27753	2.484086	-0.51429	0.607052	0.918909
Dgk	1589.243	-0.44976	0.875878	-0.51349	0.607607	0.918909

CG42376	2137.544	0.378096	0.735763	0.513883	0.607334	0.918909
GLS	1843.482	-0.65818	1.280766	-0.5139	0.607323	0.918909
Su(z)2	1482.001	-0.48634	0.945439	-0.51441	0.606965	0.918909
Gfat1	159.4373	1.79138	3.487103	0.513716	0.607451	0.918909
GlyRS	1549.614	-0.41971	0.815086	-0.51493	0.6066	0.918909
Sox21a	159.9533	1.300568	2.532236	0.513604	0.607529	0.918909
CG18178	142.3691	-1.65774	3.222965	-0.51435	0.607005	0.918909
bip1	1581.575	-0.70626	1.374357	-0.51389	0.607331	0.918909
bnl	400.4757	0.727465	1.417918	0.513052	0.607915	0.918982
Ect3	978.3422	0.790534	1.540546	0.513152	0.607845	0.918982
Tet	16434.95	-0.22147	0.432041	-0.51261	0.608227	0.918982
MAN1	891.9826	-0.62343	1.215692	-0.51282	0.608076	0.918982
CR43972	236.3432	1.206871	2.353858	0.51272	0.608147	0.918982
Vap33	9014.788	-0.25794	0.503425	-0.51238	0.608387	0.919051
Arl2	367.7509	1.128063	2.207291	0.511062	0.609307	0.919092
Elk	1410.185	0.790818	1.547501	0.511029	0.609331	0.919092
CG6999	494.8212	-0.70617	1.378975	-0.5121	0.608582	0.919092
Ost48	3686.306	0.356908	0.697427	0.511749	0.608826	0.919092
Mvl	2634.86	-0.34031	0.665514	-0.51135	0.609105	0.919092
h	1251.816	-0.56342	1.102492	-0.51104	0.609321	0.919092
CG14989	19622.81	-0.30276	0.591992	-0.51143	0.609053	0.919092
CG12194	3220.397	0.446323	0.873267	0.511096	0.609284	0.919092
robo2	667.7428	-0.9029	1.76768	-0.51078	0.609504	0.91918
Arf102F	7821.955	-0.25742	0.504293	-0.51045	0.609738	0.919361
Pka-C1	15836.22	0.229521	0.450097	0.509936	0.610096	0.919555
CG2811	952.2469	-0.50269	0.985597	-0.51004	0.610025	0.919555
Rbbp5	470.6698	0.976184	1.922829	0.507681	0.611677	0.919931
Pect	7121.028	0.353741	0.697275	0.50732	0.611931	0.919931
beta'COP	1475.349	0.427454	0.843162	0.506965	0.612179	0.919931
CG18428	813.1736	0.600475	1.17989	0.508924	0.610805	0.919931
CHKov1	667.7485	-0.59394	1.166684	-0.50909	0.610692	0.919931
CG4409	22676.22	-0.22752	0.448286	-0.50754	0.611778	0.919931
mael	110.1501	1.564121	3.075386	0.508593	0.611037	0.919931
hoe1	10216.2	0.245536	0.483337	0.508001	0.611452	0.919931
CkIIbeta	9831.611	0.238809	0.47104	0.506983	0.612167	0.919931
CG42390	1191.73	-0.4825	0.949927	-0.50793	0.6115	0.919931
CG42542	5298.268	0.309758	0.610919	0.507036	0.61213	0.919931
SmE	1458.28	-0.4607	0.906503	-0.50822	0.611301	0.919931
COX8	28840.4	0.212604	0.418231	0.508342	0.611214	0.919931
Pfdn2	2278.564	0.373611	0.734828	0.508433	0.61115	0.919931
CG13679	73.07235	1.995885	3.925989	0.508378	0.611189	0.919931
Neos	421.3694	1.116105	2.197766	0.507836	0.611568	0.919931
CG42654	151.6401	1.639093	3.235376	0.506616	0.612424	0.919955
ZnT63C	2581.313	-0.46305	0.913889	-0.50668	0.61238	0.919955
Sirt4	83.29235	1.887941	3.729326	0.506242	0.612687	0.920177
Ccs	634.5602	-1.01578	2.007939	-0.50588	0.612941	0.920213
CG42324	4671.213	0.353219	0.698077	0.505988	0.612865	0.920213
CG7231	2361.182	-0.36584	0.723461	-0.50568	0.613081	0.920252
NPFR	169.6602	1.231996	2.437514	0.505431	0.613256	0.920343
CG31219	1864.681	-0.45742	0.907378	-0.50411	0.614185	0.920653

CG3618	3222.131	-0.32557	0.645628	-0.50427	0.614074	0.920653
Ykt6	3893.736	0.310052	0.614759	0.504347	0.614018	0.920653
cnc	2900.154	-0.38022	0.754284	-0.50408	0.614207	0.920653
ppk29	188.8585	1.296179	2.571199	0.504114	0.614181	0.920653
lncRNA:CR	242.7056	1.025797	2.035064	0.504061	0.614218	0.920653
CG46338	798.7413	0.906471	1.798575	0.503994	0.614266	0.920653
mthl13	574.3111	0.901106	1.788733	0.503768	0.614424	0.920719
Trim9	435.4104	0.689373	1.369222	0.503478	0.614628	0.920853
Npc2a	8166.046	-0.2479	0.492638	-0.5032	0.614824	0.920973
CG11638	642.3112	0.701698	1.396505	0.502467	0.615339	0.921212
Lip4	195.805	1.664534	3.313432	0.50236	0.615415	0.921212
Oda	31555.69	-0.20543	0.408633	-0.50272	0.615158	0.921212
CNPYb	1440.876	0.429884	0.855797	0.50232	0.615442	0.921212
CG7378	2989.44	-0.43444	0.86518	-0.50214	0.615567	0.921227
CG1513	7426.458	0.27276	0.543554	0.501808	0.615802	0.921236
Tob	18921.51	0.226463	0.4512	0.501912	0.61573	0.921236
CG14688	4869.55	-0.30022	0.598523	-0.5016	0.615947	0.92128
Naa30A	626.225	0.718354	1.435386	0.50046	0.616751	0.92134
Orco	77.33376	-1.8652	3.727386	-0.5004	0.616791	0.92134
Sirt2	1501.382	0.725363	1.447988	0.500946	0.616409	0.92134
Chchd2	10724.61	0.227906	0.454745	0.501173	0.616249	0.92134
CG13895	1525.277	0.454383	0.907961	0.500443	0.616763	0.92134
mRpS5	3215.154	0.315945	0.630584	0.501036	0.616346	0.92134
Csas	7232.866	-0.30871	0.616384	-0.50084	0.616482	0.92134
Rpl135	599.5745	0.846736	1.696301	0.499166	0.617663	0.921352
gcm2	130.3332	-1.92555	3.867608	-0.49787	0.618579	0.921352
CG10338	1613.65	0.438964	0.88059	0.498488	0.61814	0.921352
Taf13	1437.548	0.43251	0.865986	0.499443	0.617468	0.921352
m-cup	1143.903	0.682277	1.370678	0.497766	0.618649	0.921352
CG6005	1578.464	0.414607	0.832916	0.497778	0.618641	0.921352
CG2224	1078.659	0.675487	1.357429	0.497622	0.61875	0.921352
Pfk	6729.45	0.312116	0.625114	0.499295	0.617572	0.921352
CR33317	150.5114	-1.53503	3.07595	-0.49904	0.617749	0.921352
Gr64f	206.8007	-1.52147	3.052249	-0.49847	0.61815	0.921352
GC1	689.2692	-0.57431	1.153308	-0.49797	0.618505	0.921352
Rbp1	11246.44	0.276089	0.552756	0.499477	0.617443	0.921352
wdp	5295.93	0.291034	0.581998	0.50006	0.617033	0.921352
asRNA:CR4	387.3628	-0.95236	1.910451	-0.4985	0.618132	0.921352
CG14073	2807.646	-0.35491	0.709884	-0.49996	0.617105	0.921352
CG6650	1098.446	-0.50902	1.021731	-0.49819	0.618351	0.921352
Syt1	62824.56	0.201237	0.402712	0.499705	0.617283	0.921352
dpr5	1051.062	-0.58228	1.171214	-0.49716	0.619076	0.921362
EMRE	6973.578	0.331773	0.667944	0.496708	0.619395	0.921362
mRNA-cap	568.1252	-0.72986	1.467396	-0.49739	0.618917	0.921362
CG10417	4215.549	-0.3234	0.650998	-0.49677	0.619348	0.921362
CG5835	2419.747	0.370439	0.745662	0.496792	0.619335	0.921362
CG14232	641.1547	-0.88329	1.778552	-0.49664	0.619445	0.921362
14-3-3epsi	28066.74	0.222594	0.44864	0.496154	0.619786	0.921532
CG32645	72.69529	-1.93413	3.898283	-0.49615	0.619789	0.921532
Non3	669.6833	-0.86249	1.740978	-0.49541	0.620314	0.921883

LSm3	623.8639	-0.90607	1.829812	-0.49517	0.620478	0.921883
OtopLc	5290.493	-0.2649	0.534978	-0.49516	0.620485	0.921883
Lpin	9237.329	0.232489	0.46925	0.495449	0.620283	0.921883
Phs	172.6206	-1.34788	2.725061	-0.49462	0.620866	0.922278
Sec16	2105.898	-0.40498	0.819668	-0.49408	0.621248	0.922676
lncRNA:CR	459.2512	0.782069	1.583598	0.493856	0.621408	0.922742
Tk	1352.538	-0.63898	1.294324	-0.49367	0.621536	0.922761
Fas2	1751.171	-0.49134	0.996115	-0.49326	0.621832	0.922846
mid	82.93274	-1.89088	3.834633	-0.49311	0.621938	0.922846
VhaM9.7-a	14472.29	0.214391	0.434627	0.493275	0.621818	0.922846
mRpL16	2269.749	-0.34546	0.705021	-0.49	0.624131	0.923139
lin-52	1630.936	-0.41763	0.857269	-0.48716	0.626145	0.923139
Cyp4ac2	161.8445	1.271577	2.588423	0.491256	0.623246	0.923139
CG11070	1256.216	-0.74337	1.512682	-0.49143	0.623126	0.923139
CG31760	1066.002	-0.52145	1.059734	-0.49206	0.622678	0.923139
CG9265	2294.128	-0.36614	0.747495	-0.48982	0.624259	0.923139
CG8526	318.4884	-0.93943	1.922434	-0.48867	0.625078	0.923139
CG11626	77.66168	-1.92136	3.95141	-0.48625	0.626793	0.923139
Rh3	103.1527	-1.92179	3.951285	-0.48637	0.626703	0.923139
e	5501.324	-0.31938	0.654102	-0.48826	0.625362	0.923139
awd	7909.452	0.247145	0.50686	0.487599	0.625834	0.923139
CG6675	72.11442	-1.92122	3.951448	-0.48621	0.626821	0.923139
CG30431	131.1059	-1.73554	3.558884	-0.48766	0.625789	0.923139
CG4866	531.4741	0.862406	1.75806	0.490544	0.623749	0.923139
l(1)G0320	4522.108	0.300039	0.614654	0.488144	0.625448	0.923139
CARPB	805.4343	0.69056	1.404443	0.491697	0.622934	0.923139
Hmg-2	830.6368	0.801506	1.646771	0.486714	0.626461	0.923139
CG5267	150.3054	1.198684	2.460432	0.487184	0.626128	0.923139
Vha36-1	10188.81	-0.23241	0.476372	-0.48788	0.625633	0.923139
AspRS	122.0002	1.680853	3.448944	0.487353	0.626008	0.923139
CG11635	143.2164	1.050252	2.141511	0.490426	0.623833	0.923139
nec	1618.555	0.592037	1.205599	0.491073	0.623375	0.923139
eIF5	218.0954	0.87628	1.792907	0.488748	0.62502	0.923139
beat-Va	1811.016	-0.40602	0.832899	-0.48748	0.625916	0.923139
Naa80	3942.808	-0.31858	0.655288	-0.48616	0.626851	0.923139
CG33777	69.07663	-1.92112	3.951472	-0.48618	0.62684	0.923139
sev	1192.991	-0.61547	1.256056	-0.49	0.624135	0.923139
CG17163	1633.287	0.435245	0.890143	0.488961	0.624869	0.923139
nub	148.3085	-1.45181	2.968685	-0.48904	0.624811	0.923139
tef	127.7191	-1.92205	3.951212	-0.48645	0.62665	0.923139
l(2)37Cb	818.6759	0.743358	1.524556	0.48759	0.62584	0.923139
whip	561.4844	-0.61017	1.240268	-0.49197	0.622742	0.923139
RhoGAP10	2971.761	-0.31783	0.64802	-0.49046	0.623809	0.923139
robo1	3407.561	-0.29853	0.612901	-0.48708	0.626198	0.923139
SNF4Agam	3436.041	-0.31334	0.640493	-0.48922	0.624686	0.923139
CG45050	11287.69	-0.21837	0.446151	-0.48946	0.624515	0.923139
CG7638	1776.237	0.41351	0.84427	0.489784	0.624287	0.923139
pall	642.935	-0.66349	1.353502	-0.4902	0.62399	0.923139
Cyt-c1	11645.74	0.286663	0.58762	0.487837	0.625665	0.923139
CG3165	320.9898	1.110054	2.27687	0.487535	0.625879	0.923139

Clp	526.1658	-1.06454	2.161274	-0.49255	0.622329	0.923139
CG15914	84.28416	-1.89288	3.89715	-0.48571	0.627174	0.923445
sqh	5496.382	0.25902	0.534457	0.484642	0.627931	0.923542
CG5466	2550.215	-0.37483	0.773138	-0.48481	0.627809	0.923542
CG18731	516.8489	0.687024	1.41615	0.485135	0.62758	0.923542
mRpS28	930.3984	-0.54231	1.118774	-0.48473	0.627866	0.923542
nito	428.7514	-0.75892	1.564459	-0.4851	0.627606	0.923542
trx	9559.306	0.256878	0.529312	0.485305	0.62746	0.923542
CanB	23366.9	0.212272	0.440677	0.481694	0.630023	0.924005
atms	1556.84	0.40299	0.839355	0.480119	0.631143	0.924005
Uhg2	547.6206	0.646485	1.345625	0.480435	0.630918	0.924005
ScsbetaA	12010.11	-0.23561	0.487241	-0.48355	0.628704	0.924005
CG7059	148.7494	1.228164	2.553816	0.480913	0.630578	0.924005
Ppox	193.4762	1.055946	2.188092	0.482587	0.629389	0.924005
Usp1	2173.861	-0.47873	0.99738	-0.47998	0.631239	0.924005
CG2059	1423.072	0.632255	1.310816	0.482337	0.629567	0.924005
CG13500	104.5781	-1.90887	3.951277	-0.4831	0.629022	0.924005
Mhcl	872.3046	0.510279	1.057152	0.482692	0.629314	0.924005
TM4SF	35723.44	0.203936	0.423077	0.482031	0.629784	0.924005
CG3566	2280.927	-0.34194	0.712241	-0.4801	0.631159	0.924005
CG34219	411.0125	0.949667	1.967721	0.482623	0.629364	0.924005
Rab26	2577.23	0.361144	0.750192	0.481402	0.630231	0.924005
kuz	4731.64	-0.28753	0.597591	-0.48114	0.630416	0.924005
nsl1	2820.483	-0.33885	0.704392	-0.48105	0.630479	0.924005
CG30274	110.2574	-1.9096	3.951258	-0.48329	0.62889	0.924005
spg	4540.375	0.277236	0.57731	0.480219	0.631071	0.924005
dop	4725.037	-0.35307	0.732949	-0.48172	0.630007	0.924005
Vha13	34086.37	-0.1957	0.405752	-0.48233	0.629575	0.924005
CG33233	119.7133	1.720991	3.571616	0.481852	0.629911	0.924005
CG11151	3825.763	-0.28972	0.602968	-0.4805	0.630875	0.924005
RpL26	12016.09	-0.22718	0.47067	-0.48268	0.62932	0.924005
CG32112	1329.172	-0.49622	1.027123	-0.48312	0.629012	0.924005
Grip163	1387.669	0.455234	0.945439	0.481505	0.630158	0.924005
CG5906	79.35124	-1.90442	3.951393	-0.48196	0.629834	0.924005
Oli	2256.167	0.342281	0.713594	0.479658	0.63147	0.924017
Yeti	8342.639	0.291226	0.607164	0.479649	0.631477	0.924017
CG8929	4680.877	0.30326	0.633105	0.479005	0.631935	0.924274
CG33017	140.4446	1.769451	3.694698	0.478916	0.631998	0.924274
GramD1B	4850.862	0.316057	0.659684	0.479103	0.631865	0.924274
pns	2379.008	-0.35186	0.735174	-0.47861	0.632214	0.924319
Pka-C3	325.7479	1.001141	2.092033	0.478549	0.632259	0.924319
bcn92	2221.956	-0.3556	0.744148	-0.47787	0.632745	0.924692
fzr	4653.165	-0.28665	0.599769	-0.47793	0.632697	0.924692
CG31650	2991.767	0.36467	0.764444	0.47704	0.633334	0.92483
CG13108	1361.396	0.433173	0.908077	0.477022	0.633347	0.92483
mt:ND2	7564.282	0.243231	0.509999	0.476925	0.633415	0.92483
mr	627.1974	0.718215	1.505836	0.476954	0.633395	0.92483
Dlic	5150.066	0.327267	0.685626	0.477326	0.63313	0.92483
Pp2C1	1099.115	-0.48364	1.015613	-0.47621	0.633925	0.924967
Ufc1	1492.581	-0.42813	0.898721	-0.47637	0.633808	0.924967

lncRNA:CR	3216.893	0.334914	0.703031	0.476385	0.6338	0.924967
ine	6554.791	0.401626	0.843492	0.476147	0.63397	0.924967
DCTN4-p6:	1798.349	0.378639	0.796429	0.47542	0.634487	0.925385
parvin	921.1112	-0.62708	1.318906	-0.47546	0.634462	0.925385
G6P	970.5973	-0.73964	1.557025	-0.47504	0.634761	0.925617
CrebB	544.2077	0.759697	1.599934	0.47483	0.634908	0.925663
NaCP60E	39181.12	0.200337	0.422164	0.474548	0.63511	0.925788
Ank	16090.06	-0.23888	0.503953	-0.47401	0.635493	0.926178
SdhB	1586.664	-0.39475	0.833958	-0.47335	0.635965	0.92653
Ubqn	2779.438	-0.32164	0.679334	-0.47346	0.635882	0.92653
CG11050	3873.947	0.293907	0.625818	0.469636	0.638615	0.926691
CG31817	331.4724	0.891122	1.895658	0.470086	0.638294	0.926691
EloA	474.7691	0.611346	1.301144	0.469853	0.63846	0.926691
eEF2	22201.76	-0.20573	0.436309	-0.47153	0.637262	0.926691
Ntan1	2765.994	-0.33562	0.710525	-0.47236	0.636669	0.926691
spidey	2665.009	-0.41937	0.888888	-0.47179	0.637077	0.926691
Syn2	6236.944	0.261319	0.556244	0.469793	0.638503	0.926691
CG18327	107.6761	-1.62682	3.442717	-0.47254	0.636542	0.926691
Tapdelta	2280.004	0.342189	0.726805	0.470813	0.637774	0.926691
PCB	1013.042	-0.55239	1.172126	-0.47127	0.637446	0.926691
cathD	14858.61	-0.20384	0.433365	-0.47036	0.638096	0.926691
CG13739	3455.006	0.290047	0.616055	0.470813	0.637774	0.926691
alpha-Catr	634.1409	0.706149	1.497179	0.471653	0.637175	0.926691
Stlk	5299.436	-0.25917	0.551468	-0.46997	0.638379	0.926691
Tango9	4912.437	-0.30065	0.638726	-0.4707	0.637855	0.926691
SmD2	1417.152	-0.45649	0.967445	-0.47185	0.637035	0.926691
CkIIalpha	3305.731	-0.34842	0.737166	-0.47265	0.636465	0.926691
CG45049	7782.183	0.297855	0.633582	0.470113	0.638274	0.926691
CG1927	4823.392	0.2856	0.604355	0.47257	0.63652	0.926691
CG1703	412.9353	0.702377	1.492974	0.470455	0.63803	0.926691
CG14194	1099.118	0.523783	1.114925	0.469792	0.638504	0.926691
CG17361	175.728	-1.27504	2.697314	-0.47271	0.636423	0.926691
RapGAP1	10368.7	0.242379	0.516507	0.469266	0.63888	0.926907
Trp1	5885.767	-0.28717	0.613957	-0.46774	0.639972	0.927872
Paip2	4117.558	0.285635	0.610737	0.467689	0.640007	0.927872
Axs	80.83992	1.559901	3.332141	0.468138	0.639686	0.927872
CG14515	271.0331	1.416367	3.026699	0.467957	0.639815	0.927872
CG3078	3816.066	0.284551	0.608897	0.467322	0.64027	0.928085
CG3071	686.037	-0.73444	1.597081	-0.45986	0.645614	0.928162
Ide	4320.899	0.278559	0.604645	0.460698	0.645015	0.928162
Hrb27C	22554.46	0.194423	0.41958	0.463376	0.643095	0.928162
CG4168	681.6987	0.547945	1.173658	0.466869	0.640593	0.928162
bur	1001.101	-0.50296	1.084537	-0.46376	0.642822	0.928162
Timp	4031.538	-0.32331	0.702956	-0.45993	0.645566	0.928162
Cyp12e1	3716.574	-0.29997	0.65235	-0.45982	0.645642	0.928162
Hrb87F	891.0674	0.533246	1.150348	0.463552	0.642969	0.928162
tara	1780.466	0.408579	0.88508	0.46163	0.644347	0.928162
CG17931	261.7688	-0.85355	1.85324	-0.46057	0.645105	0.928162
aralar1	3112.809	0.392207	0.851999	0.460338	0.645274	0.928162
CG1636	1538.467	0.388261	0.83908	0.462722	0.643564	0.928162

tud	5160.209	-0.26558	0.576131	-0.46098	0.644814	0.928162
Gbp1	323.2196	-1.11452	2.392396	-0.46586	0.641317	0.928162
Sec61beta	3820.087	-0.27766	0.597323	-0.46483	0.642051	0.928162
RpS23	8640.39	-0.22799	0.489441	-0.46582	0.641346	0.928162
CG10375	172.295	-0.96023	2.074262	-0.46292	0.643418	0.928162
Rbp9	13007.11	0.205389	0.443048	0.46358	0.642948	0.928162
spi	5226.833	-0.29144	0.626165	-0.46544	0.641616	0.928162
AlaRS	207.2952	1.061324	2.304292	0.460586	0.645096	0.928162
CG34159	2460.09	0.377165	0.811213	0.46494	0.641974	0.928162
Zdhhc8	2558.851	-0.36974	0.793214	-0.46612	0.641127	0.928162
5PtaseI	3399.765	-0.2874	0.624183	-0.46045	0.645194	0.928162
scra	96.59797	-1.71366	3.675469	-0.46624	0.641042	0.928162
nemy	8503.29	0.292725	0.634223	0.461549	0.644405	0.928162
YME1L	908.8513	-0.53539	1.147848	-0.46643	0.640909	0.928162
mRpL17	2600.612	0.367265	0.79449	0.462266	0.643891	0.928162
CG43850	296.6668	0.9389	2.012926	0.466435	0.640904	0.928162
Glut1	21177.14	0.220202	0.475244	0.463345	0.643117	0.928162
Rgk1	1346.442	-0.5443	1.173876	-0.46368	0.642878	0.928162
Spn42Da	107.6656	1.132955	2.461792	0.460215	0.645362	0.928162
lncRNA:CR	2267.489	-0.3859	0.837622	-0.46071	0.645005	0.928162
CG45263	13539.64	0.23391	0.505286	0.462926	0.643418	0.928162
lncRNA:CR	910.6268	-0.46294	0.99846	-0.46366	0.642892	0.928162
CG12099	2122.497	0.383469	0.82471	0.464974	0.64195	0.928162
Pgant6	494.4196	0.659359	1.429709	0.461184	0.644667	0.928162
sbr	4449.043	-0.28217	0.606383	-0.46534	0.64169	0.928162
Gapvd1	498.5556	0.620925	1.342092	0.462655	0.643612	0.928162
eag	7880.206	0.249315	0.537515	0.463829	0.64277	0.928162
CG6659	1443.909	0.438647	0.943705	0.464814	0.642065	0.928162
Papss	1058.243	0.69596	1.508697	0.461299	0.644584	0.928162
rogdi	4367.726	0.269077	0.584033	0.460723	0.644998	0.928162
Ubc4	1315.133	0.405413	0.872576	0.464616	0.642206	0.928162
Gug	6486.696	-0.24021	0.516071	-0.46547	0.641597	0.928162
ple	426.0157	1.019522	2.21276	0.460747	0.64498	0.928162
CG10031	114.2697	-1.58831	3.442413	-0.46139	0.644517	0.928162
CG34117	1556.783	-0.37356	0.812721	-0.45964	0.645775	0.928186
CG14646	521.7197	0.854468	1.861535	0.459013	0.646225	0.9285
CG30428	3610.189	-0.27887	0.607403	-0.45912	0.646145	0.9285
GstE12	11109.89	0.230857	0.503273	0.458711	0.646442	0.928645
CG6656	318.4971	0.94641	2.065196	0.458267	0.646761	0.928882
gammaCO	1924.383	0.454145	0.991239	0.45816	0.646838	0.928882
CG9630	325.6449	1.110136	2.423953	0.457986	0.646963	0.928895
Trx-2	5201.594	-0.27629	0.603974	-0.45746	0.64734	0.929105
Grd	122.1479	1.754264	3.833806	0.457578	0.647256	0.929105
Atf3	1758.495	-0.39079	0.878647	-0.44477	0.656488	0.929164
Ocrl	1579.531	0.442109	0.998121	0.442941	0.657808	0.929164
CG14818	2074.595	-0.32926	0.725976	-0.45355	0.650156	0.929164
dnc	15997.57	-0.18386	0.424575	-0.43305	0.664978	0.929164
CG15765	12486.3	0.228454	0.511499	0.446635	0.655139	0.929164
drongo	6342.233	-0.25165	0.575108	-0.43757	0.661696	0.929164
CG3625	6445.753	0.247283	0.558996	0.44237	0.658222	0.929164

kin17	907.5863	0.713475	1.643593	0.434095	0.66422	0.929164
mRpL15	962.3753	-0.51215	1.184659	-0.43232	0.66551	0.929164
CG33285	141.9085	-1.70748	3.951177	-0.43214	0.665637	0.929164
CG33288	65.34555	-1.70647	3.951495	-0.43186	0.665846	0.929164
Wnk	4966.39	-0.26098	0.580961	-0.44922	0.653271	0.929164
Pc	1189.626	-0.50978	1.148516	-0.44386	0.657147	0.929164
CG7458	127.947	-1.70729	3.896013	-0.43821	0.661231	0.929164
Vha14-2	85.43514	1.752295	3.951516	0.443449	0.657441	0.929164
jagn	3088.761	-0.32937	0.724975	-0.45432	0.649597	0.929164
CG14020	102.9148	-1.7071	3.95128	-0.43204	0.665715	0.929164
CG11034	62.99801	1.71957	3.951712	0.435145	0.663457	0.929164
CG13784	3023.407	0.301048	0.672185	0.447865	0.65425	0.929164
jp	353.427	0.648236	1.493921	0.433916	0.664349	0.929164
Sur	168.0823	-1.70761	3.951135	-0.43218	0.665609	0.929164
CG6729	112.7038	1.307996	2.97228	0.440065	0.65989	0.929164
CG5781	116.2053	1.643603	3.691421	0.445249	0.656139	0.929164
Tap42	262.4048	1.136417	2.556116	0.444587	0.656618	0.929164
Ski6	83.4307	1.725329	3.834725	0.449922	0.652766	0.929164
CG7099	425.0317	-0.71933	1.575966	-0.45644	0.648076	0.929164
CG16863	463.7308	-0.88455	2.028283	-0.43611	0.662757	0.929164
Adat1	138.4789	-1.13956	2.496948	-0.45638	0.648115	0.929164
CG15278	66.41387	-1.7065	3.951485	-0.43186	0.66584	0.929164
kek3	2642.616	0.397824	0.874704	0.454809	0.649246	0.929164
CG4455	104.2811	1.752743	3.951406	0.443575	0.65735	0.929164
crp	3543.866	-0.28134	0.632683	-0.44468	0.656551	0.929164
yellow-b	79.57195	1.752116	3.951561	0.443398	0.657478	0.929164
Rsph9	83.44584	1.752239	3.951531	0.443433	0.657453	0.929164
Acp36DE	112.0709	1.717092	3.951359	0.434557	0.663884	0.929164
MESR3	3068.762	0.301352	0.6688	0.450586	0.652288	0.929164
Grip71	102.0824	1.752699	3.951417	0.443562	0.657359	0.929164
CG33120	83.68504	-1.70688	3.951366	-0.43197	0.665762	0.929164
CG10495	992.2899	0.633552	1.457382	0.434719	0.663766	0.929164
l(2)37Cc	1645.427	-0.4019	0.901704	-0.44572	0.655802	0.929164
pigeon	1392.257	-0.44104	1.011772	-0.43591	0.662902	0.929164
tj	95.76322	-1.44857	3.273871	-0.44246	0.658154	0.929164
COX4	37582.9	0.182029	0.412038	0.441777	0.658651	0.929164
EMC1	2267.511	0.333108	0.732244	0.454914	0.649171	0.929164
CG8369	39486.29	-0.18356	0.421912	-0.43508	0.663505	0.929164
CG14687	785.4553	0.771637	1.778601	0.433845	0.664401	0.929164
TfIIFbeta	196.6896	1.001615	2.225867	0.449989	0.652718	0.929164
CG7488	468.087	0.831451	1.829183	0.454548	0.649435	0.929164
eff	9272.916	-0.24186	0.560528	-0.43148	0.666121	0.929164
SerRS-m	609.4298	0.83673	1.853919	0.451331	0.651751	0.929164
CG4546	93.39234	1.752462	3.951464	0.443497	0.657406	0.929164
fru	13339.04	-0.19311	0.43929	-0.43961	0.660222	0.929164
ninaE	169.5248	1.628943	3.77684	0.431298	0.666252	0.929164
Trm7-34	997.1145	-0.51113	1.168992	-0.43724	0.661938	0.929164
CASK	12314.89	-0.19276	0.438991	-0.43911	0.660584	0.929164
CG13850	1233.18	-0.42769	0.954465	-0.44809	0.654086	0.929164
Ndc1	112.3117	1.734817	3.951364	0.439043	0.660631	0.929164

Slimp	476.5417	0.987977	2.171277	0.455021	0.649094	0.929164
CG10560	153.0913	1.584577	3.654554	0.43359	0.664586	0.929164
wdn	409.1124	0.820948	1.800716	0.455901	0.648461	0.929164
eEF1gamm	2392.952	-0.35194	0.79975	-0.44006	0.659893	0.929164
Cnx99A	3159.406	-0.29795	0.683929	-0.43564	0.663097	0.929164
qless	883.0963	0.546442	1.243614	0.439398	0.660373	0.929164
Cul2	2788.827	0.346875	0.785834	0.44141	0.658916	0.929164
qsm	64.91396	1.751512	3.951709	0.443229	0.6576	0.929164
Hsl	1690.184	-0.39199	0.873978	-0.44851	0.653784	0.929164
CG11041	78.52495	1.752079	3.95157	0.443388	0.657485	0.929164
hrg	3257.691	0.279798	0.632297	0.442511	0.65812	0.929164
stau	6759.425	0.241848	0.546924	0.442196	0.658347	0.929164
Trf4-1	869.3449	-0.45624	1.032224	-0.442	0.658491	0.929164
fend	4591.009	-0.30049	0.681515	-0.44091	0.659277	0.929164
CG32683	2319.75	-0.38671	0.868448	-0.44529	0.656109	0.929164
BTBD9	2029.342	-0.55619	1.252702	-0.44399	0.657049	0.929164
stx	2091.233	-0.34824	0.770461	-0.45199	0.651279	0.929164
CG10433	30636.99	-0.24077	0.547782	-0.43954	0.660273	0.929164
Oatp58Dc	5936.459	-0.31722	0.694196	-0.45697	0.647696	0.929164
CG12964	70.14895	1.751719	3.951649	0.443288	0.657557	0.929164
unc-5	184.199	-0.97684	2.185317	-0.447	0.654873	0.929164
hui	135.6911	1.753227	3.951291	0.44371	0.657252	0.929164
drk	2193.403	0.353549	0.812447	0.435166	0.663442	0.929164
CG7637	140.2225	-0.97494	2.141626	-0.45523	0.648943	0.929164
CG30345	74.515	1.724863	3.951591	0.436498	0.662475	0.929164
Shaw	4007.552	-0.29597	0.686214	-0.43131	0.666243	0.929164
ptc	201.2041	1.02995	2.374363	0.433779	0.664449	0.929164
CG5033	205.1171	1.023296	2.366028	0.432495	0.665382	0.929164
CR18217	223.778	1.275031	2.893856	0.440599	0.659503	0.929164
REPTOR-BI	4159.217	-0.26364	0.607161	-0.43423	0.664125	0.929164
CG31437	65.22805	1.751528	3.951705	0.443233	0.657597	0.929164
mav	5907.484	-0.27981	0.628641	-0.4451	0.656249	0.929164
IA-2	118769.4	-0.16898	0.380801	-0.44376	0.657219	0.929164
tral	4098.652	0.271317	0.59735	0.454201	0.649684	0.929164
mxt	2542.345	-0.32384	0.744944	-0.43472	0.663763	0.929164
snoRNA:Ps	555.6862	-0.55043	1.226378	-0.44883	0.653557	0.929164
CG34200	1669.834	0.355978	0.815244	0.436652	0.662364	0.929164
CG34288	77.80928	-1.70677	3.951401	-0.43194	0.665785	0.929164
dpr12	6995.61	-0.24078	0.531253	-0.45323	0.650384	0.929164
CG40472	1753.152	0.361209	0.824817	0.437926	0.66144	0.929164
stv	574.1865	0.579536	1.275557	0.45434	0.649584	0.929164
CG14644	153.719	1.585716	3.64935	0.43452	0.663911	0.929164
Octbeta3R	2249.158	0.339128	0.784741	0.432153	0.66563	0.929164
CG42391	88.31443	-1.70694	3.951342	-0.43199	0.665748	0.929164
Sep-04	5395.002	0.251766	0.578725	0.435036	0.663537	0.929164
Thor	1289.546	-0.44109	1.0066	-0.4382	0.661242	0.929164
LanB1	134.2066	1.393224	3.147052	0.442708	0.657977	0.929164
5-HT2B	2036.257	-0.38627	0.890366	-0.43384	0.664407	0.929164
Acsl	11364.53	0.21676	0.48942	0.442892	0.657844	0.929164
mRpS29	1063.834	0.490017	1.099551	0.445652	0.655849	0.929164

Liprin-gam	13551.06	-0.2124	0.482699	-0.44004	0.659911	0.929164
CG2765	3031.908	-0.37146	0.855404	-0.43425	0.66411	0.929164
ttm2	93.67721	-1.70503	3.951264	-0.43151	0.666095	0.929164
asRNA:CR4	85.64363	-1.70691	3.951355	-0.43198	0.665755	0.929164
asRNA:CR4	772.1605	-0.90433	2.061142	-0.43875	0.66084	0.929164
Piezo	3046.521	0.303208	0.675489	0.448873	0.653524	0.929164
lncRNA:CR	92.5877	-1.70698	3.951322	-0.432	0.66574	0.929164
CG44325	5324.313	0.256162	0.585062	0.437838	0.661504	0.929164
asRNA:CR4	82.04823	1.168564	2.678744	0.436236	0.662666	0.929164
lncRNA:CR	689.487	0.534473	1.209994	0.441715	0.658695	0.929164
Exo84	560.0825	0.608663	1.362983	0.446567	0.655188	0.929164
Ntl	95.39294	-1.13323	2.56606	-0.44162	0.658762	0.929164
lncRNA:CR	348310.5	-0.2176	0.489562	-0.44447	0.656703	0.929164
Der-1	3035.608	0.348368	0.786095	0.443163	0.657648	0.929164
asRNA:CR4	125.7056	-1.70736	3.951212	-0.43211	0.66566	0.929164
hpRNA:CR4	99.36275	-1.14017	2.59304	-0.4397	0.660153	0.929164
ND-ACP	11848.55	-0.19304	0.442854	-0.43591	0.662902	0.929164
Pex10	501.1399	1.07164	2.374176	0.451373	0.651721	0.929164
CG12104	761.2875	-0.56992	1.27153	-0.44821	0.654	0.929164
CG5707	440.6934	1.074656	2.463004	0.436319	0.662605	0.929164
Larp4B	8275.751	0.219924	0.5006	0.439321	0.660429	0.929164
promL	5319.566	-0.24919	0.547371	-0.45525	0.648928	0.929164
Ack	535.2185	-0.69021	1.59464	-0.43283	0.665136	0.929164
CG15209	3217.599	-0.29457	0.655736	-0.44921	0.653277	0.929164
Lint-1	565.899	-0.66834	1.508492	-0.44305	0.657728	0.929164
Cpr11B	183.6676	-1.11242	2.503099	-0.44442	0.656742	0.929164
Tim9a	630.5754	0.524445	1.188195	0.441379	0.658938	0.929164
Rbp1-like	8190.782	0.218441	0.487064	0.448485	0.653803	0.929164
Rab3-GEF	3364.079	-0.29773	0.673152	-0.4423	0.658274	0.929164
CG8509	90.77484	1.75243	3.95148	0.443487	0.657414	0.929164
scu	3088.116	-0.358	0.815701	-0.43888	0.660746	0.929164
HisRS	1052.047	-0.51019	1.124325	-0.45377	0.649993	0.929164
CG7058	2215.937	-0.60729	1.394426	-0.43552	0.663188	0.929164
Sec61gam	3987.76	-0.25484	0.589243	-0.43249	0.665384	0.929164
CG6951	109.0973	1.752839	3.951384	0.443601	0.657331	0.929164
trc	1517.876	-0.43478	0.964954	-0.45057	0.652302	0.929164
fal	380.5835	-0.90911	2.078162	-0.43746	0.661779	0.929164
CG7484	1914.386	-0.3348	0.757314	-0.44209	0.658423	0.929164
Pop5	385.0351	1.008271	2.225591	0.453035	0.650523	0.929164
CG32163	89.70932	1.117758	2.556334	0.43725	0.66193	0.929164
Pdh	84.56619	1.728334	3.951511	0.437386	0.661832	0.929164
CG7768	230.724	1.367271	3.068457	0.445589	0.655894	0.929164
FoxK	1698.402	0.366539	0.84507	0.433738	0.664479	0.929164
Irbp18	577.9174	0.661769	1.460162	0.453216	0.650393	0.929164
CG14174	100.244	-1.7071	3.95129	-0.43204	0.665715	0.929164
CG3434	627.2927	0.653929	1.434235	0.455943	0.648431	0.929164
foi	1321.339	0.541621	1.21585	0.445467	0.655982	0.929164
qm	1174.538	-0.41825	0.92859	-0.45042	0.65241	0.929164
tow	2835.999	-0.33088	0.746208	-0.44342	0.657464	0.929164
mst	119.0438	1.753005	3.951344	0.443648	0.657297	0.929164

Obp19d	154.7465	1.753424	3.951243	0.443765	0.657212	0.929164
Ugt36A1	128.1984	-1.70738	3.951206	-0.43212	0.665656	0.929164
Sbat	85.80567	1.606748	3.521026	0.456329	0.648153	0.929164
CG3104	1691.831	0.453282	1.017457	0.445504	0.655955	0.929164
Taf10b	4420.168	0.257255	0.562864	0.457047	0.647638	0.929164
CG4238	3292.569	0.290244	0.670734	0.432726	0.665214	0.929164
CkIIalpha-i	239.3288	-0.90771	2.105781	-0.43106	0.666427	0.929246
CG2991	5092.015	0.280496	0.651501	0.430538	0.666804	0.929288
CG32549	6834.228	0.221996	0.515617	0.430544	0.6668	0.929288
Arr2	509.3729	1.139934	2.646463	0.430739	0.666658	0.929288
amon	7859.766	0.220679	0.513132	0.430063	0.66715	0.929447
rols	3380.868	-0.35697	0.829841	-0.43017	0.667072	0.929447
Sos	1187.492	0.603275	1.403414	0.429863	0.667296	0.929489
Coq7	3271.934	-0.26884	0.625669	-0.42968	0.667431	0.929516
otu	92.3363	-1.6945	3.95132	-0.42884	0.668037	0.930037
CG15043	233.0389	1.360157	3.171177	0.428912	0.667987	0.930037
Hacd2	2140.744	-0.43171	1.008455	-0.42809	0.668583	0.930153
CG6908	1264.486	0.475434	1.109423	0.428542	0.668257	0.930153
Dbp80	8647.395	0.212614	0.496483	0.42824	0.668476	0.930153
CG32219	129.9427	-1.34201	3.134608	-0.42813	0.668558	0.930153
CG13339	330.7861	0.68255	1.59616	0.42762	0.668928	0.930309
Ptip	940.4413	-0.70342	1.644929	-0.42763	0.668919	0.930309
CG10337	111.8488	-1.6865	3.951246	-0.42683	0.669504	0.930929
Uxs	379.5396	-0.94654	2.218347	-0.42669	0.669606	0.930929
rush	1053.481	0.485343	1.138036	0.426474	0.669762	0.930986
hyd	6850.462	0.219603	0.51542	0.426066	0.670059	0.931238
Bdp1	186.0027	0.9676	2.276806	0.424981	0.67085	0.93165
p38a	351.4893	0.987893	2.325203	0.424863	0.670937	0.93165
mRpL54	1562.996	0.372042	0.875206	0.42509	0.670771	0.93165
CG32055	303.4651	-1.12974	2.656758	-0.42523	0.670668	0.93165
cert	1016.453	0.423375	0.996139	0.425016	0.670825	0.93165
Pli	6350.466	0.229483	0.540789	0.424349	0.671311	0.932009
Rif1	73.56735	-1.67547	3.951419	-0.42402	0.671553	0.932067
dati	3114.412	0.290967	0.686286	0.423973	0.671585	0.932067
CG13029	71.0746	-1.67435	3.951436	-0.42373	0.67176	0.932149
CG11456	854.7099	-0.78379	1.851445	-0.42334	0.672048	0.932387
dap	331.7935	0.682165	1.613439	0.422802	0.67244	0.932551
CG11752	5486.186	0.245447	0.580471	0.422842	0.672411	0.932551
Frq2	1846.886	0.354549	0.838774	0.422699	0.672515	0.932551
Fis1	9893.7	0.198683	0.470507	0.422274	0.672825	0.932659
Zip99C	399.5928	0.642548	1.521237	0.422385	0.672744	0.932659
lncRNA:CR	122.5177	1.399076	3.316505	0.421853	0.673133	0.932716
cue	473.8812	0.648678	1.537064	0.422024	0.673008	0.932716
CG10809	1127.801	0.395066	0.936754	0.42174	0.673215	0.932716
CG13364	2122.65	-0.30306	0.719557	-0.42117	0.67363	0.932807
CG10341	620.9189	0.858323	2.037761	0.421209	0.673603	0.932807
CG12818	281.0869	-0.96889	2.299814	-0.42129	0.673543	0.932807
CG6481	168.7823	1.169811	2.779504	0.42087	0.67385	0.932951
CG34353	6175.759	-0.24327	0.578732	-0.42034	0.674236	0.933268
CG6282	7191.796	-0.21898	0.521083	-0.42024	0.674311	0.933268

RpL14	31210.57	0.16821	0.400536	0.419961	0.674514	0.933387
Rpt4	1334.861	-0.40007	0.953116	-0.41975	0.674667	0.933439
Grip75	526.4989	-0.74889	1.786649	-0.41916	0.675099	0.933693
CG31183	887.3026	-0.49694	1.185365	-0.41923	0.675048	0.933693
CG13014	504.5531	0.757152	1.80695	0.419022	0.6752	0.933693
CG1648	4350.14	0.250655	0.598767	0.418618	0.675495	0.933941
ubl	369.0138	-0.62859	1.503463	-0.41809	0.675879	0.934311
CG4042	1676.905	0.415519	0.995295	0.417483	0.676325	0.934449
COX7A	26580.9	0.184846	0.442769	0.417478	0.676329	0.934449
S6KL	1147.359	0.410245	0.982571	0.417522	0.676296	0.934449
Rox8	5160.209	0.301491	0.723036	0.416979	0.676694	0.934471
CG31038	1868.781	-0.59094	1.416733	-0.41712	0.676593	0.934471
eEF1alpha	32671.46	0.19597	0.469897	0.417048	0.676643	0.934471
Ccdc58	351.5019	0.866231	2.080751	0.416307	0.677185	0.934904
CG7564	2752.449	-0.34183	0.821241	-0.41623	0.67724	0.934904
Vsp37A	2568.846	0.280928	0.675729	0.41574	0.6776	0.93524
CG3987	584.8711	-0.59423	1.431012	-0.41525	0.677959	0.935554
Cyt-c-p	45577.01	0.16302	0.392864	0.414952	0.678177	0.935554
Toll-6	2167.774	-0.38705	0.932525	-0.41506	0.678101	0.935554
RhoGAP10	13026.6	0.180396	0.435018	0.414685	0.678372	0.935662
CG6293	732.6815	0.916233	2.218637	0.412971	0.679628	0.935913
SdhD	12263.39	0.183557	0.443938	0.413475	0.679259	0.935913
vap	2313.035	0.30698	0.742037	0.413699	0.679095	0.935913
amn	398.6986	-0.5972	1.445406	-0.41317	0.679479	0.935913
lncRNA:CR	731.6745	0.663891	1.606727	0.413195	0.679464	0.935913
lncRNA:CR	78.36821	1.633373	3.951512	0.413354	0.679347	0.935913
InR	5169.912	-0.26009	0.628872	-0.41358	0.679181	0.935913
CG18171	261.3338	-0.70998	1.719721	-0.41284	0.679721	0.935913
IntS10	277.6125	0.695659	1.682789	0.413397	0.679316	0.935913
CG4880	193.2392	1.566277	3.793529	0.412881	0.679694	0.935913
Or45a	1658.784	0.499648	1.210798	0.41266	0.679855	0.935938
nAChRalph	4713.927	-0.24058	0.583718	-0.41214	0.680233	0.936298
Alp12	760.4206	-0.45232	1.098981	-0.41158	0.680649	0.936398
TfIIA-S	4692.273	0.232991	0.566107	0.411568	0.680656	0.936398
CG42813	4026.488	-0.24199	0.587695	-0.41176	0.680512	0.936398
haf	690.7306	0.563781	1.370774	0.411287	0.680862	0.936521
Rpn3	2312.419	0.351826	0.857879	0.410111	0.681725	0.937386
Abl	9586.177	-0.19505	0.475524	-0.41019	0.681669	0.937386
UbcE2H	1581.798	0.375596	0.917567	0.409339	0.682291	0.938004
ksr	690.466	-0.48953	1.198393	-0.40849	0.682913	0.938538
Drep1	398.3133	0.758452	1.856564	0.408525	0.682888	0.938538
Coprox	166.8609	1.191808	2.920397	0.408098	0.683202	0.93865
stumps	6314.443	-0.24802	0.607809	-0.40806	0.683229	0.93865
Charon	708.3948	0.781091	1.915448	0.407785	0.683432	0.938768
Cda5	1822.57	0.469352	1.151493	0.407603	0.683565	0.938791
Plc21C	7707.777	0.260326	0.640691	0.406321	0.684507	0.938981
Sdhaf3	806.3719	-0.43225	1.064249	-0.40616	0.684629	0.938981
tou	4694.162	0.251874	0.619645	0.406482	0.684389	0.938981
Tpst	1236.559	-0.41789	1.028414	-0.40634	0.684489	0.938981
CG42541	16922.28	0.189013	0.465191	0.406313	0.684512	0.938981

stai	7936.671	0.195355	0.481004	0.406141	0.684639	0.938981
eIF2beta	2379.383	-0.31284	0.769092	-0.40677	0.684176	0.938981
lin-28	91.97369	1.219555	2.996874	0.406942	0.68405	0.938981
Tsp42Ee	17300.75	-0.23356	0.57559	-0.40578	0.684903	0.939182
CNBP	5850.435	0.243832	0.601198	0.405576	0.685054	0.939229
Rtf1	721.4631	0.504843	1.245961	0.405184	0.685343	0.939464
Hacd1	2276.792	0.34047	0.841385	0.404655	0.685731	0.939676
CG4970	130.2469	-1.29501	3.199582	-0.40474	0.685666	0.939676
CG10189	270.3544	1.222276	3.03032	0.403349	0.686692	0.940349
CG7741	1021.863	0.461053	1.141875	0.403769	0.686383	0.940349
jvl	6972.355	-0.29665	0.735203	-0.4035	0.686583	0.940349
CG15203	970.736	-0.43415	1.076335	-0.40335	0.686687	0.940349
Pex12	364.3707	-0.88432	2.194752	-0.40292	0.687005	0.940617
SNRPG	1890.517	-0.30294	0.752955	-0.40234	0.687436	0.941047
Obp99c	89.0742	-1.54042	3.835564	-0.40161	0.687968	0.941614
Gld2	75.48303	1.218434	3.035159	0.40144	0.688096	0.94163
AspRS-m	633.3553	0.748173	1.865617	0.401033	0.688396	0.94188
CG12730	7557.102	-0.2162	0.542043	-0.39887	0.689991	0.941908
PLCXD	5366.895	-0.34161	0.857655	-0.3983	0.690406	0.941908
PICK1	1645.176	-0.53789	1.349644	-0.39854	0.690232	0.941908
CG31808	12878.2	0.184258	0.461665	0.399115	0.689808	0.941908
GCC185	486.2588	-0.71869	1.803357	-0.39853	0.69024	0.941908
ninaB	12003.26	-0.25821	0.645908	-0.39977	0.689327	0.941908
Rad60	569.5295	-0.73248	1.828507	-0.40059	0.688723	0.941908
Rab4	5249.76	-0.23382	0.585367	-0.39944	0.689572	0.941908
Setd3	413.0298	0.807179	2.029011	0.397819	0.690764	0.941908
Echs1	1309.645	-0.48955	1.223472	-0.40014	0.689057	0.941908
CG32206	6769.305	0.200089	0.502401	0.398266	0.690434	0.941908
Chd1	853.6195	0.449314	1.12577	0.399117	0.689807	0.941908
CG43295	72.21207	1.557136	3.913623	0.397876	0.690722	0.941908
nahoda	253.7888	-0.68712	1.723308	-0.39872	0.690099	0.941908
CG46385	21152.99	-0.17729	0.445205	-0.39823	0.690461	0.941908
Pcyt2	7345.781	-0.20639	0.518479	-0.39807	0.69058	0.941908
Tomosyn	5123.572	0.247062	0.619952	0.398517	0.690249	0.941908
Grasp65	1737.689	0.389133	0.973455	0.399745	0.689345	0.941908
CG6683	328.5606	0.624764	1.561934	0.399994	0.689161	0.941908
CG4669	205.767	1.317115	3.300194	0.399102	0.689818	0.941908
Oatp33Ea	1567.141	0.393497	0.990195	0.397394	0.691077	0.941917
CG8778	1418.412	0.49044	1.234368	0.397321	0.691131	0.941917
GlcAT-P	20431.41	-0.18112	0.455663	-0.39749	0.691003	0.941917
Lrp4	7751.45	-0.20327	0.511786	-0.39717	0.69124	0.941917
HSPBAP1	594.0103	0.73472	1.850981	0.396936	0.691415	0.941994
mRpS25	2146.432	-0.36029	0.90803	-0.39678	0.691531	0.941994
CG11964	563.0051	0.643654	1.627092	0.395585	0.692411	0.942873
Pisd	1404.396	0.358176	0.905293	0.395647	0.692365	0.942873
Rab11	5419.341	-0.22923	0.580122	-0.39515	0.692733	0.943103
shrb	2229.817	-0.35553	0.900003	-0.39504	0.692815	0.943103
OtopLa	10048.16	0.212811	0.538933	0.394875	0.692935	0.943106
Itpr	2514.264	-0.27304	0.693181	-0.39389	0.69366	0.943932
crm	1258.487	0.524395	1.347058	0.389289	0.697062	0.944152

snf	3439.647	-0.25094	0.64277	-0.3904	0.696241	0.944152
Spat	131.0929	1.538476	3.951182	0.389371	0.697002	0.944152
CG11449	131.4068	1.538478	3.951181	0.389372	0.697001	0.944152
CG31643	328.5189	0.912018	2.319673	0.393166	0.694197	0.944152
Wee1	3378.898	0.286232	0.732079	0.390985	0.695808	0.944152
beat-IIIc	1953.726	-0.2972	0.764528	-0.38874	0.697468	0.944152
CG31793	1442.846	0.436536	1.121455	0.389258	0.697085	0.944152
Crz	71.24844	1.537616	3.951413	0.389131	0.69718	0.944152
trem	753.3716	-0.82849	2.130257	-0.38892	0.697338	0.944152
tsl	122.0953	1.5384	3.951202	0.38935	0.697017	0.944152
Fadd	602.1836	-0.66264	1.684722	-0.39332	0.69408	0.944152
Nepl13	137.7888	1.538521	3.951168	0.389384	0.696992	0.944152
CG13614	88.6159	1.537977	3.951314	0.389232	0.697105	0.944152
Jasper	932.6639	0.619134	1.592076	0.388885	0.697361	0.944152
CG16739	73.7594	1.537661	3.951396	0.389144	0.69717	0.944152
CG9294	62.66933	1.537362	3.951482	0.389059	0.697232	0.944152
Ubi-p5E	6870.265	0.199424	0.510915	0.390327	0.696295	0.944152
5-HT2A	333.47	-0.85499	2.17584	-0.39295	0.694359	0.944152
CG42668	5364.161	0.23248	0.596627	0.389657	0.69679	0.944152
SmD1	632.9754	0.519616	1.329817	0.390743	0.695987	0.944152
CG43125	75.22412	1.537715	3.951386	0.389158	0.697159	0.944152
lncRNA:CR	120.8407	0.974811	2.479538	0.393142	0.694214	0.944152
CG43245	75.1195	1.537712	3.951387	0.389158	0.69716	0.944152
CG30416	110.3775	1.538279	3.951234	0.389316	0.697042	0.944152
Brca2	176.3172	0.93732	2.390524	0.392098	0.694986	0.944152
lncRNA:CR	68.10974	1.537531	3.951436	0.389107	0.697197	0.944152
Cpr64Ab	81.18765	1.537846	3.951351	0.389195	0.697132	0.944152
CG12607	84.84946	1.537916	3.951332	0.389215	0.697117	0.944152
ArfGAP1	1188.953	-0.57459	1.467468	-0.39155	0.695392	0.944152
CG3347	84.95409	1.537918	3.951331	0.389215	0.697117	0.944152
BORCS5	1155.874	0.440991	1.135493	0.388369	0.697743	0.944365
wapl	2680.651	-0.25892	0.667484	-0.3879	0.698089	0.944423
Pdha	6457.076	-0.22165	0.572231	-0.38734	0.698504	0.944423
Pex16	622.2818	-0.60149	1.552858	-0.38734	0.698501	0.944423
Hex-C	65.1737	1.509168	3.897239	0.38724	0.698578	0.944423
Spn43Ab	13928.57	-0.17201	0.443782	-0.38759	0.698317	0.944423
Atg6	963.4562	-0.40874	1.055632	-0.3872	0.69861	0.944423
CG10576	1748.521	0.416346	1.072692	0.388132	0.697918	0.944423
CG6191	140.3805	0.842456	2.17748	0.386895	0.698834	0.944489
Ca-alpha1T	15146.71	0.201527	0.520992	0.386814	0.698894	0.944489
Map205	1317.627	-0.46201	1.195182	-0.38656	0.699081	0.944496
Hexo2	2749.563	0.317077	0.820402	0.38649	0.699134	0.944496
Rab3	37684.72	0.158515	0.410861	0.385812	0.699636	0.944576
CG2063	353.1432	-0.72288	1.874646	-0.38561	0.699786	0.944576
lncRNA:CR	87.59712	1.523059	3.951314	0.385456	0.699899	0.944576
disco-r	245.2561	-0.78569	2.037416	-0.38563	0.699768	0.944576
lncRNA:CR	91.9913	1.523858	3.951295	0.38566	0.699748	0.944576
RhoGAP19	5302.179	-0.22112	0.572992	-0.3859	0.699569	0.944576
Tbp	415.3431	0.855978	2.222257	0.385184	0.700101	0.944689
Rad23	5713.38	0.216494	0.562379	0.384962	0.700266	0.944753

Rheb	4839.388	-0.2224	0.578359	-0.38453	0.700583	0.944863
snoRNA:M	239.2719	0.802634	2.086714	0.38464	0.700504	0.944863
CG12560	358.272	0.842269	2.197787	0.383235	0.701545	0.944891
CG9986	841.6395	-0.58213	1.516364	-0.3839	0.701056	0.944891
CG14441	1702.055	0.315546	0.823332	0.383255	0.701531	0.944891
Mst77F	664.0503	-0.63684	1.66102	-0.3834	0.70142	0.944891
alphaSnap	14435.69	0.168705	0.439315	0.384018	0.700965	0.944891
CG12851	2858.006	-0.30038	0.782072	-0.38408	0.700918	0.944891
lncRNA:CR	215.9131	0.727163	1.896229	0.383478	0.701365	0.944891
Ten-a	19024.37	0.178556	0.465844	0.383295	0.701501	0.944891
CG15930	149.793	0.888766	2.323046	0.382586	0.702026	0.944904
mRpL10	1039.145	-0.39717	1.037931	-0.38266	0.701973	0.944904
Dlip3	2090.515	-0.33846	0.884445	-0.38268	0.701959	0.944904
rad	5256.551	0.234961	0.613777	0.382812	0.701859	0.944904
Lim1	1477.308	-0.3251	0.850724	-0.38215	0.702353	0.945064
CG6610	110.4254	-1.17995	3.087989	-0.38211	0.702381	0.945064
CRIF	1448.77	0.318477	0.839374	0.379422	0.704374	0.94524
CG11983	71.40318	1.137857	2.992371	0.380253	0.703758	0.94524
wkd	3211.496	0.296516	0.779334	0.380474	0.703594	0.94524
Hr96	963.9877	-0.57487	1.514472	-0.37959	0.704252	0.94524
CG1416	2305.115	0.295962	0.777757	0.380533	0.70355	0.94524
Panx	471.8407	0.73844	1.946181	0.37943	0.704368	0.94524
CSN4	971.7118	-0.41262	1.085129	-0.38025	0.703759	0.94524
Gpb5	1213.113	-0.34991	0.920246	-0.38024	0.703769	0.94524
AGO2	6154.136	-0.19728	0.520029	-0.37937	0.704416	0.94524
lncRNA:CR	85.39159	-1.49957	3.951301	-0.37951	0.704306	0.94524
IP3K2	1239.091	0.346032	0.907452	0.381322	0.702964	0.94524
CG4239	1901.546	-0.33155	0.874206	-0.37926	0.704492	0.94524
par-6	2207.338	-0.27272	0.718664	-0.37948	0.704329	0.94524
CG3902	1521.256	0.405598	1.069515	0.379235	0.704513	0.94524
CG7430	6633.885	-0.22344	0.588146	-0.37991	0.704015	0.94524
Notum	77.3715	1.50453	3.951362	0.380763	0.703379	0.94524
Prp31	427.4255	0.693394	1.818953	0.381205	0.703051	0.94524
CG4502	2831.976	0.291801	0.775109	0.376464	0.706572	0.945834
Taf11	345.4748	-0.84385	2.238792	-0.37692	0.706233	0.945834
CG4596	342.8043	-0.82409	2.188321	-0.37658	0.706483	0.945834
CG6693	1800.641	0.311371	0.826874	0.376564	0.706498	0.945834
Sbf	2001.508	-0.32656	0.863624	-0.37812	0.705339	0.945834
CG5805	1368.769	-0.33922	0.90118	-0.37642	0.706606	0.945834
CG8320	708.6535	0.424249	1.123511	0.37761	0.70572	0.945834
GstT2	227.9505	0.663743	1.75888	0.377367	0.705901	0.945834
Nfl	10896.11	0.206262	0.547782	0.376541	0.706515	0.945834
kek5	363.3596	-0.63327	1.68011	-0.37692	0.706232	0.945834
CG34404	2636.468	0.311807	0.827227	0.37693	0.706225	0.945834
lncRNA:CR	81.58778	-1.40421	3.71864	-0.37761	0.705717	0.945834
dpr6	8856.569	0.215368	0.569999	0.377839	0.70555	0.945834
Tgt	220.5575	0.981981	2.607655	0.376576	0.706489	0.945834
Pdp	5557.222	-0.22079	0.587312	-0.37593	0.706968	0.946042
CG10866	1961.503	-0.30845	0.820823	-0.37579	0.707075	0.946042
fax	7310.236	0.197256	0.525592	0.375303	0.707435	0.946042

Sting	633.0212	0.513349	1.367779	0.375316	0.707426	0.946042
ringer	5243.462	0.216199	0.575464	0.375695	0.707144	0.946042
CG11095	191.8577	-1.09346	2.913905	-0.37526	0.707469	0.946042
Cp1	3398.058	0.24414	0.650958	0.375048	0.707625	0.946093
CG5608	905.87	-0.62505	1.667438	-0.37485	0.707769	0.946129
Ripalpha	854.6685	0.443484	1.187072	0.373595	0.708706	0.946964
mlt	12984.2	0.191056	0.511695	0.373379	0.708866	0.946964
CanA-14F	3935.799	-0.23347	0.625274	-0.37339	0.708858	0.946964
OXA1L	940.0198	0.376704	1.008069	0.373689	0.708636	0.946964
CG13671	522.0322	-0.56931	1.526882	-0.37286	0.709252	0.947164
MFS18	1050.025	0.445871	1.195396	0.37299	0.709156	0.947164
CG13083	93.35659	1.471151	3.951275	0.372323	0.709652	0.947541
phol	895.6517	-0.57363	1.541557	-0.37211	0.709809	0.947593
nom	345.8434	0.636873	1.717564	0.3708	0.710786	0.947794
CG15536	396.2652	-0.85109	2.292729	-0.37121	0.710479	0.947794
Sodh-2	385.6235	0.53115	1.429355	0.371601	0.71019	0.947794
Vrp1	4142.697	-0.25754	0.693666	-0.37128	0.710432	0.947794
Cdep	14743.6	-0.15926	0.429492	-0.37081	0.710781	0.947794
asRNA:CR4	142.9108	0.951814	2.566484	0.370863	0.71074	0.947794
cmb	246.9786	0.905348	2.441216	0.370859	0.710742	0.947794
mnd	2617.995	0.310671	0.838463	0.370524	0.710992	0.94791
ND-B14.7	7324.365	0.181904	0.491765	0.3699	0.711457	0.948373
CG5903	12253.59	-0.16947	0.458534	-0.3696	0.711684	0.948518
CG5078	247.3124	-1.23718	3.376848	-0.36637	0.714089	0.949044
PolZ2	499.8365	-0.79862	2.178806	-0.36654	0.713961	0.949044
STUB1	2862.948	-0.26177	0.713944	-0.36665	0.713881	0.949044
CG9467	7273.006	-0.20434	0.556638	-0.3671	0.713545	0.949044
Mitofilin	2870.061	-0.26937	0.734054	-0.36696	0.713648	0.949044
CG13631	7829.689	-0.22142	0.602481	-0.36751	0.713241	0.949044
ssh	620.8564	-0.45143	1.224514	-0.36866	0.712381	0.949044
HmgD	3201.713	-0.25668	0.700478	-0.36643	0.714041	0.949044
Hmgs	1781.183	-0.46601	1.271365	-0.36654	0.71396	0.949044
dpr1	17025.73	0.159	0.43387	0.36647	0.714014	0.949044
CNMaR	660.7139	0.65638	1.79049	0.366592	0.713923	0.949044
Octbeta1R	1763.776	0.289372	0.787416	0.367496	0.713249	0.949044
CG40228	5445.466	-0.20043	0.545617	-0.36735	0.713357	0.949044
CG42540	19522.12	0.157636	0.428833	0.367593	0.713177	0.949044
Ca-Ma2d	630.1252	0.520985	1.421727	0.366445	0.714033	0.949044
smt3	8285.176	-0.17585	0.47722	-0.36848	0.712513	0.949044
CG14135	393.7632	0.678889	1.843358	0.368289	0.712658	0.949044
CG7903	957.0468	0.475939	1.300772	0.36589	0.714447	0.949206
Aps	3288.003	0.228478	0.624319	0.365964	0.714392	0.949206
dpr20	1560.778	-0.30707	0.840145	-0.36549	0.714743	0.949284
mRpL36	1247.282	-0.3255	0.890502	-0.36552	0.71472	0.949284
CG7483	833.1987	-0.39051	1.072505	-0.36411	0.715776	0.949338
RIC-3	4434.662	-0.22372	0.615651	-0.36338	0.716321	0.949338
Kdm4B	826.3339	-0.64696	1.780288	-0.3634	0.716306	0.949338
CG11076	4932.741	-0.203	0.557995	-0.36381	0.715999	0.949338
plx	1854.346	-0.28028	0.768624	-0.36465	0.715374	0.949338
RpS6	21200.84	0.154727	0.425243	0.363856	0.715965	0.949338

pyd	7260.574	-0.20397	0.560219	-0.36409	0.715794	0.949338
CG3511	1369.289	0.550651	1.511198	0.364381	0.715574	0.949338
nod	101.5602	1.442325	3.951311	0.365024	0.715093	0.949338
Taf6	1313.966	-0.44337	1.215237	-0.36484	0.71523	0.949338
MED11	607.3525	0.444308	1.218983	0.364491	0.715491	0.949338
CG17364	2761.989	0.262489	0.722195	0.36346	0.716261	0.949338
ERR	293.7886	0.605989	1.659221	0.365225	0.714943	0.949338
CycY	2147.206	-0.27105	0.748545	-0.36211	0.717271	0.949565
Dtd	260.5691	0.659087	1.824137	0.361314	0.717864	0.949565
rin	4875.308	-0.20254	0.559307	-0.36212	0.717262	0.949565
His4r	52266.4	0.146224	0.404241	0.361723	0.717559	0.949565
CG11110	738.7747	-0.60749	1.6831	-0.36093	0.71815	0.949565
CG8613	3439.179	-0.2559	0.707135	-0.36189	0.717437	0.949565
Tmem18	558.7611	-0.45918	1.266461	-0.36257	0.716928	0.949565
brk	1515.293	0.304489	0.841249	0.361948	0.717391	0.949565
slim	9298.784	0.193136	0.533471	0.362037	0.717324	0.949565
ebi	1199.984	0.352461	0.976755	0.360849	0.718213	0.949565
Tom20	6663.989	0.187091	0.51761	0.361451	0.717762	0.949565
FucTA	2218.731	0.327105	0.906669	0.360776	0.718267	0.949565
Tdrd3	412.909	0.51428	1.421904	0.361684	0.717588	0.949565
Srp9	1617.52	0.301416	0.834732	0.361094	0.718029	0.949565
mRpL27	1752.405	-0.30377	0.838109	-0.36245	0.717019	0.949565
CG5938	4004.045	-0.21811	0.604986	-0.36052	0.718458	0.949661
lncRNA:Hs	380337.8	-0.14106	0.391832	-0.35999	0.718853	0.950027
mthl14	616.625	0.567672	1.578319	0.359669	0.719095	0.95019
AdamTS-B	85.83307	1.128649	3.153413	0.357914	0.720408	0.95047
crq	3507.32	0.220001	0.616501	0.356854	0.721201	0.95047
plum	4619.678	-0.22124	0.619003	-0.35741	0.720785	0.95047
CG9344	485.4665	-0.52215	1.463041	-0.35689	0.721172	0.95047
CG1888	1528.566	0.39693	1.112217	0.356881	0.721181	0.95047
AIMP1	661.2927	0.702251	1.966397	0.357126	0.720998	0.95047
asRNA:CR3	114.7376	1.400651	3.914128	0.357845	0.720459	0.95047
CG17454	5802.278	0.197754	0.5523	0.358055	0.720302	0.95047
snama	919.8431	0.508431	1.417575	0.358662	0.719848	0.95047
miple1	40312.28	0.138079	0.386677	0.357091	0.721024	0.95047
Tim17b	11343.93	0.169115	0.472266	0.358094	0.720273	0.95047
Sgt1	1456.127	0.324014	0.906489	0.357438	0.720764	0.95047
CR46425	381.8444	0.613221	1.712002	0.358189	0.720202	0.95047
lncRNA:CR	278.743	-0.75089	2.095149	-0.35839	0.720049	0.95047
CG7550	271.881	0.692484	1.931702	0.358484	0.719981	0.95047
slp1	486.8744	0.802715	2.235794	0.359029	0.719573	0.95047
Rpn11	2084.183	0.265808	0.746377	0.356131	0.721742	0.950531
CAH7	32752.28	-0.17683	0.496919	-0.35584	0.721958	0.950531
e(y)3	1176.331	-0.36063	1.012533	-0.35616	0.721718	0.950531
pit	397.3959	-0.68972	1.937651	-0.35596	0.721872	0.950531
SF2	3064.214	-0.24601	0.690027	-0.35653	0.721445	0.950531
RpS21	5029.494	0.20543	0.576943	0.356066	0.721791	0.950531
metro	1254.64	-0.45608	1.282344	-0.35566	0.722094	0.950554
scrt	27235.48	0.154446	0.434562	0.355406	0.722285	0.95065
CG3397	255.0392	0.928484	2.615126	0.355044	0.722557	0.950749

CG11123	533.091	0.705204	1.988321	0.354673	0.722834	0.950749
Sesn	201.636	-0.81643	2.300099	-0.35496	0.722622	0.950749
mRpS17	1369.265	0.330191	0.930717	0.35477	0.722762	0.950749
CG5888	4396.653	-0.23613	0.666698	-0.35418	0.723203	0.950766
Drat	10061.78	0.168608	0.476039	0.354188	0.723198	0.950766
app	3221.787	-0.23356	0.658901	-0.35447	0.72299	0.950766
MED31	1921.526	-0.29321	0.829194	-0.3536	0.723636	0.951073
grass	92.57819	1.145656	3.240397	0.353554	0.723673	0.951073
CG31999	3381.486	0.243456	0.689445	0.353119	0.723999	0.951345
CG11007	2009.891	0.298103	0.846326	0.352232	0.724665	0.951993
Strip	182.6338	-0.76661	2.176983	-0.35215	0.724729	0.951993
lok	280.5502	-0.74999	2.131017	-0.35194	0.724884	0.952041
Lkb1	483.9113	0.66241	1.887089	0.351022	0.725572	0.95266
dare	585.302	0.552559	1.574272	0.350993	0.725593	0.95266
CG11839	139.1557	-1.00335	2.860765	-0.35073	0.725793	0.95272
dom	3470.887	-0.23505	0.6707	-0.35046	0.725995	0.95272
Prosalpha6	2596.177	0.441806	1.260415	0.350524	0.725945	0.95272
T48	112.8313	0.933716	2.666971	0.350104	0.726261	0.952913
Zir	4009.219	0.224068	0.641166	0.34947	0.726736	0.953029
CG11882	114.8885	-1.29495	3.70282	-0.34972	0.726549	0.953029
CG12159	1992.359	-0.26071	0.745714	-0.34961	0.726628	0.953029
CG9577	785.6772	-0.49865	1.427727	-0.34926	0.726892	0.953029
CG13928	19194.26	0.16273	0.466021	0.349191	0.726946	0.953029
Tango10	1522.239	0.32729	0.937693	0.349037	0.727061	0.953029
LSm7	465.8023	-0.46502	1.33407	-0.34857	0.72741	0.953331
Axn	1019.92	0.34635	0.994431	0.348289	0.727623	0.953453
Art4	551.5491	-0.57961	1.669779	-0.34712	0.728501	0.953676
pirk	464.7927	0.693088	1.994973	0.347417	0.728278	0.953676
CG42699	271.1916	0.837726	2.408397	0.347836	0.727964	0.953676
nrm	7044.866	0.174327	0.502219	0.347113	0.728506	0.953676
CG43367	3895.417	0.21095	0.606855	0.347613	0.728131	0.953676
CG15012	5590.322	0.18692	0.538332	0.347221	0.728426	0.953676
CG8441	1611.063	-0.52314	1.510764	-0.34628	0.729135	0.953878
SC35	2998.917	0.225905	0.652132	0.346409	0.729035	0.953878
CG13716	361.8708	-0.65468	1.890036	-0.34638	0.729054	0.953878
CG3808	393.024	-0.52595	1.518362	-0.34639	0.729049	0.953878
rngo	345.4813	0.706152	2.040851	0.346009	0.729336	0.953985
beat-Ia	1874.236	-0.29357	0.849854	-0.34543	0.729771	0.95438
CG11178	246.2627	-0.85123	2.466166	-0.34516	0.729971	0.95438
CG9723	176.1903	-0.96715	2.802263	-0.34513	0.729995	0.95438
CG5316	551.3561	-0.49898	1.450944	-0.3439	0.730923	0.954873
ND-B15	4476.666	0.195981	0.569697	0.344009	0.73084	0.954873
bru2	106.6309	-1.17294	3.408447	-0.34413	0.730751	0.954873
CG9065	6980.891	0.234355	0.681584	0.34384	0.730967	0.954873
CG6847	416.1217	-0.61827	1.796277	-0.3442	0.730698	0.954873
RpS20	9619.395	-0.16318	0.474894	-0.34362	0.731132	0.954911
mRpL52	1374.698	0.306449	0.892354	0.343417	0.731285	0.954911
RNaseX25	1737.235	-0.34042	0.991522	-0.34333	0.731353	0.954911
CG42271	1846.02	0.261095	0.760927	0.343127	0.731503	0.954952
CG32448	756.8622	0.416086	1.219418	0.341217	0.732941	0.95549

CG10958	290.3258	0.955027	2.799388	0.341156	0.732986	0.95549
AP-1gamm	3592.114	0.21535	0.629617	0.342033	0.732326	0.95549
CG11474	2405.73	0.351936	1.027814	0.342413	0.73204	0.95549
CG8180	262.3936	0.570339	1.669546	0.341613	0.732642	0.95549
hoe2	826.4251	-0.63034	1.843029	-0.34201	0.732343	0.95549
apolpp	9364.012	0.158352	0.463797	0.341425	0.732784	0.95549
CG42748	6202.251	-0.17808	0.521722	-0.34134	0.732849	0.95549
mbl	24873.02	0.186366	0.545675	0.341533	0.732702	0.95549
Hn	86.67969	-0.97944	2.872693	-0.34095	0.733144	0.955539
CG10462	525.4799	-0.57255	1.680713	-0.34066	0.733361	0.955668
Wbp2	9416.726	0.15878	0.466862	0.340102	0.73378	0.956058
CG15537	4850.899	0.221861	0.653763	0.33936	0.734339	0.956631
Nmd3	894.338	-0.39075	1.153427	-0.33877	0.734781	0.956746
lncRNA:CR	116.6638	1.299461	3.834104	0.338922	0.734669	0.956746
CG10654	1963.172	-0.29779	0.879029	-0.33877	0.734785	0.956746
RpS27	8717.519	0.169675	0.501175	0.338555	0.734945	0.9568
CG31522	3255.197	0.213558	0.63477	0.336434	0.736544	0.956985
homer	1728.739	-0.33508	1.001146	-0.3347	0.737851	0.956985
CG31897	77.56609	1.278461	3.796358	0.33676	0.736298	0.956985
Ugt37D1	909.1081	0.574094	1.699748	0.337752	0.73555	0.956985
CG12402	683.2935	-0.46096	1.376146	-0.33496	0.737653	0.956985
CG31156	540.0585	-0.54965	1.635157	-0.33614	0.736762	0.956985
CG33107	1802.7	0.324455	0.962199	0.337201	0.735965	0.956985
msi	11326.03	-0.15422	0.459574	-0.33558	0.73719	0.956985
galla-1	2957.018	-0.30585	0.91432	-0.33451	0.737991	0.956985
GIIIspla2	975.968	-0.34048	1.007356	-0.338	0.735367	0.956985
CG12659	343.1878	0.748312	2.239088	0.334204	0.738226	0.956985
CG15312	2124.561	-0.28082	0.83838	-0.33495	0.737661	0.956985
CG10082	4813.466	-0.18919	0.565309	-0.33466	0.73788	0.956985
CG1418	705.964	-0.66281	1.968748	-0.33667	0.736369	0.956985
ced-6	4653.738	0.225845	0.675862	0.334158	0.73826	0.956985
Coq9	618.5577	0.509908	1.509158	0.337876	0.735457	0.956985
sw	760.0287	-0.42936	1.273747	-0.33708	0.736056	0.956985
sona	438.1611	-0.6654	1.971464	-0.33751	0.73573	0.956985
C3G	4248.7	0.253127	0.754029	0.335699	0.737098	0.956985
CG42360	1086.59	0.331749	0.988699	0.335541	0.737217	0.956985
RpL8	15388.23	-0.15627	0.467386	-0.33435	0.738118	0.956985
TyrRS-m	159.8571	1.012185	3.027187	0.334365	0.738104	0.956985
dally	19831.58	-0.19125	0.570982	-0.33495	0.737665	0.956985
Sem1	1187.301	-0.30736	0.919981	-0.3341	0.738307	0.956985
CG9149	472.7486	-0.62592	1.870355	-0.33465	0.737887	0.956985
CG15014	592.9777	-0.65948	1.960468	-0.33639	0.736576	0.956985
Cds	15423.79	-0.15214	0.453778	-0.33528	0.737417	0.956985
CG14621	74.13902	1.163042	3.485961	0.333636	0.738654	0.957281
CG31139	209.3177	1.228542	3.684034	0.333477	0.738774	0.957281
CG6766	4923.616	0.268522	0.806011	0.333149	0.739022	0.957448
inc	4181.918	-0.24816	0.74608	-0.33262	0.739422	0.957812
Pgd	582.5518	-0.58773	1.78521	-0.32922	0.741986	0.958464
CG10979	541.4332	0.551977	1.671006	0.330326	0.741153	0.958464
CG10413	1292.82	0.446084	1.355541	0.329082	0.742094	0.958464

CG10098	1673.352	-0.43737	1.322761	-0.33065	0.740909	0.958464
Sap47	40196.45	0.128257	0.389251	0.329497	0.74178	0.958464
ATPsyndel	9307.079	-0.16839	0.507653	-0.3317	0.740113	0.958464
CG12643	327.0235	0.790165	2.396917	0.329659	0.741658	0.958464
CG13148	351.3575	0.669635	2.035684	0.328948	0.742195	0.958464
CG17977	1775.828	-0.28103	0.848377	-0.33126	0.740449	0.958464
unc80	5092.058	0.192505	0.585098	0.329014	0.742145	0.958464
mRpL24	2521.481	-0.2333	0.708998	-0.32905	0.742115	0.958464
endos	5588.047	-0.19107	0.579708	-0.32959	0.74171	0.958464
CG3257	2618.635	-0.2398	0.727476	-0.32964	0.741673	0.958464
alpha-Spec	8177.289	0.168095	0.50963	0.329838	0.741522	0.958464
nero	1408.637	-0.43128	1.305928	-0.33025	0.741212	0.958464
CG42751	107.457	-1.16339	3.535045	-0.3291	0.742079	0.958464
Acn	4127.881	0.194982	0.591049	0.329892	0.741481	0.958464
Argk	53934.54	-0.12302	0.372823	-0.32996	0.741431	0.958464
Sf3b6	2028.665	-0.24303	0.738439	-0.32911	0.742075	0.958464
meng	1361.341	0.305911	0.936517	0.326648	0.743934	0.958549
CG13398	1545.016	0.290838	0.89081	0.326487	0.744056	0.958549
Mst89B	203.2573	1.22087	3.721649	0.328045	0.742877	0.958549
Vha100-1	16898.57	-0.15907	0.484047	-0.32862	0.742443	0.958549
Gycbeta10	3591.72	0.204508	0.625687	0.326853	0.743779	0.958549
vimar	959.8378	0.484861	1.478729	0.32789	0.742995	0.958549
CG7065	855.5398	-0.41001	1.256014	-0.32643	0.744096	0.958549
pic	1336.499	0.309202	0.9475	0.326334	0.744171	0.958549
tamo	1692.043	-0.27745	0.849914	-0.32645	0.744086	0.958549
CG45002	4160.666	0.211838	0.645686	0.328082	0.74285	0.958549
da	2255.889	-0.25045	0.764958	-0.3274	0.743365	0.958549
Apl	136.8548	-0.70811	2.16752	-0.32669	0.743901	0.958549
Dab	4265.164	0.198347	0.605856	0.327383	0.743378	0.958549
CG14153	129.5452	-0.96276	2.93949	-0.32752	0.743271	0.958549
CG7565	1460.777	-0.36735	1.123292	-0.32703	0.743645	0.958549
CG5126	311.487	0.63323	1.939778	0.326445	0.744088	0.958549
Fur1	3874.548	-0.19377	0.596475	-0.32486	0.745286	0.959831
Unc-76	10200.46	0.147046	0.45799	0.321069	0.748158	0.959839
CG16903	114.6255	0.872991	2.713441	0.321729	0.747658	0.959839
CG2931	886.1565	0.357976	1.104237	0.324184	0.745799	0.959839
CG7239	466.4155	-0.4707	1.468433	-0.32055	0.748555	0.959839
MED20	287.5876	0.666377	2.086615	0.319358	0.749455	0.959839
Gdi	14600.12	-0.14288	0.445389	-0.3208	0.748361	0.959839
Spn31A	838.5208	0.580273	1.800311	0.322318	0.747212	0.959839
CG5355	1276.237	-0.31757	0.992645	-0.31992	0.749025	0.959839
CG18063	62.38617	1.236756	3.857997	0.32057	0.748537	0.959839
Tsp39D	2588.333	0.266776	0.824691	0.323486	0.746327	0.959839
CG31301	767.6935	0.442469	1.381652	0.320246	0.748782	0.959839
CG5346	160.0005	0.938052	2.899071	0.32357	0.746264	0.959839
Orct	7972.286	0.190088	0.588164	0.323189	0.746552	0.959839
CG6607	578.1574	-0.54815	1.714363	-0.31974	0.749167	0.959839
CG5913	272.9673	0.777702	2.434526	0.319447	0.749388	0.959839
Sap-r	10086.89	0.162522	0.505312	0.321628	0.747734	0.959839
Hsf	3110.38	-0.22152	0.682775	-0.32445	0.745601	0.959839

CG12895	1738.346	0.298222	0.934369	0.319169	0.749598	0.959839
Csgalnact	1996.474	0.323902	1.009839	0.320746	0.748403	0.959839
Eaf	1471.769	-0.29613	0.917694	-0.32269	0.746931	0.959839
zormin	2782.837	-0.22415	0.694223	-0.32288	0.746785	0.959839
CG11883	3117.93	-0.25065	0.783195	-0.32003	0.748946	0.959839
Ptp99A	6445.899	0.167277	0.523857	0.319319	0.749485	0.959839
SteXh:CG4	150.3966	-0.97424	3.00851	-0.32383	0.746069	0.959839
Hk	16371.49	-0.13974	0.434707	-0.32146	0.747864	0.959839
CG4610	767.5352	-0.63466	1.970421	-0.3221	0.74738	0.959839
ytr	5416.028	0.197683	0.614973	0.32145	0.74787	0.959839
lncRNA:CR	118.5931	-0.97167	3.025878	-0.32112	0.748118	0.959839
dmt	207.6692	-0.66351	2.053106	-0.32317	0.746564	0.959839
CG6023	562.9281	-0.57883	1.809562	-0.31987	0.749065	0.959839
ftz-f1	10531.39	0.172655	0.532023	0.324526	0.74554	0.959839
CG32187	139.8579	0.756236	2.346348	0.322303	0.747223	0.959839
Arl1	630.8581	-0.47628	1.489529	-0.31976	0.749154	0.959839
Sse	175.09	-0.90137	2.784769	-0.32368	0.746181	0.959839
ScsbetaG	435.1072	-0.72674	2.270451	-0.32009	0.748902	0.959839
tty	3207.164	0.222254	0.688672	0.322729	0.746901	0.959839
CG9396	168.9325	1.035798	3.247133	0.318988	0.749735	0.959862
CG31974	3684.34	-0.22868	0.718128	-0.31843	0.750156	0.959996
CG10209	148.7994	0.905768	2.844948	0.318378	0.750199	0.959996
GstO2	1986.842	-0.49001	1.537956	-0.31861	0.750022	0.959996
Sin3A	9553.707	-0.15559	0.488993	-0.31818	0.75035	0.960036
Ccp84Ab	71.81387	1.230281	3.870352	0.317873	0.750581	0.960129
Zasp67	79.33309	1.143229	3.597688	0.317768	0.750661	0.960129
CG14427	85.10547	-1.01727	3.211277	-0.31678	0.751411	0.960934
Top2	920.1402	-0.38299	1.209643	-0.31662	0.751535	0.96094
Gnf1	687.6609	-0.49967	1.579974	-0.31625	0.75181	0.961069
128up	1104.988	-0.41693	1.320568	-0.31572	0.752214	0.961069
Snoo	1992.938	-0.28122	0.889984	-0.31598	0.752018	0.961069
roh	15429.43	0.139926	0.443454	0.315536	0.752355	0.961069
kdn	4785.006	0.209558	0.663073	0.316041	0.751971	0.961069
RpII215	3121.99	0.243619	0.771698	0.315692	0.752237	0.961069
CG17721	794.7781	-0.5213	1.654673	-0.31505	0.752724	0.961097
CG14314	1891.556	-0.2986	0.948515	-0.31481	0.752904	0.961097
REPTOR	6834.461	0.21398	0.679373	0.314966	0.752787	0.961097
gammaSna	2550.753	0.225925	0.717863	0.314719	0.752975	0.961097
Ugt316A1	308.8	-0.74256	2.359041	-0.31477	0.752934	0.961097
CG10011	2661.143	-0.23112	0.735412	-0.31427	0.753319	0.961383
CG31729	3923.519	-0.21988	0.704339	-0.31218	0.754905	0.96142
CG9356	552.1654	-0.56326	1.799149	-0.31307	0.754228	0.96142
Abi	1504.374	-0.26256	0.838489	-0.31313	0.754178	0.96142
repo	3197.007	0.247764	0.790783	0.313315	0.754041	0.96142
hdc	6139.781	0.179827	0.574767	0.31287	0.75438	0.96142
Tbce	1956.751	-0.23566	0.752915	-0.313	0.75428	0.96142
GEFmeso	2158.767	-0.23927	0.763858	-0.31323	0.754103	0.96142
ewg	2543.663	0.212396	0.677581	0.313463	0.753929	0.96142
b	299.3215	-0.82516	2.639934	-0.31257	0.754607	0.96142
ps	89600.41	0.120485	0.385784	0.312312	0.754803	0.96142

ATPsynB	25829.25	-0.13148	0.420877	-0.3124	0.75474	0.96142
aay	6764.415	0.183017	0.585607	0.312525	0.754642	0.96142
Usp47	2483.328	-0.28893	0.920473	-0.3139	0.753601	0.96142
Pi4KIIalpha	715.0948	-0.46475	1.490452	-0.31182	0.75518	0.961618
INPP5E	496.2699	0.489185	1.569993	0.311584	0.755357	0.96169
Vps16B	208.8839	0.753673	2.423896	0.310935	0.75585	0.962166
Taz	973.9862	0.49755	1.601336	0.310709	0.756022	0.96221
RpS7	14906.94	0.136449	0.439569	0.310416	0.756245	0.96221
z	821.7462	-0.34613	1.114806	-0.31048	0.756193	0.96221
DppIII	1206.967	0.367449	1.184387	0.310244	0.756375	0.962213
Syx17	781.7536	-0.43204	1.393948	-0.30994	0.756606	0.962213
Cyp4g15	5059.36	0.190441	0.614219	0.310054	0.75652	0.962213
EbpIII	4792.494	0.354238	1.143763	0.309713	0.756779	0.96228
Or85f	240.5136	-0.8315	2.689919	-0.30912	0.757231	0.962702
Dg	3805.672	-0.22949	0.743425	-0.3087	0.757553	0.962958
CG6472	229.85	-0.90199	2.925751	-0.30829	0.757858	0.963067
CG30015	2725.062	-0.22298	0.723335	-0.30827	0.757878	0.963067
stck	3742.464	-0.25866	0.839545	-0.3081	0.758009	0.96308
RpL34a	3399.117	0.207959	0.675478	0.30787	0.758182	0.963147
Idh	8953.714	-0.192	0.623951	-0.30771	0.758304	0.963151
CG8861	17099.41	0.17483	0.568916	0.307304	0.758612	0.963389
nAChRalph	2550.82	-0.21858	0.713731	-0.30625	0.759416	0.964105
EndoB	5941.038	-0.16097	0.525568	-0.30627	0.7594	0.964105
CLIP-190	11118.04	-0.1806	0.590677	-0.30575	0.759799	0.964317
CG2247	1549.45	0.291314	0.953472	0.30553	0.759963	0.964317
Lon	925.7232	0.31592	1.034454	0.305398	0.760063	0.964317
Dronc	300.5985	-0.83993	2.7486	-0.30558	0.759921	0.964317
lncRNA:CR	129.583	0.996061	3.263553	0.305208	0.760208	0.964348
PIG-K	3135.546	-0.19347	0.651984	-0.29674	0.766665	0.964358
CG3527	481.3181	-0.53973	1.806435	-0.29878	0.765108	0.964358
CG12147	109.7869	1.178901	3.951336	0.298355	0.765432	0.964358
eIF4A	26683.02	0.120809	0.407859	0.296202	0.767076	0.964358
CG11865	108.101	1.166099	3.95134	0.295115	0.767906	0.964358
CG10466	254.5473	-0.66841	2.248186	-0.29731	0.76623	0.964358
CG9636	5231.857	0.180905	0.596322	0.303368	0.761609	0.964358
Ras85D	6313.863	-0.16601	0.555973	-0.2986	0.765247	0.964358
CG8483	114.4488	1.178949	3.951319	0.298369	0.765422	0.964358
EMC2B	930.78	-0.35261	1.166433	-0.30229	0.762428	0.964358
wrd	3626.306	0.19341	0.646759	0.299044	0.764906	0.964358
CG4733	118.2633	-1.01419	3.406451	-0.29773	0.765912	0.964358
CG4783	76.45458	1.178336	3.951523	0.298198	0.765552	0.964358
dsd	3563.431	0.214398	0.703584	0.304723	0.760577	0.964358
cindr	8838.798	0.16759	0.566884	0.295633	0.76751	0.964358
schlank	3213.494	0.228084	0.771837	0.295508	0.767606	0.964358
CG14442	3691.593	0.20568	0.696801	0.295178	0.767858	0.964358
CG2124	83.97511	-1.10234	3.709248	-0.29719	0.766324	0.964358
IntS8	77.26701	-1.17605	3.933245	-0.299	0.764938	0.964358
CG17385	3597.877	-0.18537	0.627409	-0.29546	0.767646	0.964358
CG18371	103.4934	1.178822	3.951362	0.298333	0.765449	0.964358
shot	16806.4	0.144762	0.483803	0.299216	0.764775	0.964358

Qsox1	2595.335	0.226994	0.758577	0.299237	0.764759	0.964358
CG13197	763.2916	0.343564	1.155789	0.297255	0.766272	0.964358
CG18858	117.2694	-0.86595	2.904708	-0.29812	0.765611	0.964358
gus	8940.389	0.160674	0.543981	0.295368	0.767713	0.964358
zfh1	1603.143	0.2889	0.9699	0.297866	0.765805	0.964358
ec	108.0494	-0.88961	2.962561	-0.30028	0.763961	0.964358
Glyat	69.69488	1.178163	3.951583	0.29815	0.765589	0.964358
snoRNA:Ps	106.9898	1.178867	3.951347	0.298346	0.765439	0.964358
vari	924.7074	0.343057	1.147623	0.298929	0.764995	0.964358
UQCR-C2	7253.932	0.159729	0.533814	0.299223	0.76477	0.964358
pzg	1054.478	0.389385	1.319335	0.295137	0.767889	0.964358
CG42789	94.16967	1.178687	3.951407	0.298296	0.765478	0.964358
CG43229	75.98839	1.178328	3.951527	0.298196	0.765554	0.964358
CG13901	356.9434	0.57143	1.887606	0.302727	0.762098	0.964358
asRNA:CR4	834.7282	-0.3789	1.267745	-0.29888	0.765034	0.964358
sick	6517.894	-0.15116	0.507617	-0.29778	0.765874	0.964358
lncRNA:CR	141.7207	1.179177	3.951239	0.298432	0.765373	0.964358
lncRNA:CR	77.5994	1.007172	3.401693	0.296079	0.767169	0.964358
lncRNA:CR	94.16967	1.178687	3.951407	0.298296	0.765478	0.964358
lncRNA:CR	113.7495	1.178942	3.951321	0.298366	0.765424	0.964358
CG44774	2024.629	0.222438	0.730999	0.304293	0.760905	0.964358
asRNA:CR4	76.22149	1.178334	3.951525	0.298197	0.765553	0.964358
lncRNA:CR	167.1278	1.179344	3.951189	0.298478	0.765338	0.964358
lncRNA:CR	86.94378	1.178563	3.951449	0.298261	0.765504	0.964358
elgi	1803.942	-0.43317	1.423414	-0.30432	0.760887	0.964358
CR46405	82.99096	-1.16443	3.834482	-0.30367	0.761377	0.964358
CG46466	2468.602	0.237698	0.804086	0.295612	0.767526	0.964358
CG13920	2834.365	0.191831	0.649361	0.295415	0.767677	0.964358
Jafrac2	1106.973	-0.49437	1.630648	-0.30318	0.761756	0.964358
CG14965	440.0291	0.675135	2.242771	0.301027	0.763394	0.964358
CG14977	1549.504	0.271591	0.918582	0.295663	0.767487	0.964358
TfIIEbeta	1076.508	-0.29181	0.978042	-0.29836	0.765425	0.964358
nAChRbeta	61259.03	0.126587	0.420745	0.300864	0.763518	0.964358
RpS15Aa	12451.1	0.134083	0.442783	0.302818	0.762029	0.964358
CtsB	6794.456	-0.16462	0.549751	-0.29944	0.764606	0.964358
CG9915	576.9122	-0.54415	1.83151	-0.2971	0.766387	0.964358
Edc3	2073.286	-0.26016	0.868973	-0.29939	0.764641	0.964358
Ilp5	134.0286	1.179132	3.951258	0.298419	0.765383	0.964358
BI-1	10893.4	-0.15029	0.505092	-0.29755	0.766047	0.964358
frm	263.0091	0.753231	2.491262	0.302349	0.762386	0.964358
CG4835	130.5322	1.179107	3.951268	0.298412	0.765389	0.964358
CG15362	83.44738	1.178494	3.951472	0.298242	0.765519	0.964358
CG9641	1461.844	-0.43453	1.475574	-0.29448	0.76839	0.964815
Mical	3910.45	0.188472	0.640515	0.294251	0.768566	0.964849
lncRNA:CR	77.14827	0.947681	3.221969	0.294131	0.768658	0.964849
CG5079	72.57443	-0.98707	3.365811	-0.29326	0.769321	0.964862
CG33641	159.7024	0.638763	2.173263	0.293919	0.76882	0.964862
CG34228	844.5113	0.385595	1.315696	0.293073	0.769466	0.964862
CG43371	74.76865	1.159906	3.95153	0.293533	0.769114	0.964862
CG43901	7414.931	0.172435	0.586945	0.293784	0.768923	0.964862

vih	1859.813	-0.22746	0.776265	-0.29302	0.76951	0.964862
RpS10b	5918.257	0.153339	0.522689	0.293366	0.769243	0.964862
cer	68.67672	0.880328	3.012215	0.292253	0.770093	0.965293
CG3589	181.5108	-0.70322	2.404998	-0.2924	0.769982	0.965293
Flo2	3187.239	-0.1817	0.623053	-0.29162	0.770576	0.965746
JMJD4	411.9368	-0.86674	2.976717	-0.29117	0.770918	0.966025
CG32772	1813.651	0.300486	1.033407	0.290772	0.771225	0.966108
eys	4575.109	0.168871	0.580624	0.290844	0.771171	0.966108
heix	118.2783	-0.78824	2.713187	-0.29052	0.771417	0.966198
lncRNA:CR	2197.908	0.250682	0.86471	0.289903	0.77189	0.966639
PRL-1	1466.03	-0.2413	0.833581	-0.28948	0.772217	0.966857
CG10237	539.1725	-0.59399	2.052753	-0.28936	0.772305	0.966857
lncRNA:CR	90.30182	-1.14263	3.951212	-0.28919	0.772439	0.966874
ND-AGGG	3975.13	0.174116	0.602476	0.289001	0.772581	0.9669
Eip78C	4620.303	0.216448	0.750664	0.288342	0.773085	0.966997
CG33169	209.7389	0.538484	1.872073	0.287641	0.773622	0.966997
nonA-l	327.9909	-0.57646	1.998402	-0.28846	0.772993	0.966997
ETHR	1083.586	-0.52543	1.826281	-0.28771	0.773571	0.966997
tio	138.1825	-0.75539	2.61839	-0.28849	0.772969	0.966997
CG6845	1232.878	-0.37007	1.286216	-0.28772	0.77356	0.966997
CG2021	569.1832	-0.36522	1.269629	-0.28766	0.77361	0.966997
CG32109	2548.293	0.215038	0.747431	0.287703	0.773574	0.966997
mt:ND6	3578.459	0.185618	0.646338	0.287184	0.773971	0.967283
ex	86.46807	-1.05234	3.669513	-0.28678	0.774281	0.967351
drosha	211.8506	0.563658	1.966446	0.286638	0.77439	0.967351
CG12004	2877.734	-0.18657	0.650356	-0.28687	0.774209	0.967351
Hydr2	3639.365	-0.17546	0.612444	-0.28648	0.774507	0.967351
CG7379	1784.06	-0.29119	1.017912	-0.28607	0.774824	0.967537
CR32745	586.6551	0.435796	1.523896	0.285975	0.774897	0.967537
CG1965	1865.595	-0.23561	0.824929	-0.28561	0.775175	0.967733
scrib	5373.386	0.152962	0.535909	0.285426	0.775318	0.967761
CG5281	1887.801	-0.22821	0.802559	-0.28435	0.776139	0.967777
Ublcp1	363.321	0.527992	1.857038	0.28432	0.776165	0.967777
Opbp	470.2406	-0.64166	2.256536	-0.28435	0.776139	0.967777
CaMKI	18345.74	0.117169	0.412121	0.284308	0.776175	0.967777
AP-1-2bet	5501.927	-0.15966	0.560904	-0.28466	0.775907	0.967777
Or71a	298.252	0.741517	2.607813	0.284344	0.776147	0.967777
CG13300	532.1499	-0.40597	1.425592	-0.28477	0.775817	0.967777
Pex5	837.3126	0.447097	1.581967	0.282621	0.777468	0.967971
CG6379	938.16	0.301523	1.064528	0.283246	0.776988	0.967971
CG7810	723.0561	-0.37659	1.335901	-0.2819	0.778019	0.967971
RpI1	289.5075	0.667695	2.367088	0.282074	0.777886	0.967971
CG8617	974.8407	-0.43948	1.553306	-0.28293	0.777228	0.967971
Syngr	12504.44	0.145764	0.51702	0.281931	0.777997	0.967971
Cog3	209.1248	-0.77539	2.739263	-0.28307	0.777127	0.967971
ema	3811.945	0.176535	0.623296	0.283229	0.777002	0.967971
RpS16	6676.64	0.162517	0.575419	0.282433	0.777612	0.967971
blw	42623.09	-0.11203	0.395504	-0.28326	0.776981	0.967971
Rpi	736.3489	-0.52262	1.846912	-0.28297	0.7772	0.967971
Dop1R2	1038.597	0.29313	1.036872	0.282706	0.777402	0.967971

mRpL21	1384.549	-0.2659	0.941996	-0.28227	0.777736	0.967971
ABCA	2337.644	-0.20688	0.732585	-0.2824	0.777637	0.967971
l(2)k14505	1869.013	0.336173	1.193976	0.281558	0.778283	0.968149
Cyp310a1	321.7803	0.637377	2.267589	0.281082	0.778648	0.968153
Den1	1637.3	0.2353	0.836687	0.281228	0.778536	0.968153
Traf6	176.5762	0.585652	2.082225	0.281262	0.778509	0.968153
MED15	238.0603	0.499028	1.77646	0.280912	0.778778	0.968165
CG7530	1809.258	-0.3537	1.26112	-0.28047	0.779121	0.968441
Ssb-c31a	3123.385	-0.17813	0.637431	-0.27945	0.779901	0.968477
Klp31E	311.9918	-0.51806	1.860975	-0.27838	0.780718	0.968477
GstZ2	497.9078	0.586401	2.099512	0.279303	0.780012	0.968477
RpL34b	6989.207	0.150115	0.536067	0.28003	0.779455	0.968477
CG31220	566.3727	0.371162	1.330056	0.279057	0.780201	0.968477
l(1)G0289	6974.961	0.1429	0.513123	0.27849	0.780636	0.968477
CG17715	397.204	0.465918	1.665506	0.279746	0.779673	0.968477
CG34172	181.5799	0.839818	3.000424	0.2799	0.779554	0.968477
spoon	2660.749	-0.21196	0.756925	-0.28002	0.77946	0.968477
mRpS35	1552.402	0.246685	0.884185	0.278998	0.780247	0.968477
CG11583	356.1688	-0.52687	1.88579	-0.27939	0.779945	0.968477
CG6340	3031.469	-0.18114	0.649997	-0.27869	0.780486	0.968477
Nelf-E	1032.844	-0.28048	1.007319	-0.27844	0.780674	0.968477
Ho	1640.405	0.408342	1.469619	0.277856	0.781123	0.968829
Lar	6097.925	-0.14253	0.514272	-0.27715	0.781662	0.96893
Arf51F	5699.026	0.187182	0.675453	0.277121	0.781688	0.96893
Pih1D1	2766.607	-0.21868	0.788242	-0.27742	0.781456	0.96893
PCID2	262.2778	0.755413	2.721885	0.277533	0.781371	0.96893
CG43610	75.32379	1.094215	3.951334	0.276923	0.781839	0.968969
Prp18	324.8669	0.524095	1.894871	0.276586	0.782098	0.96914
CG6066	234.4436	0.540648	1.95843	0.276062	0.782501	0.969359
tst	178.0906	0.88203	3.197073	0.275887	0.782635	0.969359
rhea	6722.501	0.185335	0.671784	0.275884	0.782637	0.969359
CtsF	7365.542	-0.14013	0.50941	-0.27509	0.783248	0.969507
CG5446	2938.368	-0.18365	0.66865	-0.27466	0.783579	0.969507
TM9SF4	2715.049	0.21526	0.782389	0.275132	0.783215	0.969507
CG15908	1812.333	0.261153	0.950934	0.274627	0.783602	0.969507
boca	1668.706	0.22144	0.804646	0.275202	0.783161	0.969507
mnb	3371.902	0.17293	0.628898	0.274973	0.783337	0.969507
lsn	524.7962	0.442681	1.611259	0.274743	0.783514	0.969507
CG32026	754.6312	-0.35573	1.296749	-0.27432	0.783838	0.969649
frj	4064.643	-0.17314	0.632325	-0.27381	0.784231	0.969696
CG6357	8103.11	-0.13357	0.48782	-0.2738	0.784238	0.969696
ana	12802.76	-0.13834	0.50497	-0.27395	0.784124	0.969696
Rack1	13510.95	-0.12242	0.447854	-0.27335	0.784587	0.969816
CG10286	524.0791	-0.34992	1.280814	-0.2732	0.784698	0.969816
CG5532	3131.773	0.189756	0.694047	0.273405	0.784542	0.969816
hwt	6486.63	-0.14057	0.51484	-0.27304	0.784819	0.969816
CG32425	2526.03	-0.20728	0.759839	-0.2728	0.785009	0.969902
Rpn10	3496.023	0.253807	0.932919	0.272056	0.785579	0.969996
CG9253	1004.11	0.354157	1.304577	0.271472	0.786028	0.969996
Kcmf1	2929.473	-0.18009	0.660803	-0.27253	0.785218	0.969996

DCTN6-p2	353.8972	-0.44025	1.621977	-0.27143	0.78606	0.969996
lncRNA:CR	384.585	0.465964	1.715688	0.27159	0.785937	0.969996
Patr-1	1042.778	0.260686	0.96094	0.271283	0.786174	0.969996
Hsp83	20797.45	-0.11068	0.407877	-0.27135	0.786122	0.969996
CG11593	1497.826	-0.24995	0.918469	-0.27214	0.785515	0.969996
Abp1	1332.477	0.259874	0.958501	0.271126	0.786294	0.969996
bc10	1819.802	-0.22235	0.816327	-0.27238	0.785329	0.969996
galectin	6088.387	-0.15731	0.586277	-0.26832	0.788454	0.970229
Alg11	3615.587	-0.21772	0.809517	-0.26895	0.787972	0.970229
CG7369	1269.241	0.281096	1.046834	0.26852	0.788299	0.970229
Snr1	1641.372	-0.37212	1.390625	-0.26759	0.789014	0.970229
CG9536	1919.873	0.244909	0.920821	0.265968	0.790263	0.970229
CG33303	1862.198	0.228557	0.858308	0.266288	0.790017	0.970229
Trax	545.1136	-0.4586	1.708805	-0.26837	0.788411	0.970229
Srp14	580.9267	-0.35422	1.314031	-0.26957	0.787495	0.970229
Ythdf	1911.054	-0.19882	0.747515	-0.26597	0.790263	0.970229
DIP-gamm	1317.309	0.260176	0.970464	0.268094	0.788627	0.970229
CG8079	690.3128	-0.33912	1.26272	-0.26856	0.788268	0.970229
Zip102B	3240.102	0.167206	0.628938	0.265854	0.790351	0.970229
tacc	7731.377	-0.15304	0.571716	-0.26768	0.788944	0.970229
CG10863	2957.909	-0.19088	0.711075	-0.26844	0.788358	0.970229
Mtl	7408.846	-0.18548	0.69688	-0.26616	0.790118	0.970229
CG40160	717.1885	0.555133	2.076395	0.267354	0.789197	0.970229
lncRNA:CR	114.6449	0.839315	3.138231	0.267448	0.789124	0.970229
mub	18355.5	-0.11329	0.425804	-0.26605	0.790198	0.970229
CG42784	2800.913	0.17596	0.658391	0.267257	0.789271	0.970229
Nup214	114.3346	0.8187	3.028516	0.27033	0.786906	0.970229
Golgin245	2187.722	0.231393	0.860056	0.269044	0.787896	0.970229
ND-19	8078.265	0.132339	0.489094	0.27058	0.786714	0.970229
Pof	507.2788	-0.64647	2.41129	-0.2681	0.78862	0.970229
lncRNA:CR	1185.354	0.251828	0.93453	0.26947	0.787568	0.970229
lncRNA:CR	237.157	0.514934	1.930202	0.266777	0.789641	0.970229
RpL31	13723.7	0.121813	0.45759	0.266205	0.790081	0.970229
CG1271	80.99655	1.027366	3.858846	0.266237	0.790057	0.970229
CG15814	1891.334	-0.23248	0.860259	-0.27025	0.786971	0.970229
scyl	10867.27	-0.12028	0.449716	-0.26746	0.789118	0.970229
velo	1043.606	0.288718	1.079866	0.267365	0.789188	0.970229
GABPI	4281.786	-0.17661	0.656045	-0.2692	0.787777	0.970229
CG15385	2148.051	0.285021	1.063522	0.267997	0.788702	0.970229
CG5110	2119.329	-0.21334	0.804581	-0.26516	0.790888	0.970258
CG7029	16118.63	-0.1126	0.425608	-0.26457	0.791341	0.970258
CG5001	682.6446	0.428102	1.617171	0.264723	0.791223	0.970258
CG8155	1372.327	0.396747	1.498494	0.264764	0.791191	0.970258
jeb	17864.47	0.123685	0.466973	0.264865	0.791113	0.970258
Bet5	1456.41	-0.21923	0.82807	-0.26475	0.791204	0.970258
sov	4494.083	-0.1528	0.577236	-0.26472	0.791227	0.970258
CG32407	3631.23	-0.19712	0.743003	-0.2653	0.790782	0.970258
CG5254	3380.187	0.19398	0.734092	0.264246	0.791591	0.970346
fne	35736.66	0.101925	0.386302	0.263848	0.791897	0.970346
asRNA:CR4	136.8358	0.580192	2.197538	0.264019	0.791765	0.970346

l(2)k05819	1214.877	0.402136	1.523488	0.263957	0.791813	0.970346
CG4004	1536.833	0.276768	1.049858	0.263625	0.792069	0.970409
CG15877	83.70146	-0.8952	3.403857	-0.263	0.792553	0.970706
hob	284.6451	-0.55652	2.115921	-0.26301	0.79254	0.970706
Atg13	638.3634	0.328177	1.250963	0.262339	0.79306	0.971178
CG14817	2452.494	-0.18171	0.701571	-0.259	0.795632	0.971659
pcx	1052.766	-0.48469	1.865789	-0.25978	0.795036	0.971659
CG5381	1168.274	-0.3126	1.201797	-0.26011	0.794778	0.971659
CG9338	306.706	-0.61273	2.349485	-0.26079	0.794252	0.971659
Taf12	2557.454	-0.18565	0.716616	-0.25907	0.795581	0.971659
Smurf	1699.794	0.206281	0.790525	0.260941	0.794138	0.971659
APC10	257.929	-0.63857	2.462093	-0.25936	0.795358	0.971659
ACC	2693.867	0.206452	0.792017	0.260666	0.79435	0.971659
CG14982	3949.123	-0.18588	0.717025	-0.25924	0.79545	0.971659
l(2)05714	162.8606	-0.615	2.366974	-0.25983	0.794998	0.971659
Dad1	1434.915	0.220258	0.842919	0.261304	0.793858	0.971659
betaGlu	673.6366	-0.44381	1.706033	-0.26014	0.794756	0.971659
msn	3039.644	-0.2128	0.820972	-0.2592	0.79548	0.971659
Chc	6486.468	0.149323	0.572962	0.260616	0.794388	0.971659
kto	114.9294	-0.6448	2.481886	-0.2598	0.795017	0.971659
MED10	1412.822	0.222156	0.856342	0.259424	0.795308	0.971659
Nmt	242.7095	0.607357	2.322999	0.261454	0.793742	0.971659
CG8620	678.7403	0.296023	1.135595	0.260677	0.794342	0.971659
RtcB	671.8246	-0.48747	1.885369	-0.25855	0.795981	0.971937
Csk	3628.757	0.186167	0.72146	0.258042	0.796375	0.97227
wit	386.1375	-0.41151	1.596829	-0.2577	0.796636	0.972441
CG13369	660.2418	0.292442	1.149	0.254519	0.799095	0.972589
usp	913.5682	0.292698	1.194073	0.245126	0.806359	0.972589
Spt6	1104.262	0.352956	1.380436	0.255685	0.798194	0.972589
skd	9983.975	-0.11855	0.478594	-0.24771	0.804358	0.972589
Hcs	685.0453	-0.31597	1.281882	-0.24649	0.805306	0.972589
CG2519	5471.357	-0.15807	0.617253	-0.25608	0.797886	0.972589
Nmdar1	2254.898	-0.29546	1.213335	-0.24351	0.807608	0.972589
CG9171	1223.821	-0.30713	1.246615	-0.24637	0.805395	0.972589
Aatf	229.8468	0.517666	2.084126	0.248385	0.803837	0.972589
CG8353	276.6114	-0.44217	1.742928	-0.25369	0.799732	0.972589
CG9555	127.6052	-0.89724	3.68007	-0.24381	0.807378	0.972589
SamDC	1220.198	0.229776	0.928099	0.247577	0.804462	0.972589
Bug22	1332.089	0.35535	1.44699	0.245579	0.806008	0.972589
Nup107	350.0994	0.426225	1.687464	0.252583	0.800591	0.972589
Idgf2	1574.545	-0.37206	1.521938	-0.24446	0.806871	0.972589
CG31815	1661.412	0.250622	0.979344	0.255908	0.798022	0.972589
CG6115	3112.239	-0.16291	0.666409	-0.24446	0.806877	0.972589
RPA2	268.049	0.814149	3.350781	0.242973	0.808026	0.972589
mRpL47	1384.534	-0.27867	1.104985	-0.25219	0.800894	0.972589
CG5038	364.7627	0.401554	1.633746	0.245787	0.805847	0.972589
Arpc3A	3151.465	0.184806	0.721369	0.256187	0.797806	0.972589
CG17565	1412.078	-0.37536	1.49084	-0.25178	0.801211	0.972589
CG7183	689.3179	-0.30638	1.192266	-0.25697	0.797201	0.972589
CG5793	1559.592	-0.21216	0.83956	-0.2527	0.800501	0.972589

mRpL35	1688.791	0.206268	0.842822	0.244735	0.806662	0.972589
Sec13	2493.556	0.177741	0.699741	0.25401	0.799488	0.972589
CG5510	909.4875	0.436643	1.713306	0.254854	0.798836	0.972589
CG31089	144.957	0.574658	2.302787	0.249549	0.802936	0.972589
ro	317.6605	-0.56432	2.242338	-0.25167	0.801298	0.972589
CG31064	4049.212	0.284648	1.136079	0.250553	0.80216	0.972589
ATPsyngar	24022.12	0.099436	0.406999	0.244314	0.806987	0.972589
Obp57c	86.61895	0.953155	3.85075	0.247525	0.804502	0.972589
Ypel	842.2218	0.406929	1.584533	0.256813	0.797323	0.972589
Tango11	3799.183	-0.17419	0.685218	-0.2542	0.799338	0.972589
Got1	17764.09	0.163936	0.644516	0.254355	0.799221	0.972589
BEAF-32	2407.776	-0.18934	0.774752	-0.24439	0.806926	0.972589
CG18368	130.3121	0.794828	3.263161	0.243576	0.807559	0.972589
cid	223.5865	-0.73687	2.920498	-0.25231	0.8008	0.972589
ox	5170.305	0.148731	0.58327	0.254995	0.798727	0.972589
fdl	871.065	-0.41813	1.719495	-0.24317	0.807875	0.972589
Cam	43338.03	0.104501	0.416684	0.250792	0.801975	0.972589
EndoG	271.0643	0.640159	2.630206	0.243388	0.807705	0.972589
ERp60	8360.769	-0.14149	0.566084	-0.24995	0.802624	0.972589
CG13220	6493.873	-0.12676	0.520801	-0.2434	0.807694	0.972589
CG11825	150.4858	-0.9307	3.752033	-0.24805	0.804094	0.972589
CG12910	166.7843	0.735604	2.93123	0.250954	0.80185	0.972589
CAP	10783.76	0.124043	0.488456	0.25395	0.799534	0.972589
Map60	1230.013	0.243942	0.97514	0.250161	0.802463	0.972589
Prp38	718.5229	-0.36101	1.457565	-0.24768	0.804382	0.972589
CG10214	1356.779	0.261358	1.038066	0.251774	0.801216	0.972589
mRpL38	2319.581	-0.18518	0.73732	-0.25115	0.801697	0.972589
CG8939	175.8788	0.662443	2.615755	0.253251	0.800074	0.972589
jim	40610.95	-0.09602	0.385783	-0.24891	0.803432	0.972589
mle	725.7674	0.305876	1.248376	0.245019	0.806442	0.972589
nonA	6831.391	-0.13133	0.537019	-0.24455	0.806804	0.972589
His3.3A	3652.738	0.162302	0.636385	0.255038	0.798694	0.972589
Tab2	455.4144	-0.41229	1.696047	-0.24309	0.807935	0.972589
mRpS23	316.1498	-0.55304	2.176625	-0.25408	0.799431	0.972589
Larp7	135.0681	-0.74879	2.96772	-0.25231	0.8008	0.972589
NaPi-III	6623.643	-0.13322	0.545288	-0.24432	0.806984	0.972589
CSN8	642.1409	-0.31681	1.280409	-0.24743	0.804575	0.972589
RNASEK	23525.37	0.108496	0.426179	0.254579	0.799048	0.972589
Vha44	7836.851	-0.13406	0.544807	-0.24607	0.80563	0.972589
CG3253	1596.518	0.22326	0.883317	0.252752	0.80046	0.972589
Usp15-31	3556.427	-0.2014	0.827552	-0.24337	0.807721	0.972589
lncRNA:CR	763.563	-0.42917	1.704889	-0.25173	0.801252	0.972589
ens	8895.606	0.120626	0.47735	0.252699	0.800501	0.972589
lov	853.0752	-0.36948	1.503401	-0.24576	0.805867	0.972589
CG42259	195.4076	0.708437	2.836186	0.249785	0.802754	0.972589
pre-rRNA:(	282.1062	0.44418	1.814549	0.244788	0.80662	0.972589
lncRNA:CR	1336.41	0.21518	0.865025	0.248756	0.803549	0.972589
CG13807	652.4093	0.287065	1.150376	0.24954	0.802943	0.972589
CG10359	353.7353	-0.491	1.953711	-0.25132	0.801569	0.972589
Teh3	901.149	0.337064	1.333674	0.252733	0.800474	0.972589

lncRNA:ro)	47774.55	0.102104	0.402839	0.253462	0.799911	0.972589
Kmn1	206.6763	-0.90663	3.62529	-0.25009	0.802521	0.972589
regucalcin	1515.114	-0.24579	0.996834	-0.24657	0.805243	0.972589
be	1076.641	-0.33029	1.353104	-0.2441	0.807153	0.972589
CG14407	2484.995	0.186878	0.766196	0.243903	0.807306	0.972589
CG8611	222.0132	0.49441	1.932501	0.255839	0.798075	0.972589
CG14234	823.874	-0.31796	1.268923	-0.25058	0.802142	0.972589
Kap-alpha1	4300.977	-0.16132	0.648508	-0.24876	0.803545	0.972589
wnd	1805.383	-0.19709	0.807144	-0.24419	0.807086	0.972589
Crtc	1916.735	-0.19309	0.777288	-0.24841	0.803814	0.972589
QIL1	11098.11	-0.11672	0.460966	-0.2532	0.800113	0.972589
PDCD-5	475.9461	0.336007	1.319147	0.254716	0.798943	0.972589
IntS9	1463.311	0.270953	1.109665	0.244176	0.807095	0.972589
CrebA	10028.84	-0.1297	0.52584	-0.24665	0.805182	0.972589
Sox21b	67.13454	-0.89971	3.605084	-0.24957	0.802921	0.972589
Blos4	877.2263	0.288188	1.145919	0.251491	0.801435	0.972589
vsg	11880.99	-0.11967	0.484825	-0.24683	0.805036	0.972589
Pdp1	34037.63	0.1033	0.420661	0.245566	0.806018	0.972589
tbc	2835.245	0.206313	0.840744	0.245393	0.806152	0.972589
kirre	1316.283	0.233383	0.961751	0.242665	0.808265	0.972664
CG31550	3135.875	0.162397	0.669462	0.242579	0.808332	0.972664
Fkbp12	14725.32	-0.109	0.449698	-0.24239	0.808479	0.972696
Syx7	2346.615	-0.17042	0.703589	-0.24221	0.808619	0.972718
CG31886	611.9014	-0.43438	1.796966	-0.24173	0.808991	0.972718
Calr	6707.141	0.13526	0.559425	0.241784	0.808947	0.972718
Kap-alpha3	6817.558	0.122208	0.506311	0.24137	0.809269	0.972718
CG9399	3313.291	0.160136	0.663116	0.24149	0.809175	0.972718
CG10177	482.9596	-0.49502	2.046644	-0.24187	0.808883	0.972718
CG13623	1200.944	-0.28329	1.174161	-0.24127	0.809346	0.972718
Nap1	103.1095	-0.58474	2.429105	-0.24072	0.80977	0.973082
Las	822.9315	-0.35624	1.485023	-0.23989	0.810417	0.973168
CG4390	1616.776	-0.20352	0.847646	-0.2401	0.810252	0.973168
NaPi-T	340.6244	0.516145	2.148006	0.24029	0.810105	0.973168
ND-20	12874.44	0.104795	0.436906	0.239858	0.81044	0.973168
Upf3	248.4179	-0.74576	3.10928	-0.23985	0.810448	0.973168
tai	14190.32	-0.13288	0.55629	-0.23888	0.811201	0.973199
beat-Ib	5145.532	0.146421	0.612171	0.239183	0.810964	0.973199
Pngl	561.566	0.435056	1.818332	0.239261	0.810903	0.973199
amx	433.5802	0.491814	2.057806	0.238999	0.811106	0.973199
Ptpmeg	1425.822	-0.2396	1.002487	-0.23901	0.8111	0.973199
Mi-2	1660.767	0.210509	0.879142	0.239449	0.810758	0.973199
RpL13A	21919.32	-0.09926	0.415935	-0.23865	0.811378	0.973265
CG9773	1994.331	-0.18219	0.764236	-0.23839	0.811577	0.973359
kat-60L1	2006.103	0.188409	0.792117	0.237855	0.811993	0.973713
CG1239	495.5667	0.334147	1.422538	0.234895	0.81429	0.973801
CG9121	481.1126	0.328693	1.383982	0.237498	0.81227	0.973801
Ndf	2858.888	-0.1821	0.77025	-0.23642	0.813107	0.973801
Prosbeta3	2376.747	0.202094	0.862613	0.234281	0.814767	0.973801
beat-Vb	1534.311	-0.26758	1.135261	-0.2357	0.813667	0.973801
SIDL	513.7019	-0.32417	1.371521	-0.23636	0.813155	0.973801

Sulf1	2638.6	0.1932	0.820869	0.23536	0.813929	0.973801
AP-2sigma	8980.694	0.132687	0.560633	0.236674	0.81291	0.973801
Cdc16	134.4315	-0.85419	3.614601	-0.23632	0.813187	0.973801
Tsp97E	12808.32	0.114082	0.486548	0.234472	0.814619	0.973801
sktl	1035.409	-0.3686	1.572145	-0.23446	0.814628	0.973801
Fbl6	2858.908	-0.17169	0.726104	-0.23645	0.81308	0.973801
CG8046	207.0155	-0.7146	3.050022	-0.23429	0.814756	0.973801
alt	2571.919	-0.16552	0.706609	-0.23425	0.814793	0.973801
NijA	1103.777	0.233849	0.994563	0.235127	0.81411	0.973801
Caper	5634.888	0.136732	0.577957	0.236579	0.812984	0.973801
l(1)G0469	788.1886	-0.25328	1.077815	-0.235	0.814212	0.973801
CG42494	71.16537	0.905109	3.850934	0.235036	0.814181	0.973801
alc	7543.99	0.115881	0.491483	0.235778	0.813605	0.973801
lncRNA:CR	207.9631	0.611042	2.611203	0.234008	0.814979	0.973801
CG13012	1405.527	-0.22764	0.972343	-0.23411	0.814898	0.973801
Arp10	262.1076	-0.46204	1.952477	-0.23664	0.812935	0.973801
Prestin	466.4171	0.448639	1.909251	0.234982	0.814223	0.973801
CG7841	742.6256	0.262214	1.119895	0.234142	0.814875	0.973801
CG7139	1363.01	0.215813	0.923862	0.233599	0.815297	0.973853
Hex-A	13358.92	0.105695	0.452391	0.233638	0.815266	0.973853
lncRNA:CR	3176.774	-0.18934	0.810927	-0.23348	0.815387	0.973853
mei-P26	42998.52	0.096245	0.413072	0.232999	0.815762	0.97387
CG17985	391.4256	-0.33383	1.432406	-0.23306	0.815716	0.97387
CG6841	96.12012	-0.63411	2.72154	-0.233	0.815764	0.97387
CG1077	227.1059	0.770362	3.319784	0.232052	0.816498	0.973876
CG10053	100.5875	0.727711	3.134551	0.232158	0.816415	0.973876
CG11453	154.0886	0.718587	3.092297	0.232379	0.816243	0.973876
lolal	4716.355	-0.14971	0.643719	-0.23257	0.816091	0.973876
CG33710	146.2676	-0.76667	3.294372	-0.23272	0.815978	0.973876
ebd1	456.0908	-0.32282	1.390618	-0.23214	0.81643	0.973876
cib	3031.343	0.156159	0.676895	0.230699	0.817549	0.974007
Ada1-2	294.6762	0.417726	1.825113	0.228877	0.818965	0.974007
put	1494.856	0.281369	1.219358	0.230752	0.817508	0.974007
alpha-Man	609.0628	0.307965	1.346617	0.228695	0.819106	0.974007
ClpX	2225.634	0.165312	0.719909	0.22963	0.81838	0.974007
Apc2	83.94102	-0.72905	3.168358	-0.2301	0.81801	0.974007
CG18367	155.9686	0.752292	3.268016	0.230198	0.817938	0.974007
p115	80.88604	0.758816	3.318434	0.228667	0.819128	0.974007
JhI-26	96.23324	-0.58156	2.544762	-0.22853	0.819231	0.974007
sowah	1033.527	0.255006	1.112829	0.229151	0.818751	0.974007
CG13124	5914.039	0.156005	0.677383	0.230305	0.817854	0.974007
Ddr	1490.246	-0.19219	0.835153	-0.23013	0.817992	0.974007
Kif3C	1273.927	0.207897	0.900369	0.230902	0.817391	0.974007
Lcch3	2082.775	-0.17226	0.754489	-0.22832	0.819399	0.974007
lncRNA:CR	982.1557	0.231319	1.009604	0.229119	0.818777	0.974007
CG3638	3434.385	-0.14513	0.629154	-0.23067	0.817573	0.974007
SLO2	5126.565	0.132412	0.573103	0.231045	0.81728	0.974007
nonC	804.0155	-0.31356	1.371085	-0.22869	0.819109	0.974007
CG8944	736.3808	-0.25581	1.113514	-0.22973	0.818298	0.974007
Max	931.185	-0.22777	0.997057	-0.22845	0.819299	0.974007

fz2	2878.298	0.165255	0.713824	0.231507	0.816921	0.974007
CG15431	606.8031	0.292198	1.276186	0.228962	0.818899	0.974007
Reph	4368.86	-0.14007	0.609062	-0.22997	0.818114	0.974007
CG6660	353.6129	-0.59474	2.612178	-0.22768	0.819896	0.974251
CG15602	1911.896	0.321892	1.414373	0.227586	0.819968	0.974251
CG7772	1327.046	-0.19804	0.869509	-0.22776	0.819831	0.974251
Asph	1850.193	-0.19189	0.84513	-0.22705	0.820383	0.974599
Dp1	2493.55	0.198535	0.87509	0.226874	0.820522	0.97462
CG15111	5783.176	0.135577	0.600188	0.22589	0.821287	0.975252
Amph	12115.05	0.114965	0.509021	0.225855	0.821314	0.975252
lncRNA:CR	153.1808	0.618784	2.743271	0.225564	0.82154	0.975252
Nf-YA	117.3714	-0.63429	2.81109	-0.22564	0.821483	0.975252
CG11099	2420.736	-0.16378	0.728103	-0.22495	0.822022	0.97568
Cyp4ac1	120.7966	-0.72635	3.251607	-0.22338	0.823239	0.97698
CG18540	71.92435	-0.85586	3.836663	-0.22307	0.823477	0.977118
rb	1485.589	-0.24358	1.09461	-0.22252	0.823907	0.977246
Syp	29760.11	0.095862	0.43212	0.221842	0.824437	0.977246
CG30094	416.4272	0.35077	1.574841	0.222733	0.823743	0.977246
28SrRNA-F	105.0742	0.533571	2.402528	0.222088	0.824246	0.977246
CG46448	5777.813	0.181583	0.817027	0.222249	0.82412	0.977246
Nrt	4114.058	-0.1494	0.673127	-0.22195	0.824357	0.977246
CG6418	717.9133	0.381479	1.715633	0.222355	0.824038	0.977246
cbt	4385.378	0.143109	0.645932	0.221554	0.824661	0.977367
lncRNA:CR	134.5743	0.765794	3.461812	0.221212	0.824927	0.977538
CG5068	3095.249	0.166912	0.755311	0.220984	0.825105	0.977604
mRpL50	2010.33	0.166438	0.756122	0.22012	0.825778	0.978257
MetRS-m	1032.243	-0.39508	1.802192	-0.21922	0.826475	0.978268
veli	2967.415	-0.14161	0.643871	-0.21993	0.825928	0.978268
rdo	1728.794	-0.25847	1.176828	-0.21963	0.826158	0.978268
wap	1292.461	0.2069	0.942683	0.21948	0.826276	0.978268
CG18809	1810.032	-0.21285	0.968521	-0.21976	0.826055	0.978268
CG15715	2571.15	-0.15735	0.717936	-0.21917	0.826519	0.978268
CG13337	69.75943	-0.83772	3.834054	-0.21849	0.827044	0.978606
CG8839	3056.791	-0.14597	0.668069	-0.21849	0.827047	0.978606
CG6329	24954.19	-0.09677	0.443569	-0.21817	0.827297	0.97862
CG42526	71.06923	-0.84318	3.864929	-0.21816	0.827303	0.97862
Eph	10510.73	-0.09896	0.454093	-0.21792	0.827491	0.978697
CG13527	107.7085	-0.83443	3.833781	-0.21765	0.827701	0.978802
PIP4K	9637.584	-0.10029	0.462058	-0.21704	0.828174	0.978951
CG42514	393.0896	-0.38358	1.765886	-0.21722	0.828039	0.978951
Zcchc7	958.6349	0.226888	1.045471	0.21702	0.828193	0.978951
Ssdp	5521.937	0.135425	0.624491	0.216857	0.82832	0.978957
Rbcn-3B	4280.406	0.137882	0.636907	0.216488	0.828608	0.979041
l(1)10Bb	2145.661	0.164687	0.760846	0.216453	0.828635	0.979041
Roc1a	3886.038	0.127128	0.588415	0.216052	0.828947	0.979098
eIF2Bepsil	142.3833	-0.61128	2.831026	-0.21592	0.829049	0.979098
ND-42	6334.078	-0.15132	0.700335	-0.21606	0.82894	0.979098
CG42764	443.6797	0.413816	1.918914	0.215651	0.82926	0.979202
CG7492	833.6684	0.36745	1.706299	0.215349	0.829495	0.979336
sws	3095.877	-0.14018	0.651891	-0.21504	0.829735	0.979475

CG4452	789.2493	-0.23348	1.086937	-0.2148	0.829921	0.979551
Chd64	9706.564	-0.11827	0.552158	-0.21419	0.830398	0.979969
Ubc2	681.4667	0.239216	1.121677	0.213266	0.831119	0.980007
mRpS22	1252.875	0.220368	1.034032	0.213115	0.831237	0.980007
form3	836.1032	-0.23961	1.121069	-0.21373	0.830758	0.980007
Ptx1	153.3054	0.657538	3.085466	0.213108	0.831243	0.980007
Ank2	22039.78	0.086308	0.405025	0.213094	0.831254	0.980007
bs	360.7828	0.443521	2.080639	0.213166	0.831198	0.980007
ABCB7	1237.421	-0.19322	0.906917	-0.21305	0.831284	0.980007
lncRNA:CR	254.9225	0.593415	2.78837	0.212818	0.831469	0.980081
Cdk4	658.2236	-0.29901	1.406599	-0.21258	0.831656	0.980111
Ubc10	1805.164	-0.18799	0.884765	-0.21247	0.831739	0.980111
Prosbeta1	2538.019	-0.14828	0.700139	-0.21179	0.832274	0.980454
RpL12	13642.73	0.102157	0.482308	0.211808	0.832257	0.980454
CG11878	248.9864	0.535825	2.536462	0.211249	0.832693	0.980659
SCaMC	6830.622	-0.13218	0.625434	-0.21134	0.83262	0.980659
Pis	8983.544	-0.10059	0.47859	-0.21019	0.83352	0.98149
yin	872.6426	0.287367	1.369875	0.209776	0.833843	0.981581
AlaRS-m	1029.876	-0.41238	1.965245	-0.20984	0.833794	0.981581
d4	2576.886	-0.1668	0.797064	-0.20927	0.834241	0.981766
gfzf	141.9836	0.678612	3.242897	0.209261	0.834244	0.981766
MED21	3305.154	0.129032	0.61754	0.208945	0.834491	0.981809
UK114	771.6636	-0.22333	1.069086	-0.2089	0.834526	0.981809
Chchd3	3228.695	-0.1342	0.643046	-0.20869	0.83469	0.981858
CG33635	1323.287	0.190242	0.913889	0.208167	0.835099	0.982195
RpL37a	33973.87	-0.08092	0.389058	-0.208	0.835233	0.982209
trn	1271.941	-0.20202	0.97268	-0.20769	0.835471	0.982341
Trf5	314.8622	0.456392	2.199071	0.207539	0.835589	0.982341
Socs44A	2184.061	0.191489	0.923568	0.207337	0.835747	0.982382
upSET	11816	-0.09893	0.479386	-0.20637	0.836505	0.983129
CG9775	4959.758	-0.11186	0.545169	-0.20518	0.837429	0.983172
me31B	5272.768	-0.18884	0.920878	-0.20506	0.837521	0.983172
CG9426	1444.792	0.189263	0.918254	0.206112	0.836703	0.983172
MBD-R2	940.6868	0.212899	1.038099	0.205086	0.837505	0.983172
CG14259	78.32509	0.790693	3.844888	0.205648	0.837066	0.983172
dj-1beta	1547.506	0.166635	0.811874	0.205247	0.837379	0.983172
wun2	5556.433	0.130929	0.63737	0.205422	0.837243	0.983172
CG46462	70.7263	-0.72856	3.551032	-0.20517	0.83744	0.983172
Rm62	13204.98	0.089186	0.438098	0.203575	0.838685	0.98324
Rab14	8066.48	-0.11656	0.570586	-0.20428	0.838133	0.98324
CG18766	1803.149	0.16258	0.801037	0.202962	0.839165	0.98324
Pdhb	8350.756	0.099376	0.485196	0.204815	0.837716	0.98324
tra	739.8706	0.227268	1.11124	0.204517	0.837949	0.98324
nrv2	93494.08	-0.10114	0.495103	-0.20427	0.838139	0.98324
Dpm3	2095.673	0.157069	0.771146	0.203683	0.838601	0.98324
brwl	896.8382	-0.42973	2.116117	-0.20308	0.839075	0.98324
l(2)k09022	260.2253	0.369612	1.812021	0.203978	0.838371	0.98324
Xxylt	3747.875	0.13051	0.642468	0.203139	0.839027	0.98324
asRNA:CR4	68.59508	0.78388	3.85461	0.203362	0.838852	0.98324
CG44242	4714.874	-0.11668	0.573832	-0.20334	0.838869	0.98324

asRNA:CR4	117.6064	-0.77803	3.833561	-0.20295	0.839172	0.98324
CG4360	994.8905	0.226008	1.11551	0.202605	0.839443	0.983249
CG14257	146.6679	-0.77434	3.836307	-0.20185	0.840037	0.983249
shf	336.9299	-0.45031	2.230382	-0.2019	0.839997	0.983249
CG42359	140.3182	0.615985	3.04703	0.202159	0.839792	0.983249
CG42672	3042.39	-0.16833	0.830265	-0.20274	0.839338	0.983249
bin3	4574.97	-0.12733	0.629409	-0.2023	0.839685	0.983249
Frl	1344.207	-0.21201	1.050204	-0.20187	0.840016	0.983249
Drp1	2099.895	0.162059	0.803773	0.201623	0.840211	0.983309
Pbgs	529.9634	-0.3119	1.54863	-0.2014	0.840384	0.983369
CG31642	198.3078	-0.70988	3.535388	-0.20079	0.840861	0.983599
ct	32943.88	-0.09644	0.480938	-0.20052	0.841071	0.983599
Ubr1	1816.394	-0.20059	0.999905	-0.20061	0.841003	0.983599
CG6961	251.935	0.433239	2.157833	0.200775	0.840874	0.983599
Hmu	2446.811	0.152871	0.764825	0.199876	0.841577	0.983904
Pmp70	1404.371	-0.17242	0.861976	-0.20003	0.841459	0.983904
CG11267	6622.815	0.10787	0.540348	0.19963	0.84177	0.983986
AANATL7	305.0909	-0.38783	1.95291	-0.19859	0.842583	0.984017
CG7694	905.5047	0.204947	1.031229	0.19874	0.842466	0.984017
Dh31-R	672.6482	-0.40879	2.056895	-0.19874	0.842464	0.984017
CG42594	737.9709	-0.22079	1.111823	-0.19859	0.842587	0.984017
Mmp1	3089.289	0.147185	0.741516	0.198492	0.84266	0.984017
Cdc37	1205.161	0.186791	0.937289	0.199289	0.842037	0.984017
CG32368	545.6863	-0.27812	1.398467	-0.19888	0.842358	0.984017
CG10075	990.037	0.205104	1.034095	0.198342	0.842777	0.984017
Tlk	4725.492	-0.11651	0.588861	-0.19786	0.843151	0.98431
CG18048	198.8484	0.47432	2.400003	0.197633	0.843332	0.984378
CG15160	257.8302	0.385179	1.951992	0.197326	0.843572	0.984515
Nak	5087.472	-0.12264	0.624638	-0.19633	0.844351	0.984746
mRpS24	179.8789	-0.38231	1.945001	-0.19656	0.844173	0.984746
RpL13	16772.8	0.087704	0.446811	0.19629	0.844383	0.984746
mt:tRNA:A	85.40259	-0.51476	2.621427	-0.19637	0.844324	0.984746
Cngl	14108.12	0.09303	0.47267	0.196817	0.843971	0.984746
egh	5425.419	-0.10755	0.550399	-0.1954	0.845078	0.985093
Atox1	4551.807	0.137381	0.705235	0.194802	0.845548	0.985093
Acbp1	1826.273	-0.15039	0.769309	-0.19549	0.845007	0.985093
CG31674	296.3822	-0.42233	2.168331	-0.19477	0.845573	0.985093
Ctr1A	2268.635	-0.14322	0.73419	-0.19507	0.84534	0.985093
PI31	2503.971	-0.16185	0.830601	-0.19486	0.845506	0.985093
lncRNA:CR	64.32886	-0.74842	3.834794	-0.19516	0.845264	0.985093
SCOT	103.9344	-0.5951	3.057223	-0.19466	0.845663	0.985093
CG14050	499.2616	-0.48025	2.491304	-0.19277	0.847138	0.985347
msl-1	913.117	-0.20215	1.049644	-0.19259	0.84728	0.985347
drl	896.8554	0.323211	1.665509	0.194061	0.846128	0.985347
Mapmodu	6307.186	0.115337	0.598736	0.192634	0.847246	0.985347
ari-2	1176.523	-0.22941	1.189941	-0.19279	0.847125	0.985347
KCNQ	656.6538	-0.23827	1.238737	-0.19235	0.847467	0.985347
14-3-3zeta	36762.8	-0.07769	0.402679	-0.19293	0.847011	0.985347
Stat92E	1936.038	-0.17749	0.915021	-0.19398	0.846194	0.985347
PlexA	19244.32	-0.08553	0.444788	-0.19229	0.847517	0.985347

Sec22	1894.591	0.193455	0.999997	0.193455	0.846602	0.985347
pre-lola-G	113.9461	-0.73663	3.833863	-0.19214	0.847635	0.985347
Pcf11	9826.343	0.10286	0.532436	0.193187	0.846812	0.985347
CG32485	3234.237	0.120214	0.625619	0.192153	0.847623	0.985347
CG4645	1300.011	-0.22469	1.166441	-0.19263	0.847246	0.985347
GstT3	799.4026	0.302527	1.575459	0.192025	0.847723	0.985347
Sin1	583.1408	0.409362	2.136129	0.191637	0.848026	0.985557
dwg	87.18679	-0.63467	3.333585	-0.19039	0.849006	0.985697
TrxT	119.6643	0.7312	3.83368	0.190731	0.848737	0.985697
CARPA	1088.885	0.287525	1.505218	0.191019	0.848511	0.985697
CR30279	72.00752	0.752398	3.951211	0.190422	0.848978	0.985697
CG41128	6679.887	0.096042	0.504093	0.190524	0.848899	0.985697
CG10384	1486.114	-0.27057	1.420012	-0.19054	0.848886	0.985697
CG10347	534.3419	0.370362	1.939151	0.190992	0.848532	0.985697
RhoL	839.0352	0.332737	1.759734	0.189084	0.850027	0.985708
CG31441	143.723	0.662595	3.496868	0.189482	0.849715	0.985708
Ppt1	291.3919	0.402775	2.135302	0.188627	0.850385	0.985708
CG4038	62.25393	0.677945	3.565441	0.190143	0.849197	0.985708
CG12384	7287.017	-0.09766	0.513893	-0.19004	0.849279	0.985708
Vang	232.1772	0.393422	2.076874	0.18943	0.849756	0.985708
CG3091	2596.033	-0.21969	1.159856	-0.18941	0.849768	0.985708
Vps45	584.689	-0.34557	1.834832	-0.18834	0.850612	0.985708
CG5569	2088.8	-0.13889	0.734817	-0.18901	0.850082	0.985708
asRNA:CR4	93.24062	-0.67922	3.57953	-0.18975	0.849503	0.985708
Gbeta13F	23954.76	0.075922	0.402567	0.188594	0.850411	0.985708
Rrp47	2021.629	-0.27859	1.475426	-0.18882	0.850233	0.985708
Hsp67Ba	89.61623	-0.6064	3.219567	-0.18835	0.850604	0.985708
mRpL14	1899.609	-0.15752	0.853256	-0.18461	0.853536	0.986018
CG18508	1449.398	0.155955	0.830319	0.187825	0.851014	0.986018
Trf	383.2775	-0.34617	1.881035	-0.18403	0.85399	0.986018
CG4968	1921.564	0.143384	0.769334	0.186374	0.852151	0.986018
CG10268	297.5112	-0.3507	1.897922	-0.18478	0.853399	0.986018
CG10038	757.0076	0.317572	1.734813	0.183058	0.854752	0.986018
mRpS21	326.0281	-0.29335	1.580232	-0.18564	0.852731	0.986018
smp-30	174.0951	0.449126	2.464326	0.182251	0.855386	0.986018
CG31213	64.85324	0.635098	3.417899	0.185815	0.85259	0.986018
PQBP1	982.7398	0.184836	1.002543	0.184367	0.853726	0.986018
CG10916	1306.833	-0.18267	0.994684	-0.18365	0.854291	0.986018
Rrp42	318.7628	-0.42555	2.280322	-0.18662	0.851959	0.986018
Rho1	8124.773	-0.09083	0.488398	-0.18597	0.852467	0.986018
AstC-R1	354.4318	-0.27557	1.509119	-0.1826	0.855109	0.986018
Nmda1	11458.69	-0.08828	0.483276	-0.18267	0.855054	0.986018
Ir41a	124.6447	0.704579	3.833785	0.183782	0.854185	0.986018
CG32547	131.1415	0.590994	3.185697	0.185515	0.852825	0.986018
Pink1	1678.609	0.172911	0.935638	0.184806	0.853381	0.986018
CG1024	72.70029	-0.6986	3.834242	-0.1822	0.855426	0.986018
CG1578	2930.471	-0.13085	0.713724	-0.18334	0.854532	0.986018
Zw10	290.3819	0.413433	2.22089	0.186156	0.852322	0.986018
CG34195	105.3001	-0.63029	3.419464	-0.18433	0.853758	0.986018
CG34355	751.8575	0.232496	1.248342	0.186244	0.852254	0.986018

CG42331	269.9834	-0.34361	1.879564	-0.18281	0.854945	0.986018
zld	631.9467	0.268391	1.450986	0.184972	0.853251	0.986018
Rbp6	50090.46	0.07479	0.403421	0.18539	0.852923	0.986018
CG12848	829.8916	0.196383	1.061016	0.185089	0.853159	0.986018
Hr4	327.5325	-0.34603	1.885934	-0.18348	0.854422	0.986018
rg	86.89347	-0.69899	3.835676	-0.18223	0.8554	0.986018
otk2	64.97449	-0.7015	3.835242	-0.18291	0.85487	0.986018
mRpL23	2830.584	0.121294	0.660513	0.183636	0.854299	0.986018
hec	264.4119	0.45856	2.503523	0.183166	0.854668	0.986018
sbm	2858.411	0.152159	0.832517	0.18277	0.854979	0.986018
CG32196	192.1696	-0.48969	2.63564	-0.1858	0.852604	0.986018
beg	364.3977	-0.42296	2.27504	-0.18591	0.852512	0.986018
fz	853.7399	-0.19074	1.038574	-0.18366	0.854283	0.986018
CG15456	2137.659	-0.13986	0.757345	-0.18467	0.853489	0.986018
COX5A	27630.81	0.078376	0.434786	0.180262	0.856947	0.986496
CG12321	460.6221	-0.36419	2.008236	-0.18135	0.856096	0.986496
CG3376	2459.295	0.159419	0.880723	0.181009	0.85636	0.986496
CG40439	638.2277	-0.20867	1.157475	-0.18028	0.856931	0.986496
ken	550.9689	0.230395	1.277011	0.180418	0.856825	0.986496
lncRNA:CR	200.8819	0.333942	1.847519	0.180752	0.856562	0.986496
CG2211	327.4135	0.390888	2.16522	0.18053	0.856736	0.986496
CG12093	1057.301	-0.18442	1.016844	-0.18136	0.856082	0.986496
CG12012	2646.748	0.135191	0.748781	0.180548	0.856722	0.986496
Slc45-1	532.4628	0.447839	2.486746	0.18009	0.857082	0.98651
Sema2b	1873.442	0.151369	0.843812	0.179387	0.857633	0.986752
Agpat1	831.7529	-0.28319	1.57672	-0.17961	0.857461	0.986752
CG14618	584.9313	0.352051	1.962898	0.179353	0.857661	0.986752
SMC5	820.0139	-0.20974	1.178457	-0.17798	0.858738	0.986796
elm	5734.819	0.09501	0.534361	0.177801	0.858879	0.986796
CG14641	1225.641	0.169976	0.961124	0.176851	0.859625	0.986796
CG13397	598.6891	-0.29831	1.686299	-0.1769	0.859587	0.986796
pxb	2724.783	0.199798	1.118056	0.178701	0.858172	0.986796
luna	4772.698	0.123085	0.689566	0.178497	0.858333	0.986796
mmd	8796.535	-0.08539	0.481754	-0.17724	0.85932	0.986796
CG42342	20763.76	0.080364	0.45455	0.1768	0.859666	0.986796
dpr21	12021.6	0.079423	0.446581	0.177848	0.858843	0.986796
asRNA:CR4	87.94271	-0.59253	3.313849	-0.17881	0.85809	0.986796
CG44838	9158.091	0.093238	0.522693	0.17838	0.858425	0.986796
lncRNA:CR	251.3423	-0.34256	1.93003	-0.17749	0.859123	0.986796
28SrRNA-F	67.03038	-0.51345	2.886467	-0.17788	0.858816	0.986796
lncRNA:CR	304.7737	0.418581	2.363528	0.1771	0.85943	0.986796
Gad1	11355.09	0.105609	0.595682	0.177291	0.85928	0.986796
toc	5540.668	-0.11907	0.6707	-0.17753	0.859092	0.986796
CG13694	246.3114	0.606981	3.436286	0.176639	0.859792	0.9868
maf-S	2141.51	-0.13203	0.748146	-0.17648	0.859919	0.986804
Madm	1212.79	0.191359	1.088793	0.175754	0.860487	0.986899
CG17991	200.4158	-0.54301	3.08975	-0.17575	0.860493	0.986899
Df31	3403.624	0.11721	0.666289	0.175914	0.860361	0.986899
RpL19	16361.64	0.085876	0.48734	0.176213	0.860127	0.986899
Rop	17354.39	-0.07617	0.434767	-0.1752	0.860924	0.987252

CG31100	1616.24	-0.15745	0.900648	-0.17482	0.861222	0.987449
su(Hw)	300.0793	0.327354	1.879037	0.174214	0.861697	0.987449
CG31313	172.5414	-0.50779	2.924533	-0.17363	0.862154	0.987449
Rpb7	362.9142	-0.32562	1.879549	-0.17324	0.862461	0.987449
Rab2	4551.61	-0.10509	0.604221	-0.17392	0.86193	0.987449
CG13428	81.219	-0.46968	2.698906	-0.17403	0.861846	0.987449
Pu	1790.224	0.136141	0.784338	0.173574	0.8622	0.987449
Uhg4	812.7021	0.18618	1.07242	0.173608	0.862174	0.987449
Pgk	8168.143	-0.0838	0.483768	-0.17322	0.862476	0.987449
Uhg3	1016.421	-0.1879	1.079007	-0.17414	0.861754	0.987449
lncRNA:CR	64.54478	-0.62143	3.590001	-0.1731	0.862573	0.987449
CG5541	207.8627	0.378325	2.183753	0.173246	0.862458	0.987449
CG3918	107.2819	0.571449	3.325515	0.171838	0.863565	0.9875
ORMDL	1538.426	0.146487	0.849703	0.172398	0.863125	0.9875
Fip1	1266.203	0.164909	0.957635	0.172204	0.863277	0.9875
mRpS18A	3055.021	0.111735	0.649013	0.172162	0.86331	0.9875
Bin1	1202.464	-0.15362	0.896972	-0.17126	0.864016	0.9875
Shark	145.8989	0.543196	3.144318	0.172755	0.862844	0.9875
porin	26213.55	-0.07117	0.414829	-0.17157	0.863773	0.9875
nocte	6420.909	-0.0994	0.581245	-0.17101	0.864216	0.9875
gas	158.6183	0.656985	3.83345	0.171382	0.863923	0.9875
pre-rRNA:(	199.1853	0.327731	1.911904	0.171416	0.863897	0.9875
CG9132	1635.104	0.143449	0.835197	0.171755	0.86363	0.9875
Usp32	1150.674	-0.24636	1.435812	-0.17158	0.863766	0.9875
CG1695	5775.081	-0.10018	0.58563	-0.17107	0.864171	0.9875
LamC	104.619	0.500438	2.933108	0.170617	0.864525	0.987571
CG7860	129.3092	-0.6233	3.652076	-0.17067	0.864483	0.987571
PH4alphaE	1258.04	0.164187	0.968721	0.169488	0.865412	0.988411
Rx	107.0353	-0.61293	3.618922	-0.16937	0.865506	0.988411
Mrp4	496.5041	0.252001	1.490649	0.169054	0.865754	0.988553
tos	81.21191	0.582423	3.462658	0.168201	0.866425	0.989071
CG43658	689.4383	0.20669	1.229094	0.168164	0.866454	0.989071
ND-B16.6	7348.974	0.089777	0.534467	0.167975	0.866603	0.9891
EndoGI	528.1745	-0.21154	1.265011	-0.16722	0.867195	0.989496
Bruce	5252.175	0.116385	0.696649	0.167065	0.867319	0.989496
Etf-QO	259.1985	0.347491	2.079889	0.167072	0.867313	0.989496
CG11248	644.2836	0.205592	1.233394	0.166688	0.867616	0.98958
CSN5	716.6741	-0.30636	1.838237	-0.16666	0.867639	0.98958
Mlc-c	2299.95	0.123435	0.741618	0.166441	0.86781	0.989634
CG45676	1053.734	0.185491	1.116409	0.16615	0.868039	0.989755
Scgalpha	1574.295	0.147146	0.886614	0.165964	0.868185	0.98978
eIF3b	3715.867	-0.0981	0.593219	-0.16537	0.868655	0.989975
CG10307	770.8143	0.245535	1.486717	0.165152	0.868824	0.989975
DAT	142.44	0.57094	3.44977	0.165501	0.86855	0.989975
CG32521	13107.09	-0.10468	0.633957	-0.16512	0.868849	0.989975
RpL6	15094.1	-0.08329	0.505363	-0.1648	0.869099	0.99001
COX7C	17138.91	-0.06899	0.418707	-0.16477	0.869127	0.99001
CG15657	79.1199	-0.64094	3.898843	-0.16439	0.869421	0.990205
SP1029	1113.517	-0.20166	1.228851	-0.1641	0.869651	0.990327
lncRNA:CR	167.6861	0.462088	2.820531	0.16383	0.869865	0.990429

CG31122	2893.669	0.132356	0.810753	0.163251	0.870321	0.990808
Npc2b	1473.389	-0.16491	1.011854	-0.16298	0.870537	0.990914
if	3059.008	-0.12645	0.777281	-0.16268	0.870771	0.991039
Cyp28a5	11392.76	-0.0907	0.559641	-0.16206	0.871257	0.991217
CG1360	208.6804	0.486314	3.001395	0.162029	0.871283	0.991217
CG13563	174.1058	0.427799	2.643992	0.161801	0.871463	0.991217
lncRNA:CR	171.167	-0.32418	2.001232	-0.16199	0.871314	0.991217
HP4	1252.871	-0.15827	0.97879	-0.1617	0.871545	0.991217
CG10702	144.4691	0.497647	3.089269	0.161089	0.872023	0.991476
CG31199	68.17882	0.617432	3.83952	0.16081	0.872243	0.991476
CngA	140.6707	0.345915	2.146194	0.161176	0.871955	0.991476
levy	29446.61	-0.0649	0.404045	-0.16062	0.87239	0.991476
CG8675	333.4154	-0.34676	2.156882	-0.16077	0.872275	0.991476
CG46301	498.5398	-0.20503	1.278404	-0.16038	0.872583	0.991555
RpL10	14946.88	0.07064	0.442685	0.159571	0.873219	0.991583
CG13365	2212.954	0.117061	0.754891	0.155071	0.876766	0.991583
CG3706	307.5044	0.253304	1.787419	0.141715	0.887305	0.991583
CG13760	2102.217	0.134467	0.861413	0.1561	0.875954	0.991583
CG10802	418.3823	-0.20972	1.535039	-0.13662	0.891329	0.991583
Parg	474.1553	0.362033	2.454414	0.147503	0.882735	0.991583
CG14270	1946.841	0.143125	1.038662	0.137797	0.890401	0.991583
Rpn13R	194.595	-0.39868	2.739288	-0.14554	0.884283	0.991583
RpL35	16224.56	0.072077	0.472692	0.152483	0.878806	0.991583
CG5274	450.7229	0.311457	2.08979	0.149038	0.881524	0.991583
CG10512	3141.926	-0.10041	0.705162	-0.14239	0.88677	0.991583
SAK	176.7823	0.280398	2.010618	0.139459	0.889088	0.991583
RpLP0	18841.2	0.059881	0.434831	0.13771	0.890469	0.991583
Atg17	2756.096	0.115454	0.773928	0.149179	0.881412	0.991583
Pgant5	2943.465	-0.09336	0.651643	-0.14327	0.886081	0.991583
Vps52	608.0301	-0.19884	1.268701	-0.15673	0.875457	0.991583
CG9117	97.60515	0.530377	3.579485	0.148171	0.882208	0.991583
Cpr	1722.884	-0.15865	1.11612	-0.14215	0.886965	0.991583
CG9498	234.1589	0.390979	2.861902	0.136615	0.891335	0.991583
COX5B	18739.53	-0.06643	0.438738	-0.15142	0.879645	0.991583
RpL7	23287.02	0.07673	0.506011	0.151637	0.879473	0.991583
Dpy-30L1	1786.474	-0.14711	1.048823	-0.14026	0.888454	0.991583
CG17124	2930.305	-0.08897	0.648875	-0.13711	0.89094	0.991583
CG5945	450.1044	0.360562	2.454535	0.146896	0.883214	0.991583
NimC2	68.72222	-0.54075	3.951225	-0.13686	0.891144	0.991583
Sidpn	430.7351	0.21716	1.377149	0.157688	0.874702	0.991583
l(2)37Bb	754.8768	-0.23982	1.69465	-0.14152	0.887462	0.991583
CG31800	513.522	-0.2071	1.419721	-0.14587	0.884023	0.991583
CG2656	220.4888	0.418466	2.802951	0.149295	0.881321	0.991583
IscU	29031.46	-0.06384	0.41665	-0.15323	0.878217	0.991583
RnpS1	371.3262	-0.28231	1.848934	-0.15269	0.878646	0.991583
CG9393	529.9706	-0.27383	1.924055	-0.14232	0.886829	0.991583
CG6254	609.144	0.202123	1.437784	0.140579	0.888202	0.991583
CG12279	741.165	-0.16214	1.09537	-0.14803	0.882322	0.991583
ND-23	12351.66	-0.06646	0.456434	-0.1456	0.884235	0.991583
CG3534	112.4778	-0.52258	3.361226	-0.15547	0.876449	0.991583

Trm7-32	1713.907	0.119283	0.840231	0.141965	0.887108	0.991583
Mur89F	98.72409	0.531628	3.869602	0.137386	0.890726	0.991583
Brf	240.7105	-0.29453	2.001517	-0.14715	0.883012	0.991583
Octalpha2l	7649.206	0.073348	0.485481	0.151084	0.87991	0.991583
loco	1244.325	0.135518	0.923606	0.146727	0.883348	0.991583
CG31145	3907.875	-0.08905	0.649516	-0.13711	0.890944	0.991583
CG5986	878.1321	-0.17068	1.109992	-0.15377	0.877795	0.991583
CG13630	342.063	-0.33774	2.191134	-0.15414	0.8775	0.991583
CG31510	1412.536	0.141245	0.977933	0.144432	0.885159	0.991583
LpR1	3309.923	-0.10308	0.731302	-0.14096	0.887905	0.991583
CG4849	165.5365	-0.3649	2.409656	-0.15143	0.879635	0.991583
eIF4E6	306.0611	-0.29608	1.850466	-0.16	0.872881	0.991583
CG33203	7556.348	-0.06892	0.48822	-0.14117	0.887738	0.991583
IntS11	618.0799	0.231035	1.454987	0.158788	0.873836	0.991583
ca	1056.805	-0.15692	0.9843	-0.15943	0.873332	0.991583
TkR99D	121.6463	0.410267	2.637051	0.155578	0.876366	0.991583
Sry-delta	862.4077	-0.19209	1.315491	-0.14602	0.883903	0.991583
CG1896	366.3091	-0.34341	2.292974	-0.14977	0.880949	0.991583
heph	26052.53	-0.05883	0.405665	-0.14502	0.884698	0.991583
CG11159	108.0194	0.563553	3.833835	0.146995	0.883136	0.991583
qkr54B	3753.904	0.089216	0.603723	0.147777	0.882519	0.991583
CG3198	5548.641	-0.10376	0.742387	-0.13977	0.888841	0.991583
CG9034	5030.602	0.08515	0.605513	0.140624	0.888167	0.991583
Khc	1407.569	0.147994	1.058049	0.139875	0.888759	0.991583
ATPCL	6231.981	0.080077	0.550309	0.145513	0.884306	0.991583
GPHR	2555.991	-0.11522	0.722269	-0.15952	0.873257	0.991583
fand	312.8577	0.307026	2.089588	0.146931	0.883186	0.991583
antr	302.1354	-0.33632	2.435515	-0.13809	0.890171	0.991583
Sln	1143.303	0.160331	1.029861	0.155683	0.876283	0.991583
CG18336	245.4509	-0.4398	2.781385	-0.15812	0.87436	0.991583
ND-B14	11671.04	0.066783	0.447617	0.149196	0.881399	0.991583
Prx2540-2	110.2676	0.471373	3.083001	0.152894	0.878482	0.991583
CG1407	682.1375	0.241297	1.745541	0.138236	0.890054	0.991583
CG11191	5815.176	-0.11795	0.782387	-0.15075	0.880171	0.991583
Tsp42En	107.638	-0.55485	3.50647	-0.15824	0.874271	0.991583
Adk3	5663.268	-0.08438	0.553767	-0.15238	0.87889	0.991583
Ephrin	5413.993	0.090371	0.628232	0.14385	0.885619	0.991583
mRpL13	761.731	-0.16648	1.153826	-0.14429	0.885274	0.991583
Aprt	169.4549	-0.46311	3.111949	-0.14882	0.881698	0.991583
CG30159	444.1092	-0.28131	1.874085	-0.15011	0.880681	0.991583
DIP1	7562.891	-0.0791	0.572828	-0.13809	0.890173	0.991583
GCS2alpha	12200.17	-0.07208	0.459381	-0.15691	0.875314	0.991583
Rab35	2863.165	0.089399	0.650266	0.13748	0.890651	0.991583
CG32576	2162.727	0.116523	0.742094	0.157019	0.87523	0.991583
Gyc88E	632.1522	0.162589	1.167208	0.139298	0.889215	0.991583
Mgat2	7578.399	0.075391	0.501719	0.150266	0.880555	0.991583
dlg1	13289.21	0.0629	0.445506	0.141187	0.887722	0.991583
Mtr3	362.8816	0.393877	2.567123	0.153431	0.878058	0.991583
Trl	445.7769	0.278605	1.8649	0.149394	0.881243	0.991583
Spt7	611.597	-0.29532	1.902974	-0.15519	0.876671	0.991583

CG34120	135.8097	0.53503	3.571903	0.149788	0.880932	0.991583
Nlg4	3379.551	0.102276	0.644782	0.158621	0.873967	0.991583
trv	6535.813	0.090272	0.581778	0.155166	0.876691	0.991583
CG34454	99.92102	-0.57468	3.951127	-0.14545	0.884358	0.991583
Alg-2	4206.096	-0.08702	0.600009	-0.14503	0.884685	0.991583
CG42361	4524.186	-0.08656	0.601886	-0.14381	0.885647	0.991583
Dys	2636.303	-0.11146	0.746176	-0.14938	0.881254	0.991583
CG42588	2370.2	0.137464	0.933794	0.14721	0.882966	0.991583
Lmpt	1284.911	-0.16168	1.170172	-0.13817	0.89011	0.991583
lncRNA:CR	158.3725	-0.44854	3.078842	-0.14569	0.88417	0.991583
CG42795	12952.33	0.082663	0.524678	0.15755	0.874811	0.991583
Gprk2	2210.768	-0.12451	0.814923	-0.15279	0.878563	0.991583
Rbp	16259.39	-0.07916	0.546992	-0.14472	0.884928	0.991583
lncRNA:CR	464.5007	0.293225	1.996949	0.146836	0.883261	0.991583
CG43222	783.726	-0.15294	1.103037	-0.13865	0.889727	0.991583
CG43324	11221.87	0.06857	0.483011	0.141963	0.887109	0.991583
CG13532	2266.23	0.141062	0.937684	0.150437	0.88042	0.991583
jbug	762.0994	0.163195	1.181723	0.138099	0.890162	0.991583
vir	223.7059	0.33686	2.367131	0.142307	0.886837	0.991583
Galphas	7877.408	0.078073	0.519709	0.150225	0.880587	0.991583
CG9380	151.3732	0.293411	2.092184	0.140241	0.888469	0.991583
MED30	423.686	0.249375	1.748	0.142663	0.886556	0.991583
lncRNA:CR	68.26583	0.598868	3.834367	0.156184	0.875888	0.991583
gpp	5484.659	0.084375	0.603494	0.139811	0.88881	0.991583
CG43902	1263.004	-0.14734	0.973365	-0.15137	0.879682	0.991583
lncRNA:CR	245.2738	0.390612	2.844491	0.137322	0.890776	0.991583
asRNA:CR4	65.25641	0.484499	3.048263	0.158943	0.873714	0.991583
lncRNA:CR	111.3185	-0.50183	3.496048	-0.14354	0.885861	0.991583
PIG-V	403.4251	-0.30061	2.201486	-0.13655	0.891387	0.991583
CG44422	1870.815	0.127587	0.835185	0.152765	0.878584	0.991583
Vamp7	3117.256	0.114049	0.819117	0.139234	0.889265	0.991583
lncRNA:TS:	117.1958	-0.52589	3.71453	-0.14158	0.887416	0.991583
pre-rRNA:(	74.04364	-0.52684	3.362005	-0.1567	0.875478	0.991583
Tmc	89.63613	0.595222	3.920835	0.15181	0.879337	0.991583
lola	26827.4	0.060266	0.431151	0.13978	0.888834	0.991583
caz	817.8847	-0.16677	1.057984	-0.15763	0.874746	0.991583
flz	177.2091	-0.48332	3.062456	-0.15782	0.874599	0.991583
Non2	2478.627	0.10093	0.725234	0.139168	0.889317	0.991583
Rdh	3102.194	-0.08625	0.629874	-0.13694	0.891081	0.991583
CG14997	1347.358	0.141109	0.903411	0.156196	0.875879	0.991583
dsh	238.4177	-0.26649	1.736676	-0.15345	0.878046	0.991583
Sk1	2091.656	0.119461	0.810252	0.147436	0.882788	0.991583
Usp7	3595.152	0.112687	0.724463	0.155546	0.876391	0.991583
Gmap	430.627	-0.21114	1.537087	-0.13737	0.890741	0.991583
Mvd	314.5302	-0.2948	1.969772	-0.14966	0.881033	0.991583
shi	24198.15	0.067296	0.461693	0.145759	0.884111	0.991583
Stim	3418.054	0.085811	0.627729	0.136701	0.891267	0.991583
SWIP	123.3909	-0.54882	3.648637	-0.15042	0.880436	0.991583
CalpC	176.6338	0.457036	2.879136	0.158741	0.873873	0.991583
Sap30	1070.135	-0.17957	1.171977	-0.15322	0.878228	0.991583

CG8010	339.4257	0.280258	1.830043	0.153143	0.878286	0.991583
THADA	222.7057	-0.32699	2.133844	-0.15324	0.878208	0.991583
GNBP1	217.955	0.288233	1.993491	0.144587	0.885037	0.991583
Sgf11	414.7797	-0.25831	1.885784	-0.13698	0.891049	0.991583
Arts	318.0524	-0.24153	1.647746	-0.14658	0.883464	0.991583
Sf3a2	710.3976	0.181346	1.257484	0.144214	0.885332	0.991583
RpS9	1703.102	0.117913	0.820789	0.143658	0.885771	0.991583
Hsp23	2334.468	0.110439	0.7575	0.145794	0.884084	0.991583
eIF4E1	15535.72	-0.07642	0.502198	-0.15216	0.879057	0.991583
exex	167.9754	-0.56083	3.668087	-0.15289	0.878482	0.991583
vito	76.9369	-0.52842	3.416013	-0.15469	0.877067	0.991583
Dd	3454.673	-0.09671	0.627183	-0.1542	0.877455	0.991583
CG9643	296.4149	0.357314	2.586468	0.138147	0.890124	0.991583
Bacc	25350.5	-0.05764	0.403551	-0.14283	0.886422	0.991583
RFeSP	21257.31	0.068776	0.437051	0.157365	0.874958	0.991583
CG31935	2955.296	-0.10315	0.654957	-0.1575	0.874853	0.991583
Ctr1B	345.9113	0.225077	1.653947	0.136085	0.891754	0.991602
lncRNA:CR	274.993	0.314676	2.31173	0.136121	0.891725	0.991602
kud	706.5866	-0.15047	1.107163	-0.1359	0.891898	0.991602
CG31689	667.1853	-0.16204	1.191126	-0.13604	0.891793	0.991602
Npc1a	2682.145	0.110208	0.814855	0.135249	0.892415	0.991627
CG31142	150.0416	-0.40656	2.999051	-0.13556	0.892167	0.991627
CG42495	267.5313	0.279217	2.063139	0.135336	0.892346	0.991627
shtd	467.2813	0.358287	2.645367	0.135439	0.892265	0.991627
RpL7A	14273.58	-0.06298	0.466539	-0.135	0.892615	0.991713
CG1116	852.4523	-0.15205	1.150584	-0.13215	0.894865	0.991751
stc	911.4923	-0.15156	1.125182	-0.1347	0.892849	0.991751
Ibf1	495.2381	0.235764	1.769297	0.133253	0.893993	0.991751
Rbf2	119.8487	-0.43438	3.299974	-0.13163	0.895277	0.991751
ATPsynD	25928.63	-0.05312	0.406558	-0.13065	0.896052	0.991751
CG11873	8048.455	-0.06405	0.49061	-0.13055	0.896134	0.991751
kune	2537.957	0.102136	0.772716	0.132178	0.894844	0.991751
CG16799	2381.956	-0.09373	0.721295	-0.12995	0.896604	0.991751
Fak	3208.594	0.08634	0.650803	0.132668	0.894456	0.991751
CG17982	434.327	-0.24197	1.8557	-0.13039	0.896256	0.991751
Bx42	1060.269	-0.12952	0.994537	-0.13023	0.896383	0.991751
CG7997	171.498	0.510729	3.813195	0.133937	0.893452	0.991751
S2P	856.1794	0.236374	1.779225	0.132852	0.89431	0.991751
CNT2	133.0804	-0.45258	3.436093	-0.13171	0.895211	0.991751
Duba	1345.484	-0.134	1.030968	-0.12998	0.896585	0.991751
CR18228	2653.172	0.089346	0.683266	0.130763	0.895963	0.991751
CG17168	1127.708	-0.14034	1.07057	-0.13109	0.895706	0.991751
Nlg3	5606.743	-0.07409	0.552	-0.13423	0.893223	0.991751
CG4218	116.8018	0.521498	3.879701	0.134417	0.893073	0.991751
Ndae1	2865.205	-0.08761	0.653316	-0.1341	0.893324	0.991751
RpS26	26193.5	0.055502	0.421319	0.131734	0.895194	0.991751
SERCA	6459.054	0.074667	0.560681	0.133173	0.894057	0.991751
CG3565	66.67185	-0.51536	3.948103	-0.13053	0.896145	0.991751
lncRNA:CR	222.8637	0.261871	2.009651	0.130307	0.896324	0.991751
CG44195	214.04	0.2714	2.063898	0.131499	0.895381	0.991751

asRNA:CR4	236.5355	0.388234	2.91617	0.133131	0.89409	0.991751
asRNA:CR4	63.23783	-0.51532	3.94812	-0.13052	0.896152	0.991751
asRNA:CR4	82.22047	-0.50336	3.834344	-0.13128	0.895556	0.991751
eEF5	7834.083	-0.06902	0.515759	-0.13382	0.893549	0.991751
Vav	3961.032	-0.0893	0.667591	-0.13377	0.893585	0.991751
CG6083	1455.026	-0.12654	0.955714	-0.1324	0.894668	0.991751
CG14341	540.7309	-0.23008	1.722851	-0.13354	0.893763	0.991751
LysRS	1005.899	0.132044	1.024231	0.12892	0.897421	0.991834
eEFSec	377.3471	-0.35649	2.76425	-0.12896	0.897386	0.991834
lncRNA:CR	98.34661	0.366638	2.840587	0.129071	0.897301	0.991834
Vha16-1	37653.15	0.055644	0.431396	0.128985	0.897369	0.991834
CG5577	283.2672	0.248444	1.922247	0.129247	0.897162	0.991834
CTCF	620.1841	0.222007	1.718606	0.129179	0.897216	0.991834
CG5131	1062.278	-0.19496	1.517765	-0.12845	0.89779	0.992106
Mybbp1A	181.338	0.283427	2.326523	0.121824	0.903038	0.99232
CG3719	2540.686	0.084612	0.714337	0.118448	0.905713	0.99232
CG11596	169.0961	-0.29063	2.356496	-0.12333	0.901843	0.99232
CG4199	1022.258	0.1235	1.26262	0.097812	0.922081	0.99232
CG2865	1622.513	-0.09031	0.854498	-0.10568	0.915835	0.99232
boi	124.0712	0.368036	3.835604	0.095952	0.923558	0.99232
fs(1)Yb	117.1318	-0.33262	3.101203	-0.10725	0.914587	0.99232
CG32767	7023.539	0.05867	0.534992	0.109665	0.912675	0.99232
Eaat2	13594.43	0.050186	0.55048	0.091168	0.927359	0.99232
Gsc	130.3772	0.400366	3.685893	0.108621	0.913503	0.99232
CG5955	4485.128	-0.06015	0.620045	-0.09702	0.922714	0.99232
ND-39	8985.963	0.057422	0.52642	0.10908	0.913139	0.99232
CG10508	7975.705	-0.0497	0.509557	-0.09754	0.922299	0.99232
IleRS	1070.633	0.137697	1.257251	0.109523	0.912788	0.99232
dsf	84.44041	-0.42142	3.951159	-0.10666	0.91506	0.99232
Tig	1049.839	-0.16807	1.358139	-0.12375	0.901513	0.99232
Hmgcl	93.47468	0.392083	3.837599	0.102169	0.918623	0.99232
Pcp	180.6485	0.407384	3.361381	0.121195	0.903536	0.99232
Rab30	948.6418	0.16091	1.559776	0.103162	0.917834	0.99232
CG5181	998.2097	-0.13618	1.068978	-0.1274	0.898626	0.99232
CG8460	661.9915	0.176894	1.40265	0.126114	0.899642	0.99232
PAPLA1	1766.763	-0.09505	0.861162	-0.11038	0.912109	0.99232
Rcd4	427.3817	-0.17446	2.024814	-0.08616	0.931338	0.99232
Tsp29Fb	141.3525	0.473734	3.833481	0.123578	0.901649	0.99232
eEF1delta	1416.78	-0.09167	0.851912	-0.10761	0.914305	0.99232
nmd	1314.844	-0.10866	0.92312	-0.11771	0.906301	0.99232
trk	114.4479	0.304269	2.769564	0.109862	0.912519	0.99232
kek2	3264.925	0.065915	0.68769	0.09585	0.92364	0.99232
crol	7658.37	0.048554	0.55212	0.087941	0.929924	0.99232
CG6785	77.79686	0.477521	3.859312	0.123732	0.901527	0.99232
eRF3	483.9759	-0.16502	1.790914	-0.09214	0.926583	0.99232
ND-B17	7472.059	0.049142	0.492307	0.09982	0.920487	0.99232
Tsen2	102.2739	0.461176	3.833871	0.12029	0.904254	0.99232
mEFTs	2171.101	-0.06849	0.761071	-0.08999	0.928295	0.99232
Arr1	107.1821	0.392141	3.833646	0.102289	0.918527	0.99232
Socs36E	2185.111	-0.06858	0.797473	-0.08599	0.931472	0.99232

tup	742.7892	-0.16393	1.937669	-0.0846	0.932577	0.99232
Cen	1897.724	-0.10778	1.088908	-0.09898	0.921155	0.99232
CG31677	95.86565	0.381178	3.833807	0.099425	0.9208	0.99232
alpha-Est1	101.2451	0.34785	3.833695	0.090735	0.927703	0.99232
e(y)2b	78.15769	0.36367	3.833899	0.094856	0.924429	0.99232
Scr	416.4562	0.158847	1.394658	0.113897	0.909319	0.99232
pyd3	1348.56	-0.13	1.115845	-0.1165	0.907253	0.99232
PolI	1199.69	-0.12326	1.348273	-0.09142	0.927157	0.99232
CG2747	3719.463	-0.06707	0.666193	-0.10068	0.919806	0.99232
CG11980	893.2793	0.111853	1.168023	0.095762	0.923709	0.99232
TrpRS	605.9005	-0.21556	2.059815	-0.10465	0.916653	0.99232
GstZ1	198.2183	-0.37801	3.153453	-0.11987	0.904585	0.99232
Unc-115a	1901.972	0.093986	0.79543	0.118157	0.905943	0.99232
eca	1190.39	0.095957	0.938621	0.102232	0.918572	0.99232
Tsp86D	1029.776	0.163201	1.40161	0.116438	0.907305	0.99232
Dph3	463.1007	0.138638	1.376801	0.100696	0.919792	0.99232
SdhC	24001.05	0.050502	0.425897	0.118578	0.90561	0.99232
DCAF12	127.9526	-0.35103	3.666808	-0.09573	0.923734	0.99232
Sccpdh2	860.6754	0.129985	1.242137	0.104646	0.916657	0.99232
Pp1-87B	8055.138	-0.04456	0.497984	-0.08948	0.928698	0.99232
CG5509	138.983	0.371223	3.667879	0.101209	0.919384	0.99232
Dtg	107.737	0.485416	3.940717	0.12318	0.901965	0.99232
CG5961	1712.996	-0.09973	1.046182	-0.09533	0.924054	0.99232
GILT1	1343.931	-0.08981	0.945377	-0.095	0.924319	0.99232
UQCR-C1	17924.72	0.046614	0.444466	0.104877	0.916473	0.99232
CG6966	4195.395	0.061683	0.704697	0.087532	0.930249	0.99232
CG6171	307.9114	-0.14418	1.598905	-0.09018	0.928148	0.99232
CG6218	5986.178	-0.05317	0.560235	-0.09491	0.924386	0.99232
Rh6	107.8051	0.377588	3.834557	0.09847	0.921559	0.99232
asun	616.1062	0.203697	1.877415	0.108498	0.9136	0.99232
CG6126	344.9233	-0.22237	2.483736	-0.08953	0.928661	0.99232
CCT3	332.1389	0.176954	1.522466	0.116229	0.907471	0.99232
CG3995	256.6352	-0.2434	2.444074	-0.09959	0.920671	0.99232
CG31360	1014.806	-0.10424	1.042727	-0.09996	0.920372	0.99232
CG5823	2299.24	-0.07844	0.822663	-0.09534	0.924041	0.99232
CG18600	124.4816	-0.34309	3.835414	-0.08945	0.928723	0.99232
P5cr	1414.569	-0.08018	0.900249	-0.08907	0.929027	0.99232
CG6195	1070.86	-0.18848	1.50804	-0.12498	0.900537	0.99232
GABA-B-R2	5452.175	0.06936	0.572846	0.121079	0.903628	0.99232
CG7054	169.0087	0.372286	3.087655	0.120572	0.90403	0.99232
CG31365	89.31969	0.395281	3.29034	0.120134	0.904377	0.99232
GILT2	1338.542	0.085295	0.939658	0.090772	0.927674	0.99232
CG5854	162.3446	0.337536	3.778936	0.08932	0.928827	0.99232
Rab7	9935.593	-0.04217	0.482397	-0.08742	0.930341	0.99232
Spase22-2	1769.385	-0.08688	0.806653	-0.10771	0.914226	0.99232
EMC6	3345.02	-0.06862	0.617715	-0.11109	0.911544	0.99232
dan	77.16645	-0.39393	3.833886	-0.10275	0.918162	0.99232
CG10562	768.5116	0.160643	1.620457	0.099135	0.921031	0.99232
Ets97D	110.8948	0.283075	3.247414	0.087169	0.930537	0.99232
CG5934	2657.373	0.070674	0.811516	0.087089	0.930601	0.99232

dmrt99B	121.4729	0.349086	3.727041	0.093663	0.925377	0.99232
Pcd	1390.611	0.073952	0.869612	0.08504	0.93223	0.99232
CG1542	301.1217	-0.25866	2.466777	-0.10486	0.916489	0.99232
CG11539	110.9449	0.362341	3.276139	0.1106	0.911933	0.99232
Ttc7	2952.536	-0.07965	0.727746	-0.10944	0.91285	0.99232
FAM21	613.4874	0.113272	1.330574	0.08513	0.932158	0.99232
CG13442	146.6763	0.230696	2.698093	0.085503	0.931861	0.99232
CG11180	374.8631	-0.19908	2.268965	-0.08774	0.930085	0.99232
Nnf1a	138.7285	-0.37649	3.181461	-0.11834	0.9058	0.99232
sano	785.4091	-0.14891	1.621649	-0.09182	0.926838	0.99232
CG4558	435.8574	-0.19454	1.758332	-0.11064	0.911904	0.99232
CG8300	386.266	-0.17738	1.826045	-0.09714	0.922615	0.99232
CG9657	28004.48	0.047216	0.516034	0.091499	0.927096	0.99232
Atg5	2189.291	-0.08004	0.718201	-0.11145	0.911262	0.99232
Smox	4811.115	-0.07068	0.595852	-0.11861	0.905582	0.99232
CG33181	4421.835	-0.05341	0.581223	-0.09189	0.926785	0.99232
Ogg1	64.01919	0.34772	3.838494	0.090588	0.92782	0.99232
CG11294	115.0496	0.364267	3.836062	0.094959	0.924348	0.99232
CG17754	713.735	-0.14664	1.532548	-0.09569	0.92377	0.99232
Naxe	206.4432	0.291722	2.940828	0.099197	0.920982	0.99232
Treh	30998.97	-0.06263	0.535409	-0.11698	0.90688	0.99232
Xbp1	10052.38	0.043485	0.477518	0.091066	0.92744	0.99232
Acox57D-c	254.4238	-0.37832	2.977078	-0.12708	0.898879	0.99232
CG15673	321.597	-0.34462	2.710751	-0.12713	0.898838	0.99232
TAF1C-like	298.9606	-0.19915	1.780801	-0.11183	0.910957	0.99232
Sod2	8351.821	-0.04515	0.504423	-0.08952	0.928671	0.99232
mrj	3378.478	-0.05856	0.636599	-0.092	0.926702	0.99232
CG8187	826.266	-0.11863	1.374255	-0.08632	0.93121	0.99232
CG10139	1214.886	-0.12578	1.157048	-0.10871	0.913435	0.99232
SMC2	204.7955	0.327631	3.243694	0.101006	0.919546	0.99232
Dh44-R1	419.0895	-0.24987	2.089887	-0.11956	0.904832	0.99232
CG13018	197.7088	0.20951	2.17995	0.096108	0.923435	0.99232
CG17739	2382.442	-0.09171	1.066689	-0.08598	0.931484	0.99232
SmD3	120.9734	0.455158	3.833727	0.118725	0.905493	0.99232
CG9005	4756.863	0.063992	0.576109	0.111076	0.911556	0.99232
Ggamma1	89279.56	0.032404	0.378056	0.085712	0.931696	0.99232
Gle1	966.9732	0.112249	1.194377	0.093981	0.925124	0.99232
Rpt1	1850.74	0.110926	1.01278	0.109526	0.912785	0.99232
CG1603	1252.958	-0.11415	1.108517	-0.10298	0.91798	0.99232
myo	9102.865	-0.05579	0.512284	-0.1089	0.913281	0.99232
CG9272	281.6576	0.355092	2.951913	0.120292	0.904252	0.99232
CG9451	2712.924	0.137438	1.416686	0.097013	0.922716	0.99232
dpr18	8296.601	-0.06513	0.586692	-0.11102	0.9116	0.99232
Cyp28d1	16267.93	-0.05465	0.544764	-0.10032	0.920088	0.99232
ref(2)P	7252.64	0.069547	0.797912	0.087161	0.930543	0.99232
7B2	27263.64	0.046542	0.407674	0.114164	0.909108	0.99232
CG14667	1557.452	-0.08505	0.964118	-0.08821	0.929709	0.99232
hig	26720.82	-0.03947	0.397488	-0.0993	0.920902	0.99232
cpx	111504.7	0.03825	0.413907	0.092413	0.92637	0.99232
CG4630	106.8774	-0.35828	3.839484	-0.09331	0.925654	0.99232

HDAC4	2831.116	-0.07116	0.685077	-0.10387	0.917274	0.99232
gw	18911.16	0.039384	0.439956	0.089519	0.928669	0.99232
CG2225	2424.07	-0.06441	0.738925	-0.08716	0.930542	0.99232
ND-ASHI	10234.87	-0.04927	0.458283	-0.10751	0.914384	0.99232
hth	31371.35	-0.04474	0.442745	-0.10104	0.919516	0.99232
Neb-cGP	14798.52	0.051814	0.447191	0.115865	0.90776	0.99232
CG4822	432.1501	0.229503	1.905257	0.120458	0.904121	0.99232
CG32202	634.8528	0.116661	1.161042	0.10048	0.919963	0.99232
CG4313	114.4496	-0.37389	3.079672	-0.12141	0.903369	0.99232
escl	274.1542	-0.20991	2.184014	-0.09611	0.923431	0.99232
disco	376.9242	0.161913	1.852655	0.087395	0.930358	0.99232
su(w[a])	7023.789	-0.04822	0.557607	-0.08648	0.931084	0.99232
CG9837	91.85609	-0.35183	3.834005	-0.09177	0.926884	0.99232
Adh	238.3714	0.186494	1.781275	0.104697	0.916616	0.99232
CG14408	2557.537	-0.08168	0.910965	-0.08966	0.928558	0.99232
lncRNA:CR	76.45461	0.390669	3.833925	0.101898	0.918838	0.99232
mt:tRNA:C	93.40189	-0.27932	2.543389	-0.10982	0.912549	0.99232
mt:tRNA:G	132.4312	0.28065	2.192356	0.128013	0.898139	0.99232
CG7878	1437.576	0.140484	1.211398	0.115968	0.907678	0.99232
CG34134	184.9517	-0.24422	2.480959	-0.09844	0.921585	0.99232
eIF4B	1070.595	0.09788	0.948063	0.103242	0.917771	0.99232
CG34213	139.1237	0.252696	2.772493	0.091144	0.927378	0.99232
smal	2805.048	0.063178	0.731192	0.086405	0.931145	0.99232
28SrRNA-F	4879.084	-0.05036	0.575446	-0.08751	0.930263	0.99232
snoRNA:M	70.18156	-0.3287	3.834617	-0.08572	0.931689	0.99232
Ino80	101.567	0.364214	3.23731	0.112505	0.910423	0.99232
l(3)L1231	4601.889	0.054296	0.592103	0.091701	0.926936	0.99232
Pcmt	2180.043	-0.08173	0.777857	-0.10507	0.916323	0.99232
stmA	9166.299	-0.07237	0.625324	-0.11574	0.907862	0.99232
puc	2060.784	-0.08685	0.82337	-0.10548	0.915997	0.99232
futsch	33542.28	0.047721	0.424532	0.112408	0.9105	0.99232
alpha-Man	4776.622	0.061464	0.59429	0.103424	0.917627	0.99232
corn	3898.02	0.073533	0.630695	0.116591	0.907184	0.99232
CG42450	10101.15	0.041417	0.468015	0.088496	0.929482	0.99232
CG42564	254.048	0.286034	2.529535	0.113078	0.909969	0.99232
Atg1	4261.731	-0.06498	0.578129	-0.11241	0.910502	0.99232
CG42656	170.7304	-0.3441	3.794599	-0.09068	0.927745	0.99232
ReepA	7442.676	0.053529	0.558227	0.095892	0.923607	0.99232
raskol	2458.858	0.06775	0.722799	0.093732	0.925322	0.99232
pdm3	4062.208	0.06067	0.653492	0.09284	0.926031	0.99232
lncRNA:CR	128.3869	0.406129	3.724274	0.109049	0.913164	0.99232
sdt	3726.019	0.075191	0.603388	0.124615	0.900828	0.99232
l(2)35Be	1664.143	0.108532	1.002317	0.108282	0.913772	0.99232
Sec23	3168.905	-0.0771	0.800922	-0.09627	0.923307	0.99232
VhaAC45	15242.14	0.050239	0.449018	0.111886	0.910914	0.99232
CG43191	281.5603	0.152294	1.620383	0.093986	0.92512	0.99232
CG43322	259.6856	0.278369	3.026791	0.091968	0.926723	0.99232
DnaJ-1	5562.403	0.067116	0.577503	0.116217	0.90748	0.99232
Mitf	5071.41	0.064976	0.556932	0.116668	0.907123	0.99232
fd59A	110.7243	0.374897	3.764254	0.099594	0.920667	0.99232

Cdk9	390.9311	0.161993	1.760156	0.092033	0.926672	0.99232
CG9849	1272.423	-0.09148	0.891981	-0.10256	0.918316	0.99232
CG16787	1010.707	0.126577	1.046263	0.12098	0.903707	0.99232
CG4806	309.5938	0.210318	1.723916	0.122	0.902899	0.99232
CG3776	2260.161	-0.07958	0.77553	-0.10262	0.918267	0.99232
key	1404.418	-0.11224	0.880164	-0.12752	0.898529	0.99232
wac	322.3683	-0.20575	1.98208	-0.10381	0.917323	0.99232
Hipk	16369.51	0.048682	0.430973	0.112959	0.910063	0.99232
Atac3	269.1749	-0.2581	3.002368	-0.08597	0.931494	0.99232
lncRNA:CR	96.08929	-0.34683	3.836068	-0.09041	0.927959	0.99232
lncRNA:CR	244.319	-0.20141	1.755089	-0.11476	0.908638	0.99232
lncRNA:CR	88.04109	-0.3272	3.738997	-0.08751	0.930266	0.99232
lncRNA:CR	173.8976	0.222141	2.594542	0.085619	0.931769	0.99232
asRNA:CR4	217.0328	0.260812	2.877179	0.090648	0.927772	0.99232
lncRNA:CR	132.4239	0.235929	2.794423	0.084429	0.932716	0.99232
lncRNA:CR	83.09523	0.367967	3.83442	0.095964	0.923549	0.99232
Dscam2	16654.81	0.039952	0.435292	0.091783	0.92687	0.99232
lncRNA:CR	133.1244	0.361301	3.127823	0.115512	0.908039	0.99232
lncRNA:CR	283.032	0.301599	2.654466	0.113619	0.90954	0.99232
lncRNA:CR	503.0334	0.123941	1.428415	0.086768	0.930856	0.99232
lncRNA:CR	79.71174	-0.38904	3.951298	-0.09846	0.921568	0.99232
mamo	22918.73	-0.0464	0.402505	-0.11527	0.908233	0.99232
CG32700	3081.849	0.053487	0.631386	0.084713	0.932489	0.99232
Glut4EF	23493.39	0.046515	0.406903	0.114314	0.908989	0.99232
rn	651.8444	-0.15688	1.842702	-0.08513	0.932155	0.99232
LanB2	138.0889	-0.32198	3.599507	-0.08945	0.928724	0.99232
28SrRNA-F	7308.997	0.057288	0.518629	0.110461	0.912044	0.99232
lncRNA:CR	72.46495	0.380922	3.835307	0.09932	0.920884	0.99232
lncRNA:CR	89.54874	-0.32473	3.834011	-0.0847	0.932503	0.99232
lncRNA:CR	1670.872	0.073845	0.80421	0.091823	0.926839	0.99232
lncRNA:CR	194.697	-0.21851	1.946762	-0.11224	0.910629	0.99232
Phm	8635.856	-0.06314	0.501247	-0.12597	0.899758	0.99232
Vha26	31560.03	0.039137	0.449384	0.08709	0.9306	0.99232
Letm1	2013.657	0.074985	0.792049	0.094672	0.924575	0.99232
tyn	870.9136	-0.14499	1.270364	-0.11413	0.909135	0.99232
para	44513.94	-0.03578	0.383263	-0.09334	0.925631	0.99232
scro	17191.85	-0.05454	0.52999	-0.1029	0.918042	0.99232
bor	1267.641	0.123874	1.147701	0.107932	0.914049	0.99232
PolD2	70.41339	0.410001	3.842933	0.10669	0.915035	0.99232
CG13937	4524.692	-0.05535	0.598629	-0.09246	0.926334	0.99232
Rap1	5313.338	0.0687	0.554872	0.123813	0.901463	0.99232
CG16986	911.3255	0.176352	1.546307	0.114047	0.9092	0.99232
CG33234	499.0198	0.186109	1.783168	0.10437	0.916876	0.99232
CG32276	5876.486	0.060405	0.536294	0.112634	0.910321	0.99232
CG1316	6877.557	0.069628	0.622177	0.111911	0.910894	0.99232
CG11357	1703.719	0.095243	0.801229	0.118871	0.905378	0.99232
DopEcR	14036.54	-0.04392	0.437351	-0.10043	0.920002	0.99232
sesB	138454.4	-0.04935	0.390345	-0.12644	0.899386	0.99232
Rpt3	2833.411	0.072437	0.689515	0.105054	0.916333	0.99232
Tango4	233.8955	-0.18888	1.799063	-0.10499	0.916385	0.99232

CG4318	126.0238	-0.39256	3.843428	-0.10214	0.918647	0.99232
CG1673	172.1847	-0.24536	2.023245	-0.12127	0.903478	0.99232
DNAlig4	222.4595	0.277897	2.771438	0.100272	0.920129	0.99232
ND-B18	3349.975	0.060224	0.620153	0.097112	0.922637	0.99232
CG12398	76.11111	0.37602	3.834854	0.098053	0.92189	0.99232
CG9281	1312.755	-0.09278	1.011503	-0.09173	0.926915	0.99232
MagR	3405.384	-0.07345	0.610242	-0.12037	0.904191	0.99232
CG8206	2657.824	0.083663	0.676957	0.123586	0.901643	0.99232
Rpc160	164.2868	-0.3238	2.807667	-0.11533	0.908187	0.99232
DENR	217.6675	-0.17269	1.834076	-0.09415	0.924986	0.99232
CG5162	194.108	0.367994	3.259481	0.1129	0.91011	0.99232
Arp8	252.0352	0.334168	3.015674	0.11081	0.911767	0.99232
CG32536	150.0401	-0.25779	2.716145	-0.09491	0.924387	0.99232
Grip84	1246.554	-0.09123	0.951941	-0.09584	0.923647	0.99232
mRpS14	1318.094	-0.09646	0.865029	-0.11151	0.911209	0.99232
Rpn1	1240.193	0.091217	0.951691	0.095848	0.923642	0.99232
CG7646	16597.35	0.048862	0.491821	0.099349	0.920861	0.99232
Oat	63.8807	0.352952	3.834216	0.092053	0.926656	0.99232
Gbs-76A	1670.21	-0.08612	0.85222	-0.10105	0.919508	0.99232
CG18135	6534.186	0.05614	0.576406	0.097396	0.922412	0.99232
HipHop	166.2941	0.365537	3.468983	0.105373	0.91608	0.99232
Dysb	174.1023	0.396561	3.374999	0.117499	0.906464	0.99232
anchor	4217.266	0.055111	0.611641	0.090104	0.928204	0.99232
a10	140.9046	-0.31247	3.608582	-0.08659	0.930997	0.99232
CG9674	15242.18	-0.05042	0.428894	-0.11756	0.906417	0.99232
Nplp3	122.7296	0.361947	3.377158	0.107175	0.91465	0.99232
CG5151	2991.764	-0.07635	0.659713	-0.11573	0.90787	0.99232
Cep135	75.50308	0.414057	3.842192	0.107766	0.914182	0.99232
bmm	1656.309	0.099562	1.016911	0.097906	0.922007	0.99232
Lmx1a	66.25414	-0.35059	3.834631	-0.09143	0.927152	0.99232
CG11597	673.7568	0.183689	1.598638	0.114903	0.908522	0.99232
Apt1	11046.84	0.060172	0.500964	0.120111	0.904395	0.99232
CG7607	12053.11	-0.04789	0.503905	-0.09504	0.924283	0.99232
CG7120	1333.65	-0.0942	0.857403	-0.10986	0.912518	0.99232
CG10274	192.1074	0.37257	3.284909	0.113419	0.909699	0.99232
CG13295	387.009	0.214333	1.98128	0.108179	0.913854	0.99232
Ntf-2	4173.075	0.065063	0.654669	0.099384	0.920834	0.99232
CG1812	3860.79	0.06537	0.737307	0.088661	0.929351	0.99232
capu	2794.614	-0.05893	0.657178	-0.08967	0.928553	0.99232
drm	138.0714	0.329585	3.261141	0.101064	0.919499	0.99232
alpha4GT1	1713.29	-0.12467	1.262037	-0.09879	0.921308	0.99232
Cwc25	407.4777	-0.16274	1.473849	-0.11042	0.912076	0.99232
Tnpo-SR	136.6145	0.423584	3.619822	0.117018	0.906846	0.99232
ND-B17.2	12705.13	0.037353	0.442497	0.084415	0.932727	0.99232
erm	477.8505	0.174924	1.390069	0.125838	0.89986	0.99232
Dop2R	5383.818	-0.05491	0.654981	-0.08384	0.933182	0.992674
temp	2803.372	-0.06545	0.810707	-0.08074	0.93565	0.993718
ninaD	205.2863	0.15366	1.887573	0.081406	0.935119	0.993718
tefu	247.3614	-0.19345	2.385832	-0.08108	0.935377	0.993718
PIG-L	1077.349	-0.14049	1.732377	-0.0811	0.935366	0.993718

Trxr-1	3677.165	-0.06671	0.816472	-0.0817	0.934884	0.993718
eIF4G1	23713.27	0.034723	0.429811	0.080787	0.935612	0.993718
CG32695	149.7026	-0.31231	3.833675	-0.08146	0.935073	0.993718
CG11406	62.47527	0.319995	3.950509	0.081001	0.935441	0.993718
rtv	134.6709	0.297731	3.633664	0.081937	0.934697	0.993718
asRNA:CR4	196.1819	0.211747	2.579156	0.082099	0.934568	0.993718
Hsc70Cb	4015.734	0.050892	0.617756	0.082382	0.934343	0.993718
CG3294	113.7179	-0.27666	3.387516	-0.08167	0.93491	0.993718
CG2652	159.5852	-0.24622	3.091257	-0.07965	0.936516	0.993807
CG5039	68.22504	-0.30318	3.841616	-0.07892	0.937096	0.993807
pygo	1511.863	-0.07885	0.998454	-0.07897	0.937057	0.993807
Act42A	1413.187	-0.13464	1.702769	-0.07907	0.936978	0.993807
cpa	1006.856	0.09527	1.198934	0.079462	0.936665	0.993807
CG10395	823.9088	0.093997	1.174377	0.08004	0.936205	0.993807
CG40485	85.68731	0.312499	3.950308	0.079107	0.936947	0.993807
CG34370	2766.334	0.052434	0.661825	0.079226	0.936853	0.993807
CG43843	99.92819	0.307623	3.845296	0.08	0.936237	0.993807
CG12531	4420.515	0.07544	0.950845	0.07934	0.936762	0.993807
GluRIA	3843.615	0.04693	0.589732	0.079578	0.936573	0.993807
lncRNA:CR	112.7251	0.297895	3.787114	0.07866	0.937303	0.993895
Pp2B-14D	4963.338	0.053154	0.678315	0.078361	0.937541	0.994016
CG31855	1594.732	0.06939	0.889368	0.078022	0.937811	0.994122
lncRNA:CR	263.5734	0.247422	3.175153	0.077924	0.937888	0.994122
CG4404	2708.131	0.056811	0.732393	0.077569	0.938171	0.994159
Shal	2583.386	-0.05939	0.765339	-0.0776	0.938147	0.994159
Uhg5	2425.203	-0.0553	0.715269	-0.07732	0.938371	0.994207
gol	5971.232	0.044039	0.570449	0.0772	0.938464	0.994207
lncRNA:ro)	207945.6	-0.02782	0.368101	-0.07557	0.939763	0.994254
RpLP1	18897.86	0.037241	0.486187	0.076598	0.938943	0.994254
mRpS18B	656.2128	-0.1457	1.922174	-0.0758	0.93958	0.994254
P58IPK	1295.181	-0.07021	0.931134	-0.0754	0.939893	0.994254
TFAM	607.9942	-0.09367	1.231924	-0.07604	0.939391	0.994254
CG8321	2106.165	0.060667	0.80788	0.075094	0.94014	0.994254
Crk	9763.342	-0.03621	0.483027	-0.07497	0.940242	0.994254
pros	35578.59	-0.03348	0.444752	-0.07527	0.940002	0.994254
Ac3	5136.986	0.050145	0.6604	0.075931	0.939474	0.994254
Rb97D	3330.663	0.059498	0.784357	0.075856	0.939534	0.994254
CG34039	248.1529	0.178927	2.375623	0.075318	0.939962	0.994254
Gprk1	11612.81	0.036879	0.479454	0.076919	0.938688	0.994254
CG9932	15726.35	0.037448	0.496571	0.075414	0.939885	0.994254
Myt1	618.543	-0.12422	1.627394	-0.07633	0.939155	0.994254
Pfdn1	815.3424	0.085122	1.145694	0.074297	0.940774	0.994385
gce	4459.786	0.047291	0.637471	0.074186	0.940862	0.994385
CG4101	120.8104	0.169222	2.280532	0.074203	0.940849	0.994385
Taf10	2803.55	-0.05711	0.766354	-0.07453	0.940591	0.994385
Mgat3	679.6658	0.140948	1.904153	0.074021	0.940994	0.994393
CR31032	610.0655	-0.16386	2.225682	-0.07362	0.941312	0.994598
kraken	615.645	-0.12987	1.768946	-0.07341	0.941476	0.994641
Prosalpha3	1957.658	0.056431	0.770462	0.073243	0.941613	0.994654
lbm	220.4858	-0.16436	2.261838	-0.07267	0.942072	0.994698

qkr58E-3	6061.282	-0.03803	0.52107	-0.07298	0.941819	0.994698
28SrRNA-F	778.9576	-0.07747	1.067536	-0.07257	0.94215	0.994698
CG1146	1351.451	-0.06525	0.897895	-0.07266	0.942073	0.994698
CG17328	265.4141	-0.16287	2.280733	-0.07141	0.943069	0.994948
CG31626	86.44298	0.268526	3.727266	0.072044	0.942567	0.994948
Cirl	15424.5	0.037595	0.523542	0.071808	0.942755	0.994948
pnut	3319.411	0.044673	0.623527	0.071646	0.942884	0.994948
Coa7	2876.462	0.04636	0.649885	0.071336	0.94313	0.994948
CG17261	938.9018	-0.09769	1.361158	-0.07177	0.942786	0.994948
Coa8	2119.052	-0.04717	0.732624	-0.06439	0.948664	0.994965
tko	2700.793	-0.04781	0.723064	-0.06613	0.947278	0.994965
CG3108	94.23146	0.19818	3.286833	0.060295	0.95192	0.994965
Spp	1609.45	-0.07187	1.113552	-0.06454	0.948542	0.994965
ND-15	6857.245	-0.03026	0.527148	-0.0574	0.954225	0.994965
Pitslre	9643.363	-0.02834	0.485695	-0.05836	0.953464	0.994965
CG32432	6301.457	0.031536	0.548426	0.057503	0.954145	0.994965
CG33056	2094.123	-0.04964	0.752292	-0.06598	0.947395	0.994965
CG33054	132.8194	-0.1955	2.793297	-0.06999	0.944203	0.994965
CG7407	3162.369	-0.04748	0.697294	-0.06809	0.945717	0.994965
CG14650	1708.269	0.070489	1.166193	0.060444	0.951802	0.994965
cl	1898.482	-0.04926	0.85957	-0.0573	0.954304	0.994965
CG11029	171.862	0.209773	3.224916	0.065048	0.948136	0.994965
TTLL3B	103.6165	0.259606	3.841373	0.067581	0.946119	0.994965
MME1	104.4091	0.269741	3.84983	0.070066	0.944141	0.994965
CG15141	1573.8	-0.05756	0.851888	-0.06757	0.946126	0.994965
CG11035	637.0524	0.110613	1.843351	0.060006	0.952151	0.994965
CG8507	2348.088	-0.07703	1.323621	-0.0582	0.953589	0.994965
ninaG	971.7853	-0.08534	1.361766	-0.06267	0.950031	0.994965
yrt	1132.873	-0.05462	0.929964	-0.05874	0.953162	0.994965
Nup43	418.1251	-0.1574	2.411441	-0.06527	0.947958	0.994965
Gos28	1239.856	0.060313	0.923655	0.065298	0.947937	0.994965
mdlc	129.6168	-0.19813	3.097358	-0.06397	0.948996	0.994965
Pi3K92E	2661.014	-0.04955	0.785497	-0.06308	0.9497	0.994965
hdly	520.2199	0.109193	1.701946	0.064158	0.948844	0.994965
SKIP	7419.624	-0.0325	0.538036	-0.0604	0.951836	0.994965
CG11791	4977.577	-0.03281	0.566946	-0.05787	0.953851	0.994965
Stt3B	1565.332	0.103761	1.56112	0.066466	0.947007	0.994965
CG12290	2037.968	0.044808	0.736494	0.060839	0.951487	0.994965
Hrb98DE	6366.078	0.036342	0.546971	0.066443	0.947025	0.994965
CG15535	196.3422	0.146533	2.418015	0.060601	0.951677	0.994965
Prosalpha5	1933.718	0.06152	0.991383	0.062054	0.950519	0.994965
RpL17	10194.53	0.035884	0.508471	0.070572	0.943738	0.994965
CG9650	2545.346	0.048502	0.716334	0.067709	0.946018	0.994965
mahe	8020.723	-0.03239	0.535995	-0.06043	0.951814	0.994965
CG10555	1362.387	0.060556	1.026117	0.059014	0.952941	0.994965
Dsor1	1067.818	-0.08663	1.475794	-0.0587	0.953192	0.994965
insc	72.16912	0.258704	3.943939	0.065595	0.9477	0.994965
Sara	3103.344	-0.0496	0.713348	-0.06954	0.944563	0.994965
eEF1beta	5333.051	-0.03542	0.59869	-0.05916	0.952828	0.994965
CG7639	1274.492	-0.11433	1.616235	-0.07074	0.943604	0.994965

mspo	1825.469	0.056469	0.891063	0.063373	0.94947	0.994965
mam	2668.438	0.040975	0.706729	0.057979	0.953766	0.994965
CG17574	1488.481	0.057431	0.888997	0.064602	0.948491	0.994965
CG13229	111.7774	0.252176	3.834031	0.065773	0.947559	0.994965
CG1663	295.1462	-0.19674	3.287617	-0.05984	0.952281	0.994965
CG1909	13123.86	-0.02973	0.497151	-0.05981	0.952309	0.994965
CG32850	4165.967	0.036045	0.627767	0.057417	0.954213	0.994965
chb	2600.261	-0.04376	0.681578	-0.06421	0.948804	0.994965
mRpL30	1484.422	0.058425	0.879055	0.066463	0.947009	0.994965
exd	6927.001	-0.0323	0.496681	-0.06503	0.948148	0.994965
CG8892	662.2281	-0.10438	1.775927	-0.05878	0.95313	0.994965
slpr	3906.951	0.049393	0.719589	0.06864	0.945276	0.994965
Nipped-A	9970.498	-0.03174	0.466287	-0.06807	0.945728	0.994965
vn	186.7135	-0.18147	2.836901	-0.06397	0.948995	0.994965
HIP-R	354.6475	0.093823	1.629982	0.05756	0.954099	0.994965
CtBP	6483.511	-0.03556	0.529715	-0.06714	0.946472	0.994965
Marf	2566.069	-0.04028	0.687641	-0.05858	0.953288	0.994965
CG5758	27314.48	-0.02946	0.472577	-0.06234	0.950289	0.994965
Pp2A-29B	5069.449	0.045379	0.680711	0.066664	0.946849	0.994965
Sod3	22009.08	-0.0306	0.478218	-0.06398	0.948988	0.994965
Vkor	203.2995	0.172229	2.803652	0.06143	0.951017	0.994965
CG34189	101.3977	-0.24378	3.567508	-0.06833	0.94552	0.994965
bbg	80.2538	-0.2067	3.48714	-0.05927	0.952733	0.994965
ome	1168.401	-0.07181	1.145529	-0.06269	0.950017	0.994965
mtgo	6637.402	-0.03024	0.506258	-0.05973	0.952368	0.994965
Msp300	51062.2	0.024465	0.409902	0.059685	0.952406	0.994965
whd	2379.213	-0.06188	0.982079	-0.06301	0.94976	0.994965
AGO1	15203.62	-0.02827	0.432778	-0.06531	0.947924	0.994965
CG9890	131.1515	0.212109	3.192522	0.066439	0.947028	0.994965
Rpn8	1300.253	-0.07239	1.078192	-0.06714	0.946469	0.994965
emp	407.8485	-0.16227	2.339028	-0.06937	0.944692	0.994965
CG1233	497.6885	0.124215	1.856396	0.066912	0.946652	0.994965
Atf-2	201.6262	0.173736	2.500796	0.069472	0.944614	0.994965
FASN1	13518.92	0.027084	0.452554	0.059846	0.952278	0.994965
br	1649.741	-0.05128	0.85771	-0.05978	0.952328	0.994965
Sema5c	482.7145	0.083332	1.392006	0.059865	0.952263	0.994965
aqz	64747.26	0.022693	0.379793	0.059752	0.952353	0.994965
PAN3	10388.47	0.030178	0.473396	0.063748	0.949171	0.994965
CG14961	146.366	0.147156	2.509648	0.058636	0.953242	0.994965
CG14963	105.206	-0.26847	3.849479	-0.06974	0.9444	0.994965
Atg3	2905.643	0.04119	0.666238	0.061825	0.950702	0.994965
Strump	1249.622	-0.11735	1.697527	-0.06913	0.944888	0.994965
CG10713	907.6211	-0.0608	1.037085	-0.05863	0.953249	0.994965
CG7888	67446.69	-0.03352	0.51138	-0.06556	0.947732	0.994965
pst	1364.464	-0.06629	0.967449	-0.06852	0.945375	0.994965
mal	82.64799	-0.26955	3.95034	-0.06824	0.945598	0.994965
Vps53	696.2526	-0.09824	1.382201	-0.07107	0.943339	0.994965
CG3702	3907.055	0.043718	0.737431	0.059284	0.952726	0.994965
Wdr62	3467.262	-0.04176	0.659116	-0.06336	0.949481	0.994965
clu	1783.205	0.04902	0.858727	0.057084	0.954478	0.995012

Vps28	2477.271	0.039978	0.702161	0.056936	0.954596	0.995012
PpV	5539.259	0.032295	0.653269	0.049436	0.960572	0.995041
CG32428	1176.485	-0.05017	0.984533	-0.05096	0.959359	0.995041
Hr78	589.0728	0.075323	1.349529	0.055814	0.95549	0.995041
Csp	12052.66	0.025015	0.490204	0.051029	0.959302	0.995041
CG12173	1313.784	-0.04348	0.88126	-0.04934	0.960648	0.995041
spict	631.0513	0.094679	1.78216	0.053126	0.957632	0.995041
c(2)M	69.86793	-0.18342	3.460429	-0.053	0.957728	0.995041
Hasp	29292.13	-0.02268	0.44584	-0.05088	0.959423	0.995041
Dh44	82.52315	0.133914	2.709591	0.049422	0.960583	0.995041
Nup93-2	165.9303	-0.17936	3.291182	-0.0545	0.95654	0.995041
RpL27	9284.887	0.029281	0.571914	0.051198	0.959168	0.995041
jdp	19584.29	-0.02122	0.412951	-0.05138	0.959021	0.995041
wts	2858.589	-0.03405	0.651257	-0.05228	0.958304	0.995041
mRpL32	892.3108	-0.06983	1.335874	-0.05228	0.958309	0.995041
SCAP	2278.888	0.035114	0.712611	0.049276	0.9607	0.995041
swi2	135.8302	-0.11929	2.23712	-0.05332	0.957476	0.995041
l(1)G0193	3232.436	-0.03303	0.666965	-0.04953	0.9605	0.995041
CG10932	164.9929	0.174805	3.232498	0.054077	0.956874	0.995041
CG10795	1018.786	-0.08142	1.636056	-0.04976	0.960311	0.995041
twz	820.2648	-0.07243	1.381297	-0.05243	0.958183	0.995041
bug	749.6106	0.069156	1.375344	0.050283	0.959897	0.995041
fsd	290.0375	-0.10903	2.038188	-0.0535	0.957337	0.995041
Mtor	992.9823	-0.07781	1.437367	-0.05413	0.95683	0.995041
Tsp42Ej	16001.82	-0.02364	0.475054	-0.04977	0.960308	0.995041
Lamp1	11269.44	-0.02494	0.490907	-0.0508	0.959485	0.995041
CG4537	87.23716	0.171225	3.34165	0.05124	0.959135	0.995041
CHES-1-lik	2551.679	0.036978	0.702766	0.052617	0.958037	0.995041
GstD1	30023.1	0.024384	0.459751	0.053038	0.957702	0.995041
side-IV	1322.358	0.059014	1.076761	0.054807	0.956292	0.995041
CG34133	1492.653	0.062235	1.237235	0.050302	0.959882	0.995041
Prosbeta7	2086.1	-0.03838	0.771147	-0.04977	0.960305	0.995041
Ppcs	3072.613	0.035797	0.673935	0.053116	0.957639	0.995041
Mdh2	19842	0.023714	0.44395	0.053416	0.9574	0.995041
Skeletor	271.4584	-0.1236	2.249558	-0.05494	0.956183	0.995041
CG13551	5531.345	-0.03071	0.58213	-0.05275	0.957931	0.995041
CG3860	3728.079	0.039575	0.704309	0.056189	0.955191	0.995041
Vdup1	303.9643	-0.15691	2.797709	-0.05608	0.955275	0.995041
Eip93F	8673.085	-0.02589	0.493313	-0.05249	0.958138	0.995041
lncRNA:CR	304.7639	-0.11719	2.259356	-0.05187	0.958633	0.995041
asRNA:CR4	232.8226	-0.09602	1.753961	-0.05474	0.956344	0.995041
Sec5	857.8745	0.07	1.402676	0.049905	0.960198	0.995041
CG12091	4048.909	0.036729	0.671237	0.054719	0.956363	0.995041
CG13008	111.9169	0.183814	3.547283	0.051818	0.958674	0.995041
Ada3	1054.25	-0.05142	0.953217	-0.05394	0.956983	0.995041
CG18081	1519.929	0.056472	1.030038	0.054825	0.956278	0.995041
ND-13B	5515.45	0.030359	0.554328	0.054766	0.956325	0.995041
CalpB	3003.218	-0.03584	0.666356	-0.05378	0.957106	0.995041
Arl5	962.9202	0.057646	1.072164	0.053766	0.957121	0.995041
NTPase	2944.822	0.035678	0.670874	0.053182	0.957587	0.995041

RpL36A	13167.36	-0.02465	0.502697	-0.04904	0.960885	0.995105
NT5E-2	1650.781	0.040267	0.826634	0.048712	0.961149	0.99514
Nipped-B	20589.67	-0.02231	0.458184	-0.04869	0.961167	0.99514
CG5160	67.09987	-0.18587	3.834502	-0.04847	0.96134	0.995191
Naa40	149.0145	-0.18407	3.831723	-0.04804	0.961686	0.995292
Eaf6	447.9735	0.083731	1.739559	0.048133	0.96161	0.995292
Oatp30B	1317.333	-0.06109	1.286697	-0.04748	0.962129	0.995298
klg	6177.742	-0.02606	0.551834	-0.04722	0.962341	0.995298
Wsck	877.8291	-0.05402	1.143351	-0.04724	0.962318	0.995298
CG7849	921.0052	-0.08109	1.725885	-0.04698	0.962527	0.995298
lncRNA:CR	3580.981	0.028569	0.613067	0.046601	0.962831	0.995298
dpr9	4861.144	-0.02704	0.579502	-0.04666	0.962785	0.995298
lncRNA:CR	113.3845	-0.157	3.378108	-0.04647	0.962932	0.995298
Gabat	28363.75	-0.01852	0.397456	-0.04659	0.962838	0.995298
CG8281	66.5963	-0.17899	3.835643	-0.04667	0.962779	0.995298
oaf	2199.768	0.03693	0.778801	0.047419	0.96218	0.995298
Men	4372.328	0.026535	0.597079	0.044441	0.964553	0.995731
lncRNA:CR	2540.535	0.038654	0.873165	0.044269	0.96469	0.995731
trp	75.21864	-0.17222	3.833946	-0.04492	0.964172	0.995731
SmydA-5	546.4073	-0.09872	2.231496	-0.04424	0.964715	0.995731
unc-119	4259.976	0.029905	0.668131	0.04476	0.964299	0.995731
shn	2980.299	0.040787	0.903609	0.045138	0.963997	0.995731
CG9062	283.4173	0.076867	1.720853	0.044668	0.964372	0.995731
RfC4	79.91044	-0.17029	3.792004	-0.04491	0.964181	0.995731
5-HT1B	1056.006	0.046069	1.021116	0.045116	0.964015	0.995731
stac	1287.493	-0.06619	1.494452	-0.04429	0.964671	0.995731
mRpL20	2185.082	0.040524	0.907739	0.044642	0.964392	0.995731
Edem2	1067.359	0.047798	1.10023	0.043444	0.965348	0.995744
Tmem131	673.953	0.05247	1.203987	0.043581	0.965239	0.995744
Hr3	858.4217	0.059027	1.345661	0.043864	0.965012	0.995744
CG34231	84.43264	-0.14255	3.272748	-0.04356	0.965259	0.995744
CG4577	33143	-0.01697	0.38793	-0.04374	0.965113	0.995744
y	117.8561	0.138852	3.253588	0.042677	0.965959	0.995766
fusl	958.3188	0.044521	1.047969	0.042483	0.966114	0.995766
GATAd	2186.15	-0.03455	0.810967	-0.04261	0.966013	0.995766
CG33230	326.7783	-0.10692	2.508342	-0.04262	0.966001	0.995766
Met	2283.211	0.030355	0.704954	0.043059	0.965655	0.995766
Prim1	89.95923	0.141074	3.278091	0.043035	0.965673	0.995766
Src42A	3918.503	0.024772	0.59744	0.041464	0.966926	0.996475
CG8860	66.82152	0.118721	2.888035	0.041108	0.96721	0.99664
CG6629	76.09114	0.15715	3.87183	0.040588	0.967624	0.996642
Inx7	142.327	0.156733	3.871707	0.040482	0.967709	0.996642
bun	30435.66	-0.01618	0.398968	-0.04055	0.967653	0.996642
Galt	853.3547	0.056762	1.397039	0.04063	0.967591	0.996642
CG15653	180.9958	-0.14962	3.751538	-0.03988	0.968187	0.997006
lncRNA:CR	72.45577	-0.13839	3.513657	-0.03939	0.968583	0.997196
flfl	3821.326	-0.02326	0.595943	-0.03903	0.968868	0.997196
Diap2	7738.899	0.02079	0.529549	0.03926	0.968683	0.997196
kis	25071.54	-0.01566	0.400602	-0.03908	0.968826	0.997196
Ufd4	7544.086	-0.02264	0.598347	-0.03784	0.969812	0.997855

CG12929	345.0622	-0.0852	2.263859	-0.03764	0.969979	0.997855
capt	1581.252	-0.03254	0.865396	-0.0376	0.970006	0.997855
CG43102	1808.216	0.034798	0.920222	0.037814	0.969836	0.997855
CG5339	354.6715	-0.06964	1.865691	-0.03733	0.970225	0.997953
AstA-R2	175.1769	0.140743	3.79001	0.037135	0.970377	0.997981
PPP4R2r	1489.031	0.038997	1.109292	0.035155	0.971956	0.998137
EMC7	3485.371	-0.02181	0.622917	-0.03502	0.972067	0.998137
arr	558.0726	-0.05039	1.386178	-0.03635	0.971002	0.998137
MsR1	826.4999	-0.05069	1.440169	-0.0352	0.971924	0.998137
Ir62a	352.9059	0.074286	2.083216	0.035659	0.971554	0.998137
mt:tRNA:A	254.5425	-0.06146	1.714065	-0.03585	0.971398	0.998137
rgn	6123.624	0.020053	0.55514	0.036122	0.971185	0.998137
lncRNA:CR	85.61395	-0.13571	3.886599	-0.03492	0.972145	0.998137
lncRNA:CR	82.59849	0.108439	3.079776	0.03521	0.971912	0.998137
Zasp52	470.4454	0.048492	1.37919	0.03516	0.971952	0.998137
Tsf1	19631.83	0.021785	0.623119	0.03496	0.972111	0.998137
CG32533	212.6516	-0.08952	2.5508	-0.0351	0.972004	0.998137
CG32066	1244.266	0.03333	0.919992	0.036228	0.9711	0.998137
lncRNA:CR	1847.072	0.030495	0.894559	0.03409	0.972806	0.99818
pasi1	1254.87	0.052734	1.521946	0.034649	0.97236	0.99818
CG15922	430.0127	0.046566	1.3573	0.034308	0.972632	0.99818
ATPsynbet	59151.02	0.013116	0.387656	0.033835	0.973009	0.99818
HnRNP-K	4771.175	0.019251	0.56068	0.034336	0.972609	0.99818
Ac76E	5485.711	-0.01894	0.558577	-0.0339	0.972953	0.99818
mirr	449.8909	0.067081	1.986146	0.033774	0.973057	0.99818
CG6094	271.38	-0.0661	2.073432	-0.03188	0.974569	0.998329
CAH1	3487.396	0.022604	0.70064	0.032262	0.974263	0.998329
Lrrk	1107.747	0.046851	1.425351	0.03287	0.973779	0.998329
LpR2	8764.929	-0.01789	0.537777	-0.03326	0.973467	0.998329
CG17059	862.1475	-0.03454	1.067386	-0.03236	0.974186	0.998329
DPCoAC	575.5529	-0.0449	1.380389	-0.03253	0.974051	0.998329
mEFG1	1067.974	0.04138	1.297049	0.031903	0.974549	0.998329
Vha68-1	23743.6	-0.0136	0.408457	-0.0333	0.973434	0.998329
l(3)neo38	7411.875	0.017556	0.533252	0.032922	0.973737	0.998329
CG10352	1800.472	-0.03942	1.205563	-0.0327	0.973916	0.998329
Pfdn6	2907.255	0.021001	0.658842	0.031875	0.974571	0.998329
Dlip2	829.9205	-0.04738	1.494755	-0.0317	0.974711	0.998345
TRAM	951.144	-0.04027	1.361665	-0.02958	0.976404	0.998364
CG14777	2805.532	0.017076	0.653624	0.026124	0.979158	0.998364
Xpac	599.091	-0.04511	1.854962	-0.02432	0.980596	0.998364
CSN3	1350.799	0.029187	1.216684	0.023989	0.980861	0.998364
CG7611	3438.522	-0.01436	0.641705	-0.02239	0.982141	0.998364
CG11999	1573.777	0.027894	0.902636	0.030903	0.975347	0.998364
C1GalTA	4437.159	0.015634	0.561649	0.027836	0.977793	0.998364
CG6495	2172.241	0.022442	0.713732	0.031443	0.974916	0.998364
Ing5	1332.688	-0.03102	1.027481	-0.03019	0.975914	0.998364
Ddc	8109.749	-0.01198	0.532116	-0.02251	0.982044	0.998364
CG11768	148.3961	0.075229	2.596905	0.028969	0.976889	0.998364
CG9801	1119.37	-0.0265	0.938931	-0.02822	0.977487	0.998364
Sf3b5	2040.35	-0.02017	0.756732	-0.02666	0.978731	0.998364

Lk6	16243.13	0.01191	0.456171	0.026108	0.979171	0.998364
Dic1	4668.343	-0.01531	0.635616	-0.02408	0.980785	0.998364
ATPsynE	11992.18	-0.01048	0.442533	-0.02369	0.981103	0.998364
CG6276	1269.627	0.02185	1.001802	0.021811	0.982599	0.998364
Scp2	14390.9	0.010034	0.443398	0.022629	0.981946	0.998364
RpS30	33834.19	-0.01011	0.416758	-0.02427	0.980641	0.998364
CG5071	957.7975	-0.04737	1.72901	-0.0274	0.978142	0.998364
Oseg6	515.5986	-0.04472	1.754025	-0.02549	0.979662	0.998364
rswl	693.3795	-0.0358	1.230512	-0.02909	0.976793	0.998364
Moe	4215.516	-0.01324	0.579132	-0.02285	0.981767	0.998364
CG2004	599.2691	0.040339	1.374512	0.029348	0.976587	0.998364
eIF3m	11799.12	-0.01271	0.45179	-0.02814	0.977551	0.998364
mars	112.9574	-0.07878	2.673673	-0.02946	0.976494	0.998364
slv	818.324	0.049369	1.679271	0.029399	0.976546	0.998364
Gdap2	11371.37	0.01263	0.492257	0.025657	0.979531	0.998364
tutl	19005.15	-0.01064	0.409771	-0.02595	0.979294	0.998364
Tusp	3305.494	-0.01462	0.665524	-0.02197	0.982469	0.998364
CG31712	11594.34	0.011364	0.461137	0.024644	0.980339	0.998364
jet	897.148	0.03588	1.594589	0.022501	0.982048	0.998364
Rim	26210.71	0.012768	0.414268	0.03082	0.975413	0.998364
ey	122.728	0.097713	3.338387	0.02927	0.97665	0.998364
RpS8	24716.72	0.011739	0.434389	0.027024	0.978441	0.998364
RpL41	7890.353	-0.01171	0.498763	-0.02348	0.981268	0.998364
CG34141	247.3106	0.055072	2.181749	0.025242	0.979862	0.998364
CG40470	143.933	0.083335	3.230059	0.0258	0.979417	0.998364
TrissinR	2000.713	0.019615	0.860295	0.0228	0.98181	0.998364
18SrRNA-F	343.1505	0.037028	1.494555	0.024775	0.980234	0.998364
Akr1B	4452.182	0.016597	0.560697	0.0296	0.976386	0.998364
vvl	4924.259	-0.01869	0.751912	-0.02485	0.980173	0.998364
fid	1533.682	0.021989	0.99588	0.02208	0.982384	0.998364
sgl	4419.641	0.013501	0.568037	0.023768	0.981038	0.998364
psq	9429.077	0.012503	0.518084	0.024133	0.980746	0.998364
CG31688	1563.493	0.02333	0.862729	0.027042	0.978426	0.998364
qkr58E-1	1272.847	0.022254	0.943675	0.023582	0.981186	0.998364
egl	1187.646	0.027368	0.936656	0.029219	0.97669	0.998364
gammaSna	2129.739	0.019975	0.718662	0.027794	0.977826	0.998364
DCP1	805.2965	0.028544	1.274172	0.022402	0.982127	0.998364
nerfin-1	241.8097	0.070228	2.276438	0.03085	0.975389	0.998364
CG13907	893.2454	0.034198	1.210156	0.028259	0.977456	0.998364
CG43755	260.7312	-0.06533	2.264097	-0.02885	0.976982	0.998364
RhoGEF3	6571.009	-0.0159	0.565995	-0.0281	0.977583	0.998364
hfp	9463.801	0.014722	0.496408	0.029657	0.97634	0.998364
Bgb	4642.623	0.017205	0.623468	0.027596	0.977985	0.998364
CG11537	6153.509	-0.01855	0.648258	-0.02861	0.977177	0.998364
Atg101	2690.852	0.039134	1.374033	0.028481	0.977278	0.998364
CG14200	1923.615	-0.02301	1.001631	-0.02298	0.981669	0.998364
CG7460	73.19449	-0.08701	3.83233	-0.0227	0.981887	0.998364
RpS4	16871.57	0.009301	0.424089	0.021931	0.982503	0.998364
CG4911	17837.65	-0.00952	0.438619	-0.0217	0.982691	0.998364
CG4611	259.0888	-0.07396	2.66542	-0.02775	0.977862	0.998364

CG32523	174.214	0.065862	2.624397	0.025096	0.979978	0.998364
Nmnat	1335.052	-0.02356	1.103373	-0.02136	0.982961	0.998512
Moca-cyp	1742.441	0.016019	0.778345	0.020581	0.98358	0.998627
CG3224	998.1129	0.020549	0.98563	0.020849	0.983366	0.998627
Nf1	3504.753	-0.01275	0.618187	-0.02062	0.983546	0.998627
Vps20	612.157	-0.02972	1.454538	-0.02044	0.983696	0.998627
RpS12	20454.06	0.008909	0.42476	0.020973	0.983267	0.998627
Tspo	374.0332	0.02956	1.472017	0.020081	0.983979	0.998787
Pgam5	769.522	-0.03751	1.888099	-0.01987	0.984151	0.998836
CG8372	3412.662	-0.01398	0.757661	-0.01845	0.985283	0.999003
Reps	1130.71	-0.02049	1.060069	-0.01933	0.984581	0.999003
CG12594	2141.679	0.013756	0.744054	0.018488	0.985249	0.999003
CG5455	8381.627	0.010853	0.576353	0.018831	0.984976	0.999003
spt4	635.3579	-0.02234	1.159076	-0.01927	0.984626	0.999003
cic	6713.877	-0.01	0.526857	-0.01898	0.984855	0.999003
CG32280	1756.095	0.017098	0.928714	0.01841	0.985311	0.999003
CG4789	417.0538	0.039153	2.061529	0.018992	0.984848	0.999003
ppl	1654.658	0.01389	0.902661	0.015388	0.987723	0.999061
CG10470	2179.692	0.013281	0.814351	0.016308	0.986989	0.999061
mRpL19	947.1948	-0.021	1.438319	-0.0146	0.988353	0.999061
Acyp2	912.2747	0.014728	1.020139	0.014438	0.988481	0.999061
Not3	6676.585	-0.00872	0.532872	-0.01636	0.986947	0.999061
PCNA	263.4904	-0.04513	2.846239	-0.01585	0.98735	0.999061
CG15611	3015.594	-0.01159	0.731446	-0.01584	0.987359	0.999061
Mlf	10008.96	-0.00823	0.552293	-0.0149	0.988115	0.999061
CG8726	3226.591	0.010569	0.645374	0.016376	0.986934	0.999061
dpy	1653.502	-0.02205	1.426239	-0.01546	0.987667	0.999061
vir-1	23390.44	0.007076	0.485106	0.014586	0.988362	0.999061
MFS17	17120.36	-0.00823	0.47253	-0.01743	0.986096	0.999061
gish	12149.89	0.007341	0.481527	0.015246	0.987836	0.999061
CG42263	198.6739	-0.04504	3.057265	-0.01473	0.988245	0.999061
RpL37A	9475.194	0.007631	0.468979	0.016272	0.987017	0.999061
Bsg	20214.18	-0.00735	0.410289	-0.01792	0.985704	0.999061
dikar	5127.009	0.008792	0.544288	0.016152	0.987113	0.999061
Psn	1357.248	-0.01273	0.865456	-0.01471	0.988262	0.999061
l(3)80Fj	16666.68	-0.00659	0.430972	-0.0153	0.987792	0.999061
CG13919	338.6262	-0.0461	2.636032	-0.01749	0.986048	0.999061
CG16753	405.4688	0.031653	2.063878	0.015337	0.987764	0.999061
CG17746	2568.542	-0.01326	0.900364	-0.01472	0.988252	0.999061
Teh2	5428.62	0.00987	0.574745	0.017173	0.986299	0.999061
GCS1	423.4571	-0.02438	1.553866	-0.01569	0.987483	0.999061
Ist1	2345.496	-0.01577	0.917868	-0.01718	0.986293	0.999061
CG10340	4136.75	-0.00854	0.600023	-0.01424	0.988638	0.999094
Mnt	3937.365	-0.00844	0.603248	-0.01399	0.988836	0.999169
CG3021	864.1273	0.018574	1.773335	0.010474	0.991643	0.999203
CG10803	1460.413	0.010658	0.873688	0.012199	0.990267	0.999203
CG33080	4088.447	0.006942	0.739757	0.009384	0.992513	0.999203
CG3781	557.7015	-0.0163	1.905659	-0.00855	0.993175	0.999203
CG15894	2081.362	0.009319	0.796329	0.011703	0.990663	0.999203
laza	525.9134	0.007274	1.436306	0.005064	0.995959	0.999203

Snm1	116.279	-0.02261	3.749471	-0.00603	0.995189	0.999203
CG31915	1561.981	0.009923	0.86921	0.011416	0.990892	0.999203
CG31710	1545.433	0.013636	1.397372	0.009758	0.992214	0.999203
ThrRS	2412.703	-0.00426	0.711293	-0.00599	0.995218	0.999203
DCTN5-p2!	631.2067	-0.01032	1.213492	-0.0085	0.993214	0.999203
Ref1	3487.729	0.008849	0.658349	0.013442	0.989275	0.999203
dsx	2397.043	0.008592	0.879	0.009775	0.992201	0.999203
CG9922	796.2831	-0.00527	1.154906	-0.00456	0.996358	0.999203
CG31344	6948.334	0.002879	0.612642	0.0047	0.99625	0.999203
Pak3	2840.77	0.008495	0.661442	0.012844	0.989753	0.999203
CG31229	265.1633	-0.01356	1.825456	-0.00743	0.994075	0.999203
CG6178	134.014	0.017515	2.259127	0.007753	0.993814	0.999203
fs(1)h	17161.28	-0.00559	0.528427	-0.01058	0.991557	0.999203
CG12123	1479.161	0.004994	0.861989	0.005793	0.995378	0.999203
stum	283.1163	0.015998	2.53394	0.006314	0.994963	0.999203
O-fut1	460.6669	-0.01811	1.647496	-0.01099	0.991231	0.999203
CG17716	4028.417	0.005521	0.939877	0.005874	0.995313	0.999203
ZnT49B	1696.653	-0.00417	0.791884	-0.00526	0.995802	0.999203
Nup188	93.71843	-0.02646	3.389874	-0.00781	0.993772	0.999203
wal	1949.834	0.004625	0.785365	0.005889	0.995301	0.999203
Dbp45A	1004.47	-0.01679	1.761395	-0.00953	0.992395	0.999203
CG12769	3603.769	-0.0034	0.637792	-0.00533	0.995744	0.999203
CG11141	1361.854	0.017718	1.424818	0.012435	0.990078	0.999203
ABCD	6845.658	0.002697	0.568282	0.004747	0.996213	0.999203
CG9776	2412.109	-0.00551	0.736314	-0.00748	0.994035	0.999203
CG18003	416.2089	-0.03504	2.545382	-0.01377	0.989016	0.999203
loqs	1629.473	0.008546	1.128233	0.007574	0.993957	0.999203
RSG7	3810.092	0.002943	0.644149	0.004569	0.996354	0.999203
Pglym78	20283.09	0.004732	0.419809	0.011273	0.991006	0.999203
Saf-B	5249.825	0.004328	0.651754	0.00664	0.994702	0.999203
Epac	7417.918	0.002585	0.583178	0.004432	0.996464	0.999203
Ziz	6646.288	-0.00269	0.549531	-0.00489	0.996098	0.999203
CG17683	3907.747	-0.00477	0.605554	-0.00787	0.993717	0.999203
MtnE	90.77983	0.016519	3.334148	0.004954	0.996047	0.999203
p130CAS	2661.874	0.008881	0.725225	0.012246	0.99023	0.999203
Gk1	2759.957	0.0049	0.788607	0.006213	0.995043	0.999203
ND-30	10386.93	0.006817	0.500172	0.01363	0.989125	0.999203
Ggamma3(	53843.4	-0.00284	0.376771	-0.00754	0.993981	0.999203
sbb	10395.52	-0.00269	0.49468	-0.00543	0.995668	0.999203
scb	566.415	-0.00724	1.29371	-0.0056	0.995534	0.999203
CG1275	4270.676	0.005173	0.591471	0.008746	0.993022	0.999203
Ythdc1	3527.538	0.003294	0.661895	0.004977	0.996029	0.999203
Ras64B	2930.519	-0.00654	0.674904	-0.00969	0.992269	0.999203
ImpL2	413.5248	-0.00857	1.541915	-0.00556	0.995566	0.999203
Cip4	4343.062	-0.00422	0.587411	-0.00719	0.994264	0.999203
CG11122	1164.187	-0.00962	1.055253	-0.00911	0.992729	0.999203
CG2691	399.2411	0.011538	1.427901	0.008081	0.993553	0.999203
CG6617	1228.655	-0.00843	0.920712	-0.00916	0.992692	0.999203
rictor	736.9918	0.019361	1.510859	0.012815	0.989776	0.999203
CG3961	1121.888	-0.01385	1.085179	-0.01276	0.989819	0.999203

Ube3a	692.1861	0.01944	1.671134	0.011633	0.990719	0.999203
PGRP-LF	75.23407	0.040167	3.930881	0.010218	0.991847	0.999203
CG7182	504.8238	-0.01399	1.34855	-0.01037	0.991726	0.999203
l(1)G0196	5566.315	-0.00707	0.531106	-0.01331	0.98938	0.999203
Tango14	332.2792	0.008939	1.819431	0.004913	0.99608	0.999203
Catsup	1218.332	0.003373	0.939086	0.003592	0.997134	0.999242
l(3)87Df	1796.632	0.002625	0.772122	0.003399	0.997288	0.999242
ogre	6798.114	-0.00241	0.683194	-0.00353	0.99718	0.999242
CG34393	450.3883	-0.0049	1.334805	-0.00367	0.997073	0.999242
Nedd4	4551.866	-0.00255	0.745536	-0.00342	0.997271	0.999242
CG9921	6665.514	0.002082	0.55637	0.003742	0.997015	0.999242
RpS19a	11931.17	0.001584	0.481312	0.00329	0.997375	0.999242
vkg	5180.042	0.001231	0.55241	0.002228	0.998222	0.999343
Slmap	1771.369	0.002007	0.815715	0.00246	0.998037	0.999343
CG34289	69.06509	0.010579	3.931524	0.002691	0.997853	0.999343
tweek	3966.173	-0.00176	0.7634	-0.00231	0.998158	0.999343
kibra	2519.505	0.001909	0.728806	0.00262	0.99791	0.999343
ITP	1537.586	-0.00289	1.074146	-0.00269	0.997851	0.999343
CG1785	101.2624	-0.00577	2.83871	-0.00203	0.998378	0.999374
Btk29A	1862.336	-0.00125	0.875127	-0.00143	0.998861	0.999521
l(3)mbt	577.098	0.002484	1.485544	0.001672	0.998666	0.999521
CG11961	202.2453	-0.00345	2.500199	-0.00138	0.998899	0.999521
CG1677	3680.574	0.000741	0.671747	0.001103	0.99912	0.999618
l(2)05287	216.8852	-0.00131	1.931712	-0.00068	0.999461	0.99971
CG1537	5892.174	0.000401	0.580192	0.000692	0.999448	0.99971
CG46281	166.8387	0.001964	3.825087	0.000513	0.99959	0.999715
CG2938	1315.862	-4.50E-05	1.44339	-3.12E-05	0.999975	0.999975
tRNA:Pro-(	0	NA	NA	NA	NA	NA
CG9570	0	NA	NA	NA	NA	NA
Or19b	0	NA	NA	NA	NA	NA
CG15322	0.596853	0.454699	3.988272	0.114009	0.909231	NA
Or19a	0	NA	NA	NA	NA	NA
karr	0	NA	NA	NA	NA	NA
fd19B	0	NA	NA	NA	NA	NA
CG17003	53.64368	-2.27518	3.952705	-0.5756	0.564885	NA
CG12655	0	NA	NA	NA	NA	NA
CR32821	0	NA	NA	NA	NA	NA
CG3777	0	NA	NA	NA	NA	NA
CG12470	5.943496	-1.90071	3.957353	-0.4803	0.631016	NA
ac	0	NA	NA	NA	NA	NA
sc	0.69928	1.019866	4.009445	0.254366	0.799213	NA
l(1)sc	2.509476	-1.87226	3.965749	-0.47211	0.636851	NA
ase	0	NA	NA	NA	NA	NA
CG13373	0	NA	NA	NA	NA	NA
CG32817	2.251365	0.241545	3.960486	0.060989	0.951368	NA
CG3176	47.98665	1.672485	3.412548	0.490099	0.624064	NA
arg	25.54678	1.746595	3.952934	0.441848	0.658599	NA
Cyp4g1	0	NA	NA	NA	NA	NA
Pgcl	0	NA	NA	NA	NA	NA
CG32816	12.33805	-2.25201	3.958532	-0.5689	0.569424	NA

CG3038	0.5235	1.490714	4.02555	0.370313	0.711149	NA
fz3	1.827476	-1.96736	3.880646	-0.50697	0.612179	NA
CG14635	0	NA	NA	NA	NA	NA
CG13358	0.178053	-1.16467	4.075067	-0.2858	0.775028	NA
CG13359	0	NA	NA	NA	NA	NA
CG13360	0.1047	0.613261	4.062799	0.150945	0.880019	NA
CG13361	0.132078	-1.13211	4.069918	-0.27817	0.780885	NA
CG13362	16.45073	-2.25935	3.956672	-0.57102	0.567983	NA
CG14634	0	NA	NA	NA	NA	NA
CG11664	0	NA	NA	NA	NA	NA
CG11663	0	NA	NA	NA	NA	NA
mus81	29.37713	5.548322	3.87554	1.431625	0.152251	NA
CG18823	0	NA	NA	NA	NA	NA
CG14631	0	NA	NA	NA	NA	NA
CG14629	0	NA	NA	NA	NA	NA
CG14628	0	NA	NA	NA	NA	NA
TfIIA-S-2	0.178812	-1.08808	4.061735	-0.26789	0.788788	NA
CG3690	0.666238	-2.57811	4.01049	-0.64284	0.520326	NA
CG3699	0	NA	NA	NA	NA	NA
CG3708	0	NA	NA	NA	NA	NA
CG3713	29.55684	-1.70427	3.952205	-0.43122	0.666308	NA
CG32814	14.97075	-3.48987	3.904596	-0.89379	0.371436	NA
CG11379	0	NA	NA	NA	NA	NA
CG14627	0	NA	NA	NA	NA	NA
CG14626	7.000117	-1.904	3.956398	-0.48124	0.630343	NA
CG11380	0.233093	0.692656	4.076246	0.169925	0.865069	NA
CG14625	0	NA	NA	NA	NA	NA
CG11381	0	NA	NA	NA	NA	NA
CG14624	0	NA	NA	NA	NA	NA
CG11382	0	NA	NA	NA	NA	NA
fs(1)N	15.1631	3.24396	3.876965	0.836727	0.402746	NA
Tsp2A	0.356107	-1.47099	4.037764	-0.36431	0.715628	NA
CG14770	7.846744	1.52291	3.955441	0.385017	0.700225	NA
png	14.49113	3.125105	3.87146	0.807216	0.419542	NA
CG11409	0	NA	NA	NA	NA	NA
CG32811	6.52661	1.15925	3.958017	0.292887	0.769609	NA
CG11384	1.127155	-1.04467	3.985057	-0.26215	0.793209	NA
eIF4E7	0	NA	NA	NA	NA	NA
CG14780	0	NA	NA	NA	NA	NA
dor	33.48112	6.069143	3.829785	1.584722	0.11303	NA
CG14797	14.67598	4.458059	3.84997	1.157946	0.246886	NA
b6	26.45774	1.674484	3.952757	0.423624	0.67184	NA
Scgdelta	6.972379	2.008518	3.833463	0.523943	0.600318	NA
CG3795	0	NA	NA	NA	NA	NA
CG14795	0	NA	NA	NA	NA	NA
snoRNA:M	0	NA	NA	NA	NA	NA
CG32808	41.08399	0.260724	3.835126	0.067983	0.945799	NA
O-fut2	24.43814	-4.11311	3.80769	-1.08021	0.280048	NA
CG14787	8.033656	5.68891	3.91627	1.452635	0.146325	NA
CG32806	0	NA	NA	NA	NA	NA

CG4194	11.1405	-3.47524	3.907049	-0.88948	0.373746	NA
CG14054	0	NA	NA	NA	NA	NA
CG4325	10.46999	1.73528	3.955796	0.438668	0.660902	NA
CG3857	49.14536	-5.04624	3.804291	-1.32646	0.184687	NA
CG32801	0	NA	NA	NA	NA	NA
CG14811	0.411147	-0.29058	4.046973	-0.0718	0.94276	NA
Cyp4d2	0	NA	NA	NA	NA	NA
Cyp4ae1	40.26957	1.239645	3.835228	0.323226	0.746524	NA
CG17777	0	NA	NA	NA	NA	NA
CG3457	0.396233	-1.6814	4.028351	-0.41739	0.676392	NA
Mct1	23.21806	-0.41976	3.733453	-0.11243	0.910482	NA
CG14052	0.178812	-1.08808	4.061735	-0.26789	0.788788	NA
Or2a	0	NA	NA	NA	NA	NA
CG2854	0.534919	-2.31746	4.015737	-0.5771	0.563875	NA
FarO	4.947763	-4.01998	3.888438	-1.03383	0.301216	NA
CG14053	0.712213	-1.57119	3.998613	-0.39293	0.694369	NA
Cyp4d14	0.132078	-1.13211	4.069918	-0.27817	0.780885	NA
Seipin	51.68751	0.221951	3.857521	0.057537	0.954117	NA
Klp3A	0	NA	NA	NA	NA	NA
Vha36-3	54.53617	1.513311	3.951557	0.382966	0.701745	NA
CG12496	0.53416	-1.53481	4.012395	-0.38252	0.702078	NA
CG2694	6.121549	-3.43093	3.914637	-0.87644	0.380793	NA
fs(1)Ya	4.225249	-5.62961	3.918506	-1.43667	0.150811	NA
CG12498	0	NA	NA	NA	NA	NA
dyw	46.29056	0.844852	3.951506	0.213805	0.830699	NA
HLH3B	5.594238	1.155882	3.959178	0.29195	0.770325	NA
ng1	0	NA	NA	NA	NA	NA
Sgs4	0	NA	NA	NA	NA	NA
CG10801	0	NA	NA	NA	NA	NA
CG12206	1.007638	-1.13898	3.915925	-0.29086	0.771159	NA
CG16781	0	NA	NA	NA	NA	NA
CG16782	0	NA	NA	NA	NA	NA
CG14269	0	NA	NA	NA	NA	NA
tRNA:Gln-⁻	0	NA	NA	NA	NA	NA
CG12535	27.93449	2.769589	3.917916	0.706904	0.479626	NA
CG10793	5.081286	4.061506	3.872903	1.048698	0.294317	NA
Pig1	0	NA	NA	NA	NA	NA
ng4	0	NA	NA	NA	NA	NA
ng3	0	NA	NA	NA	NA	NA
ng2	2.775422	-0.26042	3.958314	-0.06579	0.947545	NA
CG14265	28.31047	-1.7041	3.952262	-0.43117	0.666345	NA
CG3526	0.337716	1.464149	4.04412	0.362044	0.717319	NA
CG32793	12.66808	5.031562	3.897416	1.290999	0.196704	NA
CG3592	0	NA	NA	NA	NA	NA
CG3598	0.178053	-1.16467	4.075067	-0.2858	0.775028	NA
CG14420	0.565506	-0.26224	3.986711	-0.06578	0.947554	NA
CG14421	0	NA	NA	NA	NA	NA
CG14422	0.356866	-1.92326	4.037889	-0.4763	0.633858	NA
CG14423	0.282677	-0.09599	4.057934	-0.02366	0.981127	NA
CG17959	0	NA	NA	NA	NA	NA

CG4116	0	NA	NA	NA	NA	NA
CG14424	0.1047	0.613261	4.062799	0.150945	0.880019	NA
CG14419	0.178053	-1.16467	4.075067	-0.2858	0.775028	NA
CG14418	0	NA	NA	NA	NA	NA
CG14417	0.53416	-1.53481	4.012395	-0.38252	0.702078	NA
CG14416	0	NA	NA	NA	NA	NA
CG13021	0	NA	NA	NA	NA	NA
Ilp7	30.57238	1.747916	3.952603	0.442219	0.658331	NA
Gas8	0	NA	NA	NA	NA	NA
tRNA:Gln-(	1.120046	2.129602	3.955197	0.538431	0.590279	NA
tRNA:Pro-(	0	NA	NA	NA	NA	NA
CG15239	0.178812	-1.08808	4.061735	-0.26789	0.788788	NA
Gem4b	0.523116	1.34877	4.007682	0.336546	0.736459	NA
Gem4c	0	NA	NA	NA	NA	NA
CG6414	0	NA	NA	NA	NA	NA
CG15375	0.1047	0.613261	4.062799	0.150945	0.880019	NA
dgt4	0.132078	-1.13211	4.069918	-0.27817	0.780885	NA
CG15570	0.2094	1.005034	4.045341	0.248442	0.803792	NA
CG12691	0	NA	NA	NA	NA	NA
CG15572	0	NA	NA	NA	NA	NA
CG12688	0	NA	NA	NA	NA	NA
CG12693	0	NA	NA	NA	NA	NA
mei-9	15.5429	-6.22776	3.891624	-1.6003	0.109532	NA
CG15579	0	NA	NA	NA	NA	NA
CG12692	0.628123	2.274123	4.000539	0.568454	0.569727	NA
CG15578	1.340183	1.096454	3.924919	0.279357	0.779971	NA
CG15577	0	NA	NA	NA	NA	NA
CG15576	0.283512	-0.2331	4.065094	-0.05734	0.954273	NA
Muc4B	0	NA	NA	NA	NA	NA
Femcoat	0	NA	NA	NA	NA	NA
CG15571	0	NA	NA	NA	NA	NA
CG3546	0	NA	NA	NA	NA	NA
lncRNA:CR	24.15861	1.618454	3.937837	0.411001	0.681072	NA
CG7024	54.19783	-3.52218	3.899278	-0.90329	0.366371	NA
CG6978	0.2094	1.005034	4.045341	0.248442	0.803792	NA
CG12682	0	NA	NA	NA	NA	NA
boil	0.282677	-0.09599	4.057934	-0.02366	0.981127	NA
CG6927	0.178053	-1.16467	4.075067	-0.2858	0.775028	NA
CG12680	0.411147	-0.29058	4.046973	-0.0718	0.94276	NA
CG15468	0	NA	NA	NA	NA	NA
CG15471	0	NA	NA	NA	NA	NA
CG2871	0.178053	-1.16467	4.075067	-0.2858	0.775028	NA
Klf15	0	NA	NA	NA	NA	NA
CG15472	0	NA	NA	NA	NA	NA
CG15465	0	NA	NA	NA	NA	NA
dhd	0	NA	NA	NA	NA	NA
CG4198	0	NA	NA	NA	NA	NA
CG17764	10.03708	-1.02004	3.747304	-0.27221	0.785464	NA
CG3323	0	NA	NA	NA	NA	NA
CG16756	0	NA	NA	NA	NA	NA

CG4151	0	NA	NA	NA	NA	NA
Pop1	54.83023	-0.24009	3.230275	-0.07433	0.940751	NA
CG32762	0	NA	NA	NA	NA	NA
frma	0.469871	0.864471	4.023538	0.214854	0.829881	NA
NAAT1	30.88003	0.285581	3.838183	0.074405	0.940688	NA
Gr5a	0	NA	NA	NA	NA	NA
cv	6.626178	0.834101	3.855968	0.216314	0.828743	NA
CG15773	0.178053	-1.16467	4.075067	-0.2858	0.775028	NA
CG15767	0	NA	NA	NA	NA	NA
CG12239	14.31253	-0.30075	3.845539	-0.07821	0.937664	NA
Cpr5C	0.356107	-1.47099	4.037764	-0.36431	0.715628	NA
Prosbeta2l	0	NA	NA	NA	NA	NA
tRNA:Glu-(	0	NA	NA	NA	NA	NA
CG12729	0	NA	NA	NA	NA	NA
CG15764	0.356107	-1.47099	4.037764	-0.36431	0.715628	NA
CG4020	54.23456	1.750878	3.951866	0.443051	0.657729	NA
AgmNAT	0	NA	NA	NA	NA	NA
Tre1	0.178053	-1.16467	4.075067	-0.2858	0.775028	NA
CG32755	3.309582	-0.26274	3.956962	-0.0664	0.94706	NA
CG6048	0	NA	NA	NA	NA	NA
CG6067	0	NA	NA	NA	NA	NA
Grip	0.566948	0.490522	3.992077	0.122874	0.902207	NA
CG5966	5.695097	5.005505	3.924253	1.275531	0.202121	NA
CG5921	23.96085	-2.26641	3.954895	-0.57307	0.566601	NA
Btnd	0	NA	NA	NA	NA	NA
vanin-like	0.1047	0.613261	4.062799	0.150945	0.880019	NA
CG32751	0	NA	NA	NA	NA	NA
CG32750	43.45356	-1.91996	3.951805	-0.48584	0.627078	NA
Mipp2	6.339729	-1.90207	3.956958	-0.48069	0.630738	NA
raptor	40.23547	2.505067	3.563025	0.703073	0.48201	NA
fs(1)M3	0.178053	-1.16467	4.075067	-0.2858	0.775028	NA
mldr	0.209247	1.042804	4.047837	0.25762	0.7967	NA
tRNA:Phe-	0.2094	1.005034	4.045341	0.248442	0.803792	NA
CG3342	48.16197	1.750376	3.951986	0.44291	0.65783	NA
swa	0	NA	NA	NA	NA	NA
swaPsi	0.178053	-1.16467	4.075067	-0.2858	0.775028	NA
CG15880	0	NA	NA	NA	NA	NA
Pkg21D	0	NA	NA	NA	NA	NA
lectin-21Cl	0	NA	NA	NA	NA	NA
CG33127	0	NA	NA	NA	NA	NA
al	0	NA	NA	NA	NA	NA
CG13692	0.535678	-2.26616	4.021604	-0.5635	0.573098	NA
BBS8	0.2094	1.005034	4.045341	0.248442	0.803792	NA
Pph13	0	NA	NA	NA	NA	NA
CG11835	0.894061	-2.00006	4.030294	-0.49626	0.619714	NA
Ets21C	0.63886	-0.79045	3.986802	-0.19827	0.842837	NA
CG13690	0.31387	1.261782	4.037145	0.312543	0.754628	NA
CG4213	0	NA	NA	NA	NA	NA
Ent1	10.90318	-2.34605	3.76509	-0.62311	0.533214	NA
CG11911	0.1047	0.613261	4.062799	0.150945	0.880019	NA

CG11912	3.140998	1.694072	3.966495	0.427096	0.66931	NA
CG3345	0.178812	-1.08808	4.061735	-0.26789	0.788788	NA
Amnionles	1.570499	1.642769	3.980387	0.412716	0.679815	NA
CG3709	34.57163	1.654503	3.812925	0.43392	0.664347	NA
CG31975	0	NA	NA	NA	NA	NA
ovm	0	NA	NA	NA	NA	NA
Ir21a	0.132078	-1.13211	4.069918	-0.27817	0.780885	NA
Spn77Bc	47.18428	-5.69436	3.830699	-1.48651	0.137145	NA
CG5665	27.59826	-1.70399	3.952297	-0.43114	0.666368	NA
in	0.528311	-1.72839	4.011871	-0.43082	0.6666	NA
CG13247	0	NA	NA	NA	NA	NA
zye	60.78647	-2.03529	3.538433	-0.5752	0.565159	NA
mag	0	NA	NA	NA	NA	NA
CG18281	4.866759	-1.81691	3.846772	-0.47232	0.636697	NA
CG13250	0	NA	NA	NA	NA	NA
knrl	3.426486	0.40965	3.855283	0.106257	0.915379	NA
CG5199	60.85185	0.417134	3.834227	0.108792	0.913367	NA
Toll-9	0.178053	-1.16467	4.075067	-0.2858	0.775028	NA
CG13251	0.178053	-1.16467	4.075067	-0.2858	0.775028	NA
Spc105R	0.178053	-1.16467	4.075067	-0.2858	0.775028	NA
CG3288	0	NA	NA	NA	NA	NA
CG11458	0.132078	-1.13211	4.069918	-0.27817	0.780885	NA
CG3634	54.96167	-3.78299	3.801658	-0.99509	0.319693	NA
CG3698	0.178053	-1.16467	4.075067	-0.2858	0.775028	NA
Six4	0.1047	0.613261	4.062799	0.150945	0.880019	NA
CG11396	0	NA	NA	NA	NA	NA
Gr77a	43.45138	-2.2735	3.953123	-0.57512	0.565213	NA
kni	1.279731	0.371782	3.896693	0.09541	0.923989	NA
CG10588	0	NA	NA	NA	NA	NA
CR33283	0	NA	NA	NA	NA	NA
CG12984	22.88797	-2.26568	3.955079	-0.57285	0.566744	NA
CG33284	28.27352	-0.04896	3.951524	-0.01239	0.990115	NA
CG10510	1.056621	-1.8126	3.984111	-0.45496	0.64914	NA
park	62.01447	-0.10618	3.097487	-0.03428	0.972655	NA
Ac78C	52.4844	-3.22874	3.659505	-0.88229	0.377621	NA
CG33286	0.461489	-1.26887	4.028229	-0.31499	0.752766	NA
CG33287	0.178812	-1.08808	4.061735	-0.26789	0.788788	NA
CG12983	49.00082	-1.92032	3.951703	-0.48595	0.627005	NA
CG10587	18.62295	-1.9158	3.952995	-0.48464	0.627928	NA
CG11037	9.773749	-1.90931	3.95486	-0.48278	0.629255	NA
Sems	0	NA	NA	NA	NA	NA
Cpr78Cb	0.53416	-1.53481	4.012395	-0.38252	0.702078	NA
Cpr78Cc	0	NA	NA	NA	NA	NA
CG32436	0.53416	-1.53481	4.012395	-0.38252	0.702078	NA
CG32437	0.1047	0.613261	4.062799	0.150945	0.880019	NA
CG32440	0	NA	NA	NA	NA	NA
ppk5	48.05124	-9.28765	3.907707	-2.37675	0.017466	NA
CG33290	0	NA	NA	NA	NA	NA
CG7634	22.19439	6.474651	3.90132	1.659605	0.096994	NA
CG7173	0	NA	NA	NA	NA	NA

CG7202	2.824828	1.495401	3.963153	0.377326	0.705931	NA
Neu2	38.09646	-0.11829	3.951704	-0.02993	0.97612	NA
croc	4.886874	-1.89605	3.958711	-0.47896	0.631969	NA
sa	0	NA	NA	NA	NA	NA
CG12971	0	NA	NA	NA	NA	NA
CG11309	9.06951	2.008509	3.807331	0.527537	0.597821	NA
Cpr78Ca	0.178053	-1.16467	4.075067	-0.2858	0.775028	NA
CG12974	0	NA	NA	NA	NA	NA
CG9389	47.39566	-0.42051	3.834442	-0.10967	0.912674	NA
CG32445	0	NA	NA	NA	NA	NA
CG11249	0	NA	NA	NA	NA	NA
CG14573	0	NA	NA	NA	NA	NA
CG14572	0	NA	NA	NA	NA	NA
CG14564	0	NA	NA	NA	NA	NA
CG7370	15.9305	3.526832	3.903133	0.90359	0.366213	NA
eg	0	NA	NA	NA	NA	NA
CycH	3.839014	-1.92253	3.881852	-0.49526	0.620415	NA
CG14563	0	NA	NA	NA	NA	NA
CG14565	0	NA	NA	NA	NA	NA
CG14566	0	NA	NA	NA	NA	NA
CG14567	0	NA	NA	NA	NA	NA
CG14568	0	NA	NA	NA	NA	NA
CG14569	0.1047	0.613261	4.062799	0.150945	0.880019	NA
CG14570	0	NA	NA	NA	NA	NA
CG32447	0	NA	NA	NA	NA	NA
Cpr78E	0	NA	NA	NA	NA	NA
CR7448	0.233093	0.692656	4.076246	0.169925	0.865069	NA
Chs2	0	NA	NA	NA	NA	NA
P5CS	0	NA	NA	NA	NA	NA
msopa	0	NA	NA	NA	NA	NA
CG7140	5.831849	1.457586	3.95798	0.368265	0.712676	NA
tRNA:Cys-(	0	NA	NA	NA	NA	NA
CG32450	18.95015	4.947279	3.886956	1.27279	0.203093	NA
CG11437	0	NA	NA	NA	NA	NA
Trxr-2	19.89299	1.744326	3.953505	0.44121	0.659061	NA
ND-B14.5A	0	NA	NA	NA	NA	NA
CG14457	0.233093	0.692656	4.076246	0.169925	0.865069	NA
CG14456	0	NA	NA	NA	NA	NA
CG14453	0	NA	NA	NA	NA	NA
CG14452	0	NA	NA	NA	NA	NA
CG32453	0	NA	NA	NA	NA	NA
CG11370	0.178053	-1.16467	4.075067	-0.2858	0.775028	NA
CG11367	10.02176	-3.15571	3.853013	-0.81903	0.412772	NA
CG32454	5.665974	-0.13909	3.910837	-0.03556	0.97163	NA
CG14448	0.178812	-1.08808	4.061735	-0.26789	0.788788	NA
l(3)04053	57.34553	-1.70858	3.5258	-0.48459	0.627964	NA
CG14451	0	NA	NA	NA	NA	NA
CG12546	0	NA	NA	NA	NA	NA
CG14454	0	NA	NA	NA	NA	NA
CG14455	0	NA	NA	NA	NA	NA

CG11226	0.31089	-1.9003	4.033427	-0.47114	0.637542	NA
CG13239	0.178053	-1.16467	4.075067	-0.2858	0.775028	NA
CG11109	0	NA	NA	NA	NA	NA
Ssl1	39.55236	3.751206	3.819849	0.98203	0.326085	NA
CG11131	0	NA	NA	NA	NA	NA
BoYb	0	NA	NA	NA	NA	NA
CG32457	0.491923	0.364479	3.983057	0.091507	0.92709	NA
CG32461	0.53416	-1.53481	4.012395	-0.38252	0.702078	NA
snoRNA:M	1.455566	3.686462	3.938983	0.935892	0.349329	NA
CG32459	0.178053	-1.16467	4.075067	-0.2858	0.775028	NA
CG32462	0	NA	NA	NA	NA	NA
tRNA:Glu-(	0	NA	NA	NA	NA	NA
CR1075	0.282677	-0.09599	4.057934	-0.02366	0.981127	NA
Obp83cd	0	NA	NA	NA	NA	NA
Osi1	0	NA	NA	NA	NA	NA
CR31562	0	NA	NA	NA	NA	NA
Osi24	18.81464	1.644989	3.953448	0.41609	0.677344	NA
Osi2	0	NA	NA	NA	NA	NA
Osi3	0	NA	NA	NA	NA	NA
Osi5	0	NA	NA	NA	NA	NA
Osi6	0.178053	-1.16467	4.075067	-0.2858	0.775028	NA
Osi7	0.1047	0.613261	4.062799	0.150945	0.880019	NA
Osi8	0	NA	NA	NA	NA	NA
Osi9	0	NA	NA	NA	NA	NA
Osi12	0.178053	-1.16467	4.075067	-0.2858	0.775028	NA
CG15594	0.1047	0.613261	4.062799	0.150945	0.880019	NA
Osi13	0	NA	NA	NA	NA	NA
Osi14	0	NA	NA	NA	NA	NA
Osi15	0.178812	-1.08808	4.061735	-0.26789	0.788788	NA
Osi16	0.356107	-1.47099	4.037764	-0.36431	0.715628	NA
CG31560	0	NA	NA	NA	NA	NA
Osi17	0.356866	-1.92326	4.037889	-0.4763	0.633858	NA
Osi18	0	NA	NA	NA	NA	NA
CG15597	0	NA	NA	NA	NA	NA
Osi11	0.178053	-1.16467	4.075067	-0.2858	0.775028	NA
Osi4	6.938443	1.866466	3.767334	0.495434	0.620294	NA
CG31559	0	NA	NA	NA	NA	NA
Obp83g	0	NA	NA	NA	NA	NA
Obp83ef	0.104623	0.639075	4.067674	0.157111	0.875158	NA
Or83c	0.178053	-1.16467	4.075067	-0.2858	0.775028	NA
CG15580	0	NA	NA	NA	NA	NA
CG11459	0	NA	NA	NA	NA	NA
CG1208	59.85759	-6.72687	3.865653	-1.74016	0.08183	NA
CG14676	0	NA	NA	NA	NA	NA
CR10991	0	NA	NA	NA	NA	NA
CG10280	0	NA	NA	NA	NA	NA
Obp83b	0	NA	NA	NA	NA	NA
Obp83a	0	NA	NA	NA	NA	NA
CG11373	0	NA	NA	NA	NA	NA
Atu	0	NA	NA	NA	NA	NA

CG31551	0	NA	NA	NA	NA	NA
CG2100	31.94744	6.509401	3.877081	1.678944	0.093163	NA
cas	47.91745	1.536711	3.951659	0.388877	0.697367	NA
CG2104	0.310131	-1.94616	4.045058	-0.48112	0.630431	NA
CG31546	22.47914	1.661438	3.953062	0.420291	0.674273	NA
NKCC	24.38506	1.336043	3.952464	0.338028	0.735342	NA
HDAC3	0	NA	NA	NA	NA	NA
CG12590	0	NA	NA	NA	NA	NA
CG1113	41.32956	6.946828	3.877636	1.791511	0.073211	NA
CG31542	0	NA	NA	NA	NA	NA
tRNA:Arg-⁻	0.446101	0.990384	3.987592	0.248366	0.803851	NA
CG2663	0	NA	NA	NA	NA	NA
Or83a	0	NA	NA	NA	NA	NA
kkv	59.96358	-1.38319	3.072624	-0.45017	0.652591	NA
CG14668	0	NA	NA	NA	NA	NA
CG31538	53.86997	8.421106	3.845727	2.189731	0.028544	NA
Tim17a2	0.132078	-1.13211	4.069918	-0.27817	0.780885	NA
Prosbeta2l	0	NA	NA	NA	NA	NA
CG1124	10.32058	-3.05598	3.917683	-0.78005	0.435363	NA
CG12589	0	NA	NA	NA	NA	NA
plh	0	NA	NA	NA	NA	NA
CG14662	0	NA	NA	NA	NA	NA
CG14661	17.83634	-4.83024	3.877421	-1.24574	0.212862	NA
snRNA:U1:	27.01258	1.74703	3.952825	0.44197	0.658511	NA
laf	0.236778	-0.26946	4.071573	-0.06618	0.947234	NA
slx1	0.356866	-1.92326	4.037889	-0.4763	0.633858	NA
rpk	0	NA	NA	NA	NA	NA
spartin	20.22196	2.028623	3.773879	0.537543	0.590893	NA
BBS5	0	NA	NA	NA	NA	NA
CG14659	0	NA	NA	NA	NA	NA
CG14658	0	NA	NA	NA	NA	NA
Hus1-like	42.38096	-1.85567	3.812901	-0.48668	0.626484	NA
CG17387	0.357625	-1.47707	4.044794	-0.36518	0.714979	NA
CG14655	43.43676	0.361818	3.559478	0.101649	0.919035	NA
CG32945	0	NA	NA	NA	NA	NA
MP1	0	NA	NA	NA	NA	NA
CG14645	0	NA	NA	NA	NA	NA
CG31528	3.769197	1.70343	3.964029	0.429722	0.667398	NA
CG31525	0.178053	-1.16467	4.075067	-0.2858	0.775028	NA
CG1074	7.262137	0.144289	3.84494	0.037527	0.970065	NA
CG14651	0.565506	-0.26224	3.986711	-0.06578	0.947554	NA
lncRNA:CR	0.104623	0.639075	4.067674	0.157111	0.875158	NA
Mur82C	0	NA	NA	NA	NA	NA
CG9804	18.03174	-1.13858	3.800836	-0.29956	0.764511	NA
CG9855	25.27218	1.148413	3.839889	0.299074	0.764883	NA
Skp2	0.233093	0.692656	4.076246	0.169925	0.865069	NA
Vps24	32.38633	2.635777	3.757161	0.701534	0.48297	NA
CG12581	0.5892	-0.44935	3.992199	-0.11256	0.910381	NA
TwdlU	0.365171	-0.43584	4.056359	-0.10745	0.914434	NA
TwdlF	0.3141	1.240997	4.03841	0.307299	0.758616	NA

TwdlV	0	NA	NA	NA	NA	NA
Or82a	0	NA	NA	NA	NA	NA
CG1092	49.59256	7.483543	3.795403	1.971739	0.048639	NA
CG31516	0.283436	-0.20033	4.070792	-0.04921	0.960751	NA
CG14636	0	NA	NA	NA	NA	NA
Dsk	2.377398	-1.86964	3.966533	-0.47135	0.637388	NA
SP555	0	NA	NA	NA	NA	NA
CG14036	0	NA	NA	NA	NA	NA
CG31917	0	NA	NA	NA	NA	NA
tRNA:Asp-(	0	NA	NA	NA	NA	NA
TotM	35.38858	1.748836	3.952374	0.442477	0.658144	NA
CG14022	31.8287	2.968918	3.920455	0.757289	0.448877	NA
tRNA:Asp-(	0	NA	NA	NA	NA	NA
CG18269	0	NA	NA	NA	NA	NA
CG14017	0	NA	NA	NA	NA	NA
snRNA:U4:	0	NA	NA	NA	NA	NA
CG18266	0.178812	-1.08808	4.061735	-0.26789	0.788788	NA
CG12511	0	NA	NA	NA	NA	NA
CG7251	0	NA	NA	NA	NA	NA
CG14011	0	NA	NA	NA	NA	NA
CG14013	0	NA	NA	NA	NA	NA
CG14014	0.849019	-0.87463	3.964455	-0.22062	0.825391	NA
tomb	0	NA	NA	NA	NA	NA
lncRNA:CR	0.497249	-0.69954	4.001026	-0.17484	0.861205	NA
CG7236	0.466186	0.959471	4.033986	0.237847	0.812	NA
asRNA:CR3	0	NA	NA	NA	NA	NA
CG31913	0	NA	NA	NA	NA	NA
CG9029	0	NA	NA	NA	NA	NA
CG14000	3.036298	1.692169	3.966999	0.426562	0.669699	NA
CG9021	0	NA	NA	NA	NA	NA
Acp26Aa	0	NA	NA	NA	NA	NA
Acp26Ab	0.90868	2.395959	3.996542	0.599508	0.548834	NA
CG31644	0	NA	NA	NA	NA	NA
obst-E	1.151699	1.609751	3.989673	0.403479	0.686596	NA
CG14006	6.409473	1.348312	3.955806	0.340844	0.733221	NA
CG11149	0	NA	NA	NA	NA	NA
CG14007	0	NA	NA	NA	NA	NA
Vm26Ab	0	NA	NA	NA	NA	NA
Ucp4C	0	NA	NA	NA	NA	NA
Ucp4B	0	NA	NA	NA	NA	NA
CG13996	0.178053	-1.16467	4.075067	-0.2858	0.775028	NA
CG9222	0.178053	-1.16467	4.075067	-0.2858	0.775028	NA
CG13991	0	NA	NA	NA	NA	NA
Muc26B	0	NA	NA	NA	NA	NA
CG13989	0.178053	-1.16467	4.075067	-0.2858	0.775028	NA
CG31639	0	NA	NA	NA	NA	NA
CG9150	8.490945	-1.76074	3.866851	-0.45534	0.648863	NA
slmo	29.3746	0.187224	3.735255	0.050123	0.960024	NA
CG9107	0.442493	1.796125	4.02306	0.446457	0.655267	NA
H2.0	0.536437	-1.71175	4.038042	-0.42391	0.671635	NA

Gal	0	NA	NA	NA	NA	NA
CG13992	0.178053	-1.16467	4.075067	-0.2858	0.775028	NA
Vm26Aa	0	NA	NA	NA	NA	NA
CG13998	0.3141	1.240997	4.03841	0.307299	0.758616	NA
Ugt37B1	0	NA	NA	NA	NA	NA
CG13984	0.104623	0.639075	4.067674	0.157111	0.875158	NA
ppk14	0	NA	NA	NA	NA	NA
slam	0.596546	0.421256	3.969213	0.106131	0.915479	NA
CG9500	0	NA	NA	NA	NA	NA
CG9497	25.90313	6.186722	3.885785	1.592142	0.111353	NA
CG13983	0	NA	NA	NA	NA	NA
CG9550	19.33806	2.754453	3.923385	0.70206	0.482642	NA
COX5BL	0	NA	NA	NA	NA	NA
cup	0	NA	NA	NA	NA	NA
TTLL3A	0	NA	NA	NA	NA	NA
AkhR	0.178053	-1.16467	4.075067	-0.2858	0.775028	NA
Tsp	0.104623	0.639075	4.067674	0.157111	0.875158	NA
CG11327	0.209247	1.042804	4.047837	0.25762	0.7967	NA
Nse1	0	NA	NA	NA	NA	NA
cort	53.58419	0.255901	3.951247	0.064765	0.948361	NA
CG13771	0	NA	NA	NA	NA	NA
CG31636	0	NA	NA	NA	NA	NA
DIP-iota	1.602479	-1.64094	3.97352	-0.41297	0.67963	NA
IFT52	0	NA	NA	NA	NA	NA
tRNA:Arg-(	0	NA	NA	NA	NA	NA
CG11236	5.697705	-1.68799	3.957546	-0.42652	0.669726	NA
sens-2	42.93819	8.601066	3.908009	2.200882	0.027744	NA
uif	0.565506	-0.26224	3.986711	-0.06578	0.947554	NA
sip2	1.691239	2.13269	3.952787	0.539541	0.589514	NA
CG13775	42.02089	-2.2732	3.953197	-0.57503	0.565272	NA
CG18304	0.178053	-1.16467	4.075067	-0.2858	0.775028	NA
CG32829	16.43789	1.742188	3.954044	0.440609	0.659496	NA
Nha1	30.21928	-2.26967	3.95408	-0.57401	0.565963	NA
CG17375	0	NA	NA	NA	NA	NA
CG17376	22.95625	-0.30077	3.912171	-0.07688	0.938719	NA
nrv1	55.58639	0.250738	3.949678	0.063483	0.949382	NA
LUBEL	8.957704	-0.65558	3.928837	-0.16686	0.867477	NA
CG11322	0	NA	NA	NA	NA	NA
Ugt307A1	0	NA	NA	NA	NA	NA
CG31909	5.530244	-0.45535	3.868976	-0.11769	0.906311	NA
CG13786	0	NA	NA	NA	NA	NA
CG4496	14.38375	0.953029	3.76105	0.253394	0.799963	NA
Pvf2	0.178053	-1.16467	4.075067	-0.2858	0.775028	NA
Nuf2	1.856259	0.798321	3.959062	0.201644	0.840195	NA
Mst27D	23.7504	5.293715	3.876771	1.365496	0.172097	NA
Wnt10	0	NA	NA	NA	NA	NA
ninaC	17.27331	0.338997	3.841003	0.088257	0.929672	NA
gudu	0.264155	-1.54099	4.048976	-0.38059	0.703509	NA
CG5171	0	NA	NA	NA	NA	NA
CG5177	0	NA	NA	NA	NA	NA

CG15818	0	NA	NA	NA	NA	NA
CG6055	0	NA	NA	NA	NA	NA
CG6441	0	NA	NA	NA	NA	NA
Obp28a	0	NA	NA	NA	NA	NA
Gr28b	50.58672	-1.62654	3.85182	-0.42228	0.672821	NA
Gr28a	0.1047	0.613261	4.062799	0.150945	0.880019	NA
CG5958	0	NA	NA	NA	NA	NA
Spn28B	0	NA	NA	NA	NA	NA
Cyp4d21	0	NA	NA	NA	NA	NA
CG6739	0.536437	-1.71175	4.038042	-0.42391	0.671635	NA
Myo28B1	9.836325	3.136111	3.779057	0.829866	0.406615	NA
Tep3	11.85614	-0.27132	3.952006	-0.06865	0.945264	NA
ATPsynGL	0.104623	0.639075	4.067674	0.157111	0.875158	NA
Uro	0	NA	NA	NA	NA	NA
CG7164	0	NA	NA	NA	NA	NA
CG7203	0	NA	NA	NA	NA	NA
Acp1	0	NA	NA	NA	NA	NA
CG13795	0	NA	NA	NA	NA	NA
CG13794	0	NA	NA	NA	NA	NA
CG13793	0	NA	NA	NA	NA	NA
CG18585	0.104623	0.639075	4.067674	0.157111	0.875158	NA
CG14537	0	NA	NA	NA	NA	NA
tRNA:Gly-(	0.903015	-2.03286	3.962887	-0.51297	0.607969	NA
Tg	15.44133	0.158208	3.8378	0.041224	0.967118	NA
CG7367	0	NA	NA	NA	NA	NA
Spn28Dc	41.61653	-3.48473	3.923108	-0.88826	0.374402	NA
CG7227	0	NA	NA	NA	NA	NA
Spn28Db	0	NA	NA	NA	NA	NA
Spn28Da	0.132078	-1.13211	4.069918	-0.27817	0.780885	NA
CG7102	0	NA	NA	NA	NA	NA
spz3	0.209323	1.414133	4.032409	0.350692	0.725819	NA
TwdlE	0.712213	-1.57119	3.998613	-0.39293	0.694369	NA
CG31606	0.209323	1.414133	4.032409	0.350692	0.725819	NA
CG8419	0.671724	-0.20025	3.964006	-0.05052	0.959711	NA
CG8349	0	NA	NA	NA	NA	NA
CG14277	0	NA	NA	NA	NA	NA
tRNA:Lys-T	0	NA	NA	NA	NA	NA
CG14275	5.914742	-1.62872	3.882448	-0.41951	0.674844	NA
CG14273	0	NA	NA	NA	NA	NA
CG7778	0	NA	NA	NA	NA	NA
Mur29B	0	NA	NA	NA	NA	NA
Spn28F	0	NA	NA	NA	NA	NA
CG8292	0	NA	NA	NA	NA	NA
snRNA:U6a	0.910416	0.505751	3.949798	0.128045	0.898114	NA
CG13385	0	NA	NA	NA	NA	NA
CG13386	0	NA	NA	NA	NA	NA
CG31898	6.728173	0.687888	3.930528	0.175012	0.86107	NA
CheA29a	0	NA	NA	NA	NA	NA
tRNA:Asp-(	0	NA	NA	NA	NA	NA
CG13088	13.74307	0.700737	3.928104	0.178391	0.858416	NA

Sgp	54.25647	-1.89598	3.951613	-0.4798	0.631371	NA
CG17834	61.96254	-1.70637	3.951527	-0.43183	0.665867	NA
tRNA:Asp-(	0	NA	NA	NA	NA	NA
tRNA:Phe-	0	NA	NA	NA	NA	NA
tRNA:Asp-(	13.51941	1.169913	3.954372	0.295853	0.767342	NA
tRNA:Asp-(	0	NA	NA	NA	NA	NA
Bace	3.869664	-0.57813	3.808855	-0.15178	0.879357	NA
tRNA:Asp-(	0	NA	NA	NA	NA	NA
grk	1.015653	0.790107	3.976718	0.198683	0.842511	NA
fy	7.625444	-0.12463	3.874471	-0.03217	0.974338	NA
Peritrophi	0	NA	NA	NA	NA	NA
Peritrophi	0	NA	NA	NA	NA	NA
lectin-29C	0	NA	NA	NA	NA	NA
Acp29AB	0	NA	NA	NA	NA	NA
Glt	0	NA	NA	NA	NA	NA
tRNA:Asp-(	0	NA	NA	NA	NA	NA
tRNA:Asp-(	0	NA	NA	NA	NA	NA
CG12438	0	NA	NA	NA	NA	NA
CG32986	0	NA	NA	NA	NA	NA
CG32987	0	NA	NA	NA	NA	NA
CG32988	0	NA	NA	NA	NA	NA
CG32983	0	NA	NA	NA	NA	NA
CG9483	0	NA	NA	NA	NA	NA
LManVI	0	NA	NA	NA	NA	NA
LManV	0	NA	NA	NA	NA	NA
LManIV	0	NA	NA	NA	NA	NA
LManIII	0.178053	-1.16467	4.075067	-0.2858	0.775028	NA
CG9289	0	NA	NA	NA	NA	NA
tRNA:Asp-(	0.941609	1.421141	3.984935	0.356628	0.72137	NA
CG9287	0	NA	NA	NA	NA	NA
Tsp29Fa	0	NA	NA	NA	NA	NA
CG32985	0	NA	NA	NA	NA	NA
CG32984	0	NA	NA	NA	NA	NA
CG18088	0.69928	1.019866	4.009445	0.254366	0.799213	NA
CG18662	0.3141	1.240997	4.03841	0.307299	0.758616	NA
CG17906	0	NA	NA	NA	NA	NA
Try29F	6.634882	3.000912	3.871897	0.77505	0.43831	NA
CG9568	23.60178	-4.34564	3.880838	-1.11977	0.262813	NA
CG13102	0	NA	NA	NA	NA	NA
CG12439	0	NA	NA	NA	NA	NA
Toll-4	1.895259	0.506147	3.963229	0.127711	0.898378	NA
CG31609	0	NA	NA	NA	NA	NA
CG9582	0	NA	NA	NA	NA	NA
Rcd-1r	0	NA	NA	NA	NA	NA
rost	1.309066	-0.383	3.96689	-0.09655	0.923084	NA
CG13101	0	NA	NA	NA	NA	NA
CG9541	40.06199	-1.70532	3.951865	-0.43152	0.666088	NA
CG9525	0	NA	NA	NA	NA	NA
CG17005	0	NA	NA	NA	NA	NA
Cks30A	0.178812	-1.08808	4.061735	-0.26789	0.788788	NA

CG3748	0.1047	0.613261	4.062799	0.150945	0.880019	NA
CG13110	0	NA	NA	NA	NA	NA
Mco1	0.712213	-1.57119	3.998613	-0.39293	0.694369	NA
CG3769	0	NA	NA	NA	NA	NA
CG4438	0	NA	NA	NA	NA	NA
borr	0.104623	0.639075	4.067674	0.157111	0.875158	NA
aust	0.368855	-0.37404	4.015709	-0.09314	0.925789	NA
CG31883	25.34651	0.665054	3.952331	0.168269	0.866372	NA
CG17855	0	NA	NA	NA	NA	NA
Cpr30B	0	NA	NA	NA	NA	NA
CG13113	0.104623	0.639075	4.067674	0.157111	0.875158	NA
CG13114	0.2094	1.005034	4.045341	0.248442	0.803792	NA
CG3841	0	NA	NA	NA	NA	NA
CG33299	0	NA	NA	NA	NA	NA
CG31882	0.132078	-1.13211	4.069918	-0.27817	0.780885	NA
CG4382	0.3141	1.240997	4.03841	0.307299	0.758616	NA
gcm	0	NA	NA	NA	NA	NA
CG31709	0	NA	NA	NA	NA	NA
CG4017	0.178053	-1.16467	4.075067	-0.2858	0.775028	NA
CG17633	0.178053	-1.16467	4.075067	-0.2858	0.775028	NA
IP3K1	35.75338	-0.1091	3.863007	-0.02824	0.97747	NA
CG4709	26.83598	-7.79137	3.909939	-1.99271	0.046293	NA
snoRNA:M	0.178053	-1.16467	4.075067	-0.2858	0.775028	NA
snoRNA:M	2.377398	-1.86964	3.966533	-0.47135	0.637388	NA
snoRNA:U:	0.2094	1.005034	4.045341	0.248442	0.803792	NA
snoRNA:U:	4.249151	4.596953	3.930552	1.169544	0.242185	NA
Cyp4e3	48.85906	1.536765	3.951645	0.388893	0.697356	NA
CG13127	0.1047	0.613261	4.062799	0.150945	0.880019	NA
obst-B	1.046999	1.598136	3.993006	0.400234	0.688984	NA
Cpr31A	0	NA	NA	NA	NA	NA
CG13137	0	NA	NA	NA	NA	NA
CG5731	0	NA	NA	NA	NA	NA
Fum4	0	NA	NA	NA	NA	NA
CG13135	42.50817	1.749815	3.952129	0.442752	0.657945	NA
CG13133	0	NA	NA	NA	NA	NA
CG13131	0	NA	NA	NA	NA	NA
CG4995	0	NA	NA	NA	NA	NA
pim	0	NA	NA	NA	NA	NA
lft	0	NA	NA	NA	NA	NA
CG5096	0.132078	-1.13211	4.069918	-0.27817	0.780885	NA
Ir31a	0	NA	NA	NA	NA	NA
CG13144	0.64424	-0.14539	3.993669	-0.0364	0.97096	NA
Cog4	49.14673	5.433623	3.768638	1.4418	0.149359	NA
RluA-1	0	NA	NA	NA	NA	NA
Cdk1	4.470307	-2.20441	3.970887	-0.55514	0.578797	NA
CG5367	43.14144	0.51971	3.951697	0.131516	0.895367	NA
CG13138	0	NA	NA	NA	NA	NA
Hand	13.7101	-1.69969	3.953695	-0.4299	0.667268	NA
CG18301	0.178053	-1.16467	4.075067	-0.2858	0.775028	NA
CG6138	37.36155	0.475497	3.865059	0.123024	0.902088	NA

CG31872	0	NA	NA	NA	NA	NA
CG18284	0.178053	-1.16467	4.075067	-0.2858	0.775028	NA
CG31871	0	NA	NA	NA	NA	NA
CG17104	0.178053	-1.16467	4.075067	-0.2858	0.775028	NA
CG17105	0	NA	NA	NA	NA	NA
CG17107	0	NA	NA	NA	NA	NA
CG6415	0	NA	NA	NA	NA	NA
CG6431	0	NA	NA	NA	NA	NA
CG17118	0	NA	NA	NA	NA	NA
Vha16-5	0.282677	-0.09599	4.057934	-0.02366	0.981127	NA
Lip2	0	NA	NA	NA	NA	NA
Lip1	0.53416	-1.53481	4.012395	-0.38252	0.702078	NA
CG7300	0	NA	NA	NA	NA	NA
CG17108	2.377398	-1.86964	3.966533	-0.47135	0.637388	NA
CG7294	0	NA	NA	NA	NA	NA
CG7296	0	NA	NA	NA	NA	NA
CG7299	0	NA	NA	NA	NA	NA
CG7329	0	NA	NA	NA	NA	NA
CG7384	0.466186	0.959471	4.033986	0.237847	0.812	NA
CG17127	0	NA	NA	NA	NA	NA
CG31870	0	NA	NA	NA	NA	NA
CG6508	0.178053	-1.16467	4.075067	-0.2858	0.775028	NA
CG17134	0	NA	NA	NA	NA	NA
CG12517	0	NA	NA	NA	NA	NA
CG14072	33.11791	-1.7047	3.952066	-0.43134	0.666218	NA
lectin-33A	0	NA	NA	NA	NA	NA
Acp32CD	0	NA	NA	NA	NA	NA
CG14913	0	NA	NA	NA	NA	NA
CG18666	0.356107	-1.47099	4.037764	-0.36431	0.715628	NA
Art8	0	NA	NA	NA	NA	NA
aub	0.1047	0.613261	4.062799	0.150945	0.880019	NA
piwi	6.032582	-0.2788	3.849484	-0.07242	0.942264	NA
CG14069	0	NA	NA	NA	NA	NA
CG7309	1.046999	1.598136	3.993006	0.400234	0.688984	NA
CG14070	0	NA	NA	NA	NA	NA
CG14071	0	NA	NA	NA	NA	NA
aurB	0	NA	NA	NA	NA	NA
Porin2	0	NA	NA	NA	NA	NA
CG14915	0	NA	NA	NA	NA	NA
hgo	13.58927	0.289593	3.848482	0.075249	0.940017	NA
cmet	0.356107	-1.47099	4.037764	-0.36431	0.715628	NA
Vm32E	1.158217	-3.0773	3.989947	-0.77126	0.440551	NA
Gyc32E	0.954171	-1.65593	3.931926	-0.42115	0.673646	NA
CG31867	0	NA	NA	NA	NA	NA
RfC38	10.69005	1.338687	3.954459	0.338526	0.734967	NA
CG14921	2.720205	1.493763	3.963603	0.37687	0.70627	NA
AstCC	0.178053	-1.16467	4.075067	-0.2858	0.775028	NA
sala	0	NA	NA	NA	NA	NA
spz4	0.63886	-0.79045	3.986802	-0.19827	0.842837	NA
CG14930	0	NA	NA	NA	NA	NA

CG14926	0	NA	NA	NA	NA	NA
CG4983	0	NA	NA	NA	NA	NA
CG4988	0.396233	-1.6814	4.028351	-0.41739	0.676392	NA
Vha100-5	0.233093	0.692656	4.076246	0.169925	0.865069	NA
CG14931	0	NA	NA	NA	NA	NA
CG14932	0.236778	-0.26946	4.071573	-0.06618	0.947234	NA
Tsp33B	0.178053	-1.16467	4.075067	-0.2858	0.775028	NA
CG12307	0	NA	NA	NA	NA	NA
spag4	41.97228	-6.15664	3.864604	-1.59308	0.111141	NA
CG31704	8.480694	1.730894	3.956914	0.437435	0.661796	NA
CG14933	0	NA	NA	NA	NA	NA
Or33a	0.178053	-1.16467	4.075067	-0.2858	0.775028	NA
Or33b	0.1047	0.613261	4.062799	0.150945	0.880019	NA
Or33c	0	NA	NA	NA	NA	NA
Crys	0	NA	NA	NA	NA	NA
CG16964	0	NA	NA	NA	NA	NA
CG16965	0.712213	-1.57119	3.998613	-0.39293	0.694369	NA
Ada1-1	42.6194	7.055267	3.845109	1.834868	0.066525	NA
Rh5	30.29192	4.596794	3.802091	1.209017	0.226656	NA
dmGlut	0.104623	0.639075	4.067674	0.157111	0.875158	NA
CG18788	0	NA	NA	NA	NA	NA
RpL7-like	0	NA	NA	NA	NA	NA
CG14947	0	NA	NA	NA	NA	NA
Rab3-GAP	59.26679	-2.5013	3.768729	-0.6637	0.506883	NA
CG18787	22.48819	-3.74386	3.807439	-0.9833	0.325459	NA
CG18789	5.122163	0.359805	3.847332	0.093521	0.92549	NA
Mt2	10.36529	1.73509	3.955844	0.438614	0.660941	NA
CG17211	31.82862	3.548314	3.905524	0.908537	0.363594	NA
Gr33a	0	NA	NA	NA	NA	NA
CG31861	12.05115	1.374129	3.95414	0.347517	0.728203	NA
CG17217	0	NA	NA	NA	NA	NA
CG31862	0	NA	NA	NA	NA	NA
CG17010	0	NA	NA	NA	NA	NA
rho-6	0.4188	1.41068	4.034613	0.349644	0.726606	NA
Pkd2	0.3873	0.267327	4.008308	0.066693	0.946826	NA
Oatp33Eb	0.669976	0.241146	3.936344	0.061261	0.951151	NA
CR17024	0	NA	NA	NA	NA	NA
Ref2	25.27357	-3.15152	3.918373	-0.80429	0.421228	NA
CG5418	0	NA	NA	NA	NA	NA
CG5421	16.20522	-0.29634	3.93457	-0.07532	0.939963	NA
CG5435	0	NA	NA	NA	NA	NA
CG15483	0.356107	-1.47099	4.037764	-0.36431	0.715628	NA
CG15484	0	NA	NA	NA	NA	NA
CG5776	30.22693	1.683864	3.952538	0.426021	0.670093	NA
IFT43	0	NA	NA	NA	NA	NA
Nepl7	0.77483	0.786875	3.969371	0.198237	0.84286	NA
ACXB	0.233093	0.692656	4.076246	0.169925	0.865069	NA
ACXA	0	NA	NA	NA	NA	NA
ACXE	0	NA	NA	NA	NA	NA
CG16800	0	NA	NA	NA	NA	NA

Vha68-3	0.314023	1.768229	4.015745	0.440324	0.659702	NA
CG5122	4.402864	0.994202	3.785282	0.262649	0.792821	NA
Pk34A	0	NA	NA	NA	NA	NA
kmg	0.3873	0.267327	4.008308	0.066693	0.946826	NA
Rsph1	2.826131	4.274146	3.944545	1.083558	0.278561	NA
snRNA:U2:	2.601183	0.742986	3.858991	0.192534	0.847324	NA
CG16813	0	NA	NA	NA	NA	NA
CG16815	0	NA	NA	NA	NA	NA
Prosalpha6	0	NA	NA	NA	NA	NA
snRNA:U5:	0	NA	NA	NA	NA	NA
CG16820	0.63886	-0.79045	3.986802	-0.19827	0.842837	NA
Naa20B	0	NA	NA	NA	NA	NA
CG16825	0.6282	1.523021	4.015426	0.379293	0.704471	NA
CG16826	0	NA	NA	NA	NA	NA
CG31848	0.178053	-1.16467	4.075067	-0.2858	0.775028	NA
CG31730	0	NA	NA	NA	NA	NA
Mabi	0	NA	NA	NA	NA	NA
CG15480	0	NA	NA	NA	NA	NA
snRNA:U2:	0.547193	2.009611	4.015384	0.500478	0.616739	NA
CG15482	0	NA	NA	NA	NA	NA
CG9928	19.78829	1.744272	3.953518	0.441195	0.659072	NA
CG16848	0.816913	-0.91625	3.980578	-0.23018	0.817951	NA
Tehao	4.857655	-5.24295	3.926787	-1.33518	0.181819	NA
CG16852	0	NA	NA	NA	NA	NA
CG16853	10.6832	-1.6973	3.954478	-0.42921	0.66777	NA
CG9254	28.53219	0.249276	3.745282	0.066557	0.946934	NA
CG9263	0	NA	NA	NA	NA	NA
CG15638	1.675199	1.648777	3.978727	0.414398	0.678583	NA
CG16956	0.104623	0.639075	4.067674	0.157111	0.875158	NA
Vm34Ca	0	NA	NA	NA	NA	NA
CG10859	0.31387	1.261782	4.037145	0.312543	0.754628	NA
CG16957	14.0718	1.507016	3.953962	0.381141	0.703099	NA
CG18507	5.758419	2.904806	3.935248	0.738151	0.460423	NA
Gpo3	0.1047	0.613261	4.062799	0.150945	0.880019	NA
nur	28.05906	-1.66372	3.952239	-0.42096	0.673788	NA
asRNA:CR3	0	NA	NA	NA	NA	NA
CG7916	0.357625	-1.47707	4.044794	-0.36518	0.714979	NA
CG7953	0	NA	NA	NA	NA	NA
CG7968	0	NA	NA	NA	NA	NA
Ance	56.27327	-3.8721	3.653932	-1.05971	0.289277	NA
Ance-2	0	NA	NA	NA	NA	NA
Acyp	0	NA	NA	NA	NA	NA
Vajk3	0	NA	NA	NA	NA	NA
Vajk1	0	NA	NA	NA	NA	NA
CG8997	0.64106	-0.7867	4.009822	-0.19619	0.844459	NA
CG33306	0	NA	NA	NA	NA	NA
CG33307	10.81527	-3.13106	3.912235	-0.80032	0.423523	NA
PolG1	0	NA	NA	NA	NA	NA
CG16888	0	NA	NA	NA	NA	NA
CG9014	0	NA	NA	NA	NA	NA

NimB1	0	NA	NA	NA	NA	NA
NimB5	0	NA	NA	NA	NA	NA
bgm	0.178053	-1.16467	4.075067	-0.2858	0.775028	NA
CG31769	0	NA	NA	NA	NA	NA
CG15293	0	NA	NA	NA	NA	NA
CG18095	0	NA	NA	NA	NA	NA
lncRNA:CR	0	NA	NA	NA	NA	NA
NimC3	0	NA	NA	NA	NA	NA
tRNA:Gln-(	0.4188	1.41068	4.034613	0.349644	0.726606	NA
He	0	NA	NA	NA	NA	NA
NimB4	53.11742	0.647127	3.920236	0.165073	0.868886	NA
NimB2	0	NA	NA	NA	NA	NA
CG32971	0	NA	NA	NA	NA	NA
CG15286	0.282677	-0.09599	4.057934	-0.02366	0.981127	NA
Pgant35A	4.072211	1.397994	3.844884	0.363599	0.716158	NA
CG15283	0	NA	NA	NA	NA	NA
Cpr35B	0.466186	0.959471	4.033986	0.237847	0.812	NA
tRNA:Pro-/	0	NA	NA	NA	NA	NA
tRNA:Pro-/	0	NA	NA	NA	NA	NA
tRNA:Pro-/	0	NA	NA	NA	NA	NA
CG15282	0	NA	NA	NA	NA	NA
tRNA:Pro-/	0	NA	NA	NA	NA	NA
CG3473	5.025597	1.715508	3.960878	0.433113	0.664933	NA
tRNA:Gly-(	0	NA	NA	NA	NA	NA
tRNA:Gly-(	0	NA	NA	NA	NA	NA
tRNA:Gly-(	0	NA	NA	NA	NA	NA
tRNA:Gly-(	0.2094	1.005034	4.045341	0.248442	0.803792	NA
CG31835	0	NA	NA	NA	NA	NA
CG4691	0	NA	NA	NA	NA	NA
CSN1a	0.233093	0.692656	4.076246	0.169925	0.865069	NA
CG4701	0	NA	NA	NA	NA	NA
CG3491	0.178053	-1.16467	4.075067	-0.2858	0.775028	NA
ProtB	0	NA	NA	NA	NA	NA
ProtA	0	NA	NA	NA	NA	NA
CG4480	0	NA	NA	NA	NA	NA
CG15279	0.178053	-1.16467	4.075067	-0.2858	0.775028	NA
CG18420	0	NA	NA	NA	NA	NA
CG4650	0.178053	-1.16467	4.075067	-0.2858	0.775028	NA
CG31775	0	NA	NA	NA	NA	NA
yellow-c	5.647804	-1.09518	3.94024	-0.27795	0.781052	NA
CG15269	0	NA	NA	NA	NA	NA
ZnT35C	0	NA	NA	NA	NA	NA
tRNA:Leu-'	0	NA	NA	NA	NA	NA
CG31832	60.99536	1.537303	3.951498	0.389043	0.697244	NA
CG15263	0	NA	NA	NA	NA	NA
ms(2)35Ci	0	NA	NA	NA	NA	NA
CG15262	0	NA	NA	NA	NA	NA
CG4161	0.946065	-1.55144	3.957279	-0.39205	0.695022	NA
sna	0	NA	NA	NA	NA	NA
wor	0	NA	NA	NA	NA	NA

CG15258	0	NA	NA	NA	NA	NA
nht	0	NA	NA	NA	NA	NA
CG15260	17.70242	-2.26093	3.956273	-0.57148	0.567674	NA
CG15256	0.178812	-1.08808	4.061735	-0.26789	0.788788	NA
CG7631	0.356107	-1.47099	4.037764	-0.36431	0.715628	NA
CG18480	0	NA	NA	NA	NA	NA
CG18477	0	NA	NA	NA	NA	NA
CG18478	0	NA	NA	NA	NA	NA
CG31780	0	NA	NA	NA	NA	NA
CG31827	0	NA	NA	NA	NA	NA
CG31826	0	NA	NA	NA	NA	NA
Ku80	37.43781	-7.99376	3.910001	-2.04444	0.04091	NA
Gli	9.180649	5.322493	3.909097	1.361566	0.173335	NA
snRNA:U5:	0	NA	NA	NA	NA	NA
Or35a	0	NA	NA	NA	NA	NA
CG15253	0.178812	-1.08808	4.061735	-0.26789	0.788788	NA
CG15254	0	NA	NA	NA	NA	NA
Semp1	0.357625	-1.47707	4.044794	-0.36518	0.714979	NA
CG15255	0	NA	NA	NA	NA	NA
CG18109	0	NA	NA	NA	NA	NA
CG13243	0	NA	NA	NA	NA	NA
Tektin-A	0	NA	NA	NA	NA	NA
CR31824	0	NA	NA	NA	NA	NA
CG31735	0.1047	0.613261	4.062799	0.150945	0.880019	NA
CG13244	0	NA	NA	NA	NA	NA
CG10839	0.282677	-0.09599	4.057934	-0.02366	0.981127	NA
CG13245	0	NA	NA	NA	NA	NA
BicC	0	NA	NA	NA	NA	NA
CG31820	4.094408	-1.89106	3.960175	-0.47752	0.632993	NA
CG31821	0	NA	NA	NA	NA	NA
CG31823	0	NA	NA	NA	NA	NA
Tep1	0.460806	-0.60052	3.997252	-0.15023	0.880581	NA
CG7653	0	NA	NA	NA	NA	NA
CR31822	0	NA	NA	NA	NA	NA
CG18518	0	NA	NA	NA	NA	NA
CG4891	0	NA	NA	NA	NA	NA
CG4892	0	NA	NA	NA	NA	NA
CG31819	4.139081	-0.81368	3.784958	-0.21498	0.829786	NA
CG17329	0.104623	0.639075	4.067674	0.157111	0.875158	NA
Cyp303a1	0.178053	-1.16467	4.075067	-0.2858	0.775028	NA
fzy	0	NA	NA	NA	NA	NA
CG4440	0	NA	NA	NA	NA	NA
CG13258	0.1047	0.613261	4.062799	0.150945	0.880019	NA
Nepl8	1.238749	-0.26955	3.90623	-0.069	0.944986	NA
CG31816	0	NA	NA	NA	NA	NA
CG5968	27.36445	-4.7257	3.871879	-1.22052	0.222268	NA
ChLD3	0.178053	-1.16467	4.075067	-0.2858	0.775028	NA
Ugt37C2	0	NA	NA	NA	NA	NA
Ugt37E1	0	NA	NA	NA	NA	NA
CG6012	0	NA	NA	NA	NA	NA

CG31809	7.765206	-0.5101	3.950431	-0.12913	0.897258	NA
CG31810	0.132078	-1.13211	4.069918	-0.27817	0.780885	NA
Nepl9	0	NA	NA	NA	NA	NA
CG13282	8.794641	3.466039	3.919779	0.884243	0.376565	NA
CG13280	11.80165	0.084389	3.952695	0.02135	0.982967	NA
ppk17	1.179159	-1.16246	3.921297	-0.29645	0.766887	NA
glu	15.50206	4.397129	3.808727	1.154488	0.2483	NA
CG17996	0	NA	NA	NA	NA	NA
CG31807	7.203293	-3.22037	3.816344	-0.84384	0.39876	NA
Trpgamma	5.005669	-0.01925	3.950245	-0.00487	0.996112	NA
CG31743	0	NA	NA	NA	NA	NA
CG31806	21.80443	3.09609	3.881523	0.797648	0.425075	NA
CG15136	0	NA	NA	NA	NA	NA
CG6304	0.233093	0.692656	4.076246	0.169925	0.865069	NA
CG31805	0.233093	0.692656	4.076246	0.169925	0.865069	NA
CG32832	42.92525	-1.91992	3.951816	-0.48583	0.627085	NA
Gr36a	0	NA	NA	NA	NA	NA
Gr36b	0	NA	NA	NA	NA	NA
Gr36c	0	NA	NA	NA	NA	NA
CG31750	0	NA	NA	NA	NA	NA
CG31804	0	NA	NA	NA	NA	NA
CG6380	0	NA	NA	NA	NA	NA
CG31784	0	NA	NA	NA	NA	NA
CG5043	0	NA	NA	NA	NA	NA
CG5050	0	NA	NA	NA	NA	NA
Dhc36C	0.565506	-0.26224	3.986711	-0.06578	0.947554	NA
CG15147	0	NA	NA	NA	NA	NA
CG6870	0	NA	NA	NA	NA	NA
CG7094	0	NA	NA	NA	NA	NA
CG15145	36.38597	0.021996	3.730357	0.005897	0.995295	NA
CG15144	0.178053	-1.16467	4.075067	-0.2858	0.775028	NA
CG15143	0.668515	-2.46469	4.009802	-0.61467	0.538775	NA
CG15142	0	NA	NA	NA	NA	NA
qua	0	NA	NA	NA	NA	NA
CG18563	0	NA	NA	NA	NA	NA
SPH93	0.1047	0.613261	4.062799	0.150945	0.880019	NA
CG5681	0.2094	1.005034	4.045341	0.248442	0.803792	NA
Prosbeta5I	0	NA	NA	NA	NA	NA
CG5693	0	NA	NA	NA	NA	NA
CG31740	0	NA	NA	NA	NA	NA
CG31741	0	NA	NA	NA	NA	NA
Slc25A46b	0	NA	NA	NA	NA	NA
CG15152	35.70268	1.748887	3.952361	0.442492	0.658133	NA
CG15153	0.209323	1.414133	4.032409	0.350692	0.725819	NA
elfless	0	NA	NA	NA	NA	NA
btv	2.12201	-1.90008	3.94883	-0.48118	0.630392	NA
CG15155	0	NA	NA	NA	NA	NA
CG5790	0.62047	1.501254	3.987081	0.376529	0.706523	NA
CG31787	0	NA	NA	NA	NA	NA
CG31788	14.62664	1.617221	3.954103	0.408998	0.682541	NA

CG31802	0	NA	NA	NA	NA	NA
Ntf-2r	0.233093	0.692656	4.076246	0.169925	0.865069	NA
grnd	0	NA	NA	NA	NA	NA
CG10211	0	NA	NA	NA	NA	NA
CG31789	0	NA	NA	NA	NA	NA
IFT46	0	NA	NA	NA	NA	NA
amos	0	NA	NA	NA	NA	NA
CG10336	0	NA	NA	NA	NA	NA
CG15167	0	NA	NA	NA	NA	NA
CG17325	0	NA	NA	NA	NA	NA
Ugt36F1	0.2094	1.005034	4.045341	0.248442	0.803792	NA
Ugt36D1	47.69933	-3.09076	3.917316	-0.789	0.430112	NA
Ugt36E1	0.282753	-0.25398	4.020204	-0.06318	0.949626	NA
CG31752	0.53416	-1.53481	4.012395	-0.38252	0.702078	NA
CG10570	0	NA	NA	NA	NA	NA
ham	26.31472	2.744876	3.870991	0.709089	0.478269	NA
CG10348	0.411147	-0.29058	4.046973	-0.0718	0.94276	NA
CG15169	0	NA	NA	NA	NA	NA
CG31792	0	NA	NA	NA	NA	NA
robl37BC	0	NA	NA	NA	NA	NA
CG15172	0	NA	NA	NA	NA	NA
Phlpp	10.05119	1.734496	3.955995	0.438447	0.661062	NA
CG17343	0	NA	NA	NA	NA	NA
CG17344	0	NA	NA	NA	NA	NA
amd	36.32136	-1.91935	3.951981	-0.48567	0.627203	NA
CG10700	0	NA	NA	NA	NA	NA
CR33315	0	NA	NA	NA	NA	NA
CG17572	0	NA	NA	NA	NA	NA
CG10650	23.8341	1.243055	3.874244	0.320851	0.748323	NA
CG15170	0	NA	NA	NA	NA	NA
CG10750	0.178053	-1.16467	4.075067	-0.2858	0.775028	NA
CG13086	0	NA	NA	NA	NA	NA
CG17349	0.1047	0.613261	4.062799	0.150945	0.880019	NA
CG17350	0	NA	NA	NA	NA	NA
CG31798	0.892543	-2.83735	3.997814	-0.70972	0.477875	NA
CG13085	33.28358	-1.919	3.952078	-0.48557	0.627273	NA
CG17549	0	NA	NA	NA	NA	NA
fon	0.178053	-1.16467	4.075067	-0.2858	0.775028	NA
CG17564	0.466186	0.959471	4.033986	0.237847	0.812	NA
CG31797	18.93681	1.532509	3.952804	0.387702	0.698237	NA
gammaTul	0	NA	NA	NA	NA	NA
RanGAP	56.74728	2.975773	3.918927	0.759334	0.447653	NA
CG10026	0.356107	-1.47099	4.037764	-0.36431	0.715628	NA
CG13084	0	NA	NA	NA	NA	NA
CG31697	0.233093	0.692656	4.076246	0.169925	0.865069	NA
CG13082	0	NA	NA	NA	NA	NA
CG13081	0	NA	NA	NA	NA	NA
Tep4	0	NA	NA	NA	NA	NA
CG13078	0	NA	NA	NA	NA	NA
CG13077	0	NA	NA	NA	NA	NA

msb1l	0	NA	NA	NA	NA	NA
scw	0	NA	NA	NA	NA	NA
CG10463	27.97419	0.612097	3.75119	0.163174	0.870381	NA
snRNA:U4:	0.418493	1.313669	4.019283	0.326842	0.743788	NA
snRNA:U2:	0.388212	0.094116	4.038892	0.023303	0.981409	NA
snRNA:U5:	0	NA	NA	NA	NA	NA
CG16772	61.53668	0.359525	3.834959	0.093749	0.925308	NA
CG10680	5.15103	-1.89739	3.95832	-0.47934	0.631695	NA
OS9	0	NA	NA	NA	NA	NA
CG13965	0	NA	NA	NA	NA	NA
Victoria	0.732363	1.393579	3.993417	0.348969	0.727112	NA
CG10481	1.172943	-0.24009	3.970289	-0.06047	0.951781	NA
snRNA:U2:	4.471821	1.090965	3.845821	0.283675	0.776659	NA
CG13962	0	NA	NA	NA	NA	NA
snRNA:U5:	0	NA	NA	NA	NA	NA
fbp	47.31717	1.509425	3.951654	0.381973	0.702481	NA
neb	20.93999	1.744836	3.953376	0.441353	0.658957	NA
nesd	1.366752	-3.16859	3.965138	-0.79911	0.424225	NA
Kua	0	NA	NA	NA	NA	NA
CG10730	0	NA	NA	NA	NA	NA
AANATL3	0	NA	NA	NA	NA	NA
CG12617	0	NA	NA	NA	NA	NA
CG16798	45.34411	0.519731	3.879997	0.133951	0.893441	NA
Ugt37A1	0.178053	-1.16467	4.075067	-0.2858	0.775028	NA
CG10651	0.178053	-1.16467	4.075067	-0.2858	0.775028	NA
CG15475	0.3141	1.240997	4.03841	0.307299	0.758616	NA
CG17472	0	NA	NA	NA	NA	NA
CG31680	0	NA	NA	NA	NA	NA
CG18810	0	NA	NA	NA	NA	NA
CarT	3.94801	0.30349	3.956229	0.076712	0.938853	NA
CG9316	0	NA	NA	NA	NA	NA
phr6-4	0	NA	NA	NA	NA	NA
CG17470	0	NA	NA	NA	NA	NA
cad	3.737851	1.338807	3.960994	0.337998	0.735365	NA
CG31675	0.209247	1.042804	4.047837	0.25762	0.7967	NA
CG9270	0.1047	0.613261	4.062799	0.150945	0.880019	NA
Spn38F	0	NA	NA	NA	NA	NA
CheB38a	0	NA	NA	NA	NA	NA
CG33322	0.46073	-0.17853	4.00793	-0.04454	0.964471	NA
Itgbn	22.86169	-1.43517	3.743857	-0.38334	0.701467	NA
CG9259	0	NA	NA	NA	NA	NA
CG9257	0.209323	1.414133	4.032409	0.350692	0.725819	NA
CG31627	0.2094	1.005034	4.045341	0.248442	0.803792	NA
Mcm10	0.209323	1.414133	4.032409	0.350692	0.725819	NA
clumsy	0.2094	1.005034	4.045341	0.248442	0.803792	NA
snRNA:U4:	1.843226	-0.62009	3.962911	-0.15647	0.87566	NA
CG31988	0	NA	NA	NA	NA	NA
CG31624	0	NA	NA	NA	NA	NA
Ret	12.30175	-3.1116	3.915083	-0.79477	0.426746	NA
dimm	0.1047	0.613261	4.062799	0.150945	0.880019	NA

dtr	43.86831	-3.38172	3.9033	-0.86637	0.386285	NA
CG8665	4.491535	-2.50294	3.842178	-0.65144	0.514764	NA
Gr39b	0	NA	NA	NA	NA	NA
Cyp6t2Psi	5.594238	1.155882	3.959178	0.29195	0.770325	NA
Ir84a	0	NA	NA	NA	NA	NA
wa-cup	0.3141	1.240997	4.03841	0.307299	0.758616	NA
CG14598	0	NA	NA	NA	NA	NA
tRNA:Val-(	0	NA	NA	NA	NA	NA
alpha-Est2	0.53416	-1.53481	4.012395	-0.38252	0.702078	NA
Nlg1	0.178812	-1.08808	4.061735	-0.26789	0.788788	NA
alpha-Est3	0	NA	NA	NA	NA	NA
Rtnl2	0	NA	NA	NA	NA	NA
alpha-Est6	0.356107	-1.47099	4.037764	-0.36431	0.715628	NA
alpha-Est7	0	NA	NA	NA	NA	NA
Ubc84D	0.178053	-1.16467	4.075067	-0.2858	0.775028	NA
alpha-Est1	35.72998	1.790233	3.899643	0.459076	0.646179	NA
tRNA:Val-(	0	NA	NA	NA	NA	NA
tRNA:Val-(	0	NA	NA	NA	NA	NA
tRNA:Gly-T	57.31113	6.032411	3.83801	1.571755	0.116007	NA
CG31493	8.804864	-5.20569	3.890449	-1.33807	0.180874	NA
Sas-4	0	NA	NA	NA	NA	NA
sas	25.00866	-3.50752	3.901674	-0.89898	0.368664	NA
CG14609	21.51816	1.373109	3.872346	0.354593	0.722894	NA
thw	0	NA	NA	NA	NA	NA
CG14607	0	NA	NA	NA	NA	NA
CG10050	6.084524	-0.3706	3.954377	-0.09372	0.925333	NA
CR10032	0.178053	-1.16467	4.075067	-0.2858	0.775028	NA
RacGAP84	4.124062	-0.31346	3.915857	-0.08005	0.936199	NA
CG31496	0.178053	-1.16467	4.075067	-0.2858	0.775028	NA
nxf4	0	NA	NA	NA	NA	NA
Mst84Da	0	NA	NA	NA	NA	NA
Mst84Db	0	NA	NA	NA	NA	NA
Mst84Dc	0	NA	NA	NA	NA	NA
Mst84Dd	0	NA	NA	NA	NA	NA
CG17944	0	NA	NA	NA	NA	NA
CG10029	0	NA	NA	NA	NA	NA
Gld	0.46073	-0.17853	4.00793	-0.04454	0.964471	NA
Obp84a	0	NA	NA	NA	NA	NA
CG14605	0	NA	NA	NA	NA	NA
CG14606	0	NA	NA	NA	NA	NA
CG14610	0	NA	NA	NA	NA	NA
tRNA:Gly-T	0.313946	1.788224	4.01364	0.445537	0.655932	NA
Ama	0	NA	NA	NA	NA	NA
Dfd	39.33256	-2.64277	3.85029	-0.68638	0.492473	NA
tRNA:Lys-T	0	NA	NA	NA	NA	NA
tRNA:Lys-T	0	NA	NA	NA	NA	NA
7SLRNA:CF	0.132078	-1.13211	4.069918	-0.27817	0.780885	NA
ftz	0.132078	-1.13211	4.069918	-0.27817	0.780885	NA
Sodh-1	3.026906	-1.67104	3.963213	-0.42164	0.67329	NA
CG1979	0	NA	NA	NA	NA	NA

djl	0	NA	NA	NA	NA	NA
Mst84B	0	NA	NA	NA	NA	NA
tRNA:Lys-T	0	NA	NA	NA	NA	NA
tRNA:Lys-T	0	NA	NA	NA	NA	NA
tRNA:Lys-C	4.502097	1.711245	3.961986	0.431916	0.665803	NA
bcd	0.104623	0.639075	4.067674	0.157111	0.875158	NA
zen	0	NA	NA	NA	NA	NA
zen2	0	NA	NA	NA	NA	NA
CG15186	40.8306	-1.89128	3.897618	-0.48524	0.627505	NA
CG15185	0	NA	NA	NA	NA	NA
Pif2	0	NA	NA	NA	NA	NA
CG31482	0	NA	NA	NA	NA	NA
sunz	0.178053	-1.16467	4.075067	-0.2858	0.775028	NA
CG31286	0	NA	NA	NA	NA	NA
Ccp84Ag	0.178053	-1.16467	4.075067	-0.2858	0.775028	NA
Ccp84Ad	0.178812	-1.08808	4.061735	-0.26789	0.788788	NA
Ccp84Aa	0.178053	-1.16467	4.075067	-0.2858	0.775028	NA
Ccp84Ac	0	NA	NA	NA	NA	NA
Ccp84Ae	0	NA	NA	NA	NA	NA
Ccp84Af	0	NA	NA	NA	NA	NA
Ass	0	NA	NA	NA	NA	NA
CG15177	0	NA	NA	NA	NA	NA
sowi	0	NA	NA	NA	NA	NA
CG1138	0.178053	-1.16467	4.075067	-0.2858	0.775028	NA
CG1137	19.85974	-0.33589	3.76454	-0.08923	0.928902	NA
tRNA:Met-	3.664497	1.702077	3.964384	0.429342	0.667674	NA
Osi19	0	NA	NA	NA	NA	NA
Osi20	0	NA	NA	NA	NA	NA
CG17917	11.2266	-1.91107	3.954354	-0.48328	0.628896	NA
CG10298	6.207651	-1.90163	3.957084	-0.48056	0.630826	NA
CG17919	9.350061	-1.7818	3.954677	-0.45055	0.652311	NA
Hr83	0	NA	NA	NA	NA	NA
Neurochor	0.356107	-1.47099	4.037764	-0.36431	0.715628	NA
CG2616	0	NA	NA	NA	NA	NA
CG10919	0.1047	0.613261	4.062799	0.150945	0.880019	NA
CG10086	0.178053	-1.16467	4.075067	-0.2858	0.775028	NA
CG2678	0.104623	0.639075	4.067674	0.157111	0.875158	NA
lds	1.539911	0.274724	3.966541	0.06926	0.944782	NA
CG10445	0.282753	-0.25398	4.020204	-0.06318	0.949626	NA
CG18268	0	NA	NA	NA	NA	NA
CG3014	0.209323	1.414133	4.032409	0.350692	0.725819	NA
CG18744	9.081476	-2.90706	3.924892	-0.74067	0.458892	NA
CG18746	0	NA	NA	NA	NA	NA
CG18748	0	NA	NA	NA	NA	NA
CG18745	0	NA	NA	NA	NA	NA
CG18747	0	NA	NA	NA	NA	NA
CG11286	0	NA	NA	NA	NA	NA
Os-C	0	NA	NA	NA	NA	NA
CG7900	45.7174	-3.38716	3.903076	-0.86782	0.385494	NA
CG18249	10.96245	-1.91078	3.954436	-0.4832	0.628954	NA

CG15864	27.2242	-4.7603	3.866477	-1.23117	0.218258	NA
tRNA:Arg-/	0	NA	NA	NA	NA	NA
tRNA:Arg-/	0	NA	NA	NA	NA	NA
tRNA:Arg-/	0	NA	NA	NA	NA	NA
tRNA:Arg-/	0	NA	NA	NA	NA	NA
tRNA:Arg-/	0	NA	NA	NA	NA	NA
tRNA:Asn-(	1.072874	-2.0339	4.019685	-0.50598	0.612867	NA
ImpE3	0.660388	-1.75977	4.001266	-0.4398	0.660079	NA
CG11052	0.132078	-1.13211	4.069918	-0.27817	0.780885	NA
ELOVL	3.169864	-1.88225	3.962774	-0.47498	0.634799	NA
Mcm2	0	NA	NA	NA	NA	NA
ato	0.178053	-1.16467	4.075067	-0.2858	0.775028	NA
Nazo	0	NA	NA	NA	NA	NA
CG31464	0	NA	NA	NA	NA	NA
CG31463	0	NA	NA	NA	NA	NA
CG11698	0	NA	NA	NA	NA	NA
Or85a	0	NA	NA	NA	NA	NA
CG7443	0	NA	NA	NA	NA	NA
COX7AL	0.357625	-1.47707	4.044794	-0.36518	0.714979	NA
CG7352	14.3714	3.626327	3.843399	0.943521	0.345415	NA
CG7963	0	NA	NA	NA	NA	NA
Obp85a	0.4188	1.41068	4.034613	0.349644	0.726606	NA
Poxm	0	NA	NA	NA	NA	NA
CG11694	0	NA	NA	NA	NA	NA
CG11693	0	NA	NA	NA	NA	NA
CG11672	0.233093	0.692656	4.076246	0.169925	0.865069	NA
CG31454	0	NA	NA	NA	NA	NA
CG31259	0.178053	-1.16467	4.075067	-0.2858	0.775028	NA
Or85d	0	NA	NA	NA	NA	NA
CG31460	3.623677	4.272127	3.908648	1.092994	0.274397	NA
tRNA:Tyr-(	0	NA	NA	NA	NA	NA
tRNA:Tyr-(	0	NA	NA	NA	NA	NA
tRNA:Tyr-(	0	NA	NA	NA	NA	NA
Cyp313b1	1.024049	0.824862	3.952456	0.208696	0.834686	NA
tRNA:Tyr-(	0	NA	NA	NA	NA	NA
tRNA:Tyr-(	0	NA	NA	NA	NA	NA
Or85e	0	NA	NA	NA	NA	NA
Or85c	0.356866	-1.92326	4.037889	-0.4763	0.633858	NA
Or85b	0.178053	-1.16467	4.075067	-0.2858	0.775028	NA
CG8112	0	NA	NA	NA	NA	NA
CG11760	0.104623	0.639075	4.067674	0.157111	0.875158	NA
CG8136	0	NA	NA	NA	NA	NA
CG8223	33.69399	1.570012	3.740187	0.419768	0.674655	NA
CG8236	0	NA	NA	NA	NA	NA
Ir85a	3.347944	1.501875	3.961309	0.379136	0.704587	NA
tRNA:Arg-	0	NA	NA	NA	NA	NA
CG33189	2.097839	1.11873	3.972242	0.281637	0.778222	NA
CG33191	0.488184	-2.32943	4.022674	-0.57908	0.562538	NA
CG13318	0	NA	NA	NA	NA	NA
ranshi	20.1734	-0.0354	3.879381	-0.00912	0.99272	NA

CG33325	0	NA	NA	NA	NA	NA
CG11977	0	NA	NA	NA	NA	NA
Ada	0	NA	NA	NA	NA	NA
CG8420	11.065	-2.90387	3.761094	-0.77208	0.440066	NA
St2	0	NA	NA	NA	NA	NA
CR16735	0	NA	NA	NA	NA	NA
CG16736	0.6282	1.523021	4.015426	0.379293	0.704471	NA
HP1e	48.96414	4.500924	3.877655	1.160734	0.24575	NA
Srr	0	NA	NA	NA	NA	NA
CG8135	0	NA	NA	NA	NA	NA
CG16749	23.90606	-1.9174	3.952536	-0.48511	0.6276	NA
CG12951	0	NA	NA	NA	NA	NA
SpdS	39.69083	-3.50788	3.743041	-0.93717	0.34867	NA
Nepl12	0.713731	-2.6372	4.003426	-0.65874	0.510065	NA
nmdyn-D7	0	NA	NA	NA	NA	NA
CG18542	0	NA	NA	NA	NA	NA
CG32939	0	NA	NA	NA	NA	NA
alphaTub8	0.178053	-1.16467	4.075067	-0.2858	0.775028	NA
p24-2	0	NA	NA	NA	NA	NA
Unc-115b	0	NA	NA	NA	NA	NA
CG9444	0.356107	-1.47099	4.037764	-0.36431	0.715628	NA
CG9492	0.770937	-1.65034	3.976655	-0.41501	0.678137	NA
Fst	0.178812	-1.08808	4.061735	-0.26789	0.788788	NA
CG9427	10.99349	1.736178	3.955567	0.43892	0.660719	NA
CG31415	4.502097	1.711245	3.961986	0.431916	0.665803	NA
topi	0	NA	NA	NA	NA	NA
CG8534	0	NA	NA	NA	NA	NA
eloF	0	NA	NA	NA	NA	NA
CG9458	0	NA	NA	NA	NA	NA
CG16904	0	NA	NA	NA	NA	NA
Whamy	44.15721	-1.7056	3.951777	-0.4316	0.66603	NA
Rpt3R	0	NA	NA	NA	NA	NA
Spn85F	0	NA	NA	NA	NA	NA
Npc2e	0	NA	NA	NA	NA	NA
Npc2c	0	NA	NA	NA	NA	NA
ATPsyneps	0	NA	NA	NA	NA	NA
knk	0	NA	NA	NA	NA	NA
Alp13	2.617498	1.683176	3.969392	0.424039	0.671538	NA
Npc2d	0	NA	NA	NA	NA	NA
Fancl	0.356107	-1.47099	4.037764	-0.36431	0.715628	NA
CG31407	0	NA	NA	NA	NA	NA
CG5359	12.49498	4.852605	3.872106	1.253221	0.210125	NA
Gr85a	0	NA	NA	NA	NA	NA
CG12420	0	NA	NA	NA	NA	NA
CG3999	0.660388	-1.75977	4.001266	-0.4398	0.660079	NA
CG12592	13.45584	-5.37662	3.851703	-1.39591	0.162742	NA
CG31406	0	NA	NA	NA	NA	NA
tRNA:Ser-(	0	NA	NA	NA	NA	NA
mthl11	12.33805	-2.25201	3.958532	-0.5689	0.569424	NA
CG14684	0.1047	0.613261	4.062799	0.150945	0.880019	NA

CG4073	0	NA	NA	NA	NA	NA
Desi	0.2094	1.005034	4.045341	0.248442	0.803792	NA
Mcm5	25.65141	2.965217	3.921285	0.756185	0.449538	NA
CG4570	0	NA	NA	NA	NA	NA
CG14693	0.283512	-0.2331	4.065094	-0.05734	0.954273	NA
CG31272	0	NA	NA	NA	NA	NA
CG14691	0	NA	NA	NA	NA	NA
tomboy20	0	NA	NA	NA	NA	NA
CG14689	6.587971	-1.69066	3.956663	-0.42729	0.669165	NA
CG31467	0	NA	NA	NA	NA	NA
CG31391	0	NA	NA	NA	NA	NA
Tengl4	52.66406	1.750763	3.951894	0.443019	0.657752	NA
CG14696	22.71952	3.432757	3.897369	0.880788	0.378432	NA
mAcon2	0.890266	-1.59482	3.989917	-0.39971	0.689368	NA
Ugt302E1	0	NA	NA	NA	NA	NA
CG4757	0.356107	-1.47099	4.037764	-0.36431	0.715628	NA
Ugt303A1	0.411147	-0.29058	4.046973	-0.0718	0.94276	NA
CG18577	0	NA	NA	NA	NA	NA
CG4820	19.71815	4.689282	3.878268	1.209117	0.226618	NA
scpr-C	18.63659	1.743638	3.953678	0.441017	0.659201	NA
scpr-A	2.931598	1.690143	3.967537	0.425993	0.670113	NA
scpr-B	4.253697	2.01957	3.943775	0.512091	0.608588	NA
Ugt304A1	0	NA	NA	NA	NA	NA
Ugt35E1	0.178053	-1.16467	4.075067	-0.2858	0.775028	NA
PGRP-LB	1.885233	-1.25249	3.966236	-0.31579	0.752163	NA
sals	4.012275	0.045399	3.894374	0.011658	0.990699	NA
KP78a	0	NA	NA	NA	NA	NA
KP78b	0	NA	NA	NA	NA	NA
CG14708	0	NA	NA	NA	NA	NA
CG5342	0	NA	NA	NA	NA	NA
CG14710	37.35103	-2.6988	3.839693	-0.70287	0.482138	NA
CG14711	9.437583	-2.90821	3.924596	-0.74102	0.458681	NA
CG14718	0	NA	NA	NA	NA	NA
l-t	0	NA	NA	NA	NA	NA
CG14720	0	NA	NA	NA	NA	NA
CG18765	0.178053	-1.16467	4.075067	-0.2858	0.775028	NA
CG6813	53.92813	8.506755	3.908876	2.176266	0.029535	NA
Zaf1	23.63529	-2.82283	3.831658	-0.73671	0.461297	NA
CG6790	0	NA	NA	NA	NA	NA
sad	54.27657	1.679595	3.951813	0.425019	0.670823	NA
CG14731	0	NA	NA	NA	NA	NA
Hsp70Ab	22.35324	0.652205	3.746708	0.174074	0.861807	NA
aurA	0	NA	NA	NA	NA	NA
GC2	0	NA	NA	NA	NA	NA
CG10005	10.04383	1.526465	3.954462	0.386011	0.699489	NA
Hsp70Aa	0.670053	0.799805	3.963306	0.201803	0.840071	NA
Dnali1	0	NA	NA	NA	NA	NA
CG6962	38.17913	-0.50667	3.929428	-0.12894	0.897404	NA
ssp5	0	NA	NA	NA	NA	NA
tRNA:Lys-T	0	NA	NA	NA	NA	NA

Adk1	0.178053	-1.16467	4.075067	-0.2858	0.775028	NA
eIF3d2	0.2094	1.005034	4.045341	0.248442	0.803792	NA
CG4702	5.679341	-1.8997	3.957646	-0.48001	0.631222	NA
CG14740	0	NA	NA	NA	NA	NA
CG14739	0	NA	NA	NA	NA	NA
CG31358	0	NA	NA	NA	NA	NA
tRNA:Thr-T	0	NA	NA	NA	NA	NA
Rsbp15	1.883219	1.475262	3.968901	0.371705	0.710112	NA
CG3916	0	NA	NA	NA	NA	NA
CG3942	0	NA	NA	NA	NA	NA
svp	22.23288	-1.93366	3.736996	-0.51744	0.604851	NA
CG17738	0.178053	-1.16467	4.075067	-0.2858	0.775028	NA
CG4066	0	NA	NA	NA	NA	NA
CG10013	0.178812	-1.08808	4.061735	-0.26789	0.788788	NA
CG4115	0	NA	NA	NA	NA	NA
GstD4	0	NA	NA	NA	NA	NA
GstD5	6.095603	1.339992	3.956027	0.338722	0.734819	NA
GstD6	0	NA	NA	NA	NA	NA
GstD7	0	NA	NA	NA	NA	NA
GstD8	0	NA	NA	NA	NA	NA
GstD11	0	NA	NA	NA	NA	NA
CG33098	0	NA	NA	NA	NA	NA
Desat2	0.178053	-1.16467	4.075067	-0.2858	0.775028	NA
CG10035	31.82878	1.748183	3.952537	0.442294	0.658277	NA
GstD10	54.03022	-0.32344	3.836432	-0.08431	0.932813	NA
Tim17a1	0.356107	-1.47099	4.037764	-0.36431	0.715628	NA
CG10041	0.712213	-1.57119	3.998613	-0.39293	0.694369	NA
tRNA:Thr-T	0	NA	NA	NA	NA	NA
Cyp313a3	0	NA	NA	NA	NA	NA
Cyp313a2	0	NA	NA	NA	NA	NA
Cyp313a5	0	NA	NA	NA	NA	NA
Pglym87	0	NA	NA	NA	NA	NA
CG17404	0.178812	-1.08808	4.061735	-0.26789	0.788788	NA
CG4860	0	NA	NA	NA	NA	NA
CG4830	0.178812	-1.08808	4.061735	-0.26789	0.788788	NA
Hsp70Bbb	2.615581	1.492057	3.964088	0.376393	0.706624	NA
Hsp70Bc	36.78058	0.766546	3.835252	0.199868	0.841583	NA
Hug	16.19714	1.629148	3.953821	0.412044	0.680307	NA
CG18530	0	NA	NA	NA	NA	NA
CG11598	0.233093	0.692656	4.076246	0.169925	0.865069	NA
CG11600	0	NA	NA	NA	NA	NA
CG11608	0	NA	NA	NA	NA	NA
CG6225	0	NA	NA	NA	NA	NA
CG14395	9.548171	1.40437	3.954332	0.355147	0.722479	NA
Gnmt	0	NA	NA	NA	NA	NA
NijC	0.356107	-1.47099	4.037764	-0.36431	0.715628	NA
Ugt37A2	0	NA	NA	NA	NA	NA
Ugt37A3	0.233093	0.692656	4.076246	0.169925	0.865069	NA
CG14391	0	NA	NA	NA	NA	NA
CG31347	20.06734	0.235201	3.843993	0.061187	0.951211	NA

Cyp313a4	0	NA	NA	NA	NA	NA
CG6753	0	NA	NA	NA	NA	NA
lncRNA:alp	14.38293	1.72461	3.764217	0.458159	0.646838	NA
Hsp70Ba	0.774906	0.100364	3.977538	0.025233	0.979869	NA
CG17207	0	NA	NA	NA	NA	NA
d-cup	0	NA	NA	NA	NA	NA
CG10909	0	NA	NA	NA	NA	NA
Cyp304a1	0	NA	NA	NA	NA	NA
CG7091	12.45929	1.738298	3.955028	0.439516	0.660288	NA
ry	1.700385	-0.69042	3.913484	-0.17642	0.859963	NA
CG11668	1.602479	-1.64094	3.97352	-0.41297	0.67963	NA
CG11670	0	NA	NA	NA	NA	NA
CG31157	0	NA	NA	NA	NA	NA
sim	56.00094	-1.92067	3.951604	-0.48605	0.626933	NA
timeout	35.4428	-3.51547	3.90037	-0.90132	0.367419	NA
snk	0	NA	NA	NA	NA	NA
CG17327	0	NA	NA	NA	NA	NA
yellow-f2	0.53416	-1.53481	4.012395	-0.38252	0.702078	NA
yellow-f	0.178812	-1.08808	4.061735	-0.26789	0.788788	NA
CG14383	0.64106	-0.7867	4.009822	-0.19619	0.844459	NA
CG14384	0	NA	NA	NA	NA	NA
CG8138	0	NA	NA	NA	NA	NA
CG14380	0	NA	NA	NA	NA	NA
CG8141	0	NA	NA	NA	NA	NA
CG8630	0	NA	NA	NA	NA	NA
Osi22	0.536437	-1.71175	4.038042	-0.42391	0.671635	NA
CG8773	0.356107	-1.47099	4.037764	-0.36431	0.715628	NA
CG8774	0.806253	0.654822	3.981166	0.16448	0.869353	NA
mthl12	0.178053	-1.16467	4.075067	-0.2858	0.775028	NA
CheA87a	0	NA	NA	NA	NA	NA
CG8870	0	NA	NA	NA	NA	NA
CG14377	0	NA	NA	NA	NA	NA
yellow-e	0	NA	NA	NA	NA	NA
yellow-e2	0	NA	NA	NA	NA	NA
yellow-e3	0	NA	NA	NA	NA	NA
CG14374	0	NA	NA	NA	NA	NA
CG9799	58.9733	-0.20209	3.836286	-0.05268	0.957989	NA
Lip3	0	NA	NA	NA	NA	NA
wntD	0	NA	NA	NA	NA	NA
apn	0	NA	NA	NA	NA	NA
TBC1D5	0	NA	NA	NA	NA	NA
CG8476	0	NA	NA	NA	NA	NA
CG8508	0	NA	NA	NA	NA	NA
Ir87a	0.178812	-1.08808	4.061735	-0.26789	0.788788	NA
Act87E	0.282753	-0.25398	4.020204	-0.06318	0.949626	NA
CG14370	0	NA	NA	NA	NA	NA
CG14369	0	NA	NA	NA	NA	NA
CG31337	0	NA	NA	NA	NA	NA
CG12538	0	NA	NA	NA	NA	NA
asRNA:CR1	7.098664	-3.99432	3.837261	-1.04093	0.297908	NA

CG9269	0	NA	NA	NA	NA	NA
CG10841	0	NA	NA	NA	NA	NA
CG14367	50.74518	-1.70595	3.951664	-0.4317	0.665957	NA
CG9312	43.03167	1.749869	3.952114	0.442768	0.657934	NA
Cht5	42.92689	2.972935	3.919558	0.758488	0.448159	NA
CG9297	0.461489	-1.26887	4.028229	-0.31499	0.752766	NA
CG9288	0	NA	NA	NA	NA	NA
tRNA:Phe-	0	NA	NA	NA	NA	NA
tRNA:Phe-	0.1047	0.613261	4.062799	0.150945	0.880019	NA
Rbp4	0.178053	-1.16467	4.075067	-0.2858	0.775028	NA
CG9757	0	NA	NA	NA	NA	NA
CG9759	0.178053	-1.16467	4.075067	-0.2858	0.775028	NA
ems	5.905203	1.274247	3.957034	0.322021	0.747437	NA
CG31517	0	NA	NA	NA	NA	NA
Orc2	0.282753	-0.25398	4.020204	-0.06318	0.949626	NA
CG9925	0.74894	0.958264	3.97722	0.240938	0.809603	NA
CG9926	0.178053	-1.16467	4.075067	-0.2858	0.775028	NA
Art6	0.356866	-1.92326	4.037889	-0.4763	0.633858	NA
Art9	0.590718	-0.57492	4.003363	-0.14361	0.885809	NA
E5	0.2094	1.005034	4.045341	0.248442	0.803792	NA
CG14362	0	NA	NA	NA	NA	NA
CG31495	0.314023	1.768229	4.015745	0.440324	0.659702	NA
Or88a	0	NA	NA	NA	NA	NA
CG3199	0	NA	NA	NA	NA	NA
CG9616	0	NA	NA	NA	NA	NA
CG9624	0	NA	NA	NA	NA	NA
CG9631	4.095225	-1.68037	3.96008	-0.42433	0.671327	NA
Sp212	0	NA	NA	NA	NA	NA
CG33109	0	NA	NA	NA	NA	NA
CG31326	0.264155	-1.54099	4.048976	-0.38059	0.703509	NA
CG31327	0.411147	-0.29058	4.046973	-0.0718	0.94276	NA
CG31533	0.282753	-0.25398	4.020204	-0.06318	0.949626	NA
CG9722	0.178053	-1.16467	4.075067	-0.2858	0.775028	NA
CG14355	0.132078	-1.13211	4.069918	-0.27817	0.780885	NA
CG14356	0	NA	NA	NA	NA	NA
Kif19A	0.442209	-2.31142	4.024999	-0.57427	0.565788	NA
CG14357	0.104623	0.639075	4.067674	0.157111	0.875158	NA
CG9920	0.178053	-1.16467	4.075067	-0.2858	0.775028	NA
tRNA:Ser-(	0	NA	NA	NA	NA	NA
CG14839	0.178812	-1.08808	4.061735	-0.26789	0.788788	NA
IFT54	0	NA	NA	NA	NA	NA
CG31321	0	NA	NA	NA	NA	NA
soti	0	NA	NA	NA	NA	NA
CG14841	0	NA	NA	NA	NA	NA
CG14840	0	NA	NA	NA	NA	NA
CG14850	0	NA	NA	NA	NA	NA
CG14851	0.1047	0.613261	4.062799	0.150945	0.880019	NA
CG14852	0	NA	NA	NA	NA	NA
Cad88C	0.233093	0.692656	4.076246	0.169925	0.865069	NA
BigH1	0	NA	NA	NA	NA	NA

Rad17	0	NA	NA	NA	NA	NA
CG7886	0	NA	NA	NA	NA	NA
Cys	0.536437	-1.71175	4.038042	-0.42391	0.671635	NA
CG8087	0	NA	NA	NA	NA	NA
CG14854	0	NA	NA	NA	NA	NA
CG14855	14.55965	-5.38613	3.885812	-1.3861	0.165716	NA
CG14856	0	NA	NA	NA	NA	NA
CG14857	13.74906	6.274049	3.852454	1.628585	0.103401	NA
CG3610	2.202539	2.272562	3.954673	0.574652	0.565526	NA
CG33332	0.357625	-1.47707	4.044794	-0.36518	0.714979	NA
CG14860	0.2094	1.005034	4.045341	0.248442	0.803792	NA
RpS5b	0.1047	0.613261	4.062799	0.150945	0.880019	NA
CG7362	0.460806	-0.60052	3.997252	-0.15023	0.880581	NA
CG14861	0	NA	NA	NA	NA	NA
RpL10Aa	0	NA	NA	NA	NA	NA
CG3984	0	NA	NA	NA	NA	NA
Mf	42.27433	5.351597	3.865168	1.38457	0.166184	NA
CG6974	0	NA	NA	NA	NA	NA
CG4210	57.16616	1.751077	3.951817	0.443107	0.657689	NA
CG4338	22.83796	8.258528	3.846797	2.146858	0.031805	NA
CG14866	0	NA	NA	NA	NA	NA
CG6118	0.356107	-1.47099	4.037764	-0.36431	0.715628	NA
CG6125	24.76767	-0.60444	3.837471	-0.15751	0.874843	NA
h-cup	11.21736	-1.69782	3.954309	-0.42936	0.667662	NA
l(3)neo43	45.17854	0.122833	3.408704	0.036035	0.971254	NA
Amt	0	NA	NA	NA	NA	NA
Spn88Eb	0.712213	-1.57119	3.998613	-0.39293	0.694369	NA
Act88F	0.1047	0.613261	4.062799	0.150945	0.880019	NA
B9d1	0	NA	NA	NA	NA	NA
CG5213	0	NA	NA	NA	NA	NA
AOX4	0.460806	-0.60052	3.997252	-0.15023	0.880581	NA
AOX2	0	NA	NA	NA	NA	NA
CG31296	0	NA	NA	NA	NA	NA
Trissin	6.041173	2.443859	3.932681	0.621423	0.534321	NA
CG31446	0	NA	NA	NA	NA	NA
CG5399	0.178053	-1.16467	4.075067	-0.2858	0.775028	NA
CG4520	0	NA	NA	NA	NA	NA
CG12784	0	NA	NA	NA	NA	NA
Ttc26	0	NA	NA	NA	NA	NA
CG5614	0	NA	NA	NA	NA	NA
Uros2	0	NA	NA	NA	NA	NA
CG9593	1.150856	1.439869	3.979295	0.36184	0.717471	NA
CG4576	0	NA	NA	NA	NA	NA
spn-E	0	NA	NA	NA	NA	NA
CG9632	0.178812	-1.08808	4.061735	-0.26789	0.788788	NA
Pbp45	1.465799	1.63603	3.98226	0.410829	0.681198	NA
rec	0	NA	NA	NA	NA	NA
c(3)G	16.59954	6.419444	3.899614	1.646174	0.099728	NA
CG5623	0	NA	NA	NA	NA	NA
CG31294	0.1047	0.613261	4.062799	0.150945	0.880019	NA

CR31292	0	NA	NA	NA	NA	NA
Fbxl7	22.37695	1.173937	3.953007	0.296973	0.766487	NA
GATAe	0	NA	NA	NA	NA	NA
pnr	0.233093	0.692656	4.076246	0.169925	0.865069	NA
CG10264	0	NA	NA	NA	NA	NA
CG32855	0	NA	NA	NA	NA	NA
CG10185	0	NA	NA	NA	NA	NA
IKKbeta	4.638391	-1.96453	3.907524	-0.50276	0.615136	NA
CG10407	24.04478	-5.41448	3.873955	-1.39766	0.162215	NA
CG31088	0	NA	NA	NA	NA	NA
CG5478	0	NA	NA	NA	NA	NA
CG14877	26.00759	1.607857	3.952694	0.406775	0.684173	NA
Sb	0	NA	NA	NA	NA	NA
CG8925	0	NA	NA	NA	NA	NA
Zip89B	0	NA	NA	NA	NA	NA
tRNA:Phe-	8.391356	1.163791	3.956459	0.29415	0.768644	NA
tRNA:Val-/	18.64746	1.172702	3.953425	0.296629	0.76675	NA
CG6901	0	NA	NA	NA	NA	NA
CG17929	0	NA	NA	NA	NA	NA
CG17930	0.178053	-1.16467	4.075067	-0.2858	0.775028	NA
CG31279	1.465799	1.63603	3.98226	0.410829	0.681198	NA
tRNA:Thr-/	0	NA	NA	NA	NA	NA
CG10405	19.58586	-1.70224	3.952863	-0.43064	0.666733	NA
Gyc89Da	0	NA	NA	NA	NA	NA
Gyc89Db	13.0246	0.398469	3.843263	0.10368	0.917423	NA
CG14891	0.356866	-1.92326	4.037889	-0.4763	0.633858	NA
Decay	4.112682	-2.19841	3.972482	-0.55341	0.579983	NA
Cad89D	0.132078	-1.13211	4.069918	-0.27817	0.780885	NA
tRNA:Thr-/	0	NA	NA	NA	NA	NA
ss	19.13148	0.080845	3.833637	0.021088	0.983175	NA
CG10317	0.178812	-1.08808	4.061735	-0.26789	0.788788	NA
CG14880	0	NA	NA	NA	NA	NA
CG14892	2.745815	3.964215	3.942889	1.005409	0.3147	NA
NPF	29.71301	1.535031	3.952119	0.388407	0.697715	NA
CG17562	0	NA	NA	NA	NA	NA
CG17560	0	NA	NA	NA	NA	NA
CG14893	0	NA	NA	NA	NA	NA
Glut3	0	NA	NA	NA	NA	NA
lncRNA:bx	0.3141	1.240997	4.03841	0.307299	0.758616	NA
CG31275	0	NA	NA	NA	NA	NA
Ubx	0.178053	-1.16467	4.075067	-0.2858	0.775028	NA
modSP	49.35567	1.672616	3.951898	0.423244	0.672117	NA
CG14905	0.178053	-1.16467	4.075067	-0.2858	0.775028	NA
CG10345	0	NA	NA	NA	NA	NA
VhaM9.7-c	0	NA	NA	NA	NA	NA
MESK4	11.12828	-2.88421	3.758206	-0.76744	0.442818	NA
CG8907	15.71724	-1.91446	3.953377	-0.48426	0.628201	NA
Actn3	0	NA	NA	NA	NA	NA
Abd-B	0.236778	-0.26946	4.071573	-0.06618	0.947234	NA
abd-A	0.1047	0.613261	4.062799	0.150945	0.880019	NA

CG5225	0	NA	NA	NA	NA	NA
CG31268	0	NA	NA	NA	NA	NA
CG31269	0	NA	NA	NA	NA	NA
CG31265	0	NA	NA	NA	NA	NA
CG31267	0.6282	1.523021	4.015426	0.379293	0.704471	NA
CG5246	0	NA	NA	NA	NA	NA
CG5255	0	NA	NA	NA	NA	NA
CG5265	0	NA	NA	NA	NA	NA
CG31262	0	NA	NA	NA	NA	NA
TwdlW	0.178053	-1.16467	4.075067	-0.2858	0.775028	NA
CG4053	0	NA	NA	NA	NA	NA
CG17475	0.178053	-1.16467	4.075067	-0.2858	0.775028	NA
CG17477	0	NA	NA	NA	NA	NA
CG18213	7.741737	1.18143	3.903954	0.302624	0.762177	NA
CG4009	0	NA	NA	NA	NA	NA
CG31418	0	NA	NA	NA	NA	NA
CG31419	0	NA	NA	NA	NA	NA
CG17283	0	NA	NA	NA	NA	NA
CG5860	0	NA	NA	NA	NA	NA
CG5863	0	NA	NA	NA	NA	NA
CG5866	0	NA	NA	NA	NA	NA
CG33333	0	NA	NA	NA	NA	NA
cysu	1.06832	-1.61145	3.983918	-0.40449	0.685853	NA
CG14330	1.398559	1.091484	3.982137	0.274095	0.784011	NA
Eh	0	NA	NA	NA	NA	NA
CG14331	0	NA	NA	NA	NA	NA
CG14332	0	NA	NA	NA	NA	NA
P5cr-2	0	NA	NA	NA	NA	NA
Sgs5bis	0	NA	NA	NA	NA	NA
Sgs5	0	NA	NA	NA	NA	NA
CG14327	0	NA	NA	NA	NA	NA
tRNA:Val-(	0	NA	NA	NA	NA	NA
CG14326	0	NA	NA	NA	NA	NA
tRNA:Ala-/	0.233093	0.692656	4.076246	0.169925	0.865069	NA
tRNA:Pro-7	0	NA	NA	NA	NA	NA
AttD	0	NA	NA	NA	NA	NA
CG14325	0	NA	NA	NA	NA	NA
tRNA:Pro-7	0	NA	NA	NA	NA	NA
tRNA:Pro-7	0	NA	NA	NA	NA	NA
tRNA:Ala-/	0	NA	NA	NA	NA	NA
tRNA:Ala-/	0	NA	NA	NA	NA	NA
tRNA:Ala-/	0	NA	NA	NA	NA	NA
tRNA:Ala-/	0	NA	NA	NA	NA	NA
tRNA:Ala-/	0	NA	NA	NA	NA	NA
tRNA:Ala-/	0	NA	NA	NA	NA	NA
tRNA:Ala-/	0	NA	NA	NA	NA	NA
tRNA:Val-/	0	NA	NA	NA	NA	NA
tRNA:Thr-/	0	NA	NA	NA	NA	NA
tRNA:Thr-/	0	NA	NA	NA	NA	NA
CG14323	0	NA	NA	NA	NA	NA

tRNA:Ala-/	0	NA	NA	NA	NA	NA
CG14324	0	NA	NA	NA	NA	NA
tRNA:Pro-⁻	0.178812	-1.08808	4.061735	-0.26789	0.788788	NA
Edg91	0	NA	NA	NA	NA	NA
CG12347	0.104623	0.639075	4.067674	0.157111	0.875158	NA
CG17801	0.71449	-2.47883	4.014121	-0.61753	0.536887	NA
Atg8b	0	NA	NA	NA	NA	NA
CG7794	0	NA	NA	NA	NA	NA
CG14317	0	NA	NA	NA	NA	NA
tRNA:Gly-1	0.702005	1.501538	3.971695	0.37806	0.705386	NA
tRNA:Gly-1	0	NA	NA	NA	NA	NA
CG14316	0	NA	NA	NA	NA	NA
CG14315	0	NA	NA	NA	NA	NA
CG18598	0	NA	NA	NA	NA	NA
CG18599	0.233093	0.692656	4.076246	0.169925	0.865069	NA
hmw	0	NA	NA	NA	NA	NA
lncRNA:CR	0	NA	NA	NA	NA	NA
CG7675	0	NA	NA	NA	NA	NA
WRNexo	0	NA	NA	NA	NA	NA
CG7126	0.178053	-1.16467	4.075067	-0.2858	0.775028	NA
CG7142	0.310131	-1.94616	4.045058	-0.48112	0.630431	NA
CG14313	0	NA	NA	NA	NA	NA
cona	0	NA	NA	NA	NA	NA
CG7691	0	NA	NA	NA	NA	NA
CG14305	0.178053	-1.16467	4.075067	-0.2858	0.775028	NA
CG14304	0.732669	2.681564	3.986192	0.672713	0.50113	NA
CG10864	0.894061	-2.00006	4.030294	-0.49626	0.619714	NA
Vha100-4	0.282753	-0.25398	4.020204	-0.06318	0.949626	NA
CG31231	0.178053	-1.16467	4.075067	-0.2858	0.775028	NA
CG31230	0	NA	NA	NA	NA	NA
CG31226	0	NA	NA	NA	NA	NA
CG7720	4.416522	-2.34843	3.828177	-0.61346	0.539573	NA
CG7715	47.41589	-1.92023	3.95173	-0.48592	0.627024	NA
CG7714	0.236701	-0.24276	4.020028	-0.06039	0.951846	NA
Muc91C	0.638783	-0.20822	3.989517	-0.05219	0.958375	NA
CG14301	30.07346	-6.25491	3.869434	-1.61649	0.105988	NA
tRNA:Pro-⁻	0.236778	-0.26946	4.071573	-0.06618	0.947234	NA
CG7695	0	NA	NA	NA	NA	NA
CG14298	0.132078	-1.13211	4.069918	-0.27817	0.780885	NA
CG14297	0	NA	NA	NA	NA	NA
Rh2	0	NA	NA	NA	NA	NA
CG14286	0	NA	NA	NA	NA	NA
CG6026	0.488184	-2.32943	4.022674	-0.57908	0.562538	NA
CG14292	0.836986	1.408818	3.9887	0.353202	0.723937	NA
CG14294	0.31387	1.261782	4.037145	0.312543	0.754628	NA
Cyp12a5	37.02758	-2.99414	3.905974	-0.76655	0.443347	NA
nos	0.233093	0.692656	4.076246	0.169925	0.865069	NA
CG14282	0	NA	NA	NA	NA	NA
Gdn1	0.86242	-0.6641	3.93138	-0.16892	0.865858	NA
CG14280	1.450416	2.221915	3.921545	0.566592	0.570992	NA

CG5217	0.178053	-1.16467	4.075067	-0.2858	0.775028	NA
CG5250	0.178053	-1.16467	4.075067	-0.2858	0.775028	NA
CG11703	11.93579	1.737599	3.955206	0.439319	0.66043	NA
CG3734	0	NA	NA	NA	NA	NA
CG18493	32.61202	0.131047	3.948353	0.03319	0.973523	NA
CG3739	0	NA	NA	NA	NA	NA
CG15025	51.15212	2.397978	3.919834	0.611755	0.5407	NA
Scsalpha2	0	NA	NA	NA	NA	NA
CG3517	49.27478	-3.16815	3.905502	-0.8112	0.417249	NA
CG31244	0	NA	NA	NA	NA	NA
CG31245	0.178053	-1.16467	4.075067	-0.2858	0.775028	NA
CG31404	0	NA	NA	NA	NA	NA
CG3581	0.53416	-1.53481	4.012395	-0.38252	0.702078	NA
CG7333	0	NA	NA	NA	NA	NA
CG7342	0.46073	-0.17853	4.00793	-0.04454	0.964471	NA
CG17752	0.356107	-1.47099	4.037764	-0.36431	0.715628	NA
CG16727	0.356107	-1.47099	4.037764	-0.36431	0.715628	NA
CG7432	3.218621	-2.17835	3.977872	-0.54762	0.583954	NA
tRNA:Val-(	0	NA	NA	NA	NA	NA
tRNA:Ala-/	0	NA	NA	NA	NA	NA
tRNA:Thr-/	0	NA	NA	NA	NA	NA
CG11659	0.715249	-1.88064	4.034336	-0.46616	0.641102	NA
CG6300	0	NA	NA	NA	NA	NA
tRNA:Val-(	0	NA	NA	NA	NA	NA
CG31459	0	NA	NA	NA	NA	NA
CG4770	0	NA	NA	NA	NA	NA
CG4854	2.81157	-0.71279	3.905405	-0.18251	0.855179	NA
CG17190	0	NA	NA	NA	NA	NA
CG4465	0.53416	-1.53481	4.012395	-0.38252	0.702078	NA
Xport-A	0	NA	NA	NA	NA	NA
CG17186	1.891372	-1.25233	3.893828	-0.32162	0.747742	NA
CG10887	43.71221	0.015847	3.93167	0.004031	0.996784	NA
Gr92a	0	NA	NA	NA	NA	NA
CG17199	3.962585	1.146346	3.962484	0.2893	0.772352	NA
CG31206	0	NA	NA	NA	NA	NA
Ir92a	0	NA	NA	NA	NA	NA
MtnC	0	NA	NA	NA	NA	NA
eIF3g2	0	NA	NA	NA	NA	NA
Or92a	0	NA	NA	NA	NA	NA
MtnB	0	NA	NA	NA	NA	NA
Dic2	0	NA	NA	NA	NA	NA
CG4335	0	NA	NA	NA	NA	NA
CG4362	0	NA	NA	NA	NA	NA
CG4367	0	NA	NA	NA	NA	NA
MtnD	5.594238	1.155882	3.959178	0.29195	0.770325	NA
Cpr92F	0.589959	-1.15593	4.01641	-0.2878	0.773499	NA
CG31205	0	NA	NA	NA	NA	NA
CG31200	59.5122	1.435774	3.951593	0.36334	0.716351	NA
CG17267	0	NA	NA	NA	NA	NA
CG15923	0.4188	1.41068	4.034613	0.349644	0.726606	NA

tRNA:Leu-'	0	NA	NA	NA	NA	NA
CG15695	0	NA	NA	NA	NA	NA
CG15696	0	NA	NA	NA	NA	NA
TotA	4.761425	1.868764	3.77732	0.494733	0.620789	NA
TotC	0	NA	NA	NA	NA	NA
TotB	0	NA	NA	NA	NA	NA
TotZ	0	NA	NA	NA	NA	NA
tRNA:Thr-(	0	NA	NA	NA	NA	NA
tRNA:Thr-(	0	NA	NA	NA	NA	NA
tRNA:Thr-(	0.2094	1.005034	4.045341	0.248442	0.803792	NA
Grik	3.978597	1.705937	3.963372	0.430426	0.666886	NA
TotX	0	NA	NA	NA	NA	NA
Takl1	0	NA	NA	NA	NA	NA
CG5697	0	NA	NA	NA	NA	NA
CG12278	4.622719	-1.89457	3.959145	-0.47853	0.632273	NA
dmrt93B	0.178812	-1.08808	4.061735	-0.26789	0.788788	NA
rtet	7.478238	-1.69271	3.955987	-0.42789	0.668735	NA
Obp93a	0	NA	NA	NA	NA	NA
CG7044	58.97275	0.361757	3.834322	0.094347	0.924833	NA
HHEX	23.3503	-0.87126	3.949683	-0.22059	0.825412	NA
r-l	50.514	0.139459	3.306955	0.042171	0.966362	NA
CG7079	13.48854	0.426445	3.850007	0.110765	0.911803	NA
CG31207	38.3485	-3.51693	3.900132	-0.90175	0.367191	NA
CG31189	14.52855	-1.91377	3.953578	-0.48406	0.628344	NA
Dhc93AB	2.799008	-3.41842	3.863738	-0.88474	0.376295	NA
Alp5	11.88699	-1.91173	3.954165	-0.48347	0.628761	NA
CG5892	0	NA	NA	NA	NA	NA
CG17298	0	NA	NA	NA	NA	NA
tin	0.209247	1.042804	4.047837	0.25762	0.7967	NA
bap	0.104623	0.639075	4.067674	0.157111	0.875158	NA
CG7907	0	NA	NA	NA	NA	NA
lbe	0	NA	NA	NA	NA	NA
lbl	1.602479	-1.64094	3.97352	-0.41297	0.67963	NA
CG3301	0	NA	NA	NA	NA	NA
CG15497	0.178053	-1.16467	4.075067	-0.2858	0.775028	NA
CG31176	3.739119	-1.67783	3.960931	-0.4236	0.671861	NA
CG15498	0	NA	NA	NA	NA	NA
slou	0.784445	-1.74882	3.934036	-0.44454	0.656656	NA
CG5849	0.357625	-1.47707	4.044794	-0.36518	0.714979	NA
CG31343	30.49306	3.974879	3.878962	1.024728	0.305492	NA
CG31233	0.264155	-1.54099	4.048976	-0.38059	0.703509	NA
CG31198	8.581471	-4.22675	3.922584	-1.07754	0.281239	NA
Burs	0	NA	NA	NA	NA	NA
CG6800	58.06591	1.537193	3.951528	0.389012	0.697267	NA
CG31431	17.77187	-0.90243	3.952083	-0.22834	0.81938	NA
CG31178	0.528311	-1.72839	4.011871	-0.43082	0.6666	NA
P5CDh2	4.711497	1.713057	3.961514	0.432425	0.665433	NA
CG31465	0	NA	NA	NA	NA	NA
CG6678	23.78052	-4.37543	3.898063	-1.12246	0.261665	NA
Qsox3	0	NA	NA	NA	NA	NA

Qsox2	0.1047	0.613261	4.062799	0.150945	0.880019	NA
CG17819	0	NA	NA	NA	NA	NA
fit	0	NA	NA	NA	NA	NA
CG31174	4.843574	0.678216	3.932375	0.17247	0.863068	NA
CG6569	0	NA	NA	NA	NA	NA
Gr93d	0	NA	NA	NA	NA	NA
Gr93c	0	NA	NA	NA	NA	NA
Gr93b	0	NA	NA	NA	NA	NA
Theg	0.53416	-1.53481	4.012395	-0.38252	0.702078	NA
BomBc3	0.132078	-1.13211	4.069918	-0.27817	0.780885	NA
CG13407	0	NA	NA	NA	NA	NA
BomT3	14.78675	0.385959	3.840239	0.100504	0.919944	NA
CG6015	0	NA	NA	NA	NA	NA
Ir94a	0	NA	NA	NA	NA	NA
CG7080	0	NA	NA	NA	NA	NA
CG13862	0	NA	NA	NA	NA	NA
rdhB	40.48828	0.319379	3.950713	0.080841	0.935569	NA
CG7071	0	NA	NA	NA	NA	NA
CG7069	13.49694	1.705698	3.833984	0.444889	0.6564	NA
tRNA:Ser-(	0	NA	NA	NA	NA	NA
tRNA:Ser-(	0	NA	NA	NA	NA	NA
CG13856	1.989299	1.663439	3.974713	0.418505	0.675578	NA
CG13855	0.1047	0.613261	4.062799	0.150945	0.880019	NA
tRNA:Ser-(	0.95296	-0.11447	3.972323	-0.02882	0.977011	NA
tRNA:Ser-(	0	NA	NA	NA	NA	NA
CG13857	0.357625	-1.47707	4.044794	-0.36518	0.714979	NA
CG5386	0	NA	NA	NA	NA	NA
CG5388	0	NA	NA	NA	NA	NA
CG5391	0.178053	-1.16467	4.075067	-0.2858	0.775028	NA
Ir94c	0.712213	-1.57119	3.998613	-0.39293	0.694369	NA
Ir94b	0	NA	NA	NA	NA	NA
Pebp1	14.34389	1.740403	3.954495	0.440108	0.659859	NA
tHMG1	0	NA	NA	NA	NA	NA
tHMG2	0	NA	NA	NA	NA	NA
CG33110	9.028017	-3.12802	3.9239	-0.79717	0.425352	NA
CG31161	56.82071	1.715879	3.951797	0.434202	0.664142	NA
btn	0	NA	NA	NA	NA	NA
sit	0	NA	NA	NA	NA	NA
CG5326	0.936057	1.478224	3.957115	0.373561	0.708731	NA
CG33099	0	NA	NA	NA	NA	NA
CG5376	0	NA	NA	NA	NA	NA
CG13847	0.178053	-1.16467	4.075067	-0.2858	0.775028	NA
pinta	23.76536	-1.92185	3.833443	-0.50134	0.616134	NA
CG17625	4.491708	-2.28312	3.879964	-0.58844	0.556238	NA
Nup133	33.71021	-4.52804	3.780305	-1.1978	0.230996	NA
CG6972	0.178053	-1.16467	4.075067	-0.2858	0.775028	NA
CG6985	0.132078	-1.13211	4.069918	-0.27817	0.780885	NA
CG7031	0.1047	0.613261	4.062799	0.150945	0.880019	NA
CG4704	0.388135	0.584845	4.044798	0.144592	0.885033	NA
Nepl15	6.020741	-0.43663	3.950216	-0.11053	0.911986	NA

Nepl14	0	NA	NA	NA	NA	NA
Takl2	0	NA	NA	NA	NA	NA
CG4907	0	NA	NA	NA	NA	NA
CG13842	0	NA	NA	NA	NA	NA
CCAP	0	NA	NA	NA	NA	NA
Tpl94D	4.17304	-2.3219	3.804616	-0.61028	0.541674	NA
Gbp3	0.63886	-0.79045	3.986802	-0.19827	0.842837	NA
Or94a	0	NA	NA	NA	NA	NA
Or94b	0	NA	NA	NA	NA	NA
CG6688	0	NA	NA	NA	NA	NA
CG13838	0	NA	NA	NA	NA	NA
CG13837	0	NA	NA	NA	NA	NA
Gr94a	0	NA	NA	NA	NA	NA
CG6726	4.170776	-1.48628	3.917111	-0.37943	0.704366	NA
CG17110	0	NA	NA	NA	NA	NA
CG17109	0.357625	-1.47707	4.044794	-0.36518	0.714979	NA
CG6733	3.929571	1.013179	3.94962	0.256526	0.797545	NA
CG6738	0	NA	NA	NA	NA	NA
CG17119	0	NA	NA	NA	NA	NA
CG17121	26.25092	-7.35505	3.909707	-1.88123	0.059941	NA
lmd	0.566189	-0.21299	4.010979	-0.0531	0.957651	NA
CG13827	49.12118	8.211406	3.847206	2.134382	0.032812	NA
bb8	0	NA	NA	NA	NA	NA
wam	0.178053	-1.16467	4.075067	-0.2858	0.775028	NA
fzo	46.18135	8.178615	3.876846	2.109605	0.034892	NA
VhaAC39-2	0.283512	-0.2331	4.065094	-0.05734	0.954273	NA
Ir94f	0	NA	NA	NA	NA	NA
Ir94g	0.1047	0.613261	4.062799	0.150945	0.880019	NA
Ir94h	0.337716	1.464149	4.04412	0.362044	0.717319	NA
CG10164	0.356107	-1.47099	4.037764	-0.36431	0.715628	NA
Ugt303B2	0	NA	NA	NA	NA	NA
Ugt303B1	0	NA	NA	NA	NA	NA
Ugt303B3	0.314023	1.768229	4.015745	0.440324	0.659702	NA
CG4374	1.090133	-1.54143	3.911316	-0.3941	0.69351	NA
CG17380	0	NA	NA	NA	NA	NA
CG31515	0	NA	NA	NA	NA	NA
CG4408	18.78199	0.815491	3.826623	0.21311	0.831241	NA
CG16710	0	NA	NA	NA	NA	NA
CG18754	1.71701	-1.85109	3.972145	-0.46602	0.641203	NA
SPE	34.34826	0.322011	3.844929	0.08375	0.933256	NA
Gba1a	0.356866	-1.92326	4.037889	-0.4763	0.633858	NA
Qsox4	0	NA	NA	NA	NA	NA
Gba1b	30.29817	-0.55558	3.887344	-0.14292	0.886354	NA
CG10301	0	NA	NA	NA	NA	NA
CG10300	0	NA	NA	NA	NA	NA
CG10232	0	NA	NA	NA	NA	NA
nau	0	NA	NA	NA	NA	NA
CG31468	0.178053	-1.16467	4.075067	-0.2858	0.775028	NA
prt	0.310131	-1.94616	4.045058	-0.48112	0.630431	NA
CG10252	0	NA	NA	NA	NA	NA

CG10184	0	NA	NA	NA	NA	NA
tRNA:Leu-(	0	NA	NA	NA	NA	NA
CG10183	0	NA	NA	NA	NA	NA
CG16723	0.104623	0.639075	4.067674	0.157111	0.875158	NA
CG33337	0	NA	NA	NA	NA	NA
CG10182	0	NA	NA	NA	NA	NA
snRNA:U1:	0.1047	0.613261	4.062799	0.150945	0.880019	NA
snRNA:U1:	0	NA	NA	NA	NA	NA
CG31141	0.356107	-1.47099	4.037764	-0.36431	0.715628	NA
CG13599	0.356107	-1.47099	4.037764	-0.36431	0.715628	NA
CG12268	0	NA	NA	NA	NA	NA
RpS19b	0.178053	-1.16467	4.075067	-0.2858	0.775028	NA
tbrd-1	0	NA	NA	NA	NA	NA
snRNA:U1:	0.712213	-1.57119	3.998613	-0.39293	0.694369	NA
CG6000	35.60687	0.38676	3.731527	0.103646	0.91745	NA
Root	2.120372	1.292103	3.861043	0.334651	0.737888	NA
Npc2f	0	NA	NA	NA	NA	NA
Kal1	13.20616	1.757063	3.858309	0.455397	0.648823	NA
CG13606	0	NA	NA	NA	NA	NA
Acp95EF	0	NA	NA	NA	NA	NA
CG13607	0	NA	NA	NA	NA	NA
tRNA:Leu-7	17.06212	0.611372	3.92928	0.155594	0.876353	NA
CG6432	0	NA	NA	NA	NA	NA
Ms	6.394311	-1.60476	3.781118	-0.42441	0.671264	NA
CG33339	0	NA	NA	NA	NA	NA
CG33340	0.178053	-1.16467	4.075067	-0.2858	0.775028	NA
CG13613	0	NA	NA	NA	NA	NA
CG33341	27.51592	1.534667	3.952215	0.388305	0.69779	NA
CG13615	0	NA	NA	NA	NA	NA
CG13616	0.236701	-0.24276	4.020028	-0.06039	0.951846	NA
CG5768	30.572	4.601396	3.882717	1.185097	0.235979	NA
CG17780	0	NA	NA	NA	NA	NA
CG17781	0	NA	NA	NA	NA	NA
CG17782	0.283512	-0.2331	4.065094	-0.05734	0.954273	NA
CG17784	0	NA	NA	NA	NA	NA
CG5762	0	NA	NA	NA	NA	NA
CG5728	34.04257	-0.35453	3.86776	-0.09166	0.926966	NA
CG5715	4.078721	-0.2095	3.773961	-0.05551	0.95573	NA
CG31128	0	NA	NA	NA	NA	NA
DZIP1	0	NA	NA	NA	NA	NA
CG13618	0.104623	0.639075	4.067674	0.157111	0.875158	NA
tRNA:Asp-(	0	NA	NA	NA	NA	NA
snRNA:U6:	1.691618	-4.37744	3.934873	-1.11247	0.265935	NA
snRNA:U6:	0.415513	-1.071	4.002654	-0.26757	0.789028	NA
snRNA:U6:	0	NA	NA	NA	NA	NA
CG13622	0	NA	NA	NA	NA	NA
CG13627	0	NA	NA	NA	NA	NA
tld	5.373551	-2.10067	3.859203	-0.54433	0.586216	NA
asp	0.178053	-1.16467	4.075067	-0.2858	0.775028	NA
Ets96B	0	NA	NA	NA	NA	NA

CG6980	0	NA	NA	NA	NA	NA
Esp	0.1047	0.613261	4.062799	0.150945	0.880019	NA
CG7016	0.104623	0.639075	4.067674	0.157111	0.875158	NA
CG31381	60.97474	-0.60687	3.732172	-0.1626	0.87083	NA
CG11069	0	NA	NA	NA	NA	NA
CG10845	0	NA	NA	NA	NA	NA
tRNA:Asp-(	0	NA	NA	NA	NA	NA
Mink	0.233093	0.692656	4.076246	0.169925	0.865069	NA
Trf4-2	0	NA	NA	NA	NA	NA
tRNA:Asp-(	0	NA	NA	NA	NA	NA
CG13643	0	NA	NA	NA	NA	NA
CG31121	0.53416	-1.53481	4.012395	-0.38252	0.702078	NA
CycB3	15.60029	1.74153	3.95421	0.440424	0.65963	NA
CG13640	0	NA	NA	NA	NA	NA
ppk22	0.104623	0.639075	4.067674	0.157111	0.875158	NA
CAH4	0	NA	NA	NA	NA	NA
niki	0	NA	NA	NA	NA	NA
CG5808	29.03263	0.186978	3.839175	0.048703	0.961156	NA
Ude	0.660388	-1.75977	4.001266	-0.4398	0.660079	NA
CG13634	0.282677	-0.09599	4.057934	-0.02366	0.981127	NA
AstA	1.503183	2.269606	3.970131	0.57167	0.567545	NA
CG31115	2.929451	1.496835	3.962734	0.377728	0.705633	NA
CG11786	2.564026	1.129155	3.968527	0.284527	0.776006	NA
fd96Ca	0	NA	NA	NA	NA	NA
fd96Cb	0	NA	NA	NA	NA	NA
Mocs2B	0	NA	NA	NA	NA	NA
CG13653	0	NA	NA	NA	NA	NA
CG31103	40.41417	1.749563	3.952192	0.442682	0.657996	NA
CG11852	0	NA	NA	NA	NA	NA
to	0	NA	NA	NA	NA	NA
CG11854	0	NA	NA	NA	NA	NA
CG13658	0	NA	NA	NA	NA	NA
CG13659	0.2094	1.005034	4.045341	0.248442	0.803792	NA
rha	0	NA	NA	NA	NA	NA
CG10669	22.46979	1.48289	3.906428	0.379603	0.704241	NA
CG10553	0	NA	NA	NA	NA	NA
CG10559	0	NA	NA	NA	NA	NA
CG31087	0.1047	0.613261	4.062799	0.150945	0.880019	NA
CG31099	0	NA	NA	NA	NA	NA
CG31288	0.233093	0.692656	4.076246	0.169925	0.865069	NA
CG31097	2.145747	-2.13625	3.989508	-0.53547	0.592328	NA
CG31102	1.072874	-2.0339	4.019685	-0.50598	0.612867	NA
bam	0	NA	NA	NA	NA	NA
Cad96Cb	0.104623	0.639075	4.067674	0.157111	0.875158	NA
CG5116	7.19094	-0.33858	3.795436	-0.08921	0.928917	NA
CG5112	0	NA	NA	NA	NA	NA
CG5107	51.77445	-1.92043	3.951661	-0.48598	0.626982	NA
CG5111	0.104623	0.639075	4.067674	0.157111	0.875158	NA
CG4582	0	NA	NA	NA	NA	NA
Lgr3	8.579107	1.524292	3.95506	0.385403	0.699939	NA

CG5024	0	NA	NA	NA	NA	NA
CG17770	0	NA	NA	NA	NA	NA
Acam	0	NA	NA	NA	NA	NA
CG14354	0.1047	0.613261	4.062799	0.150945	0.880019	NA
CG14545	0.178053	-1.16467	4.075067	-0.2858	0.775028	NA
Mst57Da	0	NA	NA	NA	NA	NA
Mst57Db	0	NA	NA	NA	NA	NA
Mst57Dc	0	NA	NA	NA	NA	NA
CG31093	0	NA	NA	NA	NA	NA
CG31380	0	NA	NA	NA	NA	NA
CG4960	0	NA	NA	NA	NA	NA
CG17196	0	NA	NA	NA	NA	NA
CG17197	0	NA	NA	NA	NA	NA
CG4730	0	NA	NA	NA	NA	NA
CG5948	51.16004	-3.52142	3.899401	-0.90307	0.36649	NA
Mco3	0.132078	-1.13211	4.069918	-0.27817	0.780885	NA
CG14540	0	NA	NA	NA	NA	NA
boss	3.769197	1.70343	3.964029	0.429722	0.667398	NA
E(spl)mdel	0	NA	NA	NA	NA	NA
E(spl)mgar	0	NA	NA	NA	NA	NA
E(spl)malp	0	NA	NA	NA	NA	NA
Kaz-m1	0	NA	NA	NA	NA	NA
E(spl)m7-H	0.356107	-1.47099	4.037764	-0.36431	0.715628	NA
E(spl)m8-H	0.104623	0.639075	4.067674	0.157111	0.875158	NA
CG14544	2.408098	1.677625	3.970879	0.422482	0.672673	NA
dysf	0	NA	NA	NA	NA	NA
CG14551	0	NA	NA	NA	NA	NA
PIG-P	0	NA	NA	NA	NA	NA
E(spl)m4-B	0.356107	-1.47099	4.037764	-0.36431	0.715628	NA
E(spl)m2-B	0	NA	NA	NA	NA	NA
CG14546	0.1047	0.613261	4.062799	0.150945	0.880019	NA
CG14556	0	NA	NA	NA	NA	NA
CG31086	0	NA	NA	NA	NA	NA
Ald2	0	NA	NA	NA	NA	NA
asRNA:CR3	0	NA	NA	NA	NA	NA
Hex-t1	0	NA	NA	NA	NA	NA
Hex-t2	0	NA	NA	NA	NA	NA
CG14237	0	NA	NA	NA	NA	NA
CG14238	0	NA	NA	NA	NA	NA
Pdf	0.178053	-1.16467	4.075067	-0.2858	0.775028	NA
ppk15	0	NA	NA	NA	NA	NA
CG6036	0	NA	NA	NA	NA	NA
TwdlM	0	NA	NA	NA	NA	NA
TwdlJ	0	NA	NA	NA	NA	NA
TwdlN	0.282753	-0.25398	4.020204	-0.06318	0.949626	NA
TwdlH	0	NA	NA	NA	NA	NA
TwdlR	0.466186	0.959471	4.033986	0.237847	0.812	NA
TwdlS	0	NA	NA	NA	NA	NA
TwdlD	0.1047	0.613261	4.062799	0.150945	0.880019	NA
CG14244	0	NA	NA	NA	NA	NA

CG6403	0	NA	NA	NA	NA	NA
TwdlQ	0	NA	NA	NA	NA	NA
TwdlK	0	NA	NA	NA	NA	NA
TwdlO	11.93297	-3.479	3.906416	-0.89059	0.373152	NA
TwdlL	51.08661	-1.89434	3.951656	-0.47938	0.63167	NA
TwdlB	0	NA	NA	NA	NA	NA
TwdlP	0.1047	0.613261	4.062799	0.150945	0.880019	NA
ms(3)K81	0	NA	NA	NA	NA	NA
Spag1	1.360102	1.453444	3.975267	0.365622	0.714647	NA
CG14253	5.547263	-1.89916	3.957803	-0.47985	0.631332	NA
TwdlC	0	NA	NA	NA	NA	NA
CG31077	0	NA	NA	NA	NA	NA
CG6295	0	NA	NA	NA	NA	NA
CG6296	0	NA	NA	NA	NA	NA
CG31076	0	NA	NA	NA	NA	NA
Gr97a	0	NA	NA	NA	NA	NA
tRNA:Gln-⁻	0	NA	NA	NA	NA	NA
tRNA:Gln-⁻	0	NA	NA	NA	NA	NA
ball	26.98997	-3.91961	3.807038	-1.02957	0.303212	NA
TwdlT	23.55419	6.962447	3.913302	1.779175	0.075211	NA
CG14258	22.36002	-0.85662	3.865561	-0.2216	0.824623	NA
CG17189	0.365171	-0.43584	4.056359	-0.10745	0.914434	NA
CG5882	0	NA	NA	NA	NA	NA
CG5909	1.788123	-2.11324	3.996051	-0.52883	0.596922	NA
ppk31	0	NA	NA	NA	NA	NA
spn-D	0.132078	-1.13211	4.069918	-0.27817	0.780885	NA
CG31068	20.13944	-0.66616	3.95203	-0.16856	0.866141	NA
PolA2	0	NA	NA	NA	NA	NA
Ser	51.15426	2.881849	3.865287	0.745572	0.455926	NA
CG6059	0	NA	NA	NA	NA	NA
Cpr97Eb	0.233093	0.692656	4.076246	0.169925	0.865069	NA
Cpr97Ea	0	NA	NA	NA	NA	NA
Nep5	0.356107	-1.47099	4.037764	-0.36431	0.715628	NA
CG6271	0	NA	NA	NA	NA	NA
CG6277	0	NA	NA	NA	NA	NA
CG6283	0	NA	NA	NA	NA	NA
CG17191	0.31387	1.261782	4.037145	0.312543	0.754628	NA
CG17192	0	NA	NA	NA	NA	NA
CG14260	0	NA	NA	NA	NA	NA
CG3330	0.2094	1.005034	4.045341	0.248442	0.803792	NA
CG3339	4.862478	-0.64598	3.931505	-0.16431	0.869488	NA
CG14262	0	NA	NA	NA	NA	NA
CG5984	1.151699	1.609751	3.989673	0.403479	0.686596	NA
CG13978	0	NA	NA	NA	NA	NA
Cyp6a18	0	NA	NA	NA	NA	NA
Gr98a	0	NA	NA	NA	NA	NA
CG12885	0.396233	-1.6814	4.028351	-0.41739	0.676392	NA
Gr98b	0.178053	-1.16467	4.075067	-0.2858	0.775028	NA
Gr98c	0.178053	-1.16467	4.075067	-0.2858	0.775028	NA
Gr98d	0	NA	NA	NA	NA	NA

CG5639	0.466186	0.959471	4.033986	0.237847	0.812	NA
CG13972	0	NA	NA	NA	NA	NA
CG5402	3.664497	1.702077	3.964384	0.429342	0.667674	NA
Ets98B	31.76204	-6.45629	3.870095	-1.66825	0.095266	NA
Or98P	1.570499	1.642769	3.980387	0.412716	0.679815	NA
Or98a	28.09611	-0.52971	3.87027	-0.13687	0.891137	NA
CG12426	0.488184	-2.32943	4.022674	-0.57908	0.562538	NA
Nep7	0	NA	NA	NA	NA	NA
CR31054	0	NA	NA	NA	NA	NA
CG4815	0.178053	-1.16467	4.075067	-0.2858	0.775028	NA
CG31131	0	NA	NA	NA	NA	NA
CG31253	0	NA	NA	NA	NA	NA
CG4884	0	NA	NA	NA	NA	NA
nenya	0	NA	NA	NA	NA	NA
Mst98Ca	0.178812	-1.08808	4.061735	-0.26789	0.788788	NA
Mst98Cb	0	NA	NA	NA	NA	NA
UQCR-14L	0	NA	NA	NA	NA	NA
CG12516	0	NA	NA	NA	NA	NA
CG1894	0	NA	NA	NA	NA	NA
Dhc98D	5.423543	-2.07187	3.783525	-0.5476	0.583965	NA
mIF2	7.174845	-1.8917	3.868038	-0.48906	0.624801	NA
Ppn	44.69223	-8.65874	3.908682	-2.21526	0.026742	NA
CG9988	0	NA	NA	NA	NA	NA
CG33346	0	NA	NA	NA	NA	NA
Sid	0	NA	NA	NA	NA	NA
intr	0	NA	NA	NA	NA	NA
aqrs	0	NA	NA	NA	NA	NA
CG9997	0.178053	-1.16467	4.075067	-0.2858	0.775028	NA
CG10000	0	NA	NA	NA	NA	NA
CG31050	0	NA	NA	NA	NA	NA
Nepl18	3.894907	2.998356	3.922914	0.764318	0.444677	NA
CG11828	0	NA	NA	NA	NA	NA
Ssl2	1.351073	-1.13435	3.970931	-0.28566	0.775136	NA
wat	0	NA	NA	NA	NA	NA
Nepl19	0.411147	-0.29058	4.046973	-0.0718	0.94276	NA
Cpsf100	34.97031	2.512594	3.749483	0.670118	0.502783	NA
CG11837	2.019106	1.540265	3.919652	0.39296	0.694349	NA
CG11841	59.53394	0.420113	3.844034	0.10929	0.912973	NA
CG11842	0.178053	-1.16467	4.075067	-0.2858	0.775028	NA
CG11843	1.452855	-1.83946	3.975716	-0.46267	0.643599	NA
Brd8	10.75107	-2.54421	3.802661	-0.66906	0.503458	NA
stg	37.0637	2.971108	3.919966	0.757942	0.448486	NA
CG31445	0.209247	1.042804	4.047837	0.25762	0.7967	NA
CR31427	0	NA	NA	NA	NA	NA
CG11951	15.58734	0.407201	3.844339	0.105922	0.915644	NA
CG14507	0	NA	NA	NA	NA	NA
Cyt-c1L	0.178812	-1.08808	4.061735	-0.26789	0.788788	NA
CG14511	0	NA	NA	NA	NA	NA
yem	28.00314	3.230829	3.850143	0.839145	0.401388	NA
beta4GalN	0	NA	NA	NA	NA	NA

Diedel	0	NA	NA	NA	NA	NA
CG2310	3.112374	-0.89023	3.914142	-0.22744	0.820082	NA
Spase12	0	NA	NA	NA	NA	NA
CG2321	5.758496	1.720238	3.959653	0.434441	0.663968	NA
CG14506	0	NA	NA	NA	NA	NA
ND-20L	0	NA	NA	NA	NA	NA
ppk30	6.616054	-2.22778	3.964757	-0.5619	0.574188	NA
Obp99a	0	NA	NA	NA	NA	NA
ppk19	0	NA	NA	NA	NA	NA
Cap-D2	0.178053	-1.16467	4.075067	-0.2858	0.775028	NA
Obp99d	33.99368	-1.81019	3.951967	-0.45805	0.646919	NA
CG15506	12.35451	2.945353	3.925793	0.750257	0.4531	NA
Obp99b	0	NA	NA	NA	NA	NA
CG7568	0	NA	NA	NA	NA	NA
CG31041	0	NA	NA	NA	NA	NA
CG11470	0.132078	-1.13211	4.069918	-0.27817	0.780885	NA
CG7567	0	NA	NA	NA	NA	NA
Prtl99C	0	NA	NA	NA	NA	NA
Cad99C	49.11413	1.723013	3.637413	0.473692	0.63572	NA
CG15515	0	NA	NA	NA	NA	NA
CG31036	8.162549	1.532669	3.903074	0.392682	0.694554	NA
Jon99Ciii	0.932373	1.053996	3.996193	0.26375	0.791972	NA
alpha-Man	0	NA	NA	NA	NA	NA
Jon99Ci	0	NA	NA	NA	NA	NA
Jon99Cii	0.1047	0.613261	4.062799	0.150945	0.880019	NA
Capa	0	NA	NA	NA	NA	NA
CG15517	0	NA	NA	NA	NA	NA
CG7829	0	NA	NA	NA	NA	NA
ncd	0.310131	-1.94616	4.045058	-0.48112	0.630431	NA
fig	4.083297	1.7071	3.963068	0.430752	0.666649	NA
CG7896	0.744924	-1.68269	3.955513	-0.4254	0.670542	NA
Sas-6	0	NA	NA	NA	NA	NA
Sry-beta	0	NA	NA	NA	NA	NA
Sry-alpha	0	NA	NA	NA	NA	NA
Rpp14b	0	NA	NA	NA	NA	NA
CG7950	0.2094	1.005034	4.045341	0.248442	0.803792	NA
CG15529	0.837599	1.568055	4.001801	0.391837	0.695178	NA
CG15530	0	NA	NA	NA	NA	NA
CG15531	0	NA	NA	NA	NA	NA
CG9747	0	NA	NA	NA	NA	NA
CG11498	0	NA	NA	NA	NA	NA
CG31029	47.89633	-1.70452	3.951708	-0.43134	0.666223	NA
Ppi1	0	NA	NA	NA	NA	NA
CG15528	0	NA	NA	NA	NA	NA
janB	0	NA	NA	NA	NA	NA
ocn	0	NA	NA	NA	NA	NA
RpS28a	0	NA	NA	NA	NA	NA
Cec-Psi1	0	NA	NA	NA	NA	NA
CecC	0	NA	NA	NA	NA	NA
CG9682	0.178053	-1.16467	4.075067	-0.2858	0.775028	NA

CG18404	0	NA	NA	NA	NA	NA
CG15533	0	NA	NA	NA	NA	NA
CG2217	0	NA	NA	NA	NA	NA
tRNA:Leu-'	0.1047	0.613261	4.062799	0.150945	0.880019	NA
tRNA:Leu-'	0	NA	NA	NA	NA	NA
CG9733	0	NA	NA	NA	NA	NA
CG9737	0	NA	NA	NA	NA	NA
CG15534	5.438891	2.034849	3.783406	0.537835	0.590691	NA
spdo	0.178053	-1.16467	4.075067	-0.2858	0.775028	NA
Jon99Fi	0	NA	NA	NA	NA	NA
PH4alphaS	0.178053	-1.16467	4.075067	-0.2858	0.775028	NA
CG15539	0	NA	NA	NA	NA	NA
CG9698	0	NA	NA	NA	NA	NA
CG9702	0	NA	NA	NA	NA	NA
CG31019	7.428251	1.522001	3.955692	0.384762	0.700414	NA
CG31021	0	NA	NA	NA	NA	NA
PH4alphaN	0	NA	NA	NA	NA	NA
PH4alphaF	0	NA	NA	NA	NA	NA
CG31013	0	NA	NA	NA	NA	NA
CG2267	0.104623	0.639075	4.067674	0.157111	0.875158	NA
CG9717	0.104623	0.639075	4.067674	0.157111	0.875158	NA
Rpt6R	0	NA	NA	NA	NA	NA
CG31524	0	NA	NA	NA	NA	NA
PH4alphaN	0.1047	0.613261	4.062799	0.150945	0.880019	NA
PH4alphaS	0	NA	NA	NA	NA	NA
CG31371	2.810487	-2.67968	3.85456	-0.6952	0.486932	NA
PH4alphaN	0	NA	NA	NA	NA	NA
PH4alphaN	0	NA	NA	NA	NA	NA
Jon99Fii	0	NA	NA	NA	NA	NA
Osi23	0	NA	NA	NA	NA	NA
Spn100A	0.1047	0.613261	4.062799	0.150945	0.880019	NA
eIF4H2	0.536437	-1.71175	4.038042	-0.42391	0.671635	NA
CG15543	0	NA	NA	NA	NA	NA
CG15544	50.30335	4.031695	3.882542	1.038416	0.299076	NA
Cpr100A	0	NA	NA	NA	NA	NA
Cyp4c3	0	NA	NA	NA	NA	NA
CG15546	0	NA	NA	NA	NA	NA
CG15545	0	NA	NA	NA	NA	NA
Npc2h	0	NA	NA	NA	NA	NA
Npc2g	0	NA	NA	NA	NA	NA
CG11313	0	NA	NA	NA	NA	NA
CG31010	0	NA	NA	NA	NA	NA
Pka-C2	0	NA	NA	NA	NA	NA
CG12069	0	NA	NA	NA	NA	NA
CG15548	0	NA	NA	NA	NA	NA
Sox100B	0	NA	NA	NA	NA	NA
CG15554	27.04578	-0.62809	3.927409	-0.15993	0.87294	NA
CG15556	1.328986	1.486893	3.928973	0.378443	0.705101	NA
CG11318	0	NA	NA	NA	NA	NA
CG31008	0	NA	NA	NA	NA	NA

Ctr1C	0	NA	NA	NA	NA	NA
CG15550	0	NA	NA	NA	NA	NA
CG15549	0	NA	NA	NA	NA	NA
bnk	13.76109	-3.67364	3.810035	-0.9642	0.334945	NA
CAH6	0.178053	-1.16467	4.075067	-0.2858	0.775028	NA
CAH16	0.132078	-1.13211	4.069918	-0.27817	0.780885	NA
CAH5	0	NA	NA	NA	NA	NA
ppk24	0.178812	-1.08808	4.061735	-0.26789	0.788788	NA
pHCl-2	0.178812	-1.08808	4.061735	-0.26789	0.788788	NA
CG15564	0	NA	NA	NA	NA	NA
CG15563	0	NA	NA	NA	NA	NA
Zwilch	13.80376	4.323275	3.857939	1.120618	0.262451	NA
gskt	0.132078	-1.13211	4.069918	-0.27817	0.780885	NA
CG11333	0.132078	-1.13211	4.069918	-0.27817	0.780885	NA
mey	0	NA	NA	NA	NA	NA
nyo	0.209247	1.042804	4.047837	0.25762	0.7967	NA
CG1635	34.16379	-3.54834	3.767886	-0.94173	0.34633	NA
CG1638	0	NA	NA	NA	NA	NA
PNPase	1.416981	1.957611	3.905528	0.501241	0.616201	NA
Ugt35D1	0.178053	-1.16467	4.075067	-0.2858	0.775028	NA
CG31204	0	NA	NA	NA	NA	NA
Ir100a	0.1047	0.613261	4.062799	0.150945	0.880019	NA
CG1971	0	NA	NA	NA	NA	NA
CG2150	0	NA	NA	NA	NA	NA
Smvt	0	NA	NA	NA	NA	NA
CG2187	0.209247	1.042804	4.047837	0.25762	0.7967	NA
salt	0.356866	-1.92326	4.037889	-0.4763	0.633858	NA
CG11550	0	NA	NA	NA	NA	NA
CG2003	4.582433	-1.5598	3.835416	-0.40668	0.68424	NA
CG2053	0.132078	-1.13211	4.069918	-0.27817	0.780885	NA
His-Psi:CR3	0	NA	NA	NA	NA	NA
His-Psi:CR3	0	NA	NA	NA	NA	NA
His-Psi:CR3	0	NA	NA	NA	NA	NA
CG11630	2.443261	-3.33948	3.872261	-0.86241	0.388461	NA
CG1421	0	NA	NA	NA	NA	NA
CG1428	0	NA	NA	NA	NA	NA
CG11634	0	NA	NA	NA	NA	NA
CR31700	0	NA	NA	NA	NA	NA
tplus3a	0	NA	NA	NA	NA	NA
ttm3	0	NA	NA	NA	NA	NA
CG10834	0	NA	NA	NA	NA	NA
CG15219	0.282753	-0.25398	4.020204	-0.06318	0.949626	NA
tplus3b	0	NA	NA	NA	NA	NA
CG31601	0.178053	-1.16467	4.075067	-0.2858	0.775028	NA
CG2528	1.437098	-2.04622	3.922998	-0.5216	0.601952	NA
CG11629	0	NA	NA	NA	NA	NA
CG3635	1.589402	-0.20894	3.970589	-0.05262	0.958034	NA
CR12628	12.77937	-0.77664	3.73913	-0.20771	0.835458	NA
CG7882	0	NA	NA	NA	NA	NA
tRNA:Arg-/	27.68305	0.038243	3.931617	0.009727	0.992239	NA

tRNA:Arg-/	0	NA	NA	NA	NA	NA
tRNA:Asn-(	0	NA	NA	NA	NA	NA
tRNA:Asn-(	0.396233	-1.6814	4.028351	-0.41739	0.676392	NA
tRNA:Lys-(	0	NA	NA	NA	NA	NA
tRNA:Ile-A	14.30498	-2.25603	3.957513	-0.57006	0.568635	NA
tRNA:Arg-/	4.083297	1.7071	3.963068	0.430752	0.666649	NA
tRNA:Arg-/	0.132078	-1.13211	4.069918	-0.27817	0.780885	NA
CG8343	0	NA	NA	NA	NA	NA
tRNA:Asn-(	0.178053	-1.16467	4.075067	-0.2858	0.775028	NA
eIF3f2	0	NA	NA	NA	NA	NA
CG17994	0.132078	-1.13211	4.069918	-0.27817	0.780885	NA
CG30432	0	NA	NA	NA	NA	NA
tRNA:Asn-(	0	NA	NA	NA	NA	NA
CG11211	43.13637	1.749881	3.952112	0.442771	0.657931	NA
tRNA:Arg-/	0.519531	-0.79833	3.992004	-0.19998	0.841494	NA
tRNA:Lys-(	0	NA	NA	NA	NA	NA
tRNA:Lys-(	0.178812	-1.08808	4.061735	-0.26789	0.788788	NA
tRNA:Lys-(	0	NA	NA	NA	NA	NA
tRNA:Asn-(	0	NA	NA	NA	NA	NA
tRNA:Asn-(	0.233093	0.692656	4.076246	0.169925	0.865069	NA
tRNA:Lys-(	32.29061	-2.20787	3.740898	-0.5902	0.555058	NA
CG7856	0	NA	NA	NA	NA	NA
Strica	0	NA	NA	NA	NA	NA
CG7881	0.69928	1.019866	4.009445	0.254366	0.799213	NA
Tsp42A	0.209247	1.042804	4.047837	0.25762	0.7967	NA
Or42b	0.282677	-0.09599	4.057934	-0.02366	0.981127	NA
Or42a	0.178812	-1.08808	4.061735	-0.26789	0.788788	NA
scaf	50.36067	1.75058	3.951939	0.442967	0.657789	NA
Ugt50B3	0	NA	NA	NA	NA	NA
TpnC4	0.132078	-1.13211	4.069918	-0.27817	0.780885	NA
CG30440	1.788123	-2.11324	3.996051	-0.52883	0.596922	NA
CG3270	0.807012	0.227825	3.984055	0.057184	0.954399	NA
Zip42C.2	0	NA	NA	NA	NA	NA
Zip42C.1	0	NA	NA	NA	NA	NA
chk	0	NA	NA	NA	NA	NA
Spn42Dc	1.762986	-3.23443	3.951497	-0.81853	0.413053	NA
Spn42De	0	NA	NA	NA	NA	NA
CheB42a	0.566265	-0.7478	3.984294	-0.18769	0.851121	NA
ppk25	33.27465	0.260813	3.950091	0.066027	0.947356	NA
Tsp42Ea	8.115026	-2.72109	3.809251	-0.71434	0.475018	NA
Tsp42Eb	0	NA	NA	NA	NA	NA
CG30160	0	NA	NA	NA	NA	NA
Tsp42Ec	0	NA	NA	NA	NA	NA
Cyp6u1	45.81478	7.075764	3.850556	1.837596	0.066122	NA
tRNA:Lys-(	6.460049	1.480691	3.957408	0.374157	0.708288	NA
tRNA:Lys-(	5.758496	1.720238	3.959653	0.434441	0.663968	NA
tRNA:Lys-(	3.140998	1.694072	3.966495	0.427096	0.66931	NA
tRNA:Lys-(	0	NA	NA	NA	NA	NA
CheB42c	24.18874	-3.28208	3.90749	-0.83994	0.400939	NA
CheB42b	11.51699	1.736994	3.955359	0.43915	0.660553	NA

Spn42Db	0.264155	-1.54099	4.048976	-0.38059	0.703509	NA
CG9447	0	NA	NA	NA	NA	NA
CG9445	0.178053	-1.16467	4.075067	-0.2858	0.775028	NA
GNBP-like3	0	NA	NA	NA	NA	NA
CG30154	0	NA	NA	NA	NA	NA
Bbd	41.81288	-0.306	3.838729	-0.07971	0.936464	NA
CG13423	0.178053	-1.16467	4.075067	-0.2858	0.775028	NA
lms	0	NA	NA	NA	NA	NA
tRNA:Trp-(	0	NA	NA	NA	NA	NA
CG13437	0	NA	NA	NA	NA	NA
CG13438	0	NA	NA	NA	NA	NA
Gr57a	0	NA	NA	NA	NA	NA
exu	38.26543	-7.13272	3.84555	-1.8548	0.063625	NA
tRNA:Trp-(	0	NA	NA	NA	NA	NA
Rcd6	5.547263	-1.89916	3.957803	-0.47985	0.631332	NA
Hil	5.893793	2.736316	3.846221	0.71143	0.476818	NA
Cpr57A	0	NA	NA	NA	NA	NA
Obp57d	0	NA	NA	NA	NA	NA
Obp57e	0	NA	NA	NA	NA	NA
CG13427	0	NA	NA	NA	NA	NA
CG30151	0	NA	NA	NA	NA	NA
CkIIbeta2	0	NA	NA	NA	NA	NA
CG13871	0	NA	NA	NA	NA	NA
CG13870	0	NA	NA	NA	NA	NA
CG16741	0	NA	NA	NA	NA	NA
Obp57a	35.05832	-1.90778	3.831405	-0.49793	0.618532	NA
CG13426	0	NA	NA	NA	NA	NA
CG11192	0	NA	NA	NA	NA	NA
Cpr56F	0	NA	NA	NA	NA	NA
Mpcp1	0	NA	NA	NA	NA	NA
plu	0	NA	NA	NA	NA	NA
tRNA:Gly-(	0	NA	NA	NA	NA	NA
CG11044	0	NA	NA	NA	NA	NA
tRNA:Gly-(	0	NA	NA	NA	NA	NA
Efhc1.2	1.712559	-0.44667	3.901753	-0.11448	0.908857	NA
CG9864	0.209247	1.042804	4.047837	0.25762	0.7967	NA
ppk6	0	NA	NA	NA	NA	NA
CG13869	0	NA	NA	NA	NA	NA
CG16894	0	NA	NA	NA	NA	NA
tRNA:Arg-(	0	NA	NA	NA	NA	NA
Obp56e	0	NA	NA	NA	NA	NA
Obp56f	0.1047	0.613261	4.062799	0.150945	0.880019	NA
CG8517	0	NA	NA	NA	NA	NA
tRNA:Ile-A	0	NA	NA	NA	NA	NA
tRNA:iMet	0	NA	NA	NA	NA	NA
tRNA:Lys-(	0	NA	NA	NA	NA	NA
Obp56h	0	NA	NA	NA	NA	NA
Obp56i	0	NA	NA	NA	NA	NA
CG30447	0	NA	NA	NA	NA	NA
CG13872	0.178812	-1.08808	4.061735	-0.26789	0.788788	NA

Obp56g	0	NA	NA	NA	NA	NA
Or56a	0	NA	NA	NA	NA	NA
tRNA:Glu-(	0	NA	NA	NA	NA	NA
tRNA:Glu-⁻	0	NA	NA	NA	NA	NA
tRNA:Glu-⁻	0	NA	NA	NA	NA	NA
tRNA:Glu-⁻	0	NA	NA	NA	NA	NA
tRNA:iMet	0.357625	-1.47707	4.044794	-0.36518	0.714979	NA
CG15120	0.1047	0.613261	4.062799	0.150945	0.880019	NA
Ir56b	0	NA	NA	NA	NA	NA
Ir56c	0	NA	NA	NA	NA	NA
CG15124	0	NA	NA	NA	NA	NA
CG15125	52.27531	-1.89497	3.95164	-0.47954	0.631554	NA
CG11018	24.69853	-1.91759	3.952484	-0.48516	0.627563	NA
isopeptida	7.616767	3.042683	3.856291	0.789018	0.430101	NA
CG15126	0	NA	NA	NA	NA	NA
Obp56a	4.273279	-1.68149	3.959707	-0.42465	0.671092	NA
Obp56d	6.075574	-1.90118	3.957216	-0.48043	0.630919	NA
Obp56c	0	NA	NA	NA	NA	NA
Obp56b	0	NA	NA	NA	NA	NA
CG15128	0	NA	NA	NA	NA	NA
CG15127	0	NA	NA	NA	NA	NA
CG15905	0.310131	-1.94616	4.045058	-0.48112	0.630431	NA
Ir56d	25.46162	-1.70363	3.952413	-0.43104	0.666442	NA
tRNA:His-(	0	NA	NA	NA	NA	NA
CG16926	0	NA	NA	NA	NA	NA
wbl	0	NA	NA	NA	NA	NA
tbrd-2	0	NA	NA	NA	NA	NA
rib	0.550877	0.424446	3.986474	0.106471	0.915208	NA
AANATL6	0	NA	NA	NA	NA	NA
AANATL4	0	NA	NA	NA	NA	NA
fest	1.098468	-0.50358	3.892767	-0.12936	0.89707	NA
CG10051	0.712213	-1.57119	3.998613	-0.39293	0.694369	NA
CG10062	0.357625	-1.47707	4.044794	-0.36518	0.714979	NA
CG10073	0	NA	NA	NA	NA	NA
tRNA:Ser-(	0.178812	-1.08808	4.061735	-0.26789	0.788788	NA
tRNA:Val-/	2.554805	0.923237	3.962137	0.233015	0.81575	NA
tRNA:Val-/	45.00211	-0.51174	3.846322	-0.13305	0.894156	NA
Arl6	0.132078	-1.13211	4.069918	-0.27817	0.780885	NA
CG9416	0.178812	-1.08808	4.061735	-0.26789	0.788788	NA
CG10474	0	NA	NA	NA	NA	NA
AANATL5	0.357625	-1.47707	4.044794	-0.36518	0.714979	NA
CG33453	0.178053	-1.16467	4.075067	-0.2858	0.775028	NA
CG33454	0.178053	-1.16467	4.075067	-0.2858	0.775028	NA
CG18605	0	NA	NA	NA	NA	NA
CG15115	0.178053	-1.16467	4.075067	-0.2858	0.775028	NA
Vha100-3	10.98544	1.52756	3.954161	0.386317	0.699262	NA
tRNA:Trp-(	0.4188	1.41068	4.034613	0.349644	0.726606	NA
CG15071	21.81159	-0.35488	3.840064	-0.09242	0.926368	NA
tRNA:Leu-/	31.23449	1.175693	3.952414	0.297462	0.766114	NA
CG15082	1.884599	1.659029	3.975915	0.41727	0.676481	NA

DptA	0	NA	NA	NA	NA	NA
DptB	0	NA	NA	NA	NA	NA
Jheh1	0	NA	NA	NA	NA	NA
CG18190	0	NA	NA	NA	NA	NA
CG15096	55.1811	-0.51522	3.948167	-0.1305	0.896174	NA
CG15093	33.5199	2.289047	3.780817	0.605437	0.544889	NA
CG17821	0	NA	NA	NA	NA	NA
CG18609	27.47424	-0.30682	3.951014	-0.07765	0.938103	NA
CG33136	0	NA	NA	NA	NA	NA
Gint3	0	NA	NA	NA	NA	NA
tRNA:Gly-(	0	NA	NA	NA	NA	NA
BomS1	0	NA	NA	NA	NA	NA
BomS4	0	NA	NA	NA	NA	NA
BomS2	0	NA	NA	NA	NA	NA
BomS3	0.132078	-1.13211	4.069918	-0.27817	0.780885	NA
BomT2	0	NA	NA	NA	NA	NA
BomS5	20.22782	0.357988	3.837242	0.093293	0.925671	NA
Idgf5	0	NA	NA	NA	NA	NA
GstE4	0	NA	NA	NA	NA	NA
GstE5	0	NA	NA	NA	NA	NA
GstE9	0	NA	NA	NA	NA	NA
CG5189	0	NA	NA	NA	NA	NA
CG30120	0	NA	NA	NA	NA	NA
Pepck2	0.837599	1.568055	4.001801	0.391837	0.695178	NA
Pepck1	0	NA	NA	NA	NA	NA
CG5327	0	NA	NA	NA	NA	NA
CG5493	0	NA	NA	NA	NA	NA
CG17669	0.178053	-1.16467	4.075067	-0.2858	0.775028	NA
GstE10	0.178053	-1.16467	4.075067	-0.2858	0.775028	NA
BomS6	0	NA	NA	NA	NA	NA
BomBc2	0	NA	NA	NA	NA	NA
BomBc1	0	NA	NA	NA	NA	NA
CG10914	11.51333	0.103462	3.87903	0.026672	0.978721	NA
CG14500	0	NA	NA	NA	NA	NA
CG30114	0	NA	NA	NA	NA	NA
fj	0	NA	NA	NA	NA	NA
CG18536	5.232674	2.459345	3.928366	0.626048	0.531284	NA
CG18537	25.46162	-1.70363	3.952413	-0.43104	0.666442	NA
Tango8	0	NA	NA	NA	NA	NA
CG5756	2.017234	-1.62286	3.916351	-0.41438	0.678596	NA
CG30323	0	NA	NA	NA	NA	NA
CG14490	0	NA	NA	NA	NA	NA
CG14491	0	NA	NA	NA	NA	NA
CG5084	0	NA	NA	NA	NA	NA
CG10912	0	NA	NA	NA	NA	NA
CG10911	0	NA	NA	NA	NA	NA
Muc55B	0	NA	NA	NA	NA	NA
CG14495	12.92548	3.94513	3.895408	1.012764	0.311173	NA
CG5767	45.58164	-1.70567	3.95175	-0.43162	0.666015	NA
CG5770	0	NA	NA	NA	NA	NA

CG5773	60.31402	-2.21535	3.952429	-0.5605	0.575136	NA
PpY-55A	0	NA	NA	NA	NA	NA
CG30110	18.12623	0.036085	3.932248	0.009177	0.992678	NA
CG14480	0	NA	NA	NA	NA	NA
POSH	19.00059	0.514523	3.799228	0.135428	0.892273	NA
ND-51L1	42.73355	-2.9315	3.918653	-0.74809	0.454407	NA
tRNA:Phe-	0	NA	NA	NA	NA	NA
snoRNA:M	0	NA	NA	NA	NA	NA
snoRNA:M	52.08906	0.959997	3.30004	0.290905	0.771124	NA
snoRNA:M	2.615581	1.492057	3.964088	0.376393	0.706624	NA
snoRNA:U:	1.163877	-0.74508	3.943157	-0.18896	0.850128	NA
snoRNA:U:	0.743406	0.099327	3.941247	0.025202	0.979894	NA
snoRNA:U	5.831972	2.276407	3.800113	0.599037	0.549148	NA
snoRNA:U:	17.74801	0.912382	3.752496	0.24314	0.807897	NA
snoRNA:U:	1.569349	1.463746	3.972245	0.368493	0.712505	NA
snoRNA:M	3.557267	2.696207	3.935151	0.68516	0.493243	NA
snoRNA:sn	0.732899	1.548046	4.007786	0.38626	0.699304	NA
snoRNA:U:	0	NA	NA	NA	NA	NA
snoRNA:U:	2.79447	1.814247	3.907967	0.464243	0.642473	NA
snoRNA:U:	0	NA	NA	NA	NA	NA
snoRNA:U:	1.583959	3.294289	3.954192	0.833113	0.404781	NA
snoRNA:sn	2.092465	1.481166	3.9672	0.373353	0.708886	NA
snoRNA:M	1.046233	1.431318	3.981858	0.35946	0.719251	NA
CG10934	0	NA	NA	NA	NA	NA
Dlish	0	NA	NA	NA	NA	NA
CG4975	12.77962	0.837322	3.952701	0.211835	0.832236	NA
CG4984	36.72276	1.535867	3.951888	0.388641	0.697541	NA
Ir54a	0	NA	NA	NA	NA	NA
sub	0	NA	NA	NA	NA	NA
Oxp	16.24556	-1.91474	3.953298	-0.48434	0.628144	NA
rhi	0	NA	NA	NA	NA	NA
tRNA:Phe-	0	NA	NA	NA	NA	NA
tRNA:Ala-T	0	NA	NA	NA	NA	NA
CG10939	0.460806	-0.60052	3.997252	-0.15023	0.880581	NA
CG10764	28.20428	1.175216	3.952575	0.297329	0.766215	NA
veil	0	NA	NA	NA	NA	NA
CG30103	0.356107	-1.47099	4.037764	-0.36431	0.715628	NA
CG4847	0	NA	NA	NA	NA	NA
insb	0	NA	NA	NA	NA	NA
Tes	23.35951	5.14908	3.837351	1.341832	0.17965	NA
mthl3	0	NA	NA	NA	NA	NA
mthl4	0	NA	NA	NA	NA	NA
Vajk4	0	NA	NA	NA	NA	NA
tRNA:Ala-T	0	NA	NA	NA	NA	NA
SmydA-6	0	NA	NA	NA	NA	NA
CG17717	0	NA	NA	NA	NA	NA
APC7	0	NA	NA	NA	NA	NA
CG14443	58.92661	6.528762	3.731039	1.749851	0.080144	NA
Mcm6	50.98199	-1.92041	3.951672	-0.48598	0.626985	NA
CG14439	33.71903	0.37291	3.845652	0.096969	0.922751	NA

Atx-1	0	NA	NA	NA	NA	NA
mAChR-C	0	NA	NA	NA	NA	NA
tRNA:Phe-	0	NA	NA	NA	NA	NA
CR4575	0.178053	-1.16467	4.075067	-0.2858	0.775028	NA
CR32733	0.574286	-2.5433	4.014753	-0.63349	0.526415	NA
CG4586	38.84344	4.015504	3.891507	1.031864	0.302136	NA
CG32736	0	NA	NA	NA	NA	NA
nullo	0.2094	1.005034	4.045341	0.248442	0.803792	NA
CG4607	3.384419	1.447151	3.896022	0.371443	0.710307	NA
lncRNA:CR	0.233093	0.692656	4.076246	0.169925	0.865069	NA
CG1958	57.55842	-1.8975	3.951574	-0.48019	0.631094	NA
CG18155	25.20102	0.25905	3.767351	0.068762	0.945179	NA
CG1999	0	NA	NA	NA	NA	NA
CG32726	0.104623	0.639075	4.067674	0.157111	0.875158	NA
CG15036	0	NA	NA	NA	NA	NA
CG15035	0.132078	-1.13211	4.069918	-0.27817	0.780885	NA
CG15034	0.236701	-0.24276	4.020028	-0.06039	0.951846	NA
CG11369	0.178053	-1.16467	4.075067	-0.2858	0.775028	NA
Ir7a	0.357625	-1.47707	4.044794	-0.36518	0.714979	NA
CG15478	0	NA	NA	NA	NA	NA
CG12689	0	NA	NA	NA	NA	NA
CG32720	0	NA	NA	NA	NA	NA
CG32719	18.46342	-1.84699	3.95291	-0.46725	0.640322	NA
CG32718	0	NA	NA	NA	NA	NA
CG1409	0	NA	NA	NA	NA	NA
Ir7c	0	NA	NA	NA	NA	NA
Ir7g	0	NA	NA	NA	NA	NA
CG15330	0	NA	NA	NA	NA	NA
CG2256	24.63898	1.467003	3.845691	0.381467	0.702857	NA
CG2233	0	NA	NA	NA	NA	NA
CG15333	0.446101	0.990384	3.987592	0.248366	0.803851	NA
CG15332	0.104623	0.639075	4.067674	0.157111	0.875158	NA
hdm	44.09142	-2.00744	3.815864	-0.52608	0.598834	NA
CR33222	0	NA	NA	NA	NA	NA
CR11386	0	NA	NA	NA	NA	NA
ND-MNLL	0	NA	NA	NA	NA	NA
Nek2	0.53416	-1.53481	4.012395	-0.38252	0.702078	NA
Ir7b	0.1047	0.613261	4.062799	0.150945	0.880019	NA
CG10920	0	NA	NA	NA	NA	NA
CG2120	0	NA	NA	NA	NA	NA
CG15336	0	NA	NA	NA	NA	NA
CG10959	0.492912	0.154683	4.013127	0.038544	0.969254	NA
CG15337	0	NA	NA	NA	NA	NA
CG1632	6.812187	0.068221	3.942824	0.017303	0.986195	NA
CG1387	0.178053	-1.16467	4.075067	-0.2858	0.775028	NA
Or7a	0	NA	NA	NA	NA	NA
CG10761	0.356107	-1.47099	4.037764	-0.36431	0.715628	NA
cyr	0.178053	-1.16467	4.075067	-0.2858	0.775028	NA
CG2260	0.466186	0.959471	4.033986	0.237847	0.812	NA
Cp7Fb	5.448478	-1.34948	3.883391	-0.3475	0.728215	NA

Cp7Fc	0.104623	0.639075	4.067674	0.157111	0.875158	NA
Cp36	0	NA	NA	NA	NA	NA
Cp38	2.913353	-2.39772	3.842118	-0.62406	0.532587	NA
CG12111	0	NA	NA	NA	NA	NA
CG33223	0.1047	0.613261	4.062799	0.150945	0.880019	NA
CG12116	33.53742	-5.48574	3.818001	-1.43681	0.150772	NA
CG15347	0	NA	NA	NA	NA	NA
CG32713	0	NA	NA	NA	NA	NA
org-1	0.233093	0.692656	4.076246	0.169925	0.865069	NA
CG32712	0.178053	-1.16467	4.075067	-0.2858	0.775028	NA
CG10962	55.62189	-7.1791	3.869574	-1.85527	0.063558	NA
CG12661	53.46411	-3.63361	3.913525	-0.92847	0.353162	NA
l(1)G0020	39.71398	-0.44893	3.854953	-0.11645	0.907292	NA
CG12057	35.00059	-1.9192	3.952021	-0.48563	0.627232	NA
CG12106	43.71874	-3.29433	3.773773	-0.87295	0.382688	NA
Obp8a	2.936409	0.416631	3.869041	0.107683	0.914247	NA
CG15368	21.97163	-3.14951	3.91884	-0.80369	0.421579	NA
CG15369	1.325477	0.764512	3.893052	0.196379	0.844314	NA
CG15370	0	NA	NA	NA	NA	NA
Gr8a	4.094408	-1.89106	3.960175	-0.47752	0.632993	NA
lz	0.178053	-1.16467	4.075067	-0.2858	0.775028	NA
CG17440	21.69568	-1.35849	3.783387	-0.35907	0.719545	NA
Erk7	0.178053	-1.16467	4.075067	-0.2858	0.775028	NA
CR17438	0	NA	NA	NA	NA	NA
Ir8a	30.63042	-3.25914	3.928093	-0.8297	0.406708	NA
SmydA-9	0.178053	-1.16467	4.075067	-0.2858	0.775028	NA
CG12115	0	NA	NA	NA	NA	NA
CG10970	0.396233	-1.6814	4.028351	-0.41739	0.676392	NA
Cubn	39.5728	-0.52138	3.772338	-0.13821	0.890074	NA
btd	0.387453	0.098632	4.002797	0.024641	0.980342	NA
Sp1	1.886966	-2.1758	3.893319	-0.55886	0.576261	NA
CG3106	0	NA	NA	NA	NA	NA
CG1889	7.217562	-4.80075	3.84009	-1.25017	0.211239	NA
CG32694	34.84252	0.118846	3.951416	0.030077	0.976006	NA
CG2990	3.455098	1.699144	3.965156	0.428519	0.668273	NA
Yp1	0	NA	NA	NA	NA	NA
Gr9a	0	NA	NA	NA	NA	NA
Yp2	0.209323	1.414133	4.032409	0.350692	0.725819	NA
CG15252	0	NA	NA	NA	NA	NA
CG1791	0	NA	NA	NA	NA	NA
CG12645	0	NA	NA	NA	NA	NA
lncRNA:CR	1.584932	-1.84571	3.973792	-0.46447	0.642311	NA
CG2909	52.7636	4.200996	3.814257	1.101393	0.270726	NA
CG2898	1.06832	-1.61145	3.983918	-0.40449	0.685853	NA
RabX2	0	NA	NA	NA	NA	NA
CG2887	0.396233	-1.6814	4.028351	-0.41739	0.676392	NA
Or9a	0	NA	NA	NA	NA	NA
CG12640	0	NA	NA	NA	NA	NA
Psf3	17.23342	-0.5553	3.750467	-0.14806	0.882294	NA
CG32681	27.46298	-0.87275	3.949352	-0.22098	0.825104	NA

CG32679	0.178812	-1.08808	4.061735	-0.26789	0.788788	NA
CG15306	0.233093	0.692656	4.076246	0.169925	0.865069	NA
Ipod	0	NA	NA	NA	NA	NA
CG15308	0	NA	NA	NA	NA	NA
CG2962	0	NA	NA	NA	NA	NA
Rab9D	0	NA	NA	NA	NA	NA
CG2111	0.283436	-0.20033	4.070792	-0.04921	0.960751	NA
CG12637	0	NA	NA	NA	NA	NA
Rab9E	36.82873	1.17637	3.952186	0.297651	0.76597	NA
CG9806	0	NA	NA	NA	NA	NA
Rab9Db	0	NA	NA	NA	NA	NA
CG1628	8.07681	-5.36937	3.853869	-1.39324	0.163546	NA
CG15225	0	NA	NA	NA	NA	NA
CAH14	0	NA	NA	NA	NA	NA
Ipk1	31.1851	0.646294	3.735272	0.173025	0.862632	NA
Cib2	61.87766	1.751356	3.951748	0.443185	0.657632	NA
CAH15	0	NA	NA	NA	NA	NA
Cht8	0.233093	0.692656	4.076246	0.169925	0.865069	NA
Cht9	0	NA	NA	NA	NA	NA
Cht4	0	NA	NA	NA	NA	NA
Cht12	0	NA	NA	NA	NA	NA
dgt3	0.104623	0.639075	4.067674	0.157111	0.875158	NA
CG3216	58.08918	-5.98332	3.575188	-1.67357	0.094216	NA
CG15650	7.857823	2.71295	3.907078	0.694368	0.487451	NA
Dso1	34.04859	-1.8805	3.95202	-0.47583	0.634194	NA
CG9993	0	NA	NA	NA	NA	NA
CG17999	0.178053	-1.16467	4.075067	-0.2858	0.775028	NA
CG9406	0	NA	NA	NA	NA	NA
tRNA:Trp-(	0	NA	NA	NA	NA	NA
tRNA:Gly-(	0.565353	0.142845	3.959283	0.036079	0.97122	NA
BBS9	0.52327	2.335758	3.994212	0.584786	0.558692	NA
CG17974	0	NA	NA	NA	NA	NA
CG9822	0	NA	NA	NA	NA	NA
CG9752	9.172321	3.702066	3.8959	0.950247	0.341987	NA
CG10505	4.881497	1.238411	3.839789	0.32252	0.747058	NA
CG30393	12.28568	-1.69873	3.954016	-0.42962	0.667472	NA
CG30391	0.712213	-1.57119	3.998613	-0.39293	0.694369	NA
tRNA:Trp-(	0	NA	NA	NA	NA	NA
CG4286	0	NA	NA	NA	NA	NA
tRNA:Gly-(	0.337793	1.451235	4.039844	0.35923	0.719423	NA
tRNA:Trp-(	0	NA	NA	NA	NA	NA
Egfr	0.283512	-0.2331	4.065094	-0.05734	0.954273	NA
CG30222	0.282753	-0.25398	4.020204	-0.06318	0.949626	NA
CG33225	0	NA	NA	NA	NA	NA
CG15674	19.89299	1.744326	3.953505	0.44121	0.659061	NA
CG15676	0	NA	NA	NA	NA	NA
Ppcdc	0	NA	NA	NA	NA	NA
Tim10	0.356107	-1.47099	4.037764	-0.36431	0.715628	NA
PIG-M	0	NA	NA	NA	NA	NA
CG30284	10.77345	-5.98626	3.923127	-1.52589	0.127037	NA

CG30283	0	NA	NA	NA	NA	NA
CG33226	0	NA	NA	NA	NA	NA
CG30287	0.178053	-1.16467	4.075067	-0.2858	0.775028	NA
CG30286	0	NA	NA	NA	NA	NA
CG30289	0	NA	NA	NA	NA	NA
CG30288	0	NA	NA	NA	NA	NA
CG10494	0.411906	-0.22397	4.020275	-0.05571	0.955572	NA
CG30398	0	NA	NA	NA	NA	NA
Grx1t	0	NA	NA	NA	NA	NA
CG30395	0	NA	NA	NA	NA	NA
CG4021	0.62774	1.374224	3.999506	0.343599	0.731148	NA
lncRNA:CR	0	NA	NA	NA	NA	NA
CG13488	0	NA	NA	NA	NA	NA
Gr58c	0	NA	NA	NA	NA	NA
Gr58a	0	NA	NA	NA	NA	NA
dany	0	NA	NA	NA	NA	NA
Gr58b	0	NA	NA	NA	NA	NA
CG13494	0.356866	-1.92326	4.037889	-0.4763	0.633858	NA
PpN58A	28.70198	-2.02706	3.814139	-0.53146	0.595101	NA
comr	0.178053	-1.16467	4.075067	-0.2858	0.775028	NA
tRNA:Gly-T	0	NA	NA	NA	NA	NA
tRNA:Gly-T	0.233093	0.692656	4.076246	0.169925	0.865069	NA
CG4372	0	NA	NA	NA	NA	NA
CG4377	0	NA	NA	NA	NA	NA
CG4363	0	NA	NA	NA	NA	NA
CG13492	0.396233	-1.6814	4.028351	-0.41739	0.676392	NA
tpr	0	NA	NA	NA	NA	NA
CG18735	0	NA	NA	NA	NA	NA
Loxl2	0.233093	0.692656	4.076246	0.169925	0.865069	NA
CG9308	7.478238	-1.69271	3.955987	-0.42789	0.668735	NA
CG30281	0	NA	NA	NA	NA	NA
mei-S332	0	NA	NA	NA	NA	NA
NtR	0	NA	NA	NA	NA	NA
CG11269	0.932373	1.053996	3.996193	0.26375	0.791972	NA
gom	34.4264	0.645301	3.731369	0.17294	0.862699	NA
CG11291	0	NA	NA	NA	NA	NA
Oatp58Db	0	NA	NA	NA	NA	NA
Oatp58Da	0	NA	NA	NA	NA	NA
CG30278	0	NA	NA	NA	NA	NA
Alp8	0.209247	1.042804	4.047837	0.25762	0.7967	NA
Alp7	0	NA	NA	NA	NA	NA
Swim	0.948342	-0.9323	3.989063	-0.23371	0.815207	NA
CG13502	0.132078	-1.13211	4.069918	-0.27817	0.780885	NA
CG11475	0	NA	NA	NA	NA	NA
CG13501	3.769197	1.70343	3.964029	0.429722	0.667398	NA
PpD5	0	NA	NA	NA	NA	NA
CG6967	5.431375	0.663113	3.848438	0.172307	0.863196	NA
Amy-d	0	NA	NA	NA	NA	NA
Cda9	20.20709	1.744484	3.953465	0.441255	0.659029	NA
CG11400	0.894061	-2.00006	4.030294	-0.49626	0.619714	NA

Gbp2	21.07336	0.92445	3.737275	0.247359	0.80463	NA
tRNA:Leu-/	0	NA	NA	NA	NA	NA
CG17290	0	NA	NA	NA	NA	NA
CG10950	0.890266	-1.59482	3.989917	-0.39971	0.689368	NA
CG12699	37.23569	1.245588	3.873662	0.321553	0.747791	NA
CG18469	0	NA	NA	NA	NA	NA
CG10953	0	NA	NA	NA	NA	NA
CG30457	0	NA	NA	NA	NA	NA
CG30458	0	NA	NA	NA	NA	NA
CG17287	0	NA	NA	NA	NA	NA
tRNA:Leu-/	0	NA	NA	NA	NA	NA
CG15605	0	NA	NA	NA	NA	NA
Spn53F	0	NA	NA	NA	NA	NA
Amy-p	0	NA	NA	NA	NA	NA
CG8950	9.343238	-5.35141	3.891488	-1.37516	0.169082	NA
Arp53D	0	NA	NA	NA	NA	NA
resilin	0.178053	-1.16467	4.075067	-0.2858	0.775028	NA
CG15919	0	NA	NA	NA	NA	NA
CG5550	0	NA	NA	NA	NA	NA
CG6426	47.60704	2.878717	3.919865	0.734392	0.46271	NA
CG6421	0.132078	-1.13211	4.069918	-0.27817	0.780885	NA
CG6429	0	NA	NA	NA	NA	NA
inaC	0.765088	-0.45272	3.976873	-0.11384	0.909366	NA
CG15614	1.193706	0.462543	3.970016	0.116509	0.907249	NA
tRNA:Gly-(	0.178053	-1.16467	4.075067	-0.2858	0.775028	NA
CG9010	0.178053	-1.16467	4.075067	-0.2858	0.775028	NA
Fen1	15.76455	-2.21017	3.871568	-0.57087	0.568087	NA
Ugt37C1	0	NA	NA	NA	NA	NA
CG15615	0.178812	-1.08808	4.061735	-0.26789	0.788788	NA
Acp53Ea	0	NA	NA	NA	NA	NA
Acp53C14l	0	NA	NA	NA	NA	NA
Acp53C14a	0	NA	NA	NA	NA	NA
CG15617	0	NA	NA	NA	NA	NA
CG33458	0	NA	NA	NA	NA	NA
S-Lap8	0.178053	-1.16467	4.075067	-0.2858	0.775028	NA
CG4945	0.71449	-2.47883	4.014121	-0.61753	0.536887	NA
CG5089	0.178053	-1.16467	4.075067	-0.2858	0.775028	NA
Amyrel	0	NA	NA	NA	NA	NA
CG8303	0.53416	-1.53481	4.012395	-0.38252	0.702078	NA
Lst	9.556822	1.165775	3.955791	0.294701	0.768222	NA
CG15712	0	NA	NA	NA	NA	NA
tRNA:Glu-(	0.715249	-1.88064	4.034336	-0.46616	0.641102	NA
tRNA:Glu-T	30.84723	-3.37581	3.869757	-0.87236	0.383013	NA
Tsf3	5.749738	-0.81538	3.955041	-0.20616	0.836664	NA
CG15701	5.758496	1.720238	3.959653	0.434441	0.663968	NA
CG3687	0	NA	NA	NA	NA	NA
CG15705	0	NA	NA	NA	NA	NA
CG9068	0.209247	1.042804	4.047837	0.25762	0.7967	NA
CG30100	0	NA	NA	NA	NA	NA
Parp16	6.86804	-1.90364	3.956501	-0.48114	0.630416	NA

CG7848	0.104623	0.639075	4.067674	0.157111	0.875158	NA
CG15711	0	NA	NA	NA	NA	NA
CG15710	0	NA	NA	NA	NA	NA
CG30324	0.178053	-1.16467	4.075067	-0.2858	0.775028	NA
CG7755	0.178053	-1.16467	4.075067	-0.2858	0.775028	NA
cato	0	NA	NA	NA	NA	NA
CG15708	0.1047	0.613261	4.062799	0.150945	0.880019	NA
krimp	0	NA	NA	NA	NA	NA
CG7786	0	NA	NA	NA	NA	NA
CG7798	0	NA	NA	NA	NA	NA
tRNA:Glu-⁻	0	NA	NA	NA	NA	NA
tRNA:Gln-(	20.03851	0.257894	3.836584	0.06722	0.946407	NA
COX6AL	0	NA	NA	NA	NA	NA
CG8399	2.973451	2.154611	3.93095	0.548115	0.583613	NA
CG8401	2.272052	1.167201	3.965566	0.294334	0.768503	NA
Jhedup	0	NA	NA	NA	NA	NA
Jhe	22.74358	3.185144	3.910177	0.814578	0.415314	NA
CG30095	0.132078	-1.13211	4.069918	-0.27817	0.780885	NA
CysRS	46.74145	7.833318	3.907689	2.004591	0.045007	NA
Ptp52F	8.188817	-1.9067	3.955613	-0.48202	0.629788	NA
CG10734	0.596853	0.454699	3.988272	0.114009	0.909231	NA
CG30091	0	NA	NA	NA	NA	NA
CG30090	0	NA	NA	NA	NA	NA
CG30088	0	NA	NA	NA	NA	NA
CG12970	0	NA	NA	NA	NA	NA
CG8299	0	NA	NA	NA	NA	NA
CG8249	0.178053	-1.16467	4.075067	-0.2858	0.775028	NA
Poxn	0	NA	NA	NA	NA	NA
CG30083	0	NA	NA	NA	NA	NA
CG30080	0	NA	NA	NA	NA	NA
Ir52a	0	NA	NA	NA	NA	NA
Ir52b	0	NA	NA	NA	NA	NA
Ir52c	0.178053	-1.16467	4.075067	-0.2858	0.775028	NA
Ir52d	0.132078	-1.13211	4.069918	-0.27817	0.780885	NA
CG30471	0	NA	NA	NA	NA	NA
CG12963	0.178053	-1.16467	4.075067	-0.2858	0.775028	NA
fus	11.60363	-3.07829	3.821689	-0.80548	0.420543	NA
CG8192	0	NA	NA	NA	NA	NA
CG30472	0	NA	NA	NA	NA	NA
Ir51b	0	NA	NA	NA	NA	NA
ItgaPS4	0.356107	-1.47099	4.037764	-0.36431	0.715628	NA
Hr51	0	NA	NA	NA	NA	NA
Mtk	0	NA	NA	NA	NA	NA
CG8160	0.104623	0.639075	4.067674	0.157111	0.875158	NA
CG8157	41.57385	7.063577	3.888074	1.816729	0.069259	NA
ND-51L2	0	NA	NA	NA	NA	NA
CG8093	0	NA	NA	NA	NA	NA
Cyp317a1	0.233093	0.692656	4.076246	0.169925	0.865069	NA
CG12853	0	NA	NA	NA	NA	NA
CG7544	36.17436	-2.1749	3.953361	-0.55014	0.582223	NA

tRNA:Leu-/	43.91365	-0.28895	3.835786	-0.07533	0.939952	NA
CG12866	0	NA	NA	NA	NA	NA
Dro	0	NA	NA	NA	NA	NA
AttA	0	NA	NA	NA	NA	NA
AttB	0.732899	1.548046	4.007786	0.38626	0.699304	NA
CG10205	0	NA	NA	NA	NA	NA
Cyp6a19	0	NA	NA	NA	NA	NA
Cyp6a8	0	NA	NA	NA	NA	NA
Cyp6a21	57.01561	1.559025	3.918779	0.397835	0.690752	NA
Pgm2b	27.73631	-1.91819	3.952312	-0.48533	0.62744	NA
CG12857	60.43855	-2.27598	3.952504	-0.57583	0.564728	NA
CG30480	0.46073	-0.17853	4.00793	-0.04454	0.964471	NA
CG30479	0	NA	NA	NA	NA	NA
CG12860	0	NA	NA	NA	NA	NA
CG12861	0	NA	NA	NA	NA	NA
Adgf-E	0	NA	NA	NA	NA	NA
CG12862	0	NA	NA	NA	NA	NA
CG10104	0	NA	NA	NA	NA	NA
Achl	0.356866	-1.92326	4.037889	-0.4763	0.633858	NA
CG12869	0.965993	2.477533	4.003194	0.618889	0.53599	NA
Had2	0.178053	-1.16467	4.075067	-0.2858	0.775028	NA
CG33468	0	NA	NA	NA	NA	NA
CG30076	1.015798	0.083253	3.968257	0.02098	0.983262	NA
CG18324	0.732899	1.548046	4.007786	0.38626	0.699304	NA
CG8468	16.86233	3.834619	3.807442	1.007138	0.313868	NA
SmydA-1	0	NA	NA	NA	NA	NA
Arc2	4.988115	1.214777	3.880852	0.313018	0.754267	NA
Obp50b	0	NA	NA	NA	NA	NA
Obp50d	0	NA	NA	NA	NA	NA
CG30075	0	NA	NA	NA	NA	NA
Obp50e	0.104623	0.639075	4.067674	0.157111	0.875158	NA
Obp50a	0	NA	NA	NA	NA	NA
CR10102	0	NA	NA	NA	NA	NA
tej	0.1047	0.613261	4.062799	0.150945	0.880019	NA
Sox15	1.675199	1.648777	3.978727	0.414398	0.678583	NA
CG18568	0	NA	NA	NA	NA	NA
St4	0	NA	NA	NA	NA	NA
DJ-1alpha	26.53136	-2.42901	3.746103	-0.64841	0.51672	NA
S-Lap5	0	NA	NA	NA	NA	NA
CG6280	0.178053	-1.16467	4.075067	-0.2858	0.775028	NA
Cpr50Ca	0.178053	-1.16467	4.075067	-0.2858	0.775028	NA
Cpr50Cb	0.178053	-1.16467	4.075067	-0.2858	0.775028	NA
CG30484	0.356107	-1.47099	4.037764	-0.36431	0.715628	NA
CG30485	0.2094	1.005034	4.045341	0.248442	0.803792	NA
CG6553	0	NA	NA	NA	NA	NA
CG6337	20.7665	-0.80729	3.937839	-0.20501	0.837566	NA
S-Lap7	0	NA	NA	NA	NA	NA
CG6220	0.1047	0.613261	4.062799	0.150945	0.880019	NA
CG6209	0.132078	-1.13211	4.069918	-0.27817	0.780885	NA
CG12464	0	NA	NA	NA	NA	NA

AttC	33.50398	1.748507	3.952456	0.442385	0.658211	NA
CG33470	0	NA	NA	NA	NA	NA
tRNA:Ile-A	0	NA	NA	NA	NA	NA
tRNA:Ile-A	0	NA	NA	NA	NA	NA
tRNA:Ile-A	0	NA	NA	NA	NA	NA
tRNA:Ile-A	0	NA	NA	NA	NA	NA
CG30065	0.178053	-1.16467	4.075067	-0.2858	0.775028	NA
Ser8	0	NA	NA	NA	NA	NA
tRNA:Ile-A	0	NA	NA	NA	NA	NA
CG13330	0	NA	NA	NA	NA	NA
CG13331	33.89143	1.691092	3.95237	0.427868	0.668747	NA
CG13332	52.98589	-1.39981	3.951506	-0.35425	0.723153	NA
link	19.01268	6.056002	3.848483	1.573608	0.115578	NA
CG13334	0	NA	NA	NA	NA	NA
tRNA:Lys-(	0	NA	NA	NA	NA	NA
cbc	6.033976	-1.15586	3.930565	-0.29407	0.768705	NA
CG30062	5.549096	1.719007	3.959971	0.434096	0.664219	NA
tRNA:Ile-A	0	NA	NA	NA	NA	NA
CG30059	0.712213	-1.57119	3.998613	-0.39293	0.694369	NA
CG18278	9.25877	-1.69565	3.955021	-0.42873	0.668118	NA
CG10814	0.3141	1.240997	4.03841	0.307299	0.758616	NA
GstE14	0	NA	NA	NA	NA	NA
CG17047	0	NA	NA	NA	NA	NA
CG4734	0	NA	NA	NA	NA	NA
CG4714	49.76334	-3.38268	3.921748	-0.86254	0.388388	NA
CG4712	16.2704	-4.33197	3.90497	-1.10935	0.26728	NA
CG30058	0	NA	NA	NA	NA	NA
Orc3	0	NA	NA	NA	NA	NA
CG15870	11.21736	-1.69782	3.954309	-0.42936	0.667662	NA
CG13326	0	NA	NA	NA	NA	NA
Or49b	0.104623	0.639075	4.067674	0.157111	0.875158	NA
vg	0.697584	-1.11707	3.977017	-0.28088	0.778801	NA
sug	0	NA	NA	NA	NA	NA
Sans	0	NA	NA	NA	NA	NA
CG13321	0.178053	-1.16467	4.075067	-0.2858	0.775028	NA
CG13324	0	NA	NA	NA	NA	NA
CG13323	0	NA	NA	NA	NA	NA
CG30487	0	NA	NA	NA	NA	NA
Lfg	0	NA	NA	NA	NA	NA
Balat	0	NA	NA	NA	NA	NA
CG30486	0	NA	NA	NA	NA	NA
Cyp301a1	4.164135	-1.88038	3.815942	-0.49277	0.622176	NA
stil	0	NA	NA	NA	NA	NA
CG8646	55.04438	-8.22974	3.875036	-2.12379	0.033688	NA
wuc	0	NA	NA	NA	NA	NA
CG12374	0.178053	-1.16467	4.075067	-0.2858	0.775028	NA
CG17580	0.1047	0.613261	4.062799	0.150945	0.880019	NA
Mos	0.3141	1.240997	4.03841	0.307299	0.758616	NA
Obp49a	0	NA	NA	NA	NA	NA
CG30053	0.104623	0.639075	4.067674	0.157111	0.875158	NA

CG8785	7.984572	0.691827	3.929781	0.176047	0.860257	NA
CG30203	33.34724	1.501157	3.952192	0.379829	0.704072	NA
CG30046	0.178812	-1.08808	4.061735	-0.26789	0.788788	NA
tRNA:His-(	0	NA	NA	NA	NA	NA
tRNA:His-(	0	NA	NA	NA	NA	NA
CG30047	0.2094	1.005034	4.045341	0.248442	0.803792	NA
CG30049	0.712972	-2.55723	4.00634	-0.63829	0.523282	NA
CG30043	0	NA	NA	NA	NA	NA
CR33013	1.099666	-0.72019	3.971513	-0.18134	0.856102	NA
CG33012	0.356107	-1.47099	4.037764	-0.36431	0.715628	NA
CG13160	0	NA	NA	NA	NA	NA
CG13159	0	NA	NA	NA	NA	NA
Cpr49Aa	1.869955	-1.27799	3.894534	-0.32815	0.742799	NA
Cpr49Ab	0	NA	NA	NA	NA	NA
CG8501	0	NA	NA	NA	NA	NA
Cpr49Ac	0	NA	NA	NA	NA	NA
Or49a	0.356107	-1.47099	4.037764	-0.36431	0.715628	NA
Cpr49Ae	60.83066	1.751298	3.951762	0.443169	0.657644	NA
Cpr49Af	0	NA	NA	NA	NA	NA
Cpr49Ag	0.2094	1.005034	4.045341	0.248442	0.803792	NA
CG30050	0.178053	-1.16467	4.075067	-0.2858	0.775028	NA
Cpr49Ah	0	NA	NA	NA	NA	NA
CG13157	0.178053	-1.16467	4.075067	-0.2858	0.775028	NA
Dera	58.41285	-3.42598	3.843202	-0.89144	0.372694	NA
s-cup	27.96254	-0.38576	3.940911	-0.09789	0.922023	NA
vis	47.73975	-9.05778	3.907933	-2.31779	0.020461	NA
dgt5	0.209247	1.042804	4.047837	0.25762	0.7967	NA
CG30334	6.42765	1.758232	3.853888	0.456223	0.64823	NA
CG13154	0.178053	-1.16467	4.075067	-0.2858	0.775028	NA
CG8834	0	NA	NA	NA	NA	NA
Cpr49Ad	0	NA	NA	NA	NA	NA
CG13155	0.178053	-1.16467	4.075067	-0.2858	0.775028	NA
tRNA:His-(	1.246373	-1.6238	3.979526	-0.40804	0.683245	NA
tRNA:His-(	0	NA	NA	NA	NA	NA
tRNA:His-(	0	NA	NA	NA	NA	NA
CG8850	0	NA	NA	NA	NA	NA
CG13186	0.356107	-1.47099	4.037764	-0.36431	0.715628	NA
CG13171	0	NA	NA	NA	NA	NA
CG13170	0	NA	NA	NA	NA	NA
CG13168	0	NA	NA	NA	NA	NA
Vha36-2	0	NA	NA	NA	NA	NA
CG8854	0	NA	NA	NA	NA	NA
Sr-CII	0	NA	NA	NA	NA	NA
Cyp6g2	0	NA	NA	NA	NA	NA
OSCP1	0	NA	NA	NA	NA	NA
SkpB	0	NA	NA	NA	NA	NA
CG8888	0	NA	NA	NA	NA	NA
CG30039	0.1047	0.613261	4.062799	0.150945	0.880019	NA
CG13193	0	NA	NA	NA	NA	NA
snoRNA:M	5.78152	-0.74901	3.771122	-0.19862	0.842562	NA

exp	9.109486	-1.8306	3.811139	-0.48033	0.630994	NA
RnrS	0.178053	-1.16467	4.075067	-0.2858	0.775028	NA
CG30037	0	NA	NA	NA	NA	NA
CG30036	0	NA	NA	NA	NA	NA
reb	0.178053	-1.16467	4.075067	-0.2858	0.775028	NA
Damm	4.871543	-2.71574	3.859855	-0.70359	0.48169	NA
Twdlbeta	0	NA	NA	NA	NA	NA
Sobp	7.407411	-1.45501	3.891103	-0.37393	0.708454	NA
Ir48c	15.44553	-2.96766	3.841902	-0.77244	0.439851	NA
tRNA:Met-	0	NA	NA	NA	NA	NA
CG13198	4.842162	-5.02909	3.937067	-1.27737	0.201472	NA
Tret1-2	4.163375	-3.32944	3.863315	-0.86181	0.388793	NA
Ir48b	0	NA	NA	NA	NA	NA
Ir48a	0	NA	NA	NA	NA	NA
CG13196	0	NA	NA	NA	NA	NA
tRNA:Met-	0.676061	-1.55209	3.991909	-0.38881	0.697417	NA
tRNA:Arg-	0	NA	NA	NA	NA	NA
CG13228	0.356107	-1.47099	4.037764	-0.36431	0.715628	NA
CG13227	0	NA	NA	NA	NA	NA
CG13226	0	NA	NA	NA	NA	NA
CR30029	0	NA	NA	NA	NA	NA
Or47a	0	NA	NA	NA	NA	NA
Cpr47Eb	0.233093	0.692656	4.076246	0.169925	0.865069	NA
CG13223	0	NA	NA	NA	NA	NA
Cpr47Ee	0.356107	-1.47099	4.037764	-0.36431	0.715628	NA
CG30033	53.44971	-0.65528	3.854454	-0.17001	0.865005	NA
sha	0.282753	-0.25398	4.020204	-0.06318	0.949626	NA
lambdaTry	0	NA	NA	NA	NA	NA
betaTry	0.492076	0.930335	3.992579	0.233016	0.815749	NA
CG30031	0	NA	NA	NA	NA	NA
deltaTry	0	NA	NA	NA	NA	NA
iotaTry	0	NA	NA	NA	NA	NA
Gr47b	0.712213	-1.57119	3.998613	-0.39293	0.694369	NA
tRNA:Thr-1	0	NA	NA	NA	NA	NA
tRNA:Thr-1	0.178053	-1.16467	4.075067	-0.2858	0.775028	NA
Tsp47F	0	NA	NA	NA	NA	NA
tRNA:SeC-	1.631653	1.102918	3.977952	0.277258	0.781582	NA
CG13202	0	NA	NA	NA	NA	NA
CG30025	0	NA	NA	NA	NA	NA
gammaTry	0	NA	NA	NA	NA	NA
epsilonTry	0.233093	0.692656	4.076246	0.169925	0.865069	NA
alphaTry	0	NA	NA	NA	NA	NA
thetaTry	0	NA	NA	NA	NA	NA
etaTry	0	NA	NA	NA	NA	NA
zetaTry	0	NA	NA	NA	NA	NA
kappaTry	0	NA	NA	NA	NA	NA
Or47b	0	NA	NA	NA	NA	NA
Obp47b	0	NA	NA	NA	NA	NA
CG18335	0	NA	NA	NA	NA	NA
TpnC47D	0	NA	NA	NA	NA	NA

Cpr47Ef	0.732439	2.426673	3.9948	0.607458	0.543547	NA
Cpr47Ed	0.104623	0.639075	4.067674	0.157111	0.875158	NA
Cpr47Ec	0	NA	NA	NA	NA	NA
Cpr47Ea	0	NA	NA	NA	NA	NA
Listericin	0	NA	NA	NA	NA	NA
CG13215	0	NA	NA	NA	NA	NA
CG13216	0.356107	-1.47099	4.037764	-0.36431	0.715628	NA
CG13217	0.941609	1.421141	3.984935	0.356628	0.72137	NA
CG13218	0	NA	NA	NA	NA	NA
CG9084	0.178053	-1.16467	4.075067	-0.2858	0.775028	NA
polyph	0	NA	NA	NA	NA	NA
Obp47a	0	NA	NA	NA	NA	NA
BBS4	7.328995	1.727313	3.957831	0.436429	0.662525	NA
CG13231	0	NA	NA	NA	NA	NA
CG13230	0	NA	NA	NA	NA	NA
Spn47C	0	NA	NA	NA	NA	NA
CAH13	43.29042	-1.02999	3.920407	-0.26272	0.792763	NA
mms4	6.024553	-2.47201	3.924044	-0.62997	0.528717	NA
CG30016	0	NA	NA	NA	NA	NA
CG33474	2.637141	-1.81621	3.890553	-0.46683	0.640625	NA
CG12896	23.8804	-3.71126	3.824901	-0.97029	0.331902	NA
CG33475	0	NA	NA	NA	NA	NA
CG33477	0.178053	-1.16467	4.075067	-0.2858	0.775028	NA
CG12898	0	NA	NA	NA	NA	NA
CG12917	0	NA	NA	NA	NA	NA
Or46a	0	NA	NA	NA	NA	NA
Hdc	44.45506	-5.83852	3.86123	-1.51209	0.130511	NA
CG12914	0	NA	NA	NA	NA	NA
CG12911	0	NA	NA	NA	NA	NA
CG12909	4.711497	1.713057	3.961514	0.432425	0.665433	NA
CG12902	0	NA	NA	NA	NA	NA
Obp46a	0	NA	NA	NA	NA	NA
lncRNA:CR	8.998797	-1.16537	3.822818	-0.30485	0.760484	NA
CG12209	0.357625	-1.47707	4.044794	-0.36518	0.714979	NA
CG12912	0	NA	NA	NA	NA	NA
Lime	0.756516	2.906385	3.966481	0.732736	0.463719	NA
CG12744	42.69616	-0.47209	3.884184	-0.12154	0.903262	NA
CG12923	57.31855	0.420785	3.8345	0.109737	0.912618	NA
CG12133	0	NA	NA	NA	NA	NA
CG12134	0	NA	NA	NA	NA	NA
eve	0	NA	NA	NA	NA	NA
CG12920	0.233093	0.692656	4.076246	0.169925	0.865069	NA
PIG-N	58.84852	-8.91412	3.845317	-2.31818	0.02044	NA
Def	0	NA	NA	NA	NA	NA
CG1441	0	NA	NA	NA	NA	NA
tRNA:iMet	11.93033	-2.91436	3.923013	-0.74289	0.45755	NA
CG30002	0	NA	NA	NA	NA	NA
CG1773	0	NA	NA	NA	NA	NA
CG10459	0.104623	0.639075	4.067674	0.157111	0.875158	NA
CG1698	6.50284	3.679278	3.897972	0.943896	0.345223	NA

Orc6	37.92535	-1.70516	3.951919	-0.43148	0.666123	NA
hebe	54.7262	0.712219	3.835088	0.185711	0.852671	NA
dila	0	NA	NA	NA	NA	NA
CG30001	0.282677	-0.09599	4.057934	-0.02366	0.981127	NA
CG1690	0.178053	-1.16467	4.075067	-0.2858	0.775028	NA
Wnt2	5.519652	-1.68736	3.957756	-0.42634	0.669859	NA
Or45b	0	NA	NA	NA	NA	NA
Alp6	0	NA	NA	NA	NA	NA
CG1827	1.822665	-0.02704	3.958245	-0.00683	0.99455	NA
CG30338	56.14706	-2.2755	3.952625	-0.57569	0.564823	NA
CG30339	4.576209	0.305937	3.955371	0.077347	0.938347	NA
CG30340	0	NA	NA	NA	NA	NA
Smyd4-1	0.178812	-1.08808	4.061735	-0.26789	0.788788	NA
CG13955	0	NA	NA	NA	NA	NA
CG8800	0	NA	NA	NA	NA	NA
CNT1	0	NA	NA	NA	NA	NA
CG8788	0	NA	NA	NA	NA	NA
Rad51D	42.71757	1.749839	3.952123	0.442759	0.65794	NA
unpg	0	NA	NA	NA	NA	NA
Tsen15	0	NA	NA	NA	NA	NA
CG8026	0	NA	NA	NA	NA	NA
CG8008	3.769197	1.70343	3.964029	0.429722	0.667398	NA
Boot	3.900384	-5.46701	3.920402	-1.3945	0.163166	NA
CG13742	0	NA	NA	NA	NA	NA
CG30350	0	NA	NA	NA	NA	NA
CG13749	7.119595	1.726543	3.958029	0.436213	0.662682	NA
CG13748	0	NA	NA	NA	NA	NA
CG13747	0	NA	NA	NA	NA	NA
CG8197	0	NA	NA	NA	NA	NA
Ance-4	15.48424	1.530975	3.953223	0.387273	0.698554	NA
CG8170	0.356107	-1.47099	4.037764	-0.36431	0.715628	NA
CG13744	0	NA	NA	NA	NA	NA
CG8172	0	NA	NA	NA	NA	NA
FANCI	0.712213	-1.57119	3.998613	-0.39293	0.694369	NA
Spt	53.36638	0.31979	3.950572	0.080948	0.935484	NA
CG8252	0	NA	NA	NA	NA	NA
CG14752	0	NA	NA	NA	NA	NA
UQCR-11L	10.55945	0.864524	3.954652	0.218609	0.826954	NA
tRNA:Leu-(	0	NA	NA	NA	NA	NA
Jon44E	0	NA	NA	NA	NA	NA
CG8738	0.178812	-1.08808	4.061735	-0.26789	0.788788	NA
CG12376	0.104623	0.639075	4.067674	0.157111	0.875158	NA
PGRP-SC1a	0	NA	NA	NA	NA	NA
PGRP-SC1b	0.132078	-1.13211	4.069918	-0.27817	0.780885	NA
PGRP-SC2	0.178053	-1.16467	4.075067	-0.2858	0.775028	NA
CG8746	0	NA	NA	NA	NA	NA
CG30356	0.4188	1.41068	4.034613	0.349644	0.726606	NA
CG14743	0.466186	0.959471	4.033986	0.237847	0.812	NA
CG14744	0	NA	NA	NA	NA	NA
CG8584	0	NA	NA	NA	NA	NA

CG8586	0	NA	NA	NA	NA	NA
CG30355	4.476411	-4.95604	3.938499	-1.25836	0.208262	NA
CG8642	0	NA	NA	NA	NA	NA
CG8736	3.975684	1.507544	3.959717	0.38072	0.703411	NA
CG12780	6.053811	-1.68915	3.957162	-0.42686	0.669482	NA
Lcp3	0	NA	NA	NA	NA	NA
Lcp4	0	NA	NA	NA	NA	NA
Cyp4e2	0.4188	1.41068	4.034613	0.349644	0.726606	NA
Cyp4e1	17.8166	6.236091	3.906317	1.596412	0.110397	NA
Mal-A2	0	NA	NA	NA	NA	NA
Mal-A4	2.144988	-3.20556	3.997331	-0.80193	0.422596	NA
Mal-A6	0	NA	NA	NA	NA	NA
Mal-A8	0.893302	-2.63419	4.009574	-0.65698	0.511197	NA
Mal-A7	0.178812	-1.08808	4.061735	-0.26789	0.788788	NA
Mal-A3	0	NA	NA	NA	NA	NA
Mal-A1	0.1047	0.613261	4.062799	0.150945	0.880019	NA
Cyp4ad1	0	NA	NA	NA	NA	NA
Lcp2	56.49656	1.537131	3.951545	0.388995	0.69728	NA
Lcp1Psi	0	NA	NA	NA	NA	NA
Lcp1	0.466186	0.959471	4.033986	0.237847	0.812	NA
CG2121	24.91085	3.934285	3.882925	1.013227	0.310952	NA
CG2127	8.069575	5.526343	3.91921	1.410066	0.15852	NA
cola	0	NA	NA	NA	NA	NA
swif	0	NA	NA	NA	NA	NA
hubl	0	NA	NA	NA	NA	NA
spaw	0	NA	NA	NA	NA	NA
sut1	52.40369	1.229032	3.863023	0.318153	0.750369	NA
sut2	0	NA	NA	NA	NA	NA
sut3	44.9711	-4.00921	3.819314	-1.04972	0.293847	NA
sand	10.55843	0.362766	3.839992	0.094471	0.924735	NA
Nup50	24.22524	-1.42522	3.906718	-0.36481	0.71525	NA
tRNA:Ile-A	2.564026	1.129155	3.968527	0.284527	0.776006	NA
Mlh1	0.722036	-1.99346	3.98794	-0.49987	0.617165	NA
CG14760	0	NA	NA	NA	NA	NA
CG30369	0	NA	NA	NA	NA	NA
CG14759	0	NA	NA	NA	NA	NA
CG30371	0.178812	-1.08808	4.061735	-0.26789	0.788788	NA
CG30375	0	NA	NA	NA	NA	NA
CG30376	0	NA	NA	NA	NA	NA
CG12126	0	NA	NA	NA	NA	NA
CG2291	0	NA	NA	NA	NA	NA
dpn	15.70146	-6.64616	3.914944	-1.69764	0.089576	NA
CG14756	1.602479	-1.64094	3.97352	-0.41297	0.67963	NA
CG8708	0	NA	NA	NA	NA	NA
CR30374	0	NA	NA	NA	NA	NA
CG12824	0	NA	NA	NA	NA	NA
PolZ1	0.356107	-1.47099	4.037764	-0.36431	0.715628	NA
CG30377	38.62953	-5.86643	3.829744	-1.53181	0.12557	NA
CG30379	0	NA	NA	NA	NA	NA
azot	0.418493	1.313669	4.019283	0.326842	0.743788	NA

CG30378	0	NA	NA	NA	NA	NA
Odc1	0.233093	0.692656	4.076246	0.169925	0.865069	NA
Odc2	0	NA	NA	NA	NA	NA
Hey	0.178053	-1.16467	4.075067	-0.2858	0.775028	NA
Dic3	0	NA	NA	NA	NA	NA
CG14763	61.78676	-3.995	3.887632	-1.02762	0.30413	NA
Sep-05	20.43145	-1.6585	3.846982	-0.43112	0.666383	NA
CG30380	0.1047	0.613261	4.062799	0.150945	0.880019	NA
Prosalpha1	0	NA	NA	NA	NA	NA
TTLL12	57.4356	-4.32895	3.794228	-1.14093	0.253899	NA
Gpo2	11.0393	-1.69765	3.954364	-0.42931	0.667697	NA
CG2070	0	NA	NA	NA	NA	NA
CG2065	0.337793	1.451235	4.039844	0.35923	0.719423	NA
Dgat2	0	NA	NA	NA	NA	NA
CG1946	0	NA	NA	NA	NA	NA
cn	0.132078	-1.13211	4.069918	-0.27817	0.780885	NA
tor	0.69928	1.019866	4.009445	0.254366	0.799213	NA
CG12826	0	NA	NA	NA	NA	NA
CG1339	0	NA	NA	NA	NA	NA
Rpe	13.54535	6.212589	3.914471	1.587083	0.112494	NA
CG12164	0	NA	NA	NA	NA	NA
CG11112	15.66869	-1.70075	3.953349	-0.43021	0.667046	NA
CG11113	0	NA	NA	NA	NA	NA
so	2.8794	0.337993	3.953187	0.085499	0.931865	NA
CG11125	41.35647	1.74968	3.952163	0.442715	0.657972	NA
fa2h	0	NA	NA	NA	NA	NA
kappaB-Ra	0	NA	NA	NA	NA	NA
CG30503	0	NA	NA	NA	NA	NA
CG11145	0	NA	NA	NA	NA	NA
CG1701	0	NA	NA	NA	NA	NA
Gr43a	0.710103	-0.24484	3.975022	-0.0616	0.950885	NA
Tsp42Ei	0.178053	-1.16467	4.075067	-0.2858	0.775028	NA
Tsp42El	34.39633	0.558152	3.883973	0.143706	0.885732	NA
Tsp42Eo	0	NA	NA	NA	NA	NA
Tsp42Er	36.65652	-2.27186	3.953529	-0.57464	0.565534	NA
CG12836	0.178053	-1.16467	4.075067	-0.2858	0.775028	NA
Spn43Aa	0	NA	NA	NA	NA	NA
CG11060	0	NA	NA	NA	NA	NA
CG33140	0	NA	NA	NA	NA	NA
CG30385	0	NA	NA	NA	NA	NA
CG30384	0.550724	1.292521	3.972655	0.325355	0.744913	NA
Or43a	0	NA	NA	NA	NA	NA
Spn43Ad	45.54681	-0.27118	3.835068	-0.07071	0.943628	NA
Cyp9b2	26.27968	1.746819	3.952878	0.441911	0.658554	NA
Cyp9b1	0	NA	NA	NA	NA	NA
CG12831	0	NA	NA	NA	NA	NA
tRNA:Thr-/	0	NA	NA	NA	NA	NA
tRNA:Thr-/	0	NA	NA	NA	NA	NA
Tsp42Eq	0	NA	NA	NA	NA	NA
Tsp42Ep	13.9432	-0.65937	3.927737	-0.16787	0.866682	NA

CG1674	29.4972	-6.15362	3.892941	-1.58071	0.113944	NA
CG32006	0.715249	-1.88064	4.034336	-0.46616	0.641102	NA
CR32009	0	NA	NA	NA	NA	NA
CR32010	0	NA	NA	NA	NA	NA
CR32011	0.1047	0.613261	4.062799	0.150945	0.880019	NA
CG11077	0.596546	0.421256	3.969213	0.106131	0.915479	NA
CR11023	0	NA	NA	NA	NA	NA
Cpsf160	9.238209	-1.83289	3.929843	-0.4664	0.640926	NA
betaNACte	0	NA	NA	NA	NA	NA
pb	0.341401	0.594712	4.003898	0.148533	0.881922	NA
Pxd	0.178812	-1.08808	4.061735	-0.26789	0.788788	NA
tRNA:Tyr-(	1.188699	-1.8233	3.980741	-0.45803	0.646931	NA
tRNA:Tyr-(	0	NA	NA	NA	NA	NA
tRNA:Tyr-(	0	NA	NA	NA	NA	NA
tRNA:Tyr-(	0	NA	NA	NA	NA	NA
tRNA:Tyr-(	0	NA	NA	NA	NA	NA
tRNA:Leu-(	0.46073	-0.17853	4.00793	-0.04454	0.964471	NA
tRNA:Leu-(	0.233093	0.692656	4.076246	0.169925	0.865069	NA
mia	17.33667	-4.8345	3.903313	-1.23856	0.215507	NA
CG10725	0	NA	NA	NA	NA	NA
Lsp2	0	NA	NA	NA	NA	NA
asRNA:CR1	0	NA	NA	NA	NA	NA
CG13064	0	NA	NA	NA	NA	NA
nompA	0.356107	-1.47099	4.037764	-0.36431	0.715628	NA
CG13970	0	NA	NA	NA	NA	NA
CG15553	0	NA	NA	NA	NA	NA
Cyp12d1-d	0	NA	NA	NA	NA	NA
Cyp12d1-p	0.492	1.04661	3.990653	0.262265	0.793117	NA
CG18853	1.251686	-2.06022	4.011654	-0.51356	0.60756	NA
nxf2	3.717393	0.271825	3.852595	0.070556	0.943751	NA
nan	50.25597	1.750571	3.951942	0.442965	0.657791	NA
CG33489	0	NA	NA	NA	NA	NA
CG33490	2.439542	0.538613	3.869507	0.139194	0.889297	NA
CG10931	0	NA	NA	NA	NA	NA
CG5002	0	NA	NA	NA	NA	NA
HLH54F	0	NA	NA	NA	NA	NA
CG6362	1.046999	1.598136	3.993006	0.400234	0.688984	NA
CG14488	0	NA	NA	NA	NA	NA
Sardh	0.368855	-0.37404	4.015709	-0.09314	0.925789	NA
Zasp66	3.674452	0.012501	3.939617	0.003173	0.997468	NA
GluRIIB	0.178053	-1.16467	4.075067	-0.2858	0.775028	NA
CG7730	0.178812	-1.08808	4.061735	-0.26789	0.788788	NA
TpnC73F	0.566189	-0.21299	4.010979	-0.0531	0.957651	NA
CG33493	44.05541	0.362719	3.834619	0.094591	0.92464	NA
CG33494	59.33321	4.105825	3.886482	1.056437	0.290768	NA
tRNA:Gly-(	0	NA	NA	NA	NA	NA
Cec2	0	NA	NA	NA	NA	NA
CG10910	0	NA	NA	NA	NA	NA
CG12783	0.523116	1.34877	4.007682	0.336546	0.736459	NA
Obp73a	1.512901	1.047032	3.89748	0.268643	0.788204	NA

salto	0	NA	NA	NA	NA	NA
sut4	6.850833	3.786056	3.83481	0.987286	0.323502	NA
CG14642	12.81654	7.110847	3.909828	1.818711	0.068956	NA
CG14692	0.178053	-1.16467	4.075067	-0.2858	0.775028	NA
CG14736	0	NA	NA	NA	NA	NA
Gr89a	0	NA	NA	NA	NA	NA
Tim17b2	0	NA	NA	NA	NA	NA
Mlc2	0	NA	NA	NA	NA	NA
CG2336	28.02054	0.443499	3.897798	0.113782	0.909411	NA
gem	1.602479	-1.64094	3.97352	-0.41297	0.67963	NA
CG30270	0	NA	NA	NA	NA	NA
mthl15	19.05693	-0.75858	3.736961	-0.20299	0.83914	NA
bt	2.366851	-3.31349	3.876975	-0.85466	0.39274	NA
Gss2	0	NA	NA	NA	NA	NA
Muc11A	20.00271	3.552593	3.864163	0.919369	0.357902	NA
CG3277	1.465799	1.63603	3.98226	0.410829	0.681198	NA
CR33318	1.569349	1.463746	3.972245	0.368493	0.712505	NA
CR33319	0	NA	NA	NA	NA	NA
CG33459	0	NA	NA	NA	NA	NA
CG33467	0.178053	-1.16467	4.075067	-0.2858	0.775028	NA
CREG	15.51291	-0.89745	3.872436	-0.23175	0.816729	NA
Ugt49B2	0	NA	NA	NA	NA	NA
CG7824	2.164524	-2.16108	3.916813	-0.55174	0.581124	NA
CG8852	0.178053	-1.16467	4.075067	-0.2858	0.775028	NA
lncRNA:CR	8.094503	2.573403	3.872154	0.664592	0.506311	NA
Jheh2	13.67116	0.36537	3.838222	0.095193	0.924162	NA
att-ORFB	0	NA	NA	NA	NA	NA
alpha-Est4	0	NA	NA	NA	NA	NA
Cyp4aa1	0	NA	NA	NA	NA	NA
Lsd-1	11.26517	-2.24925	3.959234	-0.5681	0.569965	NA
CG10853	2.381275	1.651403	3.949799	0.418098	0.675875	NA
FoxL1	30.90618	-1.91869	3.952168	-0.48548	0.627338	NA
Gr64a	0	NA	NA	NA	NA	NA
Gr64b	0	NA	NA	NA	NA	NA
Gr64c	0	NA	NA	NA	NA	NA
CG14984	0	NA	NA	NA	NA	NA
CG14983	0	NA	NA	NA	NA	NA
CG32599	0	NA	NA	NA	NA	NA
CG1461	0	NA	NA	NA	NA	NA
CG9400	0	NA	NA	NA	NA	NA
CG11674	0.178053	-1.16467	4.075067	-0.2858	0.775028	NA
peb	4.354582	-4.13202	3.889311	-1.0624	0.288053	NA
pon	0	NA	NA	NA	NA	NA
CG15473	25.3609	-7.34659	3.91328	-1.87735	0.06047	NA
CG3062	0	NA	NA	NA	NA	NA
CG3081	0.356866	-1.92326	4.037889	-0.4763	0.633858	NA
CG12684	0	NA	NA	NA	NA	NA
Chd3	0	NA	NA	NA	NA	NA
CG32214	1.450033	2.807816	3.907765	0.718522	0.472435	NA
CG12519	0.178053	-1.16467	4.075067	-0.2858	0.775028	NA

CG32213	0	NA	NA	NA	NA	NA
CG32212	0.209247	1.042804	4.047837	0.25762	0.7967	NA
Cpr76Bb	0.178053	-1.16467	4.075067	-0.2858	0.775028	NA
Cpr76Ba	0	NA	NA	NA	NA	NA
CG9452	11.51692	2.942648	3.926413	0.74945	0.453586	NA
CG9449	0	NA	NA	NA	NA	NA
brv1	0.178053	-1.16467	4.075067	-0.2858	0.775028	NA
hpRNA:CR:	61.60795	-3.52692	3.901503	-0.90399	0.366001	NA
CG18294	0	NA	NA	NA	NA	NA
825-Oak	0	NA	NA	NA	NA	NA
CG14096	0	NA	NA	NA	NA	NA
CG14095	0.178053	-1.16467	4.075067	-0.2858	0.775028	NA
CG33062	0	NA	NA	NA	NA	NA
CG1835	0	NA	NA	NA	NA	NA
hydra	0	NA	NA	NA	NA	NA
Ser7	0	NA	NA	NA	NA	NA
CG1986	0	NA	NA	NA	NA	NA
CG15249	0	NA	NA	NA	NA	NA
CG15247	24.70918	1.746322	3.953003	0.441771	0.658655	NA
CG33502	0	NA	NA	NA	NA	NA
CG32819	0	NA	NA	NA	NA	NA
CG17450	0	NA	NA	NA	NA	NA
CG32820	0	NA	NA	NA	NA	NA
RpS10a	8.103901	1.37305	3.955703	0.347106	0.728511	NA
CG12880	0.104623	0.639075	4.067674	0.157111	0.875158	NA
CG31055	1.246373	-1.6238	3.979526	-0.40804	0.683245	NA
Mlc1	0.356107	-1.47099	4.037764	-0.36431	0.715628	NA
CG5612	1.120353	0.8435	3.975033	0.212199	0.831951	NA
Crg-1	14.09855	6.466143	3.912917	1.652512	0.09843	NA
CG2901	0	NA	NA	NA	NA	NA
CR33221	0.104623	0.639075	4.067674	0.157111	0.875158	NA
CG15625	0	NA	NA	NA	NA	NA
CG3036	3.70255	-0.58155	3.863881	-0.15051	0.880362	NA
ND-49L	0	NA	NA	NA	NA	NA
Cdc23	6.992797	1.160607	3.957551	0.293264	0.769321	NA
CG31683	0	NA	NA	NA	NA	NA
CG15130	0	NA	NA	NA	NA	NA
TpnC25D	0.942299	1.584447	3.996979	0.396411	0.691802	NA
CG12934	0.233093	0.692656	4.076246	0.169925	0.865069	NA
CG17601	0	NA	NA	NA	NA	NA
Cluap1	0	NA	NA	NA	NA	NA
Obp51a	0.233093	0.692656	4.076246	0.169925	0.865069	NA
Smyd4-3	0.104623	0.639075	4.067674	0.157111	0.875158	NA
CG7763	0.2094	1.005034	4.045341	0.248442	0.803792	NA
Drip	0.178812	-1.08808	4.061735	-0.26789	0.788788	NA
CG30026	0	NA	NA	NA	NA	NA
COX4L	0	NA	NA	NA	NA	NA
IFT20	0	NA	NA	NA	NA	NA
tRNA:Glu-(	0	NA	NA	NA	NA	NA
tRNA:Glu-(	0	NA	NA	NA	NA	NA

5SrRNA:CF	0	NA	NA	NA	NA	NA
5SrRNA:CF	0	NA	NA	NA	NA	NA
5SrRNA:CF	0	NA	NA	NA	NA	NA
5SrRNA-Ps	0	NA	NA	NA	NA	NA
5SrRNA:CF	0	NA	NA	NA	NA	NA
5SrRNA:CF	0	NA	NA	NA	NA	NA
5SrRNA:CF	0	NA	NA	NA	NA	NA
5SrRNA:CF	0	NA	NA	NA	NA	NA
5SrRNA:CF	0	NA	NA	NA	NA	NA
5SrRNA:CF	0	NA	NA	NA	NA	NA
5SrRNA-Ps	0	NA	NA	NA	NA	NA
5SrRNA:CF	0	NA	NA	NA	NA	NA
5SrRNA:CF	0	NA	NA	NA	NA	NA
5SrRNA:CF	0	NA	NA	NA	NA	NA
5SrRNA:CF	0	NA	NA	NA	NA	NA
5SrRNA:CF	0	NA	NA	NA	NA	NA
5SrRNA:CF	0	NA	NA	NA	NA	NA
5SrRNA:CF	0	NA	NA	NA	NA	NA
5SrRNA-Ps	0	NA	NA	NA	NA	NA
5SrRNA:CF	0	NA	NA	NA	NA	NA
5SrRNA:CF	0	NA	NA	NA	NA	NA
5SrRNA:CF	0	NA	NA	NA	NA	NA
5SrRNA:CF	0	NA	NA	NA	NA	NA
5SrRNA:CF	0	NA	NA	NA	NA	NA
5SrRNA:CF	0	NA	NA	NA	NA	NA
5SrRNA:CF	0	NA	NA	NA	NA	NA
5SrRNA:CF	0	NA	NA	NA	NA	NA
5SrRNA:CF	0	NA	NA	NA	NA	NA
5SrRNA:CF	0	NA	NA	NA	NA	NA
5SrRNA:CF	0	NA	NA	NA	NA	NA
5SrRNA:CF	0	NA	NA	NA	NA	NA
5SrRNA:CF	0	NA	NA	NA	NA	NA
5SrRNA:CF	0	NA	NA	NA	NA	NA
5SrRNA:CF	0	NA	NA	NA	NA	NA
5SrRNA:CF	0	NA	NA	NA	NA	NA
5SrRNA:CF	0	NA	NA	NA	NA	NA
5SrRNA:CF	0	NA	NA	NA	NA	NA
5SrRNA:CF	0	NA	NA	NA	NA	NA
5SrRNA:CF	0	NA	NA	NA	NA	NA
5SrRNA:CF	0	NA	NA	NA	NA	NA
5SrRNA:CF	0	NA	NA	NA	NA	NA
5SrRNA:CF	0	NA	NA	NA	NA	NA
5SrRNA:CF	0	NA	NA	NA	NA	NA
5SrRNA:CF	0	NA	NA	NA	NA	NA
5SrRNA:CF	0	NA	NA	NA	NA	NA
5SrRNA:CF	0	NA	NA	NA	NA	NA
5SrRNA:CF	0	NA	NA	NA	NA	NA
5SrRNA:CF	0	NA	NA	NA	NA	NA
5SrRNA:CF	0	NA	NA	NA	NA	NA
5SrRNA:CF	0	NA	NA	NA	NA	NA

5SrRNA:CF	0	NA	NA	NA	NA	NA
5SrRNA:CF	0	NA	NA	NA	NA	NA
5SrRNA:CF	0	NA	NA	NA	NA	NA
5SrRNA:CF	0	NA	NA	NA	NA	NA
5SrRNA:CF	0	NA	NA	NA	NA	NA
5SrRNA:CF	0	NA	NA	NA	NA	NA
5SrRNA:CF	0	NA	NA	NA	NA	NA
5SrRNA:CF	0	NA	NA	NA	NA	NA
5SrRNA:CF	0	NA	NA	NA	NA	NA
5SrRNA:CF	0	NA	NA	NA	NA	NA
5SrRNA:CF	0	NA	NA	NA	NA	NA
5SrRNA:CF	0	NA	NA	NA	NA	NA
5SrRNA:CF	0	NA	NA	NA	NA	NA
5SrRNA-Ps	0	NA	NA	NA	NA	NA
5SrRNA:CF	0	NA	NA	NA	NA	NA
5SrRNA:CF	0	NA	NA	NA	NA	NA
5SrRNA:CF	0	NA	NA	NA	NA	NA
5SrRNA:CF	0	NA	NA	NA	NA	NA
5SrRNA:CF	0	NA	NA	NA	NA	NA
5SrRNA:CF	0	NA	NA	NA	NA	NA
5SrRNA:CF	0	NA	NA	NA	NA	NA
5SrRNA:CF	0	NA	NA	NA	NA	NA
5SrRNA:CF	0	NA	NA	NA	NA	NA
5SrRNA:CF	0	NA	NA	NA	NA	NA
5SrRNA:CF	0	NA	NA	NA	NA	NA
5SrRNA:CF	0	NA	NA	NA	NA	NA
5SrRNA:CF	0	NA	NA	NA	NA	NA
5SrRNA:CF	0	NA	NA	NA	NA	NA
5SrRNA:CF	0	NA	NA	NA	NA	NA
5SrRNA:CF	0	NA	NA	NA	NA	NA
5SrRNA:CF	0	NA	NA	NA	NA	NA
5SrRNA:CF	0	NA	NA	NA	NA	NA
5SrRNA:CF	0	NA	NA	NA	NA	NA
5SrRNA:CF	0	NA	NA	NA	NA	NA
5SrRNA:CF	0	NA	NA	NA	NA	NA
5SrRNA:CF	0	NA	NA	NA	NA	NA
5SrRNA:CF	0	NA	NA	NA	NA	NA
5SrRNA:CF	0	NA	NA	NA	NA	NA
5SrRNA:CF	0	NA	NA	NA	NA	NA
5SrRNA:CF	0	NA	NA	NA	NA	NA
5SrRNA:CF	0	NA	NA	NA	NA	NA
5SrRNA:CF	0	NA	NA	NA	NA	NA
5SrRNA:CF	0	NA	NA	NA	NA	NA
5SrRNA:CF	0	NA	NA	NA	NA	NA
5SrRNA:CF	0	NA	NA	NA	NA	NA
5SrRNA:CF	0	NA	NA	NA	NA	NA
5SrRNA:CF	0	NA	NA	NA	NA	NA
5SrRNA:CF	0	NA	NA	NA	NA	NA
5SrRNA:CF	0	NA	NA	NA	NA	NA
5SrRNA:CF	0	NA	NA	NA	NA	NA

TpnC41C	0	NA	NA	NA	NA	NA
CG12395	0	NA	NA	NA	NA	NA
Ste:CG332	0	NA	NA	NA	NA	NA
Ste:CG332	0	NA	NA	NA	NA	NA
Ste:CG332	0	NA	NA	NA	NA	NA
Ste:CG332	0	NA	NA	NA	NA	NA
Ste:CG332	0	NA	NA	NA	NA	NA
Ste:CG332	0	NA	NA	NA	NA	NA
Ste:CG332	0	NA	NA	NA	NA	NA
Ste:CG332	0	NA	NA	NA	NA	NA
Ste:CG332	0	NA	NA	NA	NA	NA
Ste:CG332	0	NA	NA	NA	NA	NA
Ste:CG332	0	NA	NA	NA	NA	NA
Ste:CG332	0	NA	NA	NA	NA	NA
CG5853	0	NA	NA	NA	NA	NA
Cpr30F	0	NA	NA	NA	NA	NA
TbCMF46	0	NA	NA	NA	NA	NA
Muc30E	6.52661	1.15925	3.958017	0.292887	0.769609	NA
aspr	0.178053	-1.16467	4.075067	-0.2858	0.775028	NA
Peritrophi	0.178053	-1.16467	4.075067	-0.2858	0.775028	NA
CR33498	0	NA	NA	NA	NA	NA
Sdic4	0	NA	NA	NA	NA	NA
CR33487	0	NA	NA	NA	NA	NA
Sdic3	0	NA	NA	NA	NA	NA
CR33496	0	NA	NA	NA	NA	NA
Sdic2	0	NA	NA	NA	NA	NA
CR33491	0	NA	NA	NA	NA	NA
Sdic1	0	NA	NA	NA	NA	NA
Phf7	0	NA	NA	NA	NA	NA
CG9777	7.88318	3.79196	3.882872	0.976586	0.328774	NA
CG9782	0.536437	-1.71175	4.038042	-0.42391	0.671635	NA
CG9903	0	NA	NA	NA	NA	NA
CG10822	0	NA	NA	NA	NA	NA
CG12158	21.13696	6.666106	3.898853	1.709761	0.08731	NA
CG12446	13.61099	1.739652	3.954685	0.439896	0.660012	NA
CG13443	0	NA	NA	NA	NA	NA
Br140	47.92664	-5.00969	3.781911	-1.32464	0.185289	NA
CG1850	0	NA	NA	NA	NA	NA
snsl	0	NA	NA	NA	NA	NA
Marcal1	30.99118	1.748008	3.952581	0.442245	0.658312	NA
CG8654	0	NA	NA	NA	NA	NA
Jon25Bii	0	NA	NA	NA	NA	NA
Jon25Biii	28.00047	-1.91823	3.952298	-0.48535	0.627431	NA
U3-55K	0	NA	NA	NA	NA	NA
ppk13	0	NA	NA	NA	NA	NA
CG33509	0.356107	-1.47099	4.037764	-0.36431	0.715628	NA
CG33510	0.178053	-1.16467	4.075067	-0.2858	0.775028	NA
CG33511	0	NA	NA	NA	NA	NA
CG33514	0	NA	NA	NA	NA	NA
Unc-89	10.61662	2.412086	3.927885	0.614093	0.539154	NA

Acp53C14(	0	NA	NA	NA	NA	NA
lectin-37D	56.69724	-8.2434	3.885137	-2.12178	0.033856	NA
upd3	0	NA	NA	NA	NA	NA
tRNA:Arg-'	0	NA	NA	NA	NA	NA
snRNA:U7	5.018952	-1.89674	3.958511	-0.47915	0.631829	NA
tRNA:Ala-/	0	NA	NA	NA	NA	NA
tRNA:Glu-'	0.53416	-1.53481	4.012395	-0.38252	0.702078	NA
tRNA:Leu-'	0	NA	NA	NA	NA	NA
tRNA:Asn-(	0	NA	NA	NA	NA	NA
tRNA:Arg-(	0.1047	0.613261	4.062799	0.150945	0.880019	NA
tRNA:Trp-(	1.361099	1.628413	3.98439	0.408698	0.682761	NA
SmydA-8	0	NA	NA	NA	NA	NA
CG33552	0	NA	NA	NA	NA	NA
CG14300	0	NA	NA	NA	NA	NA
CG13376	0	NA	NA	NA	NA	NA
CG33557	0	NA	NA	NA	NA	NA
eyg	0.411147	-0.29058	4.046973	-0.0718	0.94276	NA
osk	0	NA	NA	NA	NA	NA
MstProx	2.90234	0.112359	3.880728	0.028953	0.976902	NA
armi	2.168104	-3.94426	3.966742	-0.99433	0.320061	NA
mud	0	NA	NA	NA	NA	NA
oc	57.59648	-2.15744	3.189781	-0.67636	0.498812	NA
AQP	22.58384	1.661836	3.953052	0.420393	0.674198	NA
polo	0	NA	NA	NA	NA	NA
l(3)mbn	0	NA	NA	NA	NA	NA
Gr47a	0	NA	NA	NA	NA	NA
tll	0	NA	NA	NA	NA	NA
unc	0.492153	0.308117	3.993933	0.077146	0.938507	NA
lectin-30A	0	NA	NA	NA	NA	NA
jhamt	0	NA	NA	NA	NA	NA
bw	0	NA	NA	NA	NA	NA
dj	0	NA	NA	NA	NA	NA
Dec-01	0	NA	NA	NA	NA	NA
lectin-21C:	0	NA	NA	NA	NA	NA
CG30048	10.65817	-4.28773	3.889354	-1.10243	0.270276	NA
CG31266	0	NA	NA	NA	NA	NA
Muc12Ea	0	NA	NA	NA	NA	NA
gsb	0	NA	NA	NA	NA	NA
pug	12.4153	-1.91223	3.954028	-0.48362	0.628659	NA
CG14683	0	NA	NA	NA	NA	NA
FucTB	43.84404	-1.04519	3.779126	-0.27657	0.78211	NA
Mp20	0	NA	NA	NA	NA	NA
Doc2	0	NA	NA	NA	NA	NA
thr	0.233093	0.692656	4.076246	0.169925	0.865069	NA
CG6614	0.2094	1.005034	4.045341	0.248442	0.803792	NA
prd	0.282677	-0.09599	4.057934	-0.02366	0.981127	NA
up	18.89962	-7.35467	3.911	-1.88051	0.060039	NA
en	0.282753	-0.25398	4.020204	-0.06318	0.949626	NA
PK1-R	0.356107	-1.47099	4.037764	-0.36431	0.715628	NA
Gr22b	0	NA	NA	NA	NA	NA

CG15109	0	NA	NA	NA	NA	NA
CG17681	0	NA	NA	NA	NA	NA
ppk7	0.357625	-1.47707	4.044794	-0.36518	0.714979	NA
CG6299	0	NA	NA	NA	NA	NA
CapaR	0.1047	0.613261	4.062799	0.150945	0.880019	NA
CG15117	3.188386	-2.55369	3.944197	-0.64746	0.517337	NA
ppk23	0	NA	NA	NA	NA	NA
ppk21	0.178053	-1.16467	4.075067	-0.2858	0.775028	NA
ppk20	0.178812	-1.08808	4.061735	-0.26789	0.788788	NA
lncRNA:iak	0.178053	-1.16467	4.075067	-0.2858	0.775028	NA
tRNA:Val-(	0	NA	NA	NA	NA	NA
Prx5	0	NA	NA	NA	NA	NA
CG7215	0	NA	NA	NA	NA	NA
CG11889	0	NA	NA	NA	NA	NA
CG11891	0	NA	NA	NA	NA	NA
CG11714	0	NA	NA	NA	NA	NA
Shawn	0	NA	NA	NA	NA	NA
Tyler	0	NA	NA	NA	NA	NA
CG33155	40.95183	-0.39539	3.583194	-0.11035	0.912134	NA
mRpL53	0	NA	NA	NA	NA	NA
CG33095	0	NA	NA	NA	NA	NA
CG33626	0	NA	NA	NA	NA	NA
CG33627	0	NA	NA	NA	NA	NA
snoRNA:U:	0.4188	1.41068	4.034613	0.349644	0.726606	NA
CG33630	0	NA	NA	NA	NA	NA
CG33631	0	NA	NA	NA	NA	NA
Vajk2	0	NA	NA	NA	NA	NA
snoRNA:M	0	NA	NA	NA	NA	NA
snoRNA:2	3.476414	2.98224	3.925791	0.759653	0.447462	NA
CheA7a	0	NA	NA	NA	NA	NA
CG33644	46.75667	-0.65325	3.834813	-0.17035	0.864737	NA
CG33645	2.670799	-1.66648	3.964795	-0.42032	0.674251	NA
CG33647	0	NA	NA	NA	NA	NA
CG33648	0	NA	NA	NA	NA	NA
GatC	0	NA	NA	NA	NA	NA
PolG2	0	NA	NA	NA	NA	NA
CG12849	0	NA	NA	NA	NA	NA
CG14929	0.1047	0.613261	4.062799	0.150945	0.880019	NA
CG33654	0	NA	NA	NA	NA	NA
snmRNA:2	0	NA	NA	NA	NA	NA
Hsp22	0	NA	NA	NA	NA	NA
CG4456	0	NA	NA	NA	NA	NA
snmRNA:3	0	NA	NA	NA	NA	NA
snmRNA:4	0	NA	NA	NA	NA	NA
snoRNA:U:	6.474162	-0.67346	3.787004	-0.17783	0.858854	NA
snoRNA:O	0	NA	NA	NA	NA	NA
CG33664	1.165466	1.076041	3.987865	0.269829	0.787292	NA
CG33665	0	NA	NA	NA	NA	NA
CG33666	1.165466	1.076041	3.987865	0.269829	0.787292	NA
CG33667	0	NA	NA	NA	NA	NA

CG33668	0.178812	-1.08808	4.061735	-0.26789	0.788788	NA
CG33669	0	NA	NA	NA	NA	NA
Phax	30.16797	2.730608	3.792957	0.719915	0.471577	NA
stg1	0	NA	NA	NA	NA	NA
CG11566	0	NA	NA	NA	NA	NA
CG33671	0	NA	NA	NA	NA	NA
CG33672	0	NA	NA	NA	NA	NA
CG33673	0	NA	NA	NA	NA	NA
snoRNA:M	4.141397	0.891021	3.960538	0.224975	0.821999	NA
CG14246	0	NA	NA	NA	NA	NA
CG14245	0	NA	NA	NA	NA	NA
LSm-4	0	NA	NA	NA	NA	NA
CG17768	0	NA	NA	NA	NA	NA
CG15892	9.090636	1.165026	3.956038	0.294493	0.768381	NA
srp	0	NA	NA	NA	NA	NA
CG33680	0	NA	NA	NA	NA	NA
E(spl)m6-E	0	NA	NA	NA	NA	NA
lab	12.87448	-2.25322	3.958224	-0.56925	0.569186	NA
snmRNA:6	0	NA	NA	NA	NA	NA
CG18538	53.45653	0.445875	3.851006	0.115781	0.907826	NA
CG18539	11.75377	0.698648	3.928498	0.177841	0.858848	NA
CG33483	0	NA	NA	NA	NA	NA
snoRNA:22	14.74165	-0.14721	3.802205	-0.03872	0.969116	NA
sea	29.51656	0.374199	3.83588	0.097552	0.922288	NA
CheB38c	0	NA	NA	NA	NA	NA
snmRNA:6	0	NA	NA	NA	NA	NA
CG31016	0.178053	-1.16467	4.075067	-0.2858	0.775028	NA
CG33687	0	NA	NA	NA	NA	NA
CG33688	0	NA	NA	NA	NA	NA
CG33689	0	NA	NA	NA	NA	NA
CG33690	0	NA	NA	NA	NA	NA
snoRNA:Ps	0.178053	-1.16467	4.075067	-0.2858	0.775028	NA
nompB	0	NA	NA	NA	NA	NA
snmRNA:4	0	NA	NA	NA	NA	NA
CheA75a	0	NA	NA	NA	NA	NA
CG33700	0	NA	NA	NA	NA	NA
CR33701	0	NA	NA	NA	NA	NA
CG33702	0	NA	NA	NA	NA	NA
CG33703	0	NA	NA	NA	NA	NA
CG33704	0	NA	NA	NA	NA	NA
snmRNA:2	3.177886	-3.37317	3.879402	-0.86951	0.384569	NA
CG10332	0	NA	NA	NA	NA	NA
IM18	0	NA	NA	NA	NA	NA
snmRNA:1	0	NA	NA	NA	NA	NA
alpha4GT2	15.07671	2.952152	3.92424	0.752286	0.451879	NA
snmRNA:7	0	NA	NA	NA	NA	NA
snmRNA:3	0	NA	NA	NA	NA	NA
anox	0	NA	NA	NA	NA	NA
SLIRP1	0	NA	NA	NA	NA	NA
scaRNA:M	10.33588	0.019886	3.743965	0.005311	0.995762	NA

PGRP-LD	10.67157	1.527223	3.954255	0.386223	0.699332	NA
Pmi	0	NA	NA	NA	NA	NA
Pif1B	0	NA	NA	NA	NA	NA
CG18749	0	NA	NA	NA	NA	NA
CG33722	0	NA	NA	NA	NA	NA
CG33723	0	NA	NA	NA	NA	NA
CG33725	0	NA	NA	NA	NA	NA
scaRNA:M	0	NA	NA	NA	NA	NA
snmRNA:4	0	NA	NA	NA	NA	NA
snmRNA:4	0	NA	NA	NA	NA	NA
snmRNA:4	0	NA	NA	NA	NA	NA
snmRNA:4	0	NA	NA	NA	NA	NA
snmRNA:4	0	NA	NA	NA	NA	NA
snmRNA:4	0	NA	NA	NA	NA	NA
snmRNA:4	0	NA	NA	NA	NA	NA
snmRNA:4	0	NA	NA	NA	NA	NA
snmRNA:4	0	NA	NA	NA	NA	NA
snmRNA:4	0	NA	NA	NA	NA	NA
snmRNA:4	0	NA	NA	NA	NA	NA
snmRNA:4	0	NA	NA	NA	NA	NA
snmRNA:4	0	NA	NA	NA	NA	NA
snmRNA:4	0	NA	NA	NA	NA	NA
snmRNA:4	0	NA	NA	NA	NA	NA
snmRNA:4	0	NA	NA	NA	NA	NA
snmRNA:4	0	NA	NA	NA	NA	NA
snmRNA:4	0	NA	NA	NA	NA	NA
snmRNA:4	0	NA	NA	NA	NA	NA
snmRNA:4	0	NA	NA	NA	NA	NA
primo-2	0	NA	NA	NA	NA	NA
primo-1	0	NA	NA	NA	NA	NA
CG13561	0	NA	NA	NA	NA	NA
snmRNA:1	0	NA	NA	NA	NA	NA
CG32269	0	NA	NA	NA	NA	NA
CG32270	0	NA	NA	NA	NA	NA
CG13430	0	NA	NA	NA	NA	NA
BORCS7	0	NA	NA	NA	NA	NA
CG33752	0	NA	NA	NA	NA	NA
CG33137	0	NA	NA	NA	NA	NA
CheB93b	7.656291	-1.69306	3.95587	-0.42799	0.66866	NA
Gr64d	0	NA	NA	NA	NA	NA
Gr64e	0	NA	NA	NA	NA	NA
snoRNA:Ps	2.301712	1.486073	3.965794	0.374723	0.707867	NA
Fum2	1.337962	-2.21334	3.917153	-0.56504	0.572048	NA
gdl-ORF39	0	NA	NA	NA	NA	NA
gdl	3.559184	4.427014	3.941475	1.123187	0.261358	NA
CG33757	0.1047	0.613261	4.062799	0.150945	0.880019	NA
CG33758	0.104623	0.639075	4.067674	0.157111	0.875158	NA
CG33764	0.356107	-1.47099	4.037764	-0.36431	0.715628	NA
CG33766	4.187997	1.708209	3.962778	0.431063	0.666422	NA
CG33767	0	NA	NA	NA	NA	NA

CG33768	0	NA	NA	NA	NA	NA
CG33769	0	NA	NA	NA	NA	NA
CG33770	0.104623	0.639075	4.067674	0.157111	0.875158	NA
CG33771	0	NA	NA	NA	NA	NA
tRNA:CR32	0	NA	NA	NA	NA	NA
CG30056	0	NA	NA	NA	NA	NA
snoRNA:M	0.132078	-1.13211	4.069918	-0.27817	0.780885	NA
snoRNA:66	0	NA	NA	NA	NA	NA
CG33783	0	NA	NA	NA	NA	NA
CG33784	0	NA	NA	NA	NA	NA
CG33785	0	NA	NA	NA	NA	NA
CG33786	0	NA	NA	NA	NA	NA
Or98b	0	NA	NA	NA	NA	NA
ste24c	0	NA	NA	NA	NA	NA
CG30461	0	NA	NA	NA	NA	NA
CG9130	0	NA	NA	NA	NA	NA
CG9133	0	NA	NA	NA	NA	NA
snRNA:U2:	0	NA	NA	NA	NA	NA
CG3588	0.236778	-0.26946	4.071573	-0.06618	0.947234	NA
snmRNA:7	0	NA	NA	NA	NA	NA
Kaz1-ORFB	14.44859	1.740504	3.954469	0.440136	0.659839	NA
Kaz1-ORFA	0	NA	NA	NA	NA	NA
CG18170	0	NA	NA	NA	NA	NA
CG33791	0	NA	NA	NA	NA	NA
CG33792	48.96465	-1.70586	3.951691	-0.43168	0.665975	NA
snmRNA:8	0	NA	NA	NA	NA	NA
snoRNA:68	0	NA	NA	NA	NA	NA
CG13175	0	NA	NA	NA	NA	NA
CG33795	0	NA	NA	NA	NA	NA
CG33796	0	NA	NA	NA	NA	NA
CG14518	0	NA	NA	NA	NA	NA
CG33463	0	NA	NA	NA	NA	NA
Argl	3.131606	-1.39628	3.960959	-0.35251	0.724455	NA
CR33797	0	NA	NA	NA	NA	NA
Adhr	0	NA	NA	NA	NA	NA
CG14492	0	NA	NA	NA	NA	NA
lncRNA:CR	18.41437	1.172608	3.953457	0.296603	0.766769	NA
His1:CG33	2.093079	3.901995	3.954223	0.986792	0.323745	NA
His-Psi:CR3	0	NA	NA	NA	NA	NA
His3:CG33	0	NA	NA	NA	NA	NA
His1:CG31	0	NA	NA	NA	NA	NA
His2A:CG3	0	NA	NA	NA	NA	NA
His3:CG31	0	NA	NA	NA	NA	NA
His1:CG33	0	NA	NA	NA	NA	NA
His-Psi:CR3	0	NA	NA	NA	NA	NA
His3:CG33	0	NA	NA	NA	NA	NA
His1:CG33	0	NA	NA	NA	NA	NA
His2A:CG3	0.356866	-1.92326	4.037889	-0.4763	0.633858	NA
His3:CG33	0	NA	NA	NA	NA	NA
His1:CG33	0	NA	NA	NA	NA	NA

His-Psi:CR3	0	NA	NA	NA	NA	NA
His3:CG33	0	NA	NA	NA	NA	NA
His1:CG33	0	NA	NA	NA	NA	NA
His2A:CG3	0	NA	NA	NA	NA	NA
His3:CG33	0	NA	NA	NA	NA	NA
His1:CG33	0	NA	NA	NA	NA	NA
His2A:CG3	0	NA	NA	NA	NA	NA
His3:CG33	0	NA	NA	NA	NA	NA
His1:CG33	0	NA	NA	NA	NA	NA
His2A:CG3	0	NA	NA	NA	NA	NA
His3:CG33	0	NA	NA	NA	NA	NA
His1:CG33	0	NA	NA	NA	NA	NA
His2A:CG3	0	NA	NA	NA	NA	NA
His3:CG33	0	NA	NA	NA	NA	NA
His1:CG33	0	NA	NA	NA	NA	NA
His2A:CG3	0	NA	NA	NA	NA	NA
His3:CG33	0	NA	NA	NA	NA	NA
His1:CG33	0	NA	NA	NA	NA	NA
His2A:CG3	0	NA	NA	NA	NA	NA
His3:CG33	0	NA	NA	NA	NA	NA
His1:CG33	0	NA	NA	NA	NA	NA
His2A:CG3	0	NA	NA	NA	NA	NA
His3:CG33	0	NA	NA	NA	NA	NA
His1:CG33	0	NA	NA	NA	NA	NA
His2A:CG3	0	NA	NA	NA	NA	NA
His3:CG33	0	NA	NA	NA	NA	NA
His1:CG33	0	NA	NA	NA	NA	NA
His2A:CG3	0	NA	NA	NA	NA	NA
His3:CG33	0	NA	NA	NA	NA	NA
His1:CG33	0	NA	NA	NA	NA	NA
His2A:CG3	0	NA	NA	NA	NA	NA
His3:CG33	0	NA	NA	NA	NA	NA
His1:CG33	0	NA	NA	NA	NA	NA
His2A:CG3	0	NA	NA	NA	NA	NA
His3:CG33	0	NA	NA	NA	NA	NA
His1:CG33	0	NA	NA	NA	NA	NA
His2A:CG3	0	NA	NA	NA	NA	NA
His3:CG33	0	NA	NA	NA	NA	NA
His1:CG33	0	NA	NA	NA	NA	NA
His2A:CG3	0	NA	NA	NA	NA	NA
His3:CG33	0	NA	NA	NA	NA	NA
His1:CG33	0	NA	NA	NA	NA	NA
His2A:CG3	0	NA	NA	NA	NA	NA
His3:CG33	0	NA	NA	NA	NA	NA
His1:CG33	0	NA	NA	NA	NA	NA
His2A:CG3	0	NA	NA	NA	NA	NA
His3:CG33	0	NA	NA	NA	NA	NA
His1:CG33	0	NA	NA	NA	NA	NA
His2A:CG3	0	NA	NA	NA	NA	NA
His3:CG33	0	NA	NA	NA	NA	NA

His1:CG33	0	NA	NA	NA	NA	NA
His2A:CG3	0	NA	NA	NA	NA	NA
His3:CG33	0	NA	NA	NA	NA	NA
His1:CG33	0	NA	NA	NA	NA	NA
His2A:CG3	0	NA	NA	NA	NA	NA
His3:CG33	0	NA	NA	NA	NA	NA
His-Psi:CR3	0	NA	NA	NA	NA	NA
His-Psi:CR3	7.529166	1.263047	3.766048	0.335377	0.73734	NA
His2B:CG3	7.761965	-3.16534	3.85797	-0.82047	0.411949	NA
His4:CG33	0	NA	NA	NA	NA	NA
His2B:CG3	0	NA	NA	NA	NA	NA
His4:CG33	0	NA	NA	NA	NA	NA
His2B:CG3	0	NA	NA	NA	NA	NA
His4:CG33	0	NA	NA	NA	NA	NA
His2B:CG3	0	NA	NA	NA	NA	NA
His4:CG33	0	NA	NA	NA	NA	NA
His2B:CG3	0	NA	NA	NA	NA	NA
His4:CG33	0	NA	NA	NA	NA	NA
His2B:CG3	0	NA	NA	NA	NA	NA
His4:CG33	0	NA	NA	NA	NA	NA
His2B:CG3	0	NA	NA	NA	NA	NA
His4:CG33	0	NA	NA	NA	NA	NA
His2B:CG3	0	NA	NA	NA	NA	NA
His4:CG33	0	NA	NA	NA	NA	NA
His2B:CG3	0	NA	NA	NA	NA	NA
His4:CG33	0	NA	NA	NA	NA	NA
His2B:CG3	0	NA	NA	NA	NA	NA
His4:CG33	0	NA	NA	NA	NA	NA
His2B:CG3	0	NA	NA	NA	NA	NA
His4:CG33	0	NA	NA	NA	NA	NA
His2B:CG3	0	NA	NA	NA	NA	NA
His4:CG33	0	NA	NA	NA	NA	NA
His2B:CG3	0	NA	NA	NA	NA	NA
His4:CG33	0	NA	NA	NA	NA	NA
His2B:CG3	0	NA	NA	NA	NA	NA
His4:CG33	0	NA	NA	NA	NA	NA
His2B:CG3	0	NA	NA	NA	NA	NA
His4:CG33	0	NA	NA	NA	NA	NA
His2B:CG3	0	NA	NA	NA	NA	NA
His4:CG33	0	NA	NA	NA	NA	NA
His2B:CG3	0	NA	NA	NA	NA	NA
His4:CG33	0	NA	NA	NA	NA	NA
His2B:CG3	0	NA	NA	NA	NA	NA
His4:CG33	0	NA	NA	NA	NA	NA
His2B:CG3	0	NA	NA	NA	NA	NA
His4:CG33	0	NA	NA	NA	NA	NA
His2B:CG3	0	NA	NA	NA	NA	NA
His4:CG33	0	NA	NA	NA	NA	NA
His2B:CG3	0	NA	NA	NA	NA	NA
His4:CG31	0	NA	NA	NA	NA	NA

His2B:CG1	0	NA	NA	NA	NA	NA
His4:CG33	0	NA	NA	NA	NA	NA
His2B:CG3	0	NA	NA	NA	NA	NA
seq	0	NA	NA	NA	NA	NA
CG33912	0	NA	NA	NA	NA	NA
snmRNA:1	0	NA	NA	NA	NA	NA
snmRNA:1	0	NA	NA	NA	NA	NA
snmRNA:4	0	NA	NA	NA	NA	NA
CG13203	0.2094	1.005034	4.045341	0.248442	0.803792	NA
Tim9b	0	NA	NA	NA	NA	NA
Pstk	0	NA	NA	NA	NA	NA
waw	0	NA	NA	NA	NA	NA
Snapin	0	NA	NA	NA	NA	NA
HemK2	0	NA	NA	NA	NA	NA
CG33476	0	NA	NA	NA	NA	NA
CG33919	0	NA	NA	NA	NA	NA
CG10096	0	NA	NA	NA	NA	NA
CG10097	0	NA	NA	NA	NA	NA
lncRNA:CR	0.178812	-1.08808	4.061735	-0.26789	0.788788	NA
CG33920	0	NA	NA	NA	NA	NA
snoRNA:U	3.894745	-3.71361	3.877216	-0.9578	0.338161	NA
CG33922	0	NA	NA	NA	NA	NA
CG33923	0	NA	NA	NA	NA	NA
CG33926	0	NA	NA	NA	NA	NA
CG33927	33.6958	4.558673	3.873744	1.176813	0.23927	NA
CG33928	0	NA	NA	NA	NA	NA
CheB38b	0	NA	NA	NA	NA	NA
CheB93a	0	NA	NA	NA	NA	NA
CR33929	0	NA	NA	NA	NA	NA
snoRNA:1	7.158366	3.643568	3.84445	0.947748	0.343258	NA
Rpp20	0	NA	NA	NA	NA	NA
CG33932	0	NA	NA	NA	NA	NA
Indy-2	0	NA	NA	NA	NA	NA
CG33934	0	NA	NA	NA	NA	NA
mei-218	0	NA	NA	NA	NA	NA
mei-217	0	NA	NA	NA	NA	NA
lncRNA:CR	0	NA	NA	NA	NA	NA
CG33939	8.557672	-0.51066	3.950186	-0.12928	0.89714	NA
lncRNA:CR	0	NA	NA	NA	NA	NA
BG642312	0	NA	NA	NA	NA	NA
CG33946	0	NA	NA	NA	NA	NA
lncRNA:CR	0	NA	NA	NA	NA	NA
Menl-2	0	NA	NA	NA	NA	NA
Menl-1	0	NA	NA	NA	NA	NA
CR33629	0	NA	NA	NA	NA	NA
CG33632	0.660388	-1.75977	4.001266	-0.4398	0.660079	NA
CG33721	0	NA	NA	NA	NA	NA
CG6503	0	NA	NA	NA	NA	NA
ric8b	0	NA	NA	NA	NA	NA
Ance-3	0.593643	-1.88241	3.976168	-0.47342	0.635911	NA

CG7179	0.178053	-1.16467	4.075067	-0.2858	0.775028	NA
CG14499	1.921676	3.569804	3.94628	0.9046	0.365677	NA
CG32803	44.83741	8.292158	3.90907	2.121261	0.0339	NA
CG15888	0	NA	NA	NA	NA	NA
CG7025	0.460806	-0.60052	3.997252	-0.15023	0.880581	NA
Cpr92A	0	NA	NA	NA	NA	NA
CG12184	4.689767	-2.12618	3.87838	-0.54821	0.583545	NA
twi	0	NA	NA	NA	NA	NA
CG30280	6.651163	5.148522	3.922546	1.312546	0.189336	NA
CG12078	0.639619	-1.41861	3.980326	-0.3564	0.721538	NA
CG18636	0	NA	NA	NA	NA	NA
CR15280	0	NA	NA	NA	NA	NA
CecA2	0	NA	NA	NA	NA	NA
CG6435	28.65416	4.501433	3.878719	1.160546	0.245826	NA
E(spl)m5-H	0	NA	NA	NA	NA	NA
CG13062	0	NA	NA	NA	NA	NA
CG13059	0	NA	NA	NA	NA	NA
CG13039	0	NA	NA	NA	NA	NA
Yp3	0.365171	-0.43584	4.056359	-0.10745	0.914434	NA
CG15116	0	NA	NA	NA	NA	NA
Cby	0	NA	NA	NA	NA	NA
Cp7Fa	26.81579	3.055367	3.752685	0.814182	0.415541	NA
lncRNA:CR	0	NA	NA	NA	NA	NA
CG33964	0	NA	NA	NA	NA	NA
CG7255	0.178812	-1.08808	4.061735	-0.26789	0.788788	NA
ovo	9.65601	4.262966	3.896461	1.094061	0.273928	NA
CG14400	0	NA	NA	NA	NA	NA
CG33965	0.178053	-1.16467	4.075067	-0.2858	0.775028	NA
CG33966	0	NA	NA	NA	NA	NA
Cyp6a16	0	NA	NA	NA	NA	NA
Anp	31.70405	-2.16135	3.953675	-0.54667	0.584607	NA
Mdr50	2.504861	-0.27875	3.903025	-0.07142	0.943065	NA
Cp15	0	NA	NA	NA	NA	NA
CG8927	0	NA	NA	NA	NA	NA
CG13538	0.178053	-1.16467	4.075067	-0.2858	0.775028	NA
glob3	0.178812	-1.08808	4.061735	-0.26789	0.788788	NA
CG32857	0	NA	NA	NA	NA	NA
bin	0.209323	1.414133	4.032409	0.350692	0.725819	NA
CG9313	3.061183	-4.80925	3.930009	-1.22372	0.221056	NA
hh	0.209323	1.414133	4.032409	0.350692	0.725819	NA
CG14302	0	NA	NA	NA	NA	NA
bsh	0.3141	1.240997	4.03841	0.307299	0.758616	NA
Obp50c	0	NA	NA	NA	NA	NA
Elo68alpha	1.072874	-2.0339	4.019685	-0.50598	0.612867	NA
CG13931	0	NA	NA	NA	NA	NA
CG15357	0	NA	NA	NA	NA	NA
NetA	0.907513	-0.58764	3.917565	-0.15	0.880764	NA
CG17195	0	NA	NA	NA	NA	NA
CG17198	0	NA	NA	NA	NA	NA
Cpr73D	34.93154	1.667959	3.898276	0.427871	0.668745	NA

CG4956	0	NA	NA	NA	NA	NA
CG31904	0	NA	NA	NA	NA	NA
CG3355	1.584932	-1.84571	3.973792	-0.46447	0.642311	NA
CG13319	52.97982	9.266427	3.844618	2.410233	0.015942	NA
msd1	0	NA	NA	NA	NA	NA
Taf12L	0	NA	NA	NA	NA	NA
asRNA:Eig(	34.21758	-1.64	3.951983	-0.41498	0.678156	NA
CG33309	0.341478	0.247543	4.031301	0.061405	0.951036	NA
tRNA:Asn-(	0	NA	NA	NA	NA	NA
tRNA:Asn-(	0.1047	0.613261	4.062799	0.150945	0.880019	NA
Cyt-b5-r	0.233093	0.692656	4.076246	0.169925	0.865069	NA
Z600	0	NA	NA	NA	NA	NA
CecA1	0	NA	NA	NA	NA	NA
Or65c	46.23827	-0.87619	3.94859	-0.2219	0.824393	NA
mtSSB	5.800391	0.33584	3.847088	0.087297	0.930435	NA
lectin-28C	0.31089	-1.9003	4.033427	-0.47114	0.637542	NA
gl	0.132078	-1.13211	4.069918	-0.27817	0.780885	NA
CecB	0	NA	NA	NA	NA	NA
CG12256	0	NA	NA	NA	NA	NA
CG31274	0.5892	-0.44935	3.992199	-0.11256	0.910381	NA
CG14247	0.934208	-0.58863	3.908901	-0.15059	0.880302	NA
Edg78E	0.712213	-1.57119	3.998613	-0.39293	0.694369	NA
CG17751	0	NA	NA	NA	NA	NA
nAChRbeta	0	NA	NA	NA	NA	NA
CG13038	0	NA	NA	NA	NA	NA
CG17377	0.233093	0.692656	4.076246	0.169925	0.865069	NA
Fbp2	0	NA	NA	NA	NA	NA
CG9459	0	NA	NA	NA	NA	NA
CG30087	0	NA	NA	NA	NA	NA
CG33460	0	NA	NA	NA	NA	NA
CG33461	0	NA	NA	NA	NA	NA
CG33462	0	NA	NA	NA	NA	NA
Act57B	0.314023	1.768229	4.015745	0.440324	0.659702	NA
CG15403	0	NA	NA	NA	NA	NA
CR9337	2.406335	1.488229	3.965178	0.375325	0.707419	NA
Muc68D	0.356107	-1.47099	4.037764	-0.36431	0.715628	NA
fab1	0	NA	NA	NA	NA	NA
obst-H	0	NA	NA	NA	NA	NA
CG33985	0.2094	1.005034	4.045341	0.248442	0.803792	NA
CG33986	0.104623	0.639075	4.067674	0.157111	0.875158	NA
run	0	NA	NA	NA	NA	NA
ACXC	0.178053	-1.16467	4.075067	-0.2858	0.775028	NA
lncRNA:CR	0	NA	NA	NA	NA	NA
Rpp21	57.01968	1.537151	3.951539	0.389001	0.697276	NA
sunn	0	NA	NA	NA	NA	NA
Dso2	3.566098	-1.88656	3.961501	-0.47622	0.633916	NA
CG18545	0	NA	NA	NA	NA	NA
CG4174	2.198698	1.671135	3.972627	0.420662	0.674002	NA
CG13380	0	NA	NA	NA	NA	NA
Edg84A	0	NA	NA	NA	NA	NA

phu	3.661814	1.504942	3.960447	0.379993	0.703951	NA
CG32023	0	NA	NA	NA	NA	NA
Andorra	0	NA	NA	NA	NA	NA
CG32625	42.42716	3.203495	3.907146	0.819907	0.412269	NA
CG31693	0	NA	NA	NA	NA	NA
CG13578	9.632393	1.733649	3.956211	0.438209	0.661235	NA
tgy	0	NA	NA	NA	NA	NA
CG7912	4.288459	-4.46903	3.945299	-1.13275	0.25732	NA
Jon25Bi	0	NA	NA	NA	NA	NA
CG12620	0	NA	NA	NA	NA	NA
CG14810	0	NA	NA	NA	NA	NA
CG30383	0.760277	0.501824	3.968793	0.126442	0.899382	NA
Vago	0	NA	NA	NA	NA	NA
eya	0	NA	NA	NA	NA	NA
CG5928	50.2355	-3.52118	3.899441	-0.903	0.366529	NA
CG13044	0	NA	NA	NA	NA	NA
yuri	32.42012	-6.23816	3.871193	-1.61143	0.107086	NA
CG33093	0.178053	-1.16467	4.075067	-0.2858	0.775028	NA
CG33993	2.951685	-3.33776	3.931437	-0.84899	0.395886	NA
CG15649	0	NA	NA	NA	NA	NA
CG6262	0.357625	-1.47707	4.044794	-0.36518	0.714979	NA
CG33301	0	NA	NA	NA	NA	NA
CG12481	0	NA	NA	NA	NA	NA
CG14630	0	NA	NA	NA	NA	NA
CG33995	0	NA	NA	NA	NA	NA
CG30098	0	NA	NA	NA	NA	NA
CG4927	0	NA	NA	NA	NA	NA
CG14062	0.264155	-1.54099	4.048976	-0.38059	0.703509	NA
CG33679	2.097839	1.11873	3.972242	0.281637	0.778222	NA
CG33258	0	NA	NA	NA	NA	NA
CG33998	8.568481	0.201195	3.845387	0.052321	0.958273	NA
CG34001	0	NA	NA	NA	NA	NA
CG34002	18.61218	2.713012	3.90385	0.694958	0.487082	NA
NimB3	18.0999	2.763288	3.919351	0.705037	0.480787	NA
CG34005	0	NA	NA	NA	NA	NA
CG33772	0.546832	-1.46767	3.995602	-0.36732	0.713379	NA
CR33294	0.104623	0.639075	4.067674	0.157111	0.875158	NA
CG33773	0	NA	NA	NA	NA	NA
CG33775	0	NA	NA	NA	NA	NA
CG33658	0	NA	NA	NA	NA	NA
lncRNA:CR	0	NA	NA	NA	NA	NA
CG34007	0	NA	NA	NA	NA	NA
CG34008	0.264155	-1.54099	4.048976	-0.38059	0.703509	NA
CG34011	0.80738	-2.35989	3.971695	-0.59418	0.552393	NA
CG34012	0.356107	-1.47099	4.037764	-0.36431	0.715628	NA
CG34021	0	NA	NA	NA	NA	NA
lncRNA:CR	0	NA	NA	NA	NA	NA
CG34026	18.85817	-0.01081	3.905228	-0.00277	0.997792	NA
CG34027	0	NA	NA	NA	NA	NA
CG34028	0	NA	NA	NA	NA	NA

CG34029	0	NA	NA	NA	NA	NA
CG34030	0.132078	-1.13211	4.069918	-0.27817	0.780885	NA
CG34031	0	NA	NA	NA	NA	NA
CG34033	0	NA	NA	NA	NA	NA
CG34034	0	NA	NA	NA	NA	NA
CG33465	0	NA	NA	NA	NA	NA
CG33798	0	NA	NA	NA	NA	NA
CG34038	0	NA	NA	NA	NA	NA
CG34040	0	NA	NA	NA	NA	NA
CG34041	0	NA	NA	NA	NA	NA
ppk10	30.21707	-7.38542	3.849408	-1.91859	0.055037	NA
CG34043	0	NA	NA	NA	NA	NA
CG34045	7.971396	-3.1233	3.925024	-0.79574	0.426183	NA
lncRNA:CR	0	NA	NA	NA	NA	NA
lncRNA:CR	0	NA	NA	NA	NA	NA
CG34049	0	NA	NA	NA	NA	NA
CG34051	2.082181	-0.96846	3.93372	-0.2462	0.805531	NA
lncRNA:CR	3.769197	1.70343	3.964029	0.429722	0.667398	NA
CG34054	0	NA	NA	NA	NA	NA
ppk16	0	NA	NA	NA	NA	NA
ppk11	0	NA	NA	NA	NA	NA
CG34056	13.88816	-1.6998	3.95366	-0.42993	0.667246	NA
CG34057	0.132078	-1.13211	4.069918	-0.27817	0.780885	NA
CG17140	0	NA	NA	NA	NA	NA
CG17139	0	NA	NA	NA	NA	NA
mt:tRNA:Il	56.75501	-0.46766	3.106641	-0.15053	0.880343	NA
mt:tRNA:G	15.67504	-0.47805	3.736175	-0.12795	0.898188	NA
mt:tRNA:M	0.442416	2.217191	3.994664	0.555038	0.578869	NA
mt:tRNA:T	3.437871	1.622276	3.861099	0.420159	0.674369	NA
mt:tRNA:T	26.01033	0.003978	3.731625	0.001066	0.999149	NA
mt:tRNA:L	2.372315	0.281774	3.890111	0.072433	0.942257	NA
mt:tRNA:L	29.04862	-0.02538	3.732135	-0.0068	0.994574	NA
mt:tRNA:A	0.550724	1.292521	3.972655	0.325355	0.744913	NA
mt:tRNA:A	0	NA	NA	NA	NA	NA
mt:tRNA:S	11.31347	-0.90891	3.748552	-0.24247	0.808417	NA
mt:tRNA:G	0.341401	0.594712	4.003898	0.148533	0.881922	NA
mt:tRNA:P	2.666879	-0.602	3.892713	-0.15465	0.8771	NA
mt:tRNA:H	13.90779	0.399512	3.739452	0.106837	0.914918	NA
mt:tRNA:T	1.830721	-1.23284	3.854615	-0.31984	0.749093	NA
mt:tRNA:P	0.104623	0.639075	4.067674	0.157111	0.875158	NA
mt:tRNA:S	1.844744	-1.64151	3.932701	-0.4174	0.676386	NA
mt:tRNA:L	1.531116	2.649163	3.91345	0.676938	0.498445	NA
mt:tRNA:V	2.714169	-1.89778	3.845588	-0.4935	0.621663	NA
CG17490	0	NA	NA	NA	NA	NA
klhl10	0	NA	NA	NA	NA	NA
CG40006	51.96363	1.257247	3.951476	0.318171	0.750355	NA
CR40005	15.49635	-0.43713	3.840737	-0.11381	0.909385	NA
CG17636	0	NA	NA	NA	NA	NA
CG17707	51.43307	-3.12903	3.765235	-0.83103	0.405956	NA
CG17169	0	NA	NA	NA	NA	NA

CG17167	45.7948	-1.12672	3.86437	-0.29157	0.770618	NA
CG12061	0	NA	NA	NA	NA	NA
CR12460	0	NA	NA	NA	NA	NA
CG40198	0	NA	NA	NA	NA	NA
CG40298	0.1047	0.613261	4.062799	0.150945	0.880019	NA
Acp54A1	0	NA	NA	NA	NA	NA
Mkp	0.357625	-1.47707	4.044794	-0.36518	0.714979	NA
CG14042	0	NA	NA	NA	NA	NA
BG642163	0	NA	NA	NA	NA	NA
RhoU	13.2814	-2.73934	3.923108	-0.69826	0.485016	NA
CG34105	0	NA	NA	NA	NA	NA
CG34106	0	NA	NA	NA	NA	NA
CG34107	0.466186	0.959471	4.033986	0.237847	0.812	NA
CG34109	0.178053	-1.16467	4.075067	-0.2858	0.775028	NA
lobo	38.37439	-2.10141	3.953086	-0.53159	0.595012	NA
CG34115	7.852495	1.729066	3.957382	0.436922	0.662168	NA
CG34116	0	NA	NA	NA	NA	NA
CG34124	1.889787	-2.72002	3.946482	-0.68923	0.490682	NA
CG34129	0	NA	NA	NA	NA	NA
CG34130	0.209247	1.042804	4.047837	0.25762	0.7967	NA
Muted	0	NA	NA	NA	NA	NA
CG34135	0	NA	NA	NA	NA	NA
CG34138	17.27549	1.742783	3.953893	0.440776	0.659375	NA
CG34140	0	NA	NA	NA	NA	NA
CG17672	2.241339	-1.30622	3.964211	-0.3295	0.741775	NA
Ir10a	0	NA	NA	NA	NA	NA
RunxA	2.529472	-0.14319	3.897549	-0.03674	0.970693	NA
CG34148	9.949812	4.362198	3.829695	1.139046	0.254684	NA
CG34149	37.19161	-0.81193	3.849616	-0.21091	0.832956	NA
snRNA:U1:	13.6686	-4.28157	3.806563	-1.12479	0.26068	NA
snoRNA:M	22.10774	-3.75907	3.824501	-0.98289	0.325661	NA
snoRNA:M	3.245698	1.695863	3.966021	0.427598	0.668944	NA
stnA	0	NA	NA	NA	NA	NA
stnB	0	NA	NA	NA	NA	NA
CR40282	0	NA	NA	NA	NA	NA
CG13177	0	NA	NA	NA	NA	NA
asRNA:CR3	0	NA	NA	NA	NA	NA
CG41434	0	NA	NA	NA	NA	NA
CR41454	0	NA	NA	NA	NA	NA
lncRNA:CR	0	NA	NA	NA	NA	NA
FucTC	23.03865	-3.06613	3.829362	-0.80069	0.423311	NA
lncRNA:CR	0	NA	NA	NA	NA	NA
CR41440	33.32061	-2.84761	3.745428	-0.76029	0.447081	NA
lncRNA:CR	0.574571	1.337403	3.994282	0.334829	0.737754	NA
CG41106	0	NA	NA	NA	NA	NA
snoRNA:Ps	1.200397	-2.8489	3.970745	-0.71747	0.473083	NA
CR12798	0	NA	NA	NA	NA	NA
snoRNA:Ps	0	NA	NA	NA	NA	NA
snoRNA:Ps	3.475545	-4.83221	3.887653	-1.24296	0.213881	NA
Uhg7	0	NA	NA	NA	NA	NA

snoRNA:Oı	0	NA	NA	NA	NA	NA
snoRNA:Ps	25.17407	1.174623	3.952775	0.297164	0.766341	NA
snoRNA:Ps	0.715249	-1.88064	4.034336	-0.46616	0.641102	NA
snoRNA:Ps	0	NA	NA	NA	NA	NA
ATbp	11.85165	0.437701	3.763074	0.116315	0.907403	NA
snoRNA:Ps	0	NA	NA	NA	NA	NA
snoRNA:M	2.225846	2.347881	3.921221	0.598763	0.549331	NA
CG13051	0	NA	NA	NA	NA	NA
snoRNA:M	0	NA	NA	NA	NA	NA
snoRNA:Ps	0	NA	NA	NA	NA	NA
snoRNA:Ps	0	NA	NA	NA	NA	NA
snoRNA:Ps	0	NA	NA	NA	NA	NA
snoRNA:Ps	22.10084	2.963994	3.7823	0.783649	0.433246	NA
snoRNA:M	0	NA	NA	NA	NA	NA
snoRNA:Ps	5.529851	1.269576	3.872372	0.327855	0.743021	NA
snoRNA:Ps	0	NA	NA	NA	NA	NA
snoRNA:Ps	0	NA	NA	NA	NA	NA
CR40182	0	NA	NA	NA	NA	NA
snoRNA:M	0	NA	NA	NA	NA	NA
snoRNA:Ps	14.98477	-1.01657	3.776312	-0.2692	0.787779	NA
snoRNA:Ps	0	NA	NA	NA	NA	NA
snoRNA:Ps	0	NA	NA	NA	NA	NA
snoRNA:Ps	1.818025	-0.95856	3.935933	-0.24354	0.807587	NA
snoRNA:Ps	3.045501	1.793666	3.872031	0.463236	0.643195	NA
snoRNA:Ps	0	NA	NA	NA	NA	NA
snoRNA:M	2.63545	-3.25466	3.981159	-0.81752	0.413633	NA
kl-2	5.039264	1.402987	3.944899	0.355646	0.722106	NA
snoRNA:Ps	1.884599	1.659029	3.975915	0.41727	0.676481	NA
CG14742	0	NA	NA	NA	NA	NA
snoRNA:Ps	0.894061	-2.00006	4.030294	-0.49626	0.619714	NA
snoRNA:Ps	9.682531	-0.19292	3.844715	-0.05018	0.95998	NA
snoRNA:Ps	0	NA	NA	NA	NA	NA
snoRNA:M	4.81267	1.512878	3.958225	0.382211	0.702305	NA
snoRNA:M	0.715249	-1.88064	4.034336	-0.46616	0.641102	NA
snoRNA:Ps	1.674279	3.479487	3.968676	0.876737	0.380629	NA
snoRNA:M	5.667132	-4.69551	3.945678	-1.19004	0.234031	NA
snoRNA:M	0.2094	1.005034	4.045341	0.248442	0.803792	NA
ORY	0	NA	NA	NA	NA	NA
snoRNA:Ps	0.53416	-1.53481	4.012395	-0.38252	0.702078	NA
asRNA:CR3	22.25839	0.406871	3.837539	0.106024	0.915563	NA
snoRNA:Ps	0	NA	NA	NA	NA	NA
CR40461	8.525377	-0.95146	3.747525	-0.25389	0.79958	NA
snoRNA:Oı	16.6351	1.531556	3.953064	0.387435	0.698434	NA
snoRNA:Ps	0	NA	NA	NA	NA	NA
snoRNA:Ps	0.178053	-1.16467	4.075067	-0.2858	0.775028	NA
snoRNA:M	0.535678	-2.26616	4.021604	-0.5635	0.573098	NA
snoRNA:Ps	0.356107	-1.47099	4.037764	-0.36431	0.715628	NA
snoRNA:Ps	0.53416	-1.53481	4.012395	-0.38252	0.702078	NA
CR40068	0	NA	NA	NA	NA	NA
CG17159	0	NA	NA	NA	NA	NA

snoRNA:M	0	NA	NA	NA	NA	NA
snoRNA:Ps	0	NA	NA	NA	NA	NA
snoRNA:Ps	0	NA	NA	NA	NA	NA
snoRNA:M	3.062653	-4.16238	3.889479	-1.07016	0.284545	NA
snoRNA:Ps	0.2094	1.005034	4.045341	0.248442	0.803792	NA
snoRNA:Ps	0	NA	NA	NA	NA	NA
snoRNA:M	1.646575	-1.83931	3.938458	-0.46701	0.640491	NA
snoRNA:Ps	0	NA	NA	NA	NA	NA
ARY	0	NA	NA	NA	NA	NA
CG14397	22.25356	0.643842	3.920878	0.164209	0.869567	NA
snoRNA:Ps	5.875758	-1.68859	3.957348	-0.4267	0.6696	NA
snoRNA:Ps	0	NA	NA	NA	NA	NA
snoRNA:Ps	0	NA	NA	NA	NA	NA
snoRNA:Ps	0	NA	NA	NA	NA	NA
snoRNA:Ps	0	NA	NA	NA	NA	NA
snoRNA:Ps	13.00586	3.176553	3.803685	0.835125	0.403647	NA
snoRNA:Ps	0	NA	NA	NA	NA	NA
snoRNA:Ps	7.510116	-2.23383	3.963189	-0.56365	0.572996	NA
snoRNA:Or	6.877103	-1.01854	3.952131	-0.25772	0.796624	NA
snoRNA:Ps	0	NA	NA	NA	NA	NA
CR14578	0	NA	NA	NA	NA	NA
snoRNA:Ps	5.184038	-4.10985	3.943772	-1.04211	0.29736	NA
snoRNA:Ps	0	NA	NA	NA	NA	NA
snoRNA:Ps	43.30106	1.145615	3.951975	0.289884	0.771905	NA
snoRNA:M	2.976774	-2.12883	3.8972	-0.54625	0.584897	NA
snoRNA:Ps	38.22798	-0.70451	3.835459	-0.18368	0.854261	NA
snoRNA:M	2.082464	-1.43897	3.885873	-0.37031	0.711153	NA
snoRNA:M	0.357625	-1.47707	4.044794	-0.36518	0.714979	NA
snoRNA:Ps	0	NA	NA	NA	NA	NA
snoRNA:M	0.1047	0.613261	4.062799	0.150945	0.880019	NA
snoRNA:Ps	0	NA	NA	NA	NA	NA
snoRNA:Ps	11.08636	-2.24874	3.959363	-0.56796	0.570065	NA
CG11741	0	NA	NA	NA	NA	NA
snoRNA:Ps	1.984084	-2.49864	3.931494	-0.63554	0.525074	NA
snoRNA:Ps	0	NA	NA	NA	NA	NA
snoRNA:Ps	0	NA	NA	NA	NA	NA
snoRNA:M	0.233093	0.692656	4.076246	0.169925	0.865069	NA
snoRNA:Ps	0	NA	NA	NA	NA	NA
snoRNA:Or	0	NA	NA	NA	NA	NA
snoRNA:Ps	0	NA	NA	NA	NA	NA
snoRNA:Ps	17.6203	-0.11337	3.735348	-0.03035	0.975788	NA
snoRNA:M	8.605654	-1.26164	3.766497	-0.33496	0.737653	NA
snoRNA:Ps	0.357625	-1.47707	4.044794	-0.36518	0.714979	NA
Pp1-Y2	0	NA	NA	NA	NA	NA
CG7606	0.1047	0.613261	4.062799	0.150945	0.880019	NA
asRNA:CR3	0	NA	NA	NA	NA	NA
CR40441	0	NA	NA	NA	NA	NA
CG16824	0	NA	NA	NA	NA	NA
snoRNA:Ps	0.178053	-1.16467	4.075067	-0.2858	0.775028	NA
snoRNA:Ps	0	NA	NA	NA	NA	NA

snoRNA:Ps	0.178812	-1.08808	4.061735	-0.26789	0.788788	NA
snoRNA:Ps	0	NA	NA	NA	NA	NA
snoRNA:M	0	NA	NA	NA	NA	NA
snoRNA:Ps	0	NA	NA	NA	NA	NA
CG12828	0	NA	NA	NA	NA	NA
snoRNA:M	0	NA	NA	NA	NA	NA
snoRNA:Ps	0.1047	0.613261	4.062799	0.150945	0.880019	NA
snoRNA:M	0.3141	1.240997	4.03841	0.307299	0.758616	NA
snoRNA:Ps	0	NA	NA	NA	NA	NA
snoRNA:Ps	0	NA	NA	NA	NA	NA
snoRNA:Ps	0	NA	NA	NA	NA	NA
snoRNA:Ps	0.535678	-2.26616	4.021604	-0.5635	0.573098	NA
snoRNA:Ps	0	NA	NA	NA	NA	NA
CG15140	0	NA	NA	NA	NA	NA
Ppr-Y	0	NA	NA	NA	NA	NA
snoRNA:M	1.165466	1.076041	3.987865	0.269829	0.787292	NA
snoRNA:M	4.649119	-2.2071	3.970175	-0.55592	0.578266	NA
snoRNA:M	2.106559	-2.51934	3.952378	-0.63742	0.523849	NA
snoRNA:Or	1.251686	-2.06022	4.011654	-0.51356	0.60756	NA
snoRNA:M	1.570499	1.642769	3.980387	0.412716	0.679815	NA
snoRNA:Ps	0	NA	NA	NA	NA	NA
snoRNA:Ps	0.715249	-1.88064	4.034336	-0.46616	0.641102	NA
lncRNA:Yu	0	NA	NA	NA	NA	NA
snoRNA:Ps	0	NA	NA	NA	NA	NA
snoRNA:M	0	NA	NA	NA	NA	NA
snoRNA:Ps	0	NA	NA	NA	NA	NA
snoRNA:Ps	0	NA	NA	NA	NA	NA
snoRNA:Ps	0	NA	NA	NA	NA	NA
snoRNA:Ps	0.178053	-1.16467	4.075067	-0.2858	0.775028	NA
snoRNA:Ps	0.132078	-1.13211	4.069918	-0.27817	0.780885	NA
snoRNA:Ps	0.396233	-1.6814	4.028351	-0.41739	0.676392	NA
snoRNA:Ps	0.178053	-1.16467	4.075067	-0.2858	0.775028	NA
snoRNA:Ps	7.848331	2.115625	3.936653	0.537417	0.59098	NA
CR40100	7.339494	-6.11453	3.91646	-1.56124	0.118468	NA
snoRNA:Ps	3.218621	-2.17835	3.977872	-0.54762	0.583954	NA
snoRNA:Ps	0	NA	NA	NA	NA	NA
snoRNA:Ps	13.34042	0.040251	3.904549	0.010309	0.991775	NA
snoRNA:Ps	0.2094	1.005034	4.045341	0.248442	0.803792	NA
CG34161	37.39119	-1.70511	3.951934	-0.43146	0.666132	NA
CG34162	2.198698	1.671135	3.972627	0.420662	0.674002	NA
CG34163	11.51699	1.736994	3.955359	0.43915	0.660553	NA
CG34164	0	NA	NA	NA	NA	NA
CG34165	0	NA	NA	NA	NA	NA
CG34166	0	NA	NA	NA	NA	NA
CG34167	21.05015	5.179901	3.853643	1.344157	0.178898	NA
CG34168	0	NA	NA	NA	NA	NA
CG34169	0.132078	-1.13211	4.069918	-0.27817	0.780885	NA
CG34170	0	NA	NA	NA	NA	NA
CG34171	0	NA	NA	NA	NA	NA
CG34173	0	NA	NA	NA	NA	NA

CG34174	0	NA	NA	NA	NA	NA
CG34175	0	NA	NA	NA	NA	NA
CG34176	0	NA	NA	NA	NA	NA
CG34177	0	NA	NA	NA	NA	NA
CG34178	0	NA	NA	NA	NA	NA
CG34179	0	NA	NA	NA	NA	NA
CG34181	0	NA	NA	NA	NA	NA
RpS28-like	0	NA	NA	NA	NA	NA
CG34184	0	NA	NA	NA	NA	NA
CG34185	0	NA	NA	NA	NA	NA
CG34190	0	NA	NA	NA	NA	NA
CG34193	0	NA	NA	NA	NA	NA
CG34194	0	NA	NA	NA	NA	NA
CG34196	0	NA	NA	NA	NA	NA
CG34198	0	NA	NA	NA	NA	NA
CG34199	0	NA	NA	NA	NA	NA
CG34201	16.29153	-3.49339	3.904011	-0.89482	0.370883	NA
CG34202	0.565506	-0.26224	3.986711	-0.06578	0.947554	NA
CG34203	0.53416	-1.53481	4.012395	-0.38252	0.702078	NA
CG34204	0	NA	NA	NA	NA	NA
CG34206	26.07028	1.746756	3.952894	0.441893	0.658567	NA
CG34207	0	NA	NA	NA	NA	NA
CG34208	7.132195	-1.90434	3.956298	-0.48134	0.630272	NA
CG34210	10.1923	-2.24595	3.960077	-0.56715	0.570614	NA
CG34211	0	NA	NA	NA	NA	NA
CG34212	0	NA	NA	NA	NA	NA
CG34214	0	NA	NA	NA	NA	NA
CG34215	33.47263	1.690341	3.952388	0.427676	0.668887	NA
CG34216	0	NA	NA	NA	NA	NA
CG34217	0	NA	NA	NA	NA	NA
CG34221	0.1047	0.613261	4.062799	0.150945	0.880019	NA
CG34223	0.104623	0.639075	4.067674	0.157111	0.875158	NA
CG34224	0	NA	NA	NA	NA	NA
CG34225	0	NA	NA	NA	NA	NA
CG34227	5.653796	1.719633	3.959809	0.434272	0.664091	NA
CG34230	0	NA	NA	NA	NA	NA
CG34234	0	NA	NA	NA	NA	NA
CG34235	0	NA	NA	NA	NA	NA
CG34236	0	NA	NA	NA	NA	NA
CG34238	0	NA	NA	NA	NA	NA
CG34244	0	NA	NA	NA	NA	NA
CG34245	0	NA	NA	NA	NA	NA
CG34247	0	NA	NA	NA	NA	NA
CG34248	0.178053	-1.16467	4.075067	-0.2858	0.775028	NA
CG34251	18.06871	1.610685	3.900631	0.412929	0.679658	NA
CG34253	0	NA	NA	NA	NA	NA
CR34254	0	NA	NA	NA	NA	NA
Blos3	57.43077	-1.06601	3.121798	-0.34147	0.732746	NA
CG34256	46.84882	-2.27414	3.952963	-0.5753	0.565088	NA
CG34258	1.37921	-3.74935	3.951609	-0.94882	0.342714	NA

CG34260	32.95633	1.535458	3.952	0.388527	0.697626	NA
CG34261	30.66345	0.42979	3.835644	0.112052	0.910783	NA
CR34262	0	NA	NA	NA	NA	NA
CG34263	0	NA	NA	NA	NA	NA
CG34265	0	NA	NA	NA	NA	NA
CG34267	0	NA	NA	NA	NA	NA
CG34268	0	NA	NA	NA	NA	NA
CG34269	0	NA	NA	NA	NA	NA
Cpr65Ay	0	NA	NA	NA	NA	NA
CG34273	0	NA	NA	NA	NA	NA
CG34274	0.104623	0.639075	4.067674	0.157111	0.875158	NA
CG34276	0	NA	NA	NA	NA	NA
CG34277	0	NA	NA	NA	NA	NA
CG34278	0	NA	NA	NA	NA	NA
CG34279	5.679341	-1.8997	3.957646	-0.48001	0.631222	NA
CG34280	0	NA	NA	NA	NA	NA
CG34281	0	NA	NA	NA	NA	NA
CG34282	0.178053	-1.16467	4.075067	-0.2858	0.775028	NA
CG34283	0	NA	NA	NA	NA	NA
CG34284	0	NA	NA	NA	NA	NA
CR34285	0	NA	NA	NA	NA	NA
CG34286	0	NA	NA	NA	NA	NA
CG34287	0	NA	NA	NA	NA	NA
CG34290	0.178053	-1.16467	4.075067	-0.2858	0.775028	NA
CG34291	0	NA	NA	NA	NA	NA
CG34292	0.282753	-0.25398	4.020204	-0.06318	0.949626	NA
CG34295	0	NA	NA	NA	NA	NA
CG34296	0	NA	NA	NA	NA	NA
CG34297	1.546449	-0.90035	3.878965	-0.23211	0.816451	NA
CG34298	0	NA	NA	NA	NA	NA
CG34299	1.424426	-1.63334	3.97617	-0.41078	0.681232	NA
CG34300	26.81957	-4.85751	3.890047	-1.2487	0.211774	NA
CG34301	0	NA	NA	NA	NA	NA
CG34302	0	NA	NA	NA	NA	NA
CG34305	0	NA	NA	NA	NA	NA
CG34307	0	NA	NA	NA	NA	NA
CG34308	0	NA	NA	NA	NA	NA
CR34311	0	NA	NA	NA	NA	NA
CG34312	0	NA	NA	NA	NA	NA
CG34313	0	NA	NA	NA	NA	NA
Sws1	0	NA	NA	NA	NA	NA
CG34315	0	NA	NA	NA	NA	NA
CG34316	0	NA	NA	NA	NA	NA
Rpp14a	0	NA	NA	NA	NA	NA
CR34320	0	NA	NA	NA	NA	NA
inaF-C	0	NA	NA	NA	NA	NA
inaF-A	0.178053	-1.16467	4.075067	-0.2858	0.775028	NA
CG34323	0	NA	NA	NA	NA	NA
CG34324	4.869825	0.168899	3.875984	0.043576	0.965242	NA
CG34327	0	NA	NA	NA	NA	NA

CG34328	0	NA	NA	NA	NA	NA
Diedel3	0	NA	NA	NA	NA	NA
CG34330	0	NA	NA	NA	NA	NA
CG34331	0.356866	-1.92326	4.037889	-0.4763	0.633858	NA
CG34332	0	NA	NA	NA	NA	NA
Vml	0	NA	NA	NA	NA	NA
CG34337	32.07801	-0.78824	3.836045	-0.20548	0.837196	NA
CG34338	0	NA	NA	NA	NA	NA
CG34342	0.233093	0.692656	4.076246	0.169925	0.865069	NA
CG34345	0.178053	-1.16467	4.075067	-0.2858	0.775028	NA
ppk9	0	NA	NA	NA	NA	NA
CG34375	0	NA	NA	NA	NA	NA
CG34382	0.1047	0.613261	4.062799	0.150945	0.880019	NA
CG34386	0	NA	NA	NA	NA	NA
natalisin	0	NA	NA	NA	NA	NA
CG34398	6.650767	-2.25569	3.816227	-0.59108	0.554468	NA
Nox	17.90369	1.743194	3.95379	0.440892	0.659291	NA
CG34402	6.15927	-2.57152	3.928673	-0.65455	0.512757	NA
Lgr4	38.88874	1.61589	3.899551	0.414378	0.678597	NA
Mthfs	0	NA	NA	NA	NA	NA
CG34425	0	NA	NA	NA	NA	NA
CG34426	0.178053	-1.16467	4.075067	-0.2858	0.775028	NA
CG34427	0	NA	NA	NA	NA	NA
CG34428	0	NA	NA	NA	NA	NA
CG34429	0	NA	NA	NA	NA	NA
CG34431	4.137111	0.483953	3.852062	0.125635	0.900021	NA
CG34432	0	NA	NA	NA	NA	NA
CG34433	0	NA	NA	NA	NA	NA
CG34435	0	NA	NA	NA	NA	NA
CG34436	0.712213	-1.57119	3.998613	-0.39293	0.694369	NA
CG34437	2.303398	1.674512	3.971716	0.421609	0.67331	NA
ImgB	0	NA	NA	NA	NA	NA
CG34442	0	NA	NA	NA	NA	NA
CG34443	0	NA	NA	NA	NA	NA
CG34444	0	NA	NA	NA	NA	NA
CG34445	0	NA	NA	NA	NA	NA
CG34446	0	NA	NA	NA	NA	NA
CG34447	0	NA	NA	NA	NA	NA
CG34448	0	NA	NA	NA	NA	NA
CG34450	0	NA	NA	NA	NA	NA
CG34451	0	NA	NA	NA	NA	NA
CG34452	0	NA	NA	NA	NA	NA
CG34453	54.05472	0.127405	3.9513	0.032244	0.974278	NA
CG34456	0	NA	NA	NA	NA	NA
CG34458	0	NA	NA	NA	NA	NA
CG34459	0.356107	-1.47099	4.037764	-0.36431	0.715628	NA
CG34460	0	NA	NA	NA	NA	NA
CG34462	0.178053	-1.16467	4.075067	-0.2858	0.775028	NA
Mst77Y-16	6.207651	-1.90163	3.957084	-0.48056	0.630826	NA
CR40601	0	NA	NA	NA	NA	NA

lncRNA:CR	0	NA	NA	NA	NA	NA
CG40635	0.5235	1.490714	4.02555	0.370313	0.711149	NA
lncRNA:CR	0	NA	NA	NA	NA	NA
RYa	13.40159	1.739423	3.954743	0.439832	0.660059	NA
CR40801	0.792466	-1.7823	3.993845	-0.44626	0.655408	NA
CG40813	3.871061	1.506839	3.959948	0.38052	0.70356	NA
Su(Ste):CR	0.132078	-1.13211	4.069918	-0.27817	0.780885	NA
lncRNA:CR	0	NA	NA	NA	NA	NA
CR41243	0.31387	1.261782	4.037145	0.312543	0.754628	NA
CR41501	49.29806	2.057472	3.81542	0.539252	0.589713	NA
CG41242	0	NA	NA	NA	NA	NA
lncRNA:CR	0	NA	NA	NA	NA	NA
CR41519	0.104623	0.639075	4.067674	0.157111	0.875158	NA
CR41597	0	NA	NA	NA	NA	NA
CR41320	20.01437	1.693361	3.737582	0.453063	0.650503	NA
CG41284	0	NA	NA	NA	NA	NA
lncRNA:CR	0	NA	NA	NA	NA	NA
CR41423	0.178812	-1.08808	4.061735	-0.26789	0.788788	NA
28SrRNA-F	41.52785	-0.11744	3.506814	-0.03349	0.973284	NA
CG41562	0.732363	1.393579	3.993417	0.348969	0.727112	NA
18SrRNA:C	0	NA	NA	NA	NA	NA
Su(Ste):CR	0.2094	1.005034	4.045341	0.248442	0.803792	NA
CR41571	0	NA	NA	NA	NA	NA
CG41561	0	NA	NA	NA	NA	NA
CR40450	44.02617	-4.9226	3.860887	-1.27499	0.202313	NA
CR41379	1.827286	3.319894	3.963865	0.83754	0.402289	NA
2SrRNA-Ps	0.1047	0.613261	4.062799	0.150945	0.880019	NA
CR41506	3.574231	0.322748	3.853103	0.083763	0.933245	NA
CR41504	0	NA	NA	NA	NA	NA
CR41509	0	NA	NA	NA	NA	NA
CR41508	0	NA	NA	NA	NA	NA
CR41507	1.234675	-3.13913	3.971472	-0.79042	0.429282	NA
CR42201	0.313946	1.788224	4.01364	0.445537	0.655932	NA
snoRNA:M	0	NA	NA	NA	NA	NA
snoRNA:M	0	NA	NA	NA	NA	NA
snoRNA:M	1.164483	1.950196	3.904832	0.499432	0.617475	NA
snoRNA:M	1.464879	3.015441	3.967965	0.759947	0.447287	NA
snoRNA:M	0	NA	NA	NA	NA	NA
snoRNA:M	0	NA	NA	NA	NA	NA
snoRNA:Or	2.825288	4.097154	3.94606	1.03829	0.299135	NA
snoRNA:M	0	NA	NA	NA	NA	NA
snoRNA:M	0	NA	NA	NA	NA	NA
snoRNA:M	0	NA	NA	NA	NA	NA
snoRNA:M	0	NA	NA	NA	NA	NA
snoRNA:M	0	NA	NA	NA	NA	NA
snoRNA:M	0	NA	NA	NA	NA	NA
snoRNA:M	0	NA	NA	NA	NA	NA
snoRNA:M	0	NA	NA	NA	NA	NA
snoRNA:M	0.1047	0.613261	4.062799	0.150945	0.880019	NA
snoRNA:M	0	NA	NA	NA	NA	NA

snoRNA:M	0.283512	-0.2331	4.065094	-0.05734	0.954273	NA
snoRNA:M	0	NA	NA	NA	NA	NA
snoRNA:M	0	NA	NA	NA	NA	NA
snoRNA:M	0	NA	NA	NA	NA	NA
snoRNA:M	0	NA	NA	NA	NA	NA
snoRNA:M	0	NA	NA	NA	NA	NA
snoRNA:M	0	NA	NA	NA	NA	NA
snoRNA:M	4.927818	-5.39244	3.922516	-1.37474	0.169212	NA
snoRNA:M	0	NA	NA	NA	NA	NA
snoRNA:M	0	NA	NA	NA	NA	NA
snoRNA:M	1.094576	-2.45718	3.919279	-0.62695	0.530693	NA
snoRNA:M	0	NA	NA	NA	NA	NA
snoRNA:M	0	NA	NA	NA	NA	NA
snoRNA:M	0	NA	NA	NA	NA	NA
snoRNA:M	0.1047	0.613261	4.062799	0.150945	0.880019	NA
snoRNA:M	0	NA	NA	NA	NA	NA
snoRNA:M	0	NA	NA	NA	NA	NA
snoRNA:M	0.984682	0.033526	3.923473	0.008545	0.993182	NA
snoRNA:M	0	NA	NA	NA	NA	NA
snoRNA:M	0	NA	NA	NA	NA	NA
snoRNA:O	0	NA	NA	NA	NA	NA
snoRNA:M	6.288439	0.458237	3.86805	0.118467	0.905697	NA
snoRNA:M	0.178812	-1.08808	4.061735	-0.26789	0.788788	NA
snoRNA:M	0	NA	NA	NA	NA	NA
snoRNA:M	0	NA	NA	NA	NA	NA
snoRNA:M	0	NA	NA	NA	NA	NA
snoRNA:M	0.670206	-0.05147	3.98115	-0.01293	0.989686	NA
snoRNA:O	9.810748	-1.02007	3.863705	-0.26401	0.79177	NA
snoRNA:M	0	NA	NA	NA	NA	NA
snoRNA:O	8.239336	-2.29073	3.842116	-0.59622	0.551031	NA
snoRNA:O	0	NA	NA	NA	NA	NA
snoRNA:O	0	NA	NA	NA	NA	NA
snoRNA:M	0	NA	NA	NA	NA	NA
snoRNA:M	7.239783	0.346644	3.953483	0.087681	0.930131	NA
snoRNA:M	0	NA	NA	NA	NA	NA
snoRNA:M	1.251686	-2.06022	4.011654	-0.51356	0.60756	NA
snoRNA:M	0	NA	NA	NA	NA	NA
snoRNA:M	0.461565	-1.07936	4.002266	-0.26969	0.7874	NA
snoRNA:M	0	NA	NA	NA	NA	NA
snoRNA:M	0.178053	-1.16467	4.075067	-0.2858	0.775028	NA
snoRNA:O	0	NA	NA	NA	NA	NA
CR42490	0.178053	-1.16467	4.075067	-0.2858	0.775028	NA
psd	0	NA	NA	NA	NA	NA
Vm26Ac	0	NA	NA	NA	NA	NA
se	0	NA	NA	NA	NA	NA
tum	0	NA	NA	NA	NA	NA
t	0.442209	-2.31142	4.024999	-0.57427	0.565788	NA
tw	0	NA	NA	NA	NA	NA
stl	1.194388	0.722286	3.956144	0.182573	0.855133	NA
Cpr47Eg	0	NA	NA	NA	NA	NA

Spt-I	0	NA	NA	NA	NA	NA
snoRNA:M	0.264155	-1.54099	4.048976	-0.38059	0.703509	NA
snoRNA:Ps	0	NA	NA	NA	NA	NA
snoRNA:Ps	0	NA	NA	NA	NA	NA
snoRNA:Ps	35.25683	-3.98371	3.889472	-1.02423	0.305727	NA
snoRNA:Or	0	NA	NA	NA	NA	NA
CG34112	23.98521	-6.43086	3.846532	-1.67186	0.094552	NA
CG33342	0	NA	NA	NA	NA	NA
Lcp65Ag3	0	NA	NA	NA	NA	NA
snoRNA:Ps	0.1047	0.613261	4.062799	0.150945	0.880019	NA
snoRNA:Ps	0	NA	NA	NA	NA	NA
snoRNA:Ps	0	NA	NA	NA	NA	NA
snoRNA:Ps	21.38994	0.049084	3.857496	0.012724	0.989848	NA
snoRNA:Ps	7.688928	-2.23489	3.962917	-0.56395	0.572788	NA
snoRNA:Ps	27.5026	-7.47493	3.912742	-1.91041	0.056081	NA
snoRNA:Ps	6.464319	-0.30451	3.847808	-0.07914	0.936923	NA
snoRNA:Ps	6.766025	-1.69111	3.956514	-0.42742	0.66907	NA
snoRNA:Ps	28.75449	0.361046	3.731775	0.096749	0.922926	NA
snoRNA:Ps	4.807438	-1.68437	3.95875	-0.42548	0.670487	NA
snoRNA:Ps	0	NA	NA	NA	NA	NA
snoRNA:Ps	17.44021	-0.04625	3.740045	-0.01237	0.990133	NA
snoRNA:Ps	0.565506	-0.26224	3.986711	-0.06578	0.947554	NA
snoRNA:Or	10.2345	0.309871	3.763927	0.082327	0.934387	NA
CG13272	10.71288	-1.30008	3.811294	-0.34111	0.733018	NA
iav	0	NA	NA	NA	NA	NA
hd	7.852418	2.924533	3.930606	0.744041	0.456852	NA
frtz	38.13939	-1.02958	3.920492	-0.26261	0.792847	NA
dgo	0.178053	-1.16467	4.075067	-0.2858	0.775028	NA
Cpr65Ax1	0	NA	NA	NA	NA	NA
Cyt-c-d	31.27061	0.344623	3.839481	0.089758	0.92848	NA
mbm	42.01534	-2.0615	3.932109	-0.52427	0.600088	NA
snky	0	NA	NA	NA	NA	NA
rtp	1.256399	1.619735	3.986836	0.406271	0.684544	NA
alphaTub6	23.09031	3.408532	3.747986	0.90943	0.363123	NA
Hyls1	0	NA	NA	NA	NA	NA
eater	0	NA	NA	NA	NA	NA
Tb	0	NA	NA	NA	NA	NA
5.8SrRNA:(	0	NA	NA	NA	NA	NA
Jon65Aiv	0	NA	NA	NA	NA	NA
lncRNA:CR	0	NA	NA	NA	NA	NA
CG34222	3.43402	-1.88523	3.961894	-0.47584	0.634189	NA
CG33655	0.942299	1.584447	3.996979	0.396411	0.691802	NA
CG34241	0	NA	NA	NA	NA	NA
CG34160	53.41598	-1.70607	3.951626	-0.43174	0.665932	NA
BG642167	0	NA	NA	NA	NA	NA
Dup99B	0	NA	NA	NA	NA	NA
CG34461	0.233093	0.692656	4.076246	0.169925	0.865069	NA
CG33308	0	NA	NA	NA	NA	NA
Acbp3	0.178053	-1.16467	4.075067	-0.2858	0.775028	NA
dUTPase	0	NA	NA	NA	NA	NA

CG17242	0.178053	-1.16467	4.075067	-0.2858	0.775028	NA
CG17575	0.233093	0.692656	4.076246	0.169925	0.865069	NA
CG1288	0.178053	-1.16467	4.075067	-0.2858	0.775028	NA
glob2	0.132078	-1.13211	4.069918	-0.27817	0.780885	NA
CG14034	4.807438	-1.68437	3.95875	-0.42548	0.670487	NA
CG32388	0	NA	NA	NA	NA	NA
CG33981	0	NA	NA	NA	NA	NA
CG42237	3.978521	2.873751	3.942721	0.728875	0.466078	NA
pot	0.51577	1.033715	4.015187	0.257451	0.79683	NA
lmgA	0	NA	NA	NA	NA	NA
CG34434	0.462248	-0.67622	4.039367	-0.16741	0.867049	NA
Cht10	17.03506	1.591822	3.842586	0.414258	0.678685	NA
CG42245	0	NA	NA	NA	NA	NA
CG42246	3.830253	-1.88895	3.960794	-0.47691	0.633424	NA
DCX-EMAF	60.6503	0.647423	3.920179	0.165152	0.868825	NA
CG42249	6.822581	-2.42613	3.785184	-0.64095	0.521552	NA
PolA1	0.837599	1.568055	4.001801	0.391837	0.695178	NA
CG42255	34.3461	1.157374	3.873966	0.298757	0.765125	NA
CG42264	0	NA	NA	NA	NA	NA
CG42266	2.272811	0.29017	3.960962	0.073257	0.941601	NA
RunxB	0	NA	NA	NA	NA	NA
CG42269	0.466186	0.959471	4.033986	0.237847	0.812	NA
CG42272	8.899494	1.731975	3.956638	0.437739	0.661575	NA
CG42284	0	NA	NA	NA	NA	NA
CG42287	2.012992	2.885432	3.965929	0.727555	0.466886	NA
CG42288	28.88843	-3.09444	3.800109	-0.8143	0.415471	NA
Ir60b	0	NA	NA	NA	NA	NA
Ir60c	0	NA	NA	NA	NA	NA
Ir60d	0	NA	NA	NA	NA	NA
Ir7f	0	NA	NA	NA	NA	NA
Ir7e	0	NA	NA	NA	NA	NA
Ir7d	0	NA	NA	NA	NA	NA
CG42296	0	NA	NA	NA	NA	NA
Ir94d	0	NA	NA	NA	NA	NA
Ir94e	0	NA	NA	NA	NA	NA
CG42299	0	NA	NA	NA	NA	NA
CG42300	0	NA	NA	NA	NA	NA
CG42302	0	NA	NA	NA	NA	NA
Snup	0	NA	NA	NA	NA	NA
CG42304	0	NA	NA	NA	NA	NA
CG42305	0	NA	NA	NA	NA	NA
CG42306	0	NA	NA	NA	NA	NA
CG42307	0.356107	-1.47099	4.037764	-0.36431	0.715628	NA
CG42308	0	NA	NA	NA	NA	NA
Mlp60A	0	NA	NA	NA	NA	NA
prom	25.67408	4.169242	3.806226	1.095374	0.273353	NA
grh	17.05361	-2.42819	3.860112	-0.62905	0.529319	NA
CG42319	0.5235	1.490714	4.02555	0.370313	0.711149	NA
CG42323	0	NA	NA	NA	NA	NA
CG42329	0	NA	NA	NA	NA	NA

lectin-22C	0	NA	NA	NA	NA	NA
CCKLR-17D	49.48404	0.296766	3.834589	0.077392	0.938312	NA
comm3	0	NA	NA	NA	NA	NA
CG42335	1.360248	-0.69345	3.975274	-0.17444	0.861519	NA
Prim2	1.780533	-1.64713	3.971374	-0.41475	0.678325	NA
sqa	1.128562	-2.72595	3.934459	-0.69284	0.488411	NA
Jabba	2.565465	0.351309	3.959571	0.088724	0.929301	NA
Ir40a	0	NA	NA	NA	NA	NA
CG42353	0	NA	NA	NA	NA	NA
CG42355	0	NA	NA	NA	NA	NA
CG42356	0	NA	NA	NA	NA	NA
CG42357	0	NA	NA	NA	NA	NA
CG42362	0	NA	NA	NA	NA	NA
CG42363	0	NA	NA	NA	NA	NA
CG42364	0	NA	NA	NA	NA	NA
CG42366	0	NA	NA	NA	NA	NA
CG42367	0.1047	0.613261	4.062799	0.150945	0.880019	NA
CG42369	0	NA	NA	NA	NA	NA
Nepl6	42.55742	-0.32424	3.835188	-0.08454	0.932624	NA
CG42371	0	NA	NA	NA	NA	NA
CG42372	0	NA	NA	NA	NA	NA
CG42374	0	NA	NA	NA	NA	NA
CG42375	0	NA	NA	NA	NA	NA
CG42379	0	NA	NA	NA	NA	NA
CG42380	0	NA	NA	NA	NA	NA
CG42381	0	NA	NA	NA	NA	NA
CG42382	0.178053	-1.16467	4.075067	-0.2858	0.775028	NA
CG42383	0.2094	1.005034	4.045341	0.248442	0.803792	NA
tal-1A	0	NA	NA	NA	NA	NA
tal-2A	0	NA	NA	NA	NA	NA
tal-3A	0	NA	NA	NA	NA	NA
tal-AA	0	NA	NA	NA	NA	NA
CG42395	0	NA	NA	NA	NA	NA
CG42397	0	NA	NA	NA	NA	NA
bora	0	NA	NA	NA	NA	NA
sinah	0	NA	NA	NA	NA	NA
loopin-1	0	NA	NA	NA	NA	NA
CG34309	0	NA	NA	NA	NA	NA
out	16.97128	-3.24688	3.868596	-0.83929	0.401306	NA
Su(Ste):CR	0.264155	-1.54099	4.048976	-0.38059	0.703509	NA
Su(Ste):CR	8.415129	-1.05988	3.953582	-0.26808	0.788637	NA
Su(Ste):CR	1.662101	-1.09108	3.964539	-0.27521	0.783155	NA
Su(Ste):CR	0.178053	-1.16467	4.075067	-0.2858	0.775028	NA
Su(Ste):CR	0.776424	-0.14961	3.950397	-0.03787	0.969789	NA
Su(Ste):CR	0	NA	NA	NA	NA	NA
Su(Ste):CR	8.14869	-4.2577	3.893911	-1.09343	0.274207	NA
Su(Ste):CR	0.132078	-1.13211	4.069918	-0.27817	0.780885	NA
Su(Ste):CR	1.972325	3.162291	3.909253	0.808925	0.418558	NA
Su(Ste):CR	0.178812	-1.08808	4.061735	-0.26789	0.788788	NA
Su(Ste):CR	0	NA	NA	NA	NA	NA

Su(Ste):CR	1.642779	0.571925	3.935522	0.145324	0.884455	NA
Su(Ste):CR	0	NA	NA	NA	NA	NA
Su(Ste):CR	0	NA	NA	NA	NA	NA
Su(Ste):CR	0.462324	-0.59023	4.044233	-0.14594	0.883966	NA
Su(Ste):CR	2.290454	0.258093	3.867943	0.066726	0.9468	NA
Su(Ste):CR	0	NA	NA	NA	NA	NA
Su(Ste):CR	1.560473	-0.69219	3.96537	-0.17456	0.861426	NA
Su(Ste):CR	1.267059	0.18295	3.967592	0.046111	0.963222	NA
Su(Ste):CR	0	NA	NA	NA	NA	NA
Su(Ste):CR	0	NA	NA	NA	NA	NA
Su(Ste):CR	0	NA	NA	NA	NA	NA
Su(Ste):CR	12.31828	-1.26897	3.885309	-0.32661	0.743965	NA
Su(Ste):CR	0	NA	NA	NA	NA	NA
Su(Ste):CR	0.46073	-0.17853	4.00793	-0.04454	0.964471	NA
Su(Ste):CR	19.16026	-0.53338	3.759553	-0.14187	0.887179	NA
Su(Ste):CR	0.1047	0.613261	4.062799	0.150945	0.880019	NA
Su(Ste):CR	7.964415	0.86224	3.864498	0.223118	0.823444	NA
Su(Ste):CR	0.283512	-0.2331	4.065094	-0.05734	0.954273	NA
NimC1	8.724611	-1.69489	3.95527	-0.42851	0.668277	NA
CG42446	0.132078	-1.13211	4.069918	-0.27817	0.780885	NA
inaF-B	0.178053	-1.16467	4.075067	-0.2858	0.775028	NA
Skadu	0	NA	NA	NA	NA	NA
CG42449	0.1047	0.613261	4.062799	0.150945	0.880019	NA
CG42455	0	NA	NA	NA	NA	NA
Dpm2	0	NA	NA	NA	NA	NA
Sfp23F	0	NA	NA	NA	NA	NA
CG42460	0	NA	NA	NA	NA	NA
Sfp24Ba	15.28619	1.741265	3.954277	0.44035	0.659684	NA
Sfp24Bb	11.44399	-2.24975	3.959108	-0.56825	0.569868	NA
Sfp24Bd	8.061894	1.729706	3.957218	0.437101	0.662038	NA
CG42464	0	NA	NA	NA	NA	NA
CG42465	0	NA	NA	NA	NA	NA
Sfp24C1	0	NA	NA	NA	NA	NA
CG42467	0.178053	-1.16467	4.075067	-0.2858	0.775028	NA
Sfp24F	0	NA	NA	NA	NA	NA
Sfp26Ac	0	NA	NA	NA	NA	NA
CG42470	0.31387	1.261782	4.037145	0.312543	0.754628	NA
CG42471	0	NA	NA	NA	NA	NA
Sfp33A1	0	NA	NA	NA	NA	NA
Sfp33A2	4.711497	1.713057	3.961514	0.432425	0.665433	NA
Sfp33A3	0	NA	NA	NA	NA	NA
Sfp35C	0	NA	NA	NA	NA	NA
Sfp51E	0	NA	NA	NA	NA	NA
Sfp53D	0	NA	NA	NA	NA	NA
Sfp60F	0.104623	0.639075	4.067674	0.157111	0.875158	NA
Sfp65A	0	NA	NA	NA	NA	NA
Sfp70A4	0	NA	NA	NA	NA	NA
CG42481	0	NA	NA	NA	NA	NA
Sfp77F	0	NA	NA	NA	NA	NA
Sfp79B	0.178053	-1.16467	4.075067	-0.2858	0.775028	NA

Sfp84E	0	NA	NA	NA	NA	NA
Sfp87B	0	NA	NA	NA	NA	NA
Tdc1	27.30667	1.53463	3.952225	0.388295	0.697798	NA
nab	0	NA	NA	NA	NA	NA
CG42486	0	NA	NA	NA	NA	NA
CG42487	0	NA	NA	NA	NA	NA
CG42488	0	NA	NA	NA	NA	NA
lncRNA:CR	0	NA	NA	NA	NA	NA
CG17571	1.175108	-0.4198	3.916646	-0.10718	0.914644	NA
CG17570	0	NA	NA	NA	NA	NA
snoRNA:M	6.16458	0.254405	3.784263	0.067227	0.946401	NA
drd	0	NA	NA	NA	NA	NA
CG42493	38.86737	3.201514	3.90747	0.819332	0.412597	NA
NimC4	0	NA	NA	NA	NA	NA
CG42496	0	NA	NA	NA	NA	NA
CG42497	0	NA	NA	NA	NA	NA
CG42498	0	NA	NA	NA	NA	NA
CR42499	0	NA	NA	NA	NA	NA
CG42500	0	NA	NA	NA	NA	NA
CG42502	0	NA	NA	NA	NA	NA
Mocs2A	0	NA	NA	NA	NA	NA
CG42504	0	NA	NA	NA	NA	NA
CG42505	0	NA	NA	NA	NA	NA
CG42507	0	NA	NA	NA	NA	NA
Xport-B	0	NA	NA	NA	NA	NA
BBIP1	0	NA	NA	NA	NA	NA
CG42512	0	NA	NA	NA	NA	NA
mtg	2.097839	1.11873	3.972242	0.281637	0.778222	NA
CG42516	0	NA	NA	NA	NA	NA
CG42518	0	NA	NA	NA	NA	NA
CG17147	0.1047	0.613261	4.062799	0.150945	0.880019	NA
Pbp49	0	NA	NA	NA	NA	NA
MED9	0	NA	NA	NA	NA	NA
CG42525	0	NA	NA	NA	NA	NA
CG42529	0	NA	NA	NA	NA	NA
CR42530	0	NA	NA	NA	NA	NA
CG42531	0	NA	NA	NA	NA	NA
CR42532	0.890266	-1.59482	3.989917	-0.39971	0.689368	NA
CG42534	0.282677	-0.09599	4.057934	-0.02366	0.981127	NA
Antp	4.734085	-1.48459	3.957933	-0.37509	0.707592	NA
CG42535	0	NA	NA	NA	NA	NA
CG42536	0	NA	NA	NA	NA	NA
CG42537	0	NA	NA	NA	NA	NA
CG42538	4.856646	0.384027	3.957599	0.097035	0.922698	NA
Rrp40	1.884522	2.777951	3.96711	0.700245	0.483774	NA
serp	2.314693	-1.66033	3.966843	-0.41855	0.675543	NA
CG42544	5.943496	-1.90071	3.957353	-0.4803	0.631016	NA
CR42545	0.178053	-1.16467	4.075067	-0.2858	0.775028	NA
CR42546	0	NA	NA	NA	NA	NA
CR42548	0	NA	NA	NA	NA	NA

CG42556	8.085511	4.238261	3.901018	1.08645	0.27728	NA
CG42557	0	NA	NA	NA	NA	NA
CG42558	0	NA	NA	NA	NA	NA
CG42559	0	NA	NA	NA	NA	NA
CG42560	0	NA	NA	NA	NA	NA
CG42561	5.963526	1.517848	3.956843	0.383601	0.701274	NA
CG42562	4.019638	2.623574	3.884633	0.675372	0.499439	NA
CG42565	0	NA	NA	NA	NA	NA
CG42566	0	NA	NA	NA	NA	NA
CG42568	0	NA	NA	NA	NA	NA
DnaJ-60	0	NA	NA	NA	NA	NA
CG42570	0	NA	NA	NA	NA	NA
CG42571	0	NA	NA	NA	NA	NA
wisp	7.180792	-3.22342	3.855483	-0.83606	0.40312	NA
inaF-D	0	NA	NA	NA	NA	NA
CG42577	0	NA	NA	NA	NA	NA
CG42578	0	NA	NA	NA	NA	NA
CG42579	0	NA	NA	NA	NA	NA
CG42580	0	NA	NA	NA	NA	NA
CG42581	0	NA	NA	NA	NA	NA
CG42583	0	NA	NA	NA	NA	NA
cuff	8.553988	-1.01778	3.922987	-0.25944	0.795295	NA
bond	0	NA	NA	NA	NA	NA
CG42585	0	NA	NA	NA	NA	NA
CG42586	0	NA	NA	NA	NA	NA
CG42587	0	NA	NA	NA	NA	NA
MCPH1	40.86847	-2.95255	3.877731	-0.76141	0.446411	NA
CG42590	1.151699	1.609751	3.989673	0.403479	0.686596	NA
CG42591	0	NA	NA	NA	NA	NA
CG42592	0	NA	NA	NA	NA	NA
mei-38	0	NA	NA	NA	NA	NA
Incenp	0.178812	-1.08808	4.061735	-0.26789	0.788788	NA
CG42597	0	NA	NA	NA	NA	NA
CG42598	0.178053	-1.16467	4.075067	-0.2858	0.775028	NA
asl	4.397397	1.710278	3.962238	0.431644	0.666	NA
snoRNA:8:	1.000346	-0.36632	3.924715	-0.09334	0.925636	NA
clos	3.807412	-3.47991	3.840876	-0.90602	0.364925	NA
moi	0	NA	NA	NA	NA	NA
Cad86C	12.17836	-5.29011	3.829176	-1.38153	0.167117	NA
Sfp24Bc	10.30387	0.277343	3.953034	0.070159	0.944067	NA
Sfp26Ad	0	NA	NA	NA	NA	NA
Sfp36F	0	NA	NA	NA	NA	NA
Sfp38D	0	NA	NA	NA	NA	NA
Sfp78E	0.597459	1.53156	3.979367	0.384875	0.70033	NA
Sfp93F	0	NA	NA	NA	NA	NA
Sfp96F	0	NA	NA	NA	NA	NA
mrn	29.23132	-4.59508	3.781312	-1.21521	0.224287	NA
sing	4.471231	1.30049	3.925251	0.331314	0.740408	NA
zuc	0	NA	NA	NA	NA	NA
CG42615	0	NA	NA	NA	NA	NA

conv	0.104623	0.639075	4.067674	0.157111	0.875158	NA
Mat89Ba	35.75245	4.870061	3.82557	1.273029	0.203008	NA
CheB98a	0	NA	NA	NA	NA	NA
CheA84a	0	NA	NA	NA	NA	NA
CheA86a	0	NA	NA	NA	NA	NA
CheB53b	35.61066	-1.70495	3.951985	-0.43142	0.666165	NA
verm	0.356107	-1.47099	4.037764	-0.36431	0.715628	NA
Mst36Fb	0.830552	2.118611	3.94541	0.536981	0.591281	NA
Ccdc56	0	NA	NA	NA	NA	NA
CG42633	0	NA	NA	NA	NA	NA
CG42634	0	NA	NA	NA	NA	NA
CG42635	0	NA	NA	NA	NA	NA
CG42637	0	NA	NA	NA	NA	NA
CG42638	0.829716	2.152816	3.960988	0.543505	0.586782	NA
PPO3	0	NA	NA	NA	NA	NA
mtTFB1	0	NA	NA	NA	NA	NA
Gpa2	0	NA	NA	NA	NA	NA
alpha-Est5	1.235713	-0.14929	3.9675	-0.03763	0.969984	NA
Prosalpha3	0	NA	NA	NA	NA	NA
Pp1-Y1	0.178053	-1.16467	4.075067	-0.2858	0.775028	NA
Ir75c	0	NA	NA	NA	NA	NA
Ir75b	0	NA	NA	NA	NA	NA
lncRNA:CR	40.20534	-5.86724	3.82114	-1.53547	0.124668	NA
hkb	1.351755	-2.03446	3.928066	-0.51793	0.604508	NA
DhpD	14.96136	3.644878	3.902642	0.933951	0.350329	NA
CG13377	0.53416	-1.53481	4.012395	-0.38252	0.702078	NA
scaRNA:M	22.66263	-0.13623	3.850084	-0.03538	0.971774	NA
scaRNA:M	0.132078	-1.13211	4.069918	-0.27817	0.780885	NA
scaRNA:M	43.67276	-0.05665	3.443182	-0.01645	0.986872	NA
scaRNA:M	0.707123	-2.68537	4.000063	-0.67133	0.502009	NA
CG42649	0	NA	NA	NA	NA	NA
CG42650	0	NA	NA	NA	NA	NA
lncRNA:CR	14.60281	0.425506	3.752548	0.113391	0.90972	NA
7SLRNA:CF	2.113346	-2.98132	3.916163	-0.76129	0.446486	NA
CR42653	4.292697	1.709267	3.962502	0.431361	0.666206	NA
CG42655	38.51182	-0.35109	3.835396	-0.09154	0.927064	NA
NimA	37.40553	0.004635	3.951466	0.001173	0.999064	NA
lncRNA:CR	0	NA	NA	NA	NA	NA
CG42658	0	NA	NA	NA	NA	NA
NT1	0	NA	NA	NA	NA	NA
ms(2)34Fe	0.356107	-1.47099	4.037764	-0.36431	0.715628	NA
CG42659	0	NA	NA	NA	NA	NA
l(2)34Fc	0	NA	NA	NA	NA	NA
l(2)34Fd	4.273279	-1.68149	3.959707	-0.42465	0.671092	NA
CG42660	0	NA	NA	NA	NA	NA
CG42661	0	NA	NA	NA	NA	NA
CG42662	0	NA	NA	NA	NA	NA
CG42675	0	NA	NA	NA	NA	NA
CG42680	0	NA	NA	NA	NA	NA
CG42681	0	NA	NA	NA	NA	NA

CG42682	0	NA	NA	NA	NA	NA
CG42685	3.552619	-1.52136	3.905383	-0.38956	0.696865	NA
tobi	0	NA	NA	NA	NA	NA
CG42690	0	NA	NA	NA	NA	NA
CG42691	0	NA	NA	NA	NA	NA
CG42692	0	NA	NA	NA	NA	NA
CG42693	0.178812	-1.08808	4.061735	-0.26789	0.788788	NA
CG42694	0.178812	-1.08808	4.061735	-0.26789	0.788788	NA
lncRNA:CR	0	NA	NA	NA	NA	NA
CG42697	0	NA	NA	NA	NA	NA
CG42703	0	NA	NA	NA	NA	NA
CG42704	0	NA	NA	NA	NA	NA
CG42705	0	NA	NA	NA	NA	NA
I-3	20.38222	6.791841	3.911947	1.736179	0.082532	NA
CG42710	0.712213	-1.57119	3.998613	-0.39293	0.694369	NA
CG42711	0	NA	NA	NA	NA	NA
CG42713	0	NA	NA	NA	NA	NA
CG42714	0	NA	NA	NA	NA	NA
lncRNA:CR	0	NA	NA	NA	NA	NA
CG42716	0	NA	NA	NA	NA	NA
CG42717	0	NA	NA	NA	NA	NA
CG42718	0	NA	NA	NA	NA	NA
lncRNA:CR	0.209247	1.042804	4.047837	0.25762	0.7967	NA
CR42722	0	NA	NA	NA	NA	NA
CR42723	0	NA	NA	NA	NA	NA
Npc1b	1.479466	-0.59427	3.946397	-0.15059	0.880302	NA
CG42726	0	NA	NA	NA	NA	NA
CG42727	0	NA	NA	NA	NA	NA
CG42728	0	NA	NA	NA	NA	NA
CG42729	0	NA	NA	NA	NA	NA
B9d2	0	NA	NA	NA	NA	NA
CG42733	7.966422	-3.11497	3.915222	-0.79561	0.426261	NA
tctn	0	NA	NA	NA	NA	NA
lncRNA:CR	0	NA	NA	NA	NA	NA
asRNA:CR4	0	NA	NA	NA	NA	NA
CG42740	0	NA	NA	NA	NA	NA
asRNA:CR4	0	NA	NA	NA	NA	NA
asRNA:CR4	59.53063	1.53725	3.951513	0.389028	0.697255	NA
lncRNA:CR	36.31316	-7.82544	3.911473	-2.00064	0.045431	NA
Cpn	0	NA	NA	NA	NA	NA
dsx-c73A	9.76145	-0.30999	3.87651	-0.07997	0.936264	NA
CG42747	39.04694	6.372122	3.869921	1.646577	0.099645	NA
CG42749	0.932373	1.053996	3.996193	0.26375	0.791972	NA
CG42752	0	NA	NA	NA	NA	NA
CG42753	0.2094	1.005034	4.045341	0.248442	0.803792	NA
lncRNA:CR	0	NA	NA	NA	NA	NA
lncRNA:CR	0	NA	NA	NA	NA	NA
CG42758	0	NA	NA	NA	NA	NA
CG42759	44.7539	1.177044	3.95196	0.297838	0.765827	NA
CG42762	0	NA	NA	NA	NA	NA

CG42763	0	NA	NA	NA	NA	NA
lncRNA:CR	3.452567	1.502955	3.961005	0.379438	0.704363	NA
pre-mod(n	0	NA	NA	NA	NA	NA
pre-mod(n	0	NA	NA	NA	NA	NA
pre-mod(n	0	NA	NA	NA	NA	NA
pre-mod(n	0	NA	NA	NA	NA	NA
pre-mod(n	0	NA	NA	NA	NA	NA
pre-mod(n	0	NA	NA	NA	NA	NA
pre-mod(n	0	NA	NA	NA	NA	NA
pre-mod(n	0	NA	NA	NA	NA	NA
pre-mod(n	0	NA	NA	NA	NA	NA
CG42779	0	NA	NA	NA	NA	NA
CG42780	0	NA	NA	NA	NA	NA
CG42781	0	NA	NA	NA	NA	NA
CG42782	0	NA	NA	NA	NA	NA
CR42785	0	NA	NA	NA	NA	NA
CR42786	0	NA	NA	NA	NA	NA
Mzt1	0	NA	NA	NA	NA	NA
lncRNA:CR	0	NA	NA	NA	NA	NA
lncRNA:CR	0	NA	NA	NA	NA	NA
CG42792	0	NA	NA	NA	NA	NA
lncRNA:CR	0	NA	NA	NA	NA	NA
vnd	0.945306	-0.54642	3.960875	-0.13795	0.890276	NA
CG42798	0	NA	NA	NA	NA	NA
srw	0	NA	NA	NA	NA	NA
snoRNA:O	0	NA	NA	NA	NA	NA
snoRNA:O	0	NA	NA	NA	NA	NA
Pxt	0	NA	NA	NA	NA	NA
CG42807	1.072115	-2.75701	4.00642	-0.68815	0.491359	NA
CG42809	8.158263	1.163335	3.956616	0.294023	0.768741	NA
CG42810	23.51641	2.298851	3.841718	0.598391	0.549579	NA
CG42811	0	NA	NA	NA	NA	NA
CG42812	0	NA	NA	NA	NA	NA
CG42814	0	NA	NA	NA	NA	NA
CG42815	0	NA	NA	NA	NA	NA
CG42816	0.337716	1.464149	4.04412	0.362044	0.717319	NA
CG42818	0.236778	-0.26946	4.071573	-0.06618	0.947234	NA
CG42819	0	NA	NA	NA	NA	NA
CG42820	0	NA	NA	NA	NA	NA
CG42821	0.1047	0.613261	4.062799	0.150945	0.880019	NA
CG42822	0	NA	NA	NA	NA	NA
CG42823	0.178812	-1.08808	4.061735	-0.26789	0.788788	NA
CG42824	0	NA	NA	NA	NA	NA
CG42825	57.9821	-1.92075	3.95158	-0.48607	0.626916	NA
CG42826	0	NA	NA	NA	NA	NA
CG42828	35.97601	-8.3268	3.908845	-2.13025	0.033151	NA
CG42830	2.773631	-1.87679	3.964396	-0.47341	0.63592	NA
CG42831	0.64424	-0.14539	3.993669	-0.0364	0.97096	NA
CG42832	0	NA	NA	NA	NA	NA
CG42833	0	NA	NA	NA	NA	NA

CG42834	0	NA	NA	NA	NA	NA
CG42835	0	NA	NA	NA	NA	NA
lncRNA:CR	0.178053	-1.16467	4.075067	-0.2858	0.775028	NA
CG42837	0	NA	NA	NA	NA	NA
CR42838	0	NA	NA	NA	NA	NA
lncRNA:CR	0.356107	-1.47099	4.037764	-0.36431	0.715628	NA
d	0	NA	NA	NA	NA	NA
CG42841	1.109413	-0.57335	3.886322	-0.14753	0.882713	NA
CR42842	0	NA	NA	NA	NA	NA
lncRNA:CR	0.132078	-1.13211	4.069918	-0.27817	0.780885	NA
CG42846	35.64305	4.699317	3.829871	1.227017	0.219816	NA
CG42847	0	NA	NA	NA	NA	NA
lncRNA:CR	0	NA	NA	NA	NA	NA
CG42851	0	NA	NA	NA	NA	NA
CG42852	2.314693	-1.66033	3.966843	-0.41855	0.675543	NA
CG42853	0.178053	-1.16467	4.075067	-0.2858	0.775028	NA
CG42854	0	NA	NA	NA	NA	NA
CG42855	1.71701	-1.85109	3.972145	-0.46602	0.641203	NA
CG42857	0	NA	NA	NA	NA	NA
lncRNA:CR	0	NA	NA	NA	NA	NA
lncRNA:CR	0.132078	-1.13211	4.069918	-0.27817	0.780885	NA
asRNA:CR4	0.104623	0.639075	4.067674	0.157111	0.875158	NA
f	0	NA	NA	NA	NA	NA
sro	0.765012	-1.03648	3.979317	-0.26047	0.794504	NA
CG42866	0.528311	-1.72839	4.011871	-0.43082	0.6666	NA
CG42867	14.83604	1.618941	3.954063	0.409437	0.682219	NA
lncRNA:CR	0	NA	NA	NA	NA	NA
CG42869	0	NA	NA	NA	NA	NA
CG42870	0	NA	NA	NA	NA	NA
lncRNA:CR	15.00928	0.029243	3.737531	0.007824	0.993757	NA
CG42876	0	NA	NA	NA	NA	NA
betaNACte	0	NA	NA	NA	NA	NA
CG43049	0	NA	NA	NA	NA	NA
CG43050	0.356107	-1.47099	4.037764	-0.36431	0.715628	NA
CG43051	11.67348	-7.01189	3.911387	-1.79269	0.073023	NA
CG43052	0	NA	NA	NA	NA	NA
lncRNA:CR	0	NA	NA	NA	NA	NA
CG43054	0	NA	NA	NA	NA	NA
CG43055	0	NA	NA	NA	NA	NA
CG43057	0	NA	NA	NA	NA	NA
CG43058	0	NA	NA	NA	NA	NA
CG43059	0	NA	NA	NA	NA	NA
CG43060	0	NA	NA	NA	NA	NA
CG43061	0	NA	NA	NA	NA	NA
CG43062	0	NA	NA	NA	NA	NA
CG43063	0	NA	NA	NA	NA	NA
CG43064	0	NA	NA	NA	NA	NA
CG43068	28.3967	-1.9183	3.952279	-0.48537	0.627417	NA
CG43069	0.178053	-1.16467	4.075067	-0.2858	0.775028	NA
CG43070	0.1047	0.613261	4.062799	0.150945	0.880019	NA

CG43071	0	NA	NA	NA	NA	NA
CG43072	0	NA	NA	NA	NA	NA
CG43074	57.45379	-1.92073	3.951586	-0.48607	0.626921	NA
CG43075	0	NA	NA	NA	NA	NA
CG43076	0	NA	NA	NA	NA	NA
CG43077	0	NA	NA	NA	NA	NA
CR43080	0	NA	NA	NA	NA	NA
Vha16-4	0	NA	NA	NA	NA	NA
VhaPPA1-2	0	NA	NA	NA	NA	NA
Trpml	0	NA	NA	NA	NA	NA
CG43082	0	NA	NA	NA	NA	NA
CG43083	0	NA	NA	NA	NA	NA
CG43084	0.104623	0.639075	4.067674	0.157111	0.875158	NA
CG43088	0	NA	NA	NA	NA	NA
CG43089	4.289554	1.509777	3.959091	0.381344	0.702948	NA
CG43090	0	NA	NA	NA	NA	NA
CG43094	37.60846	0.343026	3.839417	0.089343	0.928809	NA
CG43095	55.28149	2.975539	3.918979	0.759264	0.447695	NA
CR43096	0	NA	NA	NA	NA	NA
CG43098	30.78178	1.747963	3.952592	0.442232	0.658321	NA
CR43100	0	NA	NA	NA	NA	NA
CG43101	0	NA	NA	NA	NA	NA
lncRNA:CR	0	NA	NA	NA	NA	NA
CR43105	0.31387	1.261782	4.037145	0.312543	0.754628	NA
CG43106	0	NA	NA	NA	NA	NA
CG43107	41.31225	-2.64231	3.737997	-0.70688	0.479642	NA
CG43108	2.092465	1.481166	3.9672	0.373353	0.708886	NA
CG43109	0	NA	NA	NA	NA	NA
CG43111	0.942299	1.584447	3.996979	0.396411	0.691802	NA
CG43112	0	NA	NA	NA	NA	NA
CG43114	0	NA	NA	NA	NA	NA
CG43116	0	NA	NA	NA	NA	NA
CG43117	10.96245	-1.91078	3.954436	-0.4832	0.628954	NA
CG43120	0	NA	NA	NA	NA	NA
CG43121	0	NA	NA	NA	NA	NA
CG43123	0	NA	NA	NA	NA	NA
CG43124	0	NA	NA	NA	NA	NA
CG43127	0.1047	0.613261	4.062799	0.150945	0.880019	NA
CheA46a	0	NA	NA	NA	NA	NA
CheA56a	0	NA	NA	NA	NA	NA
CG43131	0	NA	NA	NA	NA	NA
lncRNA:CR	0	NA	NA	NA	NA	NA
CG43133	0	NA	NA	NA	NA	NA
CG43134	0	NA	NA	NA	NA	NA
CG43135	0	NA	NA	NA	NA	NA
CG43136	3.921726	-4.48744	3.92807	-1.1424	0.253286	NA
CG43137	0	NA	NA	NA	NA	NA
CG43138	0	NA	NA	NA	NA	NA
CG43139	0	NA	NA	NA	NA	NA
CR43142	0	NA	NA	NA	NA	NA

DNAlig1	13.02091	-1.82671	3.846843	-0.47486	0.634886	NA
lncRNA:CR	0	NA	NA	NA	NA	NA
CG43145	0	NA	NA	NA	NA	NA
lncRNA:CR	0.233093	0.692656	4.076246	0.169925	0.865069	NA
CG43147	0.178053	-1.16467	4.075067	-0.2858	0.775028	NA
Tmhs	0	NA	NA	NA	NA	NA
lncRNA:CR	0	NA	NA	NA	NA	NA
CG43153	0	NA	NA	NA	NA	NA
CG43155	17.80533	-1.70165	3.953057	-0.43046	0.666859	NA
CG43156	0	NA	NA	NA	NA	NA
lncRNA:CR	0.104623	0.639075	4.067674	0.157111	0.875158	NA
CR43158	0	NA	NA	NA	NA	NA
asRNA:CR4	5.025597	1.715508	3.960878	0.433113	0.664933	NA
asRNA:CR4	0	NA	NA	NA	NA	NA
CG43163	7.631301	-1.99716	3.828612	-0.52164	0.601921	NA
CG43165	0	NA	NA	NA	NA	NA
lncRNA:CR	0	NA	NA	NA	NA	NA
CG43167	0	NA	NA	NA	NA	NA
CG43168	0	NA	NA	NA	NA	NA
CG43169	0	NA	NA	NA	NA	NA
CG43171	0	NA	NA	NA	NA	NA
CG43172	0	NA	NA	NA	NA	NA
CG43175	0.209247	1.042804	4.047837	0.25762	0.7967	NA
CR43176	0	NA	NA	NA	NA	NA
CR43177	24.56645	-1.91756	3.952492	-0.48515	0.627569	NA
CG43178	0	NA	NA	NA	NA	NA
CG43179	52.24526	1.750729	3.951902	0.443009	0.657759	NA
CG43181	0	NA	NA	NA	NA	NA
CG43182	0	NA	NA	NA	NA	NA
CG43183	0.357625	-1.47707	4.044794	-0.36518	0.714979	NA
CG43184	1.849088	-1.85577	3.970721	-0.46736	0.640241	NA
CG43185	0	NA	NA	NA	NA	NA
CR43186	12.33581	0.034503	3.952607	0.008729	0.993035	NA
CG43187	0.178053	-1.16467	4.075067	-0.2858	0.775028	NA
CG43188	0	NA	NA	NA	NA	NA
CG43190	0	NA	NA	NA	NA	NA
CG43192	9.789916	1.166086	3.955676	0.294788	0.768156	NA
lncRNA:CR	0.356107	-1.47099	4.037764	-0.36431	0.715628	NA
hemo	0	NA	NA	NA	NA	NA
CG43195	0	NA	NA	NA	NA	NA
CG43198	0	NA	NA	NA	NA	NA
CG43200	0	NA	NA	NA	NA	NA
CG43201	0	NA	NA	NA	NA	NA
BomT1	0	NA	NA	NA	NA	NA
CG43203	1.056573	-1.55533	3.944973	-0.39426	0.693391	NA
CG43204	7.22589	1.161221	3.95734	0.293435	0.76919	NA
CG43206	0	NA	NA	NA	NA	NA
CG43207	0	NA	NA	NA	NA	NA
CG43208	0	NA	NA	NA	NA	NA
CG43209	0.104623	0.639075	4.067674	0.157111	0.875158	NA

CG43210	0	NA	NA	NA	NA	NA
CR43211	0	NA	NA	NA	NA	NA
CR43212	6.793086	-0.22491	3.755522	-0.05989	0.952246	NA
CR43213	0	NA	NA	NA	NA	NA
CR43214	0	NA	NA	NA	NA	NA
CR43215	0	NA	NA	NA	NA	NA
CR43216	0	NA	NA	NA	NA	NA
CR43217	0	NA	NA	NA	NA	NA
CG43218	0.3141	1.240997	4.03841	0.307299	0.758616	NA
CG43220	0	NA	NA	NA	NA	NA
CG43221	0	NA	NA	NA	NA	NA
Diedel2	0	NA	NA	NA	NA	NA
CG43230	0	NA	NA	NA	NA	NA
CG43232	1.633826	-0.93012	3.966011	-0.23452	0.81458	NA
CG43233	0.523193	2.185419	4.000542	0.546281	0.584873	NA
lncRNA:CR	0	NA	NA	NA	NA	NA
CG43235	0.104623	0.639075	4.067674	0.157111	0.875158	NA
Mtkl	0	NA	NA	NA	NA	NA
CG43237	0.5235	1.490714	4.02555	0.370313	0.711149	NA
lncRNA:CR	0	NA	NA	NA	NA	NA
CR43240	0	NA	NA	NA	NA	NA
asRNA:CR4	0.356107	-1.47099	4.037764	-0.36431	0.715628	NA
CG43244	0	NA	NA	NA	NA	NA
CG43246	0	NA	NA	NA	NA	NA
lncRNA:CR	0	NA	NA	NA	NA	NA
CG43248	0.31387	1.261782	4.037145	0.312543	0.754628	NA
CG43249	0	NA	NA	NA	NA	NA
lncRNA:CR	0	NA	NA	NA	NA	NA
CG43251	0	NA	NA	NA	NA	NA
lncRNA:CR	0	NA	NA	NA	NA	NA
lncRNA:CR	0	NA	NA	NA	NA	NA
CG43254	0	NA	NA	NA	NA	NA
asRNA:CR4	0	NA	NA	NA	NA	NA
lncRNA:CR	0.104623	0.639075	4.067674	0.157111	0.875158	NA
lncRNA:CR	0	NA	NA	NA	NA	NA
lncRNA:CR	0	NA	NA	NA	NA	NA
lncRNA:CR	0	NA	NA	NA	NA	NA
lncRNA:CR	33.28143	-1.76241	3.952832	-0.44586	0.655698	NA
CG43267	0	NA	NA	NA	NA	NA
lncRNA:CR	0	NA	NA	NA	NA	NA
CG43271	0	NA	NA	NA	NA	NA
CG43272	0.178812	-1.08808	4.061735	-0.26789	0.788788	NA
CG43273	22.18905	-1.91696	3.952661	-0.48498	0.62769	NA
lncRNA:CR	0	NA	NA	NA	NA	NA
CR43275	19.45993	1.532694	3.952753	0.387754	0.698198	NA
CR43276	22.532	2.734609	3.820367	0.715797	0.474116	NA
CG43277	0	NA	NA	NA	NA	NA
lncRNA:CR	0.132078	-1.13211	4.069918	-0.27817	0.780885	NA
lncRNA:CR	1.71701	-1.85109	3.972145	-0.46602	0.641203	NA
lncRNA:CR	0	NA	NA	NA	NA	NA

CR43281	0	NA	NA	NA	NA	NA
lncRNA:CR	0	NA	NA	NA	NA	NA
lncRNA:ch	28.60885	-2.67685	3.849013	-0.69546	0.486764	NA
lncRNA:CR	0	NA	NA	NA	NA	NA
lncRNA:CR	26.85773	4.170803	3.803627	1.096533	0.272846	NA
CG43288	0	NA	NA	NA	NA	NA
lncRNA:CR	0.660388	-1.75977	4.001266	-0.4398	0.660079	NA
CG43290	0	NA	NA	NA	NA	NA
CG43291	0	NA	NA	NA	NA	NA
CG43292	0	NA	NA	NA	NA	NA
CG43293	14.65799	1.740703	3.954419	0.440192	0.659798	NA
CG43294	0	NA	NA	NA	NA	NA
CG43296	0	NA	NA	NA	NA	NA
lncRNA:CR	0	NA	NA	NA	NA	NA
lncRNA:CR	0	NA	NA	NA	NA	NA
asRNA:CR4	0.178053	-1.16467	4.075067	-0.2858	0.775028	NA
lncRNA:CR	0	NA	NA	NA	NA	NA
lncRNA:CR	0	NA	NA	NA	NA	NA
lncRNA:CR	4.603653	3.529285	3.923082	0.899621	0.368322	NA
lncRNA:CR	0	NA	NA	NA	NA	NA
CG43307	0	NA	NA	NA	NA	NA
CG43308	0	NA	NA	NA	NA	NA
CR43309	10.43414	-1.91017	3.954613	-0.48302	0.629079	NA
CR43310	3.017037	1.618643	3.847755	0.420672	0.673995	NA
CG43312	9.712619	2.649761	3.874158	0.683958	0.494002	NA
snRNA:U4	0.132078	-1.13211	4.069918	-0.27817	0.780885	NA
lncRNA:CR	4.473346	-0.84823	3.837462	-0.22104	0.825062	NA
CG43315	0	NA	NA	NA	NA	NA
CG43316	0	NA	NA	NA	NA	NA
CG43317	26.0193	-1.91787	3.952404	-0.48524	0.627506	NA
CG43318	0	NA	NA	NA	NA	NA
CG43319	0	NA	NA	NA	NA	NA
CG43321	0	NA	NA	NA	NA	NA
CG43325	0	NA	NA	NA	NA	NA
CG43326	0	NA	NA	NA	NA	NA
CG43327	0	NA	NA	NA	NA	NA
CG43328	0	NA	NA	NA	NA	NA
CG43329	27.62154	1.898482	3.856334	0.492302	0.622506	NA
CG43330	0	NA	NA	NA	NA	NA
CG43331	0	NA	NA	NA	NA	NA
CG43332	0.178812	-1.08808	4.061735	-0.26789	0.788788	NA
CG43333	0.178053	-1.16467	4.075067	-0.2858	0.775028	NA
CG43335	0	NA	NA	NA	NA	NA
CG43336	0	NA	NA	NA	NA	NA
CG43337	7.665106	2.834977	3.918606	0.723466	0.469394	NA
CG43341	0	NA	NA	NA	NA	NA
CG43342	55.35544	8.230467	3.901873	2.109363	0.034913	NA
CG43345	0	NA	NA	NA	NA	NA
CG43346	0	NA	NA	NA	NA	NA
Klp54D	2.346923	-0.0014	3.913938	-0.00036	0.999715	NA

CG43339	0	NA	NA	NA	NA	NA
CG43338	0.536437	-1.71175	4.038042	-0.42391	0.671635	NA
CG43348	0	NA	NA	NA	NA	NA
CG43349	0	NA	NA	NA	NA	NA
CG43350	0	NA	NA	NA	NA	NA
CG43351	0	NA	NA	NA	NA	NA
HP1Lcsd	0	NA	NA	NA	NA	NA
lncRNA:CR	0	NA	NA	NA	NA	NA
CG43354	0	NA	NA	NA	NA	NA
CG43355	0	NA	NA	NA	NA	NA
lncRNA:CR	0	NA	NA	NA	NA	NA
lncRNA:CR	0	NA	NA	NA	NA	NA
lncRNA:CR	0	NA	NA	NA	NA	NA
CR43359	0.462248	-0.67622	4.039367	-0.16741	0.867049	NA
CR43360	0	NA	NA	NA	NA	NA
CR43361	2.735658	4.81469	3.922071	1.227589	0.219601	NA
CG43362	0	NA	NA	NA	NA	NA
CR43364	0	NA	NA	NA	NA	NA
CG43366	1.835442	2.985712	3.902254	0.765125	0.444197	NA
Cc2d2a	7.154728	4.211453	3.881569	1.084987	0.277927	NA
tx	0	NA	NA	NA	NA	NA
Prosalpha1	0	NA	NA	NA	NA	NA
lncRNA:CR	0.178053	-1.16467	4.075067	-0.2858	0.775028	NA
vret	0.53416	-1.53481	4.012395	-0.38252	0.702078	NA
scaRNA:M	0	NA	NA	NA	NA	NA
CG43376	0	NA	NA	NA	NA	NA
CR43377	0	NA	NA	NA	NA	NA
CR43378	0	NA	NA	NA	NA	NA
CR43379	0	NA	NA	NA	NA	NA
CR43380	0	NA	NA	NA	NA	NA
CR43381	0	NA	NA	NA	NA	NA
CR43382	0	NA	NA	NA	NA	NA
CR43383	0	NA	NA	NA	NA	NA
CR43384	0	NA	NA	NA	NA	NA
CR43385	0	NA	NA	NA	NA	NA
CG43386	36.2325	0.205367	3.72082	0.055194	0.955984	NA
lncRNA:CR	0	NA	NA	NA	NA	NA
robls54B	26.38438	1.74685	3.952871	0.441919	0.658548	NA
Phae1	0.1047	0.613261	4.062799	0.150945	0.880019	NA
Phae2	0	NA	NA	NA	NA	NA
dar1	10.01121	-4.6891	3.916556	-1.19725	0.231209	NA
Coop	0.1047	0.613261	4.062799	0.150945	0.880019	NA
CG43389	0	NA	NA	NA	NA	NA
CG43390	0	NA	NA	NA	NA	NA
CG43391	0	NA	NA	NA	NA	NA
CG43392	0	NA	NA	NA	NA	NA
CG43393	0	NA	NA	NA	NA	NA
vnc	0	NA	NA	NA	NA	NA
chas	27.06311	-1.08588	3.823342	-0.28401	0.776399	NA
CG43396	49.10427	1.75047	3.951966	0.442936	0.657812	NA

CG43397	0	NA	NA	NA	NA	NA
lncRNA:CR	34.13531	1.47028	3.813895	0.385506	0.699862	NA
CG43400	0	NA	NA	NA	NA	NA
CG43401	0	NA	NA	NA	NA	NA
CG43402	0	NA	NA	NA	NA	NA
CG43403	0	NA	NA	NA	NA	NA
lncRNA:CR	0	NA	NA	NA	NA	NA
CG43405	0.178053	-1.16467	4.075067	-0.2858	0.775028	NA
CG43407	0	NA	NA	NA	NA	NA
lncRNA:CR	0	NA	NA	NA	NA	NA
lncRNA:CR	0	NA	NA	NA	NA	NA
lncRNA:CR	3.455098	1.699144	3.965156	0.428519	0.668273	NA
lncRNA:CR	0	NA	NA	NA	NA	NA
asRNA:CR4	56.58737	1.153545	3.951728	0.291909	0.770356	NA
lncRNA:CR	0	NA	NA	NA	NA	NA
lncRNA:CR	0	NA	NA	NA	NA	NA
lncRNA:CR	0	NA	NA	NA	NA	NA
lncRNA:CR	0	NA	NA	NA	NA	NA
lncRNA:CR	0	NA	NA	NA	NA	NA
lncRNA:CR	1.675199	1.648777	3.978727	0.414398	0.678583	NA
lncRNA:CR	0	NA	NA	NA	NA	NA
lncRNA:CR	0	NA	NA	NA	NA	NA
lncRNA:CR	0	NA	NA	NA	NA	NA
lncRNA:CR	0	NA	NA	NA	NA	NA
lncRNA:CR	0	NA	NA	NA	NA	NA
asRNA:CR4	0	NA	NA	NA	NA	NA
asRNA:CR4	52.51587	0.185741	3.83494	0.048434	0.961371	NA
CG43428	2.411839	-0.34285	3.871222	-0.08856	0.929428	NA
asRNA:CR4	0	NA	NA	NA	NA	NA
asRNA:CR4	0	NA	NA	NA	NA	NA
lncRNA:CR	19.36949	1.74405	3.953574	0.441132	0.659117	NA
lncRNA:CR	0	NA	NA	NA	NA	NA
asRNA:CR4	4.719178	3.907158	3.916764	0.997547	0.318499	NA
lncRNA:CR	0	NA	NA	NA	NA	NA
lncRNA:CR	0	NA	NA	NA	NA	NA
lncRNA:CR	0	NA	NA	NA	NA	NA
lncRNA:CR	0	NA	NA	NA	NA	NA
CG43438	0	NA	NA	NA	NA	NA
CG43439	0	NA	NA	NA	NA	NA
lncRNA:CR	0.356107	-1.47099	4.037764	-0.36431	0.715628	NA
CR43441	0	NA	NA	NA	NA	NA
Nha2	0.104623	0.639075	4.067674	0.157111	0.875158	NA
CG43445	29.05709	-1.91841	3.952248	-0.4854	0.627395	NA
CG43446	34.10726	2.77171	3.917435	0.707532	0.479236	NA
CG43447	0	NA	NA	NA	NA	NA
CG43448	0.2094	1.005034	4.045341	0.248442	0.803792	NA
CG43449	0	NA	NA	NA	NA	NA
CG43450	0	NA	NA	NA	NA	NA
lncRNA:CR	0.178053	-1.16467	4.075067	-0.2858	0.775028	NA
lncRNA:CR	39.12912	1.190072	3.740117	0.318191	0.75034	NA

lncRNA:CR	0.1047	0.613261	4.062799	0.150945	0.880019	NA
asRNA:CR4	0	NA	NA	NA	NA	NA
lncRNA:CR	0	NA	NA	NA	NA	NA
lncRNA:CR	0	NA	NA	NA	NA	NA
lncRNA:CR	0	NA	NA	NA	NA	NA
lncRNA:CR	0	NA	NA	NA	NA	NA
lncRNA:CR	58.22255	-2.15672	3.95241	-0.54567	0.585291	NA
asRNA:CR4	0	NA	NA	NA	NA	NA
asRNA:CR4	0	NA	NA	NA	NA	NA
CG5819	0	NA	NA	NA	NA	NA
pgc	14.7066	-3.4891	3.904725	-0.89356	0.371559	NA
CG11298	0.356866	-1.92326	4.037889	-0.4763	0.633858	NA
CG11362	0.178053	-1.16467	4.075067	-0.2858	0.775028	NA
CG10344	0	NA	NA	NA	NA	NA
babos	0	NA	NA	NA	NA	NA
T3dh	32.75527	-1.91894	3.952097	-0.48555	0.627287	NA
CG3927	0	NA	NA	NA	NA	NA
CG4269	0.1047	0.613261	4.062799	0.150945	0.880019	NA
nsr	0.283512	-0.2331	4.065094	-0.05734	0.954273	NA
CG13510	0	NA	NA	NA	NA	NA
CG13511	0	NA	NA	NA	NA	NA
CG4250	0.31089	-1.9003	4.033427	-0.47114	0.637542	NA
CG30273	0	NA	NA	NA	NA	NA
CG30269	0	NA	NA	NA	NA	NA
Obp59a	0	NA	NA	NA	NA	NA
Obp58d	0	NA	NA	NA	NA	NA
CG30275	0.104623	0.639075	4.067674	0.157111	0.875158	NA
CG30268	0.236701	-0.24276	4.020028	-0.06039	0.951846	NA
CG13526	2.198698	1.671135	3.972627	0.420662	0.674002	NA
asRNA:CR3	1.264786	0.992839	3.920274	0.253257	0.800069	NA
CG30271	32.09096	-1.33351	3.951859	-0.33744	0.735787	NA
MFS1	0	NA	NA	NA	NA	NA
CG30265	0	NA	NA	NA	NA	NA
CG12490	0	NA	NA	NA	NA	NA
CG9825	0	NA	NA	NA	NA	NA
CG9826	0	NA	NA	NA	NA	NA
CG3649	0	NA	NA	NA	NA	NA
MED23	4.910335	4.391786	3.877589	1.132608	0.257379	NA
inaD	1.412728	-3.67881	3.95877	-0.92928	0.352743	NA
CycB	0	NA	NA	NA	NA	NA
Obp58c	0	NA	NA	NA	NA	NA
CG30259	0.53416	-1.53481	4.012395	-0.38252	0.702078	NA
CG30196	0	NA	NA	NA	NA	NA
Cyp6d2	0	NA	NA	NA	NA	NA
CG3746	0	NA	NA	NA	NA	NA
CG30195	0	NA	NA	NA	NA	NA
CG4329	0	NA	NA	NA	NA	NA
tRNA:Ser-T	0	NA	NA	NA	NA	NA
CG3085	4.816197	1.713907	3.961293	0.432664	0.665259	NA
Gr59a	0	NA	NA	NA	NA	NA

Gr59b	0.712213	-1.57119	3.998613	-0.39293	0.694369	NA
CG30192	0	NA	NA	NA	NA	NA
Klp59D	0	NA	NA	NA	NA	NA
CG9899	0	NA	NA	NA	NA	NA
CG13545	0	NA	NA	NA	NA	NA
CG13544	0.178812	-1.08808	4.061735	-0.26789	0.788788	NA
Gpdh2	3.258635	-0.08803	3.89017	-0.02263	0.981947	NA
Klp59C	0	NA	NA	NA	NA	NA
Gr59d	0.233093	0.692656	4.076246	0.169925	0.865069	NA
Gr59c	0	NA	NA	NA	NA	NA
RpL37b	8.849205	-1.9079	3.955267	-0.48237	0.629543	NA
CG9876	0	NA	NA	NA	NA	NA
CG9877	0.178053	-1.16467	4.075067	-0.2858	0.775028	NA
Fib	58.69368	-1.6645	3.477177	-0.47869	0.632158	NA
tRNA:Ser-T	0	NA	NA	NA	NA	NA
CG9897	0	NA	NA	NA	NA	NA
CG32833	0.178053	-1.16467	4.075067	-0.2858	0.775028	NA
CG32834	0	NA	NA	NA	NA	NA
lncRNA:CR	0	NA	NA	NA	NA	NA
CG3788	56.8878	-1.20427	3.951673	-0.30475	0.760558	NA
CG30187	0.651586	2.224386	4.007948	0.554994	0.578899	NA
CG3092	0	NA	NA	NA	NA	NA
CG13540	0	NA	NA	NA	NA	NA
CG13541	0.178053	-1.16467	4.075067	-0.2858	0.775028	NA
CG9863	0	NA	NA	NA	NA	NA
CR30409	0	NA	NA	NA	NA	NA
CG9875	0	NA	NA	NA	NA	NA
yellow-d	0	NA	NA	NA	NA	NA
yellow-d2	0.178053	-1.16467	4.075067	-0.2858	0.775028	NA
CG30411	0	NA	NA	NA	NA	NA
CG13557	0	NA	NA	NA	NA	NA
Or59c	0	NA	NA	NA	NA	NA
Or59b	0.178053	-1.16467	4.075067	-0.2858	0.775028	NA
SkpF	0.104623	0.639075	4.067674	0.157111	0.875158	NA
Or59a	0	NA	NA	NA	NA	NA
CG9815	0	NA	NA	NA	NA	NA
CG9812	0.341324	0.112818	4.000379	0.028202	0.977501	NA
CG30414	0.178053	-1.16467	4.075067	-0.2858	0.775028	NA
CG3502	0.178053	-1.16467	4.075067	-0.2858	0.775028	NA
CG30413	0	NA	NA	NA	NA	NA
tbrd-3	0	NA	NA	NA	NA	NA
CG30412	0.178053	-1.16467	4.075067	-0.2858	0.775028	NA
Prosbeta5I	0	NA	NA	NA	NA	NA
CG3124	25.87227	-7.09074	3.888022	-1.82374	0.068191	NA
ord	0	NA	NA	NA	NA	NA
CG12782	0	NA	NA	NA	NA	NA
RpL22-like	0	NA	NA	NA	NA	NA
yip3	0	NA	NA	NA	NA	NA
LS2	17.1701	-1.91519	3.95317	-0.48447	0.628053	NA
CG13559	0.178053	-1.16467	4.075067	-0.2858	0.775028	NA

Gr59f	0.660388	-1.75977	4.001266	-0.4398	0.660079	NA
ItgaPS5	0	NA	NA	NA	NA	NA
CG30184	0	NA	NA	NA	NA	NA
apt	0.596853	0.454699	3.988272	0.114009	0.909231	NA
Eglp1	0	NA	NA	NA	NA	NA
retn	0.44234	1.822487	4.025091	0.452781	0.650706	NA
St1	7.458983	1.161798	3.957142	0.293595	0.769067	NA
CG13560	0	NA	NA	NA	NA	NA
CG12491	0	NA	NA	NA	NA	NA
CG11300	0	NA	NA	NA	NA	NA
CG18128	0	NA	NA	NA	NA	NA
Eglp4	0	NA	NA	NA	NA	NA
Eglp3	0	NA	NA	NA	NA	NA
Eglp2	0	NA	NA	NA	NA	NA
Gr59e	0	NA	NA	NA	NA	NA
CG3906	0	NA	NA	NA	NA	NA
CG30177	0.283512	-0.2331	4.065094	-0.05734	0.954273	NA
CG5539	0.1047	0.613261	4.062799	0.150945	0.880019	NA
CG5597	0.4188	1.41068	4.034613	0.349644	0.726606	NA
CG17658	0	NA	NA	NA	NA	NA
CG13562	0	NA	NA	NA	NA	NA
CG2812	0	NA	NA	NA	NA	NA
fzr2	0.388212	0.094116	4.038892	0.023303	0.981409	NA
Fmo-1	0.178053	-1.16467	4.075067	-0.2858	0.775028	NA
CG30178	0	NA	NA	NA	NA	NA
Rsph4a	0	NA	NA	NA	NA	NA
CG4324	0.236778	-0.26946	4.071573	-0.06618	0.947234	NA
CG13566	0	NA	NA	NA	NA	NA
bgcn	0.819872	-0.85327	3.993249	-0.21368	0.830798	NA
shu	0	NA	NA	NA	NA	NA
CG4763	0	NA	NA	NA	NA	NA
CG4797	0	NA	NA	NA	NA	NA
CG15800	0	NA	NA	NA	NA	NA
CG13564	0.178053	-1.16467	4.075067	-0.2858	0.775028	NA
CG16786	3.320463	-2.57495	3.942954	-0.65305	0.513723	NA
snoRNA:Ps	0	NA	NA	NA	NA	NA
CG13575	5.387731	-1.69677	3.957058	-0.4288	0.668072	NA
slbo	0.178053	-1.16467	4.075067	-0.2858	0.775028	NA
CG13577	0	NA	NA	NA	NA	NA
Ir60a	0.356107	-1.47099	4.037764	-0.36431	0.715628	NA
CG10339	0.356107	-1.47099	4.037764	-0.36431	0.715628	NA
CG3483	0	NA	NA	NA	NA	NA
CG3492	15.22063	-3.95725	3.887164	-1.01803	0.308663	NA
CG3494	0	NA	NA	NA	NA	NA
CG16837	0.356107	-1.47099	4.037764	-0.36431	0.715628	NA
Crtp	0	NA	NA	NA	NA	NA
CG13581	0	NA	NA	NA	NA	NA
CG11413	0	NA	NA	NA	NA	NA
uri	42.21116	5.442916	3.832312	1.42027	0.155529	NA
CG13587	0	NA	NA	NA	NA	NA

Samtor	3.947251	1.355039	3.960575	0.342132	0.732252	NA
Adck1	47.76895	-0.36862	3.83539	-0.09611	0.923433	NA
Cyp9c1	0	NA	NA	NA	NA	NA
CG3640	0	NA	NA	NA	NA	NA
CG30161	0	NA	NA	NA	NA	NA
Cpr60D	57.3494	-0.33305	3.835187	-0.08684	0.930798	NA
CG4781	0.178053	-1.16467	4.075067	-0.2858	0.775028	NA
CG4741	4.290956	-1.83003	3.783302	-0.48371	0.628591	NA
CG4681	0.792466	-1.7823	3.993845	-0.44626	0.655408	NA
Ir60e	0	NA	NA	NA	NA	NA
Prosalpha4	0	NA	NA	NA	NA	NA
Ssl	0	NA	NA	NA	NA	NA
CG13590	0	NA	NA	NA	NA	NA
CG13589	0	NA	NA	NA	NA	NA
CG4563	16.75191	2.95528	3.92353	0.75322	0.451318	NA
Pgam5-2	0	NA	NA	NA	NA	NA
CG15873	0.1047	0.613261	4.062799	0.150945	0.880019	NA
spz6	26.03894	1.673283	3.952785	0.423318	0.672064	NA
CG3880	0	NA	NA	NA	NA	NA
CG3650	0.1047	0.613261	4.062799	0.150945	0.880019	NA
CG9083	0	NA	NA	NA	NA	NA
CG3770	0.3141	1.240997	4.03841	0.307299	0.758616	NA
CG3829	6.410969	-4.42549	3.886842	-1.13858	0.254878	NA
CG2736	0.69928	1.019866	4.009445	0.254366	0.799213	NA
Phk-3	0	NA	NA	NA	NA	NA
CG30427	8.865743	-2.22132	3.907426	-0.56849	0.569704	NA
Tpc2	47.08818	-1.05688	3.951468	-0.26746	0.789112	NA
Ance-5	0	NA	NA	NA	NA	NA
ETH	0	NA	NA	NA	NA	NA
Lcp9	0	NA	NA	NA	NA	NA
tRNA:Ala-(	0	NA	NA	NA	NA	NA
tRNA:Ala-(	0	NA	NA	NA	NA	NA
tRNA:Ala-(	3.729492	1.144349	3.963186	0.288745	0.772777	NA
CG30430	0	NA	NA	NA	NA	NA
CG30429	0	NA	NA	NA	NA	NA
Ebp	0	NA	NA	NA	NA	NA
EbpII	0	NA	NA	NA	NA	NA
gsb-n	23.87158	1.746033	3.953075	0.44169	0.658714	NA
Lsp1gamm	0.745683	-0.62945	3.95549	-0.15913	0.873564	NA
CG13875	7.844848	0.362684	3.753528	0.096625	0.923024	NA
CG13877	0	NA	NA	NA	NA	NA
CG32845	0.104623	0.639075	4.067674	0.157111	0.875158	NA
pyx	13.76551	-5.07164	3.90693	-1.29811	0.194248	NA
CG7049	8.506594	-2.88022	3.80753	-0.75645	0.449377	NA
CG12483	0	NA	NA	NA	NA	NA
Roc1b	0	NA	NA	NA	NA	NA
Ppm1	0	NA	NA	NA	NA	NA
Reg-2	45.15878	6.743671	3.874108	1.740703	0.081736	NA
CG13893	53.47464	-3.14867	3.791755	-0.8304	0.406313	NA
Kah	0.104623	0.639075	4.067674	0.157111	0.875158	NA

CG13891	3.140998	1.694072	3.966495	0.427096	0.66931	NA
CG13884	0	NA	NA	NA	NA	NA
tRNA:Ile-T/	0	NA	NA	NA	NA	NA
tRNA:Ile-T/	0	NA	NA	NA	NA	NA
Gr61a	0.132078	-1.13211	4.069918	-0.27817	0.780885	NA
cep290	0.1047	0.613261	4.062799	0.150945	0.880019	NA
mat	2.824828	1.495401	3.963153	0.377326	0.705931	NA
CG13898	0	NA	NA	NA	NA	NA
tRNA:iMet	0	NA	NA	NA	NA	NA
hng3	11.64196	-6.91318	3.912066	-1.76714	0.077204	NA
CG32483	0	NA	NA	NA	NA	NA
hiro	0.282753	-0.25398	4.020204	-0.06318	0.949626	NA
CG17129	0	NA	NA	NA	NA	NA
LysB	0	NA	NA	NA	NA	NA
LysC	0	NA	NA	NA	NA	NA
LysE	0	NA	NA	NA	NA	NA
LysS	0	NA	NA	NA	NA	NA
LysP	0.178053	-1.16467	4.075067	-0.2858	0.775028	NA
LysD	0	NA	NA	NA	NA	NA
LysX	0	NA	NA	NA	NA	NA
galene	0.178053	-1.16467	4.075067	-0.2858	0.775028	NA
CG9129	0.53416	-1.53481	4.012395	-0.38252	0.702078	NA
asRNA:CR4	0	NA	NA	NA	NA	NA
asRNA:CR4	1.908008	-0.10149	3.903479	-0.026	0.979257	NA
asRNA:CR4	0	NA	NA	NA	NA	NA
asRNA:CR4	0	NA	NA	NA	NA	NA
asRNA:CR4	0	NA	NA	NA	NA	NA
asRNA:CR4	0	NA	NA	NA	NA	NA
asRNA:CR4	0	NA	NA	NA	NA	NA
lncRNA:CR	0	NA	NA	NA	NA	NA
asRNA:CR4	0	NA	NA	NA	NA	NA
asRNA:CR4	0	NA	NA	NA	NA	NA
asRNA:CR4	0	NA	NA	NA	NA	NA
asRNA:CR4	17.58186	3.813812	3.886509	0.981295	0.326447	NA
snoRNA:C(	0	NA	NA	NA	NA	NA
snoRNA:C(	0	NA	NA	NA	NA	NA
snoRNA:C(	0	NA	NA	NA	NA	NA
snoRNA:De	1.837946	1.501715	3.941336	0.381017	0.703191	NA
snoRNA:ht	0	NA	NA	NA	NA	NA
snoRNA:ki:	8.542628	-1.66664	3.940348	-0.42297	0.672319	NA
snoRNA:lo	0.178053	-1.16467	4.075067	-0.2858	0.775028	NA
snoRNA:nc	0	NA	NA	NA	NA	NA
snoRNA:S(	0.837599	1.568055	4.001801	0.391837	0.695178	NA
snoRNA:S(	0.466186	0.959471	4.033986	0.237847	0.812	NA
snoRNA:Tu	1.598336	-0.35371	3.908817	-0.09049	0.927898	NA
scaRNA:M	0.62774	1.374224	3.999506	0.343599	0.731148	NA
snoRNA:2F	40.69346	2.757735	3.734206	0.738506	0.460207	NA
snoRNA:ab	0	NA	NA	NA	NA	NA
scaRNA:Ps	7.383689	-0.99146	3.937704	-0.25179	0.801206	NA
snoRNA:C(	0.565353	0.142845	3.959283	0.036079	0.97122	NA

snoRNA:C(	12.28568	-1.69873	3.954016	-0.42962	0.667472	NA
scaRNA:Ps	6.053811	-1.68915	3.957162	-0.42686	0.669482	NA
snoRNA:Ec	0	NA	NA	NA	NA	NA
scaRNA:Ps	0.178053	-1.16467	4.075067	-0.2858	0.775028	NA
snoRNA:Irl	7.704395	-3.34433	3.835324	-0.87198	0.383219	NA
snoRNA:lo	0.209247	1.042804	4.047837	0.25762	0.7967	NA
snoRNA:lo	0	NA	NA	NA	NA	NA
snoRNA:lo	0	NA	NA	NA	NA	NA
snoRNA:Pi	0.178053	-1.16467	4.075067	-0.2858	0.775028	NA
scaRNA:Ps	12.66832	0.37284	3.952392	0.094333	0.924845	NA
scaRNA:Ps	0	NA	NA	NA	NA	NA
scaRNA:Ps	8.755114	2.560058	3.774782	0.6782	0.497645	NA
snmRNA:C	27.09742	1.534593	3.952235	0.388285	0.697805	NA
lncRNA:CR	0.209247	1.042804	4.047837	0.25762	0.7967	NA
asRNA:CR4	37.93315	3.597634	3.708242	0.970172	0.331961	NA
CR43482	0	NA	NA	NA	NA	NA
CR43483	0	NA	NA	NA	NA	NA
lncRNA:CR	0	NA	NA	NA	NA	NA
lncRNA:CR	0	NA	NA	NA	NA	NA
lncRNA:CR	0	NA	NA	NA	NA	NA
lncRNA:CR	0	NA	NA	NA	NA	NA
lncRNA:CR	0	NA	NA	NA	NA	NA
lncRNA:CR	0	NA	NA	NA	NA	NA
lncRNA:CR	0	NA	NA	NA	NA	NA
lncRNA:CR	0	NA	NA	NA	NA	NA
lncRNA:CR	0	NA	NA	NA	NA	NA
lncRNA:CR	0	NA	NA	NA	NA	NA
Vsx1	43.62684	-0.08014	3.736845	-0.02145	0.982889	NA
Vsx2	1.675933	0.786673	3.895325	0.201953	0.839953	NA
lncRNA:CR	0	NA	NA	NA	NA	NA
lncRNA:CR	0	NA	NA	NA	NA	NA
asRNA:CR4	0	NA	NA	NA	NA	NA
lncRNA:CR	3.455098	1.699144	3.965156	0.428519	0.668273	NA
lncRNA:CR	0	NA	NA	NA	NA	NA
asRNA:CR4	0	NA	NA	NA	NA	NA
asRNA:CR4	0.236778	-0.26946	4.071573	-0.06618	0.947234	NA
lncRNA:CR	0	NA	NA	NA	NA	NA
CG43618	0	NA	NA	NA	NA	NA
Acp98AB	0	NA	NA	NA	NA	NA
lncRNA:CR	5.090129	2.804563	3.839498	0.73045	0.465115	NA
lncRNA:CR	0	NA	NA	NA	NA	NA
lncRNA:CR	0	NA	NA	NA	NA	NA
lncRNA:CR	20.17773	-0.27155	3.843595	-0.07065	0.943675	NA
lncRNA:CR	0	NA	NA	NA	NA	NA
lncRNA:CR	5.334983	-0.21854	3.8608	-0.05661	0.95486	NA
asRNA:AST	0	NA	NA	NA	NA	NA
CG43630	0	NA	NA	NA	NA	NA
lncRNA:CR	0	NA	NA	NA	NA	NA
lncRNA:CR	0.233093	0.692656	4.076246	0.169925	0.865069	NA
lncRNA:CR	0	NA	NA	NA	NA	NA

lncRNA:CR	0	NA	NA	NA	NA	NA
lncRNA:CR	53.738	4.435927	3.793943	1.169213	0.242318	NA
asRNA:CR4	0	NA	NA	NA	NA	NA
asRNA:CR4	3.661814	1.504942	3.960447	0.379993	0.703951	NA
CG43638	53.19492	1.199356	3.898806	0.307621	0.758371	NA
lncRNA:CR	0	NA	NA	NA	NA	NA
lncRNA:CR	0	NA	NA	NA	NA	NA
lncRNA:CR	0	NA	NA	NA	NA	NA
lncRNA:CR	21.34913	0.009894	3.73445	0.00265	0.997886	NA
lncRNA:CR	0.178053	-1.16467	4.075067	-0.2858	0.775028	NA
CG43645	0	NA	NA	NA	NA	NA
CG43646	0	NA	NA	NA	NA	NA
CG43647	0.2094	1.005034	4.045341	0.248442	0.803792	NA
lncRNA:CR	0	NA	NA	NA	NA	NA
lncRNA:CR	0	NA	NA	NA	NA	NA
lncRNA:CR	34.083	3.459114	3.880687	0.891366	0.372733	NA
lncRNA:CR	0	NA	NA	NA	NA	NA
lncRNA:CR	0	NA	NA	NA	NA	NA
lncRNA:CR	4.387809	-5.38802	3.922418	-1.37365	0.169551	NA
Myo10A	1.869329	-1.24083	3.858668	-0.32157	0.747779	NA
CG43659	0	NA	NA	NA	NA	NA
lncRNA:CR	3.034074	1.498215	3.962342	0.378114	0.705346	NA
CG43666	0	NA	NA	NA	NA	NA
CG43667	0.534919	-2.31746	4.015737	-0.5771	0.563875	NA
CG43668	0.178053	-1.16467	4.075067	-0.2858	0.775028	NA
CR43669	0.178053	-1.16467	4.075067	-0.2858	0.775028	NA
CR43671	0.388212	0.094116	4.038892	0.023303	0.981409	NA
CG43672	0	NA	NA	NA	NA	NA
CG43673	0	NA	NA	NA	NA	NA
CG43675	36.03047	0.629982	3.951482	0.159429	0.873331	NA
Su(var)3-9	51.79504	2.886521	3.919648	0.736423	0.461473	NA
Rpb4	0	NA	NA	NA	NA	NA
CG43677	0	NA	NA	NA	NA	NA
CG43678	0.1047	0.613261	4.062799	0.150945	0.880019	NA
CG43679	0	NA	NA	NA	NA	NA
CG43680	0	NA	NA	NA	NA	NA
lncRNA:CR	0.178053	-1.16467	4.075067	-0.2858	0.775028	NA
lncRNA:CR	0	NA	NA	NA	NA	NA
asRNA:CR4	0	NA	NA	NA	NA	NA
lncRNA:CR	0.104623	0.639075	4.067674	0.157111	0.875158	NA
lncRNA:CR	0	NA	NA	NA	NA	NA
CR43687	0	NA	NA	NA	NA	NA
CR43688	0.178053	-1.16467	4.075067	-0.2858	0.775028	NA
CG43691	17.66118	0.448765	3.858002	0.11632	0.907399	NA
CG43694	0.819113	-2.02578	3.984237	-0.50845	0.611138	NA
lncRNA:CR	0.998761	-0.71333	3.993823	-0.17861	0.858245	NA
Hmgcr	17.55278	-1.28219	3.861796	-0.33202	0.739875	NA
lncRNA:CR	3.263305	1.139496	3.96488	0.287397	0.773808	NA
CG34334	0	NA	NA	NA	NA	NA
Gen	0	NA	NA	NA	NA	NA

CR43696	0	NA	NA	NA	NA	NA
CR43697	0	NA	NA	NA	NA	NA
lncRNA:CR	0	NA	NA	NA	NA	NA
snmRNA:C	0	NA	NA	NA	NA	NA
CG43702	0	NA	NA	NA	NA	NA
CG43703	0	NA	NA	NA	NA	NA
lncRNA:CR	0	NA	NA	NA	NA	NA
lncRNA:CR	0	NA	NA	NA	NA	NA
lncRNA:CR	0	NA	NA	NA	NA	NA
snRNA:LU	0	NA	NA	NA	NA	NA
CG43709	0.387453	0.098632	4.002797	0.024641	0.980342	NA
CG43710	0.178812	-1.08808	4.061735	-0.26789	0.788788	NA
CG43711	0	NA	NA	NA	NA	NA
snoRNA:CI	0	NA	NA	NA	NA	NA
BubR1	6.231865	-1.68968	3.956986	-0.42701	0.66937	NA
lncRNA:CR	1.246373	-1.6238	3.979526	-0.40804	0.683245	NA
lncRNA:CR	0	NA	NA	NA	NA	NA
lncRNA:CR	0.178053	-1.16467	4.075067	-0.2858	0.775028	NA
lncRNA:CR	8.899494	1.731975	3.956638	0.437739	0.661575	NA
lncRNA:CR	0	NA	NA	NA	NA	NA
lncRNA:CR	0	NA	NA	NA	NA	NA
lncRNA:CR	0	NA	NA	NA	NA	NA
lncRNA:CR	0	NA	NA	NA	NA	NA
asRNA:CR4	0	NA	NA	NA	NA	NA
CG43725	0	NA	NA	NA	NA	NA
qin	3.601651	2.267006	3.928796	0.577023	0.563924	NA
CR43727	0.357625	-1.47707	4.044794	-0.36518	0.714979	NA
asRNA:CR4	0	NA	NA	NA	NA	NA
asRNA:CR4	40.69198	0.952196	3.745205	0.254244	0.799307	NA
CG43731	0	NA	NA	NA	NA	NA
CG43732	0	NA	NA	NA	NA	NA
CR43734	0	NA	NA	NA	NA	NA
cd	0.87953	0.922254	3.965066	0.232595	0.816076	NA
Dic61B	1.073687	0.81765	3.97163	0.205873	0.83689	NA
asRNA:CR4	0	NA	NA	NA	NA	NA
CG43739	0	NA	NA	NA	NA	NA
Hmx	10.73333	0.200181	3.952912	0.050641	0.959611	NA
Cnx14D	23.18491	1.631184	3.735382	0.436685	0.66234	NA
CG43750	0	NA	NA	NA	NA	NA
lncRNA:CR	0	NA	NA	NA	NA	NA
lncRNA:CR	0.4188	1.41068	4.034613	0.349644	0.726606	NA
Send2	0.1047	0.613261	4.062799	0.150945	0.880019	NA
CG43760	0	NA	NA	NA	NA	NA
lncRNA:CR	0	NA	NA	NA	NA	NA
lncRNA:CR	0	NA	NA	NA	NA	NA
lncRNA:CR	0	NA	NA	NA	NA	NA
lncRNA:CR	4.290474	4.790691	3.933964	1.217777	0.223309	NA
lncRNA:CR	0	NA	NA	NA	NA	NA
asRNA:CR4	2.848852	-1.66884	3.963956	-0.421	0.673753	NA
lncRNA:CR	0	NA	NA	NA	NA	NA

lncRNA:CR	0	NA	NA	NA	NA	NA
lncRNA:CR	0.178053	-1.16467	4.075067	-0.2858	0.775028	NA
mwh	5.069045	2.094511	3.864031	0.542053	0.587782	NA
Det	10.63722	-3.13029	3.912376	-0.8001	0.423653	NA
CG43773	0	NA	NA	NA	NA	NA
CG43774	0	NA	NA	NA	NA	NA
CG43775	0	NA	NA	NA	NA	NA
CG43776	0	NA	NA	NA	NA	NA
CG43777	0	NA	NA	NA	NA	NA
CG43779	0	NA	NA	NA	NA	NA
wrm1	0	NA	NA	NA	NA	NA
wrm2	0	NA	NA	NA	NA	NA
CG43784	0	NA	NA	NA	NA	NA
lncRNA:CR	0	NA	NA	NA	NA	NA
PolE1	5.859281	2.931745	3.871197	0.757323	0.448857	NA
asRNA:CR4	0	NA	NA	NA	NA	NA
lncRNA:CR	0	NA	NA	NA	NA	NA
lncRNA:CR	0	NA	NA	NA	NA	NA
lncRNA:CR	0	NA	NA	NA	NA	NA
CG43796	0	NA	NA	NA	NA	NA
CG43797	0	NA	NA	NA	NA	NA
CG43798	0	NA	NA	NA	NA	NA
CG43799	0.178053	-1.16467	4.075067	-0.2858	0.775028	NA
CG43800	35.35792	-0.53866	3.835721	-0.14043	0.888318	NA
lncRNA:CR	14.76269	1.7408	3.954394	0.440219	0.659778	NA
lncRNA:CR	0	NA	NA	NA	NA	NA
lncRNA:CR	51.99156	-1.706	3.951646	-0.43172	0.665945	NA
lncRNA:CR	0	NA	NA	NA	NA	NA
asRNA:CR4	18.67304	2.998883	3.828897	0.783224	0.433496	NA
lncRNA:CR	0	NA	NA	NA	NA	NA
lncRNA:CR	0	NA	NA	NA	NA	NA
asRNA:CR4	0.104623	0.639075	4.067674	0.157111	0.875158	NA
lncRNA:CR	0	NA	NA	NA	NA	NA
lncRNA:CR	0	NA	NA	NA	NA	NA
asRNA:CR4	0	NA	NA	NA	NA	NA
CG43814	0	NA	NA	NA	NA	NA
CG43815	0	NA	NA	NA	NA	NA
CG43816	0	NA	NA	NA	NA	NA
lncRNA:CR	0.466186	0.959471	4.033986	0.237847	0.812	NA
lncRNA:CR	1.283782	0.252057	3.902549	0.064588	0.948502	NA
lncRNA:CR	0	NA	NA	NA	NA	NA
asRNA:CR4	0	NA	NA	NA	NA	NA
lncRNA:CR	0	NA	NA	NA	NA	NA
lncRNA:CR	0.178053	-1.16467	4.075067	-0.2858	0.775028	NA
asRNA:CR4	0	NA	NA	NA	NA	NA
lncRNA:CR	15.56811	6.714909	3.911652	1.716643	0.086044	NA
lncRNA:CR	0	NA	NA	NA	NA	NA
lncRNA:CR	0	NA	NA	NA	NA	NA
CG43829	0	NA	NA	NA	NA	NA
lncRNA:CR	0	NA	NA	NA	NA	NA

lncRNA:CR	0	NA	NA	NA	NA	NA
lncRNA:CR	0	NA	NA	NA	NA	NA
asRNA:CR4	0	NA	NA	NA	NA	NA
lncRNA:CR	0	NA	NA	NA	NA	NA
lncRNA:CR	0	NA	NA	NA	NA	NA
lncRNA:CR	22.40578	1.745474	3.953216	0.441533	0.658827	NA
lncRNA:CR	0	NA	NA	NA	NA	NA
lncRNA:CR	0	NA	NA	NA	NA	NA
CG43841	0	NA	NA	NA	NA	NA
asRNA:CR4	0	NA	NA	NA	NA	NA
CG43844	0	NA	NA	NA	NA	NA
lncRNA:CR	0	NA	NA	NA	NA	NA
lncRNA:CR	0.2094	1.005034	4.045341	0.248442	0.803792	NA
lncRNA:CR	0	NA	NA	NA	NA	NA
lncRNA:CR	0.104623	0.639075	4.067674	0.157111	0.875158	NA
CG43851	0	NA	NA	NA	NA	NA
lncRNA:CR	0	NA	NA	NA	NA	NA
lncRNA:CR	0.178053	-1.16467	4.075067	-0.2858	0.775028	NA
lncRNA:CR	0	NA	NA	NA	NA	NA
lncRNA:CR	29.81161	0.220283	3.866794	0.056968	0.954571	NA
lncRNA:CR	0	NA	NA	NA	NA	NA
lncRNA:CR	0.1047	0.613261	4.062799	0.150945	0.880019	NA
lncRNA:CR	0	NA	NA	NA	NA	NA
lncRNA:CR	0	NA	NA	NA	NA	NA
asRNA:CR4	0	NA	NA	NA	NA	NA
asRNA:CR4	0	NA	NA	NA	NA	NA
asRNA:CR4	0	NA	NA	NA	NA	NA
asRNA:CR4	0	NA	NA	NA	NA	NA
asRNA:CR4	0	NA	NA	NA	NA	NA
lncRNA:CR	5.697705	-1.68799	3.957546	-0.42652	0.669726	NA
asRNA:CR4	0	NA	NA	NA	NA	NA
asRNA:CR4	0	NA	NA	NA	NA	NA
lncRNA:CR	0	NA	NA	NA	NA	NA
CG43880	0	NA	NA	NA	NA	NA
lncRNA:CR	0	NA	NA	NA	NA	NA
lncRNA:CR	0	NA	NA	NA	NA	NA
lncRNA:CR	6.072596	1.721931	3.959216	0.434917	0.663622	NA
lncRNA:CR	0	NA	NA	NA	NA	NA
asRNA:CR4	0	NA	NA	NA	NA	NA
CG43886	6.587971	-1.69066	3.956663	-0.42729	0.669165	NA
lncRNA:CR	32.6204	0.432143	3.83534	0.112674	0.910289	NA
lncRNA:CR	0	NA	NA	NA	NA	NA
lncRNA:CR	0.178812	-1.08808	4.061735	-0.26789	0.788788	NA
asRNA:CR4	0	NA	NA	NA	NA	NA
lncRNA:CR	0	NA	NA	NA	NA	NA
lncRNA:CR	0	NA	NA	NA	NA	NA
CG43894	0.387453	0.098632	4.002797	0.024641	0.980342	NA
CG43895	0	NA	NA	NA	NA	NA
CG43896	0.283436	-0.20033	4.070792	-0.04921	0.960751	NA
CG43897	22.64086	0.516917	3.880485	0.133209	0.894028	NA

asRNA:CR4	54.18659	0.11364	3.378239	0.033639	0.973165	NA
lncRNA:CR	16.54259	1.742266	3.954024	0.440631	0.65948	NA
asRNA:CR4	0.178053	-1.16467	4.075067	-0.2858	0.775028	NA
lncRNA:CR	10.78858	-2.22416	3.890836	-0.57164	0.567566	NA
lncRNA:CR	0.357625	-1.47707	4.044794	-0.36518	0.714979	NA
asRNA:CR4	0.660388	-1.75977	4.001266	-0.4398	0.660079	NA
lncRNA:CR	0	NA	NA	NA	NA	NA
asRNA:CR4	0	NA	NA	NA	NA	NA
lncRNA:CR	23.30932	1.174184	3.952924	0.297042	0.766434	NA
lncRNA:CR	0.1047	0.613261	4.062799	0.150945	0.880019	NA
lncRNA:CR	0	NA	NA	NA	NA	NA
lncRNA:CR	0	NA	NA	NA	NA	NA
CG43915	1.176056	-2.16791	3.961611	-0.54723	0.584222	NA
asRNA:CR4	18.67427	-1.27098	3.952487	-0.32157	0.747782	NA
lncRNA:CR	0	NA	NA	NA	NA	NA
lncRNA:CR	0.466186	0.959471	4.033986	0.237847	0.812	NA
CG43919	50.3431	-3.16839	3.905461	-0.81127	0.41721	NA
CG43920	0	NA	NA	NA	NA	NA
CG43922	0.104623	0.639075	4.067674	0.157111	0.875158	NA
CG43923	0	NA	NA	NA	NA	NA
CG43924	0	NA	NA	NA	NA	NA
CG43925	5.636447	-2.58102	3.841249	-0.67192	0.501634	NA
asRNA:CR4	0	NA	NA	NA	NA	NA
lncRNA:CR	0	NA	NA	NA	NA	NA
lncRNA:CR	0	NA	NA	NA	NA	NA
lncRNA:CR	0.894061	-2.00006	4.030294	-0.49626	0.619714	NA
lncRNA:CR	0.209247	1.042804	4.047837	0.25762	0.7967	NA
CG43931	0	NA	NA	NA	NA	NA
lncRNA:CR	0.132078	-1.13211	4.069918	-0.27817	0.780885	NA
asRNA:CR4	1.787364	-3.08936	3.999387	-0.77246	0.439844	NA
CG43935	0.178053	-1.16467	4.075067	-0.2858	0.775028	NA
asRNA:CR4	2.617498	1.683176	3.969392	0.424039	0.671538	NA
lncRNA:CR	0.104623	0.639075	4.067674	0.157111	0.875158	NA
CG43938	0	NA	NA	NA	NA	NA
lncRNA:CR	0.787655	0.325119	3.927268	0.082785	0.934022	NA
lncRNA:CR	0.178053	-1.16467	4.075067	-0.2858	0.775028	NA
lncRNA:CR	0.411147	-0.29058	4.046973	-0.0718	0.94276	NA
lncRNA:CR	0.1047	0.613261	4.062799	0.150945	0.880019	NA
asRNA:CR4	0.605633	0.047977	3.956223	0.012127	0.990324	NA
lncRNA:CR	0	NA	NA	NA	NA	NA
asRNA:CR4	0	NA	NA	NA	NA	NA
lncRNA:CR	0	NA	NA	NA	NA	NA
lncRNA:CR	0	NA	NA	NA	NA	NA
lncRNA:CR	0	NA	NA	NA	NA	NA
lncRNA:CR	0	NA	NA	NA	NA	NA
CG43954	0	NA	NA	NA	NA	NA
lncRNA:CR	9.119426	-2.24191	3.961111	-0.56598	0.571408	NA
lncRNA:CR	0	NA	NA	NA	NA	NA
lncRNA:CR	52.44935	-8.45153	3.846053	-2.19745	0.027988	NA
asRNA:CR4	0	NA	NA	NA	NA	NA

Mhc	1.182007	-0.53267	3.905671	-0.13638	0.891517	NA
CG43965	0	NA	NA	NA	NA	NA
lncRNA:CR	25.11472	7.247321	3.912065	1.852556	0.063946	NA
lncRNA:CR	0	NA	NA	NA	NA	NA
CG43968	1.185692	-1.26854	3.910876	-0.32436	0.745664	NA
lncRNA:CR	0	NA	NA	NA	NA	NA
asRNA:CR4	0.356107	-1.47099	4.037764	-0.36431	0.715628	NA
CR43975	45.79152	-8.64584	3.908436	-2.2121	0.02696	NA
lncRNA:CR	0	NA	NA	NA	NA	NA
lncRNA:CR	0.356107	-1.47099	4.037764	-0.36431	0.715628	NA
asRNA:CR4	0	NA	NA	NA	NA	NA
CG43980	0.666997	-3.07779	3.974176	-0.77445	0.438666	NA
asRNA:CR4	0	NA	NA	NA	NA	NA
CG43982	0	NA	NA	NA	NA	NA
lncRNA:CR	0	NA	NA	NA	NA	NA
lncRNA:CR	0	NA	NA	NA	NA	NA
lncRNA:CR	0.178812	-1.08808	4.061735	-0.26789	0.788788	NA
lncRNA:CR	0	NA	NA	NA	NA	NA
lncRNA:CR	53.60629	2.975255	3.919042	0.759179	0.447745	NA
lncRNA:CR	0	NA	NA	NA	NA	NA
lncRNA:CR	0	NA	NA	NA	NA	NA
lncRNA:CR	0	NA	NA	NA	NA	NA
CG43993	2.670799	-1.66648	3.964795	-0.42032	0.674251	NA
lncRNA:CR	17.48199	1.17221	3.953592	0.296492	0.766854	NA
CG43996	7.957118	2.925265	3.930435	0.74426	0.456719	NA
CG43997	0	NA	NA	NA	NA	NA
CG43998	0	NA	NA	NA	NA	NA
CG43999	0	NA	NA	NA	NA	NA
CG44000	0	NA	NA	NA	NA	NA
CG44001	0	NA	NA	NA	NA	NA
CR44003	4.083297	1.7071	3.963068	0.430752	0.666649	NA
CG44004	0.264155	-1.54099	4.048976	-0.38059	0.703509	NA
CG44005	16.42585	1.531457	3.953091	0.387407	0.698455	NA
CG44006	1.361099	1.628413	3.98439	0.408698	0.682761	NA
CG44013	0.178812	-1.08808	4.061735	-0.26789	0.788788	NA
CG44014	0	NA	NA	NA	NA	NA
lncRNA:CR	0	NA	NA	NA	NA	NA
asRNA:CR4	0	NA	NA	NA	NA	NA
asRNA:CR4	0	NA	NA	NA	NA	NA
lncRNA:CR	12.0336	1.713627	3.901155	0.439261	0.660472	NA
Pburs	0	NA	NA	NA	NA	NA
lncRNA:CR	0	NA	NA	NA	NA	NA
CR44028	0	NA	NA	NA	NA	NA
lncRNA:CR	0	NA	NA	NA	NA	NA
lncRNA:CR	5.246801	1.5796	3.7704	0.418948	0.675254	NA
lncRNA:CR	0	NA	NA	NA	NA	NA
asRNA:CR4	0.178053	-1.16467	4.075067	-0.2858	0.775028	NA
lncRNA:CR	0	NA	NA	NA	NA	NA
CG44037	0.1047	0.613261	4.062799	0.150945	0.880019	NA
CG44038	0	NA	NA	NA	NA	NA

lncRNA:CR	0	NA	NA	NA	NA	NA
CG44040	0.178053	-1.16467	4.075067	-0.2858	0.775028	NA
lncRNA:CR	0	NA	NA	NA	NA	NA
lncRNA:CR	0.519377	-0.84038	3.994824	-0.21037	0.833381	NA
CG44044	8.391356	1.163791	3.956459	0.29415	0.768644	NA
asRNA:CR4	0	NA	NA	NA	NA	NA
asRNA:CR4	1.539076	1.809495	3.954298	0.457602	0.647238	NA
asRNA:CR4	12.35459	1.738163	3.955063	0.439478	0.660315	NA
lncRNA:CR	0.1047	0.613261	4.062799	0.150945	0.880019	NA
asRNA:CR4	0	NA	NA	NA	NA	NA
lncRNA:CR	4.296714	1.161077	3.83414	0.302826	0.762023	NA
lncRNA:CR	18.64746	1.172702	3.953425	0.296629	0.76675	NA
asRNA:CR4	0	NA	NA	NA	NA	NA
Tep5Psi	0	NA	NA	NA	NA	NA
lncRNA:iab	0.282753	-0.25398	4.020204	-0.06318	0.949626	NA
asRNA:CR4	0	NA	NA	NA	NA	NA
asRNA:CR4	22.09455	-6.28991	3.881714	-1.6204	0.105147	NA
lncRNA:CR	0	NA	NA	NA	NA	NA
lncRNA:CR	0	NA	NA	NA	NA	NA
lncRNA:CR	0.104623	0.639075	4.067674	0.157111	0.875158	NA
lncRNA:CR	0	NA	NA	NA	NA	NA
lncRNA:CR	0	NA	NA	NA	NA	NA
lncRNA:CR	0	NA	NA	NA	NA	NA
asRNA:CR4	1.012366	-0.44098	3.915726	-0.11262	0.910333	NA
asRNA:CR4	0	NA	NA	NA	NA	NA
asRNA:CR4	0	NA	NA	NA	NA	NA
asRNA:CR4	49.25079	-2.0781	3.839737	-0.54121	0.588364	NA
lncRNA:CR	0	NA	NA	NA	NA	NA
asRNA:CR4	0.178053	-1.16467	4.075067	-0.2858	0.775028	NA
lncRNA:CR	0	NA	NA	NA	NA	NA
lncRNA:CR	0	NA	NA	NA	NA	NA
asRNA:CR4	5.601706	-0.71134	3.846202	-0.18495	0.853272	NA
lncRNA:CR	14.70171	4.588677	3.790777	1.210485	0.226093	NA
lncRNA:CR	30.62342	0.645379	3.920576	0.164613	0.869248	NA
lncRNA:CR	61.46341	-7.3543	3.810193	-1.93017	0.053586	NA
lncRNA:CR	0.178053	-1.16467	4.075067	-0.2858	0.775028	NA
lncRNA:CR	5.018952	-1.89674	3.958511	-0.47915	0.631829	NA
lncRNA:CR	14.8957	-2.05089	3.95628	-0.51839	0.604187	NA
CR44079	0	NA	NA	NA	NA	NA
lncRNA:CR	0.31089	-1.9003	4.033427	-0.47114	0.637542	NA
lncRNA:CR	0	NA	NA	NA	NA	NA
lncRNA:CR	0.660388	-1.75977	4.001266	-0.4398	0.660079	NA
asRNA:CR4	13.60102	1.529869	3.953541	0.386962	0.698785	NA
lncRNA:CR	0	NA	NA	NA	NA	NA
asRNA:CR4	0	NA	NA	NA	NA	NA
lncRNA:CR	0	NA	NA	NA	NA	NA
CG44090	0	NA	NA	NA	NA	NA
lncRNA:CR	0	NA	NA	NA	NA	NA
asRNA:CR4	35.80241	-0.76211	3.835757	-0.19868	0.842509	NA
CG44094	0	NA	NA	NA	NA	NA

asRNA:CR4	25.6553	-0.01617	3.838211	-0.00421	0.996639	NA
lncRNA:CR	0	NA	NA	NA	NA	NA
lncRNA:CR	0	NA	NA	NA	NA	NA
lncRNA:CR	0	NA	NA	NA	NA	NA
CG44102	27.00065	-2.26818	3.954453	-0.57358	0.566255	NA
lncRNA:CR	0	NA	NA	NA	NA	NA
lncRNA:CR	0	NA	NA	NA	NA	NA
asRNA:CR4	0	NA	NA	NA	NA	NA
asRNA:CR4	0.906754	-0.02643	3.908482	-0.00676	0.994604	NA
asRNA:CR4	0	NA	NA	NA	NA	NA
lncRNA:CR	56.07563	-1.89572	3.833098	-0.49457	0.620907	NA
lncRNA:CR	0	NA	NA	NA	NA	NA
lncRNA:CR	16.56183	0.395184	3.841278	0.102878	0.91806	NA
lncRNA:CR	0	NA	NA	NA	NA	NA
lncRNA:CR	0	NA	NA	NA	NA	NA
asRNA:CR4	0	NA	NA	NA	NA	NA
asRNA:CR4	0	NA	NA	NA	NA	NA
asRNA:CR4	11.09014	2.752213	3.921894	0.701756	0.482831	NA
lncRNA:CR	0	NA	NA	NA	NA	NA
lncRNA:CR	0	NA	NA	NA	NA	NA
asRNA:CR4	10.53854	-5.91314	3.921221	-1.50799	0.131558	NA
lncRNA:CR	0	NA	NA	NA	NA	NA
lncRNA:CR	0	NA	NA	NA	NA	NA
fd3F	0.356107	-1.47099	4.037764	-0.36431	0.715628	NA
asRNA:CR4	0	NA	NA	NA	NA	NA
lncRNA:CR	0	NA	NA	NA	NA	NA
lncRNA:CR	0	NA	NA	NA	NA	NA
lncRNA:CR	46.09172	-3.63267	3.81368	-0.95254	0.340824	NA
asRNA:CR4	0	NA	NA	NA	NA	NA
CG4267	0	NA	NA	NA	NA	NA
lncRNA:CR	0	NA	NA	NA	NA	NA
lncRNA:CR	0	NA	NA	NA	NA	NA
asRNA:CR4	0	NA	NA	NA	NA	NA
lncRNA:CR	6.437242	-2.22638	3.96512	-0.56149	0.574463	NA
lncRNA:CR	0	NA	NA	NA	NA	NA
CG44139	0.712213	-1.57119	3.998613	-0.39293	0.694369	NA
CG44140	0	NA	NA	NA	NA	NA
CG44141	0	NA	NA	NA	NA	NA
CG44142	0	NA	NA	NA	NA	NA
lncRNA:CR	0	NA	NA	NA	NA	NA
asRNA:CR4	0	NA	NA	NA	NA	NA
lncRNA:CR	0.1047	0.613261	4.062799	0.150945	0.880019	NA
lncRNA:CR	0	NA	NA	NA	NA	NA
asRNA:CR4	0	NA	NA	NA	NA	NA
lncRNA:CR	0	NA	NA	NA	NA	NA
lncRNA:CR	27.208	-1.91809	3.952339	-0.48531	0.62746	NA
lncRNA:CR	30.33163	1.684094	3.952533	0.42608	0.67005	NA
lncRNA:CR	56.77181	1.702996	3.810244	0.446952	0.65491	NA
ppk18	0.178812	-1.08808	4.061735	-0.26789	0.788788	NA
Np	0.178053	-1.16467	4.075067	-0.2858	0.775028	NA

lncRNA:CR	0.209247	1.042804	4.047837	0.25762	0.7967	NA
asRNA:CR4	0	NA	NA	NA	NA	NA
lncRNA:CR	0	NA	NA	NA	NA	NA
lncRNA:CR	55.96105	-0.31099	3.834692	-0.0811	0.935363	NA
Irk1	0.233093	0.692656	4.076246	0.169925	0.865069	NA
asRNA:CR4	0	NA	NA	NA	NA	NA
CG44163	0	NA	NA	NA	NA	NA
FDY	50.10731	-0.07241	3.912127	-0.01851	0.985233	NA
lncRNA:CR	54.85822	-3.52234	3.899253	-0.90334	0.366347	NA
lncRNA:CR	0	NA	NA	NA	NA	NA
asRNA:CR4	0	NA	NA	NA	NA	NA
asRNA:CR4	0	NA	NA	NA	NA	NA
lncRNA:CR	0	NA	NA	NA	NA	NA
lncRNA:CR	12.35394	1.168965	3.954696	0.295589	0.767544	NA
lncRNA:CR	0	NA	NA	NA	NA	NA
lncRNA:CR	0	NA	NA	NA	NA	NA
CG44174	0	NA	NA	NA	NA	NA
lncRNA:CR	0	NA	NA	NA	NA	NA
asRNA:CR4	0	NA	NA	NA	NA	NA
lncRNA:CR	3.245698	1.695863	3.966021	0.427598	0.668944	NA
asRNA:CR4	36.54536	0.972774	3.836399	0.253564	0.799832	NA
lncRNA:CR	0	NA	NA	NA	NA	NA
asRNA:CR4	0	NA	NA	NA	NA	NA
asRNA:CR4	0	NA	NA	NA	NA	NA
lncRNA:CR	0	NA	NA	NA	NA	NA
lncRNA:CR	0	NA	NA	NA	NA	NA
lncRNA:CR	58.04537	-1.70624	3.951569	-0.43179	0.665895	NA
lncRNA:CR	0.178053	-1.16467	4.075067	-0.2858	0.775028	NA
lncRNA:CR	0	NA	NA	NA	NA	NA
lncRNA:CR	0	NA	NA	NA	NA	NA
lncRNA:CR	0	NA	NA	NA	NA	NA
asRNA:CR4	0.132078	-1.13211	4.069918	-0.27817	0.780885	NA
CG44194	0	NA	NA	NA	NA	NA
lncRNA:CR	0	NA	NA	NA	NA	NA
lncRNA:CR	0	NA	NA	NA	NA	NA
CG44198	0	NA	NA	NA	NA	NA
lncRNA:CR	0	NA	NA	NA	NA	NA
eIF4E3	0.209247	1.042804	4.047837	0.25762	0.7967	NA
lncRNA:CR	0	NA	NA	NA	NA	NA
asRNA:CR4	0.715249	-1.88064	4.034336	-0.46616	0.641102	NA
lncRNA:CR	0.178053	-1.16467	4.075067	-0.2858	0.775028	NA
lncRNA:CR	1.050006	0.081601	3.967032	0.02057	0.983589	NA
lncRNA:CR	0	NA	NA	NA	NA	NA
Oseg1	56.67021	1.966039	3.772312	0.521176	0.602244	NA
asRNA:CR4	0	NA	NA	NA	NA	NA
asRNA:CR4	0.1047	0.613261	4.062799	0.150945	0.880019	NA
lncRNA:CR	0	NA	NA	NA	NA	NA
Gr22c	0	NA	NA	NA	NA	NA
Gr2a	0.2094	1.005034	4.045341	0.248442	0.803792	NA
Ir47b	28.08548	-3.92041	3.842805	-1.0202	0.307636	NA

Ir56e	0	NA	NA	NA	NA	NA
lncRNA:CR	0	NA	NA	NA	NA	NA
lncRNA:CR	0	NA	NA	NA	NA	NA
lncRNA:CR	0	NA	NA	NA	NA	NA
CG44215	0	NA	NA	NA	NA	NA
lncRNA:CR	0.104623	0.639075	4.067674	0.157111	0.875158	NA
lncRNA:CR	0	NA	NA	NA	NA	NA
lncRNA:CR	0	NA	NA	NA	NA	NA
lncRNA:CR	19.58586	-1.70224	3.952863	-0.43064	0.666733	NA
lncRNA:CR	28.59406	0.113972	3.951527	0.028843	0.97699	NA
lncRNA:CR	6.491396	1.723946	3.958697	0.435483	0.663212	NA
Ir60f	0	NA	NA	NA	NA	NA
asRNA:CR4	0	NA	NA	NA	NA	NA
lncRNA:CR	0	NA	NA	NA	NA	NA
lncRNA:CR	0	NA	NA	NA	NA	NA
CG44227	0	NA	NA	NA	NA	NA
asRNA:CR4	0	NA	NA	NA	NA	NA
lncRNA:CR	0	NA	NA	NA	NA	NA
lncRNA:CR	0	NA	NA	NA	NA	NA
lncRNA:CR	0	NA	NA	NA	NA	NA
asRNA:CR4	0	NA	NA	NA	NA	NA
lncRNA:CR	48.07628	-1.92027	3.951719	-0.48593	0.627015	NA
asRNA:CR4	0.178053	-1.16467	4.075067	-0.2858	0.775028	NA
asRNA:CR4	0	NA	NA	NA	NA	NA
lncRNA:CR	0	NA	NA	NA	NA	NA
CG44243	0.1047	0.613261	4.062799	0.150945	0.880019	NA
CG44249	0	NA	NA	NA	NA	NA
CG44250	0	NA	NA	NA	NA	NA
CG44251	0	NA	NA	NA	NA	NA
Fatp2	16.75199	1.742418	3.953985	0.440674	0.659449	NA
CG44253	0	NA	NA	NA	NA	NA
sud1	13.45938	-2.22969	3.889841	-0.57321	0.566503	NA
lncRNA:CR	0	NA	NA	NA	NA	NA
CG44257	60.53811	-1.70633	3.951542	-0.43181	0.665877	NA
lncRNA:CR	0	NA	NA	NA	NA	NA
CG44259	0.528311	-1.72839	4.011871	-0.43082	0.6666	NA
lncRNA:CR	0	NA	NA	NA	NA	NA
CG44261	0	NA	NA	NA	NA	NA
lncRNA:CR	0	NA	NA	NA	NA	NA
lncRNA:CR	0	NA	NA	NA	NA	NA
lncRNA:CR	0	NA	NA	NA	NA	NA
lncRNA:CR	45.63199	1.147331	3.951921	0.290322	0.77157	NA
asRNA:CR4	0	NA	NA	NA	NA	NA
asRNA:CR4	0	NA	NA	NA	NA	NA
lncRNA:CR	56.96877	4.991552	3.805778	1.311572	0.189665	NA
lncRNA:CR	3.026728	2.697288	3.902197	0.691223	0.489426	NA
CG44270	0	NA	NA	NA	NA	NA
CG44271	0	NA	NA	NA	NA	NA
CG42688	0	NA	NA	NA	NA	NA
CG17097	0	NA	NA	NA	NA	NA

CG32727	0	NA	NA	NA	NA	NA
CG13639	0	NA	NA	NA	NA	NA
CG18258	0	NA	NA	NA	NA	NA
CG18234	0	NA	NA	NA	NA	NA
CG15425	0	NA	NA	NA	NA	NA
lncRNA:CR	0.178053	-1.16467	4.075067	-0.2858	0.775028	NA
CG14717	39.02448	1.53608	3.95183	0.388701	0.697497	NA
asRNA:CR4	0	NA	NA	NA	NA	NA
lncRNA:CR	0	NA	NA	NA	NA	NA
lncRNA:CR	0	NA	NA	NA	NA	NA
lncRNA:CR	0	NA	NA	NA	NA	NA
asRNA:CR4	0	NA	NA	NA	NA	NA
lncRNA:CR	0	NA	NA	NA	NA	NA
lncRNA:CR	0	NA	NA	NA	NA	NA
lncRNA:CR	0	NA	NA	NA	NA	NA
lncRNA:CR	0	NA	NA	NA	NA	NA
lncRNA:CR	0.178053	-1.16467	4.075067	-0.2858	0.775028	NA
lncRNA:CR	0	NA	NA	NA	NA	NA
lncRNA:CR	0	NA	NA	NA	NA	NA
lncRNA:CR	0	NA	NA	NA	NA	NA
lncRNA:CR	0.5235	1.490714	4.02555	0.370313	0.711149	NA
lncRNA:CR	0	NA	NA	NA	NA	NA
CG44286	0	NA	NA	NA	NA	NA
asRNA:CR4	0	NA	NA	NA	NA	NA
lncRNA:CR	0	NA	NA	NA	NA	NA
lncRNA:CR	0	NA	NA	NA	NA	NA
CG44290	0	NA	NA	NA	NA	NA
asRNA:CR4	50.06972	9.06589	3.907623	2.320052	0.020338	NA
lncRNA:CR	0	NA	NA	NA	NA	NA
asRNA:CR4	0	NA	NA	NA	NA	NA
CG44296	0.132078	-1.13211	4.069918	-0.27817	0.780885	NA
asRNA:CR4	0	NA	NA	NA	NA	NA
lncRNA:CR	0.53416	-1.53481	4.012395	-0.38252	0.702078	NA
lncRNA:CR	0.178812	-1.08808	4.061735	-0.26789	0.788788	NA
lncRNA:CR	0.1047	0.613261	4.062799	0.150945	0.880019	NA
lncRNA:CR	26.73231	0.108549	3.837492	0.028287	0.977434	NA
Sfp33A4	0.178053	-1.16467	4.075067	-0.2858	0.775028	NA
CG44303	0	NA	NA	NA	NA	NA
asRNA:CR4	0	NA	NA	NA	NA	NA
lncRNA:CR	0	NA	NA	NA	NA	NA
lncRNA:CR	0.233093	0.692656	4.076246	0.169925	0.865069	NA
lncRNA:CR	0	NA	NA	NA	NA	NA
lncRNA:CR	0	NA	NA	NA	NA	NA
lncRNA:CR	0	NA	NA	NA	NA	NA
lncRNA:CR	0	NA	NA	NA	NA	NA
lncRNA:CR	0	NA	NA	NA	NA	NA
lncRNA:CR	0	NA	NA	NA	NA	NA
CG44314	0.5235	1.490714	4.02555	0.370313	0.711149	NA
lncRNA:CR	0	NA	NA	NA	NA	NA
lncRNA:CR	0	NA	NA	NA	NA	NA

lncRNA:CR	5.273793	-1.01041	3.929562	-0.25713	0.797079	NA
lncRNA:CR	0	NA	NA	NA	NA	NA
lncRNA:CR	0	NA	NA	NA	NA	NA
lncRNA:CR	0	NA	NA	NA	NA	NA
lncRNA:CR	0	NA	NA	NA	NA	NA
lncRNA:CR	0	NA	NA	NA	NA	NA
lncRNA:CR	0.132078	-1.13211	4.069918	-0.27817	0.780885	NA
CG44329	0	NA	NA	NA	NA	NA
lncRNA:CR	0	NA	NA	NA	NA	NA
lncRNA:CR	36.88016	3.915449	3.797741	1.030994	0.302543	NA
lncRNA:CR	0	NA	NA	NA	NA	NA
lncRNA:CR	0	NA	NA	NA	NA	NA
CG44335	0.356107	-1.47099	4.037764	-0.36431	0.715628	NA
lncRNA:TS	0	NA	NA	NA	NA	NA
lncRNA:CR	0	NA	NA	NA	NA	NA
lncRNA:CR	0	NA	NA	NA	NA	NA
lncRNA:CR	0	NA	NA	NA	NA	NA
asRNA:CR4	51.7467	1.177464	3.951818	0.297955	0.765738	NA
CG44341	0.5235	1.490714	4.02555	0.370313	0.711149	NA
lncRNA:CR	0	NA	NA	NA	NA	NA
lncRNA:CR	14.13231	-1.91351	3.953652	-0.48398	0.628397	NA
lncRNA:CR	0.178053	-1.16467	4.075067	-0.2858	0.775028	NA
lncRNA:CR	0	NA	NA	NA	NA	NA
lncRNA:CR	39.24263	-7.94987	3.910145	-2.03314	0.042038	NA
lncRNA:CR	0	NA	NA	NA	NA	NA
lncRNA:CR	0	NA	NA	NA	NA	NA
asRNA:CR4	0	NA	NA	NA	NA	NA
lncRNA:CR	0	NA	NA	NA	NA	NA
lncRNA:CR	0	NA	NA	NA	NA	NA
lncRNA:CR	0	NA	NA	NA	NA	NA
CG44355	0.536437	-1.71175	4.038042	-0.42391	0.671635	NA
lncRNA:CR	0.466186	0.959471	4.033986	0.237847	0.812	NA
asRNA:CR4	7.328995	1.727313	3.957831	0.436429	0.662525	NA
lncRNA:CR	2.509476	-1.87226	3.965749	-0.47211	0.636851	NA
lncRNA:CR	0	NA	NA	NA	NA	NA
CG44362	0	NA	NA	NA	NA	NA
lncRNA:CR	0.178053	-1.16467	4.075067	-0.2858	0.775028	NA
lncRNA:CR	0.807012	0.227825	3.984055	0.057184	0.954399	NA
lncRNA:CR	50.98879	2.974775	3.919148	0.759036	0.447831	NA
asRNA:CR4	0	NA	NA	NA	NA	NA
asRNA:CR4	57.45379	-1.92073	3.951586	-0.48607	0.626921	NA
lncRNA:CR	0	NA	NA	NA	NA	NA
asRNA:CR4	15.35954	1.623059	3.953965	0.410489	0.681447	NA
lncRNA:CR	0	NA	NA	NA	NA	NA
asRNA:CR4	0	NA	NA	NA	NA	NA
lncRNA:CR	0	NA	NA	NA	NA	NA
asRNA:CR4	0.69928	1.019866	4.009445	0.254366	0.799213	NA
asRNA:CR4	0.178812	-1.08808	4.061735	-0.26789	0.788788	NA
lncRNA:CR	0	NA	NA	NA	NA	NA
lncRNA:CR	0	NA	NA	NA	NA	NA

lncRNA:CR	0.233093	0.692656	4.076246	0.169925	0.865069	NA
lncRNA:CR	0.178053	-1.16467	4.075067	-0.2858	0.775028	NA
asRNA:CR4	0	NA	NA	NA	NA	NA
asRNA:CR4	0	NA	NA	NA	NA	NA
CR44383	2.043558	0.685373	3.968489	0.172704	0.862884	NA
asRNA:CR4	1.251686	-2.06022	4.011654	-0.51356	0.60756	NA
lncRNA:CR	0	NA	NA	NA	NA	NA
CG44388	0	NA	NA	NA	NA	NA
lncRNA:CR	0	NA	NA	NA	NA	NA
asRNA:CR4	36.40609	5.614564	3.869983	1.450798	0.146836	NA
CR44391	0	NA	NA	NA	NA	NA
lncRNA:CR	0	NA	NA	NA	NA	NA
lncRNA:CR	0	NA	NA	NA	NA	NA
CR44394	0	NA	NA	NA	NA	NA
lncRNA:CR	2.165895	0.59106	3.93154	0.150338	0.880498	NA
lncRNA:CR	0	NA	NA	NA	NA	NA
lncRNA:CR	0	NA	NA	NA	NA	NA
lncRNA:CR	0	NA	NA	NA	NA	NA
lncRNA:CR	0	NA	NA	NA	NA	NA
lncRNA:CR	0	NA	NA	NA	NA	NA
CG44403	0.209323	1.414133	4.032409	0.350692	0.725819	NA
IBIN	0	NA	NA	NA	NA	NA
CG44405	0	NA	NA	NA	NA	NA
CG44406	0.178812	-1.08808	4.061735	-0.26789	0.788788	NA
lncRNA:CR	0	NA	NA	NA	NA	NA
lncRNA:CR	0	NA	NA	NA	NA	NA
lncRNA:CR	0	NA	NA	NA	NA	NA
lncRNA:CR	0	NA	NA	NA	NA	NA
lncRNA:CR	0	NA	NA	NA	NA	NA
lncRNA:CR	0	NA	NA	NA	NA	NA
lncRNA:CR	0	NA	NA	NA	NA	NA
lncRNA:CR	0	NA	NA	NA	NA	NA
asRNA:CR4	0	NA	NA	NA	NA	NA
lncRNA:CR	0	NA	NA	NA	NA	NA
lncRNA:CR	0	NA	NA	NA	NA	NA
lncRNA:CR	0	NA	NA	NA	NA	NA
lncRNA:CR	0.178053	-1.16467	4.075067	-0.2858	0.775028	NA
Prosalpha4	0	NA	NA	NA	NA	NA
beat-IIIa	0	NA	NA	NA	NA	NA
CG44426	0	NA	NA	NA	NA	NA
asRNA:CR4	0	NA	NA	NA	NA	NA
CG44428	0	NA	NA	NA	NA	NA
lncRNA:CR	0	NA	NA	NA	NA	NA
asRNA:CR4	6.800512	1.520433	3.956126	0.384324	0.700738	NA
lncRNA:CR	0	NA	NA	NA	NA	NA
CG44433	0.356107	-1.47099	4.037764	-0.36431	0.715628	NA
CG44434	0	NA	NA	NA	NA	NA
CG44435	0	NA	NA	NA	NA	NA
CG44437	57.97741	-0.51855	3.729977	-0.13902	0.889433	NA
CG44438	0	NA	NA	NA	NA	NA

CG44439	0	NA	NA	NA	NA	NA
lncRNA:CR	36.53199	-2.17586	3.953338	-0.55038	0.582056	NA
lncRNA:CR	0.2094	1.005034	4.045341	0.248442	0.803792	NA
lncRNA:CR	0	NA	NA	NA	NA	NA
lncRNA:CR	0.356107	-1.47099	4.037764	-0.36431	0.715628	NA
lncRNA:CR	18.76824	-3.57913	3.799552	-0.94199	0.346199	NA
lncRNA:CR	0	NA	NA	NA	NA	NA
lncRNA:CR	16.75199	1.742418	3.953985	0.440674	0.659449	NA
lncRNA:CR	0.2094	1.005034	4.045341	0.248442	0.803792	NA
lncRNA:TS:	19.80125	1.470231	3.952641	0.371962	0.709921	NA
lncRNA:CR	53.04399	1.536977	3.951587	0.388952	0.697312	NA
lncRNA:CR	0	NA	NA	NA	NA	NA
lncRNA:CR	0	NA	NA	NA	NA	NA
lncRNA:CR	0	NA	NA	NA	NA	NA
lncRNA:CR	0	NA	NA	NA	NA	NA
lncRNA:CR	0	NA	NA	NA	NA	NA
lncRNA:CR	0	NA	NA	NA	NA	NA
lncRNA:CR	0	NA	NA	NA	NA	NA
lncRNA:CR	0.178053	-1.16467	4.075067	-0.2858	0.775028	NA
lncRNA:CR	0	NA	NA	NA	NA	NA
lncRNA:CR	0	NA	NA	NA	NA	NA
lncRNA:CR	0	NA	NA	NA	NA	NA
lncRNA:CR	0	NA	NA	NA	NA	NA
lncRNA:CR	0.178812	-1.08808	4.061735	-0.26789	0.788788	NA
lncRNA:CR	3.04686	1.344349	3.854011	0.348818	0.727226	NA
lncRNA:CR	0	NA	NA	NA	NA	NA
lncRNA:CR	0	NA	NA	NA	NA	NA
lncRNA:CR	0	NA	NA	NA	NA	NA
lncRNA:CR	0	NA	NA	NA	NA	NA
lncRNA:CR	0	NA	NA	NA	NA	NA
lncRNA:CR	0	NA	NA	NA	NA	NA
lncRNA:CR	0.104623	0.639075	4.067674	0.157111	0.875158	NA
lncRNA:CR	4.880176	-1.84563	3.862392	-0.47785	0.63276	NA
CG44475	0	NA	NA	NA	NA	NA
CG44476	2.503372	-2.15366	3.984641	-0.54049	0.588858	NA
CG44477	0	NA	NA	NA	NA	NA
lncRNA:CR	0	NA	NA	NA	NA	NA
lncRNA:CR	0	NA	NA	NA	NA	NA
lncRNA:CR	0	NA	NA	NA	NA	NA
lncRNA:CR	0	NA	NA	NA	NA	NA
lncRNA:CR	0.178053	-1.16467	4.075067	-0.2858	0.775028	NA
lncRNA:CR	0	NA	NA	NA	NA	NA
lncRNA:CR	0	NA	NA	NA	NA	NA
lncRNA:CR	0	NA	NA	NA	NA	NA
lncRNA:CR	0.282753	-0.25398	4.020204	-0.06318	0.949626	NA
lncRNA:CR	0	NA	NA	NA	NA	NA
lncRNA:CR	0	NA	NA	NA	NA	NA
lncRNA:CR	0	NA	NA	NA	NA	NA
lncRNA:CR	0	NA	NA	NA	NA	NA
lncRNA:CR	0	NA	NA	NA	NA	NA

lncRNA:CR	13.76214	-1.20321	3.952973	-0.30438	0.760838	NA
lncRNA:CR	3.782135	-0.08243	3.888969	-0.0212	0.98309	NA
lncRNA:CR	0	NA	NA	NA	NA	NA
lncRNA:CR	0.178053	-1.16467	4.075067	-0.2858	0.775028	NA
lncRNA:CR	32.77108	1.748369	3.95249	0.442346	0.658239	NA
lncRNA:CR	0	NA	NA	NA	NA	NA
lncRNA:CR	41.3135	2.474548	3.884939	0.636959	0.524151	NA
lncRNA:CR	0	NA	NA	NA	NA	NA
lncRNA:CR	5.989472	-3.42885	3.914999	-0.87582	0.381125	NA
lncRNA:CR	0.418493	1.313669	4.019283	0.326842	0.743788	NA
lncRNA:CR	0	NA	NA	NA	NA	NA
lncRNA:CR	2.809039	-0.20446	3.817847	-0.05355	0.95729	NA
lncRNA:CR	0	NA	NA	NA	NA	NA
lncRNA:CR	0.536437	-1.71175	4.038042	-0.42391	0.671635	NA
lncRNA:CR	36.73885	-0.44728	3.950028	-0.11323	0.909844	NA
lncRNA:CR	0	NA	NA	NA	NA	NA
lncRNA:CR	0	NA	NA	NA	NA	NA
asRNA:CR4	0	NA	NA	NA	NA	NA
lncRNA:CR	0	NA	NA	NA	NA	NA
lncRNA:CR	22.85558	7.152658	3.910546	1.829069	0.067389	NA
asRNA:CR4	11.98042	-2.25114	3.958753	-0.56865	0.569594	NA
lncRNA:CR	0	NA	NA	NA	NA	NA
lncRNA:CR	0	NA	NA	NA	NA	NA
lncRNA:CR	0.178053	-1.16467	4.075067	-0.2858	0.775028	NA
lncRNA:CR	0.310131	-1.94616	4.045058	-0.48112	0.630431	NA
lncRNA:CR	0.178053	-1.16467	4.075067	-0.2858	0.775028	NA
lncRNA:CR	0.104623	0.639075	4.067674	0.157111	0.875158	NA
lncRNA:CR	0.837599	1.568055	4.001801	0.391837	0.695178	NA
CG44521	0	NA	NA	NA	NA	NA
lncRNA:CR	0	NA	NA	NA	NA	NA
lncRNA:CR	0	NA	NA	NA	NA	NA
lncRNA:CR	0	NA	NA	NA	NA	NA
lncRNA:CR	0	NA	NA	NA	NA	NA
lncRNA:CR	0.356107	-1.47099	4.037764	-0.36431	0.715628	NA
lncRNA:CR	0	NA	NA	NA	NA	NA
lncRNA:CR	0	NA	NA	NA	NA	NA
lncRNA:CR	0	NA	NA	NA	NA	NA
lncRNA:CR	0	NA	NA	NA	NA	NA
CG44532	41.08433	-3.16599	3.905889	-0.81057	0.417614	NA
lncRNA:CR	0	NA	NA	NA	NA	NA
lncRNA:CR	0.104623	0.639075	4.067674	0.157111	0.875158	NA
lncRNA:CR	0.356107	-1.47099	4.037764	-0.36431	0.715628	NA
lncRNA:CR	0	NA	NA	NA	NA	NA
lncRNA:CR	0	NA	NA	NA	NA	NA
lncRNA:CR	0	NA	NA	NA	NA	NA
lncRNA:CR	0	NA	NA	NA	NA	NA
lncRNA:CR	0	NA	NA	NA	NA	NA
lncRNA:CR	0	NA	NA	NA	NA	NA
lncRNA:CR	0	NA	NA	NA	NA	NA
lncRNA:CR	56.83034	-1.66616	3.951565	-0.42165	0.673284	NA

lncRNA:CR	0	NA	NA	NA	NA	NA
lncRNA:CR	0	NA	NA	NA	NA	NA
lncRNA:CR	0.178053	-1.16467	4.075067	-0.2858	0.775028	NA
lncRNA:CR	0	NA	NA	NA	NA	NA
lncRNA:CR	0	NA	NA	NA	NA	NA
lncRNA:CR	0	NA	NA	NA	NA	NA
lncRNA:CR	0	NA	NA	NA	NA	NA
lncRNA:CR	0.732516	2.619177	3.987823	0.656794	0.511314	NA
lncRNA:CR	0	NA	NA	NA	NA	NA
lncRNA:CR	17.30218	-1.91525	3.953153	-0.48449	0.628041	NA
lncRNA:CR	0	NA	NA	NA	NA	NA
lncRNA:CR	0	NA	NA	NA	NA	NA
lncRNA:CR	21.67767	1.173743	3.953075	0.296919	0.766528	NA
lncRNA:CR	0	NA	NA	NA	NA	NA
lncRNA:CR	0.712213	-1.57119	3.998613	-0.39293	0.694369	NA
lncRNA:CR	0	NA	NA	NA	NA	NA
lncRNA:CR	0	NA	NA	NA	NA	NA
lncRNA:CR	0	NA	NA	NA	NA	NA
lncRNA:CR	0	NA	NA	NA	NA	NA
lncRNA:CR	0	NA	NA	NA	NA	NA
lncRNA:CR	0	NA	NA	NA	NA	NA
lncRNA:CR	1.452855	-1.83946	3.975716	-0.46267	0.643599	NA
lncRNA:CR	35.62268	-0.27627	3.83623	-0.07202	0.94259	NA
lncRNA:CR	0.178053	-1.16467	4.075067	-0.2858	0.775028	NA
CG44569	0	NA	NA	NA	NA	NA
lncRNA:CR	1.42283	-0.01324	3.949901	-0.00335	0.997325	NA
lncRNA:CR	0	NA	NA	NA	NA	NA
lncRNA:CR	0.178053	-1.16467	4.075067	-0.2858	0.775028	NA
lncRNA:CR	0	NA	NA	NA	NA	NA
CG44574	0	NA	NA	NA	NA	NA
lncRNA:CR	0	NA	NA	NA	NA	NA
lncRNA:CR	0	NA	NA	NA	NA	NA
lncRNA:CR	0	NA	NA	NA	NA	NA
lncRNA:CR	0	NA	NA	NA	NA	NA
lncRNA:CR	0	NA	NA	NA	NA	NA
lncRNA:CR	0	NA	NA	NA	NA	NA
lncRNA:CR	0.178812	-1.08808	4.061735	-0.26789	0.788788	NA
lncRNA:CR	0	NA	NA	NA	NA	NA
lncRNA:CR	0	NA	NA	NA	NA	NA
lncRNA:CR	0	NA	NA	NA	NA	NA
lncRNA:CR	0	NA	NA	NA	NA	NA
lncRNA:CR	0	NA	NA	NA	NA	NA
lncRNA:CR	0	NA	NA	NA	NA	NA
lncRNA:CR	0	NA	NA	NA	NA	NA
lncRNA:CR	0	NA	NA	NA	NA	NA
asRNA:CR4	0	NA	NA	NA	NA	NA
lncRNA:CR	5.299592	-1.24712	3.956386	-0.31522	0.752597	NA
lncRNA:CR	0.282753	-0.25398	4.020204	-0.06318	0.949626	NA
lncRNA:CR	28.0941	0.324571	3.837217	0.084585	0.932591	NA
lncRNA:CR	34.73333	-3.05451	3.802354	-0.80332	0.421789	NA

lncRNA:CR	0.837599	1.568055	4.001801	0.391837	0.695178	NA
lncRNA:CR	1.434452	0.968283	3.97124	0.243824	0.807367	NA
asRNA:CR4	0	NA	NA	NA	NA	NA
asRNA:CR4	0	NA	NA	NA	NA	NA
lncRNA:CR	0	NA	NA	NA	NA	NA
lncRNA:CR	0.310131	-1.94616	4.045058	-0.48112	0.630431	NA
lncRNA:CR	0	NA	NA	NA	NA	NA
lncRNA:CR	0	NA	NA	NA	NA	NA
lncRNA:CR	0	NA	NA	NA	NA	NA
lncRNA:CR	0.492153	0.308117	3.993933	0.077146	0.938507	NA
lncRNA:CR	0	NA	NA	NA	NA	NA
lncRNA:CR	0	NA	NA	NA	NA	NA
lncRNA:CR	0	NA	NA	NA	NA	NA
lncRNA:CR	0	NA	NA	NA	NA	NA
lncRNA:CR	0	NA	NA	NA	NA	NA
lncRNA:CR	0.104623	0.639075	4.067674	0.157111	0.875158	NA
lncRNA:CR	0	NA	NA	NA	NA	NA
lncRNA:CR	0	NA	NA	NA	NA	NA
lncRNA:CR	0	NA	NA	NA	NA	NA
lncRNA:CR	0	NA	NA	NA	NA	NA
lncRNA:CR	0	NA	NA	NA	NA	NA
lncRNA:CR	0	NA	NA	NA	NA	NA
lncRNA:CR	60.90505	6.471408	3.858031	1.677386	0.093467	NA
lncRNA:CR	0.104623	0.639075	4.067674	0.157111	0.875158	NA
CG44622	0	NA	NA	NA	NA	NA
CG44623	0	NA	NA	NA	NA	NA
CG44624	0	NA	NA	NA	NA	NA
CG44625	0	NA	NA	NA	NA	NA
lncRNA:CR	0	NA	NA	NA	NA	NA
lncRNA:CR	0	NA	NA	NA	NA	NA
lncRNA:CR	0	NA	NA	NA	NA	NA
lncRNA:CR	0	NA	NA	NA	NA	NA
lncRNA:CR	0.104623	0.639075	4.067674	0.157111	0.875158	NA
lncRNA:CR	4.920897	1.714724	3.961081	0.432893	0.665093	NA
lncRNA:CR	0	NA	NA	NA	NA	NA
lncRNA:CR	25.63925	-5.92924	3.896742	-1.52159	0.128112	NA
lncRNA:CR	0	NA	NA	NA	NA	NA
lncRNA:CR	0	NA	NA	NA	NA	NA
lncRNA:CR	0.132078	-1.13211	4.069918	-0.27817	0.780885	NA
lncRNA:CR	0	NA	NA	NA	NA	NA
lncRNA:CR	11.0393	-1.69765	3.954364	-0.42931	0.667697	NA
lncRNA:CR	2.814602	0.57184	3.871466	0.147706	0.882575	NA
lncRNA:CR	12.14519	1.737886	3.955133	0.4394	0.660372	NA
lncRNA:CR	17.69429	1.74306	3.953824	0.440854	0.659319	NA
lncRNA:CR	0	NA	NA	NA	NA	NA
lncRNA:CR	0	NA	NA	NA	NA	NA
lncRNA:CR	0	NA	NA	NA	NA	NA
lncRNA:CR	0	NA	NA	NA	NA	NA
lncRNA:CR	0	NA	NA	NA	NA	NA
lncRNA:CR	0	NA	NA	NA	NA	NA

lncRNA:CR	0	NA	NA	NA	NA	NA
asRNA:CR4	0	NA	NA	NA	NA	NA
lncRNA:CR	0.1047	0.613261	4.062799	0.150945	0.880019	NA
lncRNA:CR	1.351073	-1.13435	3.970931	-0.28566	0.775136	NA
lncRNA:CR	0.356866	-1.92326	4.037889	-0.4763	0.633858	NA
lncRNA:CR	0	NA	NA	NA	NA	NA
CR44659	0	NA	NA	NA	NA	NA
lncRNA:CR	0	NA	NA	NA	NA	NA
lncRNA:CR	0	NA	NA	NA	NA	NA
lncRNA:CR	0.104623	0.639075	4.067674	0.157111	0.875158	NA
lncRNA:CR	0	NA	NA	NA	NA	NA
CG44666	0.178053	-1.16467	4.075067	-0.2858	0.775028	NA
lncRNA:CR	0	NA	NA	NA	NA	NA
lncRNA:CR	0	NA	NA	NA	NA	NA
lncRNA:CR	0	NA	NA	NA	NA	NA
lncRNA:CR	0	NA	NA	NA	NA	NA
lncRNA:CR	0	NA	NA	NA	NA	NA
lncRNA:CR	0	NA	NA	NA	NA	NA
lncRNA:CR	0	NA	NA	NA	NA	NA
lncRNA:CR	0.1047	0.613261	4.062799	0.150945	0.880019	NA
lncRNA:CR	0	NA	NA	NA	NA	NA
lncRNA:CR	0	NA	NA	NA	NA	NA
lncRNA:CR	0	NA	NA	NA	NA	NA
lncRNA:CR	0	NA	NA	NA	NA	NA
lncRNA:CR	0.132078	-1.13211	4.069918	-0.27817	0.780885	NA
lncRNA:CR	0	NA	NA	NA	NA	NA
lncRNA:CR	0	NA	NA	NA	NA	NA
lncRNA:CR	0	NA	NA	NA	NA	NA
lncRNA:CR	0	NA	NA	NA	NA	NA
lncRNA:CR	0	NA	NA	NA	NA	NA
lncRNA:CR	0	NA	NA	NA	NA	NA
lncRNA:CR	0	NA	NA	NA	NA	NA
lncRNA:CR	0	NA	NA	NA	NA	NA
lncRNA:CR	0	NA	NA	NA	NA	NA
lncRNA:CR	0	NA	NA	NA	NA	NA
asRNA:CR4	3.030212	1.13655	3.965915	0.28658	0.774434	NA
lncRNA:CR	0	NA	NA	NA	NA	NA
lncRNA:CR	3.204959	-1.67301	3.96255	-0.42221	0.672875	NA
lncRNA:CR	0	NA	NA	NA	NA	NA
lncRNA:CR	0	NA	NA	NA	NA	NA
lncRNA:CR	0	NA	NA	NA	NA	NA
lncRNA:CR	0	NA	NA	NA	NA	NA
lncRNA:CR	0	NA	NA	NA	NA	NA
lncRNA:CR	0	NA	NA	NA	NA	NA
lncRNA:CR	0	NA	NA	NA	NA	NA
lncRNA:CR	0	NA	NA	NA	NA	NA
lncRNA:CR	0	NA	NA	NA	NA	NA
lncRNA:CR	24.18053	0.369972	3.836136	0.096444	0.923168	NA
asRNA:CR4	9.520716	1.525762	3.954655	0.385814	0.699634	NA
asRNA:CR4	12.65941	1.529109	3.953735	0.386751	0.698941	NA

lncRNA:CR	0	NA	NA	NA	NA	NA
CR44706	0	NA	NA	NA	NA	NA
lncRNA:CR	0	NA	NA	NA	NA	NA
lncRNA:CR	0	NA	NA	NA	NA	NA
lncRNA:CR	0	NA	NA	NA	NA	NA
lncRNA:CR	0	NA	NA	NA	NA	NA
lncRNA:CR	0	NA	NA	NA	NA	NA
lncRNA:CR	0	NA	NA	NA	NA	NA
lncRNA:CR	0	NA	NA	NA	NA	NA
lncRNA:CR	9.004194	1.73223	3.956573	0.437811	0.661523	NA
lncRNA:CR	13.52991	4.302045	3.889439	1.106084	0.26869	NA
lncRNA:CR	0	NA	NA	NA	NA	NA
lncRNA:CR	8.132723	-5.4006	3.911847	-1.38058	0.167409	NA
lncRNA:CR	0	NA	NA	NA	NA	NA
lncRNA:CR	0	NA	NA	NA	NA	NA
lncRNA:CR	0	NA	NA	NA	NA	NA
lncRNA:CR	0	NA	NA	NA	NA	NA
lncRNA:CR	0.752547	0.662441	3.960528	0.167261	0.867165	NA
asRNA:CR4	32.72265	-2.27063	3.95384	-0.57428	0.565776	NA
lncRNA:CR	0	NA	NA	NA	NA	NA
lncRNA:CR	0	NA	NA	NA	NA	NA
lncRNA:CR	0.488184	-2.32943	4.022674	-0.57908	0.562538	NA
lncRNA:CR	0	NA	NA	NA	NA	NA
lncRNA:CR	0	NA	NA	NA	NA	NA
lncRNA:CR	0	NA	NA	NA	NA	NA
lncRNA:CR	0	NA	NA	NA	NA	NA
lncRNA:CR	0.233093	0.692656	4.076246	0.169925	0.865069	NA
lncRNA:CR	0	NA	NA	NA	NA	NA
lncRNA:CR	0	NA	NA	NA	NA	NA
lncRNA:CR	0	NA	NA	NA	NA	NA
lncRNA:CR	0	NA	NA	NA	NA	NA
lncRNA:CR	21.36913	-1.85672	3.95265	-0.46974	0.63854	NA
lncRNA:CR	0.357625	-1.47707	4.044794	-0.36518	0.714979	NA
lncRNA:CR	0	NA	NA	NA	NA	NA
lncRNA:CR	0	NA	NA	NA	NA	NA
lncRNA:CR	0	NA	NA	NA	NA	NA
lncRNA:CR	0.178053	-1.16467	4.075067	-0.2858	0.775028	NA
lncRNA:CR	28.74697	-1.04237	3.91275	-0.2664	0.789928	NA
lncRNA:CR	0	NA	NA	NA	NA	NA
lncRNA:CR	0	NA	NA	NA	NA	NA
lncRNA:CR	0	NA	NA	NA	NA	NA
lncRNA:CR	0	NA	NA	NA	NA	NA
lncRNA:CR	0	NA	NA	NA	NA	NA
lncRNA:CR	0	NA	NA	NA	NA	NA
lncRNA:CR	0.132078	-1.13211	4.069918	-0.27817	0.780885	NA
lncRNA:CR	1.259804	-3.02464	3.93518	-0.76862	0.442122	NA
Tfb5	0	NA	NA	NA	NA	NA
lncRNA:CR	0	NA	NA	NA	NA	NA
lncRNA:CR	0.233093	0.692656	4.076246	0.169925	0.865069	NA
lncRNA:CR	9.416093	1.525613	3.954696	0.385773	0.699665	NA

lncRNA:CR	51.4574	-1.70598	3.951653	-0.43171	0.66595	NA
lncRNA:CR	0	NA	NA	NA	NA	NA
lncRNA:CR	0	NA	NA	NA	NA	NA
lncRNA:CR	0	NA	NA	NA	NA	NA
lncRNA:CR	0	NA	NA	NA	NA	NA
lncRNA:CR	0	NA	NA	NA	NA	NA
lncRNA:CR	0	NA	NA	NA	NA	NA
lncRNA:CR	0	NA	NA	NA	NA	NA
lncRNA:CR	0	NA	NA	NA	NA	NA
lncRNA:CR	0	NA	NA	NA	NA	NA
lncRNA:CR	0.1047	0.613261	4.062799	0.150945	0.880019	NA
lncRNA:CR	0.337793	1.451235	4.039844	0.35923	0.719423	NA
lncRNA:CR	0	NA	NA	NA	NA	NA
lncRNA:CR	0	NA	NA	NA	NA	NA
lncRNA:CR	0	NA	NA	NA	NA	NA
lncRNA:CR	0.104623	0.639075	4.067674	0.157111	0.875158	NA
lncRNA:CR	49.68049	-3.09354	3.917238	-0.78972	0.429689	NA
lncRNA:CR	0	NA	NA	NA	NA	NA
lncRNA:CR	0.132078	-1.13211	4.069918	-0.27817	0.780885	NA
lncRNA:CR	0	NA	NA	NA	NA	NA
lncRNA:CR	0	NA	NA	NA	NA	NA
lncRNA:CR	0	NA	NA	NA	NA	NA
lncRNA:CR	0.1047	0.613261	4.062799	0.150945	0.880019	NA
lncRNA:CR	0	NA	NA	NA	NA	NA
lncRNA:CR	0	NA	NA	NA	NA	NA
lncRNA:CR	28.3967	-1.9183	3.952279	-0.48537	0.627417	NA
lncRNA:CR	0	NA	NA	NA	NA	NA
lncRNA:CR	0	NA	NA	NA	NA	NA
asRNA:TS1	0	NA	NA	NA	NA	NA
asRNA:CR4	0	NA	NA	NA	NA	NA
lncRNA:CR	0.233093	0.692656	4.076246	0.169925	0.865069	NA
lncRNA:CR	0	NA	NA	NA	NA	NA
lncRNA:CR	0	NA	NA	NA	NA	NA
lncRNA:CR	0	NA	NA	NA	NA	NA
lncRNA:CR	0	NA	NA	NA	NA	NA
lncRNA:CR	0	NA	NA	NA	NA	NA
lncRNA:CR	26.32944	-3.50887	3.901453	-0.89938	0.368453	NA
lncRNA:CR	0	NA	NA	NA	NA	NA
lncRNA:CR	0.178812	-1.08808	4.061735	-0.26789	0.788788	NA
lncRNA:CR	0.356107	-1.47099	4.037764	-0.36431	0.715628	NA
lncRNA:CR	0	NA	NA	NA	NA	NA
asRNA:CR4	0	NA	NA	NA	NA	NA
lncRNA:CR	0	NA	NA	NA	NA	NA
lncRNA:CR	37.06377	1.749099	3.952308	0.442551	0.65809	NA
lncRNA:CR	0	NA	NA	NA	NA	NA
lncRNA:CR	0	NA	NA	NA	NA	NA
lncRNA:CR	0.536437	-1.71175	4.038042	-0.42391	0.671635	NA
CG44815	48.71464	-6.40365	3.803354	-1.68369	0.092243	NA
lncRNA:CR	0	NA	NA	NA	NA	NA
lncRNA:CR	33.01942	-1.91897	3.952088	-0.48556	0.62728	NA

asRNA:CR4	0	NA	NA	NA	NA	NA
lncRNA:CR	0	NA	NA	NA	NA	NA
lncRNA:CR	0.178053	-1.16467	4.075067	-0.2858	0.775028	NA
lncRNA:CR	0.1047	0.613261	4.062799	0.150945	0.880019	NA
lncRNA:CR	0.178053	-1.16467	4.075067	-0.2858	0.775028	NA
lncRNA:CR	0	NA	NA	NA	NA	NA
lncRNA:CR	0	NA	NA	NA	NA	NA
lncRNA:CR	0	NA	NA	NA	NA	NA
lncRNA:CR	33.36378	-0.87424	3.949021	-0.22138	0.824796	NA
lncRNA:CR	0	NA	NA	NA	NA	NA
lncRNA:CR	26.25761	0.295888	3.835781	0.077139	0.938513	NA
lncRNA:CR	0.178053	-1.16467	4.075067	-0.2858	0.775028	NA
lncRNA:CR	6.386696	1.723466	3.95882	0.435348	0.66331	NA
lncRNA:CR	45.16509	1.606671	3.397908	0.472841	0.636327	NA
lncRNA:CR	23.42968	-1.65532	3.952493	-0.41881	0.675359	NA
lncRNA:CR	0.233093	0.692656	4.076246	0.169925	0.865069	NA
CG44836	0.675302	-2.05376	3.984422	-0.51545	0.606241	NA
CR44839	0	NA	NA	NA	NA	NA
ana3	2.703587	-2.11665	3.896693	-0.54319	0.586998	NA
asRNA:CR4	0	NA	NA	NA	NA	NA
lncRNA:CR	0	NA	NA	NA	NA	NA
asRNA:CR4	3.86362	-1.36225	3.963244	-0.34372	0.731057	NA
Gyc76C	10.67704	-1.33672	3.747935	-0.35666	0.72135	NA
lncRNA:CR	29.95418	-1.87492	3.952167	-0.4744	0.635212	NA
lncRNA:CR	0.1047	0.613261	4.062799	0.150945	0.880019	NA
lncRNA:CR	0.1047	0.613261	4.062799	0.150945	0.880019	NA
asRNA:CR4	0	NA	NA	NA	NA	NA
lncRNA:CR	1.849088	-1.85577	3.970721	-0.46736	0.640241	NA
asRNA:CR4	0	NA	NA	NA	NA	NA
asRNA:CR4	0	NA	NA	NA	NA	NA
lncRNA:CR	40.77652	-0.3465	3.951463	-0.08769	0.930124	NA
lncRNA:CR	0	NA	NA	NA	NA	NA
lncRNA:CR	0	NA	NA	NA	NA	NA
lncRNA:CR	0	NA	NA	NA	NA	NA
lncRNA:CR	0	NA	NA	NA	NA	NA
lncRNA:CR	0	NA	NA	NA	NA	NA
lncRNA:CR	0	NA	NA	NA	NA	NA
lncRNA:CR	0	NA	NA	NA	NA	NA
lncRNA:CR	0	NA	NA	NA	NA	NA
lncRNA:CR	0	NA	NA	NA	NA	NA
lncRNA:CR	0	NA	NA	NA	NA	NA
lncRNA:CR	0.178812	-1.08808	4.061735	-0.26789	0.788788	NA
lncRNA:CR	0	NA	NA	NA	NA	NA
asRNA:CR4	0	NA	NA	NA	NA	NA
lncRNA:CR	0	NA	NA	NA	NA	NA
lncRNA:CR	0.732899	1.548046	4.007786	0.38626	0.699304	NA
CG44869	0	NA	NA	NA	NA	NA
lncRNA:CR	0	NA	NA	NA	NA	NA
lncRNA:CR	0.712213	-1.57119	3.998613	-0.39293	0.694369	NA
asRNA:CR4	0	NA	NA	NA	NA	NA

asRNA:CR4	13.47192	-1.91304	3.953785	-0.48385	0.628491	NA
lncRNA:CR	58.58902	1.537214	3.951522	0.389018	0.697263	NA
lncRNA:CR	0	NA	NA	NA	NA	NA
pre-mod(n	0	NA	NA	NA	NA	NA
pre-mod(n	0	NA	NA	NA	NA	NA
pre-mod(n	0	NA	NA	NA	NA	NA
pre-mod(n	0	NA	NA	NA	NA	NA
pre-mod(n	0	NA	NA	NA	NA	NA
pre-mod(n	0	NA	NA	NA	NA	NA
pre-mod(n	0	NA	NA	NA	NA	NA
pre-mod(n	0	NA	NA	NA	NA	NA
pre-mod(n	0	NA	NA	NA	NA	NA
lncRNA:CR	0	NA	NA	NA	NA	NA
lncRNA:CR	14.34389	1.740403	3.954495	0.440108	0.659859	NA
lncRNA:CR	0	NA	NA	NA	NA	NA
lncRNA:CR	0	NA	NA	NA	NA	NA
lncRNA:CR	0	NA	NA	NA	NA	NA
Tgs1	0	NA	NA	NA	NA	NA
asRNA:CR4	0	NA	NA	NA	NA	NA
lncRNA:CR	0	NA	NA	NA	NA	NA
asRNA:CR4	2.128938	-1.0769	3.887431	-0.27702	0.781765	NA
lncRNA:CR	0.357625	-1.47707	4.044794	-0.36518	0.714979	NA
asRNA:CR4	0	NA	NA	NA	NA	NA
asRNA:CR4	0	NA	NA	NA	NA	NA
lncRNA:CR	0	NA	NA	NA	NA	NA
lncRNA:CR	0	NA	NA	NA	NA	NA
lncRNA:CR	14.91797	1.17086	3.95405	0.296117	0.767141	NA
lncRNA:CR	0	NA	NA	NA	NA	NA
lncRNA:CR	0	NA	NA	NA	NA	NA
lncRNA:CR	0	NA	NA	NA	NA	NA
lncRNA:CR	0	NA	NA	NA	NA	NA
CR44906	0	NA	NA	NA	NA	NA
asRNA:CR4	0	NA	NA	NA	NA	NA
lncRNA:CR	0	NA	NA	NA	NA	NA
asRNA:CR4	0	NA	NA	NA	NA	NA
asRNA:CR4	46.52949	2.372784	3.844405	0.617204	0.5371	NA
lncRNA:CR	0	NA	NA	NA	NA	NA
lncRNA:CR	0	NA	NA	NA	NA	NA
lncRNA:CR	19.96634	1.650754	3.953312	0.417562	0.676267	NA
lncRNA:CR	0	NA	NA	NA	NA	NA
lncRNA:CR	0	NA	NA	NA	NA	NA
lncRNA:CR	0	NA	NA	NA	NA	NA
lncRNA:CR	0.387376	0.625691	4.009146	0.156066	0.875981	NA
lncRNA:CR	0	NA	NA	NA	NA	NA
lncRNA:CR	0	NA	NA	NA	NA	NA
lncRNA:CR	0.233093	0.692656	4.076246	0.169925	0.865069	NA
lncRNA:CR	0	NA	NA	NA	NA	NA
lncRNA:CR	0	NA	NA	NA	NA	NA
lncRNA:CR	0	NA	NA	NA	NA	NA
lncRNA:CR	0	NA	NA	NA	NA	NA

lncRNA:CR	0.104623	0.639075	4.067674	0.157111	0.875158	NA
lncRNA:CR	0.178053	-1.16467	4.075067	-0.2858	0.775028	NA
lncRNA:CR	0	NA	NA	NA	NA	NA
lncRNA:CR	0	NA	NA	NA	NA	NA
lncRNA:CR	0	NA	NA	NA	NA	NA
lncRNA:CR	0	NA	NA	NA	NA	NA
lncRNA:CR	0	NA	NA	NA	NA	NA
lncRNA:CR	0	NA	NA	NA	NA	NA
lncRNA:CR	0	NA	NA	NA	NA	NA
lncRNA:CR	0	NA	NA	NA	NA	NA
lncRNA:CR	6.247973	0.287943	3.851599	0.074759	0.940406	NA
lncRNA:CR	0	NA	NA	NA	NA	NA
lncRNA:CR	0	NA	NA	NA	NA	NA
lncRNA:CR	0	NA	NA	NA	NA	NA
lncRNA:CR	0	NA	NA	NA	NA	NA
lncRNA:CR	0.282677	-0.09599	4.057934	-0.02366	0.981127	NA
lncRNA:CR	0	NA	NA	NA	NA	NA
lncRNA:CR	0	NA	NA	NA	NA	NA
lncRNA:CR	0.178053	-1.16467	4.075067	-0.2858	0.775028	NA
lncRNA:CR	0.466186	0.959471	4.033986	0.237847	0.812	NA
lncRNA:CR	0	NA	NA	NA	NA	NA
lncRNA:CR	0	NA	NA	NA	NA	NA
lncRNA:CR	0	NA	NA	NA	NA	NA
lncRNA:CR	0.233093	0.692656	4.076246	0.169925	0.865069	NA
lncRNA:CR	0	NA	NA	NA	NA	NA
lncRNA:CR	0.132078	-1.13211	4.069918	-0.27817	0.780885	NA
CG44954	0	NA	NA	NA	NA	NA
lncRNA:CR	0.337793	1.451235	4.039844	0.35923	0.719423	NA
CG44956	0	NA	NA	NA	NA	NA
lncRNA:CR	0.2094	1.005034	4.045341	0.248442	0.803792	NA
asRNA:CR4	0	NA	NA	NA	NA	NA
asRNA:CR4	0	NA	NA	NA	NA	NA
asRNA:CR4	1.070597	-3.05742	3.990949	-0.76609	0.443624	NA
asRNA:CR4	0.806253	0.654822	3.981166	0.16448	0.869353	NA
asRNA:CR4	2.860997	-2.16734	3.980875	-0.54444	0.586141	NA
lncRNA:CR	0	NA	NA	NA	NA	NA
lncRNA:CR	0	NA	NA	NA	NA	NA
lncRNA:CR	21.43243	-3.03925	3.86974	-0.78539	0.432226	NA
lncRNA:CR	0	NA	NA	NA	NA	NA
lncRNA:CR	0	NA	NA	NA	NA	NA
asRNA:CR4	0	NA	NA	NA	NA	NA
lncRNA:CR	0	NA	NA	NA	NA	NA
lncRNA:CR	0	NA	NA	NA	NA	NA
asRNA:CR4	0	NA	NA	NA	NA	NA
lncRNA:CR	0.5235	1.490714	4.02555	0.370313	0.711149	NA
lncRNA:CR	0	NA	NA	NA	NA	NA
asRNA:CR4	10.67157	1.527223	3.954255	0.386223	0.699332	NA
asRNA:CR4	0	NA	NA	NA	NA	NA
lncRNA:CR	24.70517	0.356184	3.838128	0.092801	0.926061	NA
lncRNA:CR	0	NA	NA	NA	NA	NA

asRNA:CR4	0	NA	NA	NA	NA	NA
lncRNA:CR	0	NA	NA	NA	NA	NA
asRNA:CR4	0	NA	NA	NA	NA	NA
lncRNA:CR	0.1047	0.613261	4.062799	0.150945	0.880019	NA
lncRNA:CR	0	NA	NA	NA	NA	NA
lncRNA:CR	0	NA	NA	NA	NA	NA
asRNA:CR4	0	NA	NA	NA	NA	NA
asRNA:CR4	0	NA	NA	NA	NA	NA
lncRNA:CR	18.94521	-0.23186	3.861384	-0.06005	0.952119	NA
lncRNA:CR	33.64127	-0.93046	3.896581	-0.23879	0.811269	NA
lncRNA:CR	0.178053	-1.16467	4.075067	-0.2858	0.775028	NA
asRNA:CR4	0	NA	NA	NA	NA	NA
asRNA:CR4	1.06832	-1.61145	3.983918	-0.40449	0.685853	NA
asRNA:CR4	6.568725	-2.05969	3.83095	-0.53764	0.590823	NA
lncRNA:CR	0	NA	NA	NA	NA	NA
asRNA:CR4	4.816197	1.713907	3.961293	0.432664	0.665259	NA
asRNA:CR4	0	NA	NA	NA	NA	NA
lncRNA:CR	0	NA	NA	NA	NA	NA
lncRNA:CR	24.91851	2.96466	3.92141	0.756019	0.449638	NA
lncRNA:CR	0	NA	NA	NA	NA	NA
lncRNA:CR	0	NA	NA	NA	NA	NA
asRNA:CR4	0	NA	NA	NA	NA	NA
asRNA:CR4	0	NA	NA	NA	NA	NA
lncRNA:CR	0	NA	NA	NA	NA	NA
CG45011	0.178053	-1.16467	4.075067	-0.2858	0.775028	NA
CG45012	0	NA	NA	NA	NA	NA
lncRNA:CR	0	NA	NA	NA	NA	NA
lncRNA:CR	0	NA	NA	NA	NA	NA
asRNA:CR4	0	NA	NA	NA	NA	NA
asRNA:CR4	7.642194	-3.40016	3.943094	-0.86231	0.388519	NA
lncRNA:CR	0	NA	NA	NA	NA	NA
lncRNA:CR	0	NA	NA	NA	NA	NA
lncRNA:CR	0.1047	0.613261	4.062799	0.150945	0.880019	NA
lncRNA:CR	0	NA	NA	NA	NA	NA
lncRNA:CR	3.978597	1.705937	3.963372	0.430426	0.666886	NA
asRNA:CR4	0	NA	NA	NA	NA	NA
asRNA:CR4	0	NA	NA	NA	NA	NA
lncRNA:CR	0	NA	NA	NA	NA	NA
lncRNA:CR	0	NA	NA	NA	NA	NA
lncRNA:CR	0	NA	NA	NA	NA	NA
CR45033	0	NA	NA	NA	NA	NA
lncRNA:CR	0	NA	NA	NA	NA	NA
lncRNA:CR	21.51768	-0.07372	3.880056	-0.019	0.984841	NA
lncRNA:CR	18.52259	5.861174	3.857864	1.519279	0.128692	NA
lncRNA:CR	0	NA	NA	NA	NA	NA
lncRNA:CR	0	NA	NA	NA	NA	NA
lncRNA:CR	0	NA	NA	NA	NA	NA
lncRNA:CR	0	NA	NA	NA	NA	NA
asRNA:CR4	0	NA	NA	NA	NA	NA
asRNA:CR4	0	NA	NA	NA	NA	NA

lncRNA:CR	0	NA	NA	NA	NA	NA
asRNA:CR4	3.729492	1.144349	3.963186	0.288745	0.772777	NA
CG45045	0	NA	NA	NA	NA	NA
asRNA:CR4	0	NA	NA	NA	NA	NA
lncRNA:CR	15.06118	-1.62798	3.95333	-0.4118	0.680487	NA
lncRNA:CR	54.756	-9.28132	3.844412	-2.41424	0.015768	NA
lncRNA:CR	25.81773	-1.70371	3.952393	-0.43106	0.666426	NA
asRNA:CR4	6.972663	-1.74081	3.955814	-0.44006	0.65989	NA
lncRNA:CR	0	NA	NA	NA	NA	NA
asRNA:CR4	0.712213	-1.57119	3.998613	-0.39293	0.694369	NA
lncRNA:CR	0	NA	NA	NA	NA	NA
asRNA:CR4	0	NA	NA	NA	NA	NA
CG45060	0	NA	NA	NA	NA	NA
CG45061	4.397397	1.710278	3.962238	0.431644	0.666	NA
CG45062	3.830253	-1.88895	3.960794	-0.47691	0.633424	NA
CG45063	0.178053	-1.16467	4.075067	-0.2858	0.775028	NA
CG45064	0	NA	NA	NA	NA	NA
CG45065	0	NA	NA	NA	NA	NA
CG45066	0	NA	NA	NA	NA	NA
CG45067	0	NA	NA	NA	NA	NA
PIG-Z	0	NA	NA	NA	NA	NA
CG45069	0	NA	NA	NA	NA	NA
CG45072	0.132078	-1.13211	4.069918	-0.27817	0.780885	NA
CG45073	0	NA	NA	NA	NA	NA
CG45075	0	NA	NA	NA	NA	NA
CG45076	0.233093	0.692656	4.076246	0.169925	0.865069	NA
CG45078	0	NA	NA	NA	NA	NA
fau	0	NA	NA	NA	NA	NA
CG45079	0	NA	NA	NA	NA	NA
CG45080	0	NA	NA	NA	NA	NA
CG45081	0	NA	NA	NA	NA	NA
lncRNA:CR	26.78423	-0.33788	3.8373	-0.08805	0.929836	NA
CG45084	0	NA	NA	NA	NA	NA
CG45085	0	NA	NA	NA	NA	NA
CG45086	0	NA	NA	NA	NA	NA
CG45087	0	NA	NA	NA	NA	NA
CG45088	0	NA	NA	NA	NA	NA
CG45089	0	NA	NA	NA	NA	NA
SclA	0	NA	NA	NA	NA	NA
SclB	0	NA	NA	NA	NA	NA
CG45092	0	NA	NA	NA	NA	NA
CG45095	0	NA	NA	NA	NA	NA
lncRNA:CR	0	NA	NA	NA	NA	NA
lncRNA:CR	0	NA	NA	NA	NA	NA
lncRNA:CR	0	NA	NA	NA	NA	NA
CG45099	0	NA	NA	NA	NA	NA
CG45100	0	NA	NA	NA	NA	NA
CG45101	0	NA	NA	NA	NA	NA
lncRNA:CR	0	NA	NA	NA	NA	NA
asRNA:CR4	1.762219	-0.18545	3.949645	-0.04695	0.962551	NA

CG45105	0.178053	-1.16467	4.075067	-0.2858	0.775028	NA
lncRNA:yaı	0.104623	0.639075	4.067674	0.157111	0.875158	NA
tRNA:Thr-1	0	NA	NA	NA	NA	NA
tRNA:Thr-1	0	NA	NA	NA	NA	NA
asRNA:CR4	0	NA	NA	NA	NA	NA
lncRNA:CR	0	NA	NA	NA	NA	NA
tRNA:Leu-(	0.357625	-1.47707	4.044794	-0.36518	0.714979	NA
lncRNA:CR	0	NA	NA	NA	NA	NA
lncRNA:CR	7.264273	-1.90467	3.956202	-0.48144	0.630204	NA
lncRNA:CR	0	NA	NA	NA	NA	NA
lncRNA:CR	0	NA	NA	NA	NA	NA
lncRNA:CR	34.10083	1.69146	3.952362	0.427962	0.668679	NA
CG45116	0	NA	NA	NA	NA	NA
lncRNA:CR	0	NA	NA	NA	NA	NA
lncRNA:CR	0.132078	-1.13211	4.069918	-0.27817	0.780885	NA
asRNA:CR4	8.475891	5.42735	3.881918	1.39811	0.16208	NA
Kmn2	0.984306	0.3138	3.972938	0.078984	0.937045	NA
CG45122	0	NA	NA	NA	NA	NA
lncRNA:CR	0	NA	NA	NA	NA	NA
asRNA:CR4	13.07791	1.529441	3.953645	0.386843	0.698872	NA
lncRNA:CR	0	NA	NA	NA	NA	NA
asRNA:CR4	0.715249	-1.88064	4.034336	-0.46616	0.641102	NA
asRNA:CR4	27.02402	-0.38229	3.951361	-0.09675	0.922926	NA
lncRNA:CR	0.6282	1.523021	4.015426	0.379293	0.704471	NA
asRNA:CR4	0	NA	NA	NA	NA	NA
asRNA:CR4	30.40563	-3.24507	3.925567	-0.82665	0.408435	NA
asRNA:CR4	0	NA	NA	NA	NA	NA
asRNA:CR4	1.788123	-2.11324	3.996051	-0.52883	0.596922	NA
asRNA:CR4	0	NA	NA	NA	NA	NA
asRNA:CR4	0	NA	NA	NA	NA	NA
asRNA:CR4	6.502747	3.328933	3.858011	0.862862	0.388213	NA
asRNA:CR4	13.47192	-1.91304	3.953785	-0.48385	0.628491	NA
lncRNA:CR	0.178053	-1.16467	4.075067	-0.2858	0.775028	NA
lncRNA:CR	0.528311	-1.72839	4.011871	-0.43082	0.6666	NA
asRNA:CR4	0.209247	1.042804	4.047837	0.25762	0.7967	NA
lncRNA:CR	0.132078	-1.13211	4.069918	-0.27817	0.780885	NA
asRNA:CR4	0	NA	NA	NA	NA	NA
asRNA:CR4	0	NA	NA	NA	NA	NA
lncRNA:CR	0	NA	NA	NA	NA	NA
CG45154	0	NA	NA	NA	NA	NA
lncRNA:CR	58.58902	1.537214	3.951522	0.389018	0.697263	NA
lncRNA:CR	0	NA	NA	NA	NA	NA
lncRNA:CR	0	NA	NA	NA	NA	NA
lncRNA:CR	22.55783	-1.86002	3.952562	-0.47059	0.637936	NA
asRNA:CR4	0	NA	NA	NA	NA	NA
asRNA:CR4	0	NA	NA	NA	NA	NA
asRNA:CR4	0	NA	NA	NA	NA	NA
asRNA:CR4	5.444396	1.718358	3.960139	0.433914	0.664351	NA
lncRNA:CR	0	NA	NA	NA	NA	NA
lncRNA:CR	0.466186	0.959471	4.033986	0.237847	0.812	NA

asRNA:CR4	1.001866	-0.01426	3.93462	-0.00362	0.997108	NA
lncRNA:CR	0	NA	NA	NA	NA	NA
lncRNA:CR	0	NA	NA	NA	NA	NA
asRNA:CR4	0	NA	NA	NA	NA	NA
lncRNA:CR	0	NA	NA	NA	NA	NA
lncRNA:CR	0	NA	NA	NA	NA	NA
lncRNA:CR	0	NA	NA	NA	NA	NA
asRNA:CR4	5.842825	-1.3066	3.855864	-0.33886	0.734715	NA
lncRNA:CR	0.368779	-0.59727	3.999361	-0.14934	0.881285	NA
lncRNA:CR	0	NA	NA	NA	NA	NA
lncRNA:CR	0.178053	-1.16467	4.075067	-0.2858	0.775028	NA
lncRNA:CR	1.574509	-1.80121	3.864174	-0.46613	0.641122	NA
lncRNA:CR	0	NA	NA	NA	NA	NA
asRNA:CR4	24.1766	-0.41914	3.836896	-0.10924	0.913013	NA
lncRNA:CR	0	NA	NA	NA	NA	NA
lncRNA:CR	0	NA	NA	NA	NA	NA
lncRNA:CR	0.536437	-1.71175	4.038042	-0.42391	0.671635	NA
asRNA:CR4	36.14557	-2.93027	3.918963	-0.74771	0.454632	NA
asRNA:CR4	15.8231	1.086724	3.773657	0.287977	0.773365	NA
lncRNA:CR	0	NA	NA	NA	NA	NA
asRNA:CR4	0	NA	NA	NA	NA	NA
lncRNA:CR	0	NA	NA	NA	NA	NA
asRNA:CR4	10.00445	2.535938	3.852337	0.658286	0.510355	NA
lncRNA:CR	43.16435	-0.24515	3.81209	-0.06431	0.948725	NA
lncRNA:CR	0	NA	NA	NA	NA	NA
Cep89	0	NA	NA	NA	NA	NA
Zmynd10	0	NA	NA	NA	NA	NA
hog	0.942299	1.584447	3.996979	0.396411	0.691802	NA
asRNA:CR4	0	NA	NA	NA	NA	NA
lncRNA:CR	0	NA	NA	NA	NA	NA
lncRNA:CR	0	NA	NA	NA	NA	NA
asRNA:CR4	0	NA	NA	NA	NA	NA
lncRNA:CR	55.95161	3.2621	3.911881	0.833895	0.40434	NA
lncRNA:CR	0	NA	NA	NA	NA	NA
asRNA:CR4	0	NA	NA	NA	NA	NA
asRNA:CR4	0.660388	-1.75977	4.001266	-0.4398	0.660079	NA
CG45207	0	NA	NA	NA	NA	NA
asRNA:CR4	0	NA	NA	NA	NA	NA
asRNA:CR4	0	NA	NA	NA	NA	NA
asRNA:CR4	0	NA	NA	NA	NA	NA
lncRNA:CR	51.87386	-3.06763	3.877788	-0.79108	0.428899	NA
lncRNA:CR	0	NA	NA	NA	NA	NA
asRNA:CR4	0	NA	NA	NA	NA	NA
lncRNA:CR	0	NA	NA	NA	NA	NA
lncRNA:CR	0.178812	-1.08808	4.061735	-0.26789	0.788788	NA
lncRNA:CR	4.292697	1.709267	3.962502	0.431361	0.666206	NA
lncRNA:CR	2.485045	-1.42724	3.888448	-0.36705	0.713585	NA
lncRNA:CR	34.2073	-0.339	3.836652	-0.08836	0.929592	NA
lncRNA:CR	0	NA	NA	NA	NA	NA
mfr	0	NA	NA	NA	NA	NA

CR45227	0.546963	2.042449	4.016608	0.508501	0.611102	NA
lncRNA:CR	0	NA	NA	NA	NA	NA
lncRNA:CR	0	NA	NA	NA	NA	NA
lncRNA:CR	0.178053	-1.16467	4.075067	-0.2858	0.775028	NA
lncRNA:CR	1.046999	1.598136	3.993006	0.400234	0.688984	NA
lncRNA:CR	0	NA	NA	NA	NA	NA
lncRNA:CR	0	NA	NA	NA	NA	NA
lncRNA:CR	59.71745	0.86843	3.668413	0.236732	0.812865	NA
lncRNA:CR	0.566112	-1.1989	3.981989	-0.30108	0.763353	NA
lncRNA:CR	0	NA	NA	NA	NA	NA
lncRNA:CR	0.132078	-1.13211	4.069918	-0.27817	0.780885	NA
lncRNA:CR	0	NA	NA	NA	NA	NA
lncRNA:CR	52.30276	-1.92048	3.951653	-0.48599	0.626971	NA
lncRNA:CR	53.39712	-0.42981	3.835303	-0.11207	0.91077	NA
lncRNA:CR	0	NA	NA	NA	NA	NA
lncRNA:CR	0	NA	NA	NA	NA	NA
lncRNA:CR	3.034074	1.498215	3.962342	0.378114	0.705346	NA
lncRNA:CR	0	NA	NA	NA	NA	NA
lncRNA:CR	0	NA	NA	NA	NA	NA
lncRNA:CR	0	NA	NA	NA	NA	NA
lncRNA:CR	0.356107	-1.47099	4.037764	-0.36431	0.715628	NA
lncRNA:CR	0	NA	NA	NA	NA	NA
lncRNA:CR	0	NA	NA	NA	NA	NA
lncRNA:CR	0.104623	0.639075	4.067674	0.157111	0.875158	NA
lncRNA:CR	0.178053	-1.16467	4.075067	-0.2858	0.775028	NA
asRNA:CR4	0	NA	NA	NA	NA	NA
lncRNA:CR	0.233093	0.692656	4.076246	0.169925	0.865069	NA
lncRNA:CR	0	NA	NA	NA	NA	NA
asRNA:CR4	0	NA	NA	NA	NA	NA
lncRNA:CR	0	NA	NA	NA	NA	NA
lncRNA:CR	0.660388	-1.75977	4.001266	-0.4398	0.660079	NA
asRNA:CR4	14.91797	1.17086	3.95405	0.296117	0.767141	NA
lncRNA:CR	0	NA	NA	NA	NA	NA
lncRNA:CR	0	NA	NA	NA	NA	NA
lncRNA:CR	17.79899	1.743127	3.953807	0.440873	0.659305	NA
asRNA:CR4	0	NA	NA	NA	NA	NA
lncRNA:CR	0	NA	NA	NA	NA	NA
asRNA:CR4	0	NA	NA	NA	NA	NA
asRNA:CR4	0.178053	-1.16467	4.075067	-0.2858	0.775028	NA
CG45273	0	NA	NA	NA	NA	NA
lncRNA:CR	59.64785	-1.7063	3.951551	-0.4318	0.665883	NA
asRNA:CR4	0	NA	NA	NA	NA	NA
lncRNA:CR	0	NA	NA	NA	NA	NA
lncRNA:CR	1.089612	1.039859	3.940679	0.263878	0.791874	NA
lncRNA:CR	0	NA	NA	NA	NA	NA
lncRNA:CR	1.188699	-1.8233	3.980741	-0.45803	0.646931	NA
lncRNA:CR	0	NA	NA	NA	NA	NA
lncRNA:CR	0	NA	NA	NA	NA	NA
lncRNA:CR	0.233093	0.692656	4.076246	0.169925	0.865069	NA
lncRNA:CR	11.70063	-4.87591	3.85987	-1.26323	0.206506	NA

lncRNA:CR	0	NA	NA	NA	NA	NA
lncRNA:CR	0.356107	-1.47099	4.037764	-0.36431	0.715628	NA
lncRNA:CR	0	NA	NA	NA	NA	NA
lncRNA:CR	9.79293	-1.69634	3.954799	-0.42893	0.667972	NA
lncRNA:CR	0	NA	NA	NA	NA	NA
lncRNA:CR	0	NA	NA	NA	NA	NA
lncRNA:CR	0	NA	NA	NA	NA	NA
lncRNA:CR	0	NA	NA	NA	NA	NA
lncRNA:CR	0	NA	NA	NA	NA	NA
lncRNA:CR	0	NA	NA	NA	NA	NA
lncRNA:CR	0	NA	NA	NA	NA	NA
lncRNA:CR	0	NA	NA	NA	NA	NA
lncRNA:CR	0.282753	-0.25398	4.020204	-0.06318	0.949626	NA
lncRNA:CR	0	NA	NA	NA	NA	NA
asRNA:CR4	0	NA	NA	NA	NA	NA
lncRNA:CR	0	NA	NA	NA	NA	NA
lncRNA:CR	0	NA	NA	NA	NA	NA
lncRNA:CR	0.282753	-0.25398	4.020204	-0.06318	0.949626	NA
lncRNA:CR	0	NA	NA	NA	NA	NA
asRNA:CR4	0	NA	NA	NA	NA	NA
lncRNA:CR	0	NA	NA	NA	NA	NA
CG45307	0	NA	NA	NA	NA	NA
lncRNA:CR	0.104623	0.639075	4.067674	0.157111	0.875158	NA
lncRNA:CR	0	NA	NA	NA	NA	NA
lncRNA:CR	0.356107	-1.47099	4.037764	-0.36431	0.715628	NA
lncRNA:CR	0	NA	NA	NA	NA	NA
lncRNA:CR	0	NA	NA	NA	NA	NA
CR45315	0.178053	-1.16467	4.075067	-0.2858	0.775028	NA
lncRNA:CR	0	NA	NA	NA	NA	NA
lncRNA:CR	0	NA	NA	NA	NA	NA
lncRNA:CR	0	NA	NA	NA	NA	NA
lncRNA:CR	0	NA	NA	NA	NA	NA
lncRNA:CR	0	NA	NA	NA	NA	NA
lncRNA:CR	0	NA	NA	NA	NA	NA
lncRNA:CR	0	NA	NA	NA	NA	NA
lncRNA:CR	1.879809	1.619289	3.898416	0.415371	0.67787	NA
lncRNA:CR	3.51509	-3.04388	3.928899	-0.77474	0.438493	NA
asRNA:CR4	0.69928	1.019866	4.009445	0.254366	0.799213	NA
lncRNA:CR	0	NA	NA	NA	NA	NA
lncRNA:CR	0	NA	NA	NA	NA	NA
asRNA:CR4	0	NA	NA	NA	NA	NA
lncRNA:CR	0	NA	NA	NA	NA	NA
lncRNA:CR	0	NA	NA	NA	NA	NA
lncRNA:CR	0	NA	NA	NA	NA	NA
lncRNA:CR	0	NA	NA	NA	NA	NA
lncRNA:CR	0	NA	NA	NA	NA	NA
lncRNA:CR	0	NA	NA	NA	NA	NA
lncRNA:CR	34.25344	8.910206	3.845848	2.316838	0.020513	NA
asRNA:CR4	0	NA	NA	NA	NA	NA
asRNA:CR4	0	NA	NA	NA	NA	NA

asRNA:CR4	0	NA	NA	NA	NA	NA
lncRNA:CR	0	NA	NA	NA	NA	NA
lncRNA:CR	0	NA	NA	NA	NA	NA
lncRNA:CR	0	NA	NA	NA	NA	NA
lncRNA:CR	0.807511	1.368689	3.976452	0.344199	0.730697	NA
lncRNA:CR	0	NA	NA	NA	NA	NA
asRNA:CR4	17.43425	-1.91531	3.953136	-0.4845	0.628029	NA
lncRNA:CR	22.55783	-1.86002	3.952562	-0.47059	0.637936	NA
lncRNA:CR	0	NA	NA	NA	NA	NA
lncRNA:CR	0	NA	NA	NA	NA	NA
lncRNA:CR	0.132078	-1.13211	4.069918	-0.27817	0.780885	NA
lncRNA:CR	0	NA	NA	NA	NA	NA
lncRNA:CR	0.132078	-1.13211	4.069918	-0.27817	0.780885	NA
lncRNA:CR	0	NA	NA	NA	NA	NA
lncRNA:CR	0	NA	NA	NA	NA	NA
lncRNA:CR	0	NA	NA	NA	NA	NA
lncRNA:CR	0.236701	-0.24276	4.020028	-0.06039	0.951846	NA
lncRNA:CR	2.053389	-0.89329	3.886943	-0.22982	0.818233	NA
lncRNA:CR	0	NA	NA	NA	NA	NA
lncRNA:CR	0	NA	NA	NA	NA	NA
lncRNA:CR	12.02044	-0.34591	3.842436	-0.09002	0.928269	NA
asRNA:CR4	0	NA	NA	NA	NA	NA
asRNA:CR4	0	NA	NA	NA	NA	NA
lncRNA:CR	0	NA	NA	NA	NA	NA
lncRNA:CR	0	NA	NA	NA	NA	NA
asRNA:CR4	0	NA	NA	NA	NA	NA
lncRNA:CR	0	NA	NA	NA	NA	NA
lncRNA:CR	0	NA	NA	NA	NA	NA
lncRNA:CR	0	NA	NA	NA	NA	NA
lncRNA:CR	26.28346	-1.91792	3.952389	-0.48526	0.627494	NA
Cenp-C	0.178053	-1.16467	4.075067	-0.2858	0.775028	NA
asRNA:CR4	0	NA	NA	NA	NA	NA
lncRNA:CR	0	NA	NA	NA	NA	NA
lncRNA:CR	1.311343	-1.38229	3.935643	-0.35122	0.72542	NA
lncRNA:CR	0	NA	NA	NA	NA	NA
lncRNA:CR	12.66861	2.946279	3.925581	0.750533	0.452933	NA
lncRNA:CR	0	NA	NA	NA	NA	NA
lncRNA:CR	0.264155	-1.54099	4.048976	-0.38059	0.703509	NA
asRNA:CR4	0	NA	NA	NA	NA	NA
CR45385	0.178053	-1.16467	4.075067	-0.2858	0.775028	NA
lncRNA:CR	17.06609	1.74264	3.95393	0.440736	0.659404	NA
lncRNA:CR	0	NA	NA	NA	NA	NA
lncRNA:CR	0	NA	NA	NA	NA	NA
lncRNA:CR	0	NA	NA	NA	NA	NA
lncRNA:CR	0	NA	NA	NA	NA	NA
CR45391	0	NA	NA	NA	NA	NA
lncRNA:CR	0.1047	0.613261	4.062799	0.150945	0.880019	NA
lncRNA:CR	0	NA	NA	NA	NA	NA
lncRNA:CR	0	NA	NA	NA	NA	NA
lncRNA:CR	0	NA	NA	NA	NA	NA

asRNA:CR4	0.418493	1.313669	4.019283	0.326842	0.743788	NA
asRNA:CR4	0	NA	NA	NA	NA	NA
lncRNA:CR	0	NA	NA	NA	NA	NA
lncRNA:CR	0.104623	0.639075	4.067674	0.157111	0.875158	NA
lncRNA:CR	0	NA	NA	NA	NA	NA
lncRNA:CR	0	NA	NA	NA	NA	NA
lncRNA:CR	0	NA	NA	NA	NA	NA
lncRNA:CR	31.03826	-1.91871	3.952163	-0.48548	0.627334	NA
lncRNA:CR	4.470307	-2.20441	3.970887	-0.55514	0.578797	NA
lncRNA:CR	0	NA	NA	NA	NA	NA
lncRNA:CR	0.1047	0.613261	4.062799	0.150945	0.880019	NA
lncRNA:CR	0	NA	NA	NA	NA	NA
lncRNA:CR	0.178053	-1.16467	4.075067	-0.2858	0.775028	NA
lncRNA:CR	0	NA	NA	NA	NA	NA
lncRNA:CR	20.91648	-5.61529	3.897424	-1.44077	0.14965	NA
lncRNA:CR	0.178053	-1.16467	4.075067	-0.2858	0.775028	NA
CG45413	0	NA	NA	NA	NA	NA
lncRNA:CR	5.811418	-1.90022	3.957496	-0.48016	0.631116	NA
lncRNA:CR	0	NA	NA	NA	NA	NA
lncRNA:CR	0	NA	NA	NA	NA	NA
lncRNA:CR	31.54074	-1.71799	3.812837	-0.45058	0.652293	NA
lncRNA:CR	19.78865	-8.32814	3.846501	-2.16512	0.030378	NA
lncRNA:CR	0	NA	NA	NA	NA	NA
lncRNA:CR	0	NA	NA	NA	NA	NA
lncRNA:CR	0	NA	NA	NA	NA	NA
lncRNA:CR	0	NA	NA	NA	NA	NA
lncRNA:CR	0	NA	NA	NA	NA	NA
lncRNA:CR	0	NA	NA	NA	NA	NA
lncRNA:CR	0	NA	NA	NA	NA	NA
lncRNA:CR	0	NA	NA	NA	NA	NA
CG45428	0	NA	NA	NA	NA	NA
lncRNA:CR	0	NA	NA	NA	NA	NA
lncRNA:CR	1.981645	1.940655	3.957663	0.490354	0.623884	NA
lncRNA:CR	0.341324	0.112818	4.000379	0.028202	0.977501	NA
lncRNA:CR	12.24092	1.528761	3.953831	0.386653	0.699013	NA
lncRNA:CR	0	NA	NA	NA	NA	NA
lncRNA:CR	0	NA	NA	NA	NA	NA
lncRNA:CR	27.4313	2.96645	3.921008	0.756553	0.449318	NA
lncRNA:CR	0	NA	NA	NA	NA	NA
lncRNA:CR	0	NA	NA	NA	NA	NA
lncRNA:CR	0	NA	NA	NA	NA	NA
lncRNA:CR	0	NA	NA	NA	NA	NA
lncRNA:CR	0	NA	NA	NA	NA	NA
lncRNA:CR	0	NA	NA	NA	NA	NA
lncRNA:CR	0	NA	NA	NA	NA	NA
lncRNA:CR	2.097839	1.11873	3.972242	0.281637	0.778222	NA
lncRNA:CR	0	NA	NA	NA	NA	NA
asRNA:CR4	0	NA	NA	NA	NA	NA
lncRNA:CR	0	NA	NA	NA	NA	NA
lncRNA:CR	0	NA	NA	NA	NA	NA

lncRNA:CR	0	NA	NA	NA	NA	NA
lncRNA:CR	0	NA	NA	NA	NA	NA
lncRNA:CR	0.178053	-1.16467	4.075067	-0.2858	0.775028	NA
lncRNA:CR	0	NA	NA	NA	NA	NA
lncRNA:CR	0.178053	-1.16467	4.075067	-0.2858	0.775028	NA
lncRNA:CR	0	NA	NA	NA	NA	NA
lncRNA:CR	0	NA	NA	NA	NA	NA
lncRNA:CR	0	NA	NA	NA	NA	NA
lncRNA:CR	0	NA	NA	NA	NA	NA
lncRNA:CR	0	NA	NA	NA	NA	NA
CR45458	4.467006	0.772124	3.77768	0.204391	0.838048	NA
lncRNA:CR	43.67991	0.357798	3.834646	0.093307	0.92566	NA
lncRNA:CR	0	NA	NA	NA	NA	NA
lncRNA:CR	20.20579	-2.26351	3.955621	-0.57223	0.567168	NA
lncRNA:CR	25.61656	3.064656	3.899904	0.785829	0.431968	NA
lncRNA:CR	0	NA	NA	NA	NA	NA
asRNA:CR4	0	NA	NA	NA	NA	NA
asRNA:CR4	5.758496	1.720238	3.959653	0.434441	0.663968	NA
asRNA:CR4	0	NA	NA	NA	NA	NA
asRNA:CR4	15.87189	0.102102	3.878599	0.026324	0.978999	NA
CR45470	4.173233	-1.2228	3.859453	-0.31683	0.75137	NA
lncRNA:CR	4.117185	-4.39498	3.860397	-1.13848	0.254921	NA
lncRNA:CR	4.027089	2.292019	3.823753	0.599416	0.548895	NA
lncRNA:CR	0	NA	NA	NA	NA	NA
lncRNA:CR	0	NA	NA	NA	NA	NA
lncRNA:CR	0	NA	NA	NA	NA	NA
asRNA:CR4	18.42939	1.302761	3.855833	0.337867	0.735463	NA
lncRNA:CR	4.905149	-4.66714	3.94586	-1.18279	0.23689	NA
asRNA:CR4	0	NA	NA	NA	NA	NA
lncRNA:CR	0.178053	-1.16467	4.075067	-0.2858	0.775028	NA
lncRNA:CR	0	NA	NA	NA	NA	NA
lncRNA:CR	0	NA	NA	NA	NA	NA
asRNA:CR4	10.7016	-4.57918	3.854492	-1.18801	0.234829	NA
CG45486	0	NA	NA	NA	NA	NA
CG45487	0	NA	NA	NA	NA	NA
CG45488	0	NA	NA	NA	NA	NA
CG45489	0	NA	NA	NA	NA	NA
CG45490	0	NA	NA	NA	NA	NA
CG45491	0	NA	NA	NA	NA	NA
CG45492	0	NA	NA	NA	NA	NA
CG45493	0	NA	NA	NA	NA	NA
CG45494	0	NA	NA	NA	NA	NA
CR45495	0	NA	NA	NA	NA	NA
CR45496	0	NA	NA	NA	NA	NA
CR45497	0	NA	NA	NA	NA	NA
CR45498	0	NA	NA	NA	NA	NA
CR45499	0	NA	NA	NA	NA	NA
lncRNA:CR	0	NA	NA	NA	NA	NA
asRNA:CR4	0	NA	NA	NA	NA	NA
lncRNA:CR	1.165466	1.076041	3.987865	0.269829	0.787292	NA

lncRNA:CR	0	NA	NA	NA	NA	NA
lncRNA:CR	0	NA	NA	NA	NA	NA
lncRNA:CR	0	NA	NA	NA	NA	NA
lncRNA:CR	0	NA	NA	NA	NA	NA
lncRNA:CR	0.178053	-1.16467	4.075067	-0.2858	0.775028	NA
lncRNA:CR	0	NA	NA	NA	NA	NA
lncRNA:CR	0	NA	NA	NA	NA	NA
lncRNA:CR	0	NA	NA	NA	NA	NA
lncRNA:CR	0	NA	NA	NA	NA	NA
lncRNA:CR	0	NA	NA	NA	NA	NA
lncRNA:CR	0	NA	NA	NA	NA	NA
lncRNA:CR	0	NA	NA	NA	NA	NA
lncRNA:CR	14.1377	0.321112	3.845108	0.083512	0.933445	NA
lncRNA:CR	0	NA	NA	NA	NA	NA
asRNA:CR4	0.104623	0.639075	4.067674	0.157111	0.875158	NA
lncRNA:CR	0	NA	NA	NA	NA	NA
asRNA:CR4	22.7084	-3.5958	3.920039	-0.91729	0.358993	NA
lncRNA:CR	0	NA	NA	NA	NA	NA
lncRNA:CR	0	NA	NA	NA	NA	NA
lncRNA:CR	0	NA	NA	NA	NA	NA
lncRNA:CR	0	NA	NA	NA	NA	NA
asRNA:CR4	0	NA	NA	NA	NA	NA
lncRNA:CR	0.178812	-1.08808	4.061735	-0.26789	0.788788	NA
asRNA:CR4	0.466186	0.959471	4.033986	0.237847	0.812	NA
lncRNA:CR	0	NA	NA	NA	NA	NA
lncRNA:CR	0	NA	NA	NA	NA	NA
lncRNA:CR	0.178053	-1.16467	4.075067	-0.2858	0.775028	NA
lncRNA:CR	0	NA	NA	NA	NA	NA
lncRNA:CR	0	NA	NA	NA	NA	NA
lncRNA:CR	0.357625	-1.47707	4.044794	-0.36518	0.714979	NA
asRNA:CR4	0	NA	NA	NA	NA	NA
lncRNA:CR	0.356107	-1.47099	4.037764	-0.36431	0.715628	NA
lncRNA:CR	0	NA	NA	NA	NA	NA
lncRNA:CR	0	NA	NA	NA	NA	NA
lncRNA:CR	0	NA	NA	NA	NA	NA
lncRNA:CR	0	NA	NA	NA	NA	NA
CG45544	0	NA	NA	NA	NA	NA
CG45545	0	NA	NA	NA	NA	NA
CG45546	9.773749	-1.90931	3.95486	-0.48278	0.629255	NA
lncRNA:CR	20.86827	-1.91658	3.952771	-0.48487	0.627769	NA
CR45548	0	NA	NA	NA	NA	NA
asRNA:CR4	21.27488	0.66979	3.951899	0.169486	0.865415	NA
lncRNA:CR	0	NA	NA	NA	NA	NA
lncRNA:CR	3.871196	-3.05496	3.926716	-0.77799	0.436573	NA
lncRNA:CR	0.1047	0.613261	4.062799	0.150945	0.880019	NA
lncRNA:CR	0	NA	NA	NA	NA	NA
lncRNA:CR	0	NA	NA	NA	NA	NA
lncRNA:CR	0	NA	NA	NA	NA	NA
lncRNA:CR	8.674033	2.146231	3.887461	0.552091	0.580886	NA
lncRNA:CR	0	NA	NA	NA	NA	NA

lncRNA:CR	13.2445	-6.27204	3.895023	-1.61027	0.107339	NA
lncRNA:CR	0	NA	NA	NA	NA	NA
lncRNA:CR	0	NA	NA	NA	NA	NA
lncRNA:CR	0	NA	NA	NA	NA	NA
lncRNA:CR	0	NA	NA	NA	NA	NA
lncRNA:CR	0	NA	NA	NA	NA	NA
lncRNA:CR	0	NA	NA	NA	NA	NA
lncRNA:CR	17.75203	-0.87337	3.952279	-0.22098	0.825109	NA
lncRNA:CR	0	NA	NA	NA	NA	NA
lncRNA:CR	0	NA	NA	NA	NA	NA
lncRNA:CR	0	NA	NA	NA	NA	NA
lncRNA:CR	0	NA	NA	NA	NA	NA
lncRNA:CR	0	NA	NA	NA	NA	NA
lncRNA:CR	0	NA	NA	NA	NA	NA
lncRNA:CR	0.53416	-1.53481	4.012395	-0.38252	0.702078	NA
lncRNA:CR	0	NA	NA	NA	NA	NA
lncRNA:CR	0	NA	NA	NA	NA	NA
lncRNA:CR	0.282677	-0.09599	4.057934	-0.02366	0.981127	NA
lncRNA:CR	0	NA	NA	NA	NA	NA
lncRNA:CR	0.1047	0.613261	4.062799	0.150945	0.880019	NA
lncRNA:CR	26.46968	1.534479	3.952266	0.388253	0.697829	NA
lncRNA:CR	0.178053	-1.16467	4.075067	-0.2858	0.775028	NA
lncRNA:CR	0	NA	NA	NA	NA	NA
lncRNA:CR	0	NA	NA	NA	NA	NA
lncRNA:CR	0	NA	NA	NA	NA	NA
lncRNA:CR	0	NA	NA	NA	NA	NA
lncRNA:CR	0.178053	-1.16467	4.075067	-0.2858	0.775028	NA
lncRNA:CR	0	NA	NA	NA	NA	NA
lncRNA:CR	0	NA	NA	NA	NA	NA
lncRNA:CR	0	NA	NA	NA	NA	NA
lncRNA:CR	1.072874	-2.0339	4.019685	-0.50598	0.612867	NA
lncRNA:CR	0	NA	NA	NA	NA	NA
lncRNA:CR	0	NA	NA	NA	NA	NA
lncRNA:CR	0	NA	NA	NA	NA	NA
lncRNA:CR	0	NA	NA	NA	NA	NA
lncRNA:CR	0	NA	NA	NA	NA	NA
lncRNA:CR	43.37157	-0.27373	3.950641	-0.06929	0.94476	NA
lncRNA:CR	0	NA	NA	NA	NA	NA
lncRNA:CR	0	NA	NA	NA	NA	NA
lncRNA:CR	0	NA	NA	NA	NA	NA
asRNA:CR4	0	NA	NA	NA	NA	NA
lncRNA:CR	5.831849	1.457586	3.95798	0.368265	0.712676	NA
CG45603	3.350398	1.697551	3.965576	0.428072	0.668599	NA
asRNA:CR4	0.337793	1.451235	4.039844	0.35923	0.719423	NA
lncRNA:CR	0	NA	NA	NA	NA	NA
lncRNA:CR	0	NA	NA	NA	NA	NA
lncRNA:CR	0	NA	NA	NA	NA	NA
lncRNA:CR	0	NA	NA	NA	NA	NA
lncRNA:CR	0	NA	NA	NA	NA	NA
lncRNA:CR	0	NA	NA	NA	NA	NA

asRNA:CR4	16.01909	1.741867	3.954124	0.440519	0.659561	NA
asRNA:CR4	6.281996	1.722971	3.958948	0.435209	0.66341	NA
lncRNA:CR	0	NA	NA	NA	NA	NA
lncRNA:CR	9.323729	1.165404	3.955911	0.294598	0.768301	NA
lncRNA:CR	2.113243	-1.8635	3.968379	-0.46959	0.63865	NA
lncRNA:CR	3.993031	0.347252	3.851261	0.090166	0.928155	NA
lncRNA:CR	8.452972	-1.9072	3.955468	-0.48217	0.629686	NA
lncRNA:CR	0	NA	NA	NA	NA	NA
lncRNA:CR	0	NA	NA	NA	NA	NA
asRNA:CR4	0	NA	NA	NA	NA	NA
lncRNA:CR	0	NA	NA	NA	NA	NA
asRNA:CR4	0	NA	NA	NA	NA	NA
lncRNA:CR	0	NA	NA	NA	NA	NA
lncRNA:CR	10.25653	-2.77849	3.836392	-0.72425	0.468914	NA
lncRNA:CR	2.512798	1.684724	3.970151	0.424348	0.671312	NA
lncRNA:CR	0	NA	NA	NA	NA	NA
lncRNA:CR	4.18493	1.509067	3.95929	0.381146	0.703095	NA
asRNA:CR4	0	NA	NA	NA	NA	NA
lncRNA:CR	0	NA	NA	NA	NA	NA
lncRNA:CR	0	NA	NA	NA	NA	NA
lncRNA:CR	0	NA	NA	NA	NA	NA
lncRNA:CR	0.3141	1.240997	4.03841	0.307299	0.758616	NA
lncRNA:CR	0	NA	NA	NA	NA	NA
lncRNA:CR	0.62774	1.374224	3.999506	0.343599	0.731148	NA
lncRNA:CR	0	NA	NA	NA	NA	NA
lncRNA:CR	0	NA	NA	NA	NA	NA
lncRNA:CR	0	NA	NA	NA	NA	NA
lncRNA:CR	0	NA	NA	NA	NA	NA
lncRNA:CR	0	NA	NA	NA	NA	NA
lncRNA:CR	0	NA	NA	NA	NA	NA
lncRNA:CR	0	NA	NA	NA	NA	NA
lncRNA:CR	4.4988	1.511101	3.958721	0.381714	0.702673	NA
lncRNA:CR	0	NA	NA	NA	NA	NA
lncRNA:CR	0.178053	-1.16467	4.075067	-0.2858	0.775028	NA
lncRNA:CR	0.365171	-0.43584	4.056359	-0.10745	0.914434	NA
lncRNA:CR	0	NA	NA	NA	NA	NA
asRNA:CR4	0	NA	NA	NA	NA	NA
lncRNA:CR	0	NA	NA	NA	NA	NA
lncRNA:CR	43.6302	-2.27354	3.953114	-0.57513	0.565206	NA
lncRNA:CR	0	NA	NA	NA	NA	NA
lncRNA:CR	0	NA	NA	NA	NA	NA
lncRNA:CR	0	NA	NA	NA	NA	NA
lncRNA:CR	0	NA	NA	NA	NA	NA
lncRNA:CR	0	NA	NA	NA	NA	NA
lncRNA:CR	0	NA	NA	NA	NA	NA
lncRNA:CR	0	NA	NA	NA	NA	NA
lncRNA:CR	0	NA	NA	NA	NA	NA
lncRNA:CR	37.04619	6.605198	3.827551	1.725698	0.084402	NA
lncRNA:CR	0	NA	NA	NA	NA	NA
lncRNA:CR	17.58959	1.742992	3.953841	0.440835	0.659332	NA

asRNA:CR4	0	NA	NA	NA	NA	NA
lncRNA:CR	0	NA	NA	NA	NA	NA
lncRNA:CR	0	NA	NA	NA	NA	NA
lncRNA:CR	0	NA	NA	NA	NA	NA
lncRNA:CR	0	NA	NA	NA	NA	NA
lncRNA:CR	1.361099	1.628413	3.98439	0.408698	0.682761	NA
lncRNA:CR	2.451623	0.000923	3.913399	0.000236	0.999812	NA
lncRNA:CR	0	NA	NA	NA	NA	NA
lncRNA:CR	0	NA	NA	NA	NA	NA
lncRNA:CR	37.90137	1.749223	3.952277	0.442586	0.658065	NA
lncRNA:CR	3.010332	-1.56487	3.960938	-0.39507	0.692788	NA
lncRNA:CR	0	NA	NA	NA	NA	NA
lncRNA:CR	0	NA	NA	NA	NA	NA
lncRNA:CR	0	NA	NA	NA	NA	NA
lncRNA:CR	0	NA	NA	NA	NA	NA
lncRNA:CR	30.60546	1.052815	3.952339	0.266378	0.789948	NA
asRNA:CR4	0	NA	NA	NA	NA	NA
lncRNA:CR	3.036298	1.692169	3.966999	0.426562	0.669699	NA
lncRNA:CR	1.602479	-1.64094	3.97352	-0.41297	0.67963	NA
asRNA:CR4	0	NA	NA	NA	NA	NA
lncRNA:CR	0.178053	-1.16467	4.075067	-0.2858	0.775028	NA
lncRNA:CR	0	NA	NA	NA	NA	NA
lncRNA:CR	0	NA	NA	NA	NA	NA
CG45690	0	NA	NA	NA	NA	NA
CG45691	0.1047	0.613261	4.062799	0.150945	0.880019	NA
lncRNA:CR	0	NA	NA	NA	NA	NA
lncRNA:CR	1.133183	-2.25669	3.937492	-0.57313	0.566557	NA
lncRNA:CR	0	NA	NA	NA	NA	NA
lncRNA:CR	0	NA	NA	NA	NA	NA
lncRNA:CR	0	NA	NA	NA	NA	NA
lncRNA:CR	0.534919	-2.31746	4.015737	-0.5771	0.563875	NA
lncRNA:CR	0	NA	NA	NA	NA	NA
lncRNA:CR	0.178812	-1.08808	4.061735	-0.26789	0.788788	NA
lncRNA:CR	0	NA	NA	NA	NA	NA
asRNA:CR4	0	NA	NA	NA	NA	NA
lncRNA:CR	0.1047	0.613261	4.062799	0.150945	0.880019	NA
lncRNA:CR	0	NA	NA	NA	NA	NA
lncRNA:CR	0.356107	-1.47099	4.037764	-0.36431	0.715628	NA
lncRNA:CR	0	NA	NA	NA	NA	NA
lncRNA:CR	0.104623	0.639075	4.067674	0.157111	0.875158	NA
lncRNA:CR	13.07791	1.529441	3.953645	0.386843	0.698872	NA
lncRNA:CR	0	NA	NA	NA	NA	NA
lncRNA:CR	0	NA	NA	NA	NA	NA
asRNA:CR4	0.62774	1.374224	3.999506	0.343599	0.731148	NA
asRNA:CR4	0	NA	NA	NA	NA	NA
asRNA:CR4	0	NA	NA	NA	NA	NA
asRNA:CR4	0	NA	NA	NA	NA	NA
CR45716	0	NA	NA	NA	NA	NA
lncRNA:CR	0	NA	NA	NA	NA	NA
lncRNA:CR	0	NA	NA	NA	NA	NA

lncRNA:CR	0	NA	NA	NA	NA	NA
lncRNA:CR	0	NA	NA	NA	NA	NA
lncRNA:CR	0.178053	-1.16467	4.075067	-0.2858	0.775028	NA
lncRNA:CR	0	NA	NA	NA	NA	NA
lncRNA:CR	0	NA	NA	NA	NA	NA
lncRNA:CR	0.357625	-1.47707	4.044794	-0.36518	0.714979	NA
lncRNA:CR	0	NA	NA	NA	NA	NA
lncRNA:CR	0	NA	NA	NA	NA	NA
lncRNA:CR	0	NA	NA	NA	NA	NA
lncRNA:CR	0	NA	NA	NA	NA	NA
lncRNA:CR	0	NA	NA	NA	NA	NA
lncRNA:CR	0	NA	NA	NA	NA	NA
lncRNA:CR	0	NA	NA	NA	NA	NA
lncRNA:CR	6.177296	1.72246	3.95908	0.435066	0.663515	NA
lncRNA:CR	0	NA	NA	NA	NA	NA
lncRNA:CR	0	NA	NA	NA	NA	NA
lncRNA:CR	0.178053	-1.16467	4.075067	-0.2858	0.775028	NA
lncRNA:CR	0	NA	NA	NA	NA	NA
asRNA:CR4	0	NA	NA	NA	NA	NA
lncRNA:CR	0	NA	NA	NA	NA	NA
lncRNA:CR	0	NA	NA	NA	NA	NA
lncRNA:CR	0	NA	NA	NA	NA	NA
lncRNA:CR	0	NA	NA	NA	NA	NA
lncRNA:CR	0.713731	-2.6372	4.003426	-0.65874	0.510065	NA
lncRNA:CR	0	NA	NA	NA	NA	NA
lncRNA:CR	0	NA	NA	NA	NA	NA
lncRNA:CR	0	NA	NA	NA	NA	NA
lncRNA:CR	0	NA	NA	NA	NA	NA
lncRNA:CR	0	NA	NA	NA	NA	NA
lncRNA:CR	0	NA	NA	NA	NA	NA
lncRNA:CR	0.5235	1.490714	4.02555	0.370313	0.711149	NA
lncRNA:TS	0.233093	0.692656	4.076246	0.169925	0.865069	NA
lncRNA:CR	0	NA	NA	NA	NA	NA
lncRNA:CR	0	NA	NA	NA	NA	NA
lncRNA:CR	0	NA	NA	NA	NA	NA
lncRNA:CR	0	NA	NA	NA	NA	NA
lncRNA:CR	0	NA	NA	NA	NA	NA
lncRNA:CR	0	NA	NA	NA	NA	NA
lncRNA:CR	0	NA	NA	NA	NA	NA
Acp33A	45.58164	-1.70567	3.95175	-0.43162	0.666015	NA
p38c	0	NA	NA	NA	NA	NA
squ	0.894061	-2.00006	4.030294	-0.49626	0.619714	NA
JYalpha	0.104623	0.639075	4.067674	0.157111	0.875158	NA
mil	0	NA	NA	NA	NA	NA
Ubc87F	0	NA	NA	NA	NA	NA
lncRNA:CR	0	NA	NA	NA	NA	NA
lncRNA:CR	48.79017	1.750442	3.951972	0.442929	0.657817	NA
AOX1	26.87653	1.675648	3.95273	0.423922	0.671623	NA
CG45764	0	NA	NA	NA	NA	NA
CG45765	0	NA	NA	NA	NA	NA

CG45766	0	NA	NA	NA	NA	NA
CG45770	0	NA	NA	NA	NA	NA
CR45771	0	NA	NA	NA	NA	NA
CR45772	0	NA	NA	NA	NA	NA
CR45773	0	NA	NA	NA	NA	NA
CR45775	0	NA	NA	NA	NA	NA
CR45776	0	NA	NA	NA	NA	NA
CR45777	0	NA	NA	NA	NA	NA
asRNA:CR4	0	NA	NA	NA	NA	NA
CR45779	0	NA	NA	NA	NA	NA
CR45780	0	NA	NA	NA	NA	NA
kl-3	0.310131	-1.94616	4.045058	-0.48112	0.630431	NA
kl-5	0.209323	1.414133	4.032409	0.350692	0.725819	NA
chp	14.02979	1.740091	3.954574	0.44002	0.659923	NA
lncRNA:CR	0	NA	NA	NA	NA	NA
asRNA:CR4	17.69135	-2.63201	3.819493	-0.6891	0.490761	NA
lncRNA:CR	0	NA	NA	NA	NA	NA
lncRNA:CR	0	NA	NA	NA	NA	NA
lncRNA:CR	0	NA	NA	NA	NA	NA
lncRNA:CR	0	NA	NA	NA	NA	NA
Su(Ste):CR	8.63859	2.129376	3.882221	0.548494	0.583353	NA
Su(Ste):CR	0.104623	0.639075	4.067674	0.157111	0.875158	NA
Su(Ste):CR	0.209247	1.042804	4.047837	0.25762	0.7967	NA
asRNA:CR4	0	NA	NA	NA	NA	NA
asRNA:CR4	4.751466	-4.28836	3.925119	-1.09254	0.274595	NA
WDY	0	NA	NA	NA	NA	NA
asRNA:CR4	0	NA	NA	NA	NA	NA
asRNA:CR4	0.178812	-1.08808	4.061735	-0.26789	0.788788	NA
asRNA:CR4	40.83052	1.504801	3.951769	0.380792	0.703358	NA
lncRNA:CR	0	NA	NA	NA	NA	NA
lncRNA:CR	15.74203	2.861944	3.86825	0.739855	0.459388	NA
asRNA:CR4	0	NA	NA	NA	NA	NA
asRNA:CR4	0.357625	-1.47707	4.044794	-0.36518	0.714979	NA
lncRNA:CR	12.05347	5.69107	3.888842	1.463436	0.143348	NA
lncRNA:CR	46.03423	1.536597	3.95169	0.388846	0.69739	NA
lncRNA:CR	0	NA	NA	NA	NA	NA
lncRNA:CR	0	NA	NA	NA	NA	NA
asRNA:CR4	0	NA	NA	NA	NA	NA
asRNA:CR4	0	NA	NA	NA	NA	NA
asRNA:CR4	0.357625	-1.47707	4.044794	-0.36518	0.714979	NA
asRNA:CR4	11.75152	-1.69829	3.954156	-0.42949	0.667564	NA
lncRNA:CR	2.136639	-1.65662	3.968112	-0.41748	0.676326	NA
lncRNA:CR	0	NA	NA	NA	NA	NA
asRNA:CR4	0.675586	2.168561	4.004381	0.541547	0.58813	NA
lncRNA:CR	6.759704	1.159951	3.957776	0.293082	0.76946	NA
lncRNA:CR	0.233093	0.692656	4.076246	0.169925	0.865069	NA
lncRNA:CR	0	NA	NA	NA	NA	NA
asRNA:CR4	0	NA	NA	NA	NA	NA
asRNA:CR4	0	NA	NA	NA	NA	NA
asRNA:CR4	0	NA	NA	NA	NA	NA

asRNA:CR4	0.233093	0.692656	4.076246	0.169925	0.865069	NA
asRNA:CR4	0	NA	NA	NA	NA	NA
asRNA:CR4	26.36506	1.534459	3.952272	0.388247	0.697833	NA
lncRNA:CR	15.07537	-0.74317	3.839833	-0.19354	0.846534	NA
asRNA:CR4	24.45125	-4.6558	3.831429	-1.21516	0.224305	NA
PRY	0	NA	NA	NA	NA	NA
Mst77Y-4	0	NA	NA	NA	NA	NA
Mst77Y-13	0	NA	NA	NA	NA	NA
Mst77Y-7	0	NA	NA	NA	NA	NA
Mst77Y-9	0	NA	NA	NA	NA	NA
Mst77Y-8F	0	NA	NA	NA	NA	NA
asRNA:CR4	2.992556	-1.1917	3.874831	-0.30755	0.758426	NA
2SrRNA:CF	0	NA	NA	NA	NA	NA
28SrRNA:C	0	NA	NA	NA	NA	NA
18SrRNA:C	0	NA	NA	NA	NA	NA
5.8SrRNA:(	0	NA	NA	NA	NA	NA
2SrRNA:CF	0	NA	NA	NA	NA	NA
18SrRNA:C	0	NA	NA	NA	NA	NA
5.8SrRNA:(	0	NA	NA	NA	NA	NA
2SrRNA:CF	0	NA	NA	NA	NA	NA
28SrRNA:C	0	NA	NA	NA	NA	NA
pre-rRNA:(	1.105046	0.352707	3.9248	0.089866	0.928394	NA
5.8SrRNA-I	0	NA	NA	NA	NA	NA
2SrRNA-Ps	0	NA	NA	NA	NA	NA
28SrRNA-F	10.77055	-0.35551	3.74939	-0.09482	0.924459	NA
5.8SrRNA:(	0	NA	NA	NA	NA	NA
28SrRNA-F	33.79481	-0.61642	3.732525	-0.16515	0.868827	NA
5.8SrRNA-I	2.765401	1.298228	3.824442	0.339456	0.734267	NA
5.8SrRNA-I	1.882835	1.186085	3.870456	0.306446	0.759265	NA
2SrRNA-Ps	0.822293	-0.3043	3.943495	-0.07717	0.938492	NA
28SrRNA-F	30.24361	-1.48273	3.741689	-0.39627	0.691904	NA
18SrRNA-F	0	NA	NA	NA	NA	NA
28SrRNA-F	0	NA	NA	NA	NA	NA
5.8SrRNA-I	6.317042	-0.01047	3.760156	-0.00278	0.997779	NA
2SrRNA:CF	2.595543	0.932825	3.870466	0.241011	0.809547	NA
asRNA:CR4	0	NA	NA	NA	NA	NA
lncRNA:CR	0	NA	NA	NA	NA	NA
asRNA:CR4	0	NA	NA	NA	NA	NA
asRNA:CR4	28.38777	-3.16628	3.910246	-0.80974	0.41809	NA
asRNA:CR4	0	NA	NA	NA	NA	NA
asRNA:CR4	0	NA	NA	NA	NA	NA
asRNA:CR4	0	NA	NA	NA	NA	NA
asRNA:CR4	0	NA	NA	NA	NA	NA
asRNA:CR4	0.466186	0.959471	4.033986	0.237847	0.812	NA
asRNA:CR4	14.77113	-0.51583	3.827631	-0.13477	0.892797	NA
asRNA:CR4	0	NA	NA	NA	NA	NA
lncRNA:CR	0.209247	1.042804	4.047837	0.25762	0.7967	NA
lncRNA:CR	0.546909	-1.52446	3.990381	-0.38203	0.702437	NA
lncRNA:CR	5.392151	-0.24879	3.939994	-0.06315	0.949651	NA
CR45879	0	NA	NA	NA	NA	NA

lncRNA:CR	0	NA	NA	NA	NA	NA
lncRNA:CR	14.39647	-1.91368	3.953602	-0.48403	0.628361	NA
asRNA:CR4	0	NA	NA	NA	NA	NA
lncRNA:CR	0	NA	NA	NA	NA	NA
lncRNA:CR	0	NA	NA	NA	NA	NA
asRNA:CR4	0	NA	NA	NA	NA	NA
asRNA:CR4	0	NA	NA	NA	NA	NA
lncRNA:CR	0	NA	NA	NA	NA	NA
lncRNA:CR	0	NA	NA	NA	NA	NA
asRNA:CR4	0.712213	-1.57119	3.998613	-0.39293	0.694369	NA
asRNA:CR4	0.104623	0.639075	4.067674	0.157111	0.875158	NA
asRNA:CR4	0	NA	NA	NA	NA	NA
asRNA:CR4	0	NA	NA	NA	NA	NA
lncRNA:CR	0	NA	NA	NA	NA	NA
asRNA:CR4	0	NA	NA	NA	NA	NA
asRNA:CR4	0.236701	-0.24276	4.020028	-0.06039	0.951846	NA
asRNA:CR4	0	NA	NA	NA	NA	NA
lncRNA:CR	0	NA	NA	NA	NA	NA
lncRNA:CR	0	NA	NA	NA	NA	NA
lncRNA:CR	0	NA	NA	NA	NA	NA
lncRNA:CR	0	NA	NA	NA	NA	NA
lncRNA:CR	0	NA	NA	NA	NA	NA
lncRNA:CR	4.502097	1.711245	3.961986	0.431916	0.665803	NA
lncRNA:CR	0	NA	NA	NA	NA	NA
lncRNA:CR	0	NA	NA	NA	NA	NA
lncRNA:CR	0	NA	NA	NA	NA	NA
lncRNA:CR	7.896637	-0.10602	3.843338	-0.02759	0.977993	NA
asRNA:CR4	23.49128	-7.65283	3.910018	-1.95724	0.05032	NA
asRNA:CR4	19.44283	-0.35691	3.940359	-0.09058	0.927827	NA
asRNA:CR4	0	NA	NA	NA	NA	NA
asRNA:CR4	7.405948	-4.33579	3.832055	-1.13145	0.257865	NA
lncRNA:CR	0.178053	-1.16467	4.075067	-0.2858	0.775028	NA
lncRNA:CR	1.251686	-2.06022	4.011654	-0.51356	0.60756	NA
lncRNA:CR	0	NA	NA	NA	NA	NA
lncRNA:CR	0	NA	NA	NA	NA	NA
lncRNA:CR	0	NA	NA	NA	NA	NA
lncRNA:CR	0.1047	0.613261	4.062799	0.150945	0.880019	NA
lncRNA:CR	0	NA	NA	NA	NA	NA
lncRNA:CR	0	NA	NA	NA	NA	NA
lncRNA:dn	23.13868	1.745763	3.953144	0.441614	0.658769	NA
asRNA:CR4	2.377398	-1.86964	3.966533	-0.47135	0.637388	NA
asRNA:CR4	0	NA	NA	NA	NA	NA
asRNA:CR4	0	NA	NA	NA	NA	NA
asRNA:CR4	18.51832	1.532353	3.952846	0.387658	0.698269	NA
asRNA:CR4	0	NA	NA	NA	NA	NA
CCY	0	NA	NA	NA	NA	NA
CR45931	0	NA	NA	NA	NA	NA
CR45932	0	NA	NA	NA	NA	NA
CR45933	0	NA	NA	NA	NA	NA
CR45934	0.104623	0.639075	4.067674	0.157111	0.875158	NA

lncRNA:CR	0.236701	-0.24276	4.020028	-0.06039	0.951846	NA
lncRNA:CR	19.57889	1.744162	3.953546	0.441164	0.659094	NA
lncRNA:CR	0	NA	NA	NA	NA	NA
lncRNA:CR	34.21651	5.408294	3.867203	1.398503	0.161962	NA
lncRNA:CR	0	NA	NA	NA	NA	NA
lncRNA:CR	0.536437	-1.71175	4.038042	-0.42391	0.671635	NA
lncRNA:CR	60.09046	0.108146	3.877936	0.027888	0.977752	NA
lncRNA:CR	22.58528	-1.91707	3.95263	-0.48501	0.627668	NA
asRNA:CR4	0	NA	NA	NA	NA	NA
asRNA:CR4	2.089576	-1.64443	3.854892	-0.42658	0.669683	NA
lncRNA:CR	6.242746	-5.91587	3.917721	-1.51003	0.131037	NA
CR45946	0	NA	NA	NA	NA	NA
CR45947	0	NA	NA	NA	NA	NA
CR45948	0	NA	NA	NA	NA	NA
lncRNA:CR	1.150933	2.556099	3.971366	0.643632	0.519814	NA
lncRNA:CR	0	NA	NA	NA	NA	NA
asRNA:CR4	3.689025	2.035225	3.827627	0.53172	0.59492	NA
asRNA:CR4	0	NA	NA	NA	NA	NA
lncRNA:CR	14.55648	2.381884	3.924069	0.606993	0.543855	NA
lncRNA:CR	0	NA	NA	NA	NA	NA
asRNA:CR4	0	NA	NA	NA	NA	NA
lncRNA:CR	0	NA	NA	NA	NA	NA
lncRNA:CR	0	NA	NA	NA	NA	NA
asRNA:CR4	0.1047	0.613261	4.062799	0.150945	0.880019	NA
lncRNA:CR	0.1047	0.613261	4.062799	0.150945	0.880019	NA
lncRNA:CR	0	NA	NA	NA	NA	NA
lncRNA:CR	22.26746	4.480072	3.881716	1.154147	0.24844	NA
lncRNA:CR	0.1047	0.613261	4.062799	0.150945	0.880019	NA
lncRNA:CR	0	NA	NA	NA	NA	NA
lncRNA:CR	0	NA	NA	NA	NA	NA
asRNA:CR4	0	NA	NA	NA	NA	NA
asRNA:CR4	4.273279	-1.68149	3.959707	-0.42465	0.671092	NA
asRNA:CR4	0	NA	NA	NA	NA	NA
lncRNA:CR	3.406565	-1.59698	3.959971	-0.40328	0.686741	NA
lncRNA:CR	8.375918	2.928025	3.929792	0.745084	0.456221	NA
asRNA:CR4	0	NA	NA	NA	NA	NA
lncRNA:CR	0	NA	NA	NA	NA	NA
lncRNA:CR	0	NA	NA	NA	NA	NA
lncRNA:CR	0	NA	NA	NA	NA	NA
lncRNA:CR	0.233093	0.692656	4.076246	0.169925	0.865069	NA
lncRNA:CR	0	NA	NA	NA	NA	NA
lncRNA:CR	0	NA	NA	NA	NA	NA
lncRNA:CR	61.3842	-0.37547	3.848515	-0.09756	0.92228	NA
lncRNA:CR	0	NA	NA	NA	NA	NA
lncRNA:CR	0	NA	NA	NA	NA	NA
lncRNA:CR	0	NA	NA	NA	NA	NA
pre-mod(n	0	NA	NA	NA	NA	NA
pre-mod(n	0	NA	NA	NA	NA	NA
pre-mod(n	0	NA	NA	NA	NA	NA
pre-mod(n	0	NA	NA	NA	NA	NA

pre-mod(n	0	NA	NA	NA	NA	NA
lncRNA:CR	0	NA	NA	NA	NA	NA
asRNA:CR4	0	NA	NA	NA	NA	NA
lncRNA:CR	18.32249	1.743452	3.953725	0.440964	0.659239	NA
lncRNA:CR	0.178053	-1.16467	4.075067	-0.2858	0.775028	NA
lncRNA:CR	0	NA	NA	NA	NA	NA
asRNA:CR4	0	NA	NA	NA	NA	NA
lncRNA:CR	0	NA	NA	NA	NA	NA
lncRNA:CR	0	NA	NA	NA	NA	NA
lncRNA:CR	0	NA	NA	NA	NA	NA
lncRNA:CR	15.79724	-2.14689	3.83328	-0.56007	0.575435	NA
lncRNA:CR	24.17597	1.976401	3.849234	0.513453	0.607634	NA
asRNA:CR4	0.31089	-1.9003	4.033427	-0.47114	0.637542	NA
lncRNA:CR	7.300184	-1.69234	3.956109	-0.42778	0.668812	NA
lncRNA:CR	5.027078	-3.91478	3.923105	-0.99788	0.318338	NA
lncRNA:CR	0	NA	NA	NA	NA	NA
lncRNA:CR	10.15877	1.720737	3.854791	0.446389	0.655316	NA
lncRNA:CR	0	NA	NA	NA	NA	NA
asRNA:CR4	0	NA	NA	NA	NA	NA
lncRNA:CR	4.397397	1.710278	3.962238	0.431644	0.666	NA
lncRNA:CR	0	NA	NA	NA	NA	NA
lncRNA:CR	1.788123	-2.11324	3.996051	-0.52883	0.596922	NA
lncRNA:CR	0	NA	NA	NA	NA	NA
lncRNA:CR	0	NA	NA	NA	NA	NA
asRNA:CR4	0	NA	NA	NA	NA	NA
lncRNA:CR	0	NA	NA	NA	NA	NA
asRNA:CR4	0	NA	NA	NA	NA	NA
lncRNA:CR	25.17312	6.917906	3.91192	1.768417	0.076991	NA
lncRNA:CR	38.69348	1.176553	3.952124	0.297701	0.765931	NA
lncRNA:CR	25.75618	1.74666	3.952918	0.441866	0.658586	NA
lncRNA:CR	0	NA	NA	NA	NA	NA
CG46025	0	NA	NA	NA	NA	NA
CG46026	0	NA	NA	NA	NA	NA
CR46027	0	NA	NA	NA	NA	NA
lncRNA:CR	0	NA	NA	NA	NA	NA
asRNA:CR4	3.587175	0.666222	3.934688	0.16932	0.865545	NA
lncRNA:CR	0	NA	NA	NA	NA	NA
lncRNA:CR	0	NA	NA	NA	NA	NA
lncRNA:CR	0	NA	NA	NA	NA	NA
asRNA:CR4	0	NA	NA	NA	NA	NA
asRNA:CR4	0	NA	NA	NA	NA	NA
lncRNA:CR	7.060928	0.241064	3.929271	0.061351	0.95108	NA
lncRNA:fla	37.98432	-0.00713	3.671125	-0.00194	0.998451	NA
asRNA:CR4	0	NA	NA	NA	NA	NA
lncRNA:CR	0	NA	NA	NA	NA	NA
lncRNA:CR	0	NA	NA	NA	NA	NA
lncRNA:CR	0	NA	NA	NA	NA	NA
CG46042	0	NA	NA	NA	NA	NA
lncRNA:CR	0	NA	NA	NA	NA	NA
asRNA:CR4	6.192502	-0.21435	3.95184	-0.05424	0.956744	NA

asRNA:CR4	0	NA	NA	NA	NA	NA
lncRNA:CR	0	NA	NA	NA	NA	NA
asRNA:CR4	0	NA	NA	NA	NA	NA
asRNA:CR4	36.44735	-2.05857	3.837895	-0.53638	0.591696	NA
asRNA:CR4	0	NA	NA	NA	NA	NA
asRNA:CR4	3.557191	1.503975	3.960718	0.379723	0.704151	NA
lncRNA:CR	0	NA	NA	NA	NA	NA
lncRNA:CR	0	NA	NA	NA	NA	NA
asRNA:CR4	0	NA	NA	NA	NA	NA
asRNA:CR4	0	NA	NA	NA	NA	NA
lncRNA:CR	2.680666	-3.72746	3.969815	-0.93895	0.347756	NA
lncRNA:CR	0	NA	NA	NA	NA	NA
asRNA:CR4	0	NA	NA	NA	NA	NA
CG46059	0	NA	NA	NA	NA	NA
lncRNA:CR	0	NA	NA	NA	NA	NA
lncRNA:CR	0	NA	NA	NA	NA	NA
lncRNA:CR	0	NA	NA	NA	NA	NA
asRNA:CR4	0	NA	NA	NA	NA	NA
asRNA:CR4	10.02672	-0.48795	3.879095	-0.12579	0.899899	NA
asRNA:CR4	0	NA	NA	NA	NA	NA
asRNA:CR4	5.519652	-1.68736	3.957756	-0.42634	0.669859	NA
asRNA:CR4	0	NA	NA	NA	NA	NA
asRNA:CR4	0	NA	NA	NA	NA	NA
asRNA:CR4	0	NA	NA	NA	NA	NA
CR46072	0	NA	NA	NA	NA	NA
lncRNA:CR	29.83767	0.042745	3.951496	0.010817	0.991369	NA
asRNA:CR4	0	NA	NA	NA	NA	NA
lncRNA:CR	0.178812	-1.08808	4.061735	-0.26789	0.788788	NA
lncRNA:CR	0	NA	NA	NA	NA	NA
lncRNA:CR	0	NA	NA	NA	NA	NA
lncRNA:CR	0.4188	1.41068	4.034613	0.349644	0.726606	NA
lncRNA:CR	0	NA	NA	NA	NA	NA
asRNA:CR4	0	NA	NA	NA	NA	NA
asRNA:CR4	30.44537	1.53513	3.95209	0.388435	0.697694	NA
lncRNA:CR	0	NA	NA	NA	NA	NA
lncRNA:CR	0	NA	NA	NA	NA	NA
lncRNA:CR	0	NA	NA	NA	NA	NA
lncRNA:CR	0	NA	NA	NA	NA	NA
lncRNA:CR	0	NA	NA	NA	NA	NA
asRNA:CR4	0	NA	NA	NA	NA	NA
lncRNA:CR	1.320777	-1.83209	3.977996	-0.46056	0.645117	NA
asRNA:CR4	0	NA	NA	NA	NA	NA
asRNA:CR4	0	NA	NA	NA	NA	NA
asRNA:CR4	37.16358	7.864241	3.908785	2.01194	0.044226	NA
lncRNA:CR	0.5235	1.490714	4.02555	0.370313	0.711149	NA
asRNA:CR4	0	NA	NA	NA	NA	NA
asRNA:CR4	0	NA	NA	NA	NA	NA
lncRNA:CR	2.082181	-0.96846	3.93372	-0.2462	0.805531	NA
asRNA:CR4	0	NA	NA	NA	NA	NA
lncRNA:CR	0	NA	NA	NA	NA	NA

asRNA:CR4	0	NA	NA	NA	NA	NA
lncRNA:CR	0	NA	NA	NA	NA	NA
asRNA:CR4	0.3141	1.240997	4.03841	0.307299	0.758616	NA
asRNA:CR4	0.712213	-1.57119	3.998613	-0.39293	0.694369	NA
lncRNA:CR	0	NA	NA	NA	NA	NA
asRNA:CR4	1.849088	-1.85577	3.970721	-0.46736	0.640241	NA
lncRNA:CR	1.675199	1.648777	3.978727	0.414398	0.678583	NA
asRNA:CR4	8.452972	-1.9072	3.955468	-0.48217	0.629686	NA
lncRNA:CR	0.837599	1.568055	4.001801	0.391837	0.695178	NA
lncRNA:CR	0.178053	-1.16467	4.075067	-0.2858	0.775028	NA
lncRNA:CR	0	NA	NA	NA	NA	NA
lncRNA:CR	0	NA	NA	NA	NA	NA
lncRNA:CR	0	NA	NA	NA	NA	NA
lncRNA:CR	35.78123	2.772161	3.917333	0.707665	0.479153	NA
lncRNA:CR	50.36067	1.75058	3.951939	0.442967	0.657789	NA
lncRNA:CR	0	NA	NA	NA	NA	NA
lncRNA:CR	0	NA	NA	NA	NA	NA
lncRNA:CR	0	NA	NA	NA	NA	NA
rgr	24.45421	-0.95665	3.870901	-0.24714	0.804801	NA
lncRNA:CR	0.712972	-2.55723	4.00634	-0.63829	0.523282	NA
lncRNA:CR	0	NA	NA	NA	NA	NA
lncRNA:CR	0	NA	NA	NA	NA	NA
lncRNA:CR	1.111482	-3.16801	3.986608	-0.79466	0.426809	NA
lncRNA:CR	10.7864	-0.32907	3.842081	-0.08565	0.931746	NA
lncRNA:CR	17.77561	-6.78916	3.913679	-1.73473	0.082789	NA
lncRNA:CR	0.233093	0.692656	4.076246	0.169925	0.865069	NA
asRNA:CR4	11.18848	1.167813	3.955086	0.295269	0.767789	NA
asRNA:CR4	0	NA	NA	NA	NA	NA
lncRNA:CR	0	NA	NA	NA	NA	NA
lncRNA:CR	27.32668	1.747117	3.952803	0.441994	0.658493	NA
lncRNA:CR	0.178812	-1.08808	4.061735	-0.26789	0.788788	NA
asRNA:CR4	0	NA	NA	NA	NA	NA
asRNA:CR4	0	NA	NA	NA	NA	NA
asRNA:CR4	0.388212	0.094116	4.038892	0.023303	0.981409	NA
asRNA:CR4	0.178812	-1.08808	4.061735	-0.26789	0.788788	NA
asRNA:CR4	51.86084	1.254897	3.729034	0.336521	0.736478	NA
asRNA:CR4	0	NA	NA	NA	NA	NA
asRNA:CR4	31.48333	1.686518	3.952477	0.426699	0.669598	NA
asRNA:CR4	0	NA	NA	NA	NA	NA
lncRNA:CR	8.606562	1.391373	3.954671	0.35183	0.724965	NA
lncRNA:CR	0.388059	-0.03373	4.021335	-0.00839	0.993308	NA
lncRNA:CR	0	NA	NA	NA	NA	NA
asRNA:CR4	9.614877	-1.69611	3.95487	-0.42887	0.66802	NA
lncRNA:CR	0	NA	NA	NA	NA	NA
lncRNA:CR	0	NA	NA	NA	NA	NA
asRNA:CR4	0	NA	NA	NA	NA	NA
CR46150	0	NA	NA	NA	NA	NA
CR46151	0	NA	NA	NA	NA	NA
CR46152	0	NA	NA	NA	NA	NA
lncRNA:CR	0	NA	NA	NA	NA	NA

CR46154	0	NA	NA	NA	NA	NA
CR46155	0	NA	NA	NA	NA	NA
lncRNA:CR	0	NA	NA	NA	NA	NA
CR46157	0	NA	NA	NA	NA	NA
CR46158	0	NA	NA	NA	NA	NA
CR46159	0	NA	NA	NA	NA	NA
CR46160	0	NA	NA	NA	NA	NA
CR46161	0	NA	NA	NA	NA	NA
CR46162	0	NA	NA	NA	NA	NA
CR46163	0	NA	NA	NA	NA	NA
CR46164	0	NA	NA	NA	NA	NA
CR46165	0	NA	NA	NA	NA	NA
CR46166	0	NA	NA	NA	NA	NA
CR46167	0	NA	NA	NA	NA	NA
CR46168	0	NA	NA	NA	NA	NA
CR46169	0	NA	NA	NA	NA	NA
CR46170	0	NA	NA	NA	NA	NA
CR46171	0	NA	NA	NA	NA	NA
CR46172	0	NA	NA	NA	NA	NA
CR46173	0	NA	NA	NA	NA	NA
CR46174	0	NA	NA	NA	NA	NA
CR46175	0	NA	NA	NA	NA	NA
CR46176	0	NA	NA	NA	NA	NA
CR46177	0	NA	NA	NA	NA	NA
CR46178	0	NA	NA	NA	NA	NA
CR46179	0	NA	NA	NA	NA	NA
CR46180	0	NA	NA	NA	NA	NA
CR46181	0	NA	NA	NA	NA	NA
CR46182	0	NA	NA	NA	NA	NA
CR46183	0	NA	NA	NA	NA	NA
CR46184	0	NA	NA	NA	NA	NA
CR46185	0	NA	NA	NA	NA	NA
CR46186	0	NA	NA	NA	NA	NA
CR46187	0	NA	NA	NA	NA	NA
CR46188	0	NA	NA	NA	NA	NA
CR46189	0	NA	NA	NA	NA	NA
CR46190	0	NA	NA	NA	NA	NA
CG46191	0	NA	NA	NA	NA	NA
CG46192	0	NA	NA	NA	NA	NA
CG46193	0	NA	NA	NA	NA	NA
asRNA:CR4	0	NA	NA	NA	NA	NA
lncRNA:CR	0	NA	NA	NA	NA	NA
lncRNA:CR	0	NA	NA	NA	NA	NA
lncRNA:CR	0	NA	NA	NA	NA	NA
lncRNA:CR	0	NA	NA	NA	NA	NA
lncRNA:CR	1.778595	1.471835	3.969892	0.370749	0.710824	NA
lncRNA:CR	0	NA	NA	NA	NA	NA
lncRNA:CR	0	NA	NA	NA	NA	NA
lncRNA:CR	8.030548	1.525016	3.95632	0.385463	0.699894	NA
lncRNA:CR	0	NA	NA	NA	NA	NA

lncRNA:CR	0	NA	NA	NA	NA	NA
lncRNA:CR	0	NA	NA	NA	NA	NA
lncRNA:CR	0	NA	NA	NA	NA	NA
lncRNA:CR	0	NA	NA	NA	NA	NA
lncRNA:CR	0	NA	NA	NA	NA	NA
lncRNA:CR	0	NA	NA	NA	NA	NA
lncRNA:CR	0	NA	NA	NA	NA	NA
lncRNA:CR	0	NA	NA	NA	NA	NA
lncRNA:CR	0	NA	NA	NA	NA	NA
lncRNA:CR	0.356107	-1.47099	4.037764	-0.36431	0.715628	NA
lncRNA:CR	0	NA	NA	NA	NA	NA
lncRNA:CR	0	NA	NA	NA	NA	NA
lncRNA:CR	0	NA	NA	NA	NA	NA
lncRNA:CR	0.233093	0.692656	4.076246	0.169925	0.865069	NA
lncRNA:CR	0	NA	NA	NA	NA	NA
lncRNA:CR	0	NA	NA	NA	NA	NA
lncRNA:CR	0	NA	NA	NA	NA	NA
lncRNA:CR	0	NA	NA	NA	NA	NA
lncRNA:CR	13.78281	-3.1405	3.92095	-0.80095	0.423159	NA
lncRNA:CR	0.233093	0.692656	4.076246	0.169925	0.865069	NA
lncRNA:CR	0	NA	NA	NA	NA	NA
lncRNA:CR	0	NA	NA	NA	NA	NA
lncRNA:CR	0	NA	NA	NA	NA	NA
CR46232	0	NA	NA	NA	NA	NA
lncRNA:CR	0	NA	NA	NA	NA	NA
lncRNA:CR	0	NA	NA	NA	NA	NA
lncRNA:CR	4.187997	1.708209	3.962778	0.431063	0.666422	NA
lncRNA:CR	0	NA	NA	NA	NA	NA
lncRNA:CR	0	NA	NA	NA	NA	NA
lncRNA:CR	0	NA	NA	NA	NA	NA
lncRNA:CR	0	NA	NA	NA	NA	NA
lncRNA:CR	0	NA	NA	NA	NA	NA
lncRNA:CR	0	NA	NA	NA	NA	NA
lncRNA:CR	0	NA	NA	NA	NA	NA
lncRNA:CR	0	NA	NA	NA	NA	NA
CG46244	0	NA	NA	NA	NA	NA
lncRNA:CR	0	NA	NA	NA	NA	NA
asRNA:CR4	21.34314	1.533287	3.952591	0.387919	0.698076	NA
corolla	0	NA	NA	NA	NA	NA
lncRNA:CR	0.365171	-0.43584	4.056359	-0.10745	0.914434	NA
lncRNA:CR	0	NA	NA	NA	NA	NA
mh	32.79769	0.40497	3.835261	0.105591	0.915907	NA
lncRNA:CR	0	NA	NA	NA	NA	NA
lncRNA:CR	1.224286	0.5443	3.941384	0.138099	0.890162	NA
asRNA:CR4	0	NA	NA	NA	NA	NA
lncRNA:CR	0	NA	NA	NA	NA	NA
lncRNA:CR	49.26498	-1.92033	3.951699	-0.48595	0.627001	NA
asRNA:CR4	0	NA	NA	NA	NA	NA
asRNA:CR4	0	NA	NA	NA	NA	NA
lncRNA:CR	0.1047	0.613261	4.062799	0.150945	0.880019	NA

lncRNA:CR	60.80649	-5.92873	3.798939	-1.56063	0.118612	NA
lncRNA:CR	0	NA	NA	NA	NA	NA
lncRNA:CR	3.399867	0.625277	3.948538	0.158357	0.874176	NA
asRNA:CR4	0.6282	1.523021	4.015426	0.379293	0.704471	NA
asRNA:CR4	7.175252	2.865514	3.90792	0.733258	0.463401	NA
ltl	33.89211	2.282995	3.888173	0.587164	0.557094	NA
CG4836	0.76953	-0.78481	3.994657	-0.19646	0.844247	NA
asRNA:CR4	0	NA	NA	NA	NA	NA
lncRNA:CR	4.195678	1.148144	3.961859	0.289799	0.77197	NA
asRNA:CR4	0	NA	NA	NA	NA	NA
lncRNA:CR	0	NA	NA	NA	NA	NA
asRNA:CR4	0	NA	NA	NA	NA	NA
CG46270	0	NA	NA	NA	NA	NA
lncRNA:CR	21.00638	-4.09826	3.910875	-1.04791	0.294679	NA
CR46272	0	NA	NA	NA	NA	NA
CR46273	0	NA	NA	NA	NA	NA
CR46274	0	NA	NA	NA	NA	NA
SdicA	0	NA	NA	NA	NA	NA
SdicB	0	NA	NA	NA	NA	NA
SdicC	0	NA	NA	NA	NA	NA
CR46279	0.132078	-1.13211	4.069918	-0.27817	0.780885	NA
PPO1	0	NA	NA	NA	NA	NA
solo	0	NA	NA	NA	NA	NA
vas	2.826898	1.687978	3.968112	0.425386	0.670556	NA
Drs	0	NA	NA	NA	NA	NA
IMPPP	0	NA	NA	NA	NA	NA
Dhc16F	1.775574	-1.56317	3.850786	-0.40593	0.684791	NA
Alp1	0	NA	NA	NA	NA	NA
Ak2	36.24403	-0.32411	3.715943	-0.08722	0.930495	NA
lncRNA:CR	0.178812	-1.08808	4.061735	-0.26789	0.788788	NA
nw	0	NA	NA	NA	NA	NA
Duox	2.702574	-1.65369	3.877152	-0.42652	0.669727	NA
vilya	46.89963	-7.63202	3.912315	-1.95077	0.051085	NA
CG46298	0	NA	NA	NA	NA	NA
lncRNA:CR	0	NA	NA	NA	NA	NA
CR46300	0	NA	NA	NA	NA	NA
Osi21	0	NA	NA	NA	NA	NA
CG31600	0	NA	NA	NA	NA	NA
CG46302	2.241108	-0.36611	3.822753	-0.09577	0.923702	NA
CG46303	5.777958	-5.10368	3.905934	-1.30665	0.191332	NA
CG46304	0	NA	NA	NA	NA	NA
CG46305	0	NA	NA	NA	NA	NA
CG46306	0	NA	NA	NA	NA	NA
CG46307	0	NA	NA	NA	NA	NA
CG46308	0	NA	NA	NA	NA	NA
CG46309	0	NA	NA	NA	NA	NA
CG46310	0	NA	NA	NA	NA	NA
CG46311	0	NA	NA	NA	NA	NA
CG46312	0	NA	NA	NA	NA	NA
CG46313	0	NA	NA	NA	NA	NA

CG46314	0	NA	NA	NA	NA	NA
CG46315	0	NA	NA	NA	NA	NA
CG46316	0	NA	NA	NA	NA	NA
CG46317	0	NA	NA	NA	NA	NA
CG46318	0	NA	NA	NA	NA	NA
CG46319	0	NA	NA	NA	NA	NA
CG46320	0	NA	NA	NA	NA	NA
CG46321	0	NA	NA	NA	NA	NA
lncRNA:CR	0.1047	0.613261	4.062799	0.150945	0.880019	NA
l(2)k05911	0	NA	NA	NA	NA	NA
ds	2.796185	-0.43502	3.958512	-0.10989	0.912493	NA
Oaz	55.08358	0.295828	3.950253	0.074888	0.940303	NA
lncRNA:TS:	0.104623	0.639075	4.067674	0.157111	0.875158	NA
lncRNA:TS:	0	NA	NA	NA	NA	NA
lncRNA:TS:	0	NA	NA	NA	NA	NA
lncRNA:CR	0	NA	NA	NA	NA	NA
lncRNA:TS4	0.411147	-0.29058	4.046973	-0.0718	0.94276	NA
lncRNA:CR	0	NA	NA	NA	NA	NA
lncRNA:TS(	0	NA	NA	NA	NA	NA
lncRNA:TS!	0.104623	0.639075	4.067674	0.157111	0.875158	NA
lncRNA:TS:	0	NA	NA	NA	NA	NA
lncRNA:TS:	0	NA	NA	NA	NA	NA
lncRNA:TS:	0	NA	NA	NA	NA	NA
lncRNA:TS:	0.283436	-0.20033	4.070792	-0.04921	0.960751	NA
btl	0	NA	NA	NA	NA	NA
mah	0	NA	NA	NA	NA	NA
CG46335	0.4188	1.41068	4.034613	0.349644	0.726606	NA
lncRNA:CR	0	NA	NA	NA	NA	NA
knon	0	NA	NA	NA	NA	NA
Fuca	0	NA	NA	NA	NA	NA
sisRNA:1	0	NA	NA	NA	NA	NA
hpRNA:1	0	NA	NA	NA	NA	NA
sisRNA:4	0.132078	-1.13211	4.069918	-0.27817	0.780885	NA
CG32500	2.823422	-0.23191	3.951154	-0.05869	0.953196	NA
prg	24.01921	5.432886	3.853673	1.409794	0.1586	NA
hpRNA:CR4	0	NA	NA	NA	NA	NA
hpRNA:CR4	0	NA	NA	NA	NA	NA
lncRNA:CR	24.21525	-1.70339	3.952491	-0.43097	0.666493	NA
lncRNA:CR	0.894061	-2.00006	4.030294	-0.49626	0.619714	NA
lncRNA:CR	26.05119	1.534399	3.952288	0.388231	0.697845	NA
sisRNA:CR4	0.3141	1.240997	4.03841	0.307299	0.758616	NA
sisRNA:CR4	0	NA	NA	NA	NA	NA
sisRNA:3	41.16015	1.065863	3.91908	0.271968	0.785647	NA
sisRNA:CR4	0	NA	NA	NA	NA	NA
sisRNA:CR4	0	NA	NA	NA	NA	NA
sisRNA:2	0	NA	NA	NA	NA	NA
sisRNA:CR4	0	NA	NA	NA	NA	NA
sisRNA:CR4	0	NA	NA	NA	NA	NA
AdamTS-A	0.932373	1.053996	3.996193	0.26375	0.791972	NA
stw	30.98428	1.72462	3.898599	0.442369	0.658222	NA

ich	46.84806	-3.6295	3.914224	-0.92726	0.353792	NA
vrs	0	NA	NA	NA	NA	NA
Had1	0	NA	NA	NA	NA	NA
hll	0.132078	-1.13211	4.069918	-0.27817	0.780885	NA
snoRNA:O	0.5235	1.490714	4.02555	0.370313	0.711149	NA
snoRNA:O	3.455098	1.699144	3.965156	0.428519	0.668273	NA
snoRNA:O	0	NA	NA	NA	NA	NA
snoRNA:O	0	NA	NA	NA	NA	NA
snoRNA:O	0	NA	NA	NA	NA	NA
snoRNA:O	30.4167	4.477644	3.850131	1.162985	0.244836	NA
snoRNA:M	0	NA	NA	NA	NA	NA
snoRNA:M	0.536437	-1.71175	4.038042	-0.42391	0.671635	NA
scaRNA:M	0	NA	NA	NA	NA	NA
snoRNA:O	31.51468	1.748118	3.952553	0.442276	0.65829	NA
snoRNA:O	0	NA	NA	NA	NA	NA
scaRNA:M	0	NA	NA	NA	NA	NA
snoRNA:M	0	NA	NA	NA	NA	NA
snoRNA:O	0	NA	NA	NA	NA	NA
CG46383	0	NA	NA	NA	NA	NA
snoRNA:M	0	NA	NA	NA	NA	NA
Orc1	0.31387	1.261782	4.037145	0.312543	0.754628	NA
CG46388	0	NA	NA	NA	NA	NA
lncRNA:osl	0	NA	NA	NA	NA	NA
lncRNA:SM	0	NA	NA	NA	NA	NA
lncRNA:CR	0	NA	NA	NA	NA	NA
lncRNA:en	0	NA	NA	NA	NA	NA
lncRNA:CR	40.77362	6.844073	3.846532	1.779284	0.075193	NA
CG46394	0.104623	0.639075	4.067674	0.157111	0.875158	NA
CG46395	0	NA	NA	NA	NA	NA
CG46396	0	NA	NA	NA	NA	NA
CG46398	0	NA	NA	NA	NA	NA
CG46399	0	NA	NA	NA	NA	NA
sbRNA:1	0	NA	NA	NA	NA	NA
CR46401	0.356107	-1.47099	4.037764	-0.36431	0.715628	NA
CR46402	0.924544	-1.7993	3.988349	-0.45114	0.651889	NA
CR46403	0	NA	NA	NA	NA	NA
CR46404	0.104623	0.639075	4.067674	0.157111	0.875158	NA
CR46406	0	NA	NA	NA	NA	NA
CR46407	0	NA	NA	NA	NA	NA
lncRNA:CR	0	NA	NA	NA	NA	NA
lncRNA:CR	0	NA	NA	NA	NA	NA
lncRNA:CR	0	NA	NA	NA	NA	NA
lncRNA:CR	0	NA	NA	NA	NA	NA
CG46412	0	NA	NA	NA	NA	NA
CR46413	0	NA	NA	NA	NA	NA
lncRNA:CR	0	NA	NA	NA	NA	NA
CR46415	0	NA	NA	NA	NA	NA
lncRNA:CR	0	NA	NA	NA	NA	NA
CR46417	0	NA	NA	NA	NA	NA
CR46418	0	NA	NA	NA	NA	NA

CR46419	0	NA	NA	NA	NA	NA
CR46420	0	NA	NA	NA	NA	NA
CR46421	0	NA	NA	NA	NA	NA
CR46422	0	NA	NA	NA	NA	NA
CR46423	0	NA	NA	NA	NA	NA
CR46424	0	NA	NA	NA	NA	NA
CG46426	0	NA	NA	NA	NA	NA
CG46427	0	NA	NA	NA	NA	NA
CG46428	0	NA	NA	NA	NA	NA
CG46429	0	NA	NA	NA	NA	NA
CG46430	0.178053	-1.16467	4.075067	-0.2858	0.775028	NA
lncRNA:CR	0	NA	NA	NA	NA	NA
CR46432	0	NA	NA	NA	NA	NA
CG46433	0	NA	NA	NA	NA	NA
CG46434	0	NA	NA	NA	NA	NA
CG46435	0	NA	NA	NA	NA	NA
Osi10a	28.00047	-1.91823	3.952298	-0.48535	0.627431	NA
Osi10b	4.754797	-1.89533	3.958922	-0.47875	0.632117	NA
CG46439	0	NA	NA	NA	NA	NA
Iyd	0	NA	NA	NA	NA	NA
FASN3	0.732899	1.548046	4.007786	0.38626	0.699304	NA
nvd	0.872712	-0.58069	3.940867	-0.14735	0.882854	NA
CG46440	0	NA	NA	NA	NA	NA
CG46441	0	NA	NA	NA	NA	NA
CG46442	0	NA	NA	NA	NA	NA
CG46443	0	NA	NA	NA	NA	NA
lncRNA:CR	0	NA	NA	NA	NA	NA
lncRNA:CR	0	NA	NA	NA	NA	NA
lncRNA:CR	0	NA	NA	NA	NA	NA
CR46447	0	NA	NA	NA	NA	NA
slf	3.21584	-3.35157	3.92887	-0.85306	0.393624	NA
Nplp2	0.132078	-1.13211	4.069918	-0.27817	0.780885	NA
lncRNA:CR	0	NA	NA	NA	NA	NA
lncRNA:CR	0.985641	0.934855	3.921487	0.238393	0.811576	NA
asRNA:CR4	0	NA	NA	NA	NA	NA
asRNA:CR4	0	NA	NA	NA	NA	NA
lncRNA:CR	5.347781	-1.55895	3.918344	-0.39786	0.690734	NA
lncRNA:CR	0	NA	NA	NA	NA	NA
CG46456	0	NA	NA	NA	NA	NA
CG46457	0	NA	NA	NA	NA	NA
CG46458	0	NA	NA	NA	NA	NA
CG46459	0	NA	NA	NA	NA	NA
CG46460	0	NA	NA	NA	NA	NA
CR46461	0	NA	NA	NA	NA	NA
CG46463	0	NA	NA	NA	NA	NA
CG46464	0	NA	NA	NA	NA	NA
CG46465	0.178053	-1.16467	4.075067	-0.2858	0.775028	NA
CG46467	0	NA	NA	NA	NA	NA
CG46468	0	NA	NA	NA	NA	NA
asRNA:CR4	0	NA	NA	NA	NA	NA

asRNA:CR4	0	NA	NA	NA	NA	NA
asRNA:CR4	0.46073	-0.17853	4.00793	-0.04454	0.964471	NA
lncRNA:CR	0	NA	NA	NA	NA	NA
asRNA:CR4	1.188699	-1.8233	3.980741	-0.45803	0.646931	NA
lncRNA:CR	0	NA	NA	NA	NA	NA
asRNA:CR4	20.14764	0.653009	3.773981	0.173029	0.862629	NA
asRNA:CR4	6.040806	-0.34722	3.850518	-0.09017	0.928149	NA
lncRNA:CR	0	NA	NA	NA	NA	NA
dbf	0.396233	-1.6814	4.028351	-0.41739	0.676392	NA
CG46491	0.1047	0.613261	4.062799	0.150945	0.880019	NA
CG46492	0.53416	-1.53481	4.012395	-0.38252	0.702078	NA
lncRNA:Vir	0.732899	1.548046	4.007786	0.38626	0.699304	NA
ccb	20.7453	1.173452	3.953172	0.296838	0.76659	NA
lncRNA:CR	0	NA	NA	NA	NA	NA
lncRNA:CR	0.178053	-1.16467	4.075067	-0.2858	0.775028	NA
lncRNA:CR	0	NA	NA	NA	NA	NA
Pen	31.99226	-0.29486	3.849831	-0.07659	0.93895	NA
esg	0	NA	NA	NA	NA	NA
Datp	0	NA	NA	NA	NA	NA
Myo61F	0.178053	-1.16467	4.075067	-0.2858	0.775028	NA
tRNA:Glu-(	0	NA	NA	NA	NA	NA
tRNA:Glu-(	0	NA	NA	NA	NA	NA
tRNA:Glu-(	0	NA	NA	NA	NA	NA
tRNA:Glu-(	0	NA	NA	NA	NA	NA
tRNA:Glu-(	0	NA	NA	NA	NA	NA
tRNA:Glu-(	0	NA	NA	NA	NA	NA
tRNA:Glu-(	0	NA	NA	NA	NA	NA
CG32320	0.104623	0.639075	4.067674	0.157111	0.875158	NA
tRNA:Glu-(	0	NA	NA	NA	NA	NA
tRNA:Glu-(	0	NA	NA	NA	NA	NA
CG9168	0	NA	NA	NA	NA	NA
FucTD	0	NA	NA	NA	NA	NA
robl62A	0	NA	NA	NA	NA	NA
stet	49.14683	2.326131	3.923627	0.592852	0.55328	NA
Naa30B	0	NA	NA	NA	NA	NA
SA-2	0	NA	NA	NA	NA	NA
CG9173	0	NA	NA	NA	NA	NA
CG9184	0	NA	NA	NA	NA	NA
Psf1	0	NA	NA	NA	NA	NA
CG32318	0	NA	NA	NA	NA	NA
Klp61F	0	NA	NA	NA	NA	NA
CG9192	0	NA	NA	NA	NA	NA
Bro	0	NA	NA	NA	NA	NA
CG12011	0	NA	NA	NA	NA	NA
CG12035	0.1047	0.613261	4.062799	0.150945	0.880019	NA
CG12020	0	NA	NA	NA	NA	NA
GV1	0	NA	NA	NA	NA	NA
Cpr62Bb	0	NA	NA	NA	NA	NA
Cpr62Ba	0.492153	0.308117	3.993933	0.077146	0.938507	NA
Dhc62B	2.057989	0.390629	3.876247	0.100775	0.919729	NA

Cht2	0.178053	-1.16467	4.075067	-0.2858	0.775028	NA
PIG-Wb	0	NA	NA	NA	NA	NA
Oseg4	0.209247	1.042804	4.047837	0.25762	0.7967	NA
CG13930	0	NA	NA	NA	NA	NA
RabX5	0	NA	NA	NA	NA	NA
CG13926	7.597237	-0.04835	3.749374	-0.01289	0.989712	NA
CG32313	7.94478	1.493767	3.901153	0.382904	0.701791	NA
CG13924	0.178053	-1.16467	4.075067	-0.2858	0.775028	NA
tRNA:Cys-(	0	NA	NA	NA	NA	NA
Cnb	0	NA	NA	NA	NA	NA
CG11815	0	NA	NA	NA	NA	NA
mu2	1.510528	-3.57574	3.956385	-0.90379	0.366107	NA
CG5687	37.58727	1.749177	3.952288	0.442573	0.658074	NA
dsb	0.1047	0.613261	4.062799	0.150945	0.880019	NA
CNMa	0	NA	NA	NA	NA	NA
Cpr62Bc	0	NA	NA	NA	NA	NA
CG13810	0.178053	-1.16467	4.075067	-0.2858	0.775028	NA
CG8960	0	NA	NA	NA	NA	NA
Oseg2	0	NA	NA	NA	NA	NA
CG32301	0.1047	0.613261	4.062799	0.150945	0.880019	NA
CG32305	0.209247	1.042804	4.047837	0.25762	0.7967	NA
CG13806	0	NA	NA	NA	NA	NA
obst-I	0	NA	NA	NA	NA	NA
CG32302	0	NA	NA	NA	NA	NA
tRNA:Cys-(	0.104623	0.639075	4.067674	0.157111	0.875158	NA
yellow-g2	0	NA	NA	NA	NA	NA
CG13801	0.178053	-1.16467	4.075067	-0.2858	0.775028	NA
CG32298	0	NA	NA	NA	NA	NA
CG32299	0	NA	NA	NA	NA	NA
yellow-g	0.178053	-1.16467	4.075067	-0.2858	0.775028	NA
CG15878	0	NA	NA	NA	NA	NA
CG15820	0	NA	NA	NA	NA	NA
CR15821	0	NA	NA	NA	NA	NA
CG15879	18.41716	1.242528	3.952837	0.314338	0.753264	NA
CG15822	0.2094	1.005034	4.045341	0.248442	0.803792	NA
CG16984	0.932373	1.053996	3.996193	0.26375	0.791972	NA
CG16985	0.178812	-1.08808	4.061735	-0.26789	0.788788	NA
MEP-1	18.65959	-7.94727	3.909133	-2.033	0.042052	NA
CG1143	0.178053	-1.16467	4.075067	-0.2858	0.775028	NA
CG1246	0	NA	NA	NA	NA	NA
Acp62F	0	NA	NA	NA	NA	NA
CG14949	2.720205	1.493763	3.963603	0.37687	0.70627	NA
CG12182	0.547193	2.009611	4.015384	0.500478	0.616739	NA
CG32295	0.732899	1.548046	4.007786	0.38626	0.699304	NA
Cyp4d20	0	NA	NA	NA	NA	NA
CG16762	0	NA	NA	NA	NA	NA
CPT2	32.15775	-8.55648	3.908352	-2.18928	0.028576	NA
CG2113	0	NA	NA	NA	NA	NA
aly	0	NA	NA	NA	NA	NA
CG32487	0	NA	NA	NA	NA	NA

CG32488	0	NA	NA	NA	NA	NA
CG32846	15.82342	6.300527	3.898026	1.616338	0.106021	NA
CG9970	14.75188	1.530561	3.953337	0.387157	0.69864	NA
spz5	0.1047	0.613261	4.062799	0.150945	0.880019	NA
snRNA:U5:	2.376752	1.184528	3.965092	0.298739	0.765139	NA
tRNA:Cys-(	0	NA	NA	NA	NA	NA
tRNA:Cys-(	0	NA	NA	NA	NA	NA
tRNA:Cys-(	0	NA	NA	NA	NA	NA
tRNA:Lys-C	0	NA	NA	NA	NA	NA
tRNA:Cys-(	0	NA	NA	NA	NA	NA
CG32284	0	NA	NA	NA	NA	NA
CG14957	0	NA	NA	NA	NA	NA
CG14958	0	NA	NA	NA	NA	NA
ckd	0.341478	0.247543	4.031301	0.061405	0.951036	NA
CG14960	0.264155	-1.54099	4.048976	-0.38059	0.703509	NA
CG12017	0.1047	0.613261	4.062799	0.150945	0.880019	NA
CG12009	13.05737	-3.85746	3.884392	-0.99307	0.320678	NA
Drsl2	0	NA	NA	NA	NA	NA
Drsl3	0	NA	NA	NA	NA	NA
Drsl4	0.178812	-1.08808	4.061735	-0.26789	0.788788	NA
Drsl5	0	NA	NA	NA	NA	NA
CG12016	0	NA	NA	NA	NA	NA
CG12010	0	NA	NA	NA	NA	NA
Drsl6	0	NA	NA	NA	NA	NA
Drsl1	0	NA	NA	NA	NA	NA
CG33160	0	NA	NA	NA	NA	NA
CG32277	0	NA	NA	NA	NA	NA
CG33159	0	NA	NA	NA	NA	NA
CG32271	0.178053	-1.16467	4.075067	-0.2858	0.775028	NA
CG14966	45.76658	0.270517	3.850027	0.070264	0.943984	NA
tRNA:Met-	0	NA	NA	NA	NA	NA
Cht7	0	NA	NA	NA	NA	NA
tRNA:Lys-C	0	NA	NA	NA	NA	NA
CG10357	0	NA	NA	NA	NA	NA
CG10862	0	NA	NA	NA	NA	NA
CG14974	0	NA	NA	NA	NA	NA
ImpE2	0	NA	NA	NA	NA	NA
CG32266	21.44256	-3.50308	3.902405	-0.89767	0.36936	NA
Awh	42.05878	0.319444	3.950691	0.080858	0.935555	NA
Gr63a	0	NA	NA	NA	NA	NA
Acp63F	0	NA	NA	NA	NA	NA
CG32263	0	NA	NA	NA	NA	NA
ppk27	1.570499	1.642769	3.980387	0.412716	0.679815	NA
Ugt305A1	0	NA	NA	NA	NA	NA
dib	0	NA	NA	NA	NA	NA
CG32259	24.70788	1.174519	3.95281	0.297135	0.766363	NA
CG14990	0	NA	NA	NA	NA	NA
Akh	5.549096	1.719007	3.959971	0.434096	0.664219	NA
CG32260	0.178053	-1.16467	4.075067	-0.2858	0.775028	NA
CG1299	28.85338	-2.37636	3.824083	-0.62142	0.534324	NA

CG18675	0	NA	NA	NA	NA	NA
Cpr64Ad	0.2094	1.005034	4.045341	0.248442	0.803792	NA
pav	0	NA	NA	NA	NA	NA
CG1273	0	NA	NA	NA	NA	NA
asRNA:CR3	0	NA	NA	NA	NA	NA
Cpr64Ac	0	NA	NA	NA	NA	NA
Cpr64Aa	0.589959	-1.15593	4.01641	-0.2878	0.773499	NA
CG15005	5.15103	-1.89739	3.95832	-0.47934	0.631695	NA
mas	0.46073	-0.17853	4.00793	-0.04454	0.964471	NA
Faa	29.13396	0.70714	3.92688	0.180077	0.857092	NA
Dpy-30L2	0	NA	NA	NA	NA	NA
Claspin	17.58978	4.862249	3.865835	1.257749	0.208483	NA
slow	22.04207	5.969532	3.832821	1.557478	0.119357	NA
CG15020	0	NA	NA	NA	NA	NA
CG15021	0	NA	NA	NA	NA	NA
CG32249	0	NA	NA	NA	NA	NA
CG32248	6.766025	-1.69111	3.956514	-0.42742	0.66907	NA
CG11349	0	NA	NA	NA	NA	NA
CG7465	42.57865	-6.91802	3.832657	-1.80502	0.071072	NA
CG11353	0	NA	NA	NA	NA	NA
CG32246	0	NA	NA	NA	NA	NA
Tie	2.202539	2.272562	3.954673	0.574652	0.565526	NA
CG13720	0	NA	NA	NA	NA	NA
CG13721	0	NA	NA	NA	NA	NA
CG13722	0.132078	-1.13211	4.069918	-0.27817	0.780885	NA
CG32241	0.178053	-1.16467	4.075067	-0.2858	0.775028	NA
CG15024	0	NA	NA	NA	NA	NA
CG15023	0	NA	NA	NA	NA	NA
CG15022	0.1047	0.613261	4.062799	0.150945	0.880019	NA
CG11345	0.178053	-1.16467	4.075067	-0.2858	0.775028	NA
CG32240	0	NA	NA	NA	NA	NA
CG11342	27.55977	3.191976	3.909044	0.816562	0.414179	NA
dyl	0	NA	NA	NA	NA	NA
TTLL1B	43.85347	4.462231	3.748827	1.190301	0.233928	NA
RhoGEF64	53.19271	-4.35312	3.873036	-1.12396	0.261032	NA
CG13713	0	NA	NA	NA	NA	NA
CG13711	0	NA	NA	NA	NA	NA
Dnah3	3.754299	-3.35495	3.965996	-0.84593	0.397592	NA
CG13705	35.5289	-1.91926	3.952005	-0.48564	0.627221	NA
CG13704	6.794867	-2.22911	3.964412	-0.56228	0.573926	NA
CG32235	0	NA	NA	NA	NA	NA
CG32237	0.132078	-1.13211	4.069918	-0.27817	0.780885	NA
CG13708	20.45977	-1.90968	3.739211	-0.51072	0.609549	NA
CG18808	0	NA	NA	NA	NA	NA
CG12493	0	NA	NA	NA	NA	NA
CG13712	0	NA	NA	NA	NA	NA
CG15876	0	NA	NA	NA	NA	NA
CG18418	1.022344	-2.80547	3.981013	-0.70471	0.480989	NA
CG7514	0.178053	-1.16467	4.075067	-0.2858	0.775028	NA
ATPsynCF6	0	NA	NA	NA	NA	NA

CG32232	24.32907	0.933374	3.903833	0.239092	0.811035	NA
CG2157	4.273279	-1.68149	3.959707	-0.42465	0.671092	NA
CG15208	0.1047	0.613261	4.062799	0.150945	0.880019	NA
v	0.178053	-1.16467	4.075067	-0.2858	0.775028	NA
CG2145	50.46788	-6.82068	3.836142	-1.778	0.075403	NA
SmydA-4	0.95493	-0.53313	3.924428	-0.13585	0.89194	NA
C901	1.072874	-2.0339	4.019685	-0.50598	0.612867	NA
Rab9Fb	0	NA	NA	NA	NA	NA
Rab9Fa	0	NA	NA	NA	NA	NA
feo	0.816913	-0.91625	3.980578	-0.23018	0.817951	NA
CG32669	11.0323	-0.23399	3.853354	-0.06072	0.951579	NA
Ant2	41.14964	5.687613	3.840096	1.481112	0.138577	NA
CG2076	0	NA	NA	NA	NA	NA
CG2061	0	NA	NA	NA	NA	NA
CG15200	0	NA	NA	NA	NA	NA
CG11106	0	NA	NA	NA	NA	NA
CG15198	0.104623	0.639075	4.067674	0.157111	0.875158	NA
CG11756	0	NA	NA	NA	NA	NA
CG1738	11.20282	2.941535	3.926668	0.749117	0.453786	NA
sisA	0	NA	NA	NA	NA	NA
CG1394	0.178053	-1.16467	4.075067	-0.2858	0.775028	NA
antdh	0	NA	NA	NA	NA	NA
CG32668	10.69829	-1.91048	3.954523	-0.48311	0.629015	NA
CG15199	0.590718	-0.57492	4.003363	-0.14361	0.885809	NA
ssp7	0.356107	-1.47099	4.037764	-0.36431	0.715628	NA
CG15196	1.960396	-3.42446	3.91235	-0.87529	0.381414	NA
CG11699	0	NA	NA	NA	NA	NA
Reepl1	0	NA	NA	NA	NA	NA
CG2444	0.132078	-1.13211	4.069918	-0.27817	0.780885	NA
CG2533	0.104623	0.639075	4.067674	0.157111	0.875158	NA
CG1561	9.643054	1.306255	3.954754	0.3303	0.741173	NA
CG33235	1.015653	0.790107	3.976718	0.198683	0.842511	NA
Or10a	0	NA	NA	NA	NA	NA
Gr10a	0	NA	NA	NA	NA	NA
Gr10b	0	NA	NA	NA	NA	NA
CG15221	1.989299	1.663439	3.974713	0.418505	0.675578	NA
m	24.44335	-0.39632	3.846162	-0.10304	0.917929	NA
Karl	20.15605	1.648222	3.818865	0.4316	0.666032	NA
CG15739	11.80318	1.681662	3.822762	0.439908	0.660004	NA
CG1840	32.49042	-2.60768	3.813826	-0.68374	0.494137	NA
CG1806	6.920855	1.189058	3.955844	0.300583	0.763733	NA
fw	10.94123	2.606354	3.914804	0.665769	0.505559	NA
CG18130	8.404177	-2.23868	3.961941	-0.56505	0.572043	NA
tsg	0	NA	NA	NA	NA	NA
gd	1.027601	-2.18695	3.915354	-0.55856	0.576463	NA
Sclp	0	NA	NA	NA	NA	NA
lncRNA:CR	0	NA	NA	NA	NA	NA
lncRNA:CR	0	NA	NA	NA	NA	NA
CG15740	0.178053	-1.16467	4.075067	-0.2858	0.775028	NA
CG15741	0	NA	NA	NA	NA	NA

dy	3.336187	-0.97256	3.796706	-0.25616	0.797829	NA
Cyp318a1	0	NA	NA	NA	NA	NA
lncRNA:CR	34.66276	-0.75215	3.836304	-0.19606	0.844563	NA
CG2750	5.440409	1.515841	3.9574	0.38304	0.70169	NA
CG1924	24.73324	0.508069	3.762734	0.135027	0.892591	NA
CG11356	0.732363	1.393579	3.993417	0.348969	0.727112	NA
moon	0	NA	NA	NA	NA	NA
Uch-L5R	0.178053	-1.16467	4.075067	-0.2858	0.775028	NA
Cyp311a1	16.4892	0.545269	3.862506	0.14117	0.887736	NA
CG2577	0.178812	-1.08808	4.061735	-0.26789	0.788788	NA
CG2574	0	NA	NA	NA	NA	NA
CG15927	0.528311	-1.72839	4.011871	-0.43082	0.6666	NA
Cpr11A	0.178812	-1.08808	4.061735	-0.26789	0.788788	NA
Lsp1alpha	0	NA	NA	NA	NA	NA
CG2556	0	NA	NA	NA	NA	NA
Fpgs	10.26059	1.734896	3.955893	0.43856	0.660981	NA
CG11085	0	NA	NA	NA	NA	NA
Mks1	0	NA	NA	NA	NA	NA
CG15731	0	NA	NA	NA	NA	NA
CG32651	0	NA	NA	NA	NA	NA
Ir11a	1.361099	1.628413	3.98439	0.408698	0.682761	NA
CG15728	0	NA	NA	NA	NA	NA
Aven	1.613947	1.895597	3.896457	0.486492	0.626618	NA
CG32642	0	NA	NA	NA	NA	NA
Mur11Da	0	NA	NA	NA	NA	NA
CG15721	7.074029	4.68901	3.889581	1.205531	0.227998	NA
CG32643	0	NA	NA	NA	NA	NA
CG2209	4.816197	1.713907	3.961293	0.432664	0.665259	NA
CG15717	7.075951	0.348699	3.843569	0.090723	0.927713	NA
MFS10	10.27892	-5.98386	3.878613	-1.54278	0.122884	NA
CG15742	0.178053	-1.16467	4.075067	-0.2858	0.775028	NA
CG32639	0	NA	NA	NA	NA	NA
CG12723	0	NA	NA	NA	NA	NA
CG12715	0	NA	NA	NA	NA	NA
CG15719	2.493505	-2.83145	3.945175	-0.7177	0.472943	NA
CG15744	27.08613	-0.40611	3.741707	-0.10854	0.91357	NA
lncRNA:CR	53.93676	-0.8499	3.834632	-0.22164	0.824595	NA
dmrt11E	0	NA	NA	NA	NA	NA
CG15754	0	NA	NA	NA	NA	NA
CG15756	0.356107	-1.47099	4.037764	-0.36431	0.715628	NA
Cpr12A	0.178053	-1.16467	4.075067	-0.2858	0.775028	NA
CG11816	0	NA	NA	NA	NA	NA
CG12725	0	NA	NA	NA	NA	NA
CG32631	0.104623	0.639075	4.067674	0.157111	0.875158	NA
CG32633	0	NA	NA	NA	NA	NA
BthD	0	NA	NA	NA	NA	NA
Tango2	37.23489	0.382619	3.78468	0.101097	0.919474	NA
CG11162	0	NA	NA	NA	NA	NA
CG11158	6.9072	-0.95341	3.846074	-0.24789	0.804219	NA
mus101	4.840354	2.903273	3.920944	0.740452	0.459025	NA

CG10996	0	NA	NA	NA	NA	NA
CG10993	0.837599	1.568055	4.001801	0.391837	0.695178	NA
Ndc80	0.178053	-1.16467	4.075067	-0.2858	0.775028	NA
CG11068	37.42258	-7.43411	3.913063	-1.89982	0.057457	NA
Ste12DOR	0.341478	0.247543	4.031301	0.061405	0.951036	NA
tRNA:Arg-'	0	NA	NA	NA	NA	NA
tRNA:Ser-(	0	NA	NA	NA	NA	NA
tRNA:Ser-(	0	NA	NA	NA	NA	NA
tRNA:Arg-'	0	NA	NA	NA	NA	NA
tRNA:Arg-'	0	NA	NA	NA	NA	NA
tRNA:Arg-'	0	NA	NA	NA	NA	NA
tRNA:Arg-'	0	NA	NA	NA	NA	NA
tRNA:Ser-/	0	NA	NA	NA	NA	NA
tRNA:Ser-/	0	NA	NA	NA	NA	NA
CG12480	0	NA	NA	NA	NA	NA
CG12479	0	NA	NA	NA	NA	NA
tRNA:Ser-/	0	NA	NA	NA	NA	NA
tRNA:Arg-'	0.209323	1.414133	4.032409	0.350692	0.725819	NA
tRNA:Ser-(	0	NA	NA	NA	NA	NA
tRNA:Ser-/	0	NA	NA	NA	NA	NA
tRNA:Ser-/	0	NA	NA	NA	NA	NA
CG1368	0	NA	NA	NA	NA	NA
CG11581	0	NA	NA	NA	NA	NA
CG11584	0	NA	NA	NA	NA	NA
CG32603	0	NA	NA	NA	NA	NA
CG11585	0	NA	NA	NA	NA	NA
yl	0.356107	-1.47099	4.037764	-0.36431	0.715628	NA
betaNACte	16.24556	-1.91474	3.953298	-0.48434	0.628144	NA
betaNACte	0	NA	NA	NA	NA	NA
CG9411	0.104623	0.639075	4.067674	0.157111	0.875158	NA
betaNACte	2.197088	1.483728	3.966465	0.374068	0.708354	NA
betaNACte	0	NA	NA	NA	NA	NA
CG13403	0	NA	NA	NA	NA	NA
CG12540	0	NA	NA	NA	NA	NA
CG5334	2.386561	-0.84224	3.87503	-0.21735	0.827935	NA
CG5347	0	NA	NA	NA	NA	NA
Ag5r	1.636199	1.416598	3.959479	0.357774	0.720512	NA
Ag5r2	0	NA	NA	NA	NA	NA
CG14410	0	NA	NA	NA	NA	NA
dob	50.366	3.758038	3.841771	0.978205	0.327973	NA
CG33177	0	NA	NA	NA	NA	NA
CG33178	0	NA	NA	NA	NA	NA
CG9030	0	NA	NA	NA	NA	NA
CG9503	22.52959	-3.5953	3.920126	-0.91714	0.35907	NA
Eo	0	NA	NA	NA	NA	NA
CG14406	18.23161	2.284207	3.922196	0.582379	0.560311	NA
CG9514	46.61855	-1.92039	3.729988	-0.51485	0.606656	NA
CG9519	0.178053	-1.16467	4.075067	-0.2858	0.775028	NA
CG9522	0	NA	NA	NA	NA	NA
CG12539	0	NA	NA	NA	NA	NA

CG5662	0	NA	NA	NA	NA	NA
dah	46.2728	-1.89472	3.812621	-0.49696	0.619217	NA
Pp1-13C	3.004951	1.268861	3.962825	0.320191	0.748823	NA
CG9114	0	NA	NA	NA	NA	NA
CG9106	0.233093	0.692656	4.076246	0.169925	0.865069	NA
PPYR1	0.932373	1.053996	3.996193	0.26375	0.791972	NA
CG9101	0	NA	NA	NA	NA	NA
CG32588	0	NA	NA	NA	NA	NA
CG9095	2.255529	-2.74072	3.879247	-0.70651	0.479871	NA
CG9072	0.104623	0.639075	4.067674	0.157111	0.875158	NA
CG6294	0	NA	NA	NA	NA	NA
CG15641	0	NA	NA	NA	NA	NA
CG6324	0	NA	NA	NA	NA	NA
Alp11	11.80161	-2.25069	3.958868	-0.56852	0.569683	NA
CG8117	0	NA	NA	NA	NA	NA
CG8119	0	NA	NA	NA	NA	NA
CG12708	0	NA	NA	NA	NA	NA
CG15599	0.178053	-1.16467	4.075067	-0.2858	0.775028	NA
CG15646	0.178053	-1.16467	4.075067	-0.2858	0.775028	NA
CG9213	0	NA	NA	NA	NA	NA
CG15642	59.97658	1.515634	3.951499	0.383559	0.701305	NA
CG8191	1.728708	-4.08437	3.944501	-1.03546	0.300454	NA
CG33172	53.53591	0.647144	3.920233	0.165078	0.868883	NA
lncRNA:CR	2.96562	-1.05157	3.948849	-0.2663	0.790011	NA
CG8565	0	NA	NA	NA	NA	NA
Myb	0	NA	NA	NA	NA	NA
CG32581	53.63374	0.70988	3.926378	0.180798	0.856526	NA
Efhc1.1	33.23222	5.611975	3.80839	1.473582	0.140594	NA
CG8952	0	NA	NA	NA	NA	NA
snRNA:U2:	5.611999	-3.46526	3.88618	-0.89169	0.372559	NA
UQCR-14	0	NA	NA	NA	NA	NA
CG3679	6.996679	3.207923	3.880624	0.826651	0.408435	NA
CG12507	0	NA	NA	NA	NA	NA
snRNA:U5:	0	NA	NA	NA	NA	NA
mei-41	34.89398	2.711137	3.829482	0.707965	0.478967	NA
CG4301	7.50187	-0.36741	3.752704	-0.0979	0.922008	NA
Traf-like	0	NA	NA	NA	NA	NA
CG9981	0.209247	1.042804	4.047837	0.25762	0.7967	NA
CG32573	0	NA	NA	NA	NA	NA
CG4653	0	NA	NA	NA	NA	NA
TwdlX	1.191279	2.587225	3.944347	0.655932	0.511868	NA
TwdlY	1.157666	0.845933	3.944497	0.214459	0.830189	NA
TwdlZ	0.1047	0.613261	4.062799	0.150945	0.880019	NA
CG13005	8.179791	-1.22454	3.954419	-0.30966	0.756817	NA
ppk28	0	NA	NA	NA	NA	NA
CG4872	29.67269	0.452298	3.836636	0.117889	0.906155	NA
CG13002	0	NA	NA	NA	NA	NA
CG4928	0.787578	1.071505	3.94115	0.271876	0.785717	NA
CG33252	0	NA	NA	NA	NA	NA
CG13004	14.33141	-5.1087	3.872918	-1.31908	0.187141	NA

CG9672	20.0718	0.317621	3.951304	0.080384	0.935932	NA
CG9673	4.680909	0.306279	3.955251	0.077436	0.938277	NA
sphe	1.684167	-1.89559	3.906369	-0.48526	0.627495	NA
CG9676	20.72232	-1.00423	3.935683	-0.25516	0.798598	NA
CG13010	0	NA	NA	NA	NA	NA
CG15865	0	NA	NA	NA	NA	NA
CG4949	26.35188	-1.70379	3.952363	-0.43108	0.66641	NA
CG4955	0	NA	NA	NA	NA	NA
CG16700	0	NA	NA	NA	NA	NA
CG4991	0.178053	-1.16467	4.075067	-0.2858	0.775028	NA
CG5070	0	NA	NA	NA	NA	NA
CG32564	0	NA	NA	NA	NA	NA
CG32563	0	NA	NA	NA	NA	NA
CG12998	0.1047	0.613261	4.062799	0.150945	0.880019	NA
CG5172	0	NA	NA	NA	NA	NA
CG10597	0	NA	NA	NA	NA	NA
CG10598	0	NA	NA	NA	NA	NA
CG12995	0.178812	-1.08808	4.061735	-0.26789	0.788788	NA
TTLL1A	0	NA	NA	NA	NA	NA
Arpc3B	47.66106	1.103248	3.951587	0.279191	0.780098	NA
CG8945	0.396233	-1.6814	4.028351	-0.41739	0.676392	NA
CG13000	0	NA	NA	NA	NA	NA
wus	0.1047	0.613261	4.062799	0.150945	0.880019	NA
B-H2	0	NA	NA	NA	NA	NA
B-H1	0.282677	-0.09599	4.057934	-0.02366	0.981127	NA
CG8568	37.93296	1.263721	3.750677	0.336932	0.736169	NA
p-cup	0	NA	NA	NA	NA	NA
CG12994	0	NA	NA	NA	NA	NA
CG8661	0	NA	NA	NA	NA	NA
CG8664	0	NA	NA	NA	NA	NA
unc-4	30.24016	1.289554	3.867131	0.333465	0.738783	NA
CG6398	1.046999	1.598136	3.993006	0.400234	0.688984	NA
Mco4	0.356107	-1.47099	4.037764	-0.36431	0.715628	NA
CG32554	2.229917	-0.25931	3.961959	-0.06545	0.947816	NA
CG8142	41.67252	0.371839	3.835694	0.096942	0.922773	NA
CG12986	0	NA	NA	NA	NA	NA
CG12990	12.99789	-1.69923	3.953847	-0.42977	0.667366	NA
CG12985	0	NA	NA	NA	NA	NA
CG6788	0	NA	NA	NA	NA	NA
Gss1	0.466186	0.959471	4.033986	0.237847	0.812	NA
CR32496	0	NA	NA	NA	NA	NA
CG15373	0	NA	NA	NA	NA	NA
lncRNA:CR	0.732899	1.548046	4.007786	0.38626	0.699304	NA
Him	0	NA	NA	NA	NA	NA
lncRNA:CR	0.989172	0.122741	3.950507	0.03107	0.975214	NA
CG6106	2.544779	-0.269	3.959075	-0.06795	0.945829	NA
CG15056	0	NA	NA	NA	NA	NA
CG6290	0	NA	NA	NA	NA	NA
CG32551	0.178053	-1.16467	4.075067	-0.2858	0.775028	NA
CG32548	0	NA	NA	NA	NA	NA

upd1	0.178053	-1.16467	4.075067	-0.2858	0.775028	NA
CG15059	0.356107	-1.47099	4.037764	-0.36431	0.715628	NA
upd2	3.039809	-2.17312	3.979294	-0.54611	0.584992	NA
Hesr	0	NA	NA	NA	NA	NA
Xrcc2	0	NA	NA	NA	NA	NA
CG15047	0.1047	0.613261	4.062799	0.150945	0.880019	NA
tRNA:Pro-(	0	NA	NA	NA	NA	NA
phm	0	NA	NA	NA	NA	NA
Cyp308a1	0.337793	1.451235	4.039844	0.35923	0.719423	NA
CG12609	0.178053	-1.16467	4.075067	-0.2858	0.775028	NA
CG6873	0	NA	NA	NA	NA	NA
Cyp18a1	0.282753	-0.25398	4.020204	-0.06318	0.949626	NA
CG6696	0.132078	-1.13211	4.069918	-0.27817	0.780885	NA
CG15040	31.09191	0.655504	3.927741	0.166891	0.867456	NA
CG15042	0	NA	NA	NA	NA	NA
CG6470	0.314023	1.768229	4.015745	0.440324	0.659702	NA
CG15044	0	NA	NA	NA	NA	NA
CG15046	1.529126	-1.18057	3.969053	-0.29744	0.766127	NA
psh	0.1047	0.613261	4.062799	0.150945	0.880019	NA
Hayan	0	NA	NA	NA	NA	NA
CR6900	47.63831	3.559934	3.903583	0.911966	0.361787	NA
Pvf1	0.770937	-1.65034	3.976655	-0.41501	0.678137	NA
SdhBL	0.314023	1.768229	4.015745	0.440324	0.659702	NA
CG14190	0	NA	NA	NA	NA	NA
CG7406	0	NA	NA	NA	NA	NA
CG14191	0	NA	NA	NA	NA	NA
CG33253	0.282753	-0.25398	4.020204	-0.06318	0.949626	NA
Mec2	0	NA	NA	NA	NA	NA
HP1D3csd	0	NA	NA	NA	NA	NA
Mur18B	0	NA	NA	NA	NA	NA
Muc18B	0	NA	NA	NA	NA	NA
CG7884	0	NA	NA	NA	NA	NA
CG8051	0.178053	-1.16467	4.075067	-0.2858	0.775028	NA
CG8034	0.1047	0.613261	4.062799	0.150945	0.880019	NA
CG8028	0	NA	NA	NA	NA	NA
CG7992	0	NA	NA	NA	NA	NA
CG14196	4.816197	1.713907	3.961293	0.432664	0.665259	NA
CG14195	32.4569	2.969217	3.920388	0.757378	0.448823	NA
CG7914	14.5227	0.316307	3.951755	0.080042	0.936204	NA
Inx5	0	NA	NA	NA	NA	NA
CG15882	0	NA	NA	NA	NA	NA
CG7423	0	NA	NA	NA	NA	NA
narya	8.549286	4.495795	3.877208	1.159544	0.246234	NA
CG14204	0.178053	-1.16467	4.075067	-0.2858	0.775028	NA
CG14205	0.357625	-1.47707	4.044794	-0.36518	0.714979	NA
CG14212	0	NA	NA	NA	NA	NA
Zw	4.167137	-2.13584	3.890062	-0.54905	0.582972	NA
CG14227	53.87678	-2.20788	3.952601	-0.55859	0.576442	NA
et	27.08669	0.318515	3.951	0.080616	0.935747	NA
CG14223	0.236778	-0.26946	4.071573	-0.06618	0.947234	NA

CG14221	0.368779	-0.59727	3.999361	-0.14934	0.881285	NA
CG14219	0.523116	1.34877	4.007682	0.336546	0.736459	NA
CG14218	0	NA	NA	NA	NA	NA
CG12231	0	NA	NA	NA	NA	NA
CG14230	61.28999	0.451195	3.846158	0.11731	0.906614	NA
SkpD	0	NA	NA	NA	NA	NA
SkpC	0	NA	NA	NA	NA	NA
SkpE	0	NA	NA	NA	NA	NA
CG12702	0	NA	NA	NA	NA	NA
meso18E	0.578178	0.709574	3.962639	0.179066	0.857886	NA
CG14185	0	NA	NA	NA	NA	NA
CG6996	0.233093	0.692656	4.076246	0.169925	0.865069	NA
CG17145	0	NA	NA	NA	NA	NA
CG13813	9.843966	-1.74148	3.755972	-0.46366	0.642895	NA
CG32228	3.734244	-3.93648	3.895617	-1.01049	0.31226	NA
Spn77Bb	0.178053	-1.16467	4.075067	-0.2858	0.775028	NA
CR6434	1.296331	0.32833	3.890767	0.084387	0.932749	NA
CG17122	0	NA	NA	NA	NA	NA
CG7017	3.204959	-1.67301	3.96255	-0.42221	0.672875	NA
asRNA:CR3	0	NA	NA	NA	NA	NA
CG7290	0	NA	NA	NA	NA	NA
CG7298	0	NA	NA	NA	NA	NA
obst-F	0	NA	NA	NA	NA	NA
CG7335	0	NA	NA	NA	NA	NA
obst-J	0	NA	NA	NA	NA	NA
CG7365	0	NA	NA	NA	NA	NA
CG14187	53.67173	1.537006	3.951579	0.38896	0.697306	NA
Ir76b	0.104623	0.639075	4.067674	0.157111	0.875158	NA
CG17732	0.620262	-2.71754	3.999923	-0.6794	0.496886	NA
schuy	44.46488	1.536494	3.951717	0.388817	0.697412	NA
hale	0.414754	-1.14733	4.015515	-0.28573	0.775088	NA
CG14104	31.23582	0.74645	3.836369	0.194572	0.845728	NA
Cpr76Bc	0	NA	NA	NA	NA	NA
Cpr76Bd	0.1047	0.613261	4.062799	0.150945	0.880019	NA
Rcd7	1.430498	-2.08135	4.005348	-0.51964	0.603313	NA
CG9300	0.236778	-0.26946	4.071573	-0.06618	0.947234	NA
ms(3)76Cc	0	NA	NA	NA	NA	NA
Gbeta76C	3.893142	0.325078	3.852087	0.08439	0.932746	NA
ms(3)76Ca	0	NA	NA	NA	NA	NA
lush	0	NA	NA	NA	NA	NA
CG9279	0	NA	NA	NA	NA	NA
CG9666	0	NA	NA	NA	NA	NA
CG14085	0.178053	-1.16467	4.075067	-0.2858	0.775028	NA
CG14086	0.847327	-2.62258	4.005321	-0.65477	0.512614	NA
ms(3)76Ba	1.251686	-2.06022	4.011654	-0.51356	0.60756	NA
CR33255	0	NA	NA	NA	NA	NA
hpRNA:CR:	4.411568	-1.49887	3.862047	-0.3881	0.69794	NA
pip	1.180677	-1.30885	3.976465	-0.32915	0.742044	NA
CG14089	0	NA	NA	NA	NA	NA
CG14088	0	NA	NA	NA	NA	NA

CG6836	35.04917	4.270749	3.883235	1.099791	0.271423	NA
CG14075	0.679117	1.734471	3.96601	0.437334	0.661869	NA
CG14079	0.178053	-1.16467	4.075067	-0.2858	0.775028	NA
Spn75F	0	NA	NA	NA	NA	NA
Acp76A	0.1047	0.613261	4.062799	0.150945	0.880019	NA
SmydA-2	19.05246	-2.92317	3.920759	-0.74556	0.455931	NA
CG3819	27.81959	0.318582	3.950977	0.080634	0.935733	NA
CG6893	0	NA	NA	NA	NA	NA
CG6885	0.414754	-1.14733	4.015515	-0.28573	0.775088	NA
CG6865	0	NA	NA	NA	NA	NA
NijB	0.283512	-0.2331	4.065094	-0.05734	0.954273	NA
CG6839	0.178053	-1.16467	4.075067	-0.2858	0.775028	NA
CG18223	0	NA	NA	NA	NA	NA
CG14077	0	NA	NA	NA	NA	NA
Ir75d	0.264155	-1.54099	4.048976	-0.38059	0.703509	NA
Rad9	42.63364	-1.88881	3.951801	-0.47796	0.632678	NA
CG12477	0.178053	-1.16467	4.075067	-0.2858	0.775028	NA
Dic4	0	NA	NA	NA	NA	NA
Cyp12c1	0	NA	NA	NA	NA	NA
CG32198	0.233093	0.692656	4.076246	0.169925	0.865069	NA
CG7271	0.2094	1.005034	4.045341	0.248442	0.803792	NA
CG18233	0.1047	0.613261	4.062799	0.150945	0.880019	NA
CG18231	0.264155	-1.54099	4.048976	-0.38059	0.703509	NA
CG32201	0	NA	NA	NA	NA	NA
CG32199	0.356107	-1.47099	4.037764	-0.36431	0.715628	NA
tRNA:Pro-(	0	NA	NA	NA	NA	NA
term	0.178812	-1.08808	4.061735	-0.26789	0.788788	NA
Met75Ca	0	NA	NA	NA	NA	NA
Met75Cb	0	NA	NA	NA	NA	NA
CG4306	0	NA	NA	NA	NA	NA
geko	26.67969	-1.918	3.952367	-0.48528	0.627479	NA
CG7330	6.207651	-1.90163	3.957084	-0.48056	0.630826	NA
CG7320	0	NA	NA	NA	NA	NA
grim	6.347558	-0.81697	3.860925	-0.2116	0.832419	NA
CG13700	0	NA	NA	NA	NA	NA
CG5103	2.453201	-4.08442	3.895709	-1.04844	0.294436	NA
CG13699	0.178053	-1.16467	4.075067	-0.2858	0.775028	NA
Cyp312a1	0	NA	NA	NA	NA	NA
lncRNA:CR	0	NA	NA	NA	NA	NA
CG7408	47.55102	1.177227	3.951898	0.297889	0.765788	NA
CG7402	0	NA	NA	NA	NA	NA
Tsp74F	0	NA	NA	NA	NA	NA
CG16775	0	NA	NA	NA	NA	NA
CG5506	0	NA	NA	NA	NA	NA
CG5567	49.36089	-7.70823	3.843115	-2.00572	0.044886	NA
CG32189	0	NA	NA	NA	NA	NA
CG32188	0	NA	NA	NA	NA	NA
Ir75a	0	NA	NA	NA	NA	NA
Cln3	0	NA	NA	NA	NA	NA
edin	0	NA	NA	NA	NA	NA

CG7542	0	NA	NA	NA	NA	NA
CG32182	0	NA	NA	NA	NA	NA
CG32181	0	NA	NA	NA	NA	NA
Adgf-A	2.346336	-0.97604	3.932042	-0.24823	0.803959	NA
Adgf-A2	1.557478	-1.35228	3.96773	-0.34082	0.733239	NA
Adgf-B	0.178053	-1.16467	4.075067	-0.2858	0.775028	NA
CG6034	0	NA	NA	NA	NA	NA
CG6052	14.13449	1.740196	3.954547	0.440049	0.659901	NA
Jon74E	28.17852	-3.51055	3.901177	-0.89987	0.36819	NA
CG7707	0	NA	NA	NA	NA	NA
CG13723	0	NA	NA	NA	NA	NA
CG13724	0.178812	-1.08808	4.061735	-0.26789	0.788788	NA
tap	6.386696	1.723466	3.95882	0.435348	0.66331	NA
SecCl	0	NA	NA	NA	NA	NA
qjt	1.609311	-2.0987	4.000255	-0.52464	0.599832	NA
CG6333	0.178053	-1.16467	4.075067	-0.2858	0.775028	NA
CG13733	0	NA	NA	NA	NA	NA
CG12229	1.387557	0.928325	3.968008	0.233952	0.815022	NA
Coq4	0.418493	1.313669	4.019283	0.326842	0.743788	NA
frc	0	NA	NA	NA	NA	NA
blot	15.78941	-2.00358	3.760538	-0.53279	0.594178	NA
CG13731	0	NA	NA	NA	NA	NA
CG13728	0.462324	-0.59023	4.044233	-0.14594	0.883966	NA
Cad74A	3.428902	-0.09856	3.948141	-0.02496	0.980084	NA
brv2	0.387453	0.098632	4.002797	0.024641	0.980342	NA
CG13727	0.178053	-1.16467	4.075067	-0.2858	0.775028	NA
tRNA:Pro-(	0	NA	NA	NA	NA	NA
Or74a	0.1047	0.613261	4.062799	0.150945	0.880019	NA
CG13725	0.356107	-1.47099	4.037764	-0.36431	0.715628	NA
ND-24L	0	NA	NA	NA	NA	NA
CG6497	2.895532	1.407909	3.855446	0.365174	0.714981	NA
CG32170	0.469871	0.864471	4.023538	0.214854	0.829881	NA
CG13028	0	NA	NA	NA	NA	NA
Fit2	50.34066	2.476387	3.842284	0.644509	0.519245	NA
CG7724	4.292697	1.709267	3.962502	0.431361	0.666206	NA
Ilp8	0	NA	NA	NA	NA	NA
CG6652	0.356107	-1.47099	4.037764	-0.36431	0.715628	NA
CG13022	0	NA	NA	NA	NA	NA
CG11905	0.1047	0.613261	4.062799	0.150945	0.880019	NA
CG13023	0	NA	NA	NA	NA	NA
snRNA:U1:	0	NA	NA	NA	NA	NA
CG13032	1.945653	-0.39222	3.885108	-0.10095	0.919587	NA
CG9692	0	NA	NA	NA	NA	NA
CG13031	0	NA	NA	NA	NA	NA
PGRP-SB2	0	NA	NA	NA	NA	NA
CG9701	0	NA	NA	NA	NA	NA
CG32161	0	NA	NA	NA	NA	NA
CG13024	0.178053	-1.16467	4.075067	-0.2858	0.775028	NA
Dbp73D	25.62709	-7.41624	3.907554	-1.89792	0.057706	NA
PGRP-SB1	1.958586	-1.65227	3.969602	-0.41623	0.677241	NA

CG13026	0	NA	NA	NA	NA	NA
Lasp	0	NA	NA	NA	NA	NA
Galphaf	26.59378	1.746911	3.952855	0.441936	0.658535	NA
CG4098	50.45739	0.169876	3.501337	0.048517	0.961304	NA
spd-2	0.411147	-0.29058	4.046973	-0.0718	0.94276	NA
CG13063	0	NA	NA	NA	NA	NA
lncRNA:CR	0	NA	NA	NA	NA	NA
CG13060	0	NA	NA	NA	NA	NA
CG13058	0	NA	NA	NA	NA	NA
CG33061	0	NA	NA	NA	NA	NA
CG33060	0	NA	NA	NA	NA	NA
retinin	43.13629	2.972992	3.919545	0.758504	0.448149	NA
CG13056	39.75538	-1.91967	3.951888	-0.48576	0.627137	NA
CG4998	0	NA	NA	NA	NA	NA
CG33257	0	NA	NA	NA	NA	NA
tRNA:Met-	0	NA	NA	NA	NA	NA
Cpr72Ea	0	NA	NA	NA	NA	NA
Cpr72Eb	0	NA	NA	NA	NA	NA
CG4842	0	NA	NA	NA	NA	NA
CG18814	0	NA	NA	NA	NA	NA
Gagr	0	NA	NA	NA	NA	NA
st	50.10738	-0.01706	3.262872	-0.00523	0.995828	NA
CG4229	12.99789	-1.69923	3.953847	-0.42977	0.667366	NA
Mipp1	0.732899	1.548046	4.007786	0.38626	0.699304	NA
CG13033	0.132078	-1.13211	4.069918	-0.27817	0.780885	NA
aos	0.178053	-1.16467	4.075067	-0.2858	0.775028	NA
GluRS-m	0.1047	0.613261	4.062799	0.150945	0.880019	NA
Agpat4	0	NA	NA	NA	NA	NA
Cpr72Ec	0	NA	NA	NA	NA	NA
CG13040	0	NA	NA	NA	NA	NA
CG13041	0	NA	NA	NA	NA	NA
CG13042	0.178053	-1.16467	4.075067	-0.2858	0.775028	NA
CG13043	0	NA	NA	NA	NA	NA
CG13075	9.715876	0.790869	3.749971	0.2109	0.832965	NA
ATPsynbet	0	NA	NA	NA	NA	NA
tRNA:Met-	0	NA	NA	NA	NA	NA
CG13071	0	NA	NA	NA	NA	NA
CG13070	0	NA	NA	NA	NA	NA
CG13069	0.178053	-1.16467	4.075067	-0.2858	0.775028	NA
CG13068	0.178053	-1.16467	4.075067	-0.2858	0.775028	NA
CG13067	0	NA	NA	NA	NA	NA
CG13066	0	NA	NA	NA	NA	NA
CG13065	0	NA	NA	NA	NA	NA
CG4982	0	NA	NA	NA	NA	NA
CG13045	0	NA	NA	NA	NA	NA
CG13046	0	NA	NA	NA	NA	NA
CG13047	0.178812	-1.08808	4.061735	-0.26789	0.788788	NA
CG13048	0	NA	NA	NA	NA	NA
CG13049	0.178053	-1.16467	4.075067	-0.2858	0.775028	NA
CG13050	0	NA	NA	NA	NA	NA

CG4950	1.228742	-0.20401	3.923681	-0.052	0.958532	NA
CG13053	37.03508	-1.70508	3.951944	-0.43145	0.666139	NA
CG13054	0	NA	NA	NA	NA	NA
CG13055	0.597612	0.189345	3.999077	0.047347	0.962237	NA
CG13073	0	NA	NA	NA	NA	NA
CG32152	0	NA	NA	NA	NA	NA
CG5157	0	NA	NA	NA	NA	NA
CG13074	2.124287	-3.00082	3.903937	-0.76867	0.442092	NA
CG5235	12.66869	1.738562	3.954961	0.43959	0.660234	NA
CG6244	23.32498	-1.70323	3.952551	-0.43092	0.666526	NA
CG13445	0	NA	NA	NA	NA	NA
CG17028	51.25665	0.217308	3.834572	0.056671	0.954807	NA
meru	0.598264	-0.35879	4.007615	-0.08953	0.928662	NA
CG7276	0	NA	NA	NA	NA	NA
yellow-k	0	NA	NA	NA	NA	NA
Eig71Eb	0.178053	-1.16467	4.075067	-0.2858	0.775028	NA
Eig71Ed	0.104623	0.639075	4.067674	0.157111	0.875158	NA
Eig71Eg	0	NA	NA	NA	NA	NA
Eig71Ei	0	NA	NA	NA	NA	NA
Eig71Ek	0	NA	NA	NA	NA	NA
CG7304	0	NA	NA	NA	NA	NA
Pgant8	0	NA	NA	NA	NA	NA
comm2	0	NA	NA	NA	NA	NA
CG7579	0	NA	NA	NA	NA	NA
Eig71Ej	0	NA	NA	NA	NA	NA
Eig71Eh	0	NA	NA	NA	NA	NA
Eig71Ef	0	NA	NA	NA	NA	NA
Eig71Ee	0	NA	NA	NA	NA	NA
Eig71Ec	0.178053	-1.16467	4.075067	-0.2858	0.775028	NA
Eig71Ea	0	NA	NA	NA	NA	NA
CG13449	0.233093	0.692656	4.076246	0.169925	0.865069	NA
mex1	0	NA	NA	NA	NA	NA
CG13454	0	NA	NA	NA	NA	NA
Best4	0	NA	NA	NA	NA	NA
CG33259	0.233093	0.692656	4.076246	0.169925	0.865069	NA
Ran-like	0	NA	NA	NA	NA	NA
cocoon	0	NA	NA	NA	NA	NA
Best3	0	NA	NA	NA	NA	NA
Tom	0	NA	NA	NA	NA	NA
Brd	0	NA	NA	NA	NA	NA
Ocho	0	NA	NA	NA	NA	NA
CG3349	0.461565	-1.07936	4.002266	-0.26969	0.7874	NA
CG32148	0	NA	NA	NA	NA	NA
EAChm	0	NA	NA	NA	NA	NA
CG32847	0	NA	NA	NA	NA	NA
CG6888	0	NA	NA	NA	NA	NA
CG13457	0.71449	-2.47883	4.014121	-0.61753	0.536887	NA
CG16959	0	NA	NA	NA	NA	NA
CG13460	0	NA	NA	NA	NA	NA
CG18649	0	NA	NA	NA	NA	NA

CG13461	0	NA	NA	NA	NA	NA
CG12310	0	NA	NA	NA	NA	NA
CG18581	0	NA	NA	NA	NA	NA
ind	0.283512	-0.2331	4.065094	-0.05734	0.954273	NA
Rpn12R	0	NA	NA	NA	NA	NA
Reck	14.18074	-6.2064	3.89028	-1.59536	0.110632	NA
BobA	0	NA	NA	NA	NA	NA
CG13465	0	NA	NA	NA	NA	NA
shd	0	NA	NA	NA	NA	NA
dlp	52.72043	0.619272	3.841596	0.161202	0.871934	NA
tRNA:iMet	0	NA	NA	NA	NA	NA
CG5048	0	NA	NA	NA	NA	NA
goddard	0	NA	NA	NA	NA	NA
CG13476	0	NA	NA	NA	NA	NA
CG13473	0	NA	NA	NA	NA	NA
gnu	0	NA	NA	NA	NA	NA
Magt4b	0.536437	-1.71175	4.038042	-0.42391	0.671635	NA
CG13471	0	NA	NA	NA	NA	NA
CG17177	0	NA	NA	NA	NA	NA
CG13474	0	NA	NA	NA	NA	NA
CG4914	0	NA	NA	NA	NA	NA
tRNA:iMet	0	NA	NA	NA	NA	NA
CG9592	0	NA	NA	NA	NA	NA
HGTX	29.13665	1.175374	3.952522	0.297373	0.766182	NA
Lk	0	NA	NA	NA	NA	NA
CG4613	0	NA	NA	NA	NA	NA
CG3868	0	NA	NA	NA	NA	NA
CG13481	0	NA	NA	NA	NA	NA
saturn	0	NA	NA	NA	NA	NA
CG7924	0.356107	-1.47099	4.037764	-0.36431	0.715628	NA
CG7906	0	NA	NA	NA	NA	NA
CG13482	5.413809	0.308289	3.954543	0.077958	0.937861	NA
Fbp1	1.487119	-0.47654	3.965238	-0.12018	0.904342	NA
Tsp68C	0	NA	NA	NA	NA	NA
Hml	34.03076	-6.55315	3.869613	-1.69349	0.090362	NA
CG13737	41.57107	-0.41604	3.941747	-0.10555	0.915941	NA
ssp2	0	NA	NA	NA	NA	NA
Hsc70-1	0.712213	-1.57119	3.998613	-0.39293	0.694369	NA
CG17362	0	NA	NA	NA	NA	NA
CG9040	0	NA	NA	NA	NA	NA
CG6661	0	NA	NA	NA	NA	NA
Pex1	48.02639	-0.87637	3.948549	-0.22195	0.824354	NA
CG8100	0	NA	NA	NA	NA	NA
CG13738	1.154422	-3.36701	3.968232	-0.84849	0.396164	NA
CG8750	0	NA	NA	NA	NA	NA
CG8757	6.161573	0.633982	3.820035	0.165962	0.868186	NA
CG10738	37.18266	-6.09488	3.823513	-1.59405	0.110924	NA
CG10710	3.853845	-0.30875	3.956334	-0.07804	0.937796	NA
CG10116	0.104623	0.639075	4.067674	0.157111	0.875158	NA
CG33262	0	NA	NA	NA	NA	NA

tRNA:Pro-/	0	NA	NA	NA	NA	NA
tRNA:Pro-/	0	NA	NA	NA	NA	NA
tRNA:Pro-/	0	NA	NA	NA	NA	NA
tRNA:Leu-(	0	NA	NA	NA	NA	NA
tRNA:Asp-(	0	NA	NA	NA	NA	NA
SP	6.759704	1.159951	3.957776	0.293082	0.76946	NA
CG14113	0	NA	NA	NA	NA	NA
CG17687	0.178053	-1.16467	4.075067	-0.2858	0.775028	NA
SNCF	0	NA	NA	NA	NA	NA
CG14111	0	NA	NA	NA	NA	NA
ImpL1	0	NA	NA	NA	NA	NA
CG14110	0.233093	0.692656	4.076246	0.169925	0.865069	NA
tRNA:Val-/	0.178812	-1.08808	4.061735	-0.26789	0.788788	NA
CG14109	0	NA	NA	NA	NA	NA
CG10140	0.178053	-1.16467	4.075067	-0.2858	0.775028	NA
CG10154	0	NA	NA	NA	NA	NA
tRNA:Val-/	0	NA	NA	NA	NA	NA
CG14105	2.121376	0.634749	3.940869	0.161068	0.87204	NA
CG14106	0.356107	-1.47099	4.037764	-0.36431	0.715628	NA
CG33263	0	NA	NA	NA	NA	NA
CG32121	57.86031	-2.25893	3.898804	-0.57939	0.562325	NA
CG10222	33.40808	1.599386	3.815985	0.419128	0.675123	NA
sens	0.660388	-1.75977	4.001266	-0.4398	0.660079	NA
Poc1	0.178053	-1.16467	4.075067	-0.2858	0.775028	NA
CG14107	0	NA	NA	NA	NA	NA
CG32119	0	NA	NA	NA	NA	NA
CG14120	0.567024	-0.28781	3.989037	-0.07215	0.942482	NA
CG14118	0.178053	-1.16467	4.075067	-0.2858	0.775028	NA
CG10749	0	NA	NA	NA	NA	NA
CG11262	0	NA	NA	NA	NA	NA
CR14117	7.611441	3.315511	3.918634	0.846088	0.397503	NA
ste14	7.884683	0.438792	3.857335	0.113755	0.909432	NA
CG11251	0	NA	NA	NA	NA	NA
CG17300	0	NA	NA	NA	NA	NA
CG14115	0.104623	0.639075	4.067674	0.157111	0.875158	NA
Cul6	0	NA	NA	NA	NA	NA
CG10748	0.955237	-0.24643	3.926835	-0.06275	0.949962	NA
Or69a	0	NA	NA	NA	NA	NA
CG10752	0	NA	NA	NA	NA	NA
CG32115	12.87809	1.738817	3.954896	0.439662	0.660182	NA
CG32117	0	NA	NA	NA	NA	NA
CG12520	0	NA	NA	NA	NA	NA
lncRNA:CR	25.07789	0.224222	3.836219	0.058449	0.953391	NA
CG17666	0.178053	-1.16467	4.075067	-0.2858	0.775028	NA
CG32110	0	NA	NA	NA	NA	NA
CG10943	0.1047	0.613261	4.062799	0.150945	0.880019	NA
CG32107	3.306991	0.324254	3.865979	0.083874	0.933157	NA
CG10969	0	NA	NA	NA	NA	NA
CG32106	0	NA	NA	NA	NA	NA
Obp69a	0.104623	0.639075	4.067674	0.157111	0.875158	NA

CG10657	0	NA	NA	NA	NA	NA
CG10663	1.256399	1.619735	3.986836	0.406271	0.684544	NA
CG32102	0	NA	NA	NA	NA	NA
toe	17.06609	1.74264	3.95393	0.440736	0.659404	NA
Smyd4-2	0	NA	NA	NA	NA	NA
CG17154	0	NA	NA	NA	NA	NA
DEF8	44.16891	2.265868	3.820182	0.593131	0.553093	NA
CAH2	0.282753	-0.25398	4.020204	-0.06318	0.949626	NA
Est-P	0	NA	NA	NA	NA	NA
CG11529	6.86804	-1.90364	3.956501	-0.48114	0.630416	NA
Ir68b	0.233093	0.692656	4.076246	0.169925	0.865069	NA
CG10907	61.1854	-4.04898	3.863031	-1.04813	0.294576	NA
CG7264	2.560185	3.612111	3.952886	0.913791	0.360827	NA
Sprn	0.565506	-0.26224	3.986711	-0.06578	0.947554	NA
CCDC151	0	NA	NA	NA	NA	NA
CG11588	0.31089	-1.9003	4.033427	-0.47114	0.637542	NA
Rpt4R	0	NA	NA	NA	NA	NA
CG7252	0.178053	-1.16467	4.075067	-0.2858	0.775028	NA
CG17826	0	NA	NA	NA	NA	NA
CG7248	0	NA	NA	NA	NA	NA
CG14125	0	NA	NA	NA	NA	NA
CG6938	0.1047	0.613261	4.062799	0.150945	0.880019	NA
CG6931	0.104623	0.639075	4.067674	0.157111	0.875158	NA
Muc68E	0.104623	0.639075	4.067674	0.157111	0.875158	NA
prc	1.246373	-1.6238	3.979526	-0.40804	0.683245	NA
CG11570	0	NA	NA	NA	NA	NA
obst-G	0.178053	-1.16467	4.075067	-0.2858	0.775028	NA
CG5883	0	NA	NA	NA	NA	NA
SdhAL	0	NA	NA	NA	NA	NA
CG12521	0	NA	NA	NA	NA	NA
tRNA:Gln-ˉ	0	NA	NA	NA	NA	NA
CG5964	0	NA	NA	NA	NA	NA
Tim13	0	NA	NA	NA	NA	NA
Vha16-3	0	NA	NA	NA	NA	NA
Vha16-2	0	NA	NA	NA	NA	NA
CG33500	0	NA	NA	NA	NA	NA
Sgs7	0	NA	NA	NA	NA	NA
Sgs3	0.178053	-1.16467	4.075067	-0.2858	0.775028	NA
CG7512	0	NA	NA	NA	NA	NA
CG7377	0	NA	NA	NA	NA	NA
CG32087	0.357625	-1.47707	4.044794	-0.36518	0.714979	NA
Gr68a	0	NA	NA	NA	NA	NA
CG14132	1.452855	-1.83946	3.975716	-0.46267	0.643599	NA
crim	5.549096	1.719007	3.959971	0.434096	0.664219	NA
Dnai2	0	NA	NA	NA	NA	NA
Pldn	54.52358	-1.63077	3.835842	-0.42514	0.670734	NA
CG6071	3.830253	-1.88895	3.960794	-0.47691	0.633424	NA
Muc68Ca	1.743906	0.331025	3.909891	0.084664	0.932529	NA
CG14137	0.209247	1.042804	4.047837	0.25762	0.7967	NA
CG33268	0.104623	0.639075	4.067674	0.157111	0.875158	NA

CG32086	0	NA	NA	NA	NA	NA
CG33269	2.796185	-0.43502	3.958512	-0.10989	0.912493	NA
CG33270	30.43823	0.793074	3.951793	0.200687	0.840943	NA
CG33271	0.1047	0.613261	4.062799	0.150945	0.880019	NA
CG33272	0.2094	1.005034	4.045341	0.248442	0.803792	NA
Sgs8	0	NA	NA	NA	NA	NA
CG7573	0.233093	0.692656	4.076246	0.169925	0.865069	NA
CG11726	0.178053	-1.16467	4.075067	-0.2858	0.775028	NA
CG7560	0	NA	NA	NA	NA	NA
CG7557	0	NA	NA	NA	NA	NA
CG7551	0	NA	NA	NA	NA	NA
Fum3	0.283436	-0.20033	4.070792	-0.04921	0.960751	NA
CG12289	0	NA	NA	NA	NA	NA
CG6149	0	NA	NA	NA	NA	NA
CG6168	0	NA	NA	NA	NA	NA
Cpr67Fa1	0	NA	NA	NA	NA	NA
Cpr67Fa2	0	NA	NA	NA	NA	NA
Cpr67Fb	0	NA	NA	NA	NA	NA
CG14147	0	NA	NA	NA	NA	NA
Fad2	0	NA	NA	NA	NA	NA
CG32079	0	NA	NA	NA	NA	NA
CG32081	0	NA	NA	NA	NA	NA
SuUR	0	NA	NA	NA	NA	NA
CG12522	0	NA	NA	NA	NA	NA
nol	27.35154	0.351828	3.838529	0.091657	0.926971	NA
CG14143	0	NA	NA	NA	NA	NA
IRSp53	10.45349	-2.27299	3.895083	-0.58355	0.559521	NA
CG6216	0	NA	NA	NA	NA	NA
CG32074	0	NA	NA	NA	NA	NA
CG32073	0	NA	NA	NA	NA	NA
CG32071	0	NA	NA	NA	NA	NA
Elo68beta	12.51686	-2.25242	3.958427	-0.56902	0.569342	NA
klu	2.315095	-3.77244	3.930617	-0.95976	0.337177	NA
CG18628	37.37177	-2.27194	3.953479	-0.57467	0.565515	NA
CG14154	0.915764	-1.24472	3.920815	-0.31746	0.750892	NA
CG12523	0	NA	NA	NA	NA	NA
CG6409	50.3216	-1.92039	3.951682	-0.48597	0.62699	NA
CG14151	0	NA	NA	NA	NA	NA
CG12362	0	NA	NA	NA	NA	NA
CG32061	0	NA	NA	NA	NA	NA
S-Lap3	0.282753	-0.25398	4.020204	-0.06318	0.949626	NA
S-Lap4	1.17302	-1.0781	3.973292	-0.27134	0.786132	NA
Gem3	0.209323	1.414133	4.032409	0.350692	0.725819	NA
Ir67c	0	NA	NA	NA	NA	NA
Ir67b	0	NA	NA	NA	NA	NA
CG8072	39.88746	-1.91968	3.951885	-0.48576	0.627135	NA
CG8065	0	NA	NA	NA	NA	NA
Or67d	0.104623	0.639075	4.067674	0.157111	0.875158	NA
can	20.70922	-0.66171	3.927095	-0.1685	0.866191	NA
Or67c	0.209247	1.042804	4.047837	0.25762	0.7967	NA

ect	0	NA	NA	NA	NA	NA
CG6628	0.5892	-0.44935	3.992199	-0.11256	0.910381	NA
CG32054	0	NA	NA	NA	NA	NA
CG32053	0	NA	NA	NA	NA	NA
CG14160	0.660388	-1.75977	4.001266	-0.4398	0.660079	NA
Ilp1	0	NA	NA	NA	NA	NA
Ilp2	1.150856	1.439869	3.979295	0.36184	0.717471	NA
Hez	0	NA	NA	NA	NA	NA
CG6709	0	NA	NA	NA	NA	NA
Ilp4	3.90674	1.303404	3.85744	0.337893	0.735443	NA
Ilp3	0.178053	-1.16467	4.075067	-0.2858	0.775028	NA
Or67a	56.01438	2.975657	3.918953	0.759299	0.447674	NA
CG3222	0	NA	NA	NA	NA	NA
CG3306	0	NA	NA	NA	NA	NA
CG8329	0	NA	NA	NA	NA	NA
CG18179	0	NA	NA	NA	NA	NA
CG18180	0.178053	-1.16467	4.075067	-0.2858	0.775028	NA
CG16719	0	NA	NA	NA	NA	NA
CG6749	39.33834	1.536107	3.951823	0.388708	0.697492	NA
Or67b	0.209247	1.042804	4.047837	0.25762	0.7967	NA
CG3088	0	NA	NA	NA	NA	NA
Ir67a	0.178053	-1.16467	4.075067	-0.2858	0.775028	NA
CG4476	9.102223	1.525146	3.954825	0.385642	0.699762	NA
CG4477	0	NA	NA	NA	NA	NA
CG32040	16.62954	-2.25959	3.956611	-0.57109	0.567936	NA
CG4461	0	NA	NA	NA	NA	NA
CG32037	1.06832	-1.61145	3.983918	-0.40449	0.685853	NA
CG32036	0.178812	-1.08808	4.061735	-0.26789	0.788788	NA
CG3437	1.188699	-1.8233	3.980741	-0.45803	0.646931	NA
CG3448	0	NA	NA	NA	NA	NA
CG3982	0.6282	1.523021	4.015426	0.379293	0.704471	NA
Klp67A	39.72894	2.852469	3.814609	0.747775	0.454596	NA
CG4483	0	NA	NA	NA	NA	NA
tRNA:Thr-/	0	NA	NA	NA	NA	NA
GNBP3	42.19407	1.749778	3.952138	0.442742	0.657952	NA
CG13314	0	NA	NA	NA	NA	NA
Doc3	0	NA	NA	NA	NA	NA
Doc1	0	NA	NA	NA	NA	NA
CG13315	54.53546	2.089514	3.81177	0.548174	0.583572	NA
CG5194	0	NA	NA	NA	NA	NA
CG5280	0	NA	NA	NA	NA	NA
CG5653	0.178812	-1.08808	4.061735	-0.26789	0.788788	NA
ValRS-m	20.56194	-6.87598	3.848295	-1.78676	0.073976	NA
Cpr66D	0	NA	NA	NA	NA	NA
Pex7	2.601344	-1.94058	3.843265	-0.50493	0.613608	NA
Cp18	47.86426	-0.73412	3.838201	-0.19127	0.848316	NA
Cp19	0	NA	NA	NA	NA	NA
Cp16	0	NA	NA	NA	NA	NA
CG6576	16.94897	1.531701	3.953025	0.387476	0.698404	NA
CG32024	0	NA	NA	NA	NA	NA

CG13308	0	NA	NA	NA	NA	NA
CG13309	0	NA	NA	NA	NA	NA
Mcm7	23.63412	0.363931	3.8372	0.094843	0.92444	NA
CG13312	0.178053	-1.16467	4.075067	-0.2858	0.775028	NA
CG13311	0	NA	NA	NA	NA	NA
CG13310	0.178053	-1.16467	4.075067	-0.2858	0.775028	NA
Acbp5	0.209247	1.042804	4.047837	0.25762	0.7967	NA
CG13306	0	NA	NA	NA	NA	NA
Prm	0.71449	-2.47883	4.014121	-0.61753	0.536887	NA
Cpr66Cb	0	NA	NA	NA	NA	NA
Jon66Ci	0.178053	-1.16467	4.075067	-0.2858	0.775028	NA
mtrm	0	NA	NA	NA	NA	NA
GstO3	48.21994	-0.6997	3.843113	-0.18207	0.85553	NA
snoRNA:U4	0.528311	-1.72839	4.011871	-0.43082	0.6666	NA
snoRNA:U4	0	NA	NA	NA	NA	NA
S-Lap1	23.50303	-1.70325	3.952538	-0.43093	0.666522	NA
S-Lap2	0.178053	-1.16467	4.075067	-0.2858	0.775028	NA
Cdc6	0	NA	NA	NA	NA	NA
GstO1	0	NA	NA	NA	NA	NA
mkg-p	0	NA	NA	NA	NA	NA
Jon66Cii	0.536437	-1.71175	4.038042	-0.42391	0.671635	NA
CG33057	0	NA	NA	NA	NA	NA
fan	0	NA	NA	NA	NA	NA
ldbr	0.132078	-1.13211	4.069918	-0.27817	0.780885	NA
mus301	0.460806	-0.60052	3.997252	-0.15023	0.880581	NA
CG8006	0.578255	0.214162	3.950818	0.054207	0.95677	NA
CG8012	1.250927	-2.85877	4.004056	-0.71397	0.475246	NA
CG13678	0	NA	NA	NA	NA	NA
CG13670	0	NA	NA	NA	NA	NA
Gr66a	0	NA	NA	NA	NA	NA
CG7201	0	NA	NA	NA	NA	NA
CG7213	32.66638	1.748349	3.952495	0.442341	0.658243	NA
CG13674	0	NA	NA	NA	NA	NA
CG7366	0	NA	NA	NA	NA	NA
CG7387	0	NA	NA	NA	NA	NA
mthl7	0	NA	NA	NA	NA	NA
RecQ4	10.31535	0.546884	3.885881	0.140736	0.888078	NA
CG13675	0.697584	-1.11707	3.977017	-0.28088	0.778801	NA
CG13676	0	NA	NA	NA	NA	NA
tRNA:Leu-(	2.955291	2.974173	3.948131	0.753312	0.451263	NA
tRNA:Leu-(	0	NA	NA	NA	NA	NA
tRNA:Leu-(	0	NA	NA	NA	NA	NA
tRNA:Leu-(	0	NA	NA	NA	NA	NA
tRNA:Leu-(	0	NA	NA	NA	NA	NA
tRNA:Leu-(	0	NA	NA	NA	NA	NA
eIF4E5	0	NA	NA	NA	NA	NA
tRNA:Ala-A	0	NA	NA	NA	NA	NA
CG32371	51.73315	-2.1338	3.836203	-0.55623	0.578056	NA
mthl6	0	NA	NA	NA	NA	NA
Cyp316a1	0.178053	-1.16467	4.075067	-0.2858	0.775028	NA

frac	0	NA	NA	NA	NA	NA
CG14838	0.178812	-1.08808	4.061735	-0.26789	0.788788	NA
CG32373	0.932373	1.053996	3.996193	0.26375	0.791972	NA
PGRP-SD	0.64106	-0.7867	4.009822	-0.19619	0.844459	NA
Mcad	1.603238	-2.77841	3.960305	-0.70157	0.48295	NA
CG7409	0	NA	NA	NA	NA	NA
CG8492	0.3873	0.267327	4.008308	0.066693	0.946826	NA
CG7457	6.195187	-0.9937	3.870835	-0.25671	0.797399	NA
CG7716	0	NA	NA	NA	NA	NA
CG14837	3.978597	1.705937	3.963372	0.430426	0.666886	NA
CG32376	0	NA	NA	NA	NA	NA
CG32374	0	NA	NA	NA	NA	NA
CG16998	0	NA	NA	NA	NA	NA
CG7548	0	NA	NA	NA	NA	NA
CG8539	3.026906	-1.67104	3.963213	-0.42164	0.67329	NA
Cyp4d8	0	NA	NA	NA	NA	NA
CG14826	0	NA	NA	NA	NA	NA
mei-P22	0	NA	NA	NA	NA	NA
CG8607	54.06369	0.355682	3.835105	0.092744	0.926107	NA
mus312	0	NA	NA	NA	NA	NA
CG8563	0.836986	1.408818	3.9887	0.353202	0.723937	NA
CG32379	0.535678	-2.26616	4.021604	-0.5635	0.573098	NA
CG8562	0	NA	NA	NA	NA	NA
CG18417	0.178053	-1.16467	4.075067	-0.2858	0.775028	NA
CG33278	0	NA	NA	NA	NA	NA
Tsp66A	0	NA	NA	NA	NA	NA
CG8541	0	NA	NA	NA	NA	NA
CG8543	0	NA	NA	NA	NA	NA
sphinx2	0	NA	NA	NA	NA	NA
sphinx1	0.132078	-1.13211	4.069918	-0.27817	0.780885	NA
CG14835	0	NA	NA	NA	NA	NA
CG8560	0.233093	0.692656	4.076246	0.169925	0.865069	NA
CG8564	0	NA	NA	NA	NA	NA
CG14834	0	NA	NA	NA	NA	NA
Cln7	2.617498	1.683176	3.969392	0.424039	0.671538	NA
RhoGEF4	0	NA	NA	NA	NA	NA
CG14830	0.4188	1.41068	4.034613	0.349644	0.726606	NA
CG14829	0	NA	NA	NA	NA	NA
CG14820	0	NA	NA	NA	NA	NA
CG14823	33.69012	0.334761	3.835151	0.087288	0.930443	NA
Cpr65Ec	0	NA	NA	NA	NA	NA
BBS1	1.172943	-0.24009	3.970289	-0.06047	0.951781	NA
Acbp6	0	NA	NA	NA	NA	NA
melt	0.712972	-2.55723	4.00634	-0.63829	0.523282	NA
Acbp4	0	NA	NA	NA	NA	NA
Cpr65Eb	0.178053	-1.16467	4.075067	-0.2858	0.775028	NA
Cpr65Ea	0	NA	NA	NA	NA	NA
CG32391	0	NA	NA	NA	NA	NA
CG17744	0.356107	-1.47099	4.037764	-0.36431	0.715628	NA
CG10063	0	NA	NA	NA	NA	NA

CG10064	0.233093	0.692656	4.076246	0.169925	0.865069	NA
CG8519	0.965478	0.284617	3.909784	0.072796	0.941968	NA
Prat2	0	NA	NA	NA	NA	NA
Rsph3	6.534036	0.399922	3.95438	0.101134	0.919444	NA
SP1173	51.4057	-0.30865	3.840908	-0.08036	0.935952	NA
CG32395	0	NA	NA	NA	NA	NA
eIF4E4	0.337793	1.451235	4.039844	0.35923	0.719423	NA
CG32396	5.618481	0.256232	3.939283	0.065045	0.948138	NA
zpg	0	NA	NA	NA	NA	NA
Lcp65Ad	0	NA	NA	NA	NA	NA
Lcp65Ac	0	NA	NA	NA	NA	NA
Lcp65Ab2	0	NA	NA	NA	NA	NA
Lcp65APsi	0	NA	NA	NA	NA	NA
Lcp65Ab1	0	NA	NA	NA	NA	NA
Lcp65Aa	0	NA	NA	NA	NA	NA
Cpr65Az	0	NA	NA	NA	NA	NA
Prdm13	0	NA	NA	NA	NA	NA
CG7376	0	NA	NA	NA	NA	NA
CG7386	0.178812	-1.08808	4.061735	-0.26789	0.788788	NA
CG8219	0	NA	NA	NA	NA	NA
Or65a	0	NA	NA	NA	NA	NA
CG10163	0	NA	NA	NA	NA	NA
Best2	0	NA	NA	NA	NA	NA
Mdr65	9.063163	-1.59667	3.901916	-0.4092	0.682393	NA
CG10226	31.2421	2.010797	3.841139	0.52349	0.600633	NA
CG13299	0	NA	NA	NA	NA	NA
CG13297	0	NA	NA	NA	NA	NA
Acp65Aa	0	NA	NA	NA	NA	NA
Cpr65Ax2	0	NA	NA	NA	NA	NA
Cpr65Aw	0	NA	NA	NA	NA	NA
Cpr65Av	0	NA	NA	NA	NA	NA
CG5568	0.178053	-1.16467	4.075067	-0.2858	0.775028	NA
CG18586	0.104623	0.639075	4.067674	0.157111	0.875158	NA
CG5592	0.715249	-1.88064	4.034336	-0.46616	0.641102	NA
CG13288	15.49551	2.952995	3.924049	0.752538	0.451728	NA
yip7	0.356866	-1.92326	4.037889	-0.4763	0.633858	NA
CG6462	0	NA	NA	NA	NA	NA
Jon65Aiii	0.1047	0.613261	4.062799	0.150945	0.880019	NA
Jon65Aii	1.165466	1.076041	3.987865	0.269829	0.787292	NA
CG6592	0.356866	-1.92326	4.037889	-0.4763	0.633858	NA
CG6602	0	NA	NA	NA	NA	NA
Ets65A	33.62193	-1.20414	3.737232	-0.3222	0.747301	NA
Lcp65Ae	0	NA	NA	NA	NA	NA
Lcp65Af	0	NA	NA	NA	NA	NA
Lcp65Ag1	0	NA	NA	NA	NA	NA
Lcp65Ag2	0.178053	-1.16467	4.075067	-0.2858	0.775028	NA
Cpr65Au	0	NA	NA	NA	NA	NA
CG10469	0.132078	-1.13211	4.069918	-0.27817	0.780885	NA
CG10472	0	NA	NA	NA	NA	NA
Jon65Ai	0.53416	-1.53481	4.012395	-0.38252	0.702078	NA

CG10477	49.80582	1.710623	3.951918	0.432859	0.665117	NA
alphaKap4	0.178053	-1.16467	4.075067	-0.2858	0.775028	NA
CG10479	35.69879	-0.80072	3.862535	-0.2073	0.835773	NA
CG10486	0	NA	NA	NA	NA	NA
CG32413	0	NA	NA	NA	NA	NA
QC	0.178053	-1.16467	4.075067	-0.2858	0.775028	NA
PolE2	0	NA	NA	NA	NA	NA
Alp9	0	NA	NA	NA	NA	NA
mthl2	0.104623	0.639075	4.067674	0.157111	0.875158	NA
mad2	0.756593	2.288499	4.007508	0.571053	0.567964	NA
Tektin-C	0.104623	0.639075	4.067674	0.157111	0.875158	NA
Cralbp	0	NA	NA	NA	NA	NA
Ppat-Dpck	44.5857	4.998179	3.815108	1.310102	0.190161	NA
CG10591	18.16143	-1.70178	3.953016	-0.4305	0.666832	NA
Alp10	0.104623	0.639075	4.067674	0.157111	0.875158	NA
spo	2.704526	-0.12723	3.962037	-0.03211	0.974384	NA
CG13285	20.41649	1.744587	3.953439	0.441283	0.659008	NA
tRNA:Ser-/	0	NA	NA	NA	NA	NA
tRNA:Val-T	0	NA	NA	NA	NA	NA
blanks	0.104623	0.639075	4.067674	0.157111	0.875158	NA
Ir64a	0	NA	NA	NA	NA	NA
tRNA:Val-T	0.178053	-1.16467	4.075067	-0.2858	0.775028	NA
lama	52.53549	5.177539	3.863175	1.340229	0.180171	NA
CG4597	0	NA	NA	NA	NA	NA
CG15212	0.178053	-1.16467	4.075067	-0.2858	0.775028	NA
Gdap1	0	NA	NA	NA	NA	NA
CG10674	11.07347	0.05554	3.740588	0.014848	0.988153	NA
CG15213	0	NA	NA	NA	NA	NA
tRNA:Arg-"	14.37984	-3.72513	3.833179	-0.97181	0.331144	NA
CG1494	0.712213	-1.57119	3.998613	-0.39293	0.694369	NA
Ir20a	0.178053	-1.16467	4.075067	-0.2858	0.775028	NA
CG10918	0	NA	NA	NA	NA	NA
whe	0	NA	NA	NA	NA	NA
lcs	0	NA	NA	NA	NA	NA
snoRNA:M	0	NA	NA	NA	NA	NA
CG1801	2.166279	2.499239	3.918069	0.637875	0.523555	NA
tRNA:Ile-A	1.631653	1.102918	3.977952	0.277258	0.781582	NA
CG11666	0	NA	NA	NA	NA	NA
CG1724	0	NA	NA	NA	NA	NA
CR32511	0	NA	NA	NA	NA	NA
CG1503	37.76609	1.837408	3.89293	0.471986	0.636937	NA
CG15446	26.35188	-1.70379	3.952363	-0.43108	0.66641	NA
CG15449	0	NA	NA	NA	NA	NA
CG1304	0	NA	NA	NA	NA	NA
Ser6	0.1047	0.613261	4.062799	0.150945	0.880019	NA
CG11227	0	NA	NA	NA	NA	NA
CR11235	0	NA	NA	NA	NA	NA
CG1314	0.31387	1.261782	4.037145	0.312543	0.754628	NA
CG32507	0.233093	0.692656	4.076246	0.169925	0.865069	NA
CG15452	0	NA	NA	NA	NA	NA

CG1324	0.104623	0.639075	4.067674	0.157111	0.875158	NA
CG1631	0.178053	-1.16467	4.075067	-0.2858	0.775028	NA
Obp19a	0.357625	-1.47707	4.044794	-0.36518	0.714979	NA
Obp19b	0	NA	NA	NA	NA	NA
CG15461	1.251686	-2.06022	4.011654	-0.51356	0.60756	NA
Obp19c	0	NA	NA	NA	NA	NA
CG15458	0.104623	0.639075	4.067674	0.157111	0.875158	NA
CG15459	0	NA	NA	NA	NA	NA
jb	0	NA	NA	NA	NA	NA
CG12679	0	NA	NA	NA	NA	NA
CG1504	59.88496	-0.92728	3.834286	-0.24184	0.808905	NA
Inx6	0	NA	NA	NA	NA	NA
tilB	0	NA	NA	NA	NA	NA
Cda4	0	NA	NA	NA	NA	NA
CG14615	0	NA	NA	NA	NA	NA
CG15635	0.209247	1.042804	4.047837	0.25762	0.7967	NA
CG11929	0	NA	NA	NA	NA	NA
Elba3	0.53416	-1.53481	4.012395	-0.38252	0.702078	NA
CG15629	0	NA	NA	NA	NA	NA
Ir25a	0.3873	0.267327	4.008308	0.066693	0.946826	NA
CG3225	38.42487	1.749297	3.952259	0.442607	0.65805	NA
CG3251	0	NA	NA	NA	NA	NA
Bsg25A	0	NA	NA	NA	NA	NA
CG15429	0	NA	NA	NA	NA	NA
Traf4	0	NA	NA	NA	NA	NA
Tps1	0	NA	NA	NA	NA	NA
CG33003	0	NA	NA	NA	NA	NA
ND-B8	0.53416	-1.53481	4.012395	-0.38252	0.702078	NA
HP6	0	NA	NA	NA	NA	NA
tRNA:Trp-(	7.328995	1.727313	3.957831	0.436429	0.662525	NA
Sr-CIII	0	NA	NA	NA	NA	NA
Sr-CI	0	NA	NA	NA	NA	NA
CG2955	0	NA	NA	NA	NA	NA
lectin-24D	0.178812	-1.08808	4.061735	-0.26789	0.788788	NA
ft	25.18057	2.402066	3.924325	0.612097	0.540474	NA
Or24a	0	NA	NA	NA	NA	NA
bowl	0	NA	NA	NA	NA	NA
CG31960	0	NA	NA	NA	NA	NA
CR31958	0	NA	NA	NA	NA	NA
bark	41.52989	-4.2848	3.850407	-1.11282	0.265786	NA
TTLL4B	0.178053	-1.16467	4.075067	-0.2858	0.775028	NA
CG15422	0.178053	-1.16467	4.075067	-0.2858	0.775028	NA
CG15423	0	NA	NA	NA	NA	NA
CG31773	47.01027	1.750282	3.952013	0.442884	0.65785	NA
CG31776	18.95069	1.743818	3.953632	0.441067	0.659164	NA
Pgant4	36.49196	2.1296	3.827282	0.556426	0.57792	NA
CG8851	0	NA	NA	NA	NA	NA
IFT57	0.178812	-1.08808	4.061735	-0.26789	0.788788	NA
CG2816	0	NA	NA	NA	NA	NA
lectin-24A	0	NA	NA	NA	NA	NA

CG31955	11.51692	2.942648	3.926413	0.74945	0.453586	NA
cutlet	0.178053	-1.16467	4.075067	-0.2858	0.775028	NA
CG31777	0	NA	NA	NA	NA	NA
CG31778	0	NA	NA	NA	NA	NA
CG3604	0	NA	NA	NA	NA	NA
IM33	0	NA	NA	NA	NA	NA
CG16713	10.43414	-1.91017	3.954613	-0.48302	0.629079	NA
Acp24A4	0	NA	NA	NA	NA	NA
CG3513	0	NA	NA	NA	NA	NA
CG16704	1.71701	-1.85109	3.972145	-0.46602	0.641203	NA
CG15418	0	NA	NA	NA	NA	NA
odd	0.178812	-1.08808	4.061735	-0.26789	0.788788	NA
sob	0.104623	0.639075	4.067674	0.157111	0.875158	NA
Spindly	0	NA	NA	NA	NA	NA
Sr-CIV	0.104623	0.639075	4.067674	0.157111	0.875158	NA
CG3213	1.032167	-2.82383	3.952034	-0.71452	0.474903	NA
CG31954	0	NA	NA	NA	NA	NA
CG15414	0.590718	-0.57492	4.003363	-0.14361	0.885809	NA
Pif1	10.68396	-2.91163	3.923713	-0.74206	0.458051	NA
CG3246	0.178053	-1.16467	4.075067	-0.2858	0.775028	NA
CR31953	0	NA	NA	NA	NA	NA
tRNA:Ser-/	0	NA	NA	NA	NA	NA
CG15404	0	NA	NA	NA	NA	NA
CG15406	0	NA	NA	NA	NA	NA
CG33281	0	NA	NA	NA	NA	NA
CG33282	0	NA	NA	NA	NA	NA
CG3285	0.264155	-1.54099	4.048976	-0.38059	0.703509	NA
CG15408	0	NA	NA	NA	NA	NA
CG8838	0	NA	NA	NA	NA	NA
CG8840	0	NA	NA	NA	NA	NA
CG31952	17.27549	1.742783	3.953893	0.440776	0.659375	NA
Fign	1.246373	-1.6238	3.979526	-0.40804	0.683245	NA
OtopLb	0.178053	-1.16467	4.075067	-0.2858	0.775028	NA
CG31698	0	NA	NA	NA	NA	NA
tRNA:Ser-/	0	NA	NA	NA	NA	NA
Cpr23B	0	NA	NA	NA	NA	NA
CG2975	0.1047	0.613261	4.062799	0.150945	0.880019	NA
CG2983	0	NA	NA	NA	NA	NA
CG8813	30.01746	2.825397	3.921362	0.720514	0.471209	NA
CG17264	0.178053	-1.16467	4.075067	-0.2858	0.775028	NA
CG17219	15.78326	-4.82319	3.879528	-1.24324	0.213779	NA
snRNA:U5:	0	NA	NA	NA	NA	NA
PpD6	0	NA	NA	NA	NA	NA
CG17258	0.807012	0.227825	3.984055	0.057184	0.954399	NA
okr	0	NA	NA	NA	NA	NA
Ts	0	NA	NA	NA	NA	NA
CG3117	0	NA	NA	NA	NA	NA
CG18557	0	NA	NA	NA	NA	NA
CG18558	0	NA	NA	NA	NA	NA
CG3119	0	NA	NA	NA	NA	NA

Elba2	0	NA	NA	NA	NA	NA
CG15395	0	NA	NA	NA	NA	NA
Prosbeta4l	0	NA	NA	NA	NA	NA
Or23a	0.53416	-1.53481	4.012395	-0.38252	0.702078	NA
Prosbeta4l	0	NA	NA	NA	NA	NA
Trf4	0.1047	0.613261	4.062799	0.150945	0.880019	NA
Gr23a	0.178053	-1.16467	4.075067	-0.2858	0.775028	NA
CG2964	0.178053	-1.16467	4.075067	-0.2858	0.775028	NA
CG3123	0.53416	-1.53481	4.012395	-0.38252	0.702078	NA
CG15399	0	NA	NA	NA	NA	NA
CG9961	0	NA	NA	NA	NA	NA
CG33125	13.17699	-0.04195	3.934483	-0.01066	0.991492	NA
CG9962	0	NA	NA	NA	NA	NA
Rad1	0	NA	NA	NA	NA	NA
Cyp309a2	0.64424	-0.14539	3.993669	-0.0364	0.97096	NA
Cyp309a1	0.132078	-1.13211	4.069918	-0.27817	0.780885	NA
CG3515	0	NA	NA	NA	NA	NA
CG17234	0	NA	NA	NA	NA	NA
CG4270	0	NA	NA	NA	NA	NA
CG4271	0	NA	NA	NA	NA	NA
CG31949	20.92335	0.036905	3.932004	0.009386	0.992511	NA
CG16995	1.608552	-3.02178	4.000667	-0.75532	0.450057	NA
CG15390	12.70381	2.601219	3.852935	0.675126	0.499595	NA
dpp	0.492	1.04661	3.990653	0.262265	0.793117	NA
CG3528	0	NA	NA	NA	NA	NA
CG31948	0	NA	NA	NA	NA	NA
CG18641	0	NA	NA	NA	NA	NA
CG3557	0.387453	0.098632	4.002797	0.024641	0.980342	NA
CG3597	0	NA	NA	NA	NA	NA
robl22E	0.178053	-1.16467	4.075067	-0.2858	0.775028	NA
CG17237	0	NA	NA	NA	NA	NA
CG31681	0	NA	NA	NA	NA	NA
Send1	0	NA	NA	NA	NA	NA
CG17239	0	NA	NA	NA	NA	NA
Ser12	0	NA	NA	NA	NA	NA
Tengl1	0	NA	NA	NA	NA	NA
Tengl2	0	NA	NA	NA	NA	NA
Tengl3	0	NA	NA	NA	NA	NA
tRNA:Arg-	0	NA	NA	NA	NA	NA
Nplp4	0.1047	0.613261	4.062799	0.150945	0.880019	NA
tRNA:Gln-(	0.2094	1.005034	4.045341	0.248442	0.803792	NA
tRNA:Gln-(	0	NA	NA	NA	NA	NA
tRNA:Gly-(	0.178053	-1.16467	4.075067	-0.2858	0.775028	NA
Kebab	0	NA	NA	NA	NA	NA
Or22c	0	NA	NA	NA	NA	NA
CG12674	0	NA	NA	NA	NA	NA
tRNA:Arg-	0	NA	NA	NA	NA	NA
CG15386	0	NA	NA	NA	NA	NA
CG33124	0.71449	-2.47883	4.014121	-0.61753	0.536887	NA
CG31668	0.233093	0.692656	4.076246	0.169925	0.865069	NA

CG4259	0.233093	0.692656	4.076246	0.169925	0.865069	NA
tRNA:Gln-(	23.99654	-0.2631	3.937905	-0.06681	0.946732	NA
tRNA:Gln-(	1.981396	-0.40108	3.850773	-0.10416	0.917045	NA
tRNA:Gln-(	12.82013	1.16938	3.954559	0.295704	0.767456	NA
CG15353	0.31387	1.261782	4.037145	0.312543	0.754628	NA
Obp22a	0	NA	NA	NA	NA	NA
tRNA:Gly-(	20.12002	-1.7024	3.952811	-0.43068	0.6667	NA
Or22a	0.53416	-1.53481	4.012395	-0.38252	0.702078	NA
Or22b	0.233093	0.692656	4.076246	0.169925	0.865069	NA
CG17648	0	NA	NA	NA	NA	NA
Gr22e	0	NA	NA	NA	NA	NA
CG7295	0	NA	NA	NA	NA	NA
CG15358	1.303633	3.749586	3.93937	0.951824	0.341186	NA
c-cup	0	NA	NA	NA	NA	NA
CG31664	0	NA	NA	NA	NA	NA
CG31933	0	NA	NA	NA	NA	NA
Gr22a	0	NA	NA	NA	NA	NA
Gr22d	0	NA	NA	NA	NA	NA
CG17650	0	NA	NA	NA	NA	NA
Gr22f	0	NA	NA	NA	NA	NA
halo	0	NA	NA	NA	NA	NA
CG18132	0	NA	NA	NA	NA	NA
CG5397	0	NA	NA	NA	NA	NA
a5	0.660388	-1.75977	4.001266	-0.4398	0.660079	NA
CG5440	0	NA	NA	NA	NA	NA
Cdlc2	1.754666	-0.19119	3.90635	-0.04894	0.960963	NA
CG14346	0.132078	-1.13211	4.069918	-0.27817	0.780885	NA
snRNA:U3	0	NA	NA	NA	NA	NA
CG31928	0	NA	NA	NA	NA	NA
CG33128	0	NA	NA	NA	NA	NA
CG31926	0	NA	NA	NA	NA	NA
CG31661	0	NA	NA	NA	NA	NA
CG7420	37.72406	-6.09319	3.83485	-1.5889	0.112083	NA
CG18131	0	NA	NA	NA	NA	NA
CG31659	0	NA	NA	NA	NA	NA
CG5565	0	NA	NA	NA	NA	NA
CG31924	0	NA	NA	NA	NA	NA
CG5561	0	NA	NA	NA	NA	NA
CG5556	0	NA	NA	NA	NA	NA
CG4415	18.53189	1.743577	3.953693	0.441	0.659213	NA
ast	3.261816	-4.09457	3.920422	-1.04442	0.296291	NA
CG14339	0	NA	NA	NA	NA	NA
CG31922	18.89855	0.335597	3.734209	0.089871	0.92839	NA
CG5011	0	NA	NA	NA	NA	NA
CG14342	0	NA	NA	NA	NA	NA
CG5139	0	NA	NA	NA	NA	NA
CG5080	0.67891	-1.66339	3.984816	-0.41743	0.676362	NA
Iris	0	NA	NA	NA	NA	NA
CG4375	0	NA	NA	NA	NA	NA
CG2839	3.796853	-0.83753	3.822349	-0.21912	0.82656	NA

Hsp60B	0	NA	NA	NA	NA	NA
CG12506	0	NA	NA	NA	NA	NA
CG13946	0	NA	NA	NA	NA	NA
CG13947	0	NA	NA	NA	NA	NA
Gr21a	0.178053	-1.16467	4.075067	-0.2858	0.775028	NA
CG3544	0	NA	NA	NA	NA	NA
Nnf1b	0	NA	NA	NA	NA	NA
CG13949	0	NA	NA	NA	NA	NA
CG13950	0	NA	NA	NA	NA	NA
ninaA	0	NA	NA	NA	NA	NA
Lsp1beta	0.64424	-0.14539	3.993669	-0.0364	0.97096	NA
GluRIIC	0.104623	0.639075	4.067674	0.157111	0.875158	NA
snRNA:U1:	0	NA	NA	NA	NA	NA
CG15824	0	NA	NA	NA	NA	NA
