# Supplementary material for: Parkinson’s disease-associated PINK1 loss disrupts ensheathing glia and causes dopaminergic neuron synapse loss
Source: eLife. 2026 Aug 13;14:RP105386. doi: 10.7554/eLife.105386 (PMC13472629; doi:10.7554/eLife.105386)
Supplement: Peer review history

# Editor report: eLife Assessment

**DOI:** 10.7554/eLife.105386.3.sa0

**Authors:** Hugo J Bellen

**Affiliations:** Baylor College of Medicine United States

**Keywords:** Solid, Valuable

This **valuable** study explores the role of Pink1 in regulating mitochondria-organelle contacts and glial function, advancing our understanding of the mechanisms underlying neurodegenerative diseases. The findings highlight key genes and cellular processes that are critical in maintaining neuronal health, with implications for glial biology and Parkinson's disease research. The methodology and data are **solid**. This work will be of significant interest to researchers in neuroscience, cell biology, and neurodegenerative diseases.

# Referee report: Reviewer #1 (Public review)

**DOI:** 10.7554/eLife.105386.3.sa1

Summary:

This study investigates the impact of Pink1 loss on glial function and neuronal health in a Drosophila model, highlighting the role of mitochondria-organelle contacts and key genes such as Ccz1, Vps13, Mon1, and Rab7. The work provides insights into cellular processes underlying neurodegenerative diseases, with a focus on glia-neuron interactions.

Comments on revised version:

I have reviewed the revised manuscript and the authors' responses to previous comments. The authors have addressed the key concerns raised by the reviewers, including validation of the Mz-GAL4 line and additional control experiments. The remaining issues caused by experimental constraints are understandable in this study.

However, several concerns remain. Notably, some key results were removed due to the use of inadequately characterized fly lines, and the lack of follow-up experiments to address these issues raises concerns regarding the validity and reliability of the findings. Furthermore, the absence of experiments examining Rab7-mediated membrane trafficking or the interactions between mitochondria and lysosomes in the Pink1 mutant presents a limitation. These missing elements reduce the clarity and interpretability of Figure 5 for readers.

On a positive note, the data showing that reducing Vps35/Vps13 enhances neuronal function and rescues Pink1 mutant phenotypes in ensheathing glia contributes meaningfully to the overall narrative.

Despite these limitations, this research addresses an important question in neuroscience using the Drosophila model. It provides a novel perspective on Parkinson's disease and neurodegeneration by exploring mechanisms underlying Pink1 loss and suggesting a role for mitochondria-organelle interactions in ensheathing glia, potentially regulated via Vps35/Vps13-mediated pathways.

Overall, the current version presents a clear and meaningful contribution to the field.

# Referee report: Reviewer #2 (Public review)

**DOI:** 10.7554/eLife.105386.3.sa2

Summary:

This study proposes a novel role for ensheathing glia (EG) in a Pink1-model of Parkinson's disease and shows that this cell population exhibits the highest number of DEG in a pre-symptomatic stage. In the olfactory system, there seems to be morphological changes in this cell-type that resembles an 'activated' state and the authors further show that the neuronal loss of Pink1 is responsible for this defect. The authors go on to show that manipulation of Pink1 in EG also leads to some defects in the visual system and in the dopaminergic neurons (DAN) that innervate the mushroom body (MB), and performed a screen based on the 'on-transient' defect of the ERG to identify potential genes that may modulate the function of EG in synaptic regulation. They focus on several genes related to vesicle trafficking including Vps13, and Vps35 and performed some additional experiments in the visual system and MB to propose the role of vesicle/lipid trafficking in EG as an important factor for PD pathogenesis.

Strengths:

The study proposes functional and mechanistic connections between several genes that have been linked to PD (PINK1, VPS35 and VPS13A/C). I feel that the data presented in Figure 1-Figure 3C are performed with rigor and are convincing/novel. The selection of Drosophila to study the questions is also a strength and the lab has extensive experiences in this field and model organism.

Weaknesses:

In this revised manuscript, a number of concerns raised by this and the other reviewer was addressed. The authors now admitted that some of the genetic reagents used in their screen and follow up assays were inappropriately utilized, and changed the latter half of the paper (Fig 3D-F4) quite significantly (e.g. now only 1 gene is considered as a hit in Fig3D, analysis of several genes in Fig4 have been removed and replaced by some experiments performed on Vps35). The transition between Figure 3D and Figure 4 is quite abrupt, and they don't seem to follow up on the CG17660 (the single hit from their screen, which is not further validated so it is not clear whether this genetic reagent is clean or not) and the effect of Vps35 RNAi in synaptic phenotype. Therefore, there is still a weakness in Figure 3D-Figure 4, which weakens the paper, especially since the new model diagram the authors provided in Figure 5 is not really investigated at the molecular level.

# Author comment: Author response

**DOI:** 10.7554/eLife.105386.3.sa3

**Authors:** Lorenzo Ghezzi, Sabine Kuenen, Ulrike Pech, Nils Schoovaerts, Ayse Kilic, Suresh Poovathingal, Kristofer Davie, Jochen Lamote, Roman Praschberger, Patrik Verstreken

**Affiliations:** VIB-KU Leuven Center for Brain and Disease Research Leuven Belgium; VIB-KU Leuven Center for Brain and Disease Research Leuven Belgium; VIB-KU Leuven Center for Brain and Disease Research Leuven Belgium; VIB-KU Leuven Center for Brain and Disease Research Leuven Belgium; VIB-KU Leuven Center for Brain and Disease Research Leuven Belgium; VIB-KU Leuven Center for Brain and Disease Research Leuven Belgium; KU Leuven Leuven Belgium; VIB-KU Leuven Center for Brain and Disease Research Leuven Belgium; VIB-KU Leuven Center for Brain and Disease Research Leuven Belgium; VIB-KU Leuven Center for Brain and Disease Research Leuven Belgium

The following is the authors’ response to the original reviews.

**Public Reviews:**

**Reviewer #1 (Public review):**

Summary:

This study investigates the impact of Pink1 loss on glial function and neuronal health in a Drosophila model, highlighting the role of mitochondria-organelle contacts and key genes such as Ccz1, Vps13, Mon1, and Rab7. The work provides insights into cellular processes underlying neurodegenerative diseases, with a focus on glia-neuron interactions. While the findings are promising, the study lacks critical controls, detailed mechanistic evidence, and explanatory figures to strengthen its claims.

Strengths:

(1) The study addresses an important topic in neuroscience, exploring the mechanisms of Pink1 loss, which has implications for Parkinson's disease and neurodegeneration.

(2) The focus on mitochondria-organelle contacts and their regulation by Rab7-mediated pathways is novel and provides a potential mechanism for neuronal dysfunction.

(3) The identification of key genes (Ccz1, Vps13, Mon1, Rab7) and their potential roles in Pink1-related pathways adds valuable knowledge to the field.

(4) The manuscript uses a combination of genetic tools, Drosophila models, and functional assays to approach the problem from multiple angles.

Weaknesses:

(1) Specificity of Mz-Gal4: The study lacks validation of Mz-Gal4 specificity, as it may also drive expression in a few neurons or other types of glia. Additional control experiments using nls-GFP with Elav, Repo, or Draper antibody staining or alternative glial drivers would be helpful.

We have addressed this issue of Gal4 driver specificity based on new experiments in the revised manuscript.

(2) DLG staining is central to the story but is not well-supported by high-resolution Z-stack imaging, which should be included in the supplementary figures.

We have included these in the supplement.

(3) The manuscript does not confirm whether the candidate RNAi (Ccz1, Vps13, Mon1, Rab7) directly influence Rab7-mediated membrane trafficking or mitochondria-lysosome contacts in Pink1 mutants.

This is indeed the case. These more mechanistic experiments were not yet performed.

(4) Using ERG as a readout for EG effects in the antenna is not a direct or appropriate assay. Alternative functional assays relevant to antenna glia should be considered.

We made the assumption that ensheating glial function is conserved across brain regions and now make this explicit in the reworded manuscript.

(5) A graphical explanation of the interactions and functions of the candidate genes in Pink1 KO mutants is missing. This would greatly enhance the manuscript's clarity.

We have included such a scheme in the new manuscript.

(6) The study lacks details on sample sizes, effect sizes, and reproducibility, which are necessary for robust conclusions.

We have included these essential data in the reworked document.

(7) There are repeated words on page 3 ("olfactory Olfactory Receptor Neurons") and a lack of explanation in Figure 3C regarding the most up-regulated and down-regulated genes and the significance of large red dots.

We have included the requested information.

**Reviewer #2 (Public review):**

Summary:

This study proposes a novel role for ensheathing glia (EG) in a Pink1-model of Parkinson's disease and shows that this cell population exibits the highest number of DEG in a pre-symptomatic stage. In the olfactory system, there seems to be morphological changes in this cell-type that resembles an 'activated' state and the authors further show that the neuronal loss of Pink1 is responsible for this defect. The authors go on to show that manipulation of Pink1 in EG also leads to some defects in the visual system and in the dopaminergic neurons (DAN) that innervate the mushroom body (MB), and performed a screen based on the 'on-transient' defect of the ERG to identify potential genes that may modulate the function of EG in synaptic regulation. They focus on several genes related to Rab7/Vps13, and performed some additional experiments in the visual system and MB to propose the role of vesicle/lipid trafficking in EG as a important factor for PD pathogenesis.

Strengths:

The study proposes functional and mechanistic connections between several genes that have been linked to PD (PINK1, VPS13A/C). I feel that the data presented in Figure 1 and Fig3A-C are performed with rigor and are convincing/novel. The selection of Drosophila to study the questions is also a strength and the lab has extensive experiences in this field and model organism.

Weaknesses:

There is one fundamental concern I have with the genetic experiments performed in this paper (especially in Fig 3D and Fig4, see major issue #1), and I feel that there is a bit of a disconnect between the EG 'activation' phenotype the author show in the olfactory system and the other two neuronal systems (visual system, MB DAN) that the authors investigate see major issue #2. Also, there are quite a bit of information that is not provided in the manuscript (see major issues #3 and #4), which makes me difficult to judge the rigor and interpretation of several experiments.

Major Concern #1: A number of lines used in this study are referred to as "RNAi" lines but when I look at the actual genotypes of reagents listed in the table in the METHODS section, many are actually NOT RNAi lines. Quite a few lines, including lines that the authors use as RNAi against Ccz1, Rab7 and Mon1, are gRNA lines for the TKO (TRiP-CRISPR knockout) system. While these reagents can theoretically knock-out these genes in somatic cells if used in combination with UAS-Cas9, there is no mention that UAS-Cas9 was used in this work throughout the manuscript. Hence, when these lines are just crossed to GAL4 with or without the Pink1 mutant, they shouldn't be having any effects. Similarly, the strongest hit from their screen was a TOE (TRiP-CRISPR Over Expression) gRNA against PIG-A, which could allow overexpression of PIG-A if there is a UAS-dCas9::VP64. However, I also do not see any mention that such activator was introduced into the crossing scheme. Considering that 3 of the 4 'hits' from their screen are not RNAi lines, I am quite skeptical of the study. Similarly, except for Vps13, all reagents used in Fig4 are TKO gRNA lines. Therefore, if this experiment was conducted without an UAS-Cas9, most of the data shown here are problematic. Also, note that several of the 'RNAi' lines listed in the Table in the METHODS section are actually MiMIC alleles. While some MiMIC lines could function as strong LOF alleles (if they are inserted in the exon or in an intron of the gene in the same orientation as the gene), some of the lines are not expected to affect gene function (e.g. FASN2 and CG17712, MiMICs are in introns and face the opposite orientation). Hence, the rationale of including these reagents in the screen doesn't make much sense. The description of the modifier screen should be much more detailed in the RESULTS and METHODS section and if the UAS-Cas9/dCas9::VP64 transgenes were not introduced when the TKO/TOE reagents were utilized, what can be concluded?

In addition, for the 4 genes that the authors further study in Fig4, there are many other reagents that the authors can use, including mutant alleles, previously characterized RNAi lines (e.g. Vps13) and dominant negative/constitute active lines (e.g. especially for Rab7). The authors should validate their results with independent reagents to really convincingly show that the same conclusions can be drawn for the Vps13/Rab7 related genes since this is the key takeaway message of this paper.

Also, they do not show whether the manipulation of these genes in a wild-type background (they only show what happens in Pink1 mutants) affect ERG and MB DAN synapse morphology. If these manipulations alone dramatically affect these phenotypes, it would be very difficult to interpret their data.

We sincerely thank the reviewer for spotting this major oversight regarding the use of the TKO (TRiP-CRISPR knockout) and TOE (TRiP-CRISPR Over Expression) systems and the MiMIC alleles. As the reviewer pointed out, these lines were not used as intended, therefore our results and conclusions regarding the genetic interactions between Pink1 and several genes (PIG-A, Rab7, Ccz1, CG10646, Mon1, FASN2, CG17712), are incorrect and based on a technical mistake. These results were removed from the manuscript. While our mistake compromises the data regarding PIG-A, Rab7, Ccz1, CG10646, Mon1, FASN2, CG17712, it does not affect the results and conclusions for most of the genes of the screening and for Vps13 where we did use RNAi lines.

Also, in the reworked manuscript, we provide additional evidence that modulation of vesicle trafficking proteins involved in mitochondria–endoplasmic reticulum (ER) membrane interactions, such as Vps13 and Vps35, influences neuronal function and rescues *Pink1* mutant phenotypes when selectively downregulated in EG.

Major Concern #2: In Figure 1, the authors show some morphological evidence that EG are 'activated' in Pink1 mutants, but whether the same phenomenon occurs in the visual system and in the MB is not shown. Since all of the studies in Fig3D and Fig4 are done in the visual system and MB, it is not clear whether the visual system and MB phenotypes are related to 'activation' of EG.

Also, in the RNA-seq data in Fig1A and Fig3C, is there any molecular evidence that EG are indeed 'activated'? The only evidence that the authors show to state that EG are 'activated' in young Pink1 null animals is based on increased CD8::GFP staining in the olfactory system.

The authors cannot draw a strong conclusion that indeed EG are 'activated' based on these data (e.g. perhaps the expression level of CD8::GFP is just increased). Additional evidence that the EG are 'activated' could be provided by looking at the increase in Draper intensity (as reported by Doherty et al. and MacDonald et al. that the authors cite), not only in the olfactory system, but also in the visual system and in the MB. It would also be informative if the authors can look at morphology of the EG in the visual system and MB to convincingly that the data shown in Fig4 is relevant to EG 'activation'.

In line with the identification of DEG across the ensheating glia cluster in our single cell sequencing (where we did not distinguish between EG of different brain regions) we made the assumption that EG-(dys) function is consistent in the *Pink1* mutant and conserved across brain regions. Nonetheless, to make clear that we did not consistently analyze EG morphology in the different brain regions that we probed in functional assays, we added a note in the manuscript. Furthermore, we also toned down our conclusion that the EG in *Pink1* mutants are in an activated state: we note the similarity in phenotype in *Pink1* mutants and situations of neuronal damage (where EG are activated) but added that the phenotype in *Pink1* mutants may also be the result of the mere upregulation of GFP expression/fluorescence.

Major Concern #3: In Fig3, there is no clear explanation why they focus on the ON transients and ignore the OFF transients, and also why the difference in the depolarization is not quantified in Fig4.

We included this explanation in the reworked manuscript: In the *Drosophila* ERG, the sustained depolarization primarily reflects phototransduction in photoreceptors (and is defective when photoreceptors degenerate), whereas the ON and OFF transients arise from second-order lamina neurons and are widely used as readouts of signal transfer. We wanted to assess function and focused on the ON transient because in general it provides an onset-locked, more robust readout of function (Vilinsky & Johnson, 2012).

Major Concern #4: While the authors claim that mz709-GAL4 is a EG specific driver, do the authors know that this is indeed true in the tissues and stages that are studied here? The Ito et al,. paper that is cited in the METHOD section has only looked at the expression of this reporter in embryonic and larval stages. The authors need to that the authors should validate their findings with an additional EG specific driver and/or provide additional data that mz709-GAL4 is indeed specific to EG in the adult fly brain and eye. If mz709-GAL4 is expressed in other cell-types, the interpretation of many of the data in this paper becomes quite questionable. I believe the data in Fig3B is suggesting that mz709-GAL4 is indeed specific to glia cells and not expressed in neurons, but whether this driver is truly specific to EG (and not in other glial types), especially in the visual system (including the lamina as well as in the eye), is not obvious.

We labelled animals that express *UAS-HisTag-eGFP* (used also in our paper) under control of *MZ709-Gal4* with anti-Elav (a neuronal marker) and find no significant overlap (see below “recommendation for authors”), consistent with *MZ709-Gal4* not driving expression in neurons. This is consistent with previous published work: Indeed, *MZ709-Gal4* has been amply used in adult flies and shown to be ensheating glia-specific (Doherty et al., 2009; Li et al., 2023; Sehgal et al.,2018). In the lamina neuropil of the *Drosophila* eye, MZ709-Gal4 is expressed in the marginal glia (Stenesen et al., 2019) which are neuropil-associated glia and are equivalent to generic ensheathing glia (Kremer et al., 2017). *MZ709-Gal4* is also expressed also in satellite glia (Stenesen et al., 2019), but these glia enwrap the cell bodies of the lamina neurons and not the neuropil where synapses reside.

**Recommendations for the authors:**

**Reviewing Editor Comments:**

We strongly encourage you to very carefully edit this manuscript. The reviewers made many probing comments that you should consider carefully.

**Reviewer #1 (Recommendations for the authors):**

(1) Validate the specificity of Mz-Gal4 by performing experiments with nls-GFP and Elav antibody staining to ensure there is no neuronal overlap. Additionally, consider using alternative glial-specific drivers, such as Repo-Gal4 or WG-Gal4, to confirm the findings.

We expressed *HisTag-eGFP* (used also in our paper) under control of *MZ709-Gal4* and labelled fly brains with anti-Elav (a neuronal marker). We do not observe significant overlap between the labels indicating *MZ709-Gal4* does not express Gal4 in neurons (Supplementary figure 1).

As indicated, these observations are consistent with previous published work. *MZ709-Gal4* has been amply used in adult flies and shown to be ensheating glia-specific (Doherty et al., 2009; Li et al., 2023; Sehgal et al., 2018; Stahl et al., 2018). In the lamina neuropil of the *Drosophila* eye, *MZ709-Gal4* is expressed in the marginal glia (Stenesen et al., 2019) which are neuropil-associated glia and are equivalent to generic ensheathing glia (Kremer et al., 2017). *MZ709-Gal4* is also expressed also in satellite glia (Stenesen et al., 2019), but these glia enwrap the cell bodies of the lamina neurons and not the neuropil where synapses reside.

(2) Include high-resolution Z-stack imaging of DLG staining to strengthen the assessment of synaptic integrity and ensure the robustness of the conclusions. These images should be added to either the main or supplementary figures.

We included 2 supplementary figures (2 and 3) showing Z stacks that were used to delineate regions of interest at the MBs for the quantification of dopaminergic neuron afferents invasion. Our approach is identical to the one we used in Kaempf et al. 2026 (Kaempf et al., 2026).

(3) Demonstrate whether the candidate RNAi (Ccz1, Vps13, Mon1, Rab7) directly influence Rab7-mediated membrane trafficking or mitochondria-lysosome contacts in Pink1 mutants. Use an appropriate method to confirm changes in organelle contacts in response to the RNAi treatments.

Ccz1, Mon1 and Rab 7 were removed due to the technical mistake we made. We did confirm and maintain that Vps35 and Vps13 downregulation in EG rescues neuronal defects in *Pink1* mutants. In the reworked manuscript we present a possible mechanism that involves the role of Vps35 and Vps13 in regulating ER-mitochondrial contacts, in line with our previous work (Valadas et al., 2018), while not ruling out possible other mechanisms.

(4) Provide an alternative functional assay or evidence to support the use of ERG as a readout for EG effects in the antenna. Consider using a more direct assay relevant to antenna glia function.

We agree that a more direct functional assay of antennal glia would be a nice addition (e.g., single-sensillum recordings or glial/ORN Ca^2+^ imaging). However, implementing such assays would require new experimental pipelines and substantial additional data generation that is beyond our current ability and the scope of this revision.

(5) Add a graphical illustration explaining the proposed mechanism of how Ccz1, Vps13, Mon1, and Rab7 function in Pink1 KO mutants, highlighting their interactions and roles within specific cell types.

We included a schematic of our working model in Figure 5.

(6) Clarify Figure 3C by explaining the most up-regulated and down-regulated genes and the significance of the large red dots. This will enhance the interpretability of the data.

We expanded the legend to this figure: The large red dots represent the genes that rescue *Pink1^KO-WS^* phenotype when downregulated, the dark green dots are the 50 top most deregulated genes (magnitude of deregulation) in EG in *Pink1^KO-WS^* compared to controls, while the light green dots represent whole the genes detected in our cell-type specific transcriptomic experiment.

(7) Correct repeated words on page 3 ("olfactory Olfactory Receptor Neurons") for clarity and consistency.

Of course, sorry for this.

(8) Ensure that sample sizes, effect sizes, and the number of replicates are explicitly stated for all experiments. This information is essential for evaluating the robustness and reproducibility of the findings.

We made sure we consistently added all this information in the revised manuscript.

(9) Verify and ensure that all data, reagents, and code used in the study are accessible and appropriately documented, in adherence with eLife's publishing policies.

We made sure all data, reagents and code are available and/or properly described.

By addressing these recommendations, the authors will significantly improve the clarity, rigor, and reproducibility of the manuscript.

**Reviewer #2 (Recommendations for the authors):**

Minor Points.

(1) All figures seem to lack titles.

We fixed this error.

(2) In the abstract, the authors say that Rab7 and Vps13 are mutated in PD patients but I couldn't find the reference/information for Rab7 (the authors do refer to papers that linked VPS13A/C variants to PD but no mention about RAB7A/B being linked to PD). Please discuss this in the paper or modify the abstract accordingly.

We removed this statement for rab7 from the paper.

(3) When referring to the human gene, Pink1 should be written as PINK1 according to the HGNC nomenclature rules.

We made this change.

(4) The authors say Vps13 has two mammalian orthologs but actually it has four (VPS13A/B/C/D). I guess two of the four is linked to PD so the authors should modify there statement to reflect this.

This is a misinterpretation of what we meant and we have clarified our intention: *Drosophila* possesses 3 paralogues of Vps13 - Vps13, Vps13B, and Vps13D - which we also detected in our screening (Neuman et al., 2025; Velayos-Baeza et al., 2004; Vonk et al., 2017). Among these Vps13 is most similar to human VPS13A and VPS13C (Hanna et al., 2023; McEwan & Ryan, 2022).

(5) The abbreviation 'CNS' is used in the first page of the intro but I don't see it being spelled out as "central nervous system".

We have spelled out central nervous system in the first page of the introduction.

(6) On the top of page 5, the authors state that they confirmed that the 'synaptic area of DAN show a decrease in aged (25 days) animals' but data is not shown. If they want to make a statement like this, I believe such data should be included in supplemental data. Since the phenotype in the aged animal is not relevant to this study, one could remove this statement regarding the aged animals if they prefer not to show the data.

The decreased synaptic area of DAN in 25-day old *Pink1* mutants is shown in figure 2C-D of the manuscript and is consistent with data shown in (Kaempf et al., 2026).
